# Catalytic Asymmetric Difluoroalkylation Using In Situ Generated Difluoroenol Species as the Privileged Synthon

**DOI:** 10.1002/advs.202307520

**Published:** 2024-02-06

**Authors:** Xiongda Xie, Shanliang Dong, Kemiao Hong, Jingjing Huang, Xinfang Xu

**Affiliations:** ^1^ School of Pharmaceutical Sciences Sun Yat‐sen University Guangzhou 510006 P. R. China; ^2^ School of Chemistry Sun Yat‐Sen University Guangzhou Guangdong 510275 P. R. China

**Keywords:** 1,2‐difunctionalization, asymmetric catalysis, difluoroalkylation, metal carbene, multi‐component reaction

## Abstract

A robust and practical difluoroalkylation synthon, α,α‐difluoroenol species, which generated in situ from trifluoromethyl diazo compounds and water in the presence of dirhodium complex, is disclosed. As compared to the presynthesized difluoroenoxysilane and in situ formed difluoroenolate under basic conditions, this difluoroenol intermediate displayed versatile reactivity, resulting in dramatically improved enantioselectivity under mild conditions. As demonstrated in catalytic asymmetric aldol reaction and Mannich reactions with ketones or imines in the presence of chiral organocatalysts, quinine‐derived urea, and chiral phosphoric acid (CPA), respectively, this relay catalysis strategy provides an effective platform for applying asymmetric fluorination chemistry. Moreover, this method features a novel 1,2‐difunctionalization process via installation of a carbonyl motif and an alkyl group on two vicinal carbons, which is a complementary protocol to the metal carbene *gem*‐difunctionalization reaction.

## Graphical abstract

An enantioselective three‐component difluoroalkylation reaction using an in situ formed α,α‐difluoroenol species as the key difluorinated synthon is reported. This method features a 1,2‐difunctionalization process, which is a complementary protocol to the metal carbene *gem*‐difunctionalization reaction. The disclosed relay catalysis strategy provides an effective platform for expanding asymmetric fluorination chemistry.

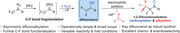

## 1 Introduction

The growing interest in selective synthesis of fluorinated compounds is evident based on their prevalence in natural products, agrochemicals, and pharmaceuticals.^[^ ^1^ ^]^ Thus, a variety of catalytic methods have been disclosed,^[^ ^2^, ^3^ ^]^ including radical processes,^[^ ^4^ ^]^ nucleophilic^[^ ^5^ ^]^ or electrophilic additions,^[^ ^6^ ^]^ and other transformations.^[^ ^7^ ^]^ In this area, an appealing task is the introduction of a difluoromethylene unit,^[^ ^8^ ^]^ which has demonstrated broad applications in medicinal chemistry and material science.^[^ ^9^ ^]^ Especially, the α,α‐difluoroketone moiety has been found in a broad spectrum of bioactive molecules with unique physicochemical properties that are useful for developing pharmaceuticals and probes for chemical biology.^[^ ^10^ ^]^ However, the difluoroalkylation has rarely been documented, which might be mainly due to the limited accessibility of fluorinated reagents that could be used as practical precursor/synthon for the difluoroalkylation.^[^ ^11^ ^]^ On the other hand, the selective cleavage of one C─F bond in the commonly used trifluoromethyl species and subsequent functionalization remains a formidable challenge, because of the inherent inertness of C─F bond and the decrease in bond dissociation energy (BDE) as defluorination occurs, resulting in undesired over‐defluorination.^[^ ^12^ ^]^

**Figure 1:**
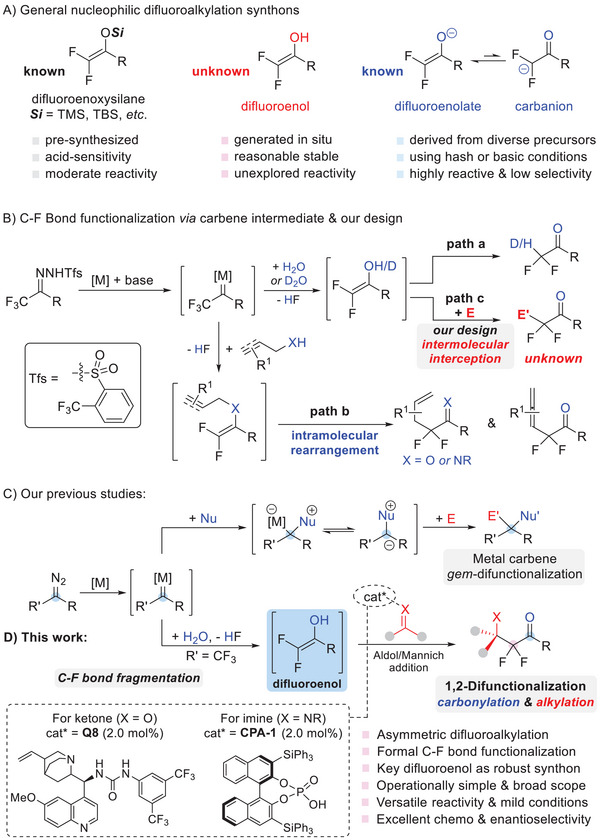
General synthons for the difluoroalkylation and our tactic for asymmetric C‐F bond functionalization. A) General nucleophilic difluoroalkylation synthons. B) C─F Bond functionalization via carbene intermediate & our design. C) Catalytic metal carbene gem‐difunctionalization. D) This work: Asymmetric difluoroalkylation via a C─F bond fragmentation and electrophilic addition sequence.

In the past decade, difluoro enol silyl ethers (difluoroenoxysilanes) have been recognized as versatile synthons for the construction of difluoroalkylated molecules through addition with a variety of electrophiles (**Figure** **1**A, left).^[^ ^13^, ^14^, ^15^ ^]^ Although relatively stable, the prepreparation, acid‐sensitivity, and moderate reactivity are the key obstacles in this method. By contrast, difluoroenolates are much more reactive species, which could be generated in situ from different types of prenucleophiles (Figure 1A, right),^[^ ^16^ ^]^ however, the reaction of difluoroenolates with carbonyl compounds has proved to be complicated and the asymmetric induction has remained a major challenge in this area.^[^ ^17^ ^]^ In this context, it is highly desirable to develop an effective catalytic method with practical difluoroalkylation precursors to overcome the above mentioned limitations, especially enabling the catalytic asymmetric difluorocarbonylation with a robust difluorinated synthon.

Recently, Bi's group has disclosed a novel C─F bond functionalization protocol using trifluoromethyl hydrazone as the fluorinated reagent (Figure 1B).^[^ ^18^ ^]^ In the presence of metal catalyst under basic conditions, these reactions initially form the metal carbene species, then C─F bond cleavage occurs to give the difluoroenol or its analog, and terminated by protonation (path a)^[^ ^18a^ ^]^ or intramolecular rearrangement (path b)^[^ ^18b,c^ ^]^ providing the α,α‐difluoroketone derivatives through a controllable mono─C─F bond functionalization process. Inspired by these advances^[^ ^13^, ^14^, ^15^, ^16^, ^17^, ^18^ ^]^ and in line with our ongoing interest in metal carbene *gem*‐difunctionalization via interception of in situ formed intermediates (Figure 1C),^[^ ^19^, ^20^ ^]^ we wondered whether a novel difluoroalkylation reaction using α,α‐difluoroenol (Figure 1A, middle) as the key synthon could be designed via an intermolecular electrophilic trapping process (Figure 1B, path c). If realized, such an unprecedented interception process might represent a complementary 1,2‐difunctionalization method for direct assembly of difluoromethylene incorporated architectures by installing a carbonyl motif and an alkyl group sequentially. Herein, we disclose the successful establishment of this goal, culminating in a practical and robust enantioselective difluoroalkylation method using an in situ formed difluoroenol species as the key fluoridated synthon under mild conditions (Figure 1D). This species has shown versatile reactivity and dramatically improved enantioselectivity as demonstrated in catalytic asymmetric aldol reaction and Mannich reactions with ketones or imines in the presence of chiral organocatalysts, quinine‐derived urea and chiral phosphoric acid CPA, respectively. In comparison to our previous studies on metal carbene *gem*‐difunctionalization reaction,^[^ ^21^ ^]^ this method features a novel 1,2‐difunctionalization process by introducing two different functionalities on the two vicinal carbons (Figure 1C vs 1D).

## 2 Results and Discussion

We commenced our studies using trifluoromethyl diazo compound **1a**, water **2a**, and isatin **3a** as model substrates for the optimization of reaction conditions (**Table** **1**). Initially, different solvents were evaluated in the presence of 2.0 mol% of Rh_2_(OAc)_4_ at 30 °C with 10 mol% of quinine‐derived squaramide catalyst **Q1** as the chiral organocatalyst (entries 1–5), which proved to be effective in our previous study for the asymmetric aldol‐type addition with isatin.^[^ ^20a^ ^]^ Tetrahydrofuran (THF) was identified as the better solvent in term of stereoselectivity, forming the difluoroalkylated product **4a** in 68% yield with 65% *ee* (entry 2), and higher yield was obtained when the reaction was conducted in ethyl acetate (entry 3, 85% yield). Then, the screening of a variety of quinine‐derived chiral organocatalysts **Q2–Q6** in THF found that 3,5‐di‐CF_3_ substituted aniline derived catalysts gave better results in terms of both yield and enantioselectivity (entries 6–10, and see Figure S1 and Table S2, Supporting Information). Further optimization of the organocatalysts by replacing the quinine or squaramide parts with chiral cyclohexanediamine or urea motifs (entries 11 and 12) resulted in that the quinine‐derived urea catalyst **Q8** giving the desired product **4a** in 72% yield with 93% *ee* (entry 12). The yield was improved to 95% by increasing the ratio of diazo compound **1a** and water to 1.5 equivalents (entry 13), while the excellent reactivity and stereoselectivity was retained by reducing the loading of both the dirhodium tetraacetate and organocatalyst **Q8** (see Table S3, Supporting Information for details). Only inferior results were observed when other metal catalysts were used instead of Rh_2_(OAc)_4_ (see Table S3, Supporting Information for details). The best results were obtained by using 1.0 mol% of Rh_2_(OAc)_4_ and 2.0 mol% of **Q8** in THF at 30 °C, which delivered the difluoroalkylated product **4a** in 95% yield with 93% *ee* (entry 14). Moreover, this reaction could also be conducted with triethylsilanol **2b** instead of water **2a**, which formed the desilylation product **4a** in 95% yield with 94% *ee* (entry 15).

Under the optimized conditions, the substrate scope with respect to the isatins **3** was explored and the results were shown in **Table** **2**. A variety of *N*‐substituted isatins **3a**–**3e** were found to provide the aldol addition products **4a**–**4e** in 88%–95% yields with ≥90% *ee*. Notably, when non‐protected isatin (**3f**) was used, the desired product **4f** was obtained in high yield with elegant enantioselectivity (95% *ee*). Furthermore, the reaction with isatins containing an electron‐donating or electron‐withdrawing group on the different positions of the aryl motif all proceeded smoothly, delivering the corresponding products **4g**–**4v** in high yields with excellent enantioselectivity (92%–96% *ee*). With electron‐deficient isatins, 10 mol% of chiral organocatalyst loading was used to retain the excellent stereoselectivity, whereas, 20 mol% of catalyst loading was used in previous studies when only 78% *ee* was achieved for the nitro‐substituted product **4r** through a decarboxylative aldol reaction^[^ ^17c^ ^]^ (vs our results in 95% *ee*). The absolute stereochemistry of **4a** was determined as *S* using X‐ray crystallography, and the other products were similarly assigned by analogy.^[^ ^22^ ^]^

**Table 1:** Condition optimization.

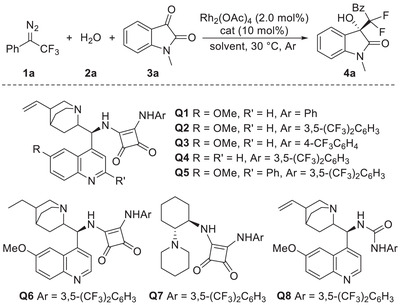				
Entry^a)^	Solvent	Cat	Yield [%]^b)^ 4a	*ee* [%]^c)^
1	DCM	Q1	42	54
2	THF	Q1	68	65
3	EA	Q1	85	60
4	PhCl	Q1	–	–
5	TBME	Q1	11	55
6	THF	Q2	71	85
7	THF	Q3	73	71
8	THF	Q4	65	82
9	THF	Q5	75	83
10	THF	Q6	66	82
11	THF	Q7	67	60
12	THF	Q8	72	93
13^d)^	THF	Q8	95	93
14^d)^, ^e)^	THF	Q8	95	93
14^d)^, ^e)^, ^f)^	THF	Q8	95	94

^a)^ The reaction was carried out on a 0.1 mmol scale: to the mixture of Rh_2_(OAc)_4_ (0.45 mg, 2.0 mol%), **2a** (0.1 mmol), **3a** (0.1 mmol), and organocatalyst (10 mol%) in the indicated solvent (1.0 mL), was added a solution of diazo compound **1a** (0.1 mmol) in the same solvent (1.0 mL) via syringe pump over 1 h under an argon atmosphere at 30 °C, and the reaction mixture was stirred for an additional 1 h under these conditions;

^b)^ Isolated yields;

^c)^ Determined by chiral HPLC analysis, see SI for detail;

^d)^ The reaction was conducted with **1a** (0.15 mmol, 1.5 equiv.) and **2a** (0.15 mmol, 1.5 equiv.);

^e)^ The reaction was conducted with reduced catalyst loading: 1.0 mol% of Rh_2_(OAc)_4_ and 2.0 mol% of **Q8**;

^f)^ The reaction was conducted with triethylsilanol **2b** instead of water, which formed the desilylation product **4a**.

DCM = dichloromethane; EA = ethyl acetate; TBME = methyl *tert*‐butyl ether.

**Table 2:** Substrate scope of isatin **3**.^a)^

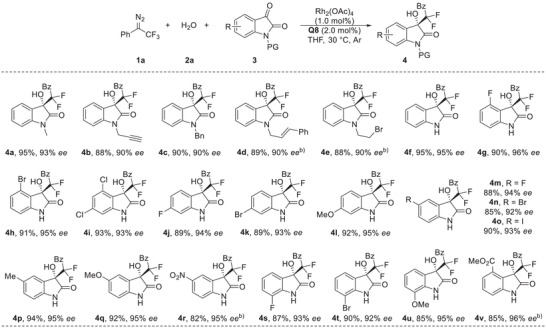

^a)^ The reaction was carried out on a 0.1 mmol scale: to the mixture of Rh_2_(OAc)_4_ (0.45 mg, 1.0 mol%), water **2a** (2.7 µL, 0.15 mmol), **3** (0.1 mmol), and **Q8** (1.2 mg, 2.0 mol%) in THF (1.0 mL), was added a solution of diazo compound **1a** (27.9 mg, 0.15 mmol) in THF (1.0 mL) via syringe pump over 1 h under an argon atmosphere at 30 °C, and the reaction mixture was stirred for an additional 1∼2 h under these conditions;

^b)^ The reaction was conducted with 10 mol% of **Q8** loading.

Then, a series of trifluoromethyl diazo compounds **1** with different substitutions at *para*‐substitution of the phenyl ring, including F, Br, Me, OMe, or CF_3_ all proceeded smoothly to afford the products **5a**–**5e** in 82%–92% yields with 91%−95% *ee* (**Table** **3**). The *meta*‐ and *ortho*‐substituted aryl diazo compounds also gave products **5f** and **5** **g** in high yields with 94% and 91% *ee*, respectively. For the formation of **5** **g**, 10 mol% of organocatalyst loading was used to retain its excellent stereoselectivity. In addition, 2‐naphthyl or 5‐benzofuranyl substituted diazo compounds were well tolerated under current conditions, producing **5** **h** and **5i** in high yields and excellent enantioselectivities. Notably, the alkyl substituted products **5j** and **5k**, which are a challenge to be obtained with high reactivity and selectivity by using reported method with the corresponding difluoroenoxysilane as the difluorinated nucleophile,^[^ ^23^ ^]^ was smoothly formed in high yields with ≥90% *ee*.

**Table 3:** Substrate scope of diazo compound **1**.^a)^

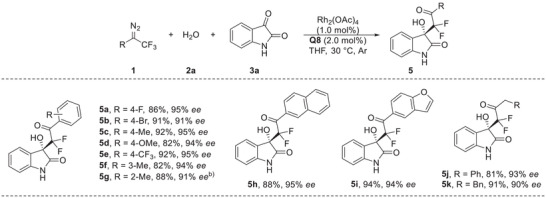

^a)^ The reaction was carried out on a 0.1 mmol scale: to the mixture of Rh_2_(OAc)_4_ (0.45 mg, 1.0 mol%), **2a** (2.7 µL, 0.15 mmol), **3a** (14.7 mg, 0.1 mmol), and **Q8** (1.2 mg, 2.0 mol%) in THF (1.0 mL), was added a solution of diazo compound **1** (0.15 mmol) in THF (1.0 mL) via syringe pump over 1 h under an argon atmosphere at 30 °C, and the reaction mixture was stirred for an additional 1∼2 h under these conditions;

^b)^ The reaction was conducted with 10 mol% of **Q8** loading.

Encouraged by above promising results, we turned our attention into the evaluation of asymmetric difluorocarbonylation with imines. After optimization of conditions (see Table S4, Supporting Information for details), this protocol was extended to Mannich‐type addition by using Rh_2_(esp)_2_ (1.0 mol%) as the metal catalyst, chiral phosphoric acid **CPA‐1** (2.0 mol%) as the organo cocatalyst, delivering the difluoroalkylated amino product **7a** in 95% yield with 94% *ee* (Table S4, Supporting Information, entry 4). Under these optimized conditions the substrate scope with respect to the imines **6** was investigated (**Table** **4**). A variety of substituents at different positions of two aryl rings were all well tolerated, leading to the corresponding products **7a‐**‐**7j** in 81%–95% yields with 90%–95% *ee*. When the imines **6k** and **6l** were used, in which the oxygen of the reactant (X in **6**) was replaced with a sulfur atom or a methylene unit as linkage, leading to the products **7k** and **7l** were formed in 95% and 78% yields, respectively, also with high enantioselectivity has been maintained well. The absolute stereochemistry of **7a** was determined as *R* using X‐ray crystallography, and the other products were assigned by analogy.^[^ ^22^ ^]^

**Table 4:** Substrate scope for the synthesis of **7**.^a)^

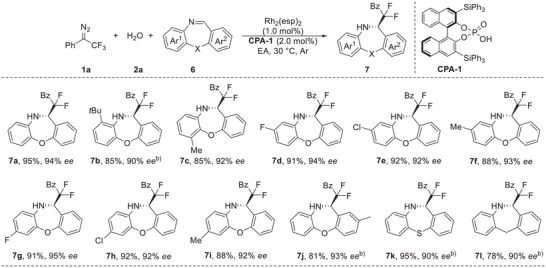

^a)^ The reaction was carried out on a 0.1 mmol scale: to the mixture of Rh_2_(esp)_2_ (0.76 mg, 1.0 mol%), **2a** (2.7 µL, 0.15 mmol) and **CPA‐1** (1.8 mg, 2.0 mol%) in EA (1.0 mL), was added a solution of diazo compound **1a** (27.9 mg, 0.15 mmol) and imine **6** (0.1 mmol) in EA (1.0 mL) via syringe pump over 2 h under an argon atmosphere at 30 °C, and the reaction mixture was stirred for an additional 1∼2 h under these conditions;

^b)^ The reaction was conducted with 10 mol% of **CPA‐1** loading.

**Figure 2:**
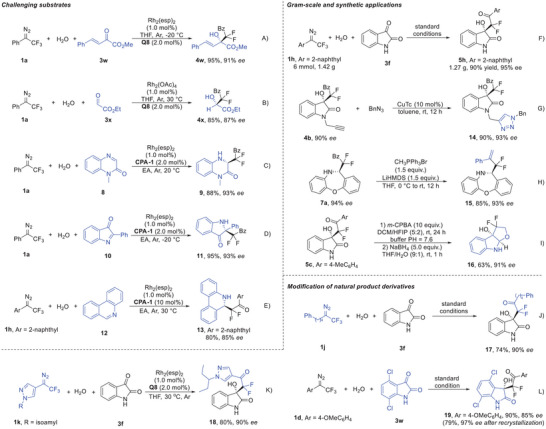
Synthetic applications and gram‐scale reaction. A) Synthesis of compound **4w**. B) Synthesis of compound **4x**. C) Synthesis of compound **9**. D) Synthesis of compound **11**. E) Synthesis of compound **13**. F) Gram‐scale reaction for the synthesis of compound **5** **h**. G) Synthesis of compound **14**. H) Synthesis of compound **15**. I) Synthesis of compound **16**. J) Synthesis of compound **17**. K) Synthesis of compound **18**. L) Synthesis of compound **19**.

Subsequently, the scope of this three‐component reaction with respect to other types of cyclic imine derivatives was examined (**Figure** **2**). Under optimized conditions, the Aldol‐type addition with linear ketones, methyl (*E*)−2‐oxo‐4‐phenylbut‐3‐enoate (**3w**) and ethyl 2‐oxoacetate (**3x**), proceeded smoothly, giving the products **4w** and **4x** in 95% and 85% yields with 91% and 87% *ee*, respectively (Figure 2A,B). The Mannich‐type adduct **9** was obtained in 88% yield with 93% *ee* under current conditions (Figure 2C), which was formed in 84% *ee* in previous work using difluoroenoxysilane as the nucleophile.^[^ ^15b^ ^]^ The 2‐phenyl‐3*H*‐indol‐3‐one **10** also worked very well under the optimum catalytic conditions at −20 °C, giving the difluorocarbonylation product **11** with comparable high reactivity and excellent enantioselectivity (Figure 2D).^[^ ^15b,d^ ^]^ Notably, the generally inert phenanthridine **12** delivered the corresponding product **13** smoothly in high yield and enantioselectivity under slightly modified conditions (Figure 2E).^[^ ^24^ ^]^

To demonstrate the synthetic utility of this strategy, the reaction was scaled up to a gram scale, and the corresponding product **5** **h** was obtained in 90% yield with 95% *ee* (Figure 2F). Then, the synthetic transformations of these generated products were performed. The product **4b**, bearing a terminal alkyne unit, was subjected to catalytic [3+2]‐cycloaddition with benzyl azide in the presence of copper(I) thiophene‐2‐carboxylate hydrate (CuTC), yielding the triazole **14** in 90% yields with 93% *ee* after separation by crystallization in DCM (Figure 2G). The Wittig reaction of **7a** proceeded smoothly, affording the terminal alkene product **15** in 85% yield without decreasing the *ee* (Figure 2H, 93% *ee*). The difluorocarbonylation adduct **5c** was converted to the tricyclic structure **16** through a sequential Baeyer‐Villiger oxidation and reductive cyclization procedure, which could potentially be used in the synthesis of the difluorinated analogues of (+)‐madindoline (Figure 2I).^[^ ^25^ ^]^ Moreover, this robust method might also be used for the modification of bioactive molecules and natural products. For example, 1,1,1‐trifluoro‐6‐phenylhexan‐2‐one, which is an inhibitor of GIVA cPLA_2_ and GVIA iPLA_2_,^[^ ^26^ ^]^ was used as the starting material for the synthesis of trifluoromethyl diazo compound **1j**, and then converted to the isatin hybrid product **17** through this three‐component reaction in 74% yield with 90% *ee* (Figure 2J). Difluoromethyl 4‐pyrazolyl ketone motif, which is the key unit of an anti‐malarial molecule,^[^ ^27^ ^]^ was decorated with isatin through an asymmetric aldol‐type addition using diazo compound **1k** as difluorination reagent, leading to adduct **18** in 80% yield with 90% *ee* after separation by crystallization in DCM (Figure 2K). In addition, the current protocol was also found to be feasible for the synthesis of the difluoro analogues of YK‐4‐279, which could potentially be used for the treatment of high‐risk and relapsing neuroblastoma,^[^ ^28^ ^]^ affording the difluorinated adduct **19** in 90% yield with 85% *ee* (Figure 2L, and after recrystallization, the *ee* was enhanced to 97% with 79% yield).

**Figure 3:**
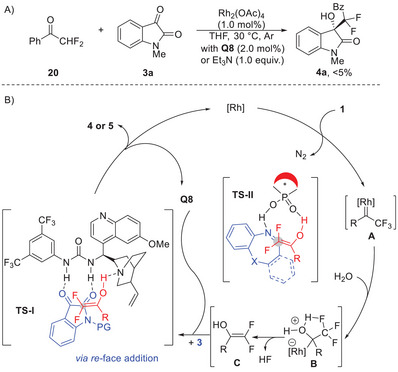
Control experiment and proposed reaction mechanism. A) Control experiment of ketone **20** with **3a** under optimal conditions. B) Proposed reaction mechanism.

To gain insight into the mechanism of this reaction, control experiments with α‐CF_2_H ketone **20** and isatin **3a** were conducted under the optimal conditions or in the presence of base Et_3_N instead of **Q8**, and no reaction occurred (**Figure** **3**A; see Figures S2 and S3, Supporting Information for details). This result suggests that a stepwise pathway is not the case in this reaction for the formation of difluorinated adduct. Furthermore, only decomposition of diazo compound **1a** was observed when the reaction was conducted in absent of water under the otherwise identical conditions, and the most of isatin **3a** was retained (see Figure S4, Supporting Information for details). Based on these results and previous studies,^[^ ^18^, ^19^, ^20^, ^21^ ^]^ a plausible reaction pathway is proposed in Figure 3B. Initially, the carbene intermediate **A** is formed followed by the generation of ylide intermediate **B** by addition with water **2a**. Subsequent C─F bond dissociation via elimination of a molecule of HF gives an enol intermediate **C**.^[^ ^18^ ^]^ A 1,3‐*H* shift process is proposed to occur directly to produce the α‐CF_2_H ketone **20** through this intermediate.^[^ ^18a^ ^]^ The electrophilic reagent isatin **3** intercepts this species with the assistance of a bifunctional urea catalyst **Q8**, delivering the aldol addition products **4** or **5** ^[^ ^21^ ^]^ and regenerates the organocatalyst simultaneously. The elegant enantioselectivity is achieved in this transformation through a dual *H*‐bonding catalysis model^[^ ^23^, ^29^ ^]^ with chiral organocatalyst through a *re*‐face addition via **TS‐I** according to the observed absolute stereochemistry of products **4a**. The asymmetric difluoroalkylation reaction with imine is realized through an analogous catalytic process via **TS‐II** with the assistance of **CPA‐1**,^[^ ^15^, ^30^ ^]^ giving the Mannich addition product **7** with excellent enantiocontrol.

## 3 Conclusion

In summary, we are reporting an asymmetric difluoroalkylation method using a robust α,α‐difluoroenol species, which is generated in situ from trifluoromethyl diazo compounds and water in the presence of a dirhodium complex. The reaction proceeds under mild conditions with low chiral organocatalyst loading and broad substrate scope. Dramatically improved enantioselectivity has been realized as demonstrated in catalytic asymmetric aldol reaction and Mannich reactions with ketones or imines in the presence of chiral organocatalysts, quinine‐derived urea and chiral phosphoric acid CPA, respectively. In comparison to well‐documented studies on metal carbene *gem*‐difunctionalization reaction, this method features a novel 1,2‐difunctionalization process by introduction of two different functionalities on the two vicinal carbons, and the disclosed relay catalysis strategy provides an effective platform for expanding asymmetric fluorination chemistry.

## 4 Experimental Section

### General Procedure for the Asymmetric Aldol‐Type Three‐Component Reaction

To a 10‐mL oven‐dried vial containing a magnetic stirring bar, isatin **3** (0.1 mmol), H_2_O (0.15 mmol, 2.7 µL, 1.5 equiv.), Rh_2_(OAc)_4_ (0.45 mg, 1.0 mol%), and organocatalyst **Q8** (1.2 mg, 2.0 mol%) in tetrahydrofuran (THF, 1.0 mL), was added a solution of diazo compound **1** (0.15 mmol, 1.5 equiv.) in 1.0 mL THF via syringe pump over 1 h under argon atmosphere at 30 °C. After addition, the reaction mixture was stirred for additional 1–2 h under these conditions until consumption of the material (monitored by TLC). Then the reaction mixture was purified by column chromatography on silica gel without any additional treatment (Hexanes : EtOAc = 5:1 to 2:1) to give the pure products **4** and **5** in good to high yields and excellent enantioselectivity.

### General Procedure for the Asymmetric Mannich‐Type Three‐Component Reaction

To a 10‐mL oven‐dried vial containing a magnetic stirring bar, H_2_O (0.15 mmol, 2.7 µL, 1.5 equiv.), Rh_2_(esp)_2_ (0.76 mg, 1.0 mol%), and chiral phosphoric acid **CPA‐1** (1.8 mg, 2.0 mol%) in ethyl acetate (EA, 1.0 mL), was added a solution of diazo compound **1** (0.15 mmol, 1.5 equiv.) and imine **6** (0.1 mmol) in 1.0 mL EA via syringe pump over 2 h under argon atmosphere at 30 °C. After addition, the reaction mixture was stirred for additional 1–2 h under these conditions until consumption of the material (monitored by TLC). Then the reaction mixture was purified by column chromatography on silica gel without any additional treatment (Hexanes : EtOAc = 50:1 to 20:1) to give the pure products **7** in good to high yields and excellent enantioselectivity.

## Conflict of Interest

The authors declare no conflict of interest.

## Author Contributions

XD.X. designed the reaction, optimized the conditions, performed data curation, investigation, and formal analysis. S.D., K.H., and J.H. performed data curation, investigation, formal analysis. S.D. performed X‐ray data curation. XF.X. conceived the platform, gathered funding, led the project, and wrote the original draft.

## Supporting information

Supporting Information

Supporting Information

Supporting Information

## Data Availability

The data that support the findings of this study are available in the supplementary material of this article.

## References

[1] a) E. P. Gillis , K. J. Eastman , M. D. Hill , D. J. Donnelly , N. A. Meanwell , J. Med. Chem. 2015, 58, 8315; 26200936 10.1021/acs.jmedchem.5b00258

[2] a) J.‐A. Ma , D. Cahard , Chem. Rev. 2004, 104, 6119; 15584697 10.1021/cr030143e

[3] a) T. Furuya , A. S. Kamlet , T. Ritter , Nature 2011, 473, 470; 21614074 10.1038/nature10108 PMC3119199

[4] a) L. Thomas , M. W. Andersen , K. D. Frederiksen , S. Troels , Angew. Chem., Int. Ed. 2016, 55, 10396; 10.1002/anie.201604152 27346239

[5] a) J.‐S. Yu , Y.‐L. Liu , J. Tang , X. Wang , J. Zhou , Angew. Chem., Int. Ed. 2014, 53, 9512; 10.1002/anie.201404432 25044065

[6] a) R. Jia , X. Wang , J. Hu , Tetrahedron Lett. 2021, 75, 153182;

[7] a) Z. Feng , Q.‐Q. Min , Y.‐L Xiao , B. Zhang , X. Zhang , Angew. Chem., Int. Ed. 2014, 53, 1669; 10.1002/anie.201309535 24453124

[8] a) S. Purser , P. R. Moore , S. Swallow , V. Gouverneur , Chem. Soc. Rev. 2008, 37, 320; 18197348 10.1039/b610213c

[9] a) N. A. Meanwell , J. Med. Chem. 2011, 54, 2529; 21413808 10.1021/jm1013693

[10] a) J. B. I. Sap , C. F. Meyer , N. J. W. Straathof , N. Iwumene , C. W. Am Ende , A. A. Trabanco , V. Gouverneur , Chem. Soc. Rev. 2021, 50, 8214; 34075979 10.1039/d1cs00360g

[11] a) R. R. Merchant , J. T. Edwards , T. Qin , M. M. Kruszyk , C. Bi , G. Che , D.‐H. Bao , W. Qiao , L. Sun , M. R. Collins , O. O. Fadeyi , G. M. Gallego , J. J. Mousseau , P. Nuhant , P. S. Baran , Science 2018, 360, 75; 29456201 10.1126/science.aar7335 PMC6349426

[12] a) H. Amii , K. Uneyama , Chem. Rev. 2009, 109, 2119; 19331346 10.1021/cr800388c

[13] a) H. Amii , T. Kobayashi , Y. Hatamoto , K. Uneyama , Chem. Commun. 1999, 35, 1323;

[14] a) Y. Gong , J.‐S. Yu , Y.‐J. Hao , Y. Zhou , J. Zhou , Asian J. Org. Chem. 2019, 8, 610;

[15] a) M.‐Y. Rong , J.‐S. Li , Y. Zhou , F.‐G Zhang , J.‐A. Ma , Org. Lett. 2020, 22, 9010; 33147031 10.1021/acs.orglett.0c03406

[16] a) G. K. S. Prakash , J. Hu , Acc. Chem. Res. 2007, 40, 921; 17708659 10.1021/ar700149s

[17] a) P. Zhang , C. Wolf , Angew. Chem., Int. Ed. 2013, 52, 7869; 10.1002/anie.201303551 23780866

[18] a) X. Zhang , X. Zhang , Q. Song , P. Sivaguru , Z. Wang , G. Zanoni , X. Bi , Angew. Chem., Int. Ed. 2022, 61, e202116190; 10.1002/anie.202116190 34889004

[19] a) C. Pei , C. Zhang , Y. Qian , X. Xu , Org. Biomol. Chem. 2018, 16, 8677; 30387481 10.1039/c8ob02420k

[20] a) G. Dong , M. Bao , X. Xie , S. Jia , W. Hu , X. Xu , Angew. Chem., Int. Ed. 2021, 60, 1992; 10.1002/anie.202012678 33006807

[21] a) Z. Kang , W. Chang , X. Tian , X. Fu , W. Zhao , X. Xu , Y. Liang , W. Hu , J. Am. Chem. Soc. 2021, 143, 20818; 34871492 10.1021/jacs.1c09148

[22] CCDC 2298623 and 2298622 contains the supplementary crystallographic data for **4a** and **7a**. These data can be obtained free of charge from the Cambridge crystallographic data centre *via* www.ccdc.cam.ac.uk/data_request/cif.

[23] Y.‐L. Liu , J. Zhou , Chem. Commun. 2012, 48, 1919. 10.1039/c2cc17140f 22228324

[24] a) M. Gómez‐Martínez , M. Del Carmen Pérez‐Aguilar , D. G. Piekarski , C. G. Daniliuc , O. García Mancheño , Angew. Chem., Int. Ed. 2021, 60, 5102; 10.1002/anie.202013380 PMC7986925 33306858

[25] a) T. Hirose , T. Sunazuka , T. Shirahata , D. Yamamoto , Y. Harigaya , I. Kuwajima , S. Omura , Org. Lett. 2002, 4, 501; 11843576 10.1021/ol017058i

[26] a) C. Baskakis , V. Magrioti , N. Cotton , D. Stephens , V. Constantinou‐Kokotou , E. A. Dennis , G. Kokotos , J. Med. Chem. 2008, 51, 8027; 19053783 10.1021/jm800649q PMC2649009

[27] E. Camerino , D. M. Wong , F. Tong , F. Körber , A. D. Gross , R. Islam , E. Viayna , J. M. Mutunga , J. Li , M. M. Totrov , J. R. Bloomquist , P. R. Carlier , Bioorg. Med. Chem. Lett. 2015, 25, 4405. 26386602 10.1016/j.bmcl.2015.09.019 PMC4593063

[28] M. Kollareddy , A. Sherrard , J. H. Park , M. Szemes , K. Gallacher , Z. Melegh , S. Oltean , M. Michaelis , J. Cinatl , A. Kaidi , K. Malik , Cancer Lett 2017, 403, 74. 28602975 10.1016/j.canlet.2017.05.027 PMC5542135

[29] a) S. M. Banik , A. Levina , A. M. Hyde , E. N. Jacobsen , Science 2017, 358, 761; 29123063 10.1126/science.aao5894 PMC5728151

[30] a) L. Simón , J. M. Goodman , J. Org. Chem. 2011, 76, 1775; 21309597 10.1021/jo102410r

