# Supplementary material for: Catalytic Asymmetric Difluoroalkylation Using In Situ Generated Difluoroenol Species as the Privileged Synthon
Source: Adv Sci (Weinh). 2024 Feb 6;11(14):2307520. doi: 10.1002/advs.202307520 (PMC11005710; doi:10.1002/advs.202307520)
Supplement: Supplementary file 1 — Supporting Information [file ADVS-11-2307520-s001.pdf]

ADVANCED SCIENCE
Open Access

Supporting Information

for *Adv. Sci.*, DOI 10.1002/advs.202307520

Catalytic Asymmetric Difluoroalkylation Using In Situ Generated Difluoroenol Species as the Privileged Synthon

*Xiongda Xie, Shanliang Dong, Kemiao Hong, Jingjing Huang and Xinfang Xu**

Supplementary Materials

Catalytic Asymmetric Difluoroalkylation Using *in situ* Generated Difluoroenol Species as the Privileged Synthon

Xiongda Xie,[a] Shanliang Dong,[a] Kemiao Hong,[a] Jingjing Huang,[b] and Xinfang Xu[a,*]

[a]*School of Pharmaceutical Sciences, Sun Yat-sen University, Guangzhou 510006 (P. R. China)*

[b]*School of Chemistry, Sun Yat-Sen University, Guangzhou, Guangdong 510275 (P. R. China)*

Corresponding authors: E-mail: xuxinfang@mail.sysu.edu.cn

Table of Contents

1. General Information **S2**

2. Condition Optimization **S2-S4**

3. General Procedure for the Asymmetric Three-component Reaction **S5-S33**

4. Control Experiment **S33-S36**

5. General Procedure for Scale Up and Synthetic Applications **S36-S45**

6. NMR Spectra of New Compounds 4, 5, 7, 9, 11, 13-19 **S46-S129**

7. HPLC Analyses Figures of Compounds 4, 5, 7, 9, 11, 13-19 **S130-S185**

8. Single-Crystal X-ray Diffraction of 4a and 7a **S186-S187**

9. References **S188**

1. General Information

All reactions were performed in oven-dried glassware under atmosphere of argon. Solvents were dried and distilled followed the standard methods before using. Chiral phosphoric acids (CPAs) and isatin **3** purchased from chemical vendors and used directly without any treatment. Analytical thin-layer chromatography was performed using glass plates pre-coated with 200-300 mesh silica gel impregnated with a fluorescent indicator (254 nm). Flash column chromatography was performed using silica gel (300-400 mesh). ^{1}H NMR and ^{13}C NMR spectra were recorded in $CDCl_3$ or DMSO-d_6 on 400/500 MHz spectrometer; chemical shifts are reported in ppm with the solvent signals as reference, and coupling constants (J) are given in Hertz. The peak information is described as: br = broad, s = singlet, d = doublet, t = triplet, q = quartet, m = multiplet, comp = composite. The enantioselectivity was determined on HPLC using Chiralpak IC, IA, OD-H, AD-H, IA-3, ID-3, and IF-3 column. High-resolution mass spectra (HRMS) were recorded on a commercial apparatus (ESI Source) and (CI Source). Starting materials **1**,[1] **6**,[2] and organocatalyst **Q**[3] were prepared according to the reported reference.

2. Condition Optimization

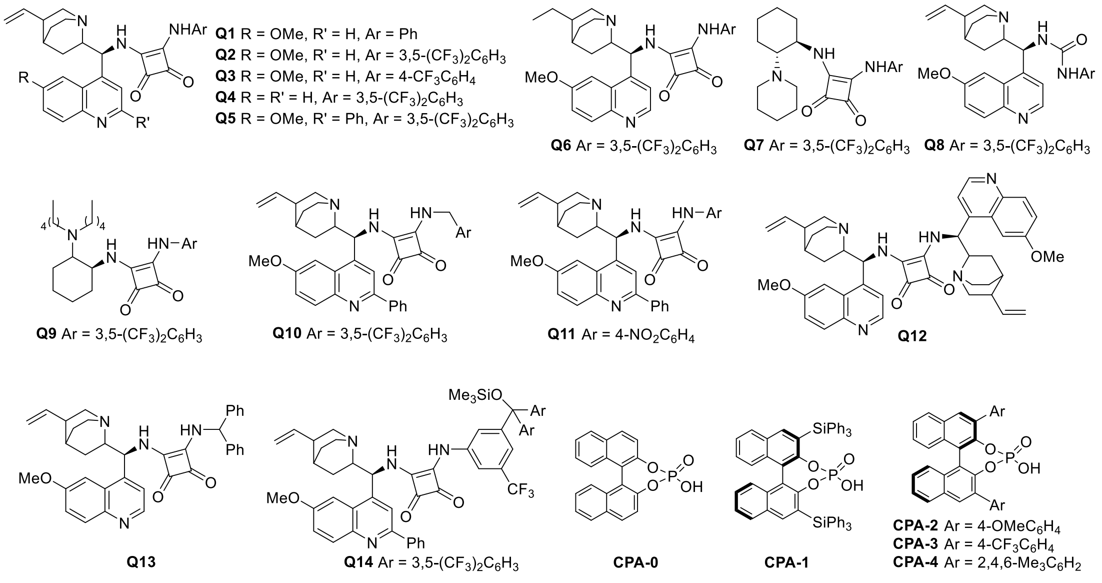

Figure S1. Chiral organocatalysts used in this work.

Table S1: Screening of solvent.[a]

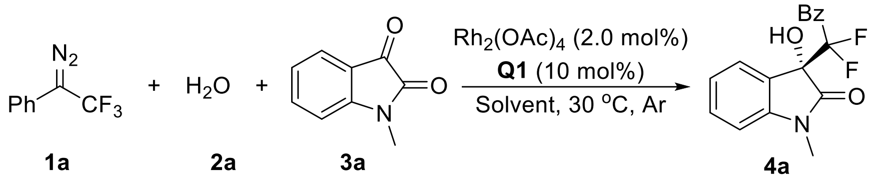

Entry	Solvent	Yield %[b] **4a**	*ee* %[c]
1	DCM	42	54
2	THF	68	65
3	EA	85	60
4	PhCl	<5	-
5	TBME	11	55

[a] The reaction was carried out on a 0.1 mmol scale: to the mixture of $Rh_2(OAc)_4$ (2.0 mol%), **2a** (0.1 mmol), **3a** (0.1 mmol), and **Q1** (10 mol%) in the indicated solvent (1.0 mL), was added a solution of diazo compound **1a** (0.1 mmol) in the same solvent (1.0 mL) *via* syringe pump over 1 h under an argon atmosphere at 30 °C, and the reaction mixture was stirred for an additional 1 h under these conditions. [b] Isolated yields. [c] Determined by chiral HPLC analysis. THF = Tetrahydrofuran, EA = Ethyl acetate, TBME = Methyl *tert*-butyl ether.

Table S2: Screening of organocatalysts.[a]

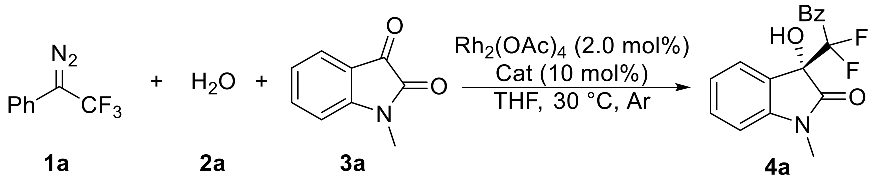

Entry	Cat	Yield %[b] **4a**	*ee* %[c]
1	**Q1**	42	54
2	**Q2**	71	85
3	**Q3**	73	71
4	**Q4**	65	82
5	**Q5**	75	83
6	**Q6**	66	82
7	**Q7**	67	60
8	**Q8**	72	93
9	**Q9**	72	55
10	**Q10**	71	85
11	**Q11**	68	77
12	**Q12**	63	47
13	**Q13**	66	29
14	**Q14**	74	85

[a] The reaction was carried out on a 0.1 mmol scale: to the mixture of $Rh_2(OAc)_4$ (2.0 mol%), **2a** (0.1 mmol), **3a** (0.1 mmol), and organocatalyst (10 mol%) in THF (1.0 mL), was added a solution of diazo compound **1a** (0.1 mmol) in the same solvent (1.0 mL) *via* syringe pump over 1 h under an argon atmosphere at 30 °C, and the reaction mixture was stirred for an additional 1 h under these conditions. [b] Isolated yields. [c] Determined by chiral HPLC analysis.

Table S3: Optimization of catalyst loading and metal catalysts.[a]

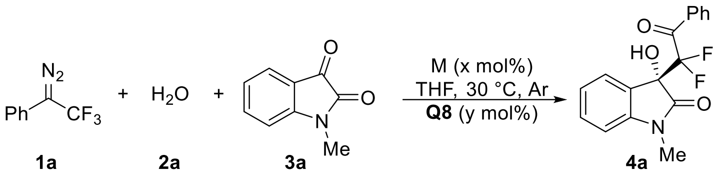

Entry	M (x mol%)	**Q8** (y mol%)	Yield %[b]	*ee* %[c]
1	$Rh_2(OAc)_4$(2.0 mol%)	(10 mol%)	72	93
2[d]	$Rh_2(OAc)_4$ (2.0 mol%)	(10 mol%)	95	93
3[d]	$Rh_2(OAc)_4$ (2.0 mol%)	(5.0 mol%)	95	93
4[d]	$Rh_2(OAc)_4$ (1.0 mol%)	(2.0 mol%)	95	93
5[d]	$Cu(MeCN)_4PF_6$ (5.0 mol%)	(2.0 mol%)	<5	-
6[d]	FeTPPCl (5.0 mol%)	(2.0 mol%)	<5	-
7[d]	CoTPP (5.0 mol%)	(2.0 mol%)	<5	-

[a] The reaction was carried out on a 0.1 mmol scale: to the mixture of M (x mol%), **2a** (0.1 mmol), **3** (0.1 mmol), and organocatalyst **Q8** (y mol%) in THF (1.0 mL), was added a solution of diazo compound **1a** (0.1 mmol) in the same solvent (1.0 mL) *via* syringe pump over 1 h under an argon atmosphere at 30 °C, and the reaction mixture was stirred for an additional 1 h under these conditions. [b] Isolated yields. [c] Determined by chiral HPLC analysis. [d] 0.15 mmol **1a** (1.5 equiv) and 0.15 mmol **2a** (1.5 equiv) were used.

Table S4: Condition optimization for the synthesis of **7a**.[a]

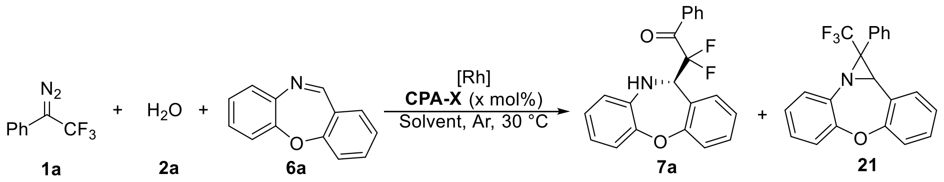

Entry	Solvent	[Rh]	**CPA-X**	Yield %[b] (**7a**/**21**)	*ee* %[c] (**7a**)
1	EA	$Rh_2(OAc)_4$	**CPA-0** (10 mol%)	-/58	-
2	EA	$Rh_2(esp)_2$	**CPA-0** (10 mol%)	95/-	-
3	EA	$Rh_2(esp)_2$	**CPA-1** (10 mol%)	95/-	95
4	EA	$Rh_2(esp)_2$	**CPA-1** (2.0 mol%)	95/-	94
5	EA	$Rh_2(esp)_2$	**CPA-2** (2.0 mol%)	95/-	0
6	EA	$Rh_2(esp)_2$	**CPA-3** (2.0 mol%)	57/-	10
7	EA	$Rh_2(esp)_2$	**CPA-4** (2.0 mol%)	95/-	39
8	THF	$Rh_2(esp)_2$	**CPA-1** (2.0 mol%)	93/-	92
9	DCE	$Rh_2(esp)_2$	**CPA-1** (2.0 mol%)	88/-	31
10	PhCl	$Rh_2(esp)_2$	**CPA-1** (2.0 mol%)	85/-	19
11	MTBE	$Rh_2(esp)_2$	**CPA-1** (2.0 mol%)	95/-	91

[a] The reaction was carried out on a 0.1 mmol scale: to the mixture of [Rh] (1.0 mol%), **2a** (0.15 mmol, 1.5 equiv) and organocatalyst (x mol%) in the indicated solvent (1.0 mL), was added a solution of diazo compound **1a** (0.15 mmol, 1.5 equiv) and **6a** (0.1 mmol) in the same solvent (1.0 mL) *via* syringe pump over 2 h under an argon atmosphere at 30 °C, and the reaction mixture was stirred for an additional 1 h under these conditions. [b] Isolated yields. [c] Determined by chiral HPLC analysis.

3. General Procedure for the Asymmetric Three-component Reaction

To a 10-mL oven-dried vial containing a magnetic stirring bar, isatin **3** (0.1 mmol), H_2O (0.15 mmol, 2.7 µL, 1.5 equiv.), $Rh_2(OAc)_4$ (0.45 mg, 1.0 mol%), and organocatalyst **Q8** (1.2 mg, 2.0 mol%) in tetrahydrofuran (THF, 1.0 mL), was added a solution of diazo compound **1** (0.15 mmol, 1.5 equiv.) in 1.0 mL THF *via* syringe pump over 1 h under argon atmosphere at 30 °C. After addition, the reaction mixture was stirred for additional 1~2 h under these conditions until consumption of the material (monitored by TLC). Then the reaction mixture was purified by column chromatography on silica gel without any additional treatment (Hexanes : EtOAc = 5:1 to 2:1) to give the pure products **4** and **5** in good to high yields and excellent enantioselectivity.

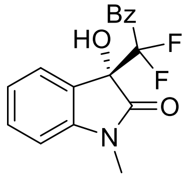

(*S*)-3-(1,1-Difluoro-2-oxo-2-phenylethyl)-3-hydroxy-1-methylindolin-2-one (4a). White solid. mp = 150 - 151 °C, 30.1 mg, 95% yield. 93% *ee*. $[\alpha]_D^{20}$ = -172.1 (c = 0.033, MeOH); ^{1}H NMR (400 MHz, $CDCl_3$) (δ, ppm) 8.05 – 7.97 (m, 2H), 7.64 – 7.58 (m, 1H), 7.52 (d, *J* = 7.4 Hz, 1H), 7.49 – 7.43 (m, 2H), 7.42 – 7.36 (m, 1H), 7.11 – 7.02 (m, 1H), 6.87 (d, *J* = 7.9 Hz, 1H), 4.35 (s, 1H), 3.22 (s, 3H); ^{13}C NMR (100 MHz, $CDCl_3$) (δ, ppm) 188.9 (t, *J* = 30.2 Hz), 172.8, 145.0, 134.8, 132.3, 131.4, 130.3, 128.8, 126.2, 124.4, 123.4, 115.7 (t, *J* = 265.3 Hz), 108.9 (d, *J* = 6.7 Hz), 76.4 (t, *J* = 24Hz), 26.6; ^{19}F NMR (376 MHz, $CDCl_3$) (δ, ppm) -108.5 (d, *J* = 299.7 Hz, 1F), -109.5 (d, *J* = 299.7 Hz, 1F); HRMS (TOF MS ESI$^+$) calculated for $C_{17}H_{14}F_2NO_3$ $[M+H]^+$: 318.0942, found 318.0940; HPLC conditions for determination of enantiomeric excess: Chiralpak IC, λ = 254 nm, hexane : 2-propanol = 80:20, flow rate = 1.0 mL/min, t_{major} = 16.8 min, t_{minor} = 14.2 min.

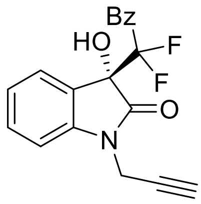

(*S*)-3-(1,1-Difluoro-2-oxo-2-phenylethyl)-3-hydroxy-1-(prop-2-yn-1-yl)indolin-2-one (4b). White solid. mp = 132 - 133 °C, 30.0 mg, 88% yield. 90% *ee*. $[\alpha]_D^{20}$ = -194.3 (c = 0.033, MeOH); ^{1}H NMR (400 MHz, $CDCl_3$) (δ, ppm) 8.06 – 7.97 (m, 2H), 7.66 – 7.59 (m, 1H), 7.54 (d, 1H), 7.50 – 7.39 (m, 3H), 7.17 – 7.05 (m, 2H), 4.51 (d, 2H), 4.32 (s, 1H), 2.28 (t, *J* = 2.5 Hz, 1H); ^{13}C NMR (100 MHz, $CDCl_3$) (δ, ppm) 188.8 (t, *J* = 30.2 Hz), 171.8, 143.1, 134.9, 132.1 (t, *J* = 2.8 Hz), 131.4, 130.4, 128.8, 126.4, 124.2, 123.7, 115.5 (t, *J* = 265.7 Hz), 110.0, 76.6 (t, *J* = 24.9 Hz), 76.2 (d, *J* = 2.5 Hz), 73.0 (d, *J* = 12.5 Hz), 29.8 (t, *J* = 4.5 Hz); ^{19}F NMR (376 MHz, $CDCl_3$) (δ, ppm) -108.3 (d, *J* = 301.7 Hz, 1F), -109.4 (d, *J* = 301.7 Hz, 1F); HRMS (TOF MS ESI$^+$) calculated for $C_{19}H_{14}F_2NO_3$ $[M+H]^+$: 342.0942, found 342.0944; HPLC conditions for determination of enantiomeric excess: Chiralpak IC, λ = 254 nm, hexane : 2-propanol = 80:20, flow rate = 1.0 mL/min, t_{minor} = 11.0 min, t_{major} = 13.3 min.

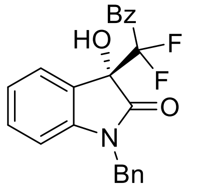

(*S*)-1-Benzyl-3-(1,1-difluoro-2-oxo-2-phenylethyl)-3-hydroxyindolin-2-one (4c). White solid. mp = 128 - 130 °C, 35.4 mg, 90% yield. 90% *ee*. $[\alpha]_D^{20}$ = -224.5 (c = 0.033, MeOH); ^{1}H NMR (400 MHz, $CDCl_3$) (δ, ppm) 8.08 – 8.01 (m, 2H), 7.62 (t, *J* = 7.4 Hz, 1H), 7.52 (d, *J* = 7.4 Hz, 1H), 7.49 – 7.43 (m, 2H), 7.37 – 7.29 (comp, 4H), 7.28 – 7.22 (m, 2H), 7.03 (t, *J* = 7.4 Hz, 1H), 6.71 (d, *J* = 7.9 Hz, 1H), 5.01 (d, *J* = 15.8 Hz, 1H), 4.80 (d, *J* = 15.8 Hz, 1H), 4.39 (s, 1H); ^{13}C NMR (100 MHz, $CDCl_3$) (δ, ppm) 189.0 (t, *J* = 29.6 Hz), 173.0, 144.2, 135.2, 134.9, 132.3 (t, *J* = 2.7 Hz), 131.3, 130.4, 129.0, 128.8, 127.9, 127.4, 126.3, 124.4, 123.4, 115.6 (t, *J* = 265.2 Hz), 110.0, 76.8 (t, *J* = 24.4 Hz), 44.3; ^{19}F NMR (376 MHz, $CDCl_3$) (δ, ppm) -108.2 (d, *J* =

301.1 Hz, 1F), -109.2 (d, J = 301.1 Hz, 1F); HRMS (TOF MS ESI$^+$) calculated for $C_{23}H_{18}F_2NO_3$ [M+H]$^+$: 394.1255, found 394.1252; HPLC conditions for determination of enantiomeric excess: Chiralpak IC, λ = 254 nm, hexane : 2-propanol = 80:20, flow rate = 1.0 mL/min, t_{minor} = 9.4 min, t_{major} = 13.3 min.

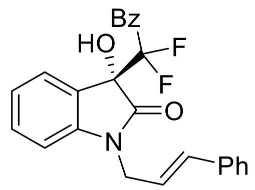

(*S*)-1-Cinnamyl-3-(1,1-difluoro-2-oxo-2-phenylethyl)-3-hydroxyindolin-2-one (4d). White solid. mp = 145 - 146 °C, 37.3 mg, 89% yield. 90% *ee*. $[\alpha]_D^{20}$ = -176.3 (c = 0.033, MeOH); ^{1}H NMR (400 MHz, DMSO-d_6) (δ, ppm) 8.11 – 7.99 (m, 2H), 7.73 (m, 1H), 7.63 – 7.55 (m, 2H), 7.52 (s, 1H), 7.44 – 7.36 (comp, 4H), 7.33 (t, J = 7.5 Hz, 2H), 7.24 (t, J = 7.2 Hz, 1H), 7.11 (d, J = 7.8 Hz, 1H), 7.09 – 7.04 (m, 1H), 6.68 (d, J = 16.1 Hz, 1H), 6.34 – 6.19 (m, 1H), 4.57 – 4.37 (m, 2H); ^{13}C NMR (100 MHz, DMSO-d_6) (δ, ppm) 187.8 (t, J = 28.5 Hz), 171.5, 143.4, 136.0, 134.7, 132.5, 131.8, 130.8, 130.2, 128.73, 128.70, 127.8, 126.2, 125.6, 125.5, 122.9, 122.5, 116.9 (t, J = 262.5 Hz), 109.6, 75.6 (t, J = 25.2 Hz), 41.2; ^{19}F NMR (376 MHz, DMSO-d_6) (δ, ppm) -108.5 (d, J = 274.0 Hz, 1F), -109.3 (d, J = 274.0 Hz, 1F); HRMS (TOF MS ESI$^+$) calculated for $C_{25}H_{20}F_2NO_3$ [M+H]$^+$: 420.1411, found 420.1409; HPLC conditions for determination of enantiomeric excess: Chiralpak IC, λ = 254 nm, hexane : 2-propanol = 80:20, flow rate = 1.0 mL/min, t_{minor} = 13.6 min, t_{major} = 19.3 min.

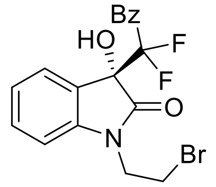

(*S*)-1-(2-Bromoethyl)-3-(1,1-difluoro-2-oxo-2-phenylethyl)-3-hydroxyindolin-2-one (4e). White solid. mp = 131 - 132 °C, 36.1 mg, 88% yield. 90% *ee*. $[\alpha]_D^{20}$ = -184.6 (c = 0.033, MeOH); ^{1}H NMR (400 MHz, $CDCl_3$) (δ, ppm) 8.02 (d, J = 7.8 Hz,

2H), 7.66 – 7.59 (m, 1H), 7.53 (d, *J* = 7.4 Hz, 1H), 7.49 – 7.43 (m, 2H), 7.43 – 7.37 (m, 1H), 7.16 – 7.03 (m, 1H), 6.96 (d, *J* = 7.9 Hz, 1H), 4.30 (s, 1H), 4.27 – 4.18 (m, 1H), 4.09 – 3.94 (m, 1H), 3.58 (t, *J* = 7.3 Hz, 2H); ^{13}C NMR (100 MHz, $CDCl_3$) (δ, ppm) 188.6 (t, *J* = 31.1 Hz), 172.8, 143.6, 135.1, 131.9 (t, *J* = 2.5 Hz), 131.4, 130.4 (t, *J* = 2.5 Hz), 128.8, 126.6, 124.2 (d, *J* = 2.1 Hz), 123.6, 115.7 (t, *J* = 265.9 Hz), 109.0, 77.2 (t, *J* = 23.2 Hz), 42.1, 26.6; ^{19}F NMR (376 MHz, $CDCl_3$) (δ, ppm) -108.2 (d, *J* = 302.2 Hz, 1F), -109.4 (d, *J* = 302.2 Hz, 1F); HRMS (TOF MS ESI$^+$) calculated for $C_{18}H_{15}BrF_2NO_3$ [M+H]$^+$: 410.0203, found 410.0201; HPLC conditions for determination of enantiomeric excess: Chiralpak IA, λ = 254 nm, hexane : 2-propanol = 80:20, flow rate = 1.0 mL/min, t_{major} = 10.4 min, t_{minor} = 23.8 min.

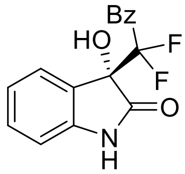

(*S*)-3-(1,1-Difluoro-2-oxo-2-phenylethyl)-3-hydroxyindolin-2-one (4f). White solid. mp = 154 - 155 °C, 28.8 mg, 95% yield. 95% *ee*. $[\alpha]_D^{20}$ = -164.5 (c = 0.033, MeOH); ^{1}H NMR (400 MHz, DMSO-d_6) (δ, ppm) 10.62 (s, 1H), 8.08 (d, *J* = 7.5 Hz, 2H), 7.87 – 7.68 (m, 1H), 7.64 – 7.45 (m, 2H), 7.43 – 7.21 (m, 3H), 7.03 – 6.94 (m, 1H), 6.88 (d, *J* = 7.4 Hz, 1H); ^{13}C NMR (100 MHz, DMSO-d_6) (δ, ppm) 187.8 (t, *J* = 28.2 Hz), 173.3, 143.0, 134.6, 132.7, 130.7, 130.3, 128.7, 126.3, 125.8, 121.8, 116.9 (t, *J* = 261.7 Hz), 110.1, 75.8 (t, *J* = 25.5 Hz); ^{19}F NMR (376 MHz, DMSO-d_6) (δ, ppm) -108.7 (d, *J* = 272.1 Hz, 1F), -109.6 (d, *J* = 272.1 Hz, 1F); HRMS (TOF MS ESI$^+$) calculated for $C_{16}H_{12}F_2NO_3$ [M+H]$^+$: 304.0785, found 304.0782; HPLC conditions for determination of enantiomeric excess: Chiralpak IC, λ = 254 nm, hexane : 2-propanol = 80:20, flow rate = 1.0 mL/min, t_{minor} = 9.7 min, t_{major} = 12.9 min.

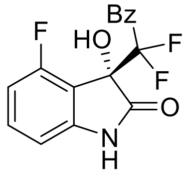

(*S*)-3-(1,1-Difluoro-2-oxo-2-phenylethyl)-4-fluoro-3-hydroxyindolin-2-one (4g). White solid. mp = 156 - 157 °C, 28.9 mg, 90% yield. 96% *ee*. $[\alpha]_D^{20}$ = -184.5 (c = 0.033, MeOH); ^{1}H NMR (400 MHz, DMSO-d_6) (δ, ppm) 10.88 (s, 1H), 8.14 (d, *J* = 7.4 Hz, 2H), 7.81 – 7.70 (m, 1H), 7.67 – 7.55 (m, 3H), 7.46 – 7.27 (m, 1H), 6.84 – 6.76 (m, 1H), 6.73 (d, *J* = 7.7 Hz, 1H); ^{13}C NMR (100 MHz, DMSO-d_6) (δ, ppm) 187.6 (t, *J* = 27.5 Hz), 172.9, 159.1 (d, *J* = 251.3 Hz), 145.0, 134.5, 133.2 (d, *J* = 8.2 Hz), 132.8, 130.5, 128.5, 116.6 (t, *J* = 261.4 Hz), 111.8 (d, *J* = 19.3 Hz), 109.7 (d, *J* = 22.0 Hz), 106.6, 77.0 (t, *J* = 27.9 Hz); ^{19}F NMR (376 MHz, DMSO-d_6) (δ, ppm) -107.6 (dd, *J* = 261.9, 18.5 Hz, 1F), -108.8 (dd, *J* = 261.9, 13.6 Hz, 1F), -112.9 (t, *J* = 17.7 Hz, 1F); HRMS (TOF MS ESI$^+$) calculated for $C_{16}H_{11}F_3NO_3$ $[M+H]^+$: 322.0691, found 322.0689; HPLC conditions for determination of enantiomeric excess: Chiralpak IA, λ = 254 nm, hexane : 2-propanol = 80:20, flow rate = 1.0 mL/min, t_{major} = 9.0 min, t_{minor} = 14.2 min.

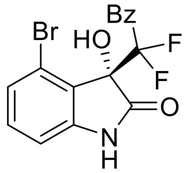

(*S*)-4-Bromo-3-(1,1-difluoro-2-oxo-2-phenylethyl)-3-hydroxyindolin-2-one (4h). White solid. mp = 153 - 154 °C, 34.8 mg, 91% yield. 95% *ee*. $[\alpha]_D^{20}$ = -174.3 (c = 0.033, MeOH); ^{1}H NMR (400 MHz, DMSO-d_6) (δ, ppm) 10.82 (s, 1H), 8.20 (d, *J* = 7.5 Hz, 2H), 7.77 – 7.67 (m, 1H), 7.62 – 7.54 (m, 2H), 7.45 (s, 1H), 7.30 – 7.13 (m, 2H), 6.88 (d, *J* = 7.3 Hz, 1H); ^{13}C NMR (100 MHz, DMSO-d_6) (δ, ppm) 187.5 (dd, *J* = 28.8, 25.6 Hz), 173.2 (d, *J* = 5.7 Hz), 145.3, 134.2, 133.2, 132.4, 130.7, 128.4, 126.7, 124.6, 120.1, 117.2 (dd, *J* = 268.0, 256.5 Hz), 109.6, 78.9 (dd, *J* = 31.5, 24.5 Hz); ^{19}F NMR (376 MHz, DMSO-d_6) (δ, ppm) -104.3 (d, *J* = 255.4 Hz, 1F), -107.0 (d, *J* = 255.4 Hz, 1F); HRMS (TOF MS ESI$^+$) calculated for $C_{16}H_{12}BrF_2NO_3$ $[M+H]^+$: 381.9890, found 381.9888; HPLC conditions for determination of enantiomeric excess: Chiralpak IA, λ = 254 nm, hexane : 2-propanol = 80:20, flow rate = 1.0 mL/min, t_{major} = 8.0 min, t_{minor} = 12.0 min.

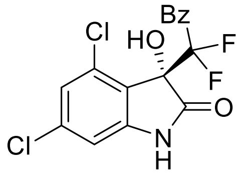

(*S*)-4,6-Dichloro-3-(1,1-difluoro-2-oxo-2-phenylethyl)-3-hydroxyindolin-2-one (4i). White solid. mp = 162 - 163 °C, 34.6 mg, 93% yield. 93% *ee*. $[\alpha]_D^{20}$ = -184.3 (c = 0.033, MeOH); ^{1}H NMR (400 MHz, DMSO-d_6) (δ, ppm) 11.03 (s, 1H), 8.16 (d, *J* = 8.0 Hz, 2H), 7.76 – 7.66 (m, 1H), 7.65 – 7.54 (m, 3H), 7.17 (d, *J* = 1.8 Hz, 1H), 6.89 (d, *J* = 1.8 Hz, 1H); ^{13}C NMR (100 MHz, DMSO-d_6) (δ, ppm) 187.4 (dd, *J* = 28.2, 26.1 Hz), 173.0 (d, *J* = 4.3 Hz), 146.0, 135.9, 134.4, 133.0, 132.6, 130.5, 128.5, 122.7 (d, *J* = 6.3 Hz), 121.8, 116.8 (dd, *J* = 266.5, 258.7 Hz), 109.3 (d, *J* = 7.9 Hz), 78.1 (dd, *J* = 30.2, 25.4 Hz); ^{19}F NMR (376 MHz, DMSO-d_6) (δ, ppm) -105.3 (d, *J* = 257.9 Hz, 1F), -107.2 (d, *J* = 257.9 Hz, 1F); HRMS (TOF MS ESI$^+$) calculated for $C_{16}H_{10}Cl_2F_2NO_3$ $[M+H]^+$: 372.0006, found 372.0004; HPLC conditions for determination of enantiomeric excess: Chiralpak IA, λ = 254 nm, hexane : 2-propanol = 80:20, flow rate = 1.0 mL/min, t_{major} = 7.9 min, t_{minor} = 13.7 min.

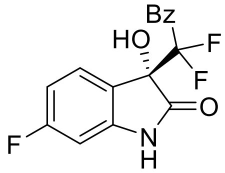

(*S*)-3-(1,1-Difluoro-2-oxo-2-phenylethyl)-6-fluoro-3-hydroxyindolin-2-one (4j). White solid. mp = 172 - 173 °C, 28.6 mg, 89% yield. 94% *ee*. $[\alpha]_D^{20}$ = -192.3 (c = 0.033, MeOH); ^{1}H NMR (500 MHz, DMSO-d_6) (δ, ppm) 10.80 (s, 1H), 8.07 (d, *J* = 7.7 Hz, 2H), 7.80 – 7.64 (m, 1H), 7.62 – 7.53 (m, 2H), 7.41 (s, 1H), 7.38 – 7.20 (m, 1H), 6.87 – 6.75 (m, 1H), 6.71 (d, *J* = 9.0 Hz, 1H); ^{13}C NMR (100 MHz, DMSO-d_6) (δ, ppm) 187.8 (t, *J* = 28.5 Hz), 173.5, 164.6, 162.6, 144.9 (d, *J* = 12.7 Hz), 134.7, 132.5, 130.3, 128.8, 127.5 (d, *J* = 10.6 Hz), 122.3, 116.8 (t, *J* = 263.1 Hz), 103.2 (dd, *J* = 1216.7, 24.9 Hz), 75.4 (t, *J* = 25.4 Hz); ^{19}F NMR (376 MHz, DMSO-d_6) (δ, ppm) -108.7 (d, *J* = 274.2 Hz, 1F), -109.1 (s, 1F), -109.5 (d, *J* = 274.2 Hz, 1F); HRMS

(TOF MS ESI+) calculated for $C_{16}H_{11}F_3NO_3$ $[M+H]^+$: 322.0691, found 322.0689; HPLC conditions for determination of enantiomeric excess: Chiralpak IA, λ = 254 nm, hexane : 2-propanol = 80:20, flow rate = 1.0 mL/min, t_{major} = 9.5 min, t_{minor} = 14.8 min.

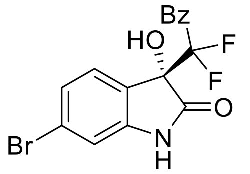

(*S*)-6-Bromo-3-(1,1-difluoro-2-oxo-2-phenylethyl)-3-hydroxyindolin-2-one (4k). White solid. mp = 182 - 183 °C, 34.0 mg, 89% yield. 93% *ee*. $[\alpha]_D^{20}$ = -202.6 (c = 0.033, MeOH); ^{1}H NMR (500 MHz, DMSO-d_6) (δ, ppm) 10.80 (s, 1H), 8.16 – 8.01 (m, 2H), 7.84 – 7.70 (m, 1H), 7.62 – 7.55 (m, 2H), 7.47 (s, 1H), 7.26 (d, *J* = 7.9 Hz, 1H), 7.19 (d, *J* = 7.9 Hz, 1H), 7.04 (s, 1H); ^{13}C NMR (100 MHz, DMSO-d_6) (δ, ppm) 187.7 (t, *J* = 28.5 Hz), 173.1, 144.6, 134.8, 132.4, 130.3, 128.8, 127.5, 125.7, 124.6, 123.5, 116.6 (t, *J* = 263.9 Hz), 113.0, 75.4 (t, *J* = 25.3 Hz); ^{19}F NMR (376 MHz, DMSO-d_6) (δ, ppm) -108.6 (d, *J* = 275.8 Hz, 1F), -109.4 (d, *J* = 275.8 Hz, 1F); HRMS (TOF MS ESI+) calculated for $C_{16}H_{11}BrF_2NO_3$ $[M+H]^+$: 381.9890, found 381.9888; HPLC conditions for determination of enantiomeric excess: Chiralpak IA, λ = 254 nm, hexane : 2-propanol = 80:20, flow rate = 1.0 mL/min, t_{major} = 10.2 min, t_{minor} = 17.8 min.

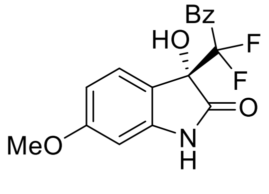

(*S*)-3-(1,1-Difluoro-2-oxo-2-phenylethyl)-3-hydroxy-6-methoxyindolin-2-one (4l). White solid. mp = 162 - 163 °C, 30.7 mg, 92% yield. 95% *ee*. $[\alpha]_D^{20}$ = -129.1 (c = 0.033, MeOH); ^{1}H NMR (400 MHz, DMSO-d_6) (δ, ppm) 10.57 (s, 1H), 8.14 – 7.95 (m, 2H), 7.89 – 7.64 (m, 1H), 7.65 – 7.50 (m, 2H), 7.40 – 7.06 (m, 2H), 6.52 (d, *J* = 8.2 Hz, 1H), 6.42 (s, 1H), 3.75 (s, 3H); ^{13}C NMR (100 MHz, DMSO-d_6) (δ, ppm)

188.0 (t, J = 28.4 Hz), 173.8, 161.4, 144.4, 134.5, 132.8, 130.3, 128.7, 126.9, 118.0, 117.0 (t, J = 261.4 Hz), 106.6, 96.8, 75.6 (t, J = 25.6 Hz), 55.3 (d, J = 7.7 Hz); ^{19}F NMR (376 MHz, DMSO-d_6) (δ, ppm) -108.7 (d, J = 271.0 Hz, 1F), -109.4 (d, J = 271.0 Hz, 1F); HRMS (TOF MS ESI$^+$) calculated for $C_{17}H_{14}F_2NO_4$ [M+H]$^+$: 334.0891, found 334.0889; HPLC conditions for determination of enantiomeric excess: Chiralpak IA, λ = 254 nm, hexane : 2-propanol = 80:20, flow rate = 1.0 mL/min, t_{major} = 14.9 min, t_{minor} = 25.9 min.

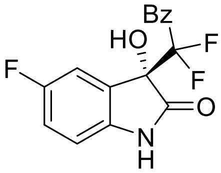

(*S*)-3-(1,1-Difluoro-2-oxo-2-phenylethyl)-5-fluoro-3-hydroxyindolin-2-one (4m). White solid. mp = 179 - 180 °C, 28.3 mg, 88% yield. 94% *ee*. $[\alpha]_D^{20}$ = -176.1 (c = 0.033, MeOH); ^{1}H NMR (400 MHz, DMSO-d_6) (δ, ppm) 10.67 (s, 1H), 8.21 – 8.01 (m, 2H), 7.82 – 7.69 (m, 1H), 7.62 – 7.55 (m, 2H), 7.51 (s, 1H), 7.22 – 7.08 (m, 2H), 6.88 (d, J = 4.9 Hz, 1H); ^{13}C NMR (100 MHz, DMSO-d_6) (δ, ppm) 187.7 (t, J = 28.5 Hz), 173.2, 158.9, 156.5, 139.2, 134.7, 132.5, 130.3, 128.7, 127.8 (d, J = 7.8 Hz), 116.7 (dd, J = 264.2, 260.0 Hz), 115.2 (dd, J = 366.4, 26.1 Hz), 111.0, 76.1 (t, J = 25.4 Hz); ^{19}F NMR (376 MHz, DMSO-d_6) (δ, ppm) -108.6 (d, J = 273.9 Hz, 1F), -109.6 (d, J = 273.9 Hz, 1F), -121.4 (s, 1F); HRMS (TOF MS ESI$^+$) calculated for $C_{16}H_{11}F_3NO_3$ [M+H]$^+$: 322.0691, found 322.0693; HPLC conditions for determination of enantiomeric excess: Chiralpak IA, λ = 254 nm, hexane : 2-propanol = 80:20, flow rate = 1.0 mL/min, t_{major} = 8.9 min, t_{minor} = 15.5 min.

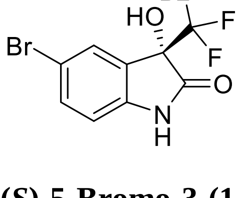

(*S*)-5-Bromo-3-(1,1-difluoro-2-oxo-2-phenylethyl)-3-hydroxyindolin-2-one (4n). White solid. mp = 189 - 190 °C, 32.5 mg, 85% yield. 92% *ee*. $[\alpha]_D^{20}$ = -229.3 (c =

0.033, MeOH); ^{1}H NMR (400 MHz, DMSO-d_6) (δ, ppm) 10.80 (s, 1H), 8.11 – 8.03 (m, 2H), 7.78 – 7.69 (m, 1H), 7.64 – 7.54 (m, 2H), 7.47 (s, 1H), 7.29 – 7.16 (m, 2H), 7.04 (s, 1H); ^{13}C NMR (100 MHz, DMSO-d_6) (δ, ppm) 187.7 (t, J = 28.6 Hz), 173.1, 144.6, 134.8, 132.4, 130.3, 128.8, 127.5, 125.7, 124.6, 123.5, 116.7 (t, J = 262.2 Hz), 113.0 (d, J = 4.7 Hz), 75.5 (t, J = 25.3 Hz); ^{19}F NMR (376 MHz, DMSO-d_6) (δ, ppm) -108.6 (d, J = 275.7 Hz, 1F), -109.4 (d, J = 275.7 Hz, 1F); HRMS (TOF MS ESI$^+$) calculated for $C_{16}H_{11}BrF_2NO_3$ [M+H]$^+$: 381.9890, found 381.9888; HPLC conditions for determination of enantiomeric excess: Chiralpak IA, λ = 254 nm, hexane : 2-propanol = 80:20, flow rate = 1.0 mL/min, t_{major} = 9.9 min, t_{minor} = 21.6 min.

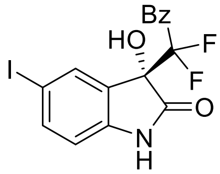

(*S*)-3-(1,1-Difluoro-2-oxo-2-phenylethyl)-3-hydroxy-5-iodoindolin-2-one (4o). White solid. mp = 257 - 258 °C, 38.6 mg, 90% yield. 93% *ee*. $[\alpha]_D^{20}$ = -129.3 (c = 0.033, MeOH); ^{1}H NMR (400 MHz, DMSO-d_6) (δ, ppm) 10.87 (s, 1H), 8.16 – 8.01 (m, 2H), 7.81 – 7.69 (m, 1H), 7.65 (d, J = 8.2 Hz, 1H), 7.62 – 7.56 (m, 3H), 7.52 (s, 1H), 6.77 (d, J = 8.1 Hz, 1H); ^{13}C NMR (100 MHz, DMSO-d_6) (δ, ppm) 187.7 (t, J = 28.2 Hz), 172.6, 142.8, 139.2, 134.8, 133.9, 132.5, 130.3, 128.9, 128.8, 116.7 (dd, J = 264.1, 260.1 Hz), 112.7 (d, J = 5.3 Hz), 84.4, 75.7 (t, J = 25.4 Hz); ^{19}F NMR (376 MHz, DMSO-d_6) (δ, ppm) -108.5 (d, J = 274.5 Hz, 1F), -109.4 (d, J = 274.5 Hz, 1F); HRMS (TOF MS ESI$^+$) calculated for $C_{16}H_{11}F_2INO_3$ [M+H]$^+$: 429.9752, found 429.9750; HPLC conditions for determination of enantiomeric excess: Chiralpak IA, λ = 254 nm, hexane : 2-propanol = 80:20, flow rate = 1.0 mL/min, t_{major} = 10.4 min, t_{minor} = 23.3 min.

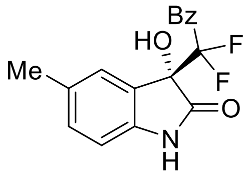

(*S*)-3-(1,1-Difluoro-2-oxo-2-phenylethyl)-3-hydroxy-5-methylindolin-2-one (4p). White solid. mp = 180 - 182 °C, 29.8 mg, 94% yield. 95% *ee*. $[\alpha]_D^{20}$ = -157.9 (c = 0.033, MeOH); ^{1}H NMR (400 MHz, DMSO-d_6) (δ, ppm) 10.51 (s, 1H), 8.14 – 8.05 (m, 2H), 7.77 – 7.69 (m, 1H), 7.63 – 7.50 (m, 2H), 7.30 (s, 1H), 7.16 (s, 1H), 7.11 (d, *J* = 7.3 Hz, 1H), 6.77 (d, *J* = 7.3 Hz, 1H), 2.23 (s, 3H); ^{13}C NMR (100 MHz, DMSO-d_6) (δ, ppm) 187.9 (t, *J* = 28.2 Hz), 173.3, 140.5, 134.5, 132.7, 130.86, 130.84, 130.7, 130.3, 128.7, 126.4, 117.0 (t, *J* = 261.6 Hz), 109.9 (d, *J* = 3.5 Hz), 76.0 (t, *J* = 25.5 Hz), 20.6 (d, *J* = 5.1 Hz); ^{19}F NMR (376 MHz, DMSO-d_6) (δ, ppm) -108.6 (d, *J* = 271.8 Hz, 1F), -109.5 (d, *J* = 271.8 Hz, 1F); HRMS (TOF MS ESI$^+$) calculated for $C_{17}H_{14}F_2NO_3$ $[M+H]^+$: 318.0942, found 318.0940; HPLC conditions for determination of enantiomeric excess: Chiralpak IA, λ = 254 nm, hexane : 2-propanol = 80:20, flow rate = 1.0 mL/min, t_{major} = 9.8 min, t_{minor} = 20.0 min.

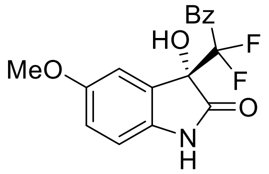

(*S*)-3-(1,1-Difluoro-2-oxo-2-phenylethyl)-3-hydroxy-5-methoxyindolin-2-one (4q). White solid. mp = 167 - 168 °C, 30.7 mg, 92% yield. 95% *ee*. $[\alpha]_D^{20}$ = -222.2 (c = 0.033, MeOH); ^{1}H NMR (400 MHz, DMSO-d_6) (δ, ppm) 10.45 (s, 1H), 8.14 – 8.05 (m, 2H), 7.78 – 7.68 (m, 1H), 7.63 – 7.53 (m, 2H), 7.37 (s, 1H), 7.00 – 6.85 (m, 2H), 6.80 (d, *J* = 8.1 Hz, 1H), 3.69 (s, 3H); ^{13}C NMR (100 MHz, DMSO-d_6) (δ, ppm) 187.8 (t, *J* = 28.3 Hz), 173.2, 154.8, 136.1, 134.6, 132.8, 130.3, 128.7, 127.4, 116.9 (dd, *J* = 263.5, 259.9 Hz), 115.3, 112.7 (d, *J* = 5.2 Hz), 110.6, 76.3 (t, *J* = 26.7 Hz), 55.6 (d, *J* = 7.2 Hz); ^{19}F NMR (376 MHz, DMSO-d_6) (δ, ppm) -108.8 (d, *J* = 271.2 Hz, 1F), -109.8 (d, *J* = 271.2 Hz, 1F); HRMS (TOF MS ESI$^+$) calculated for $C_{17}H_{14}F_2NO_4$ $[M+H]^+$: 334.0891, found 334.0893; HPLC conditions for determination

of enantiomeric excess: Chiralpak IA, λ = 254 nm, hexane : 2-propanol = 80:20, flow rate = 1.0 mL/min, t_{major} = 13.0 min, t_{minor} = 27.5 min.

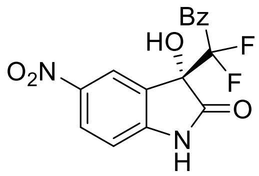

(*S*)-3-(1,1-Difluoro-2-oxo-2-phenylethyl)-3-hydroxy-5-nitroindolin-2-one (4r). White solid. mp = 170 - 172 °C, 28.6 mg, 82% yield. 95% *ee*. $[\alpha]_D^{20}$ = -197.6 (c = 0.033, MeOH); ^{1}H NMR (400 MHz, DMSO-d_6) (δ, ppm) 11.42 (s, 1H), 8.35 – 8.25 (m, 1H), 8.16 – 8.06 (m, 3H), 7.80 – 7.72 (m, 2H), 7.65 – 7.55 (m, 2H), 7.12 (d, *J* = 8.7 Hz, 1H); ^{13}C NMR (100 MHz, DMSO-d_6) (δ, ppm) 187.5 (t, *J* = 28.6 Hz), 173.4, 149.4, 142.1, 135.1, 132.0, 130.3, 128.9, 127.9, 127.2, 121.1, 116.5 (t, *J* = 263.5 Hz), 110.6, 75.2 (t, *J* = 24.8 Hz); ^{19}F NMR (376 MHz, DMSO-d_6) (δ, ppm) -108.4 (d, *J* = 281.7 Hz, 1F), -109.2 (d, *J* = 281.7 Hz, 1F); HRMS (TOF MS ESI$^+$) calculated for $C_{16}H_{11}F_2N2O_5$ $[M+H]^+$: 349.0636, found 349.0633; HPLC conditions for determination of enantiomeric excess: Chiralpak IA, λ = 254 nm, hexane : 2-propanol = 80:20, flow rate = 1.0 mL/min, t_{major} = 10.0 min, t_{minor} = 17.6 min.

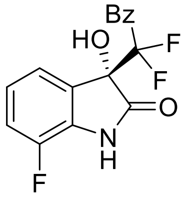

(*S*)-3-(1,1-Difluoro-2-oxo-2-phenylethyl)-7-fluoro-3-hydroxyindolin-2-one (4s). colorless oil. 27.9 mg, 87% yield. 93% *ee*. $[\alpha]_D^{20}$ = -202.6 (c = 0.033, MeOH); ^{1}H NMR (400 MHz, DMSO-d_6) (δ, ppm) 11.19 (s, 1H), 8.15 – 8.03 (m, 2H), 7.78 – 7.70 (m, 1H), 7.63 – 7.56 (m, 2H), 7.52 (s, 1H), 7.29 – 7.22 (m, 1H), 7.19 (d, *J* = 5.0 Hz, 1H), 7.02 (d, *J* = 3.5 Hz, 1H); ^{13}C NMR (100 MHz, DMSO-d_6) (δ, ppm) 187.7 (t, *J* = 28.6 Hz), 173.1, 147.6, 145.2, 134.8, 132.4, 130.3, 129.2 (d, *J* = 3.0 Hz), 128.9, 122.8 (d, *J* = 5.8 Hz), 121.8, 117.8 (d, *J* = 17.1 Hz), 116.7 (dd, *J* = 264.1, 260.9 Hz), 75.8 (t, *J* = 25.2 Hz); ^{19}F NMR (376 MHz, DMSO-d_6) (δ, ppm) -108.6 (d, *J* = 276.4 Hz, 1F), -

109.5 (d, J = 276.4 Hz, 1F), -132.7 (s, 1F); HRMS (TOF MS ESI$^+$) calculated for $C_{16}H_{11}F_3NO_3$ [M+H]$^+$: 322.0691, found 322.0686; HPLC conditions for determination of enantiomeric excess: Chiralpak IA, λ = 254 nm, hexane : 2-propanol = 80:20, flow rate = 1.0 mL/min, t_{major} = 9.5 min, t_{minor} = 14.6 min.

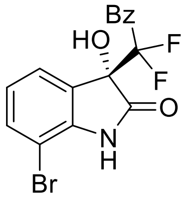

(*S*)-7-Bromo-3-(1,1-difluoro-2-oxo-2-phenylethyl)-3-hydroxyindolin-2-one (4t). colorless oil. 34.3 mg, 90% yield. 92% *ee*. $[\alpha]_D^{20}$ = -254.6 (c = 0.033, MeOH); ^1H NMR (400 MHz, $CDCl_3$) (δ, ppm) 8.21 (s, 1H), 8.10 – 7.99 (m, 2H), 7.66 – 7.60 (m, 1H), 7.49 – 7.40 (m, 4H), 6.99 – 6.91 (m, 1H), 4.78 (s, 1H); ^{13}C NMR (100 MHz, $CDCl_3$) (δ, ppm) 188.6 (t, J = 30.1 Hz), 173.5, 141.5, 135.1, 134.0, 131.8, 130.4 (t, J = 3.0 Hz), 128.9, 126.2, 125.4, 124.6, 115.5 (t, J = 266.0 Hz), 103.6, 77.9 (t, J = 23.9 Hz); ^{19}F NMR (376 MHz, $CDCl_3$) (δ, ppm) -108.2 (d, J =303.8 Hz, 1F), -109.2 (d, J = 303.8 Hz, 1F); HRMS (TOF MS ESI$^+$) calculated for $C_{16}H_{11}BrF_2NO_3$ [M+H]$^+$: 381.9890, found 381.988; HPLC conditions for determination of enantiomeric excess: Chiralpak IA, λ = 254 nm, hexane : 2-propanol = 80:20, flow rate = 1.0 mL/min, t_{major} = 11.7 min, t_{minor} = 16.4 min.

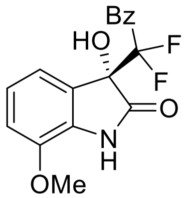

(*S*)-3-(1,1-Difluoro-2-oxo-2-phenylethyl)-3-hydroxy-7-methoxyindolin-2-one (4u). colorless oil. 28.3 mg, 85% yield. 95% *ee*. $[\alpha]_D^{20}$ = -154.6 (c = 0.033, MeOH); ^1H NMR (400 MHz, DMSO-d_6) (δ, ppm) 10.67 (s, 1H), 8.19 – 8.02 (m, 2H), 7.80 – 7.68 (m, 1H), 7.64 – 7.52 (m, 2H), 7.33 (s, 1H), 7.04 (d, J = 6.9 Hz, 1H), 7.00 – 6.92 (m, 2H), 3.83 (s, 3H); ^{13}C NMR (100 MHz, DMSO-d_6) (δ, ppm) 187.8 (t, J = 28.6 Hz), 173.3, 143.8, 134.6, 132.7, 131.7, 130.3, 128.7, 126.9, 122.5, 117.9, 116.9 (t, J =

263.5 Hz), 113.8, 76.2 (t, J = 25.5 Hz), 55.8 (d, J = 8.5 Hz); ^{19}F NMR (376 MHz, DMSO-d_6) (δ, ppm) -108.7 (d, J = 272.3 Hz, 1F), -109.6 (d, J = 272.3 Hz, 1F); HRMS (TOF MS ESI$^+$) calculated for $C_{17}H_{14}F_2NO_4$ [M+H]$^+$: 334.0891, found 334.0887; HPLC conditions for determination of enantiomeric excess: Chiralpak IA, λ = 254 nm, hexane : 2-propanol = 80:20, flow rate = 1.0 mL/min, t_{major} = 15.9 min, t_{minor} = 30.5 min.

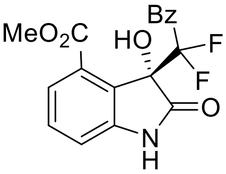

Methyl (*S*)-3-(1,1-difluoro-2-oxo-2-phenylethyl)-3-hydroxy-2-oxoindoline-4-carboxylate (4v). White solid. mp = 188 - 189 °C, 30.7 mg, 85% yield. 96% *ee*. $[\alpha]_D^{20}$ = -186.6 (c = 0.033, MeOH); ^{1}H NMR (400 MHz, DMSO-d_6) (δ, ppm) 10.96 (s, 1H), 8.13 – 8.07 (m, 2H), 7.76 – 7.67 (m, 1H), 7.63 – 7.55 (m, 2H), 7.52 – 7.44 (m, 1H), 7.39 (d, J = 7.8 Hz, 1H), 7.18 (s, 1H), 7.14 (d, J = 7.8 Hz, 1H), 3.74 (s, 3H); ^{13}C NMR (100 MHz, DMSO-d_6) (δ, ppm) 187.5 (t, J = 27.5 Hz), 172.3 (d, J = 3.9 Hz), 167.8, 144.2, 134.4, 133.2, 131.4, 130.7, 130.5, 128.6, 123.8, 123.1, 117.0 (dd, J = 264.1, 260.2 Hz), 114.2, 77.7 (dd, J = 28.7, 25.9 Hz), 52.6; ^{19}F NMR (376 MHz, DMSO-d_6) (δ, ppm) -106.9 (d, J = 259.0 Hz, 1F), -108.5 (d, J = 259.0 Hz, 1F); HRMS (TOF MS ESI$^+$) calculated for $C_{18}H_{14}F_2NO_5$ [M+H]$^+$: 362.0840, found 362.0836; HPLC conditions for determination of enantiomeric excess: Chiralpak IA, λ = 254 nm, hexane : 2-propanol = 80:20, flow rate = 1.0 mL/min, t_{major} = 11.8 min, t_{minor} = 15.6 min.

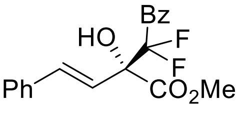

Methyl (*S*,*E*)-2-(1,1-difluoro-2-oxo-2-phenylethyl)-2-hydroxy-4-phenylbut-3-enoate (4w). Colorless oil, 32.9 mg, 95% yield. 91% *ee*; ^{1}H NMR (400 MHz, $CDCl_3$) (δ, ppm) 8.15 – 8.02 (m, 2H), 7.67 – 7.60 (m, 1H), 7.52 – 7.43 (comp, 4H), 7.39 –

7.33 (m, 2H), 7.32 – 7.27 (m, 1H), 7.06 (d, *J* = 15.8 Hz, 1H), 6.49 (d, *J* = 15.8 Hz, 1H), 4.09 (s, 1H), 3.93 (s, 3H).; ^{13}C NMR (100 MHz, $CDCl_3$) (δ, ppm) 189.4 (dd, *J* = 31.1, 28.3 Hz), 171.0 (d, *J* = 5.2 Hz), 135.8, 134.7, 134.6, 132.7 (t, *J* = 2.2 Hz), 130.5 (dd, *J* = 3.8, 2.8 Hz), 128.78, 128.76, 128.6, 120.9 (dd, *J* = 2.9, 2.0 Hz), 115.8 (dd, *J* = 267.8, 265.9 Hz), 77.7 (t, *J* = 25.1 Hz), 54.3; ^{19}F NMR (376 MHz, $CDCl_3$) (δ, ppm) -103.1 (d, *J* = 287.7 Hz), -109.7 (d, *J* = 287.8 Hz); HRMS (TOF MS ESI$^+$) calculated for $C_{19}H_{17}F_2O_4$ [M+H]$^+$: 347.1095, found 347.1092; HPLC conditions for determination of enantiomeric excess: Chiralpak OD-H, λ = 254 nm, hexane : 2-propanol = 85:15, flow rate = 1.0 mL/min, t_{major} = 7.4 min, t_{minor} = 8.7 min.

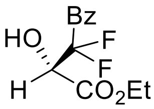

Ethyl (*S*)-3,3-difluoro-2-hydroxy-4-oxo-4-phenylbutanoate (4x). Colorless oil, 21.9 mg, 85% yield. 87% *ee*; ^{1}H NMR (400 MHz, $CDCl_3$) (δ, ppm) 8.11 – 8.05 (m, 2H), 7.70 – 7.62 (m, 1H), 7.55 – 7.48 (m, 2H), 4.76 (t, *J* = 10.7 Hz, 1H), 4.40 – 4.26 (m, 2H), 3.42 (s, 1H), 1.29 (t, *J* = 7.1 Hz, 3H); ^{13}C NMR (100 MHz, $CDCl_3$) (δ, ppm) 188.5 (t, *J* = 28.6 Hz), 168.9, 134.7, 132.4 (t, *J* = 2.1 Hz), 130.2 (t, *J* = 3.2 Hz), 128.9, 115.6 (t, *J* = 262.8 Hz), 71.1 (t, *J* = 26.7 Hz), 63.3, 14.1; ^{19}F NMR (376 MHz, $CDCl_3$) (δ, ppm) -106.9 (d, *J* = 287.6 Hz), -109.1 (d, *J* = 287.6 Hz); HRMS (TOF MS ESI$^+$) calculated for $C_{12}H_{13}F_2O_4$ [M+H]$^+$: 259.0782, found 259.0781; HPLC conditions for determination of enantiomeric excess: Chiralpak IA, λ = 254 nm, hexane : 2-propanol = 85:15, flow rate = 1.0 mL/min, t_{major} = 7.8 min, t_{minor} = 7.1 min.

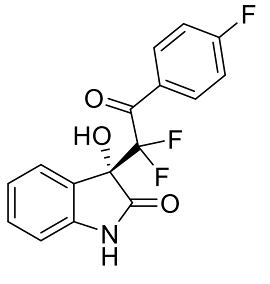

(*S*)-3-(1,1-Difluoro-2-(4-fluorophenyl)-2-oxoethyl)-3-hydroxyindolin-2-one (5a). White solid. mp = 178 - 179 °C, 27.6 mg, 86% yield. 95% *ee*. $[\alpha]_D^{20}$ = -182.2 (c = 0.033, MeOH); ^{1}H NMR (400 MHz, DMSO-d_6) (δ, ppm) 10.64 (s, 1H), 8.20 (s, 2H),

7.49 – 7.38 (m, 3H), 7.37 – 7.26 (m, 2H), 7.05 – 6.92 (m, 1H), 6.88 (d, *J* = 7.5 Hz, 1H); ^{13}C NMR (100 MHz, DMSO-d_6) (δ, ppm) 186.3 (t, *J* = 28.4 Hz), 173.3, 167.0, 164.4, 143.0, 133.7 (d, *J* = 9.0 Hz), 130.7, 129.5, 126.2, 125.8, 118.9 (d, *J* = 589.8 Hz), 116.9 (dd, *J* = 262.7, 259.3 Hz), 112.9 (d, *J* = 572.1 Hz), 76.0 (t, *J* = 25.9 Hz); ^{19}F NMR (376 MHz, DMSO-d_6) (δ, ppm) -103.1 (s, 1F), -108.8 (d, *J* = 269.2 Hz, 1F), -109.8 (d, *J* = 269.2 Hz, 1F); HRMS (TOF MS ESI$^+$) calculated for $C_{16}H_{11}F_3NO_3$ $[M+H]^+$: 322.0691, found 322.0689; HPLC conditions for determination of enantiomeric excess: Chiralpak IA, λ = 254 nm, hexane : 2-propanol = 80:20, flow rate = 1.0 mL/min, t_{major} = 9.6 min, t_{minor} = 15.7 min.

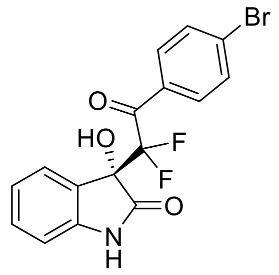

(*S*)-3-(2-(4-Bromophenyl)-1,1-difluoro-2-oxoethyl)-3-hydroxyindolin-2-one (5b). White solid. mp = 168 - 169 °C, 34.8 mg, 91% yield. 91% *ee*. $[\alpha]_D^{20}$ = -187.5 (c = 0.033, MeOH); ^1H NMR (400 MHz, DMSO-d_6) (δ, ppm) 10.64 (s, 1H), 8.06 – 7.88 (m, 2H), 7.88 – 7.75 (m, 2H), 7.41 (s, 1H), 7.38 – 7.27 (m, 2H), 7.04 – 6.97 (m, 1H), 6.87 (d, *J* = 7.5 Hz, 1H); ^{13}C NMR (100 MHz, DMSO-d_6) (δ, ppm) 187.1 (t, *J* = 28.3 Hz), 173.2, 143.0, 132.3, 131.9, 131.8, 130.8, 129.0, 126.1, 125.9, 121.9, 116.9 (dd, *J* = 263.1, 259.1 Hz), 110.1, 76.0 (dd, *J* = 27.6, 25.1 Hz); ^{19}F NMR (376 MHz, DMSO-d_6) (δ, ppm) -109.2 (d, *J* = 267.3 Hz, 1F), -110.3 (d, *J* = 267.3 Hz, 1F); HRMS (TOF MS ESI$^+$) calculated for $C_{16}H_{11}BrF_2NO_3$ $[M+H]^+$: 381.9890, found 381.9885; HPLC conditions for determination of enantiomeric excess: Chiralpak IA, λ = 254 nm, hexane : 2-propanol = 80:20, flow rate = 1.0 mL/min, t_{major} = 10.4 min, t_{minor} = 19.5 min.

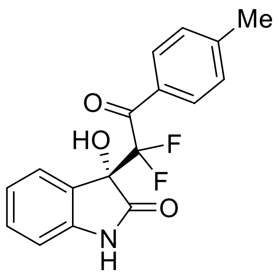

(*S*)-3-(1,1-Difluoro-2-oxo-2-(*p*-tolyl)ethyl)-3-hydroxyindolin-2-one (5c). White solid. mp = 176 - 177 °C, 29.2 mg, 92% yield. 95% *ee*. $[\alpha]_D^{20}$ = -197.2 (c = 0.033, MeOH); ^{1}H NMR (400 MHz, DMSO-d_6) (δ, ppm) 10.58 (s, 1H), 8.08 – 7.90 (m, 2H), 7.41 – 7.35 (m, 2H), 7.34 – 7.27 (m, 3H), 7.01 – 6.93 (m, 1H), 6.87 (d, *J* = 8.0 Hz, 1H), 2.40 (s, 3H); ^{13}C NMR (100 MHz, DMSO-d_6) (δ, ppm) 187.2 (t, *J* = 28.3 Hz), 173.3, 145.4, 143.0, 130.6, 130.4, 130.1, 129.2, 126.4, 125.7, 121.7, 117.0 (t, *J* = 262.0 Hz), 110.0 (d, *J* = 7.1 Hz), 75.7 (t, *J* = 25.3 Hz), 21.3 (d, *J* = 7.7 Hz); ^{19}F NMR (376 MHz, DMSO-d_6) (δ, ppm) -108.6 (d, *J* = 273.1 Hz, 1F), -109.3 (d, *J* =273.1 Hz, 1F); HRMS (TOF MS ESI$^+$) calculated for $C_{17}H_{14}F_2NO_3$ [M+H]$^+$: 318.0942, found 318.0940; HPLC conditions for determination of enantiomeric excess: Chiralpak IA, λ = 254 nm, hexane : 2-propanol = 80:20, flow rate = 1.0 mL/min, t_{major} = 11.4 min, t_{minor} = 27.6 min.

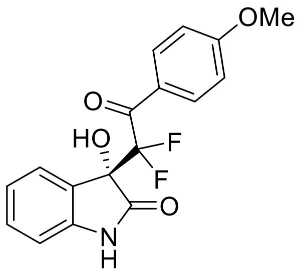

(*S*)-3-(1,1-Difluoro-2-(4-methoxyphenyl)-2-oxoethyl)-3-hydroxyindolin-2-one (5d). White solid. mp = 153 - 154 °C, 27.3 mg, 82% yield. 94% *ee*. $[\alpha]_D^{20}$ = -199.2 (c = 0.033, MeOH); ^{1}H NMR (400 MHz, DMSO-d_6) (δ, ppm) 10.57 (s, 1H), 8.14 – 8.00 (m, 2H), 7.38 – 7.21 (m, 3H), 7.17 – 7.06 (m, 2H), 7.01 – 6.93 (m, 1H), 6.86 (d, *J* = 7.7 Hz, 1H), 3.87 (s, 3H); ^{13}C NMR (100 MHz, DMSO-d_6) (δ, ppm) 185.8 (t, *J* = 28.2 Hz), 173.4, 164.3, 143.0, 133.0, 130.5, 126.5, 125.7, 125.2, 121.7, 117.2 (t, *J* = 261.9 Hz), 114.1, 110.0, 75.6 (t, *J* = 25.2 Hz), 55.7; ^{19}F NMR (376 MHz, DMSO-d_6) (δ,

ppm) -108.1 (d, J = 274.5 Hz, 1F), -108.9 (d, J = 274.5 Hz, 1F); HRMS (TOF MS ESI$^+$) calculated for $C_{17}H_{14}F_2NO_4$ [M+H]$^+$: 334.0891, found 334.0887; HPLC conditions for determination of enantiomeric excess: Chiralpak IA, λ = 254 nm, hexane : 2-propanol = 80:20, flow rate = 1.0 mL/min, t_{major} = 14.1 min, t_{minor} = 44.1 min.

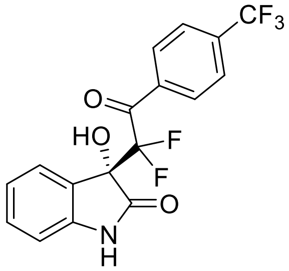

(*S*)-3-(1,1-Difluoro-2-oxo-2-(4-(trifluoromethyl)phenyl)ethyl)-3-hydroxyindolin-2-one (5e). White solid. mp = 174 - 175 °C, 34.2 mg, 92% yield. 95% *ee*. $[\alpha]_D^{20}$ = -218.2 (c = 0.033, MeOH); ^{1}H NMR (400 MHz, DMSO-d_6) (δ, ppm) 10.68 (s, 1H), 8.33 – 8.20 (m, 2H), 8.07 – 7.90 (m, 2H), 7.48 (d, J = 1.1 Hz, 1H), 7.41 – 7.28 (m, 2H), 7.09 – 6.95 (m, 1H), 6.89 (d, J = 7.7 Hz, 1H); ^{13}C NMR (100 MHz, DMSO-d_6) (δ, ppm) 187.5 (t, J = 28.2 Hz), 173.1 (d, J = 4.1 Hz), 142.9, 136.5, 133.3 (q, J = 32.1 Hz), 131.1 (d, J = 3.4 Hz), 130.9, 125.9, 125.5 (d, J = 3.7 Hz), 125.0, 122.2, 122.0, 116.7 (dd, J = 263.2, 258.0 Hz), 110.2, 76.2 (dd, J = 28.9, 24.3 Hz); ^{19}F NMR (376 MHz, DMSO-d_6) (δ, ppm) -61.9 (s, 3F), -109.7 (d, J = 265.5 Hz, 1F), -111.1 (d, J = 265.5 Hz, 1F); HRMS (TOF MS ESI$^+$) calculated for $C_{17}H_{11}F_5NO_3$ [M+H]$^+$: 372.0659, found 372.0656; HPLC conditions for determination of enantiomeric excess: Chiralpak IA, λ = 254 nm, hexane : 2-propanol = 80:20, flow rate = 1.0 mL/min, t_{major} = 7.9 min, t_{minor} = 13.2 min.

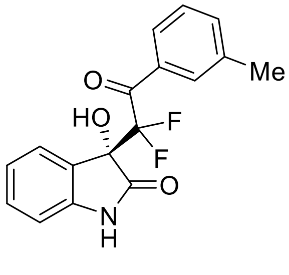

(*S*)-3-(1,1-Difluoro-2-oxo-2-(*m*-tolyl)ethyl)-3-hydroxyindolin-2-one (5f). White solid. mp = 161 - 163 °C, 26.0 mg, 82% yield. 94% *ee*. $[\alpha]_D^{20}$ = -258.2 (c = 0.033,

MeOH); ^{1}H NMR (400 MHz, DMSO-d_6) (δ, ppm) 10.62 (s, 1H), 7.90 (d, J = 7.7 Hz, 1H), 7.85 (s, 1H), 7.54 (d, J = 7.5 Hz, 1H), 7.50 – 7.43 (m, 1H), 7.37 – 7.26 (m, 3H), 7.03 – 6.93 (m, 1H), 6.87 (d, J = 7.6 Hz, 1H), 2.38 (s, 3H); ^{13}C NMR (100 MHz, DMSO-d_6) (δ, ppm) 187.9 (t, J = 28.4 Hz), 173.3, 143.0, 138.1, 135.2, 132.7, 130.7, 130.4, 128.6, 127.7, 126.3, 125.8, 121.7, 117.0 (t, J = 261.9 Hz), 110.0, 75.8 (t, J = 25.3 Hz), 20.9; ^{19}F NMR (376 MHz, DMSO-d_6) (δ, ppm) -108.6 (d, J = 272.8 Hz, 1F), -109.4 (d, J = 272.8 Hz, 1F); HRMS (TOF MS ESI$^+$) calculated for $C_{17}H_{14}F_2NO_3$ [M+H]$^+$: 318.0942, found 318.0940; HPLC conditions for determination of enantiomeric excess: Chiralpak IA, λ = 254 nm, hexane : 2-propanol = 80:20, flow rate = 1.0 mL/min, t_{major} = 9.8 min, t_{minor} = 15.1 min.

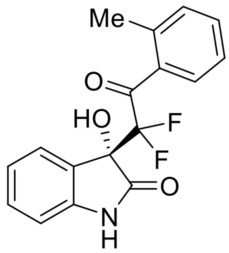

(*S*)-3-(1,1-Difluoro-2-oxo-2-(*o*-tolyl)ethyl)-3-hydroxyindolin-2-one (5g). White solid. mp = 161 - 163 °C, 27.9 mg, 88% yield. 91% *ee*. $[\alpha]_D^{20}$ = -238.2 (c = 0.033, MeOH); ^{1}H NMR (400 MHz, DMSO-d_6) (δ, ppm) 10.62 (s, 1H), 7.67 (d, J = 7.1 Hz, 1H), 7.50 – 7.42 (m, 1H), 7.35 – 7.25 (m, 4H), 7.24 (s, 1H), 6.99 – 6.92 (m, 1H), 6.86 (d, J = 7.7 Hz, 1H), 2.03 (s, 3H); ^{13}C NMR (100 MHz, DMSO-d_6) (δ, ppm) 192.5 (dd, J = 30.1, 27.3 Hz), 173.1, 143.0, 138.1, 133.5, 132.1, 131.4, 130.8, 128.6 (t, J = 4.9 Hz), 125.9, 125.8 (d, J = 2.1 Hz), 125.4, 121.7, 115.9 (dd, J = 265.8, 262.5 Hz), 110.1, 75.5 (t, J = 24.0 Hz), 19.4; ^{19}F NMR (376 MHz, DMSO-d_6) (δ, ppm) -109.9 (d, J = 268.1 Hz, 1F), -110.8 (d, J = 268.1 Hz, 1F); HRMS (TOF MS ESI$^+$) calculated for $C_{17}H_{14}F_2NO_3$ [M+H]$^+$: 318.0942, found 318.0936; HPLC conditions for determination of enantiomeric excess: Chiralpak IA, λ = 254 nm, hexane : 2-propanol = 80:20, flow rate = 1.0 mL/min, t_{major} = 8.7 min, t_{minor} = 11.2 min.

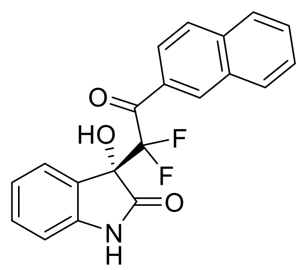

(*S*)-3-(1,1-Difluoro-2-(naphthalen-2-yl)-2-oxoethyl)-3-hydroxyindolin-2-one (5h). White solid. mp = 187 - 188 °C, 31.1 mg, 88% yield. 95% *ee*. $[\alpha]_D^{20}$ = -268.2 (c = 0.033, MeOH); ^{1}H NMR (400 MHz, DMSO-d_6) (δ, ppm) 10.65 (s, 1H), 8.82 (s, 1H), 8.18 (d, *J* = 8.1 Hz, 1H), 8.10 – 7.99 (m, 3H), 7.78 – 7.70 (m, 1H), 7.69 – 7.62 (m, 1H), 7.40 (s, 1H), 7.36 (d, *J* = 7.4 Hz, 1H), 7.33 – 7.28 (m, 1H), 7.03 – 6.95 (m, 1H), 6.88 (d, *J* = 7.7 Hz, 1H); ^{13}C NMR (100 MHz, DMSO-d_6) (δ, ppm) 187.6 (t, *J* = 28.2 Hz), 173.4, 143.0, 135.4, 133.2 (t, *J* = 4.0 Hz), 131.7, 130.7, 130.2, 130.0, 129.7, 128.3, 127.7, 127.2, 126.3, 125.9, 124.8, 121.8, 117.1 (t, *J* = 261.8 Hz), 110.1, 75.9 (t, *J* = 25.5 Hz); ^{19}F NMR (376 MHz, DMSO-d_6) (δ, ppm) -108.4 (d, *J* = 271.2 Hz, 1F), -109.2 (d, *J* = 271.2 Hz, 1F); HRMS (TOF MS ESI$^+$) calculated for $C_{20}H_{14}F_2NO_3$ $[M+H]^+$: 354.0942, found 354.0939; HPLC conditions for determination of enantiomeric excess: Chiralpak IA, λ = 254 nm, hexane : 2-propanol = 80:20, flow rate = 1.0 mL/min, t_{major} = 12.1 min, t_{minor} = 26.8 min.

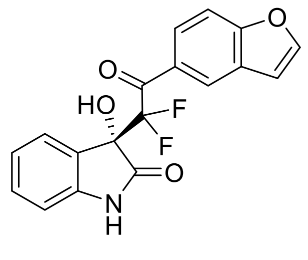

(*S*)-3-(2-(Benzofuran-5-yl)-1,1-difluoro-2-oxoethyl)-3-hydroxyindolin-2-one (5i). White solid. mp = 167 - 168 °C, 32.3 mg, 94% yield. 94% *ee*. $[\alpha]_D^{20}$ = -168.7 (c = 0.033, MeOH); ^{1}H NMR (400 MHz, DMSO-d_6) (δ, ppm) 10.60 (s, 1H), 8.52 (s, 1H), 8.15 (d, *J* = 2.2 Hz, 1H), 8.05 (d, *J* = 8.8 Hz, 1H), 7.77 (d, *J* = 8.8 Hz, 1H), 7.37 – 7.25 (m, 3H), 7.17 (d, *J* = 1.9 Hz, 1H), 7.04 – 6.93 (m, 1H), 6.88 (d, *J* = 7.7 Hz, 1H); ^{13}C NMR (100 MHz, DMSO-d_6) (δ, ppm) 187.1 (t, *J* = 28.2 Hz), 173.4, 157.3, 147.9, 143.0, 130.6, 128.0, 127.4, 126.8, 126.4, 125.7, 125.3, 121.7, 117.1 (t, *J* = 262.0 Hz),

111.7, 110.0 (d, J = 6.8 Hz), 107.7 (d, J = 7.0 Hz), 75.8 (t, J = 25.5 Hz); ^{19}F NMR (376 MHz, DMSO-d_6) (δ, ppm) -107.9 (d, J = 272.5 Hz, 1F), -108.7 (d, J = 272.5 Hz, 1F); HRMS (TOF MS ESI$^+$) calculated for $C_{18}H_{12}F_2NO_4$ [M+H]$^+$: 344.0734, found 344.0731; HPLC conditions for determination of enantiomeric excess: Chiralpak IA, λ = 254 nm, hexane : 2-propanol = 80:20, flow rate = 1.0 mL/min, t_{major} = 11.7 min, t_{minor} = 25.1 min.

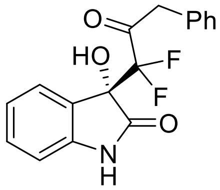

(*S*)-3-(1,1-Difluoro-2-oxo-3-phenylpropyl)-3-hydroxyindolin-2-one (5j). White solid. mp = 156 - 157 °C, 25.7 mg, 81% yield. 93% *ee*. $[\alpha]_D^{20}$ = -158.7 (c = 0.033, MeOH); ^{1}H NMR (400 MHz, DMSO-d_6) (δ, ppm) 10.68 (s, 1H), 7.48 (s, 1H), 7.40 (d, J = 7.2 Hz, 1H), 7.36 – 7.25 (m, 4H), 7.17 – 7.12 (m, 2H), 7.09 – 7.02 (m, 1H), 6.88 (d, J = 7.6 Hz, 1H), 4.27 (d, J = 18.2 Hz, 1H), 4.11 (d, J = 18.2 Hz, 1H); ^{13}C NMR (100 MHz, DMSO-d_6) (δ, ppm) 196.1 (t, J = 27.3 Hz), 173.3, 142.8, 133.0, 130.9, 129.9, 128.3, 126.9, 126.1, 125.7, 122.1, 115.6 (t, J = 261.0 Hz), 110.2, 76.5 (t, J = 26.7 Hz), 44.9; ^{19}F NMR (376 MHz, DMSO-d_6) (δ, ppm) -117.1 (d, J = 253.4 Hz, 1F), -117.78 (d, J = 253.4 Hz, 1F); HRMS (TOF MS ESI$^+$) calculated for $C_{17}H_{14}F_2NO_3$ [M+H]$^+$: 318.0942, found 318.0939; HPLC conditions for determination of enantiomeric excess: Chiralpak IA, λ = 254 nm, hexane : 2-propanol = 85:15, flow rate = 1.0 mL/min, t_{major} = 13.3 min, t_{minor} = 16.5 min.

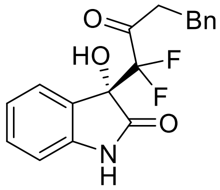

(*S*)-3-(1,1-Difluoro-2-oxo-4-phenylbutyl)-3-hydroxyindolin-2-one (5k). Yellow oil, 30.2 mg, 91% yield. 90% *ee*. $[\alpha]_D^{20}$ = -188.7 (c = 0.033, MeOH); ^{1}H NMR (400 MHz, $CDCl_3$) (δ, ppm) 8.37 (s, 1H), 7.41 (d, J = 7.1 Hz, 1H), 7.37 – 7.29 (m, 1H), 7.27 –

7.22 (m, 2H), 7.21 – 7.16 (m, 1H), 7.14 – 7.10 (m, 2H), 7.07 (t, J = 7.3 Hz, 1H), 6.86 (d, J = 7.9 Hz, 1H), 4.70 (s, 1H), 3.08 – 2.80 (m, 4H); ^{13}C NMR (100 MHz, $CDCl_3$) (δ, ppm) 200.4 (dd, J = 32.7, 25.5 Hz), 174.6, 141.6, 140.0, 131.6, 128.7, 128.5, 126.6, 126.5, 123.9, 123.7, 113.5 (dd, J = 265.1, 262.0 Hz), 111.0, 77.3 (t, J = 24.4 Hz) 40.1, 28.4; 19F NMR (376 MHz, $CDCl_3$) (δ, ppm) -116.0 (d, J = 276.3 Hz, 1F), -119.1 (d, J = 276.3 Hz, 1F); HRMS (TOF MS ESI$^+$) calculated for $C_{18}H_{16}F_2NO_3$ $[M+H]^+$: 332.1098, found 332.1095; HPLC conditions for determination of enantiomeric excess: Chiralpak OD-H, λ = 254 nm, hexane : 2-propanol = 85:15, flow rate = 1.0 mL/min, t_{major} = 12.4 min, t_{minor} = 15.5 min.

To a 10-mL oven-dried vial containing a magnetic stirring bar, H_2O (0.15 mmol, 2.7 μL, 1.5 equiv.), $Rh_2(esp)_2$ (0.76 mg, 1.0 mol%), and chiral phosphoric acid **CPA-1** (1.8 mg, 2.0 mol%) in ethyl acetate (EA, 1.0 mL), was added a solution of diazo compound **1** (0.15 mmol, 1.5 equiv.) and imine **6** (0.1 mmol) in 1.0 mL EA *via* syringe pump over 2 h under argon atmosphere at 30 °C. After addition, the reaction mixture was stirred for additional 1~2 h under these conditions until consumption of the material (monitored by TLC). Then the reaction mixture was purified by column chromatography on silica gel without any additional treatment (Hexanes : EtOAc = 50:1 to 20:1) to give the pure products **7** in good to high yields and excellent enantioselectivity.

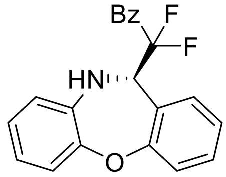

(*R*)-2-(10,11-Dihydrodibenzo[*b,f*][1,4]oxazepin-11-yl)-2,2-difluoro-1-phenylethan-1-one (7a). White solid. mp = 89 - 90 °C, 33.4 mg, 95% yield. 94% *ee*. $[\alpha]_D^{20}$ = -18.7 (c = 0.033, MeOH); ^{1}H NMR (400 MHz, $CDCl_3$) (δ, ppm) 8.02 – 7.96 (m, 2H), 7.61 – 7.53 (m, 1H), 7.44 – 7.39 (m, 2H), 7.37 – 7.30 (m, 1H), 7.27 – 7.21 (m, 2H), 7.10 – 7.02 (m, 2H), 6.90 – 6.83 (m, 1H), 6.75 – 6.68 (m, 1H), 6.61 (d, J =

7.9 Hz, 1H), 5.26 – 5.06 (m, 1H), 4.40 (d, J = 6.0 Hz, 1H); ^{13}C NMR (100 MHz, $CDCl_3$) (δ, ppm) 190.9 (dd, J = 30.6, 29.8 Hz), 158.2, 144.7, 136.7, 134.5, 132.7 (t, J = 2.4 Hz), 131.8, 130.9, 130.3 (t, J = 3.5 Hz), 128.7, 124.9, 124.2, 124.0, 121.8, 121.7, 120.0, 118.7, 117.6 (dd, J = 264.9, 261.5 Hz), 60.9 (dd, J = 26.5, 24.1 Hz); ^{19}F NMR (376 MHz, $CDCl_3$) (δ, ppm) -102.0 (d, J = 281.4 Hz, 1F), -109.1 (d, J = 281.4 Hz, 1F); HRMS (TOF MS ESI$^+$) calculated for $C_{21}H_{16}F_2NO_2$ [M+H]$^+$: 352.1149, found 352.1145; HPLC conditions for determination of enantiomeric excess: Chiralpak AD-H, λ = 254 nm, hexane : 2-propanol = 75:25, flow rate = 1.0 mL/min, t_{major} = 9.6 min, t_{minor} = 12.4 min.

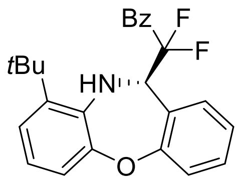

(*R*)-2-(9-(*tert*-Butyl)-10,11-dihydrodibenzo[*b,f*][1,4]oxazepin-11-yl)-2,2-difluoro-1-phenylethan-1-one (7b). White solid. mp = 101 - 102 °C, 34.6 mg, 85% yield. 90% *ee*. $[\alpha]_D^{20}$ = -20.0 (c = 0.033, MeOH); ^{1}H NMR (400 MHz, $CDCl_3$) (δ, ppm) 8.05 – 7.98 (m, 2H), 7.64 – 7.53 (m, 1H), 7.45 – 7.37 (m, 2H), 7.35 – 7.28 (m, 1H), 7.26 – 7.19 (m, 2H), 7.10 – 7.04 (m, 1H), 7.02 (d, J = 8.4 Hz, 1H), 6.76 – 6.70 (m, 1H), 6.62 (d, J = 2.3 Hz, 1H), 5.32 – 5.09 (m, 1H), 4.38 (d, J = 5.0 Hz, 1H), 1.22 (s, 9H); ^{13}C NMR (100 MHz, $CDCl_3$) (δ, ppm) 191.0 (t, J = 30.3 Hz), 158.4, 147.9, 142.6, 135.7, 134.4, 132.7 (t, J = 4.2 Hz), 131.8, 130.9, 130.3 (t, J = 3.1 Hz), 128.6, 124.1, 121.6, 121.2, 117.6 (dd, J = 264.7, 261.3 Hz), 117.1, 115.8, 60.9 (dd, J = 26.4, 24.1 Hz), 34.3, 31.4; ^{19}F NMR (376 MHz, $CDCl_3$) (δ, ppm) -101.9 (d, J = 280.6 Hz, 1F), -109.2 (d, J = 280.6 Hz, 1F); HRMS (TOF MS ESI$^+$) calculated for $C_{25}H_{24}F_2NO_2$ [M+H]$^+$: 408.1775, found 408.1769; HPLC conditions for determination of enantiomeric excess: Chiralpak AD-H, λ = 254 nm, hexane : 2-propanol = 75:25, flow rate = 1.0 mL/min, t_{minor} = 5.2 min, t_{major} = 6.2 min.

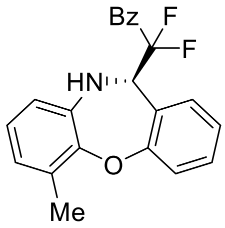

(*R*)-2,2-Difluoro-2-(6-methyl-10,11-dihydrodibenzo[*b,f*][1,4]oxazepin-11-yl)-1-phenylethan-1-one (7c). Yellow solid. mp = 109 - 112 °C, 31.2 mg, 85% yield. 92% *ee*. $[\alpha]_D^{20}$ = -19.3 (c = 0.033, MeOH); ^{1}H NMR (400 MHz, $CDCl_3$) (δ, ppm) 7.93 (d, *J* = 7.8 Hz, 2H), 7.52 – 7.42 (m, 1H), 7.37 – 7.28 (m, 2H), 7.27 – 7.21 (m, 1H), 7.20 – 7.13 (m, 2H), 7.04 – 6.95 (m, 1H), 6.68 – 6.60 (m, 1H), 6.50 (d, *J* = 7.4 Hz, 1H), 6.38 (d, *J* = 7.9 Hz, 1H), 5.19 – 5.02 (m, 1H), 4.24 (d, *J* = 5.9 Hz, 1H), 2.32 (s, 3H); ^{13}C NMR (100 MHz, $CDCl_3$) (δ, ppm) 190.9 (dd, *J* = 31.1, 29.4 Hz), 158.3, 143.9, 136.9, 134.4, 132.8 (t, *J* = 2.4 Hz), 131.92, 131.91, 130.8, 130.7, 130.3 (t, *J* = 3.4 Hz), 128.6, 124.3, 124.2, 122.0, 121.9, 117.4 (dd, *J* = 265.6, 260.3 Hz), 116.7, 60.8 (dd, *J* = 26.7, 23.9 Hz), 17.1; ^{19}F NMR (376 MHz, $CDCl_3$) (δ, ppm) -101.5 (d, *J* = 281.0 Hz, 1F), -110.1 (d, *J* = 281.0 Hz, 1F); HRMS (TOF MS ESI$^+$) calculated for $C_{22}H_{18}F_2NO_2$ $[M+H]^+$: 366.1306, found 366.1302; HPLC conditions for determination of enantiomeric excess: Chiralpak IA, λ = 254 nm, hexane : 2-propanol = 90:10, flow rate = 1.0 mL/min, t_{major} = 9.8 min, t_{minor} = 10.8 min.

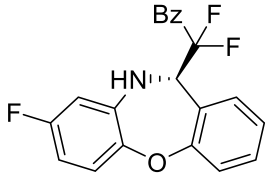

(*R*)-2,2-Difluoro-2-(8-fluoro-10,11-dihydrodibenzo[*b,f*][1,4]oxazepin-11-yl)-1-phenylethan-1-one (7d). White solid. mp = 94 - 95 °C, 33.6 mg, 91% yield. 94% *ee*. $[\alpha]_D^{20}$ = -13.3 (c = 0.033, MeOH); ^{1}H NMR (400 MHz, $CDCl_3$) (δ, ppm) 8.08 – 7.96 (m, 2H), 7.62 – 7.52 (m, 1H), 7.46 – 7.39 (m, 2H), 7.37 – 7.31 (m, 1H), 7.27 – 7.18 (m, 2H), 7.14 – 7.08 (m, 1H), 7.05 – 6.95 (m, 1H), 6.42 – 6.26 (m, 2H), 5.22 – 5.10 (m, 1H), 4.49 (d, *J* = 6.5 Hz, 1H); ^{13}C NMR (100 MHz, $CDCl_3$) (δ, ppm) 190.6 (t, *J* = 30.1 Hz), 159.6 (d, *J* = 240.8 Hz), 158.0, 140.6 (d, *J* = 2.3 Hz), 137.8 (d, *J* = 10.9 Hz),

134.5, 132.4 (t, J = 2.2 Hz), 131.9, 131.1, 130.2 (t, J = 3.3 Hz), 128.6, 124.5, 123.8, 122.6, 122.5, 121.5, 117.2 (dd, J = 264.9, 261.5 Hz), 105.2 (dd, J = 108.9, 24.9 Hz), 60.5 (dd, J = 27.0, 24.5 Hz); ^{19}F NMR (376 MHz, $CDCl_3$) (δ, ppm) -102.1 (d, J = 282.3 Hz, 1F), -109.1 (d, J = 282.3 Hz, 1F), -118.7 (s, 1F); HRMS (TOF MS ESI$^+$) calculated for $C_{21}H_{15}F_3NO_2$ $[M+H]^+$: 370.1055, found 370.1051; HPLC conditions for determination of enantiomeric excess: Chiralpak IA-3, λ = 254 nm, hexane : 2-propanol = 85:15, flow rate = 1.0 mL/min, t_{major} = 10.5 min, t_{minor} = 14.3 min.

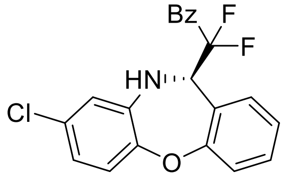

(*R*)-2-(8-Chloro-10,11-dihydrodibenzo[*b,f*][1,4]oxazepin-11-yl)-2,2-difluoro-1-phenylethan-1-one (7e). White solid. mp = 90 - 91 °C, 35.5 mg, 92% yield. 92% *ee*. $[\alpha]_D^{20}$ = -16.3 (c = 0.033, MeOH); ^{1}H NMR (400 MHz, $CDCl_3$) (δ, ppm) 8.06 – 7.95 (m, 2H), 7.61 – 7.55 (m, 1H), 7.47 – 7.39 (m, 2H), 7.37 – 7.31 (m, 1H), 7.25 – 7.17 (m, 2H), 7.15 – 7.05 (m, 1H), 6.99 (d, J = 8.5 Hz, 1H), 6.67 – 6.56 (m, 2H), 5.20 – 4.99 (m, 1H), 4.47 (d, J = 6.5 Hz, 1H); ^{13}C NMR (100 MHz, $CDCl_3$) (δ, ppm) 190.6 (dd, J = 30.7, 29.7 Hz), 157.8, 143.0, 137.7, 134.6, 132.5 (t, J = 2.5 Hz), 132.0, 131.2, 130.3 (t, J = 3.4 Hz), 129.6, 128.7, 124.5, 123.8, 122.9, 121.6, 119.5, 117.9, 117.4 (dd, J = 265.1, 261.8 Hz), 60.6 (dd, J = 27.0, 24.2 Hz); ^{19}F NMR (376 MHz, $CDCl_3$) (δ, ppm) -102.0 (d, J = 283.2 Hz, 1F), -109.0 (d, J = 283.2 Hz, 1F); HRMS (TOF MS ESI$^+$) calculated for $C_{21}H_{15}ClF_2NO_2$ $[M+H]^+$: 386.0759, found 386.0754; HPLC conditions for determination of enantiomeric excess: Chiralpak IA, λ = 254 nm, hexane : 2-propanol = 90:10, flow rate = 1.0 mL/min, t_{major} = 13.8 min, t_{minor} = 15.4 min.

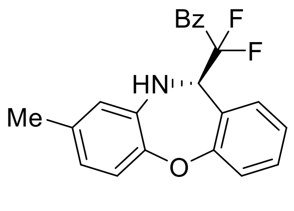

(*R*)-2,2-Difluoro-2-(8-methyl-10,11-dihydrodibenzo[*b,f*][1,4]oxazepin-11-yl)-1-phenylethan-1-one (7f). Yellow solid. mp = 104 - 105 °C, 32.2 mg, 88% yield. 93% *ee*. $[\alpha]_D^{20}$ = -18.2 (c = 0.033, MeOH); ^{1}H NMR (400 MHz, $CDCl_3$) (δ, ppm) 8.05 – 7.93 (m, 2H), 7.62 – 7.50 (m, 1H), 7.46 – 7.39 (m, 2H), 7.36 – 7.28 (m, 1H), 7.25 – 7.18 (m, 2H), 7.12 – 7.03 (m, 1H), 6.98 (d, *J* = 8.1 Hz, 1H), 6.57 – 6.47 (m, 1H), 6.43 (d, *J* = 1.4 Hz, 1H), 5.16 (m, 1H), 4.34 (d, *J* = 6.1 Hz, 1H), 2.17 (s, 3H); ^{13}C NMR (100 MHz, $CDCl_3$) (δ, ppm) 191.0 (dd, *J* = 30.8, 29.5 Hz), 158.3, 142.7, 136.2, 134.5, 134.4, 132.8 (t, *J* = 2.3 Hz), 131.8 (d, *J* = 1.0 Hz), 130.9, 130.3 (t, *J* = 3.5 Hz), 128.6 , 124.13, 124.10, 121.6, 121.5, 120.6, 119.1, 117.6 (dd, *J* = 264.8, 261.4 Hz), 60.9 (dd, *J* = 26.5, 24.1 Hz), 20.7; ^{19}F NMR (376 MHz, $CDCl_3$) (δ, ppm) -102.1 (d, *J* = 280.3 Hz, 1F), -109.0 (d, *J* = 280.3 Hz, 1F); HRMS (TOF MS ESI$^+$) calculated for $C_{22}H_{18}F_2NO_2$ $[M+H]^+$: 366.1306, found 366.1301; HPLC conditions for determination of enantiomeric excess: Chiralpak IA, λ = 254 nm, hexane : 2-propanol = 80:20, flow rate = 1.0 mL/min, t_{major} = 8.2 min, t_{minor} = 8.8 min.

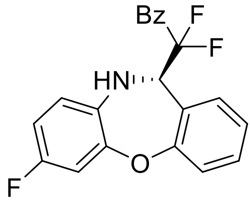

(*R*)-2,2-Difluoro-2-(7-fluoro-10,11-dihydrodibenzo[*b,f*][1,4]oxazepin-11-yl)-1-phenylethan-1-one (7g). Yellow solid. mp = 101 - 103 °C, 33.6 mg, 91% yield. 95% *ee*. $[\alpha]_D^{20}$ = -16.3 (c = 0.033, MeOH); ^{1}H NMR (400 MHz, $CDCl_3$) (δ, ppm) 8.04 – 7.95 (m, 2H), 7.61 – 7.56 (m, 1H), 7.45 – 7.39 (m, 2H), 7.37 – 7.31 (m, 1H), 7.27 – 7.19 (m, 2H), 7.13 – 7.07 (m, 1H), 6.86 – 6.82 (m, 1H), 6.64 – 6.52 (m, 2H), 5.20 – 5.10 (m, 1H), 4.33 (s, 1H); ^{13}C NMR (100 MHz, $CDCl_3$) (δ, ppm) 190.7 (dd, *J* = 30.8, 29.6 Hz), 157.6, 157.5, 155.7, 145.2 (d, *J* = 10.7 Hz), 134.6, 132.8 (d, *J* = 2.9 Hz), 132.6 (t, *J* = 2.3 Hz), 131.8 (d, *J* = 1.4 Hz), 130.9, 130.3 (t, *J* = 3.4 Hz), 128.7, 124.3,

123.7, 121.6, 119.3 (d, J = 8.9 Hz), 117.5 (dd, J = 265.1, 261.5 Hz), 110.1 (dd, J = 304.6, 23.4 Hz), 60.8 (dd, J = 26.0, 23.7 Hz); ^{19}F NMR (376 MHz, $CDCl_3$) (δ, ppm) -101.3 (d, J = 284.2 Hz, 1F), -108.9 (d, J = 284.2 Hz, 1F). -123.6 (s, 1F); HRMS (TOF MS ESI$^+$) calculated for $C_{21}H_{15}F_3NO_2$ $[M+H]^+$: 370.1055, found 370.1052; HPLC conditions for determination of enantiomeric excess: Chiralpak IA-3, λ = 254 nm, hexane : 2-propanol = 85:15, flow rate = 1.0 mL/min, t_{major} = 9.3 min, t_{minor} = 12.4 min.

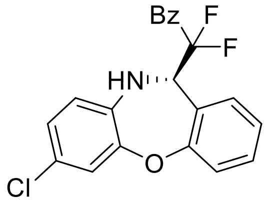

(*R*)-2-(7-Chloro-10,11-dihydrodibenzo[*b,f*][1,4]oxazepin-11-yl)-2,2-difluoro-1-phenylethan-1-one (7h). Yellow oil. 35.5 mg, 92% yield. 92% *ee*. $[\alpha]_D^{20}$ = -11.3 (c = 0.033, MeOH); ^{1}H NMR (400 MHz, $CDCl_3$) (δ, ppm) 7.05 – 6.96 (m, 2H), 6.64 – 6.54 (m, 1H), 6.46 – 6.39 (m, 2H), 6.37 – 6.31 (m, 1H), 6.25 – 6.19 (m, 2H), 6.12 – 6.06 (m, 2H), 5.85 – 5.80 (m, 1H), 5.53 (d, J = 8.5 Hz, 1H), 4.23 – 4.03 (m, 1H), 3.45 (d, J = 6.4 Hz, 1H); ^{13}C NMR (100 MHz, $CDCl_3$) (δ, ppm) 190.7 (dd, J = 30.7, 29.7 Hz), 157.6, 144.7, 135.4, 134.6, 132.5 (t, J = 2.4 Hz), 131.9 (d, J = 1.1 Hz), 131.1, 130.3 (t, J = 3.4 Hz), 128.7, 124.7, 124.5, 123.9, 123.8, 121.9, 121.7, 119.3, 117.4 (dd, J = 265.1, 261.9 Hz), 60.7 (dd, J = 26.6, 24.0 Hz); ^{19}F NMR (376 MHz, $CDCl_3$) (δ, ppm) -101.8 (d, J = 283.5 Hz, 1F), -108.9 (d, J = 283.5 Hz, 1F); HRMS (TOF MS ESI$^+$) calculated for $C_{21}H_{15}ClF_2NO_2$ $[M+H]^+$: 386.0759, found 386.0757; HPLC conditions for determination of enantiomeric excess: Chiralpak IA, λ = 254 nm, hexane : 2-propanol = 90:10, flow rate = 1.0 mL/min, t_{major} = 12.8 min, t_{minor} = 17.3 min.

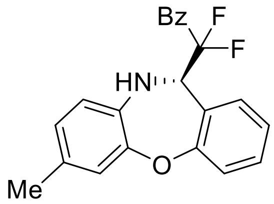

(*R*)-2,2-Difluoro-2-(7-methyl-10,11-dihydrodibenzo[*b,f*][1,4]oxazepin-11-yl)-1-phenylethan-1-one (7i). Yellow solid. mp = 101 - 102 °C, 32.2 mg, 88% yield. 92% *ee*. $[\alpha]_D^{20}$ = -22.3 (c = 0.033, MeOH); ^{1}H NMR (400 MHz, $CDCl_3$) (δ, ppm) 8.04 – 7.93 (m, 2H), 7.61 – 7.52 (m, 1H), 7.45 – 7.37 (m, 2H), 7.35 – 7.29 (m, 1H), 7.26 – 7.18 (m, 2H), 7.12 – 7.02 (m, 1H), 6.92 (s, 1H), 6.68 (d, *J* = 7.9 Hz, 1H), 6.52 (d, *J* = 8.0 Hz, 1H), 5.24 – 5.07 (m, 1H), 4.30 (s, 1H), 2.21 (s, 3H); ^{13}C NMR (100 MHz, $CDCl_3$) (δ, ppm) 191.0 (dd, *J* = 30.8, 29.5 Hz), 158.1, 144.7, 134.4, 133.9, 132.8 (t, *J* = 2.4 Hz), 131.7 (d, *J* = 1.2 Hz), 130.8, 130.3 (t, *J* = 3.5 Hz), 130.0, 128.6, 125.3, 124.04, 124.00, 122.0, 121.6, 118.8, 117.7 (dd, *J* = 264.8, 261.4 Hz), 61.0 (dd, *J* = 26.1, 23.9 Hz), 20.4; ^{19}F NMR (376 MHz, $CDCl_3$) (δ, ppm) -101.8 (d, *J* = 281.0 Hz, 1F), -109.0 (d, *J* = 281.0 Hz, 1F); HRMS (TOF MS ESI$^+$) calculated for $C_{22}H_{18}F_2NO_2$ $[M+H]^+$: 366.1306, found 366.1301; HPLC conditions for determination of enantiomeric excess: Chiralpak IA, λ = 254 nm, hexane : 2-propanol = 90:10, flow rate = 1.0 mL/min, t_{major} = 12.1 min, t_{minor} = 14.5 min.

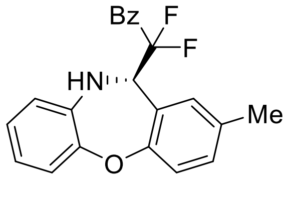

(*R*)-2,2-Difluoro-2-(2-methyl-10,11-dihydrodibenzo[*b,f*][1,4]oxazepin-11-yl)-1-phenylethan-1-one (7j). Yellow solid. mp = 101 - 102 °C, 29.6 mg, 81% yield. 93% *ee*. $[\alpha]_D^{20}$ = -24.3 (c = 0.033, MeOH); ^{1}H NMR (400 MHz, $CDCl_3$) (δ, ppm) 8.12 – 7.96 (m, 2H), 7.64 – 7.53 (m, 1H), 7.43 – 7.37 (m, 2H), 7.17 – 6.99 (m, 4H), 6.93 – 6.81 (m, 1H), 6.75 – 6.66 (m, 1H), 6.64 – 6.54 (m, 1H), 5.27 – 4.99 (m, 1H), 4.39 (d, *J* = 5.7 Hz, 1H), 2.29 (s, 3H); ^{13}C NMR (100 MHz, $CDCl_3$) (δ, ppm) 191.0 (t, *J* = 30.0 Hz), 156.0, 144.8, 136.7, 134.4, 133.8, 132.8 (t, *J* = 2.0 Hz), 132.2, 131.4, 130.3 (t, *J* = 3.2 Hz), 128.6, 124.8, 123.6, 121.7, 121.4, 119.8, 118.5, 117.6 (dd, *J* = 264.5, 261.1

Hz), 60.9 (dd, J = 26.6, 24.4 Hz), 20.7; ^{19}F NMR (376 MHz, $CDCl_3$) (δ, ppm) -101.9 (d, J = 279.8 Hz, 1F), -109.2 (d, J = 279.8 Hz, 1F); HRMS (TOF MS ESI$^+$) calculated for $C_{22}H_{18}F_2NO_2$ [M+H]$^+$: 366.1306, found 366.1302; HPLC conditions for determination of enantiomeric excess: Chiralpak IA, λ = 254 nm, hexane : 2-propanol = 90:10, flow rate = 1.0 mL/min, t_{major} = 9.8 min, t_{minor} = 20.5 min.

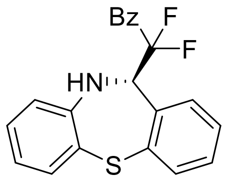

(*R*)-2-(10,11-Dihydrodibenzo[*b,f*][1,4]thiazepin-11-yl)-2,2-difluoro-1-phenylethan-1-one (7k). Yellow solid. mp = 99 - 100 °C, 34.9 mg, 95% yield. 90% *ee*. $[\alpha]_D^{20}$ = -18.9 (c = 0.033, MeOH); ^{1}H NMR (400 MHz, $CDCl_3$) (δ, ppm) 8.15 – 7.97 (m, 2H), 7.68 – 7.61 (m, 1H), 7.60 – 7.55 (m, 1H), 7.53 – 7.46 (m, 2H), 7.34 – 7.24 (m, 2H), 7.20 (d, J = 7.7 Hz, 1H), 6.98 – 6.87 (m, 1H), 6.80 – 6.65 (m, 2H), 6.54 (d, J = 8.0 Hz, 1H), 4.08 (s, 1H); ^{13}C NMR (100 MHz, $CDCl_3$) (δ, ppm) 189.3 (dd, J = 29.2, 28.3 Hz), 144.6, 138.2, 136.8, 134.5, 132.6 (t, J = 3.2 Hz), 132.5, 132.31, 132.30, 130.1, 129.1, 128.9, 128.8, 128.6, 127.4 (t, J = 3.1 Hz), 120.9 (d, J = 4.5 Hz), 120.3, 118.0 (dd, J = 261.4, 259.8 Hz), 58.8 (dd, J = 28.5, 26.4 Hz); ^{19}F NMR (376 MHz, $CDCl_3$) (δ, ppm) -102.1 (d, J = 279.8 Hz, 1F), -110.8 (d, J = 279.8 Hz, 1F); HRMS (TOF MS ESI$^+$) calculated for $C_{21}H_{16}F_2NOS$ [M+H]$^+$: 368.0921, found 368.0919; HPLC conditions for determination of enantiomeric excess: Chiralpak ID-3, λ = 254 nm, hexane : 2-propanol = 97:03, flow rate = 1.0 mL/min, t_{minor} = 8.3 min, t_{major} = 9.2 min.

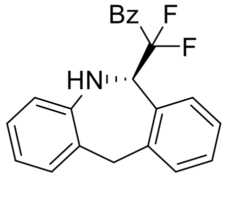

(*R*)-2-(6,11-Dihydro-5*H*-dibenzo[*b,e*]azepin-6-yl)-2,2-difluoro-1-phenylethan-1-one (7l). Yellow solid. mp = 129 - 130 °C, 27.3 mg, 78% yield. 90% *ee*. $[\alpha]_D^{20}$ = -20.9

(c = 0.033, MeOH); ^{1}H NMR (400 MHz, $CDCl_3$) (δ, ppm) 8.02 – 7.80 (m, 2H), 7.61 – 7.46 (m, 1H), 7.40 – 7.30 (m, 2H), 7.18 – 7.11 (m, 2H), 7.09 – 7.01 (m, 2H), 6.98 – 6.88 (m, 2H), 6.74 – 6.54 (m, 2H), 5.46 – 5.23 (m, 1H), 4.50 (d, *J* = 13.8 Hz, 1H), 4.03 (s, 1H), 3.40 (d, *J* = 13.8 Hz, 1H); ^{13}C NMR (100 MHz, $CDCl_3$) (δ, ppm) 191.3 (dd, *J* = 32.5, 29.2 Hz), 143.3, 142.0, 134.5, 132.9 (t, *J* = 3.8 Hz), 132.3, 132.29, 132.26, 130.3 (t, *J* = 2.9 Hz), 129.3, 128.9, 128.74, 128.71, 128.3, 127.6, 126.6, 121.3, 120.0, 117.3 (dd, *J* = 265.7, 256.7 Hz), 62.6 (dd, *J* = 25.9, 21.5 Hz), 39.4 (d, *J* = 4.7 Hz); ^{19}F NMR (376 MHz, $CDCl_3$) (δ, ppm) -100.7 (d, *J* = 283.1 Hz, 1F), -113.1 (d, *J* = 283.1 Hz, 1F); HRMS (TOF MS ESI$^+$) calculated for $C_{22}H_{18}F_2NO$ $[M+H]^+$: 350.1356, found 350.1351; HPLC conditions for determination of enantiomeric excess: Chiralpak IA, λ = 254 nm, hexane : 2-propanol = 90:10, flow rate = 1.0 mL/min, t_{major} = 7.6 min, t_{minor} = 9.7 min.

4. Control Experiment

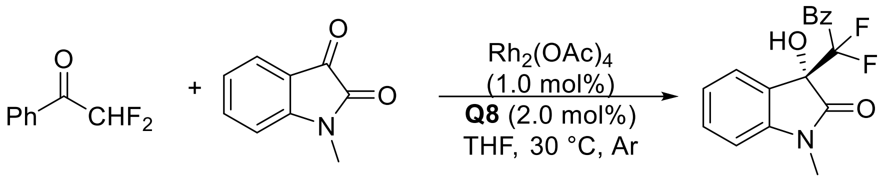

20 **3a** **4a**, <5%

To a 10-mL oven-dried vial containing a magnetic stirring bar, **20** (15.6 mg, 0.1 mmol), isatin **3a** (16.1 mg, 0.1 mmol), $Rh_2(OAc)_4$ (0.45 mg, 1.0 mol%), organocatalyst **Q8** (1.2 mg, 2.0 mol%) and tetrahydrofuran (THF, 2.0 mL) were added sequentially under argon atmosphere at 30 °C. The reaction mixture was stirred for 2 h. The resulting reaction mixture was concentrated under reduced pressure and the residue was directly subjected to proton NMR analysis with $CDCl_3$ as the solvent without any further purification. Most of the materials **20** and **3a** remained intact and no addition product **4a** was observed (Figure S2 below for details).

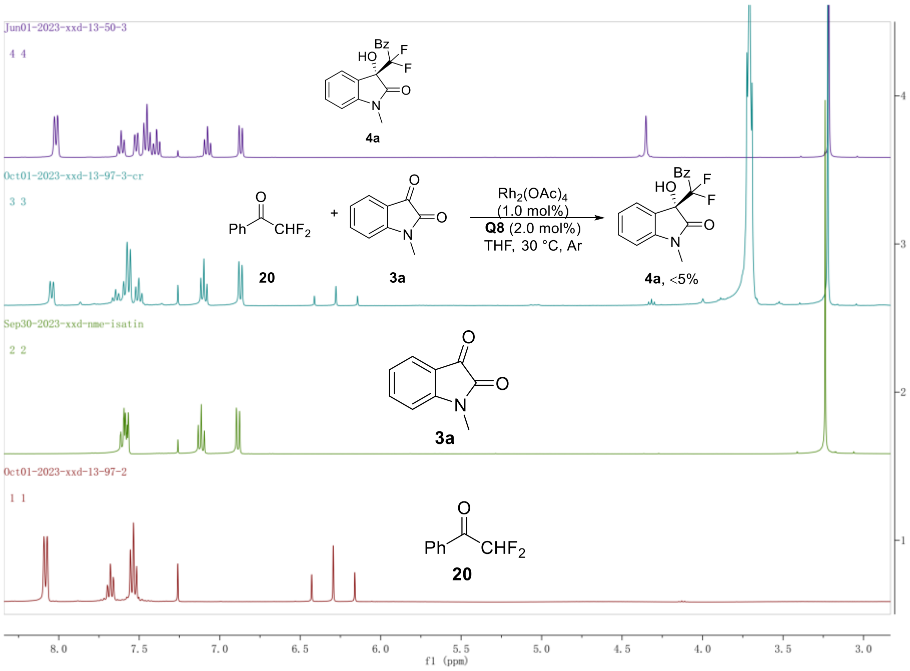

Figure S2. Proton NMR spectra of the crude reaction mixture of control experiment with **20** and **3a** under optimal conditions.

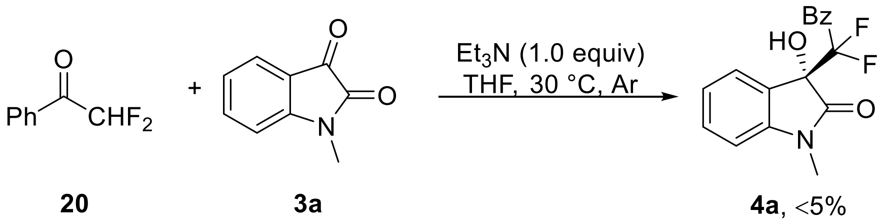

To a 10-mL oven-dried vial containing a magnetic stirring bar, **20** (15.6 mg, 0.1 mmol), isatin **3a** (16.1 mg, 0.1 mmol), organocatalyst **Q8** (1.2 mg, 2.0 mol%), Et_3N (10.1 mg, 0.1 mmol) and tetrahydrofuran (THF, 2.0 mL) were added sequentially under argon atmosphere at 30 °C. The reaction mixture was stirred for 2 h. The resulting reaction mixture was concentrated under reduced pressure and the residue was directly subjected to proton NMR analysis with $CDCl_3$ as the solvent without any further purification. Most of the materials **20** and **3a** remained intact and no addition product **4a** was observed (Figure S3 below for details).

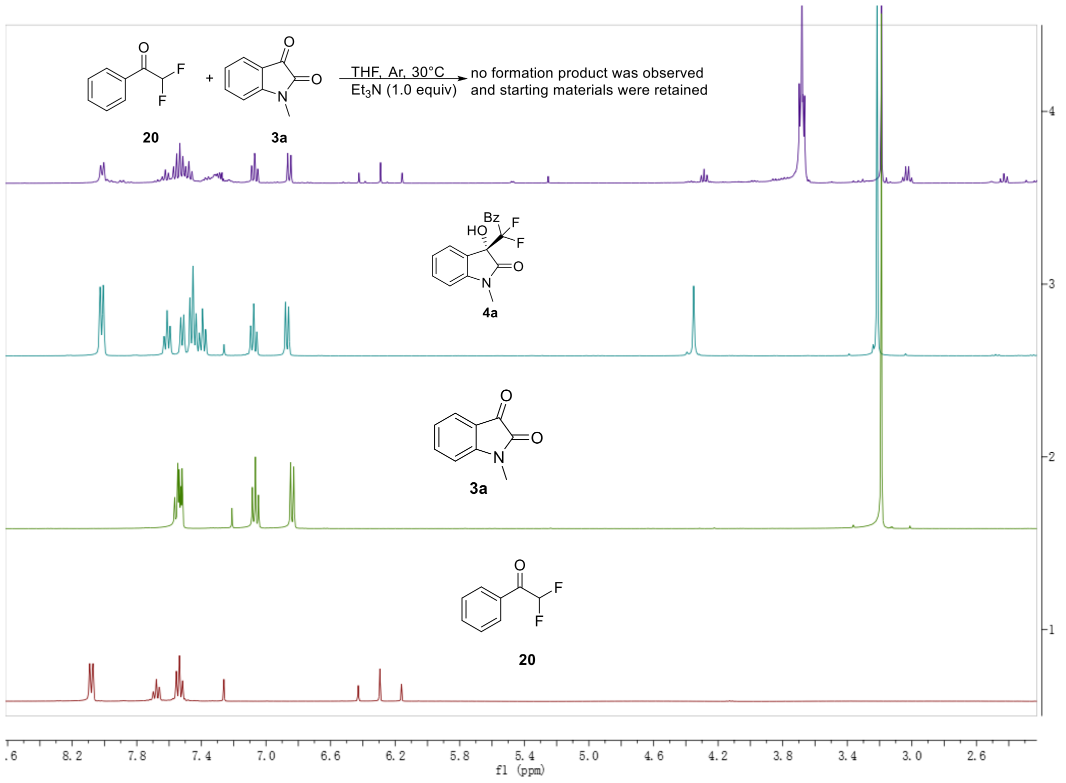

Figure S3. Proton NMR spectra of the crude reaction mixture of control experiment with **20** and **3a** in the presence of Et_3N.

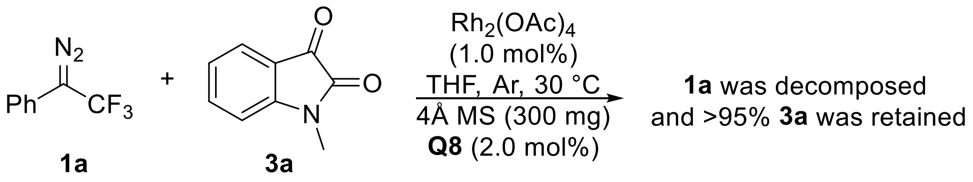

To a 10-mL oven-dried vial containing a magnetic stirring bar, isatin **3a** (16.1 mg, 0.1 mmol), $Rh_2(OAc)_4$ (0.45 mg, 1.0 mol%), organocatalyst **Q8** (1.2 mg, 2.0 mol%) and 4Å molecular sieve (300 mg) in tetrahydrofuran (THF, 1.0 mL), was added a solution of diazo compound **1a** (27.9 mg, 0.15 mmol, 1.5 equiv.) in 1.0 mL THF *via* syringe pump over 1 h under argon atmosphere at 30 °C. The reaction mixture was stirred for 2 h. The resulting reaction mixture was concentrated under reduced pressure and the residue was directly subjected to proton NMR analysis with $CDCl_3$ as the solvent without any further purification. No formation of product was observed, and only decomposition of diazo compound **1a** was observed, while the most of isatin **3a** was retained. (Figure S4 below for details).

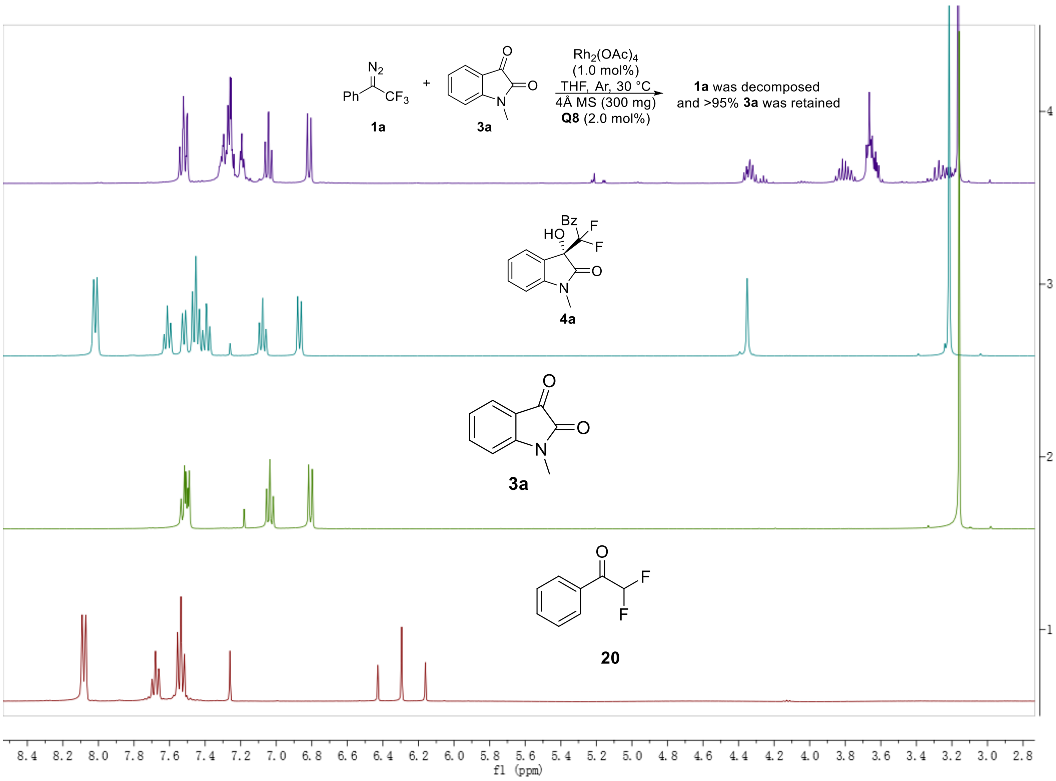

Figure S4. Proton NMR spectra of the crude reaction mixture of control experiment with **1a** and **3a** in absent of water.

5. Procedure of the Scale Up and Synthetic Applications

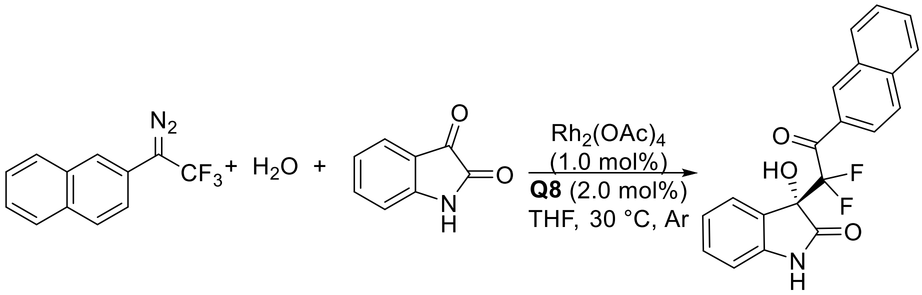

1h **2a** **3f** **5h**

To a 100-mL oven-dried vial with a magnetic stirring bar, isatin **3f** (588 mg, 4.0 mmol)**,** H_2O **2a** (108 mg, 6.0 mmol, 1.5 equiv.), $Rh_2(OAc)_4$ (18.0 mg, 1.0 mol%), organocatalyst **Q8** (48 mg, 2.0 mol%) in tetrahydrofuran (THF, 10.0 mL), was added a solution of diazo compound **1h** (1.42 g, 6.0 mmol, 1.5 equiv.) in 10.0 mL THF under argon atmosphere at 30 °C *via* syringe pump over 1 h under argon atmosphere at 30 °C. After addition, the reaction mixture was stirred for additional 2 h under these conditions until consumption of the material (monitored by TLC). Then the solvent

was evaporated in vacuo, the residue was purified by flash column chromatography on silica gel (Hexanes : EtOAc = 20:1 to 5:1) to give the 1.27 g pure product **5h** in 90% yield with 95% *ee*.

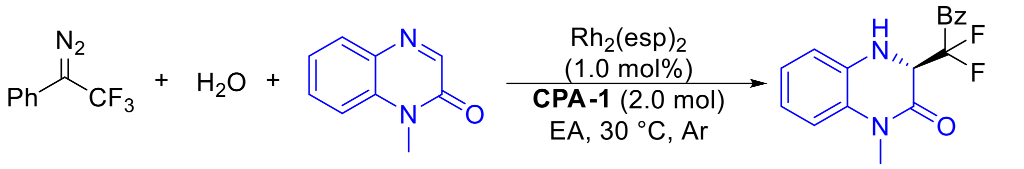

1a **2a** **8** **9**, 88% yield, 93% ee

Synthesis of 9: To a 10-mL oven-dried vial containing a magnetic stirring bar, H_2O (0.15 mmol, 2.7 μL, 1.5 equiv.), $Rh_2(esp)_2$ (0.76 mg, 1.0 mol%), and chiral phosphoric acid **CPA-1** (1.8 mg, 2.0 mol%) in ethyl acetate (EA, 1.0 mL), was added a solution of diazo compound **1a** (27.9 mg, 0.15 mmol, 1.5 equiv.) and **8** (16 mg, 0.1 mmol) in 1.0 mL EA *via* syringe pump over 2 h under argon atmosphere at 30 °C. After addition, the reaction mixture was stirred for additional 1~2 h under these conditions until consumption of the material (monitored by TLC). Then the reaction mixture was purified by column chromatography on silica gel without any additional treatment (Hexanes : EtOAc = 20:1 to 5:1) to give 27.8 mg of pure product **9** as yellow oil in 88% yield with 93% *ee.* ^{1}H NMR (400 MHz, $CDCl_3$) (δ, ppm) 8.10 – 7.88 (m, 2H), 7.67 – 7.56 (m, 1H), 7.52 – 7.42 (m, 2H), 6.98 – 6.81 (m, 3H), 6.73 (d, J = 7.7 Hz, 1H), 5.10 – 4.90 (m, 1H), 4.55 (s, 1H), 3.36 (s, 3H); ^{13}C NMR (100 MHz, $CDCl_3$) (δ, ppm) 189.0 (dd, J = 29.5, 26.8 Hz), 160.7 (d, J = 5.3 Hz), 134.4, 132.9, 132.7, 130.1, 128.8, 127.5, 124.2, 119.9, 116.9 (d, J = 10.2 Hz), 114.9 (d, J = 2.1 Hz), 114.2 (d, J = 2.4 Hz), 59.4 (dd, J = 30.2, 23.2 Hz), 29.2 (d, J = 2.3 Hz); ^{19}F NMR (376 MHz, $CDCl_3$) (δ, ppm) -107.3 (d, J = 280.4 Hz, 1F), -111.5 (d, J = 280.4 Hz, 1F); HRMS (TOF MS ESI+) calculated for $C_{17}H_{15}F_2N_2O_2$ $[M + H]^+$: 317.1102, found 317.1104; HPLC conditions for determination of enantiomeric excess: Chiralpak IA, λ = 254 nm, hexane : propanol = 80:20, flow rate =1.0 mL/min, t_{major} = 14.2 min, t_{minor} = 17.9 min.

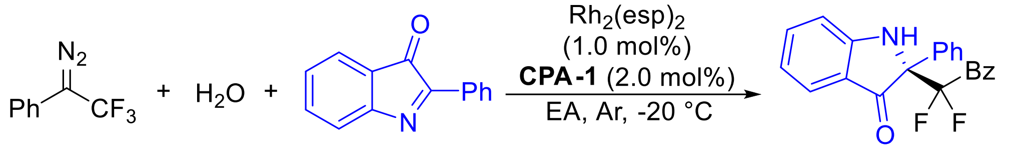

Synthesis of 11: To a 10-mL oven-dried vial containing a magnetic stirring bar, H_2O (0.15 mmol, 2.7 μL, 1.5 equiv.), $Rh_2(esp)_2$ (0.76 mg, 1.0 mol%) and chiral phosphoric acid **CPA-1** (1.8 mg, 2.0 mol%) in ethyl acetate (EA, 1.0 mL), was added a solution of diazo compound **1a** (27.9 mg, 0.15 mmol, 1.5 equiv.) and **10** (20.7 mg, 0.1 mmol) in 1.0 mL EA *via* syringe pump over 2 h under argon atmosphere at -20 °C. After addition, the reaction mixture was stirred for additional 1~2 h under these conditions until consumption of the material (monitored by TLC). Then the reaction mixture was purified by column chromatography on silica gel without any additional treatment (Hexanes : EtOAc = 20:1 to 5:1) to give 34.5 mg of pure product **11** as yellow oil in 95% yield with 93% *ee*. ^{1}H NMR (400 MHz, $CDCl_3$) (δ, ppm) 7.98 – 7.94 (m, 2H), 7.90 (d, J = 7.6 Hz, 2H), 7.64 (d, J = 7.7 Hz, 1H), 7.61 – 7.55 (m, 1H), 7.51 – 7.45 (m, 1H), 7.44 – 7.33 (m, 5H), 6.96 (d, J = 8.2 Hz, 1H), 6.91 – 6.79 (m, 1H), 5.90 (s, 1H); ^{13}C NMR (100 MHz, $CDCl_3$) (δ, ppm) 194.7, 188.5 (dd, J = 33.1, 30.3 Hz), 159.6, 137.6, 134.7, 132.5 (d, J = 2.5 Hz), 132.1 (t, J = 3.4 Hz), 130.3 (t, J = 2.4 Hz), 129.0, 128.7, 128.6, 127.3, 125.4, 120.7, 120.0, 116.9 (dd, J = 269.2, 265.5 Hz), 112.3, 71.0 (dd, J = 25.1, 22.0 Hz); ^{19}F NMR (376 MHz, $CDCl_3$) (δ, ppm) -102.8 (d, J = 299.8 Hz, 1F), -104.5 (d, J = 299.8 Hz, 1F); HRMS (TOF MS ESI+) calculated for $C_{22}H_{16}F_2NO_2$ $[M + H]^+$: 364.1149, found 364.1144; HPLC conditions for determination of enantiomeric excess: Chiralpak AD-H, λ = 254 nm, hexane : propanol = 75:25, flow rate =1.0 mL/min, t_{major} = 12.6 min, t_{minor} = 16.3 min.

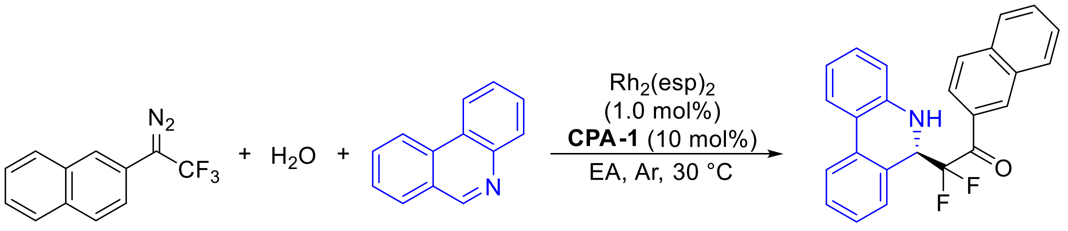

1h **12** **13**, 80%, 85% *ee*

Synthesis of 13: To a 10-mL oven-dried vial containing a magnetic stirring bar, H_2O (0.15 mmol, 2.7 μL, 1.5 equiv.), $Rh_2(esp)_2$ (0.76 mg, 1.0 mol%), and chiral phosphoric acid **CPA-1** (8.7 mg, 10 mol%) in ethyl acetate (EA, 1.0 mL), was added a solution of diazo compound **1h** (35.4 mg, 0.15 mmol, 1.5 equiv.) and **12** (17.9 mg, 0.1 mmol) in 1.0 mL EA *via* syringe pump over 2 h under argon atmosphere at 30 ℃. After addition, the reaction mixture was stirred for additional 1~2 h under these conditions until consumption of the material (monitored by TLC). Then the reaction mixture was purified by column chromatography on silica gel without any additional treatment (Hexanes : EtOAc = 50:1 to 20:1) to give 30.8 mg of pure product **13** as yellow oil in 80% yield with 85% *ee*. ^{1}H NMR (400 MHz, $CDCl_3$) (δ, ppm) 8.30 (s, 1H), 7.86 (d, J = 8.7 Hz, 1H), 7.83 – 7.70 (m, 4H), 7.62 – 7.55 (m, 2H), 7.52 – 7.43 (m, 1H), 7.38 – 7.32 (m, 1H), 7.28 – 7.17 (m, 2H), 7.09 – 6.99 (m, 1H), 6.83 – 6.72 (m, 1H), 6.59 (d, J = 7.9 Hz, 1H), 5.37 – 5.12 (m, 1H), 4.71 (s, 1H); ^{13}C NMR (100 MHz, $CDCl_3$) (δ, ppm) 190.8 (t, J = 29.6 Hz), 142.4, 135.9, 133.3 (t, J = 5.2 Hz), 133.0, 132.1, 130.2, 129.9, 129.49, 129.47, 129.44, 129.3, 128.3, 127.7, 127.0, 126.9, 125.7, 124.6, 123.1, 122.6, 120.2, 119.1, 117.5 (t, J = 264.8 Hz), 114.8, 57.3 (dd, J = 25.1, 23.4 Hz); ^{19}F NMR (376 MHz, $CDCl_3$) (δ, ppm) -104.6 (d, J = 283.3 Hz, 1F), -110.1 (d, J = 283.3 Hz, 1F); HRMS (TOF MS ESI+) calculated for $C_{25}H_{18}F_2NO$ $[M + H]^+$: 386.1356, found 386.1353; HPLC conditions for determination of enantiomeric excess: Chiralpak AD-H, λ = 254 nm, hexane : propanol = 90:10, flow rate =1.0 mL/min, t_{major} = 15.3 min, t_{minor} = 20.2 min.

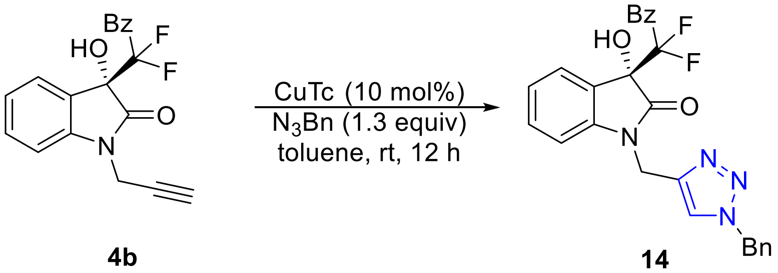

4b
90% *ee*

14
90% yield, 93% *ee*

Synthesis of 14: To a 10-mL oven-dried vial containing a magnetic stirring bar, CuTc (1.9 mg, 10 mol%), and **4b** (34.1 mg, 0.1 mmol) in toluene (0.5 mL), was added a solution of N_3Bn (17.3 mg, 0.13 mmol, 1.3 equiv.) in toluene (0.5 mL) dropwise at room temperature under a nitrogen atmosphere. After completion of the addition, the reaction mixture was stirred for additional 12 h under these conditions. When the reaction was completed (monitored by TLC), the solvent was removed under reduced pressure, yielding the triazole **14** in 90 % yields with 93% ee after separation by crystallization out in DCM. $[\alpha]_D^{20}$ = -154.0 (c = 0.033, MeOH); ^{1}H NMR (400 MHz, $CDCl_3$) (δ, ppm) 7.96 – 7.84 (m, 2H), 7.66 – 7.57 (m, 2H), 7.50 – 7.40 (m, 3H), 7.33 – 7.27 (comp, 4H), 7.24 – 7.18 (m, 2H), 7.10 – 6.97 (m, 2H), 5.45 (s, 2H), 5.13 (d, J = 15.8 Hz, 1H), 4.90 (d, J = 15.8 Hz, 1H), 4.62 (s, 1H); ^{13}C NMR (100 MHz, $CDCl_3$) (δ, ppm) 188.6 (dd, J = 30.9, 29.7 Hz), 172.5, 143.5, 142.7, 134.9, 134.5, 131.2 (t, J = 2.9 Hz), 131.4, 130.3, 129.2, 128.83, 128.80, 128.2, 126.1, 124.3, 123.5, 122.9, 116.0 (t, J = 265.5 Hz), 110.2, 76.2 (t, J = 23.5 Hz), 54.4, 36.1; ^{19}F NMR (376 MHz, $CDCl_3$) (δ, ppm) -108.2 (d, J = 301.0 Hz, 1F), -109.4 (d, J = 301.0 Hz, 1F); HRMS (TOF MS ESI$^+$) calculated for $C_{26}H_{21}F_2N4O_3$ $[M+H]^+$: 475.1582, found 475.1579; HPLC conditions for determination of enantiomeric excess: Chiralpak IA, λ = 254 nm, hexane : 2-propanol = 70:30, flow rate = 1.0 mL/min, t_{major} = 11.5 min, t_{minor} = 18.9 min.

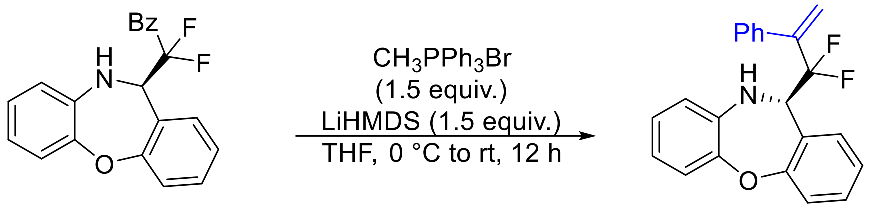

7a
94% *ee*

15
85% yield, 93% *ee*

***Synthesis of 15*:**[4] To a stirring suspension of methyl triphenyl phosphonium bromide (52.3 mg, 0.15 mmol) in dry THF (2.0 mL), was added LiHMDS (150 μL, 0.15 mmol, 1 M in THF) slowly at 0 °C under an argon atmosphere. After the mixture stirred for 30 min, the compound **7a** (52.7 mg, 0.1 mmol) was added, and then the mixture was gradually warmed to room temperature and continue stirring until full conversion. The reaction was quenched by addition of water at 0 °C, and the aqueous layer was extracted with EtOAc (5.0 mL X 3). The combined organic layer was washed with brine (5.0 mL) and dried over anhydrous Na_2SO_4. After filtered and evaporation of the solvent, the crude mixture was purified by silica gel column chromatography (ethyl acetate/petroleum ether = 1/15, v/v) to afford 29.7 mg pure **15** in 85% yield as yellow oil; 93% *ee*, $[\alpha]_D^{20}$ = -7.8 (c = 0.033, MeOH); ^{1}H NMR (500 MHz, $CDCl_3$) (δ, ppm) 7.44 – 7.30 (m, 5H), 7.30 – 7.21 (m, 1H), 7.15 (d, J = 8.1 Hz, 1H), 7.10 – 7.03 (m, 1H), 7.01 – 6.92 (m, 2H), 6.92 – 6.79 (m, 1H), 6.76 – 6.67 (m, 1H), 6.60 – 6.53 (m, 1H), 5.71 (s, 1H), 5.49 (s, 1H), 4.72 – 4.56 (m, 1H), 4.06 (s, 1H); ^{13}C NMR (125 MHz, $CDCl_3$) (δ, ppm) 157.6, 144.6, 143.1 (t, J = 22.1 Hz), 136.8, 136.6, 131.1, 130.2, 128.7, 128.6, 128.4, 125.2 (d, J = 2.2 Hz), 124.3, 123.4, 121.6 (d, J = 4.0 Hz), 121.5, 120.9 (dd, J = 9.7, 7.7 Hz), 119.9 (t, J = 3.6 Hz), 119.2, 118.9, 62.0 (t, J = 27.7 Hz); ^{19}F NMR (376 MHz, $CDCl_3$) (δ, ppm) -100.5 (d, J = 247.3 Hz, 1F), -102.0 (d, J = 247.3 Hz, 1F); HRMS (TOF MS ESI$^+$) calculated for $C_{22}H_{18}F_2NO$ $[M+H]^+$: 350.1356, found 350.1352; HPLC conditions for determination of enantiomeric excess: Chiralpak IF-3, λ = 254 nm, hexane : 2-propanol = 99:01, flow rate = 1.0 mL/min, t_{minor} = 8.7 min, t_{major} = 11.2 min.

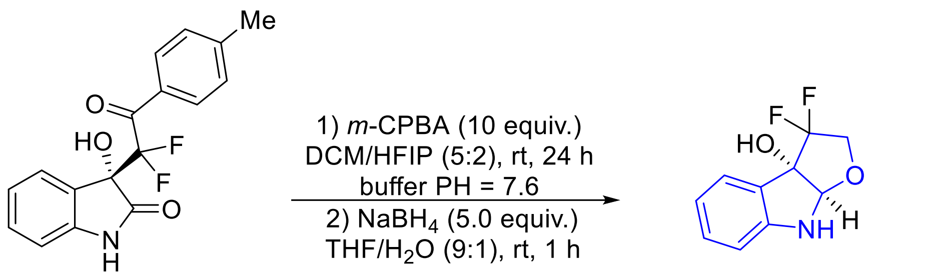

***Synthesis of 16*:**[5] The compound **5c** (31.7 mg, 0.1 mmol) was dissolved in mixed solvent DCM/HFIP (5:2, 2.0 mL), followed by the addition of *m*-CPBA (204 mg, 1.0 mmol, 85%) and phosphate buffer (66.0 μL) at room temperature. The reaction mixture was stirred until the complete consumption of **5c** (monitored by TLC analysis, about 24 h), then quenched by addition of saturated aqueous $Na_2S_2O_3$. The reaction mixture was extracted with ethyl acetate (5.0 mL × 3). The combined organic layer was washed with saturated aqueous $NaHCO_3$ and brine (5.0 mL × 2) sequentially. The organic phase dried over anhydrous Na_2SO_4 and concentrated in vacuum after filtration. The crude residue was directly used for the next step without further purification.

The above obtained crude ester was dissolved in 1.0 mL of mixed solvent THF/H_2O (9/1, v/v), then $NaBH_4$ (6.5 mg, 0.17 mmol) was added at room temperature in two portions. The resulting mixture was stirred until the completion of the starting material (monitored by TLC analysis, about 1.0 h). The reaction was quenched by the addition of 0.5 mL of saturated aqueous NH_4Cl solution, and the resulting mixture was stirred at room temperature until the generation of gas ceased. Then the reaction mixture was extracted with ethyl acetate (5.0 mL × 4), the combined organic layer was dried over anhydrous Na_2SO_4, and concentrated under vacuum after filtration. The crude mixture was purified by silica gel column chromatography (using DCM as eluent) to give product **16** as yellow oil in 63% yield with 91% *ee*. $[\alpha]_D^{20}$ = -34.3 (c = 0.033, MeOH); ^{1}H NMR (500 MHz, DMSO-*d6*) (δ, ppm) 10.56 (s, 1H), 7.37 (d, *J* = 7.4 Hz, 1H), 7.34 – 7.27 (m, 1H), 7.04 – 7.00 (m, 1H), 6.88 (s, 1H), 6.84 (d, *J* = 7.7 Hz, 1H), 5.20 (s, 1H), 4.17 – 4.04 (m, 1H), 4.01 – 3.86 (m, 1H); ^{13}C NMR (125 MHz, DMSO-d_6) (δ, ppm) 142.8, 130.3, 126.9, 126.5, 121.9, 121.3 (t, *J* = 253.6 Hz), 109.9,

76.8 (t, J = 24.8 Hz), 66.4, 58.4 (t, J = 22.7 Hz); ^{19}F NMR (376 MHz, DMSO-d_6) (δ, ppm) -112.9 (d, J = 249.6 Hz, 1F), -114.2 (d, J = 249.6 Hz, 1F) HRMS (TOF MS ESI$^+$) calculated for $C_{10}H_{10}F_2NO_2$ [M+H]$^+$: 214.0680, found 214.0677; HPLC conditions for determination of enantiomeric excess: Chiralpak OD-H, λ = 254 nm, hexane : 2-propanol = 80:20, flow rate = 1.0 mL/min, t_{minor} = 9.2 min, t_{major} = 10.8 min.

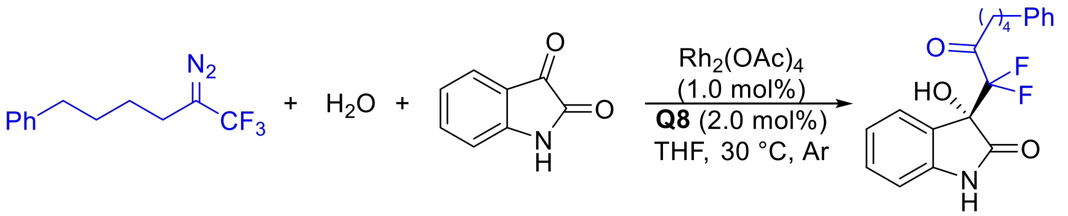

1j **3f** **17**, 74%, 90% *ee*

Synthesis of 17: To a 10-mL oven-dried vial containing a magnetic stirring bar, isatin **3f** (14.7 mg, 0.1 mmol), H_2O (0.15 mmol, 2.7 μL, 1.5 equiv.), $Rh_2(OAc)_4$ (0.45 mg, 1.0 mol%), and organocatalyst **Q8** (1.2 mg, 2.0 mol%) in tetrahydrofuran (THF, 1.0 mL), was added a solution of diazo compound **1j** (36.3 mg, 0.15 mmol, 1.5 equiv.) in 1.0 mL THF *via* syringe pump over 1 h under argon atmosphere at 30 °C. After addition, the reaction mixture was stirred for additional 1~2 h under these conditions until consumption of the material (monitored by TLC). Then the reaction mixture was purified by column chromatography on silica gel without any additional treatment (Hexanes : EtOAc = 5:1 to 2:1) to give 26.6 mg of pure product **17** as yellow oil in 74% yield with 90% *ee*. ^{1}H NMR (400 MHz, $CDCl_3$) (δ, ppm) 8.37 (s, 1H), 7.47 (d, J = 7.5 Hz, 1H), 7.35 – 7.23 (m, 3H), 7.21 – 7.11 (m, 3H), 7.11 – 7.05 (m, 1H), 6.86 (d, J = 7.8 Hz, 1H), 4.70 (s, 1H), 2.78 – 2.61 (m, 2H), 2.57 (t, J = 7.2 Hz, 2H), 1.66 – 1.50 (m, 4H); ^{13}C NMR (100 MHz, $CDCl_3$) (δ, ppm) 201.4 (dd, J = 32.9, 25.4 Hz), 174.7, 142.0, 141.7, 131.6, 128.50, 128.46, 126.7, 126.0, 124.0, 123.6, 113.3 (dd, J = 264.6, 262.2 Hz), 110.9, 77.2 (dd, J = 27.9, 3.8 Hz), 38.0, 35.7, 30.5, 22.0; ^{19}F NMR (376 MHz, $CDCl_3$) (δ, ppm) -115.7 (d, J = 278.5 Hz, 1F), -119.2 (d, J = 278.5 Hz, 1F); HRMS (TOF MS ESI+) calculated for $C_{20}H_{20}F_2NO_3$ [M + H]$^+$: 360.1411, found 360.1409; HPLC conditions for determination of enantiomeric excess: Chiralpak OD-

H, λ = 254 nm, hexane : propanol = 80:20, flow rate =1.0 mL/min, t_{major} = 7.1 min, t_{minor} = 8.5 min.

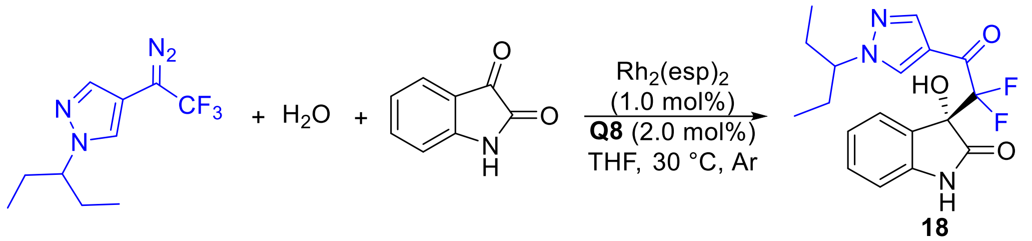

1k **2a** **3f** **18**

80% yield, 90% *ee*
(after recrystallization)

Synthesis of 18: To a 10-mL oven-dried vial containing a magnetic stirring bar, isatin **3f** (0.1 mmol, 14.7 mg), H_2O (0.15 mmol, 2.7 μL, 1.5 equiv.), $Rh_2(esp)_2$ (0.76 mg, 1.0 mol%), and organocatalyst **Q8** (1.2 mg, 2.0 mol%) in tetrahydrofuran (THF, 1.0 mL), was added a solution of diazo compound **1k** (0.15 mmol, 36.9 mg 1.5 equiv.) in 1.0 mL THF *via* syringe pump over 1 h under argon atmosphere at 30 °C. After addition, the reaction mixture was stirred for additional 1~2 h under these conditions until consumption of the material (monitored by TLC). Then the reaction mixture was purified by column chromatography on silica gel without any additional treatment (Hexanes : EtOAc = 5:1 to 2:1) to give 29.1 mg pure product **18** as white solid in 80% yield with 90% *ee* after separation by crystallization out in DCM. ^1H NMR (400 MHz, $CDCl_3$) (δ, ppm) 8.60 (s, 1H), 8.17 – 7.82 (m, 2H), 7.46 (d, J = 7.4 Hz, 1H), 7.35 – 7.21 (m, 1H), 7.07 – 6.96 (m, 1H), 6.87 (d, J = 7.8 Hz, 1H), 5.04 (s, 1H), 4.19 – 3.46 (m, 1H), 2.05 – 1.69 (m, 4H), 0.73 (t, J = 7.3 Hz, 6H); ^{13}C NMR (100 MHz, $CDCl_3$) (δ, ppm) 183.1 (dd, J = 31.8, 28.5 Hz), 174.9, 142.2, 141.9, 134.2, 131.4, 126.7, 124.6, 123.3 (d, J = 7.8 Hz), 117.7 (d, J = 2.3 Hz), 115.0 (t, J = 262.8 Hz), 110.9 (d, J = 9.7 Hz), 67.7 (d, J = 13.4 Hz), 28.0, 10.61, 10.56; ^{19}F NMR (376 MHz, $CDCl_3$) (δ, ppm) -112.2 (d, J = 281.4 Hz, 1F), -114.5 (d, J = 278.5 Hz, 1F); HRMS (TOF MS ESI+) calculated for $C_{18}H_{20}F_2N_3O_3$ $[M + H]^+$: 364.1473, found 364.1471; HPLC conditions for determination of enantiomeric excess: Chiralpak IA, λ = 254 nm, hexane : propanol = 70:30, flow rate =1.0 mL/min, t_{major} = 6.7 min, t_{minor} = 10.1 min.

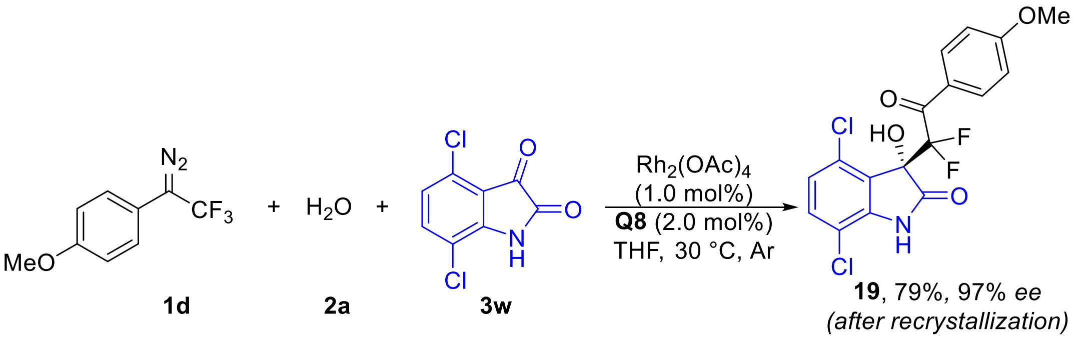

<u>Synthesis of 19</u>: To a 10-mL oven-dried vial containing a magnetic stirring bar, isatin **3w** (0.1 mmol, 21.4 mg), H_2O (0.15 mmol, 2.7 μL, 1.5 equiv.), $Rh_2(OAc)_4$ (0.45 mg, 1.0 mol%), and organocatalyst **Q8** (1.2 mg, 2.0 mol%) in tetrahydrofuran (THF, 1.0 mL), was added a solution of diazo compound **1d** (0.15 mmol, 32.4 mg 1.5 equiv.) in 1.0 mL THF *via* syringe pump over 1 h under argon atmosphere at 30 °C. After addition, the reaction mixture was stirred for additional 1~2 h under these conditions until consumption of the material (monitored by TLC). Then the reaction mixture was purified by column chromatography on silica gel without any additional treatment (Hexanes : EtOAc = 5:1 to 2:1) to give pure product **18** as white solid in 90% yield with 85% *ee* (31.8 mg, 79% yield, 97% *ee* after recrystallization in DCM and *n*-hexane). ^{1}H NMR (400 MHz, $CDCl_3$) (δ, ppm) 8.20 (s, 1H), 8.10 – 8.02 (m, 2H), 7.29 – 7.21 (m, 1H), 7.05 – 6.97 (m, 1H), 6.95 – 6.87 (m, 2H), 4.93 (s, 1H), 3.87 (s, 3H); ^{13}C NMR (100 MHz, $CDCl_3$) (δ, ppm) 187.2 (t, J = 28.9 Hz), 173.0 (t, J = 3.7 Hz), 165.1, 141.3, 133.3 (t, J = 2.6 Hz), 131.9, 131.8, 126.0, 125.3, 122.8, 115.5 (dd, J = 269.6, 266.4 Hz), 114.4, 114.2, 80.3 (dd, J = 27.7, 25.1 Hz), 55.8; ^{19}F NMR (376 MHz, $CDCl_3$) (δ, ppm) -104.0 (d, J = 287.0 Hz, 1F), -105.4 (d, J = 287.0 Hz, 1F); HRMS (TOF MS ESI+) calculated for $C_{17}H_{12}Cl_2F_2NO_4$ $[M + H]^+$: 402.0111, found 402.0109; HPLC conditions for determination of enantiomeric excess: Chiralpak IA, λ = 254 nm, hexane : propanol = 80:20, flow rate =1.0 mL/min, t_{major} = 13.5 min, t_{minor} = 21.4 min.

6. NMR Spectra of New Compounds

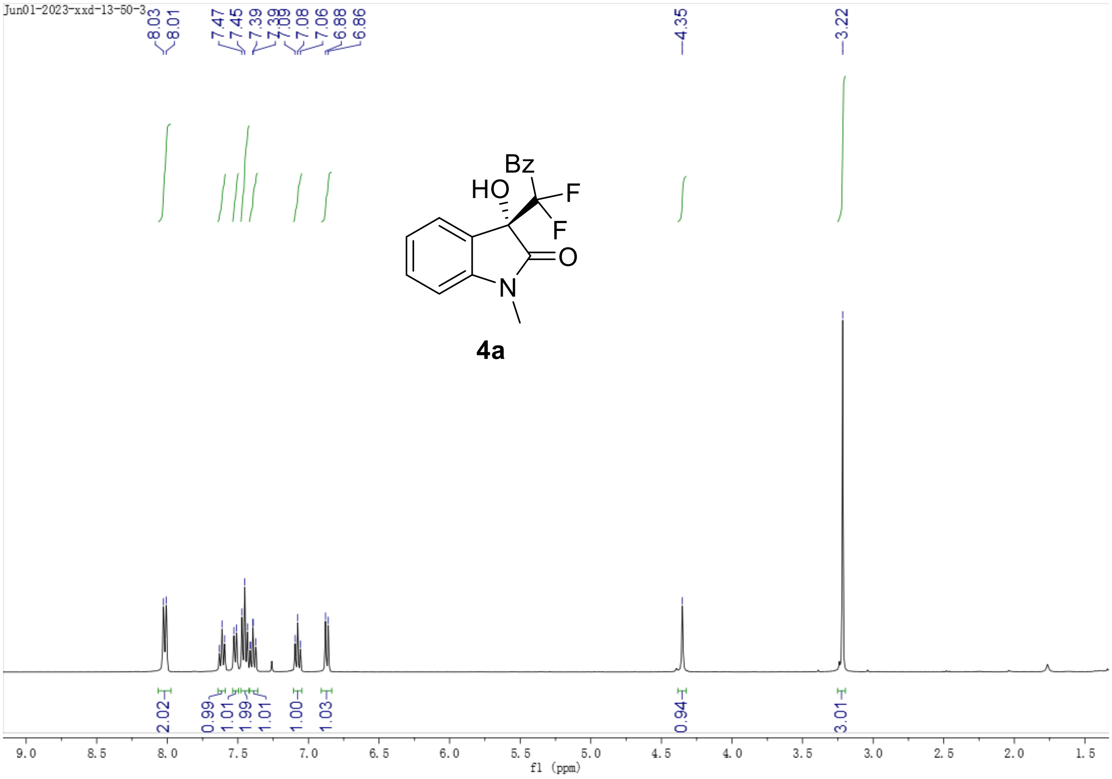

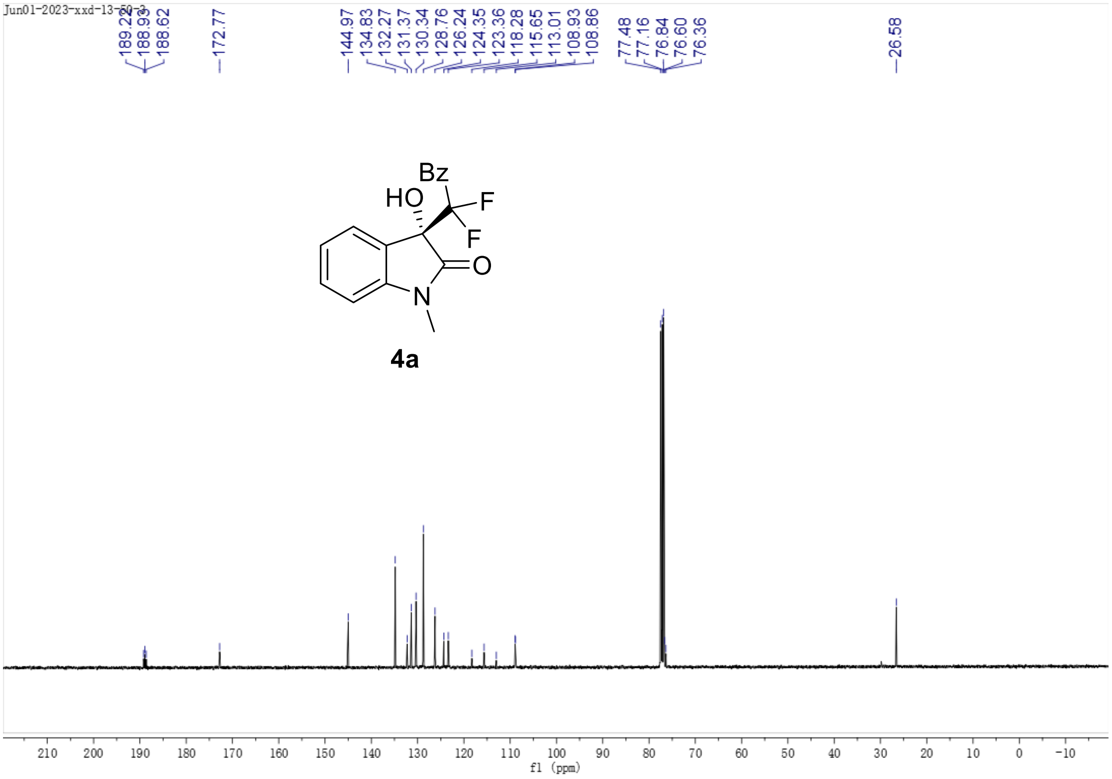

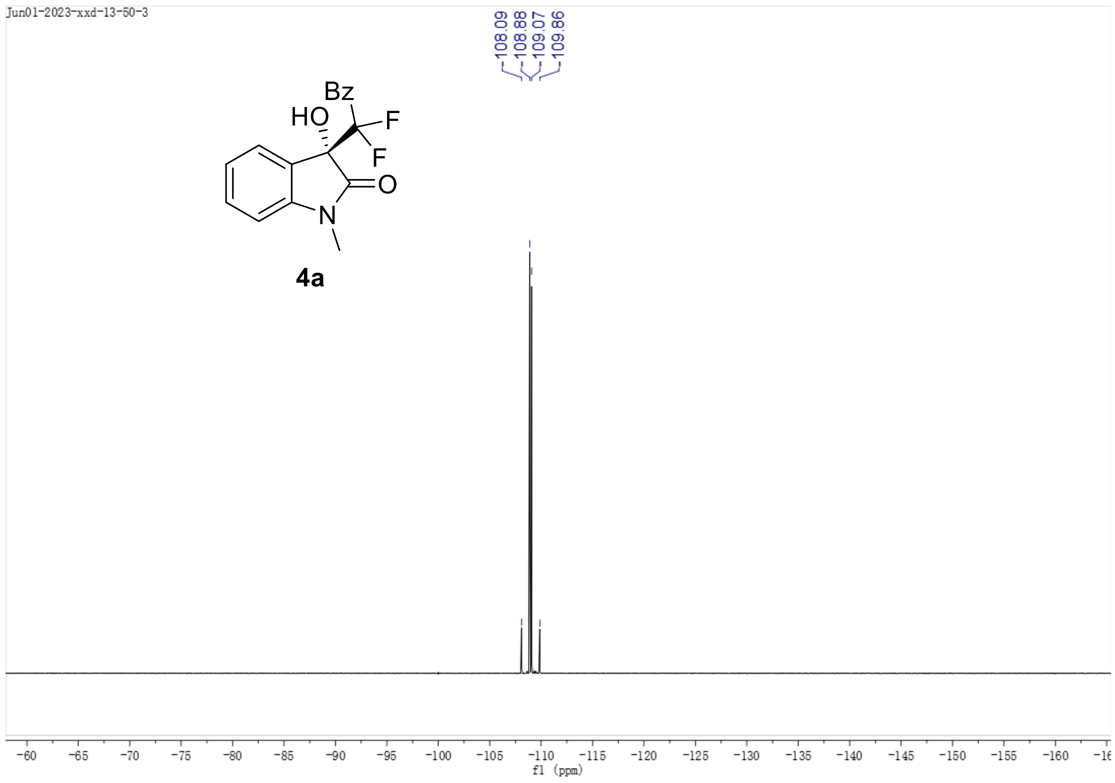
Jun01-2023-xxd-13-50-3
108.09
108.88
109.07
109.86
Bz
HO
F
F
O
N
4a
f1 (ppm)

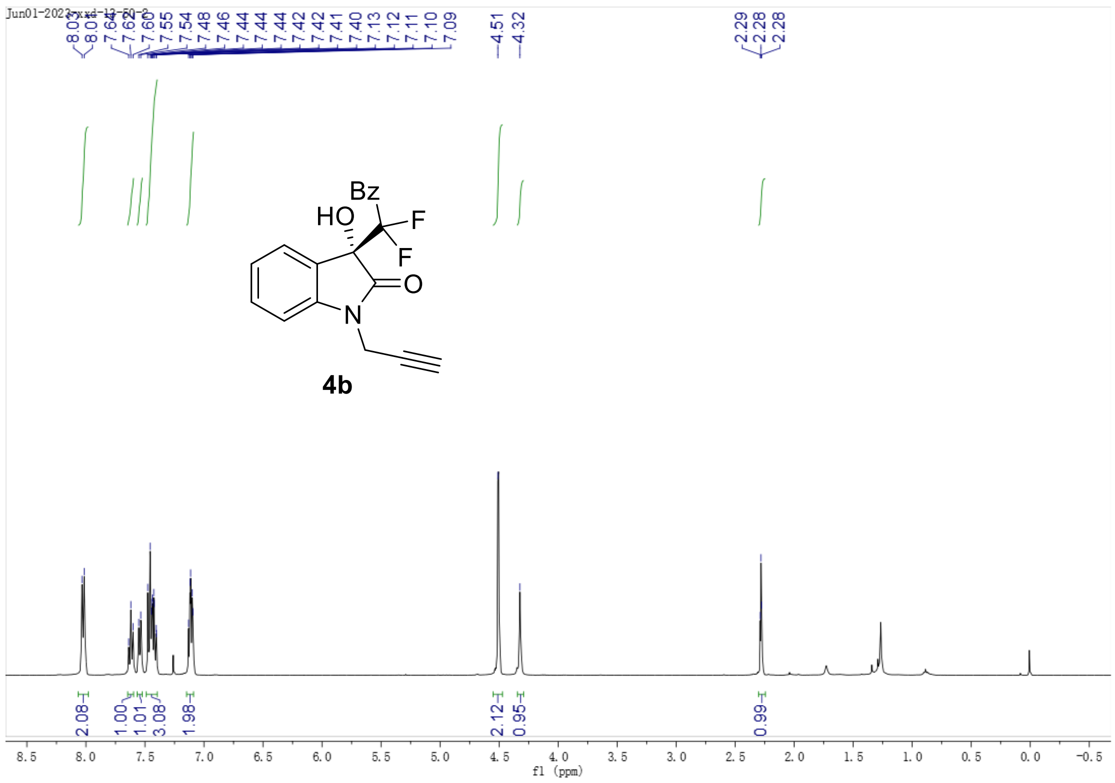
Jun01-2023-xxd-13-50-2
4.51
4.32
2.29
2.28
2.28
Bz
HO
F
F
O
N
4b
2.08
1.00
1.01
3.08
1.98
2.12
0.95
0.99
f1 (ppm)

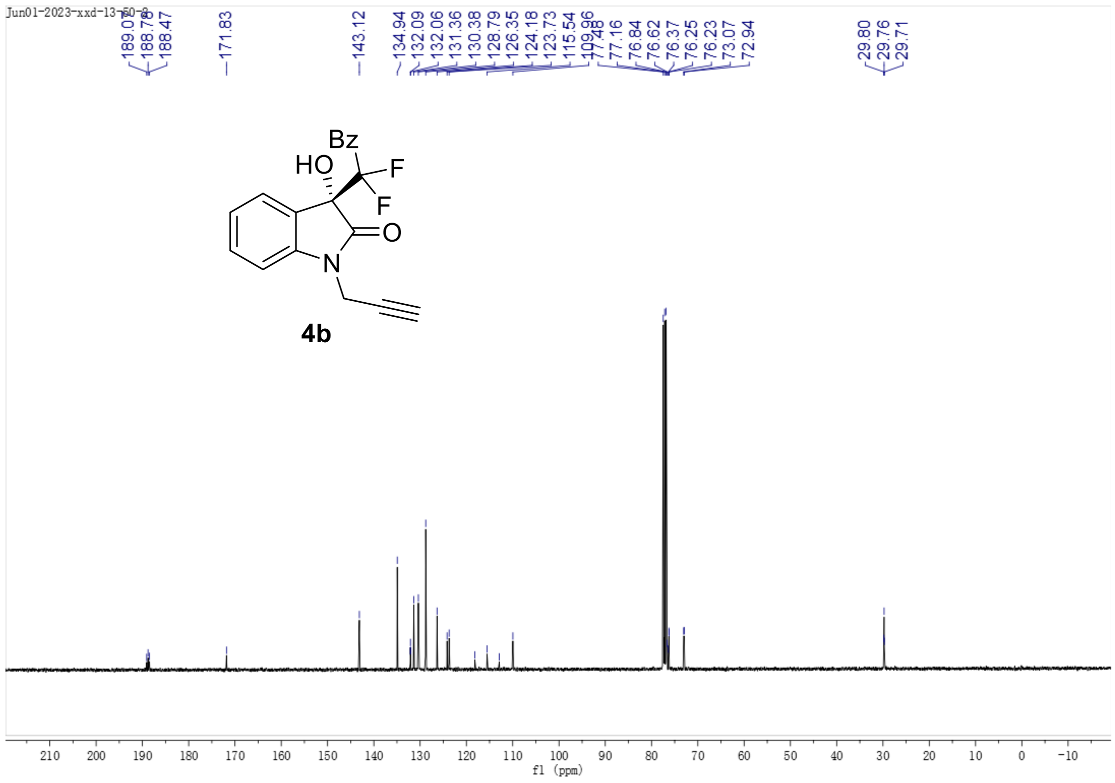
Jun01-2023-xxd-13-50-3
189.0?
188.76
188.47
171.83
143.12
134.94
132.09
132.06
131.36
130.38
128.79
126.35
124.18
123.73
115.54
109.96
77.48
77.16
76.84
76.62
76.37
76.25
76.23
73.07
72.94
29.80
29.76
29.71
Bz
HO
F
F
O
N
4b
f1 (ppm)

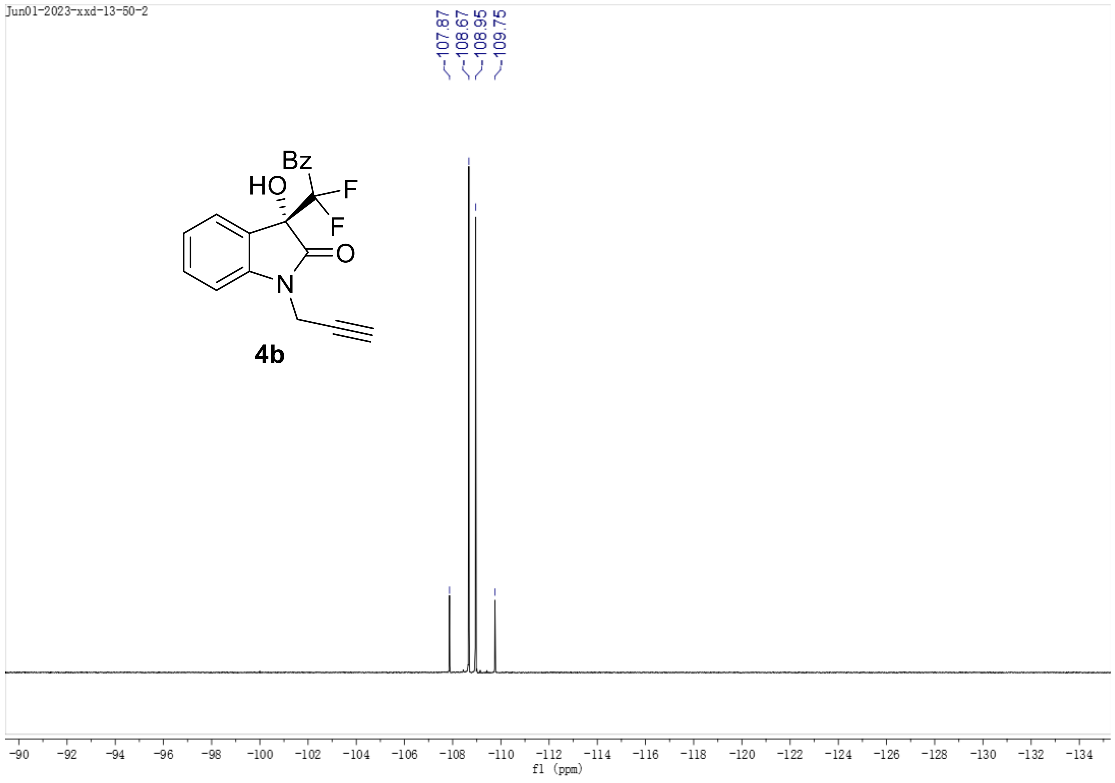
Jun01-2023-xxd-13-50-2
-107.87
-108.67
-108.95
-109.75
Bz
HO
F
F
O
N
4b
f1 (ppm)

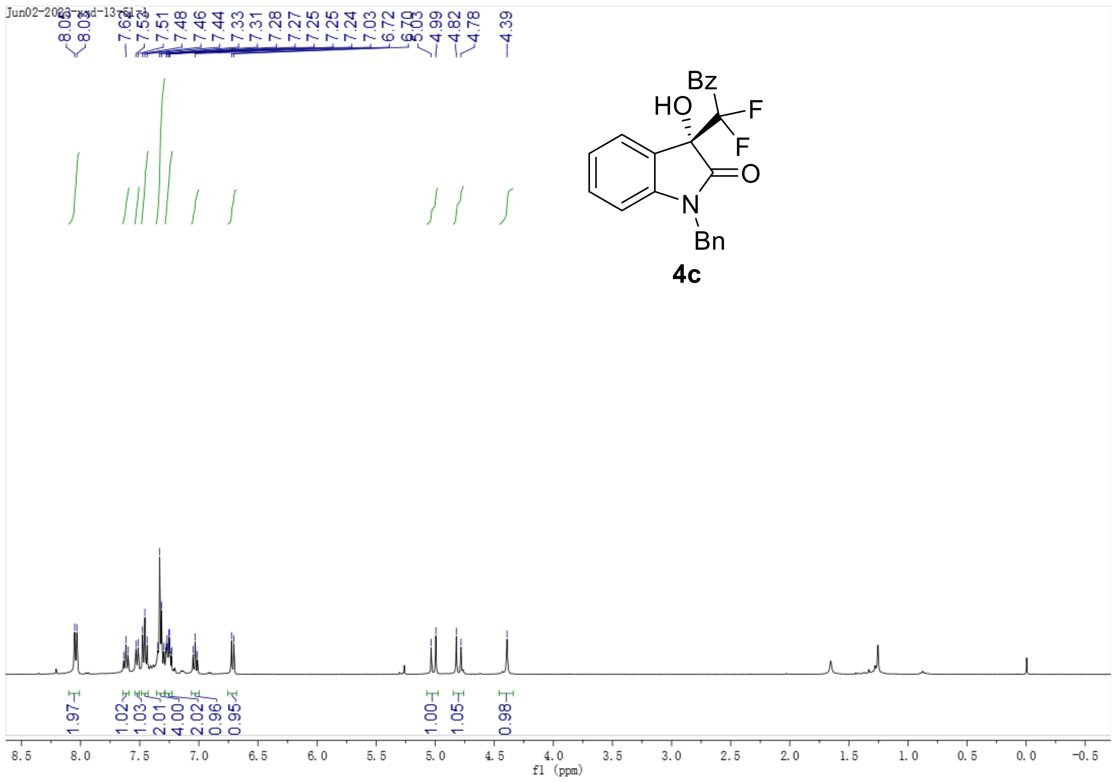

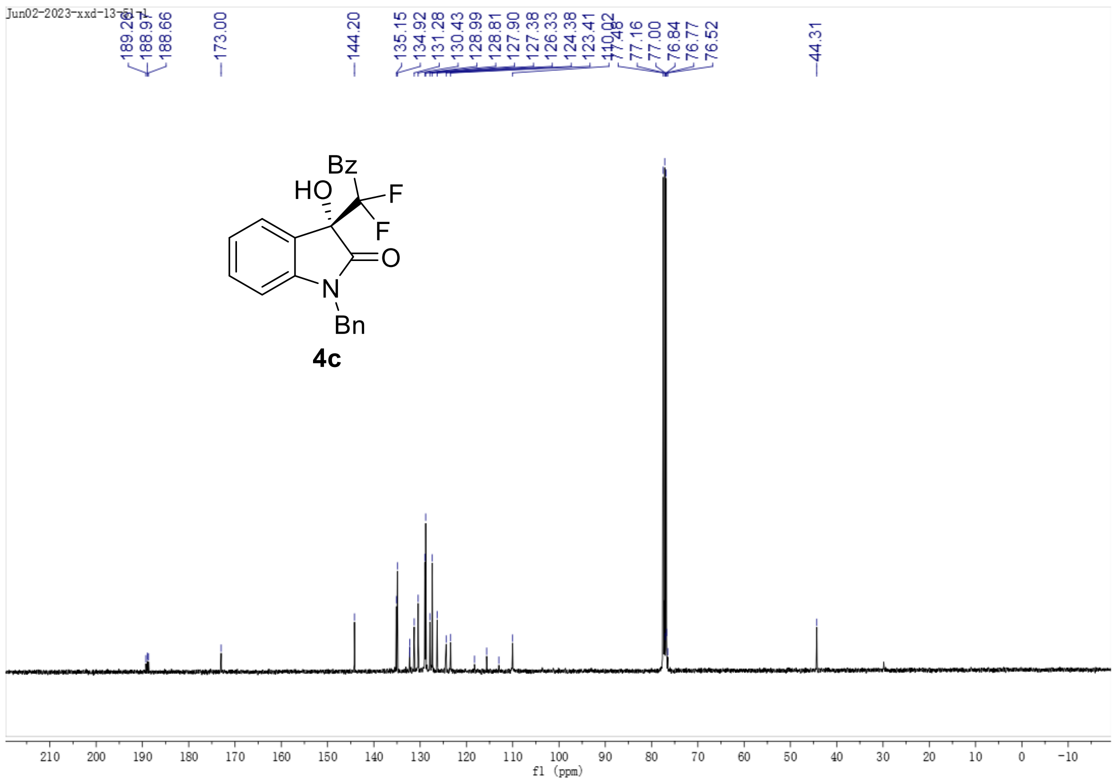

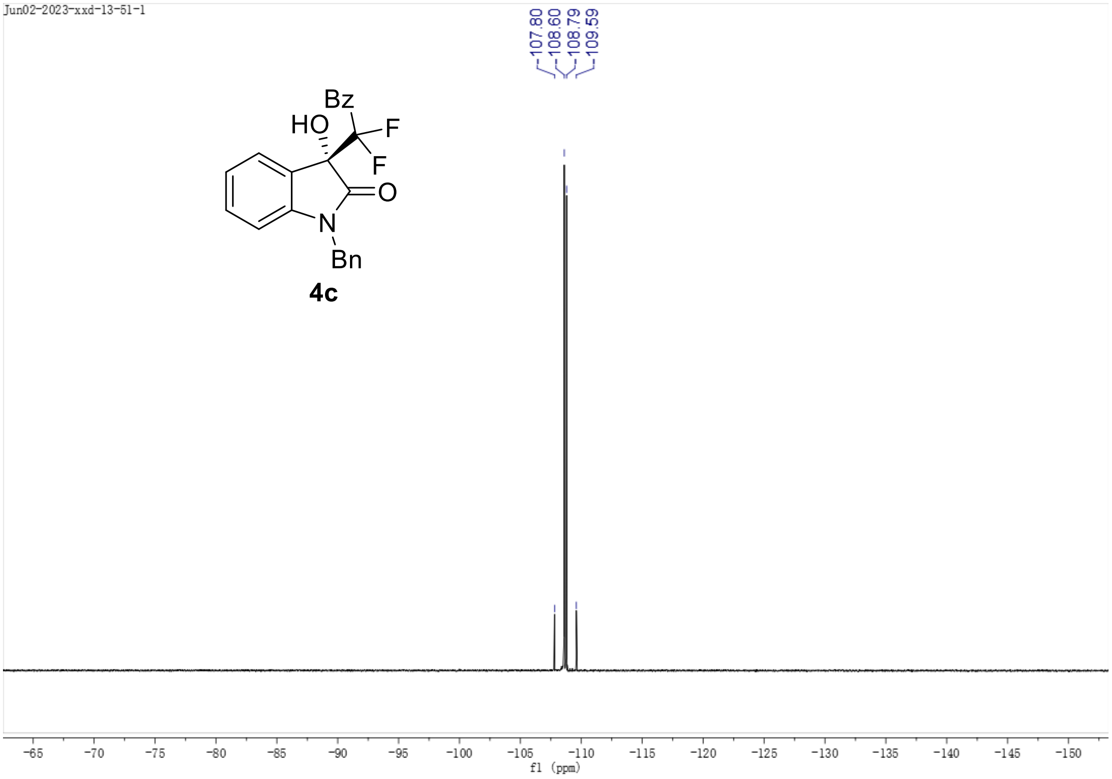
Jun02-2023-xxd-13-51-1
-107.80
-108.60
-108.79
-109.59
Bz
HO
F
F
O
N
Bn
4c
-65 -70 -75 -80 -85 -90 -95 -100 -105 -110 -115 -120 -125 -130 -135 -140 -145 -150
f1 (ppm)

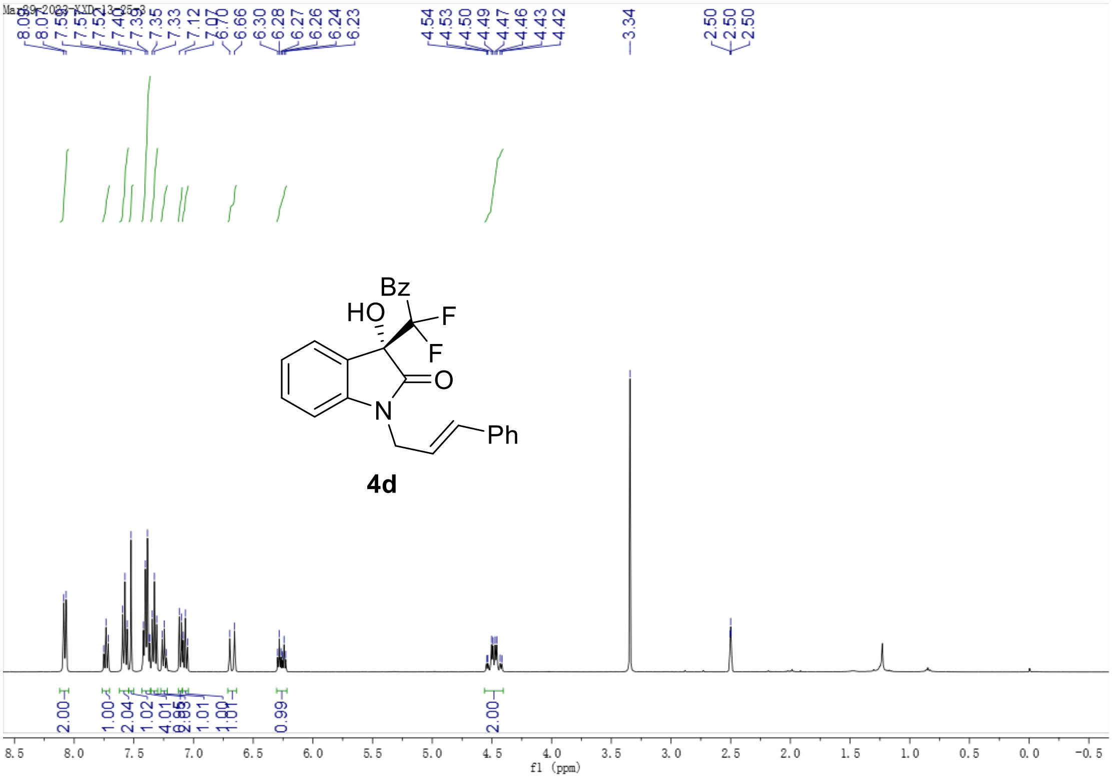
Bz
HO
F
F
O
N
Ph
4d
4.54
4.53
4.50
4.49
4.47
4.46
4.43
4.42
-3.34
2.50
2.50
2.50
2.00
1.00
2.04
1.02
4.01
0.99
2.00
8.5 8.0 7.5 7.0 6.5 6.0 5.5 5.0 4.5 4.0 3.5 3.0 2.5 2.0 1.5 1.0 0.5 0.0 -0.5
f1 (ppm)

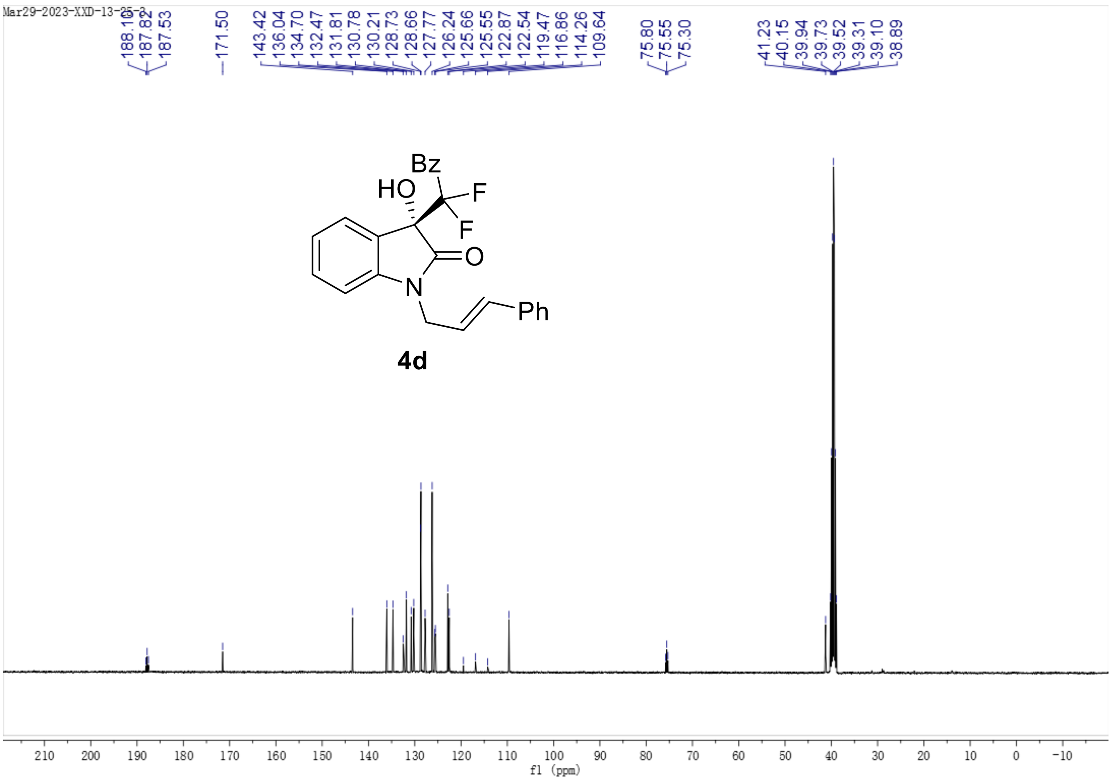
Mar29-2023-XXD-13-25-2
188.10
187.82
187.53
171.50
143.42
136.04
134.70
132.47
131.81
130.78
130.21
128.73
128.66
127.77
126.24
125.66
125.55
122.87
122.54
119.47
116.86
114.26
109.64
75.80
75.55
75.30
41.23
40.15
39.94
39.73
39.52
39.31
39.10
38.89
Bz
HO
F
F
O
N
Ph
4d
f1 (ppm)

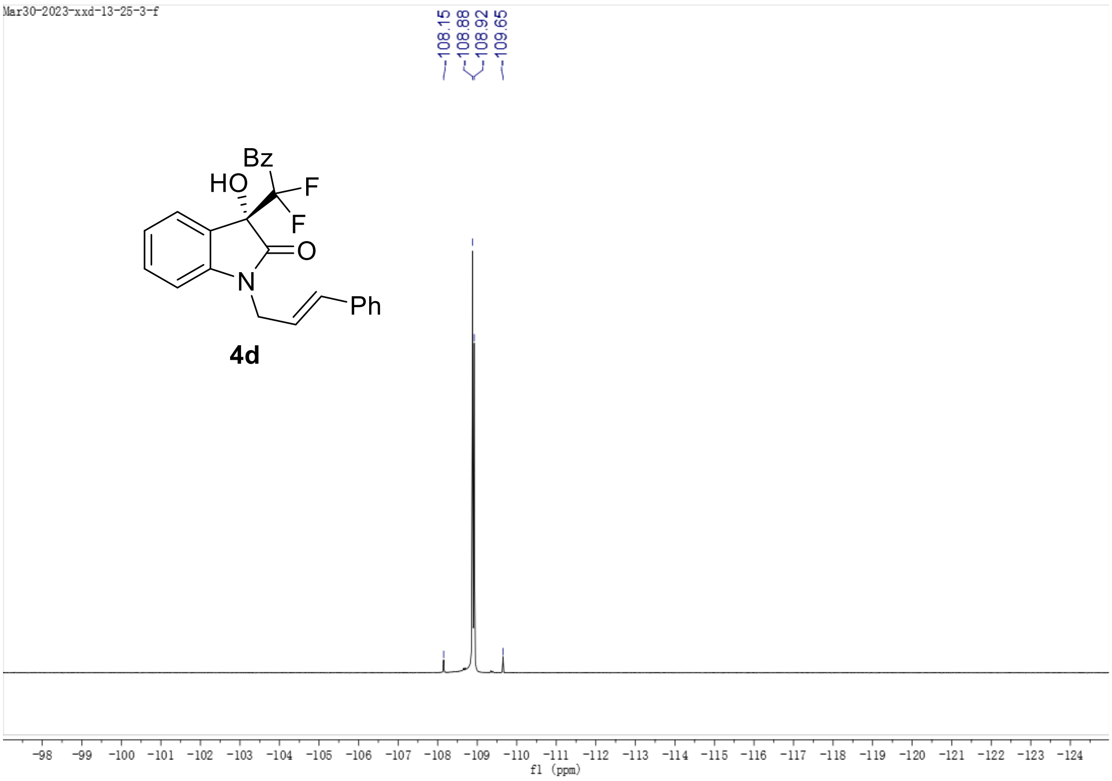
Mar30-2023-xxd-13-25-3-f
-108.15
-108.88
-108.92
-109.65
Bz
HO
F
F
O
N
Ph
4d
f1 (ppm)

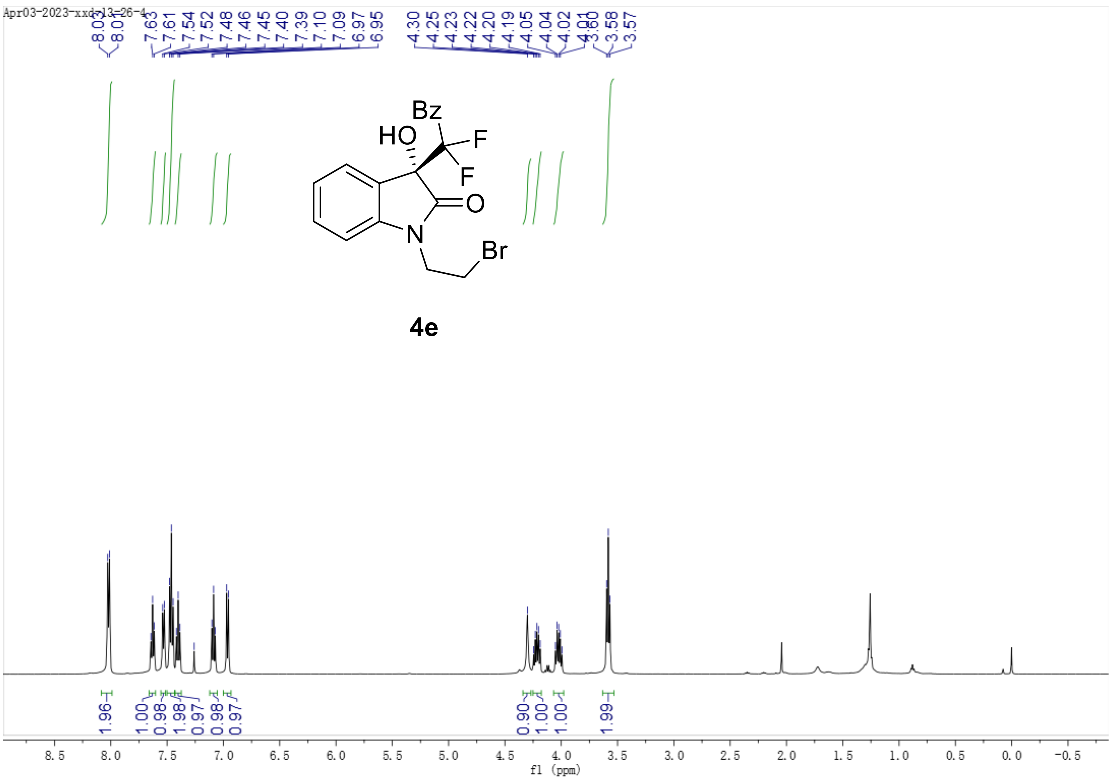
Apr03-2023-xxd-13-26-4
8.03
8.01
7.63
7.61
7.54
7.52
7.48
7.46
7.45
7.40
7.39
7.10
7.09
6.97
6.95
4.30
4.25
4.23
4.22
4.20
4.19
4.05
4.04
4.02
4.01
3.60
3.58
3.57
Bz
HO
F
F
O
N
Br
4e
1.96
1.00
0.98
1.98
0.97
0.98
0.97
0.90
1.00
1.00
1.99
f1 (ppm)

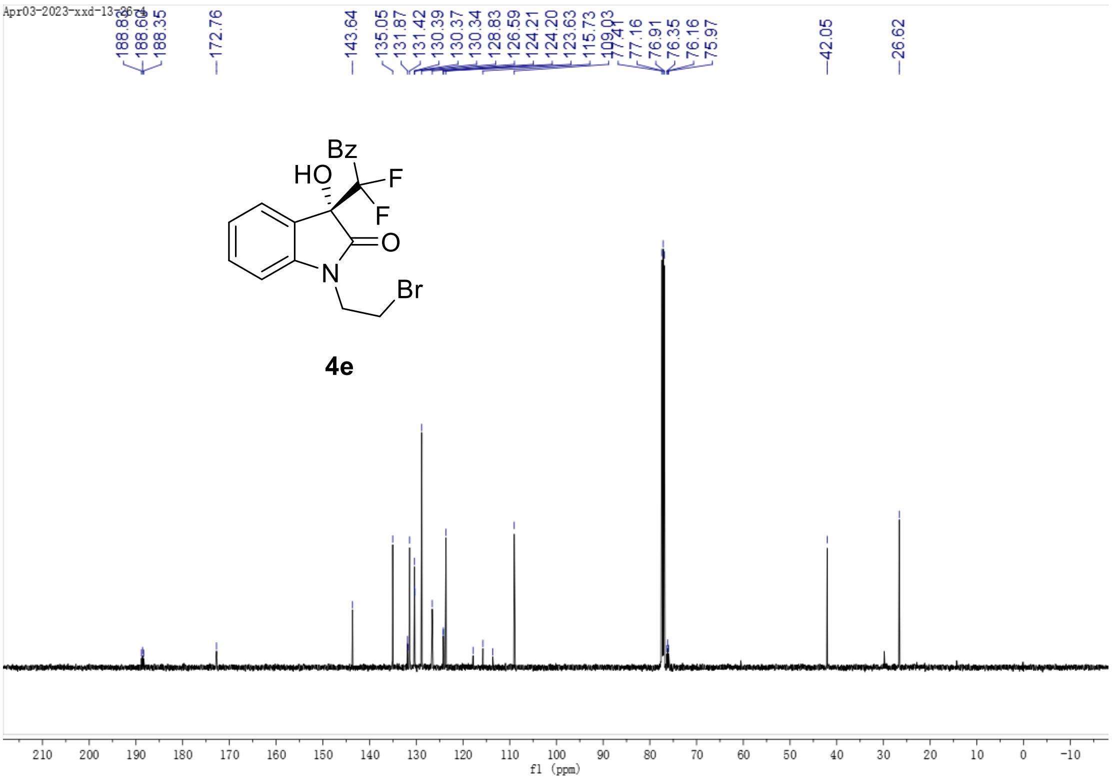
Apr03-2023-xxd-13-26-4
188.88
188.62
188.35
172.76
143.64
135.05
131.87
131.42
130.39
130.37
130.34
128.83
126.59
124.21
124.20
123.63
115.73
109.03
77.41
77.16
76.91
76.35
76.16
75.97
42.05
26.62
Bz
HO
F
F
O
N
Br
4e
f1 (ppm)

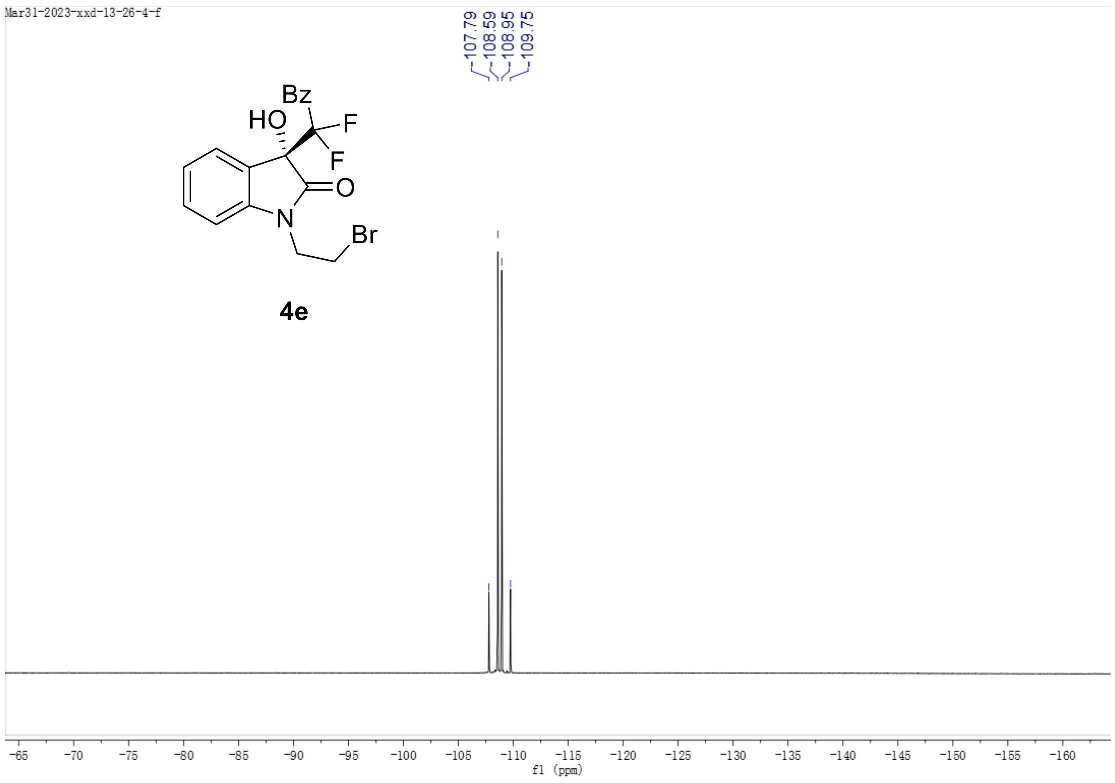
Mar31-2023-xxd-13-26-4-f
-107.79
-108.59
-108.95
-109.75
Bz
HO
F
F
O
N
Br
4e
-65 -70 -75 -80 -85 -90 -95 -100 -105 -110 -115 -120 -125 -130 -135 -140 -145 -150 -155 -160
f1 (ppm)

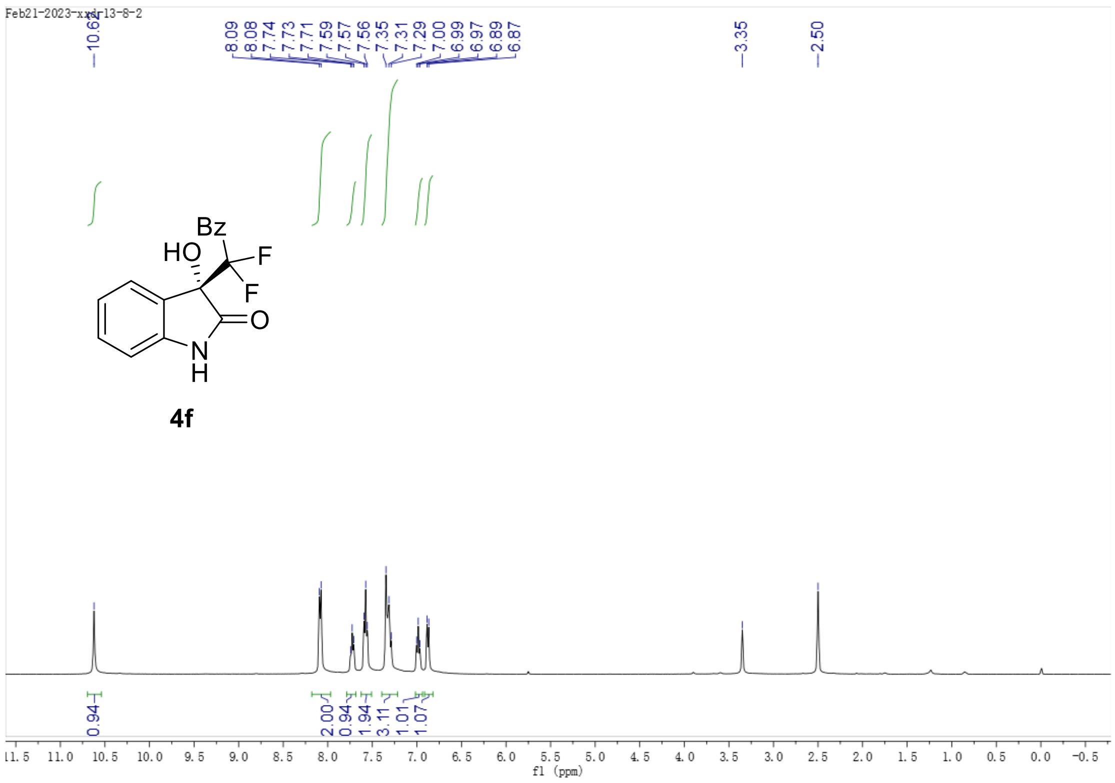
Feb21-2023-xxd-13-8-2
-10.63
8.09
8.08
7.74
7.73
7.71
7.59
7.57
7.56
7.35
7.31
7.29
7.00
6.99
6.97
6.89
6.87
-3.35
-2.50
Bz
HO
F
F
O
N
H
4f
0.94
2.00
0.94
1.94
3.11
1.01
1.07
11.5 11.0 10.5 10.0 9.5 9.0 8.5 8.0 7.5 7.0 6.5 6.0 5.5 5.0 4.5 4.0 3.5 3.0 2.5 2.0 1.5 1.0 0.5 0.0 -0.5
f1 (ppm)

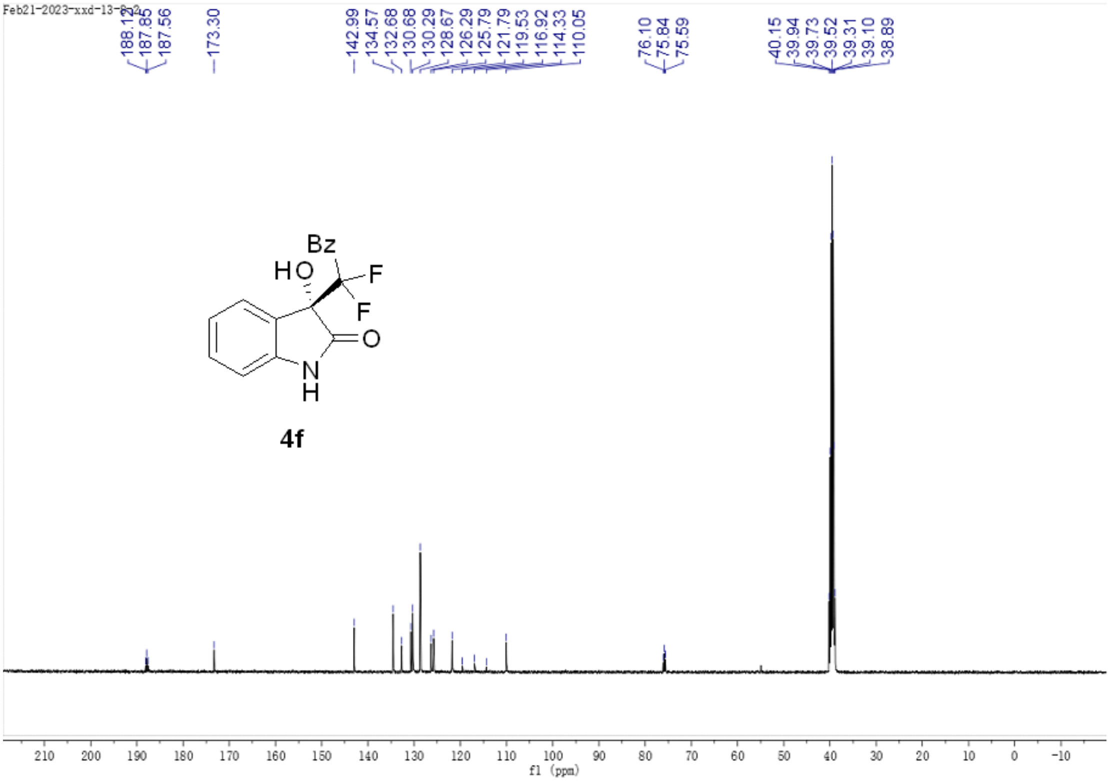
Feb21-2023-xxd-13-8-2
188.12
187.85
187.56
173.30
142.99
134.57
132.68
130.68
130.29
128.67
126.29
125.79
121.79
119.53
116.92
114.33
110.05
76.10
75.84
75.59
40.15
39.94
39.73
39.52
39.31
39.10
38.89
Bz
HO
F
F
O
N
H
4f
fl (ppm)

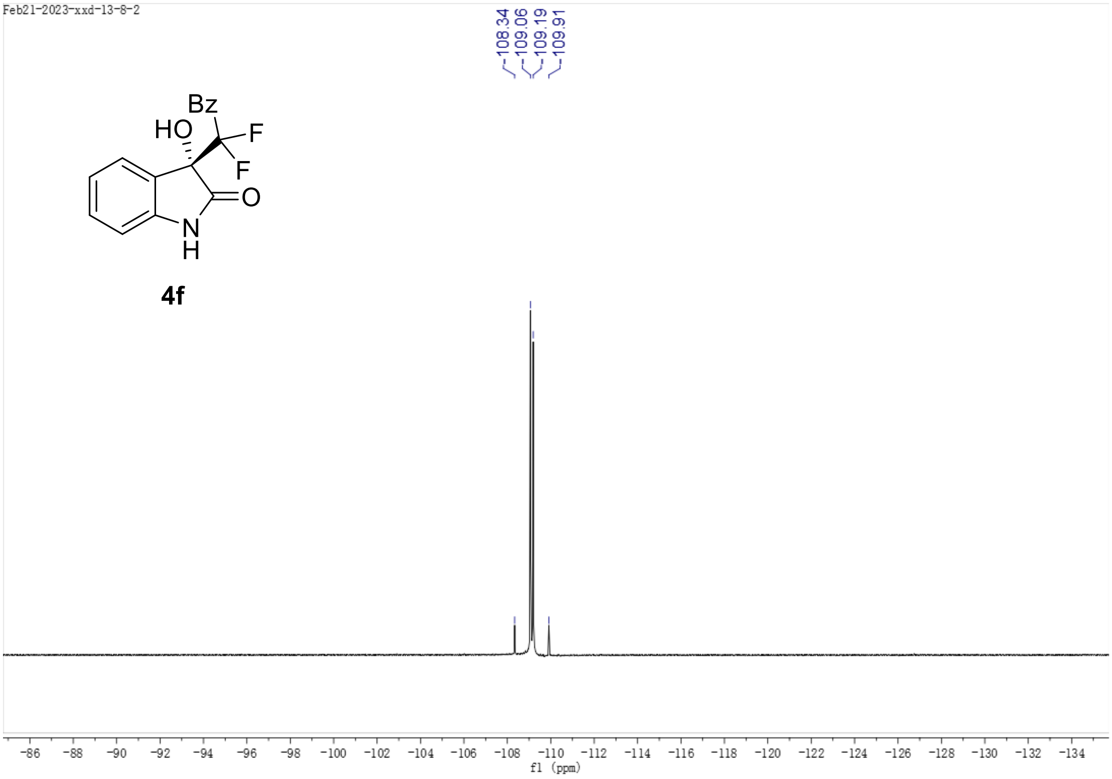
Feb21-2023-xxd-13-8-2
-108.34
-109.06
-109.19
-109.91
Bz
HO
F
F
O
N
H
4f
fl (ppm)

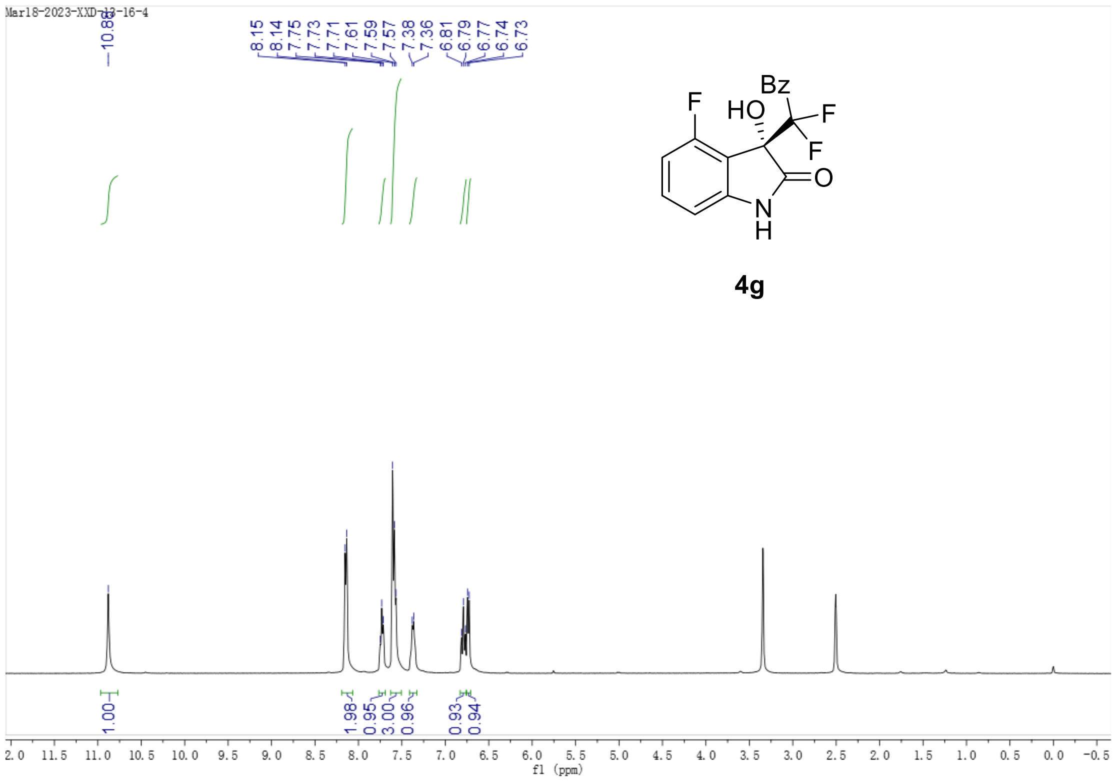

Mar18-2023-XXD-
10.88
8.15
8.14
7.75
7.73
7.71
7.61
7.59
7.57
7.38
7.36
6.81
6.79
6.77
6.74
6.73
F
HO
Bz
F
F
O
N
H
4g
1.00
1.98
0.95
3.00
0.96
0.93
0.94
f1 (ppm)

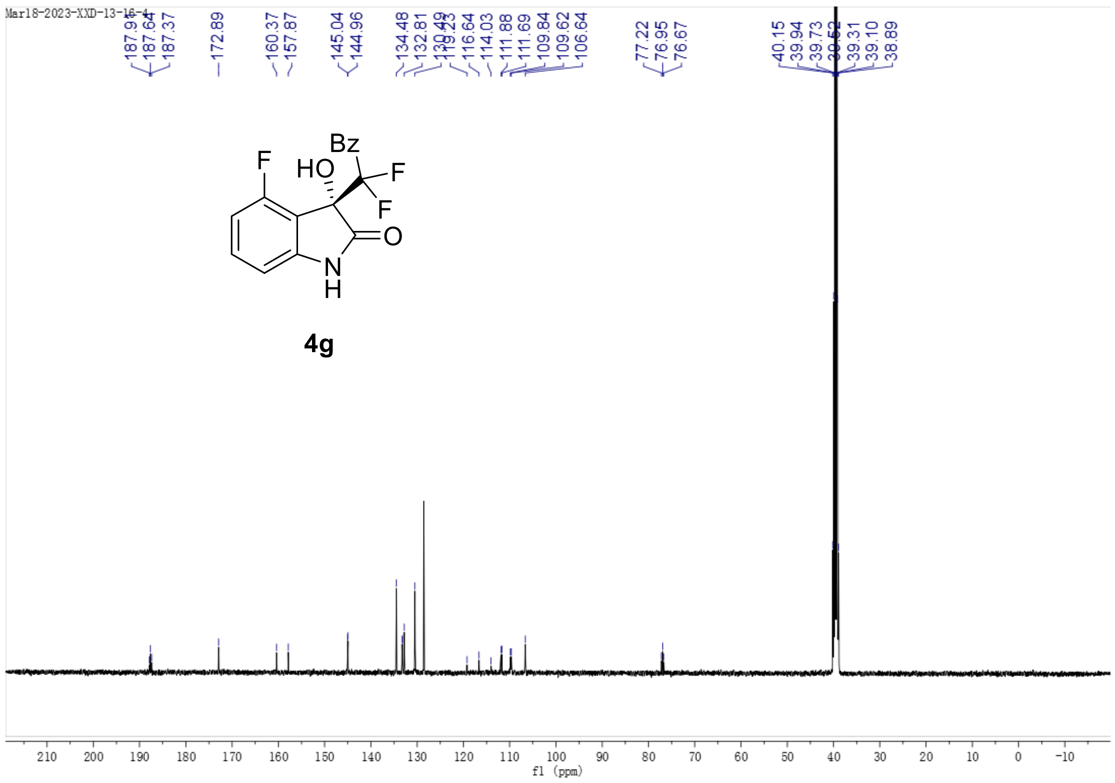

Mar18-2023-XXD-13-16-4
187.91
187.64
187.37
172.89
160.37
157.87
145.04
144.96
134.48
132.81
116.64
114.03
111.88
111.69
109.84
109.62
106.64
77.22
76.95
76.67
40.15
39.94
39.73
39.31
39.10
38.89
F
HO
Bz
F
F
O
N
H
4g
f1 (ppm)

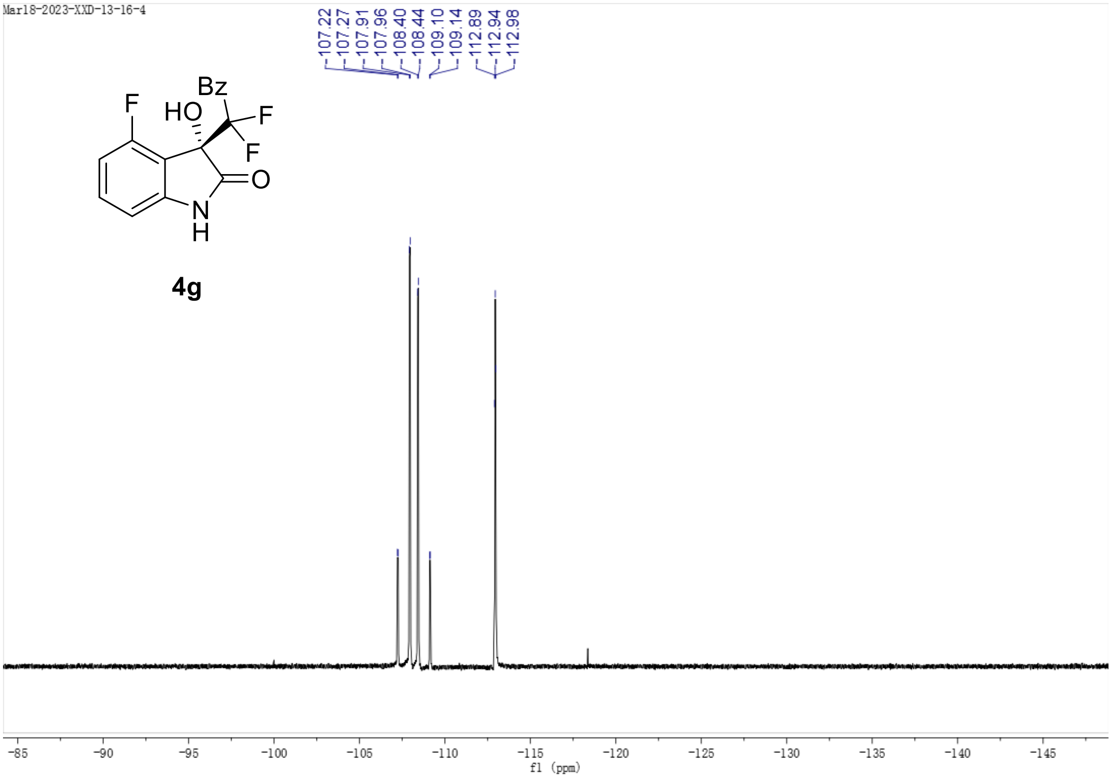
Mar18-2023-XXD-13-16-4
107.22
107.27
107.91
107.96
108.40
108.44
109.10
109.14
112.89
112.94
112.98
F
HO
Bz
F
F
O
N
H
4g
-85
-90
-95
-100
-105
-110
-115
-120
-125
-130
-135
-140
-145
f1 (ppm)

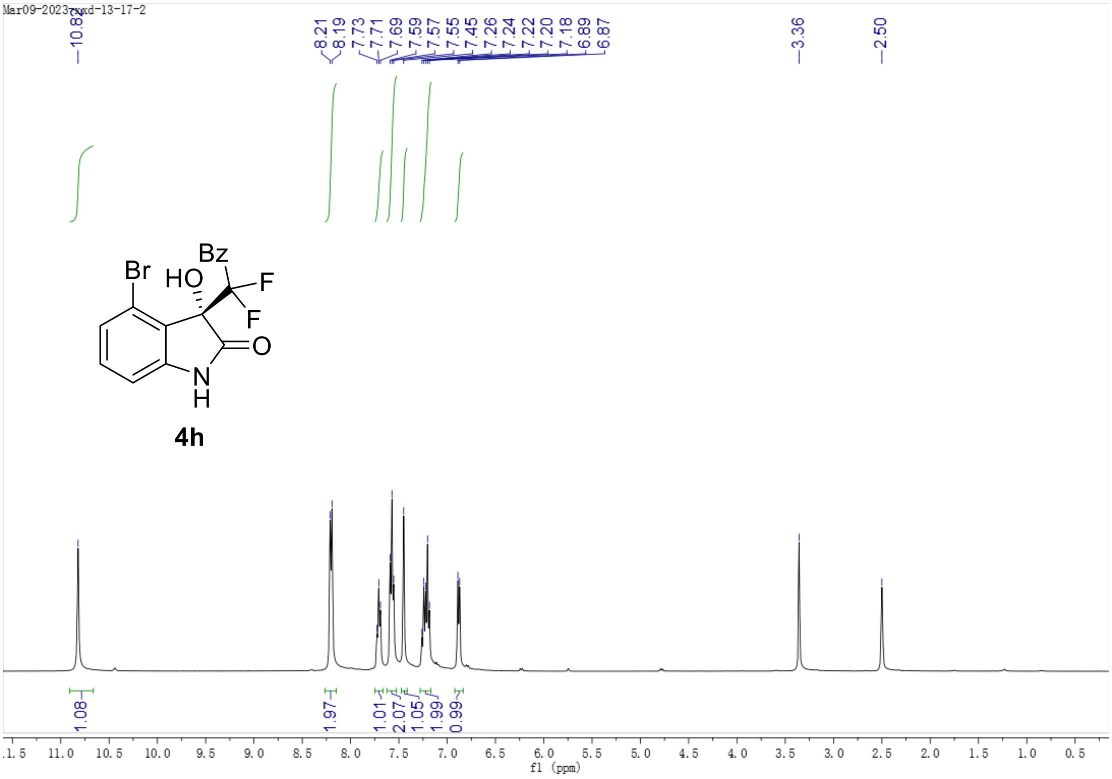
Mar09-2023-xxd-13-17-2
10.82
8.21
8.19
7.73
7.71
7.69
7.59
7.57
7.55
7.45
7.26
7.24
7.22
7.20
7.18
6.89
6.87
3.36
2.50
Br
HO
Bz
F
F
O
N
H
4h
1.08
1.97
1.01
2.07
1.05
1.99
0.99
11.0
10.5
10.0
9.5
9.0
8.5
8.0
7.5
7.0
6.5
6.0
5.5
5.0
4.5
4.0
3.5
3.0
2.5
2.0
1.5
1.0
0.5
f1 (ppm)

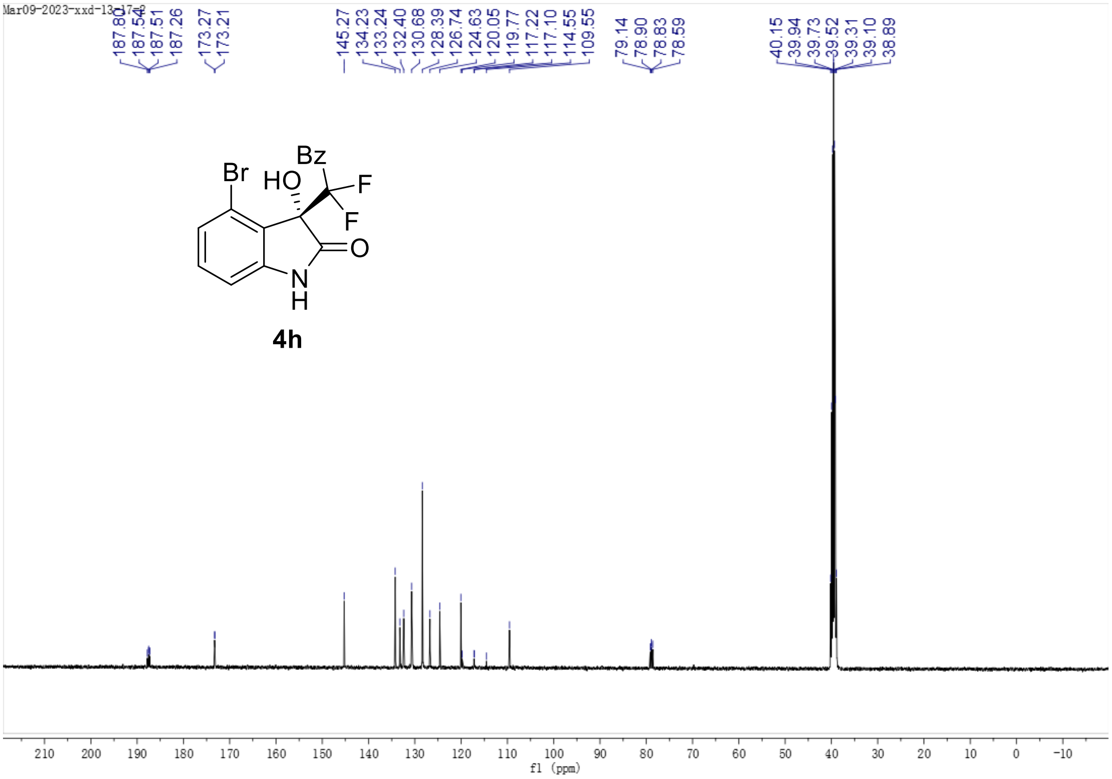
Mar09-2023-xxd-13-17-2
187.80
187.54
187.51
187.26
173.27
173.21
145.27
134.23
133.24
132.40
130.68
128.39
126.74
124.63
120.05
119.77
117.22
117.10
114.55
109.55
79.14
78.90
78.83
78.59
40.15
39.94
39.73
39.52
39.31
39.10
38.89
Br
HO
Bz
F
F
O
N
H
4h
f1 (ppm)

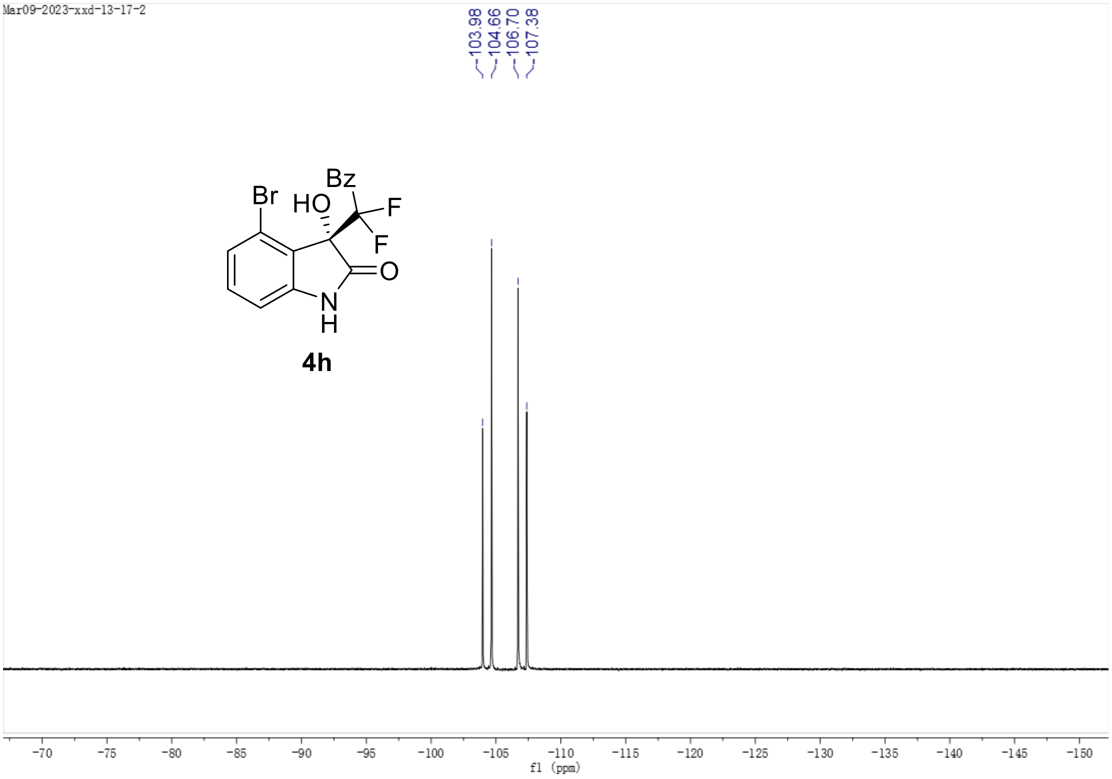
Mar09-2023-xxd-13-17-2
-103.98
-104.66
-106.70
-107.38
Br
HO
Bz
F
F
O
N
H
4h
f1 (ppm)

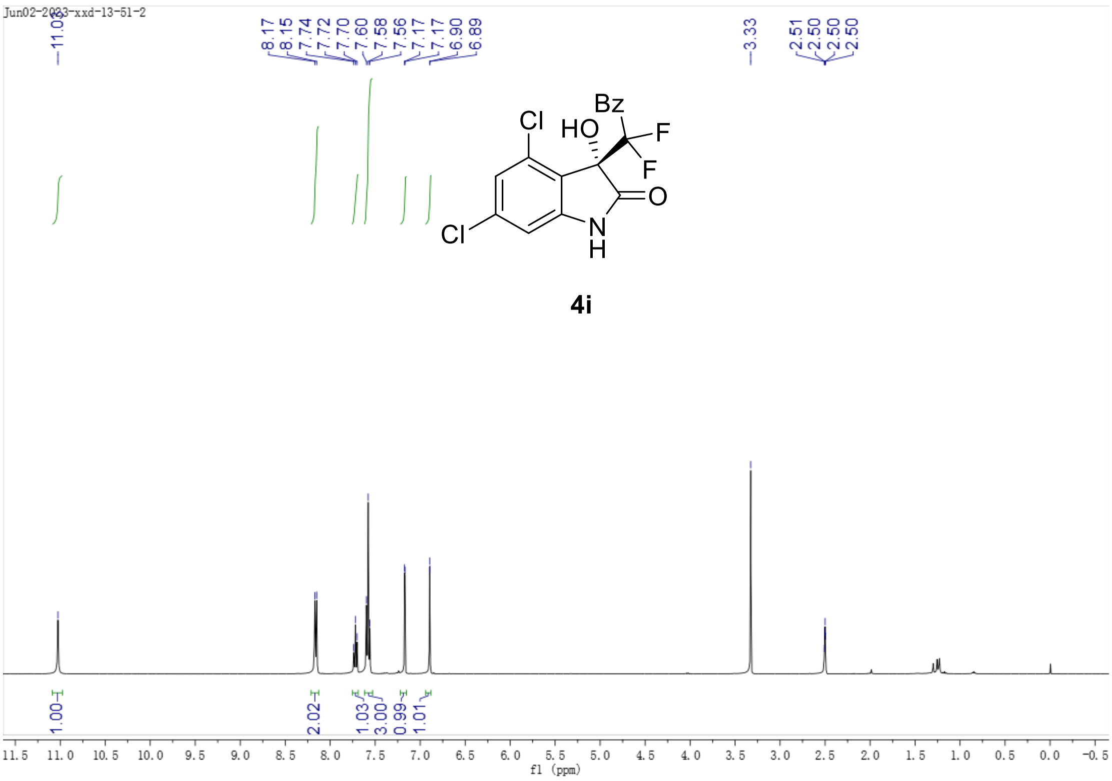

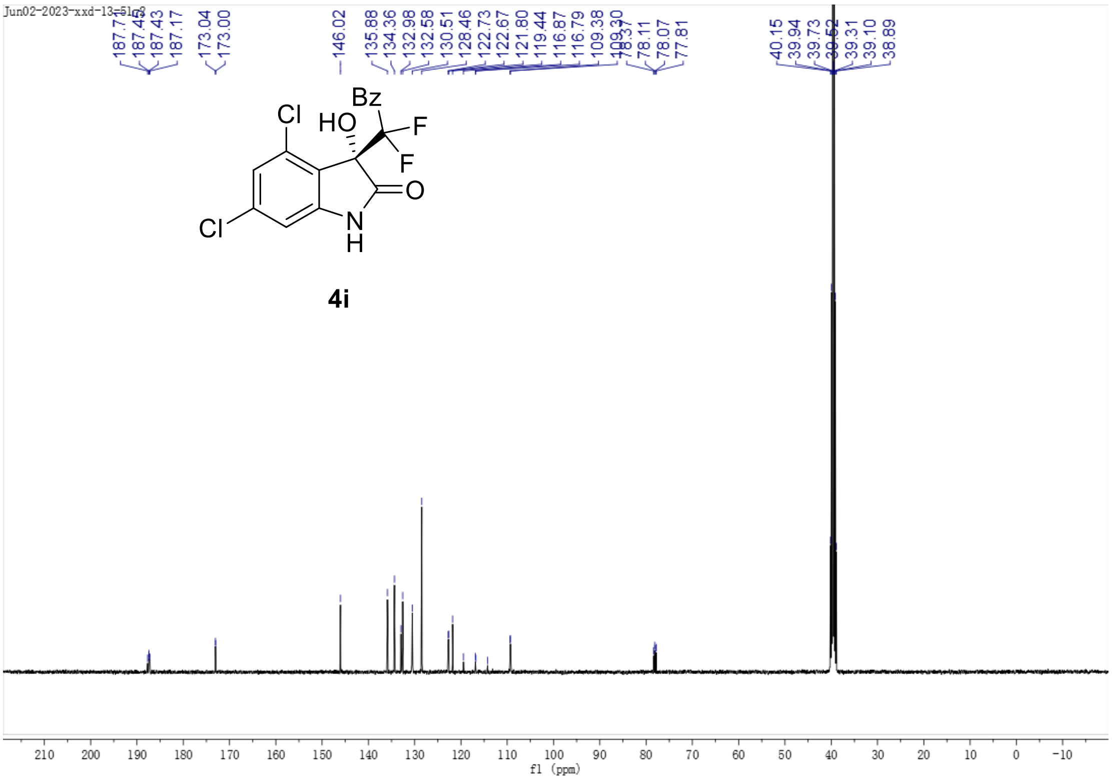

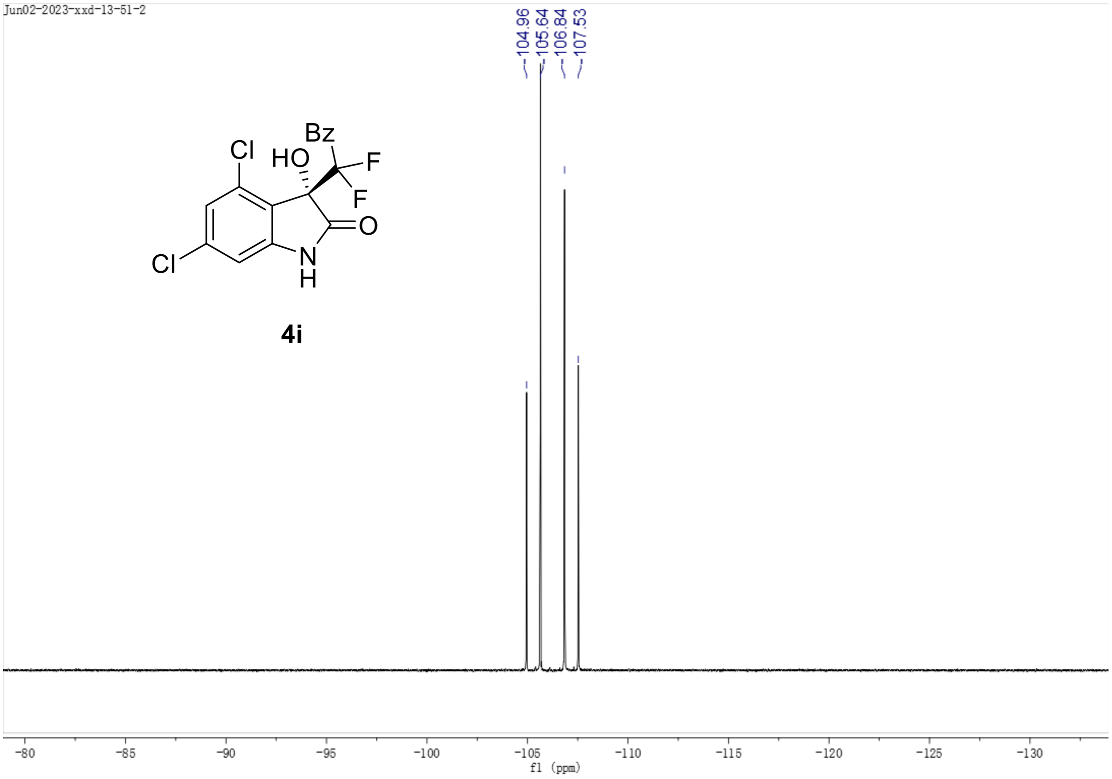
Jun02-2023-xxd-13-51-2
-104.96
-105.64
-106.84
-107.53
Cl
HO
Bz
F
F
O
Cl
N
H
-80
-85
-90
-95
-100
-105
-110
-115
-120
-125
-130
f1 (ppm)

4i

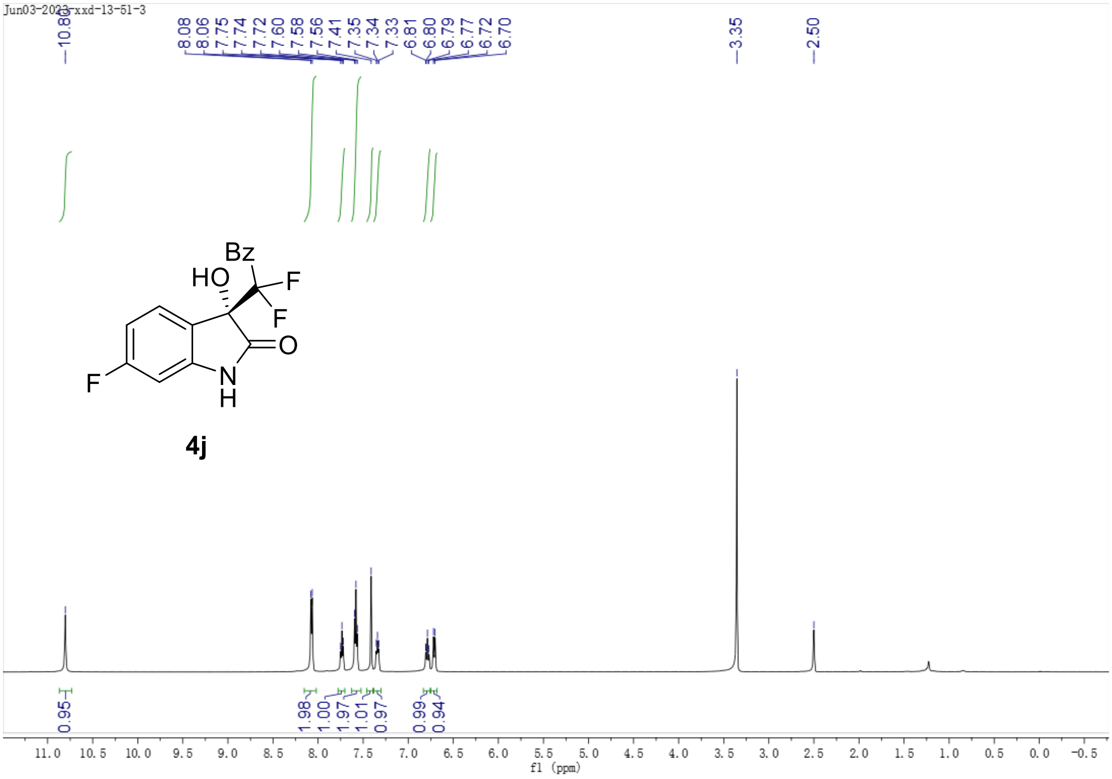
Jun03-2023-xxd-13-51-3
10.80
8.08
8.06
7.75
7.74
7.72
7.60
7.58
7.56
7.41
7.35
7.34
7.33
6.81
6.80
6.79
6.77
6.72
6.70
3.35
2.50
HO
Bz
F
F
O
F
N
H
0.95
1.98
1.00
1.97
1.01
0.97
0.99
0.94
11.0
10.5
10.0
9.5
9.0
8.5
8.0
7.5
7.0
6.5
6.0
5.5
5.0
4.5
4.0
3.5
3.0
2.5
2.0
1.5
1.0
0.5
0.0
-0.5
f1 (ppm)

4j

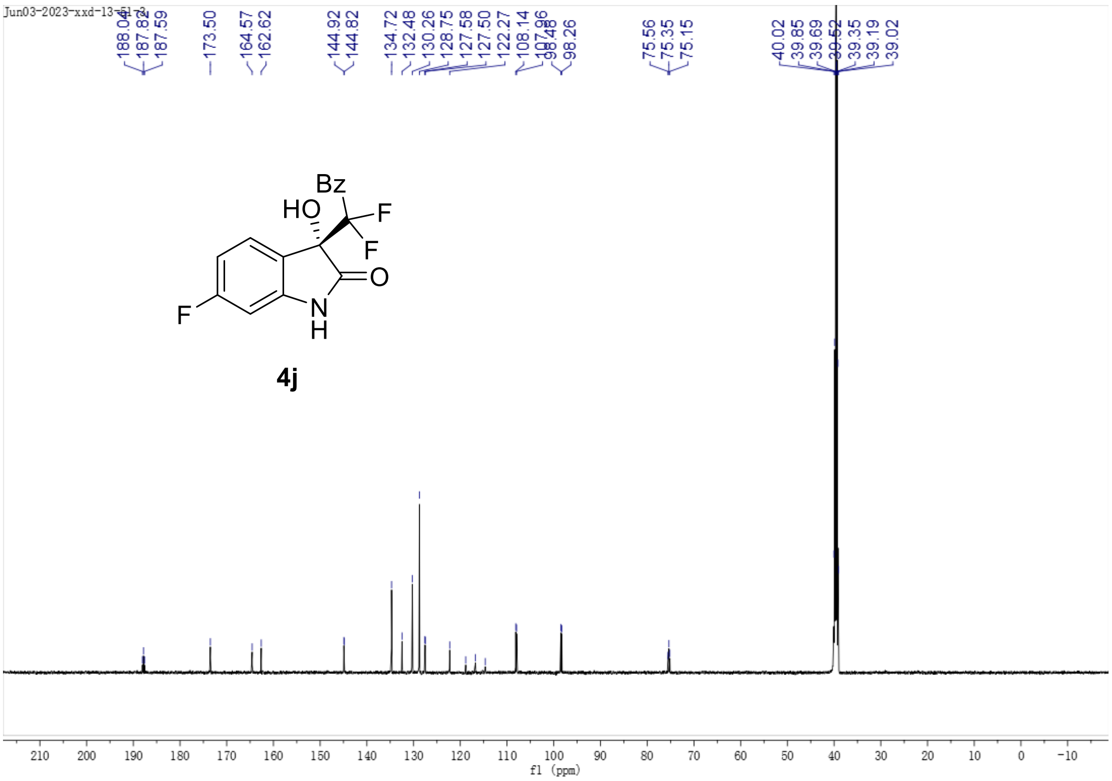
Jun03-2023-xxd-13-51-3
188.04
187.82
187.59
173.50
164.57
162.62
144.92
144.82
134.72
132.48
130.26
128.75
127.58
127.50
122.27
108.14
107.96
98.45
98.26
75.56
75.35
75.15
40.02
39.85
39.69
39.52
39.35
39.19
39.02
Bz
HO
F
F
O
N
H
F
4j
210 200 190 180 170 160 150 140 130 120 110 100 90 80 70 60 50 40 30 20 10 0 -10
f1 (ppm)

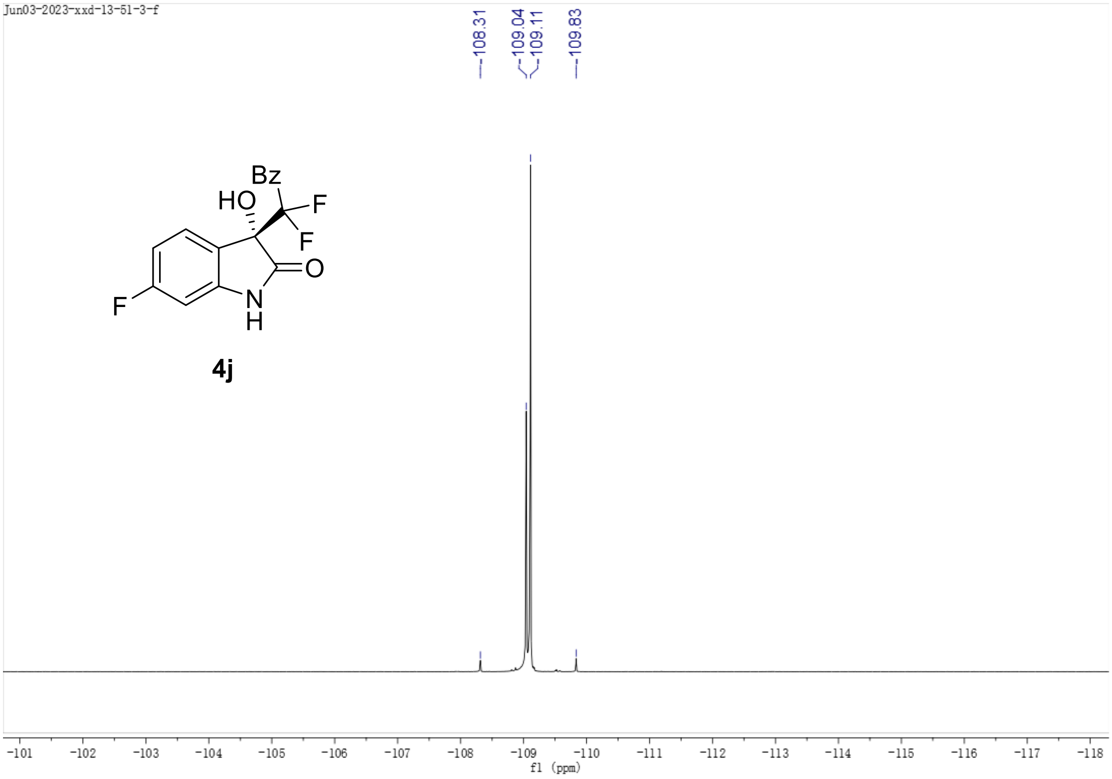
Jun03-2023-xxd-13-51-3-f
-108.31
-109.04
-109.11
-109.83
Bz
HO
F
F
O
N
H
F
4j
-101 -102 -103 -104 -105 -106 -107 -108 -109 -110 -111 -112 -113 -114 -115 -116 -117 -118
f1 (ppm)

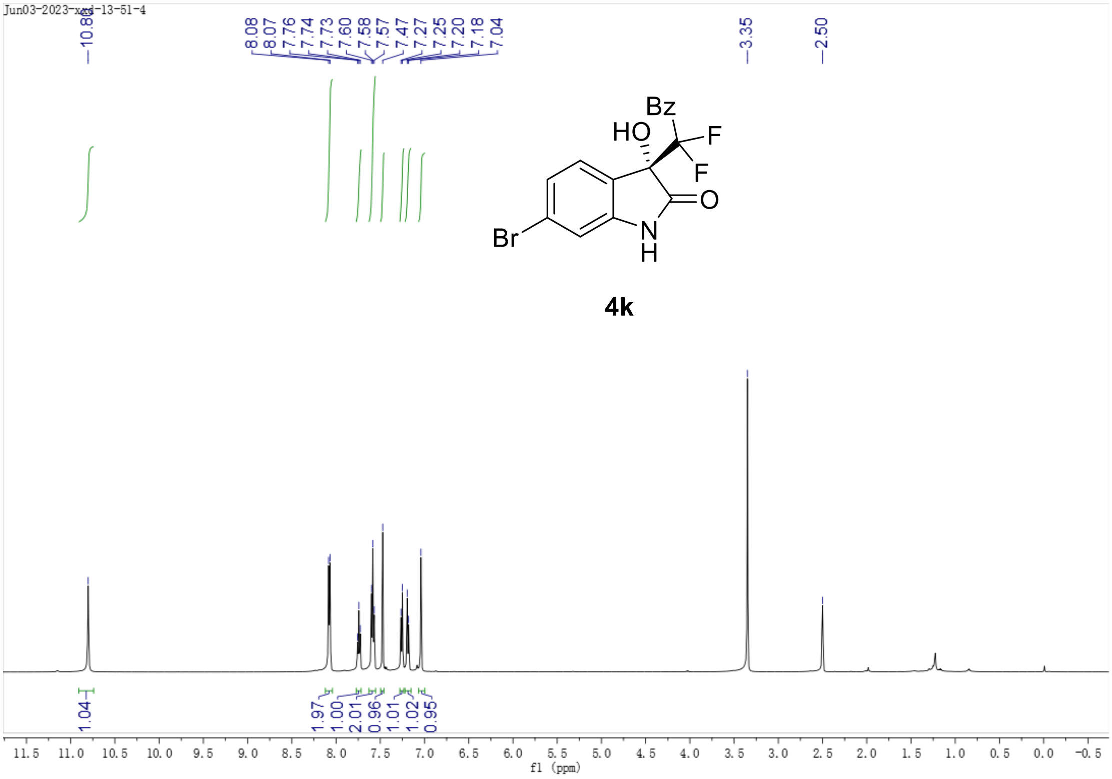
Jun03-2023-xxd-13-51-4
10.80
8.08
8.07
7.76
7.74
7.73
7.60
7.58
7.57
7.47
7.27
7.25
7.20
7.18
7.04
3.35
2.50
Bz
HO
F
F
O
N
H
Br
4k
1.04
1.97
1.00
2.01
0.96
1.01
1.02
0.95
f1 (ppm)

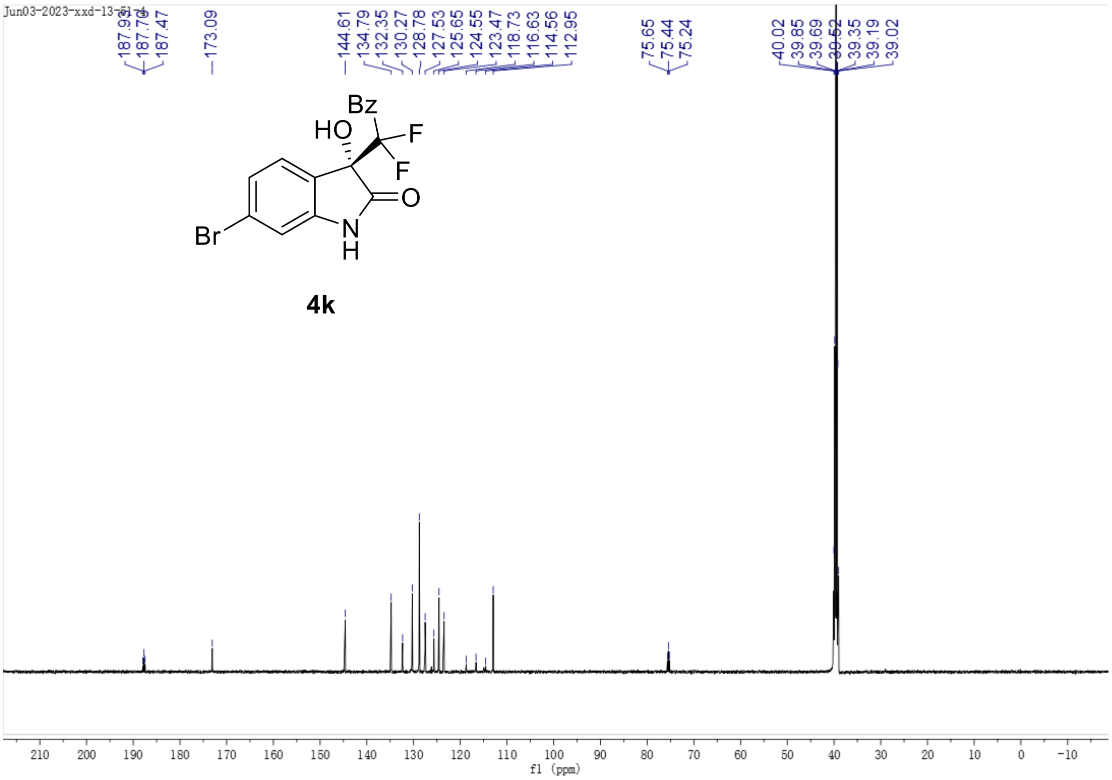
187.93
187.70
187.47
173.09
144.61
134.79
132.35
130.27
128.78
127.53
125.65
124.55
123.47
118.73
116.63
114.56
112.95
75.65
75.44
75.24
40.02
39.85
39.69
39.52
39.35
39.19
39.02
Bz
HO
F
F
O
N
H
Br
4k
f1 (ppm)

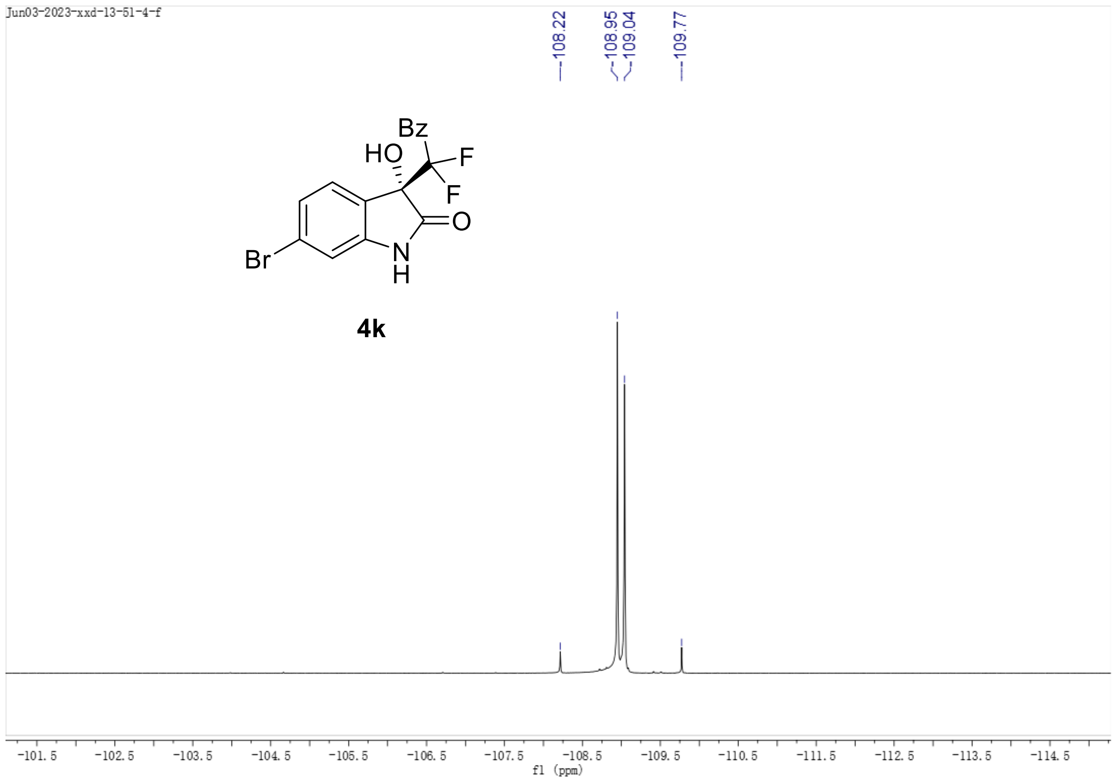
Jun03-2023-xxd-13-51-4-f
108.22
108.95
109.04
109.77
Bz
HO
F
F
O
Br
N
H
4k
-101.5
-102.5
-103.5
-104.5
-105.5
-106.5
-107.5
-108.5
-109.5
-110.5
-111.5
-112.5
-113.5
-114.5
f1 (ppm)

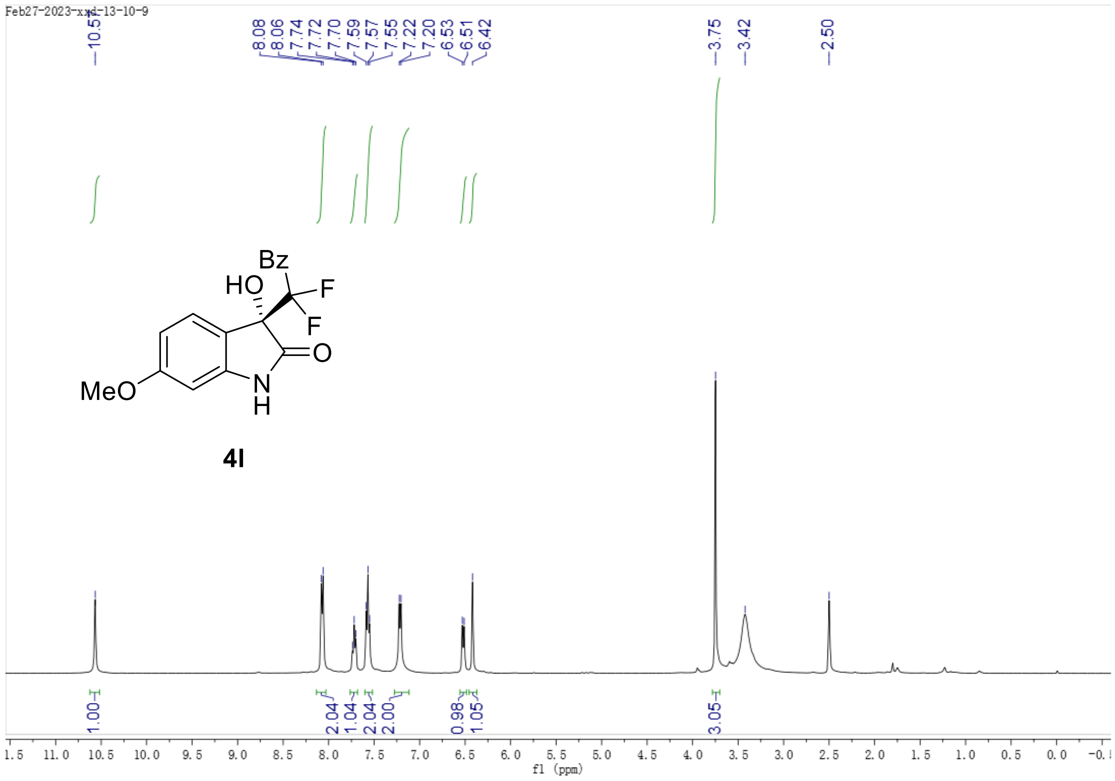
Feb27-2023-xxd-13-10-9
10.57
8.08
8.06
7.74
7.72
7.70
7.59
7.57
7.55
7.22
7.20
6.53
6.51
6.42
3.75
3.42
2.50
Bz
HO
F
F
O
MeO
N
H
4l
1.00
2.04
1.04
2.04
2.00
0.98
1.05
3.05
f1 (ppm)

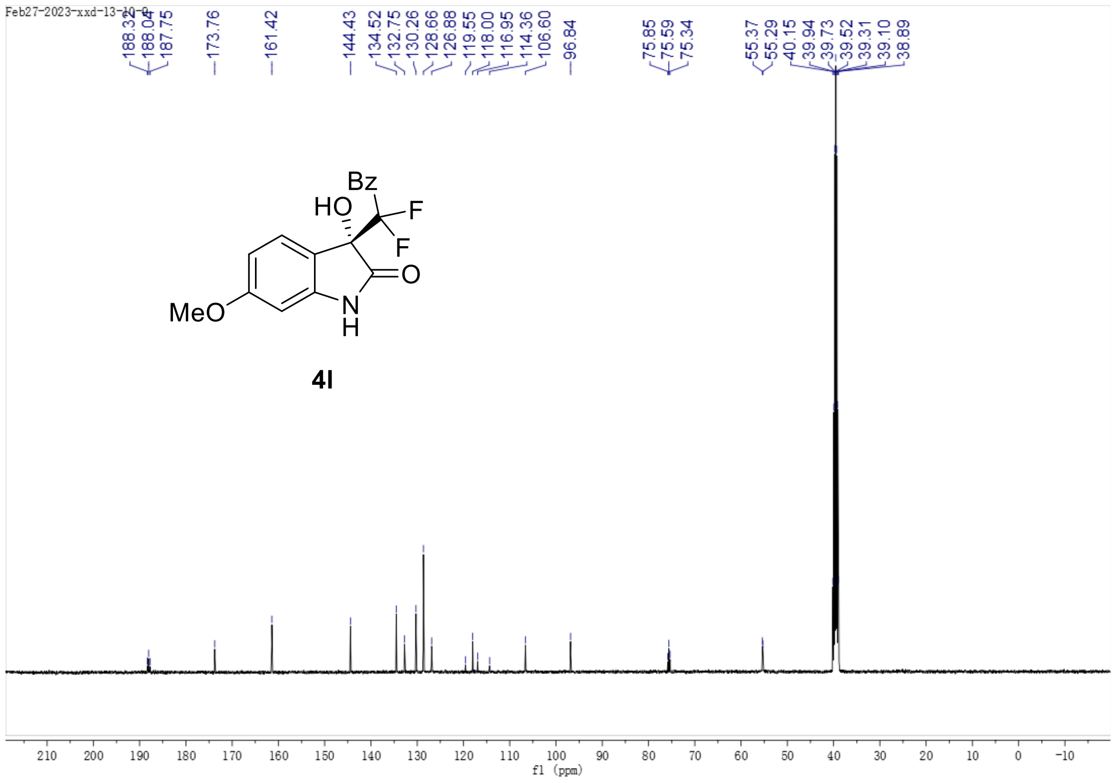
Feb27-2023-xxd-13-10-9
188.32
188.04
187.75
173.76
161.42
144.43
134.52
132.75
130.26
128.66
126.88
119.55
118.00
116.95
114.36
106.60
96.84
75.85
75.59
75.34
55.37
55.29
40.15
39.94
39.73
39.52
39.31
39.10
38.89
Bz
HO
F
F
O
MeO
N
H
4l
f1 (ppm)

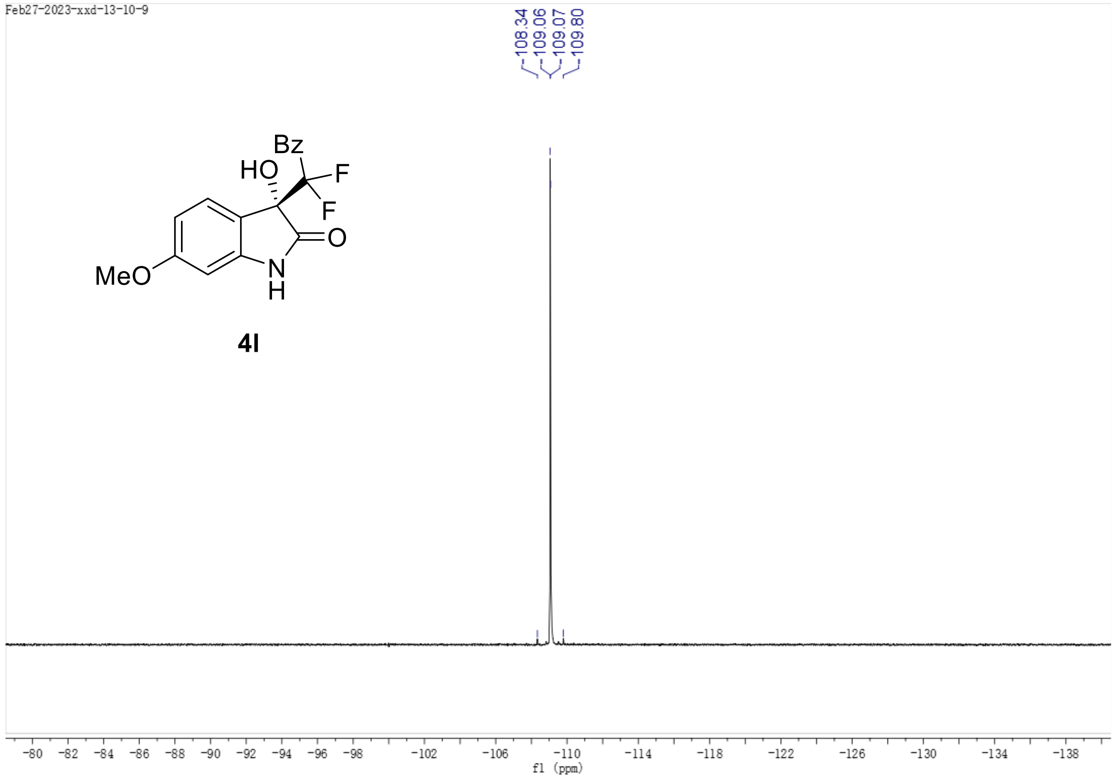
Feb27-2023-xxd-13-10-9
-108.34
-109.06
-109.07
-109.80
Bz
HO
F
F
O
MeO
N
H
4l
f1 (ppm)

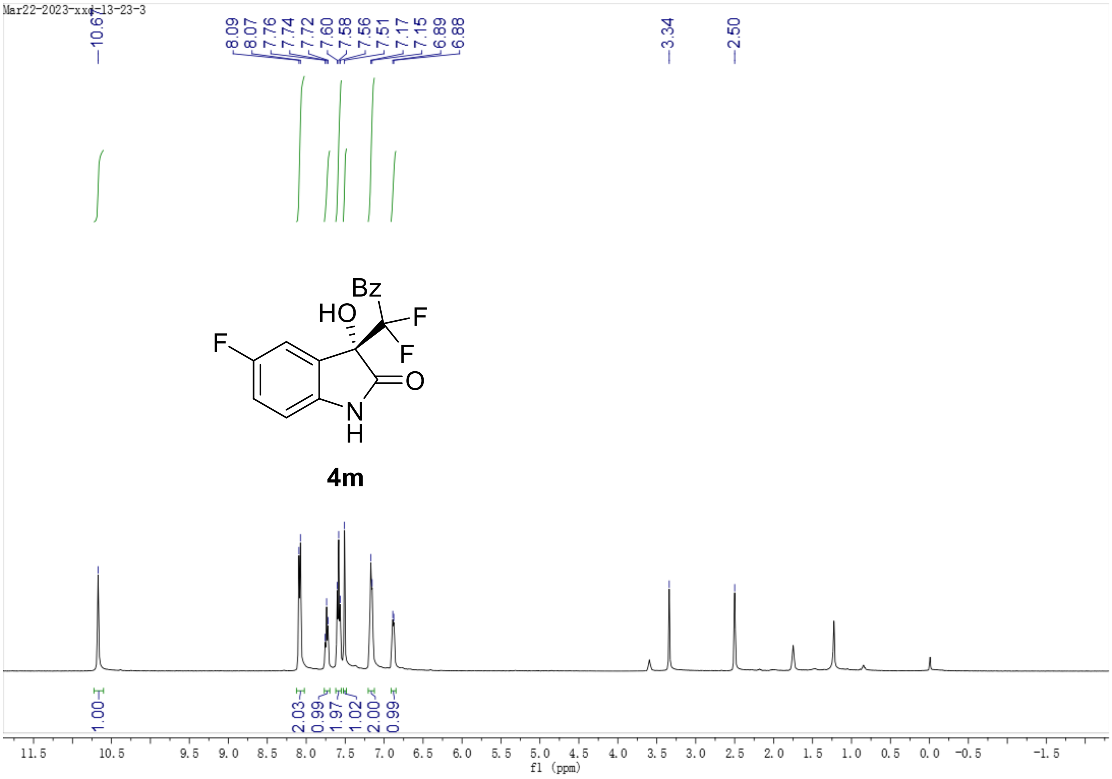
Mar22-2023-xxd-13-23-3
10.61
8.09
8.07
7.76
7.74
7.72
7.60
7.58
7.56
7.51
7.17
7.15
6.89
6.88
3.34
2.50
Bz
HO
F
F
F
O
N
H
4m
1.00
2.03
0.99
1.97
1.02
2.00
0.99
11.5 10.5 9.5 9.0 8.5 8.0 7.5 7.0 6.5 6.0 5.5 5.0 4.5 4.0 3.5 3.0 2.5 2.0 1.5 1.0 0.5 0.0 -0.5 -1.5
f1 (ppm)

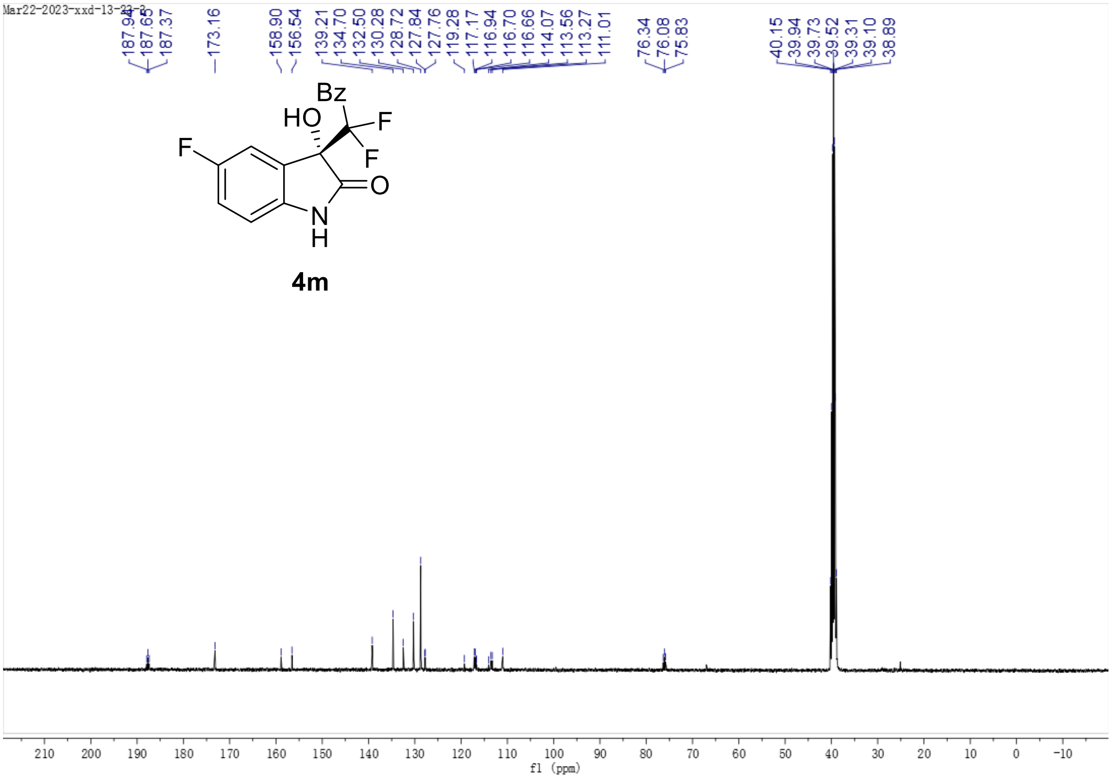
Mar22-2023-xxd-13-23-2
187.65
187.37
173.16
158.90
156.54
139.21
134.70
132.50
130.28
128.72
127.84
127.76
119.28
117.17
116.94
116.70
116.66
114.07
113.56
113.27
111.01
76.34
76.08
75.83
40.15
39.94
39.73
39.52
39.31
39.10
38.89
Bz
HO
F
F
F
O
N
H
4m
210 200 190 180 170 160 150 140 130 120 110 100 90 80 70 60 50 40 30 20 10 0 -10
f1 (ppm)

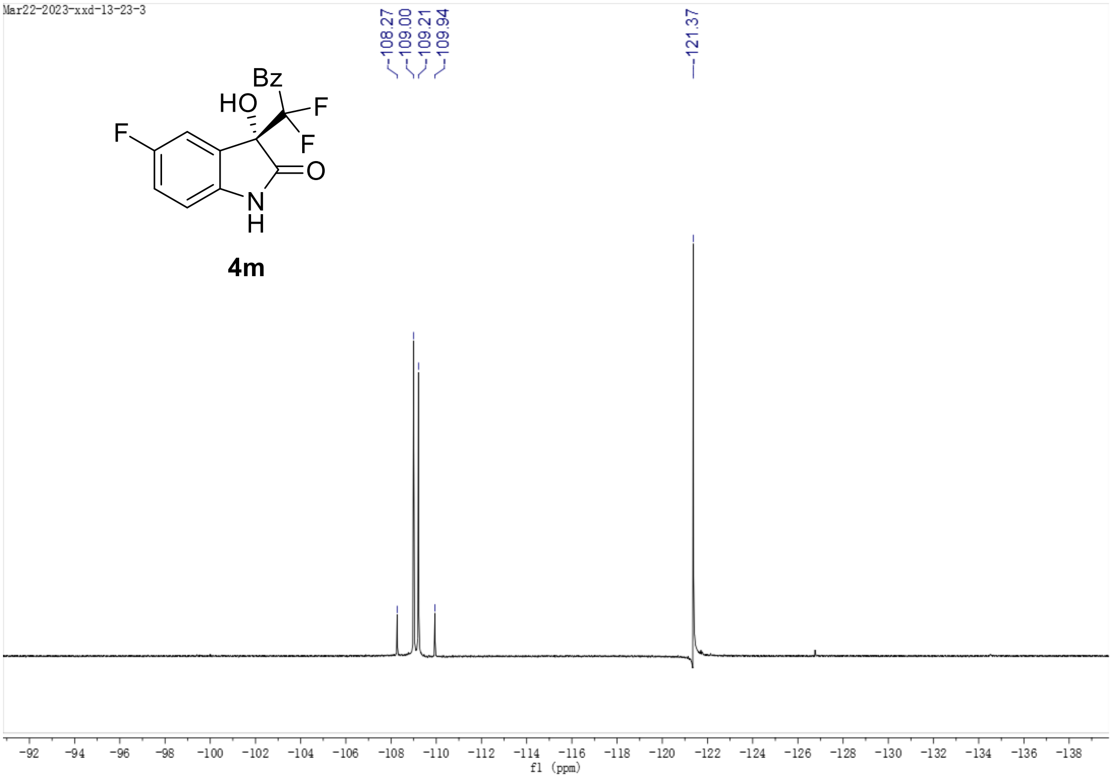
Mar22-2023-xxd-13-23-3
-108.27
-109.00
-109.21
-109.94
-121.37
Bz
HO
F
F
F
O
N
H
4m
-92 -94 -96 -98 -100 -102 -104 -106 -108 -110 -112 -114 -116 -118 -120 -122 -124 -126 -128 -130 -132 -134 -136 -138
f1 (ppm)

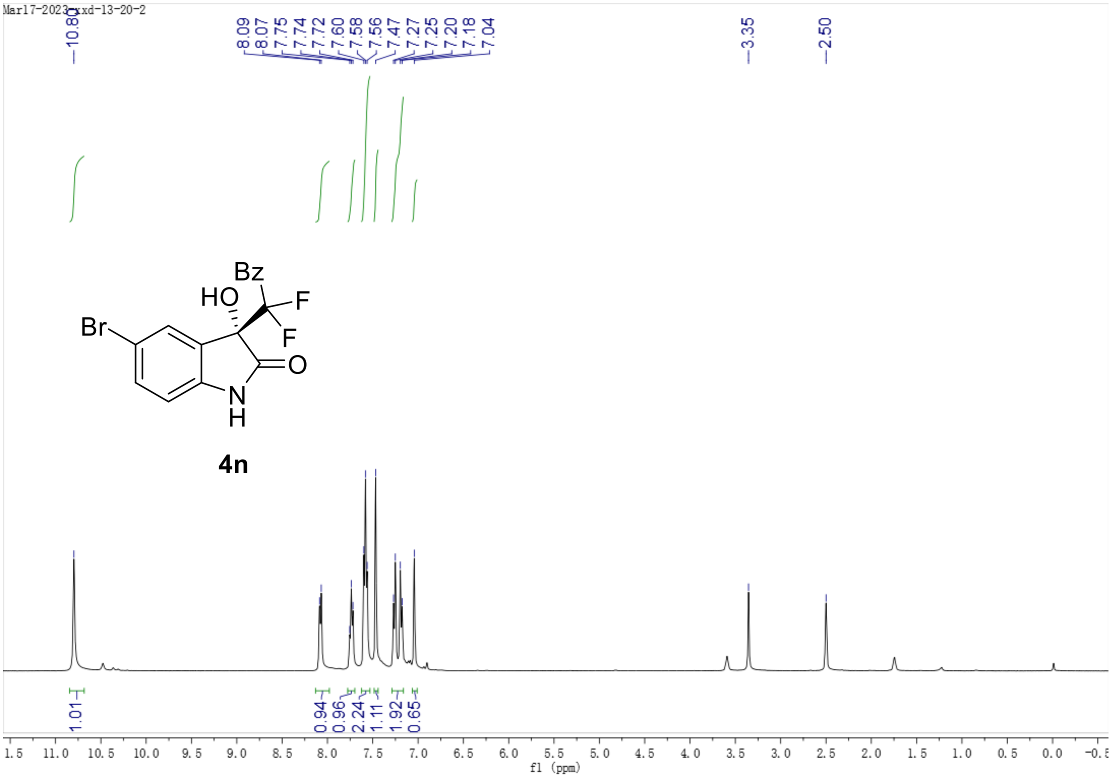
Mar17-2023-xxd-13-20-2
10.80
8.09
8.07
7.75
7.74
7.72
7.60
7.58
7.56
7.47
7.27
7.25
7.20
7.18
7.04
3.35
2.50
Bz
HO
Br
F
F
O
N
H
4n
1.01
0.94
0.96
2.24
1.11
1.92
0.65
11.0 10.5 10.0 9.5 9.0 8.5 8.0 7.5 7.0 6.5 6.0 5.5 5.0 4.5 4.0 3.5 3.0 2.5 2.0 1.5 1.0 0.5 0.0
f1 (ppm)

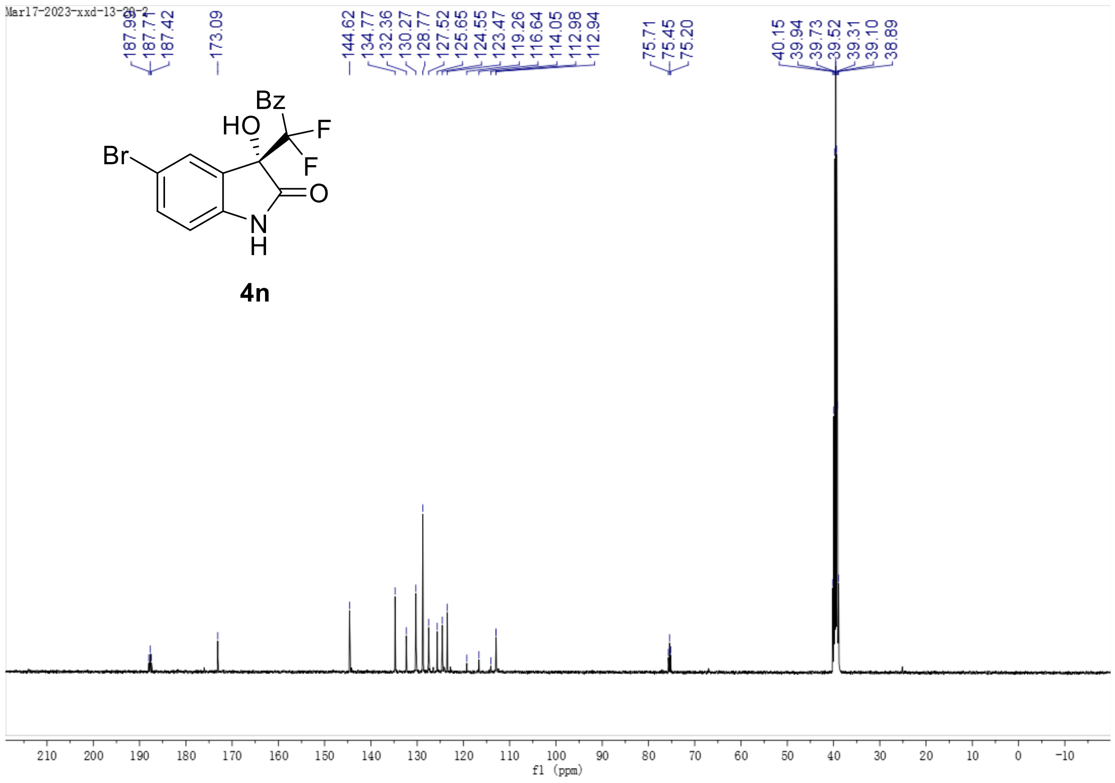
Mar17-2023-xxd-13-20-2
187.99
187.71
187.42
173.09
144.62
134.77
132.36
130.27
128.77
127.52
125.65
124.55
123.47
119.26
116.64
114.05
112.98
112.94
75.71
75.45
75.20
40.15
39.94
39.73
39.52
39.31
39.10
38.89
Bz
HO
F
Br
F
O
N
H
4n
f1 (ppm)

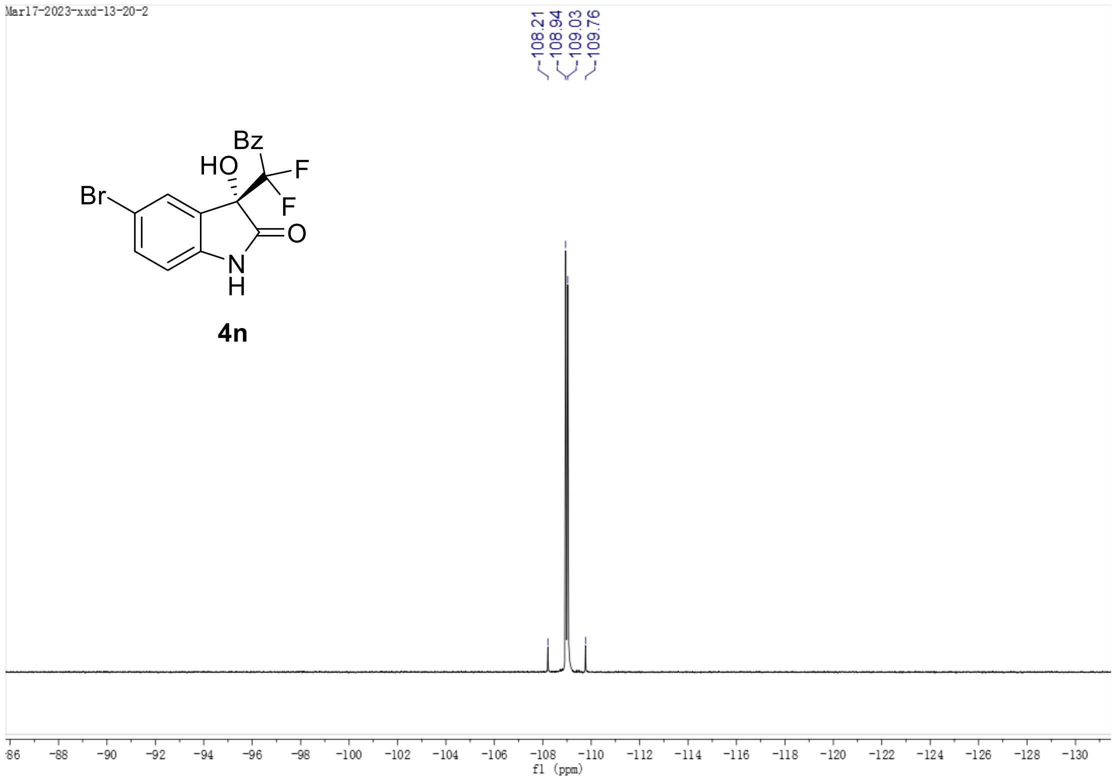
Mar17-2023-xxd-13-20-2
-108.21
-108.94
-109.03
-109.76
Bz
HO
F
Br
F
O
N
H
4n
f1 (ppm)

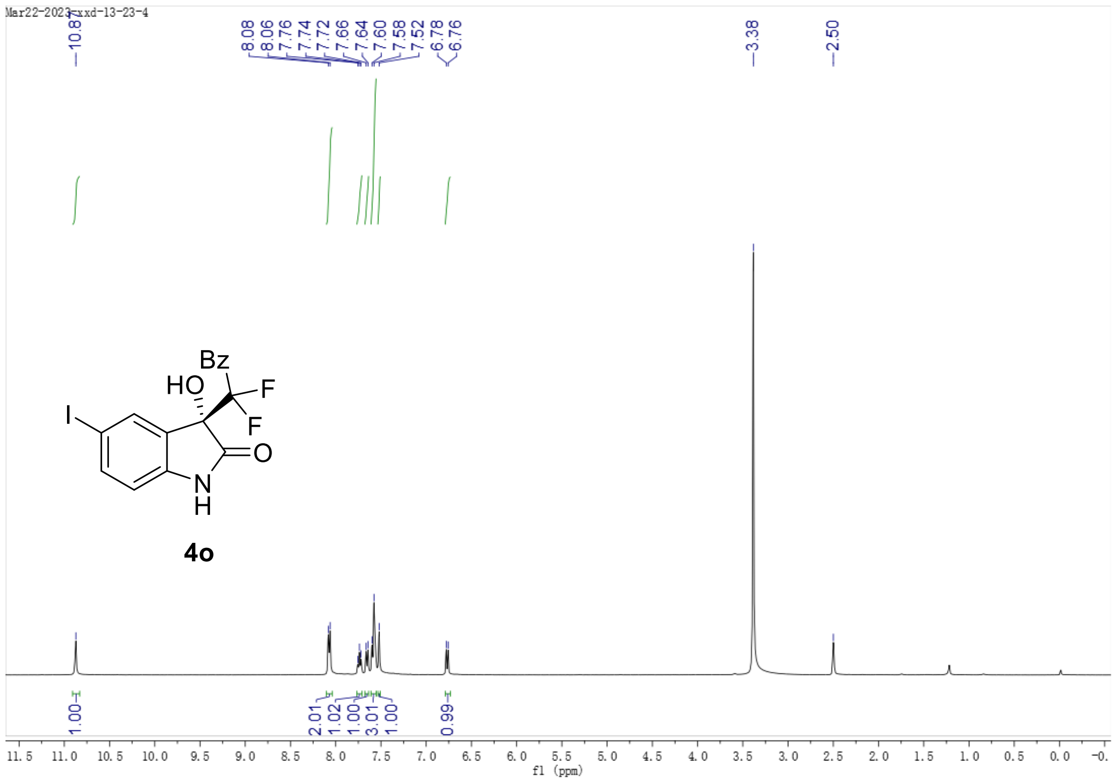

Mar22-2023-xxd-13-23-4
10.87
8.08
8.06
7.76
7.74
7.72
7.66
7.64
7.60
7.58
7.52
6.78
6.76
3.38
2.50
Bz
HO
F
F
I
O
N
H
4o
1.00
2.01
1.02
1.00
3.01
1.00
0.99
f1 (ppm)

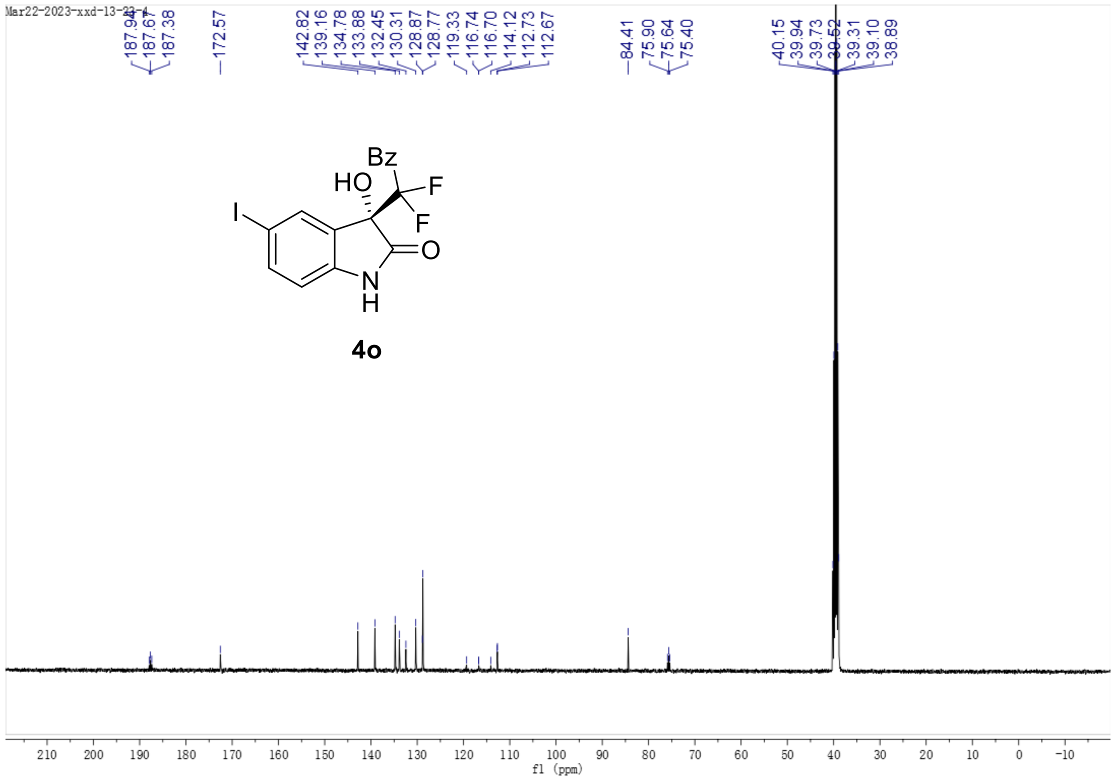

Mar22-2023-xxd-13-23-4
187.94
187.67
187.38
172.57
142.82
139.16
134.78
133.88
132.45
130.31
128.87
128.77
119.33
116.74
116.70
114.12
112.73
112.67
84.41
75.90
75.64
75.40
40.15
39.94
39.73
39.52
39.31
39.10
38.89
Bz
HO
F
F
I
O
N
H
4o
f1 (ppm)

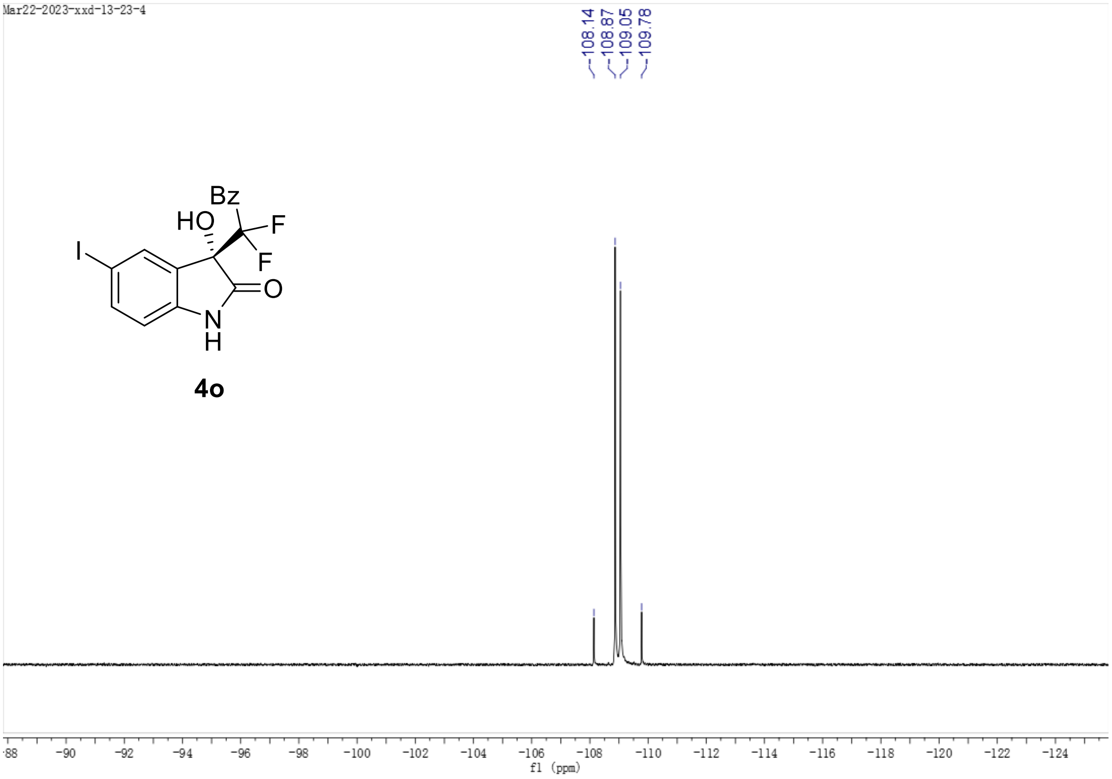

Mar22-2023-xxd-13-23-4
-108.14
-108.87
-109.05
-109.78
Bz
HO
F
F
I
O
N
H
4o
f1 (ppm)

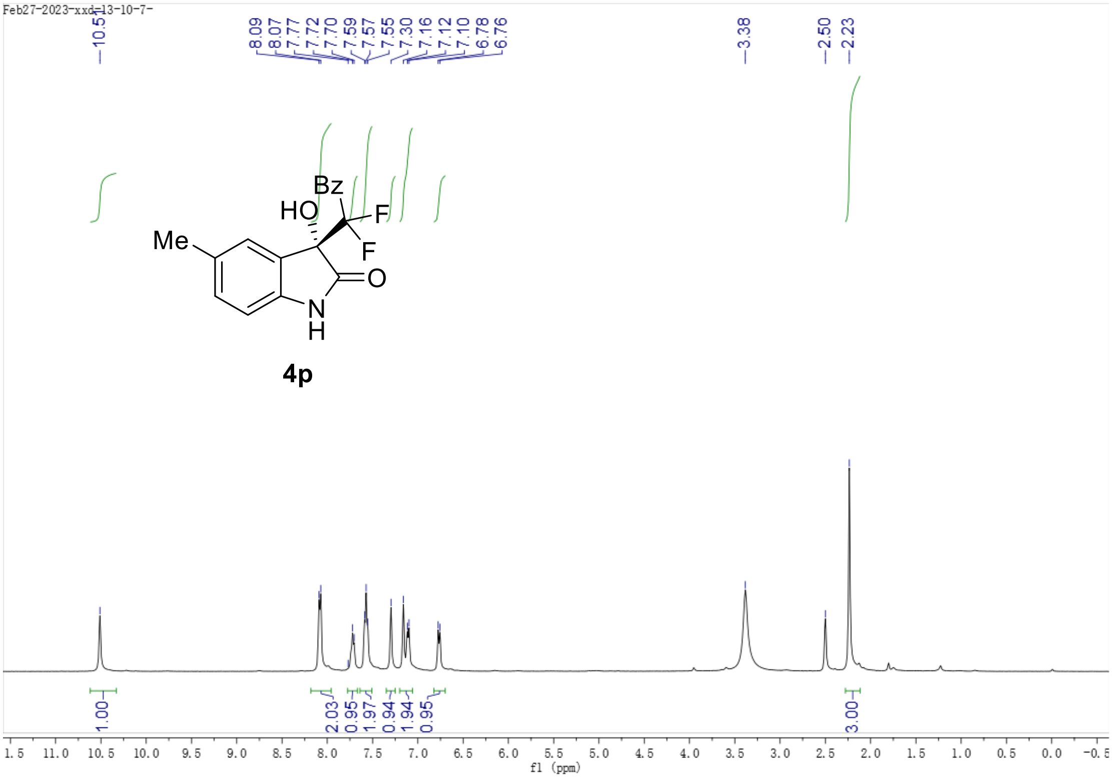

Feb27-2023-xxd-13-10-7-
-10.51
8.09
8.07
7.77
7.72
7.70
7.59
7.57
7.55
7.30
7.16
7.12
7.10
6.78
6.76
-3.38
-2.50
-2.23
Bz
HO
F
F
Me
O
N
H
4p
1.00
2.03
0.95
1.97
0.94
1.94
0.95
3.00
f1 (ppm)

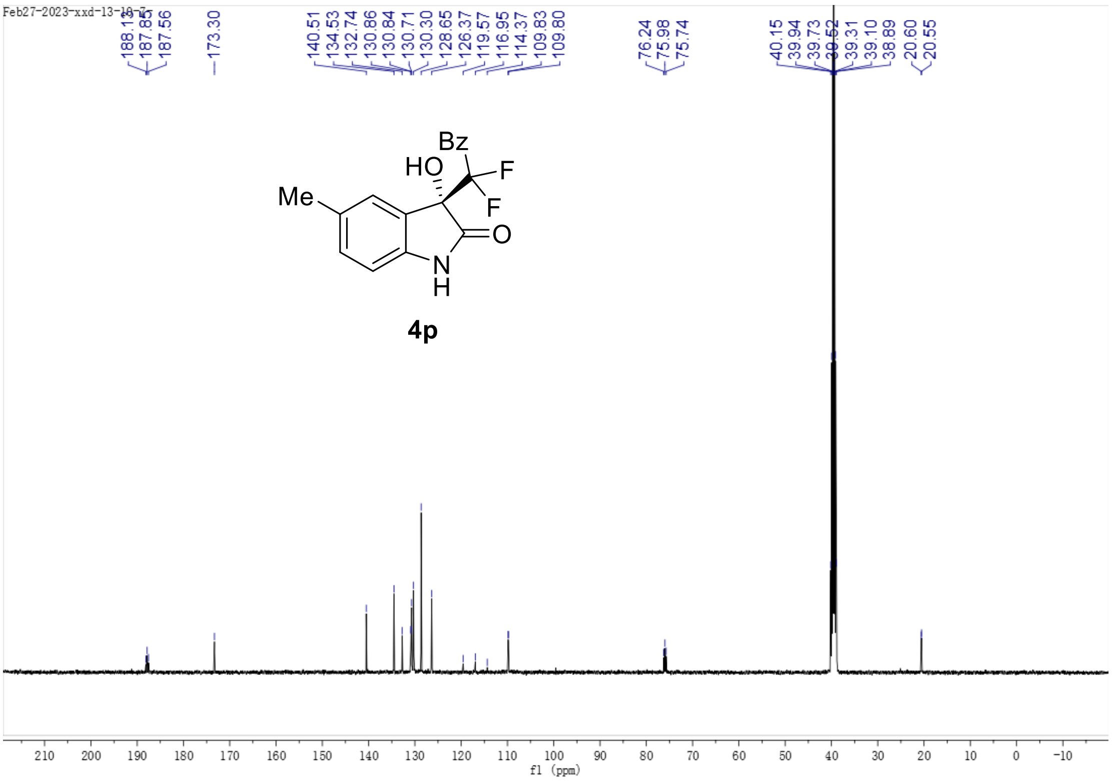
Feb27-2023-xxd-13-10-7-
188.13
187.85
187.56
173.30
140.51
134.53
132.74
130.86
130.84
130.71
130.30
128.65
126.37
119.57
116.95
114.37
109.83
109.80
76.24
75.98
75.74
40.15
39.94
39.73
39.52
39.31
39.10
38.89
20.60
20.55
Bz
HO
F
F
Me
O
N
H
4p
210 200 190 180 170 160 150 140 130 120 110 100 90 80 70 60 50 40 30 20 10 0 -10
f1 (ppm)

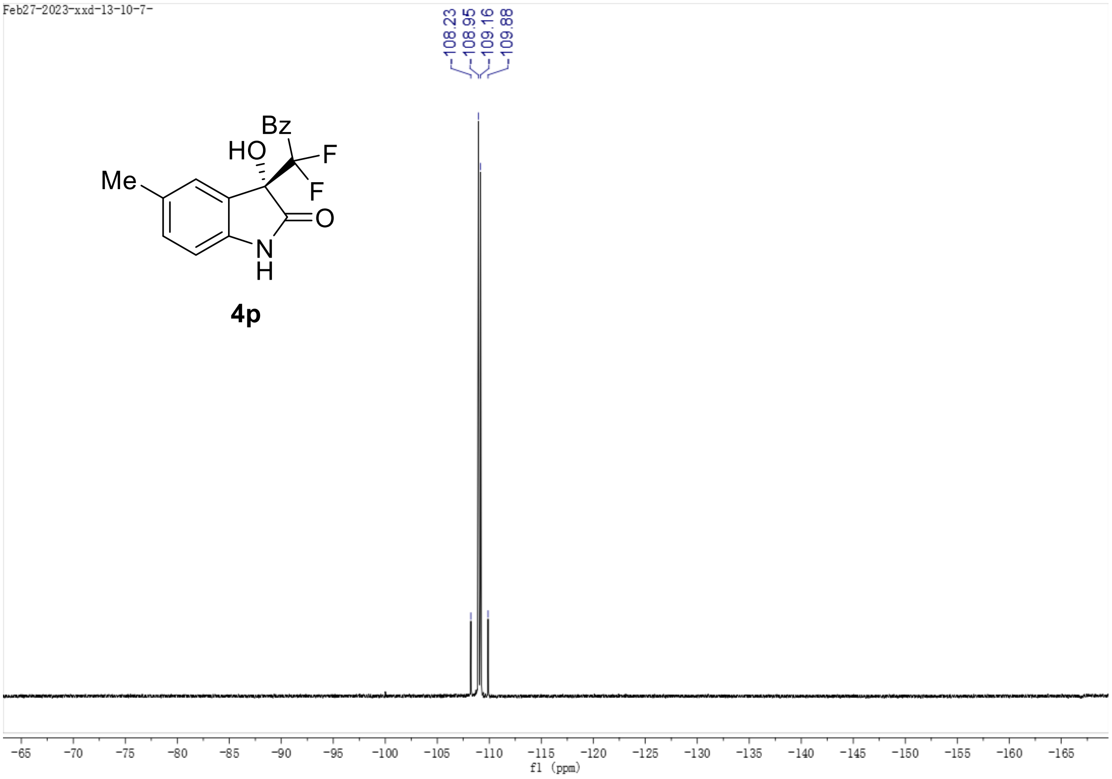
Feb27-2023-xxd-13-10-7-
-108.23
-108.95
-109.16
-109.88
Bz
HO
F
F
Me
O
N
H
4p
-65 -70 -75 -80 -85 -90 -95 -100 -105 -110 -115 -120 -125 -130 -135 -140 -145 -150 -155 -160 -165
f1 (ppm)

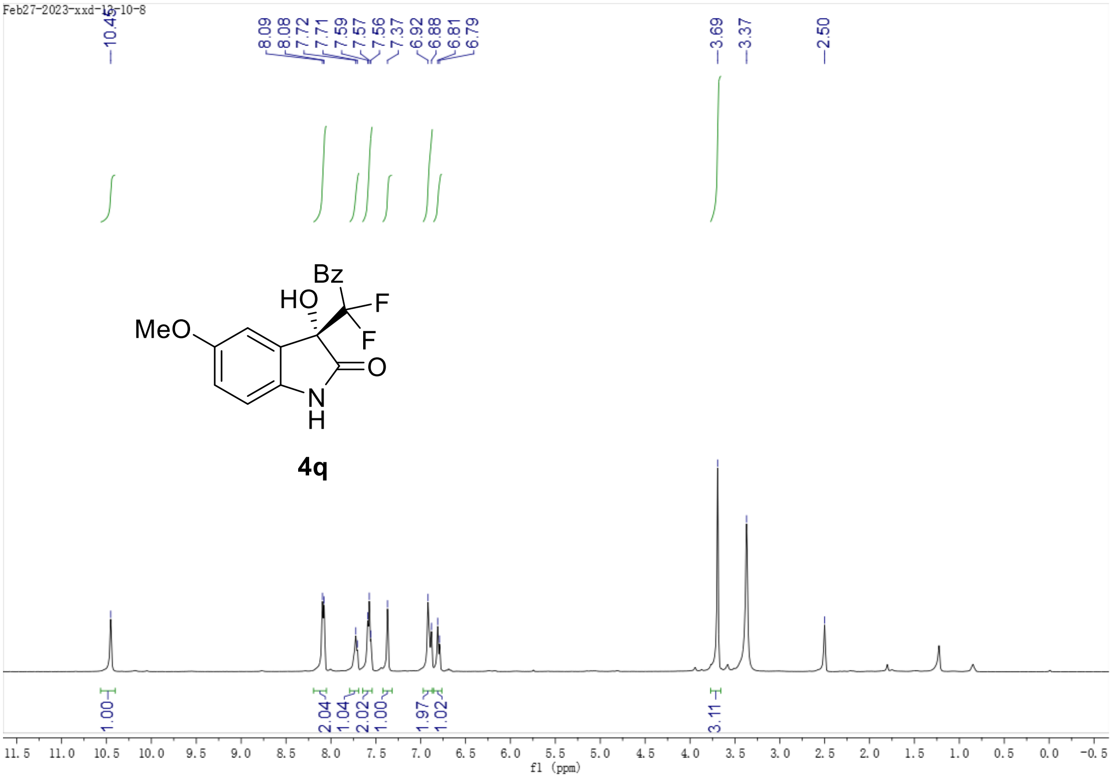

Feb27-2023-xxd-13-10-8
MeO
HO
Bz
F
F
O
N
H
4q
f1 (ppm)

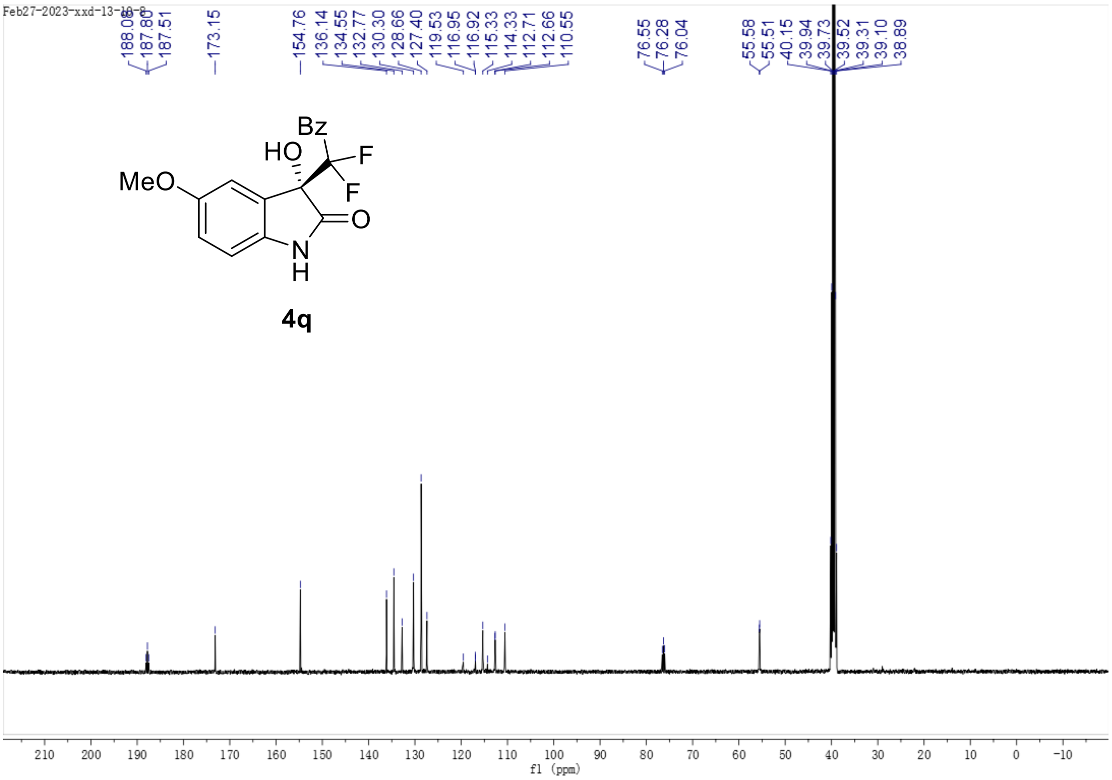

Feb27-2023-xxd-13-10-8
MeO
HO
Bz
F
F
O
N
H
4q
f1 (ppm)

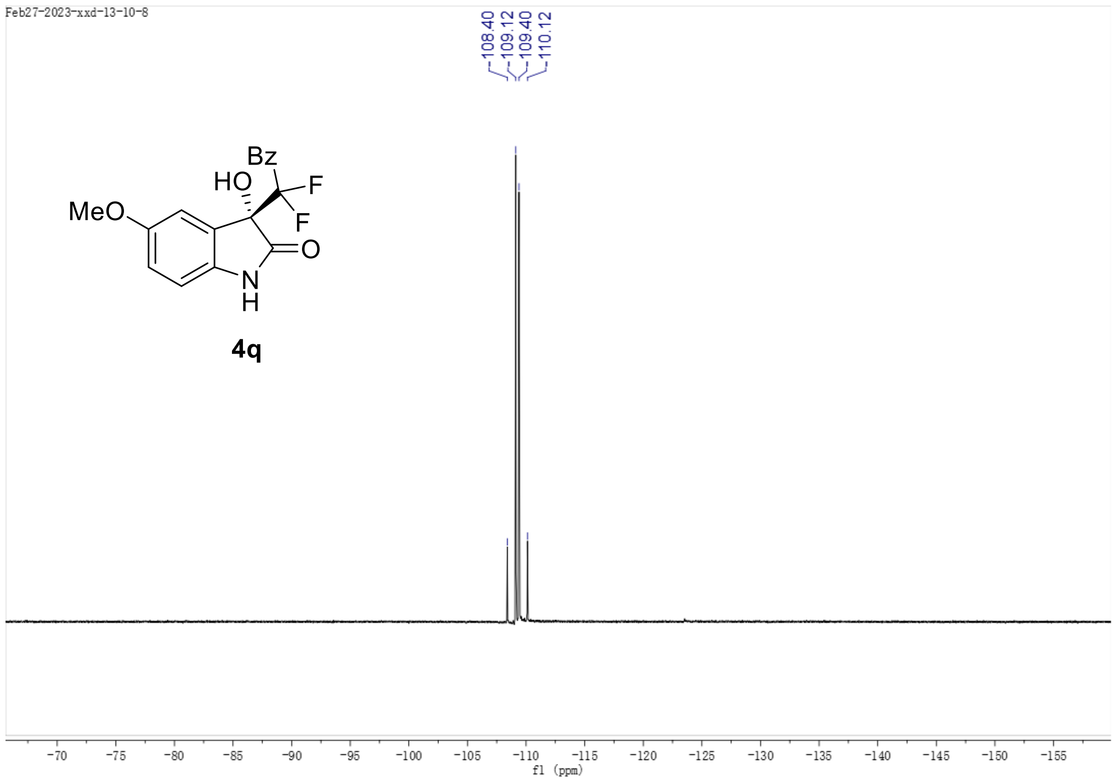

Feb27-2023-xxd-13-10-8
-108.40
-109.12
-109.40
-110.12
Bz
HO
F
F
MeO
O
N
H
4q
f1 (ppm)

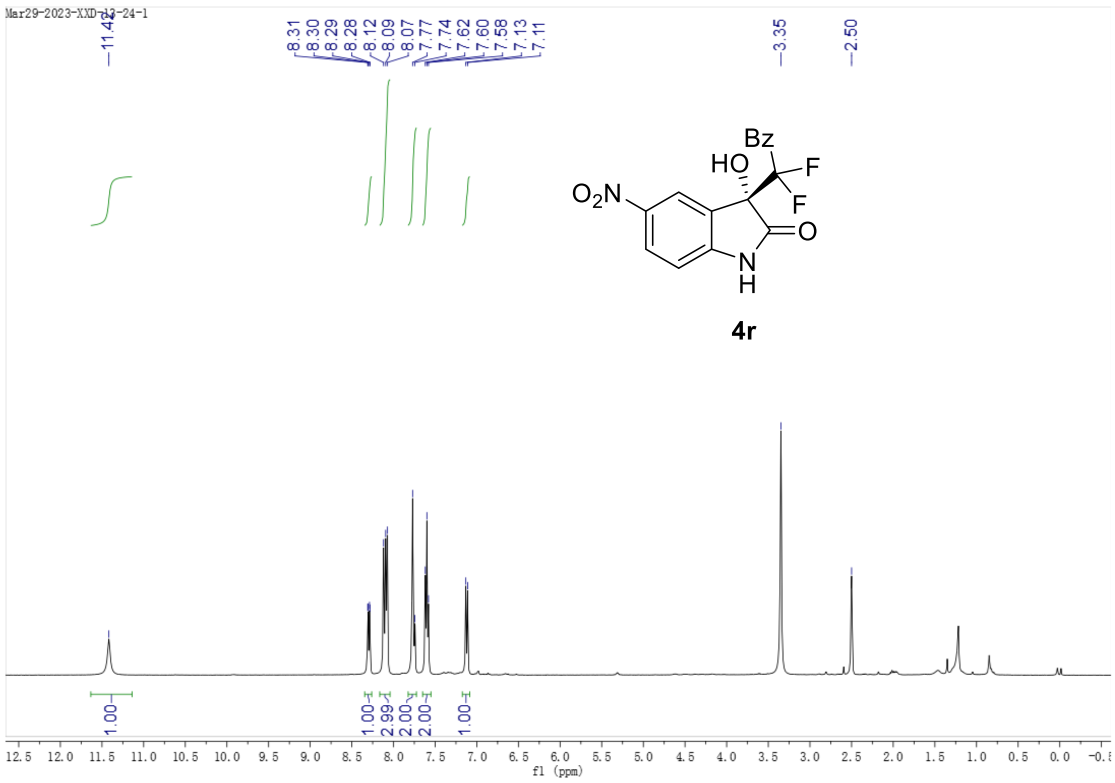

Mar29-2023-XXD-13-24-1
-11.42
8.31
8.30
8.29
8.28
8.12
8.09
8.07
7.77
7.74
7.62
7.60
7.58
7.13
7.11
-3.35
-2.50
Bz
HO
F
F
O2N
O
N
H
4r
1.00
1.00
2.99
2.00
2.00
1.00
f1 (ppm)

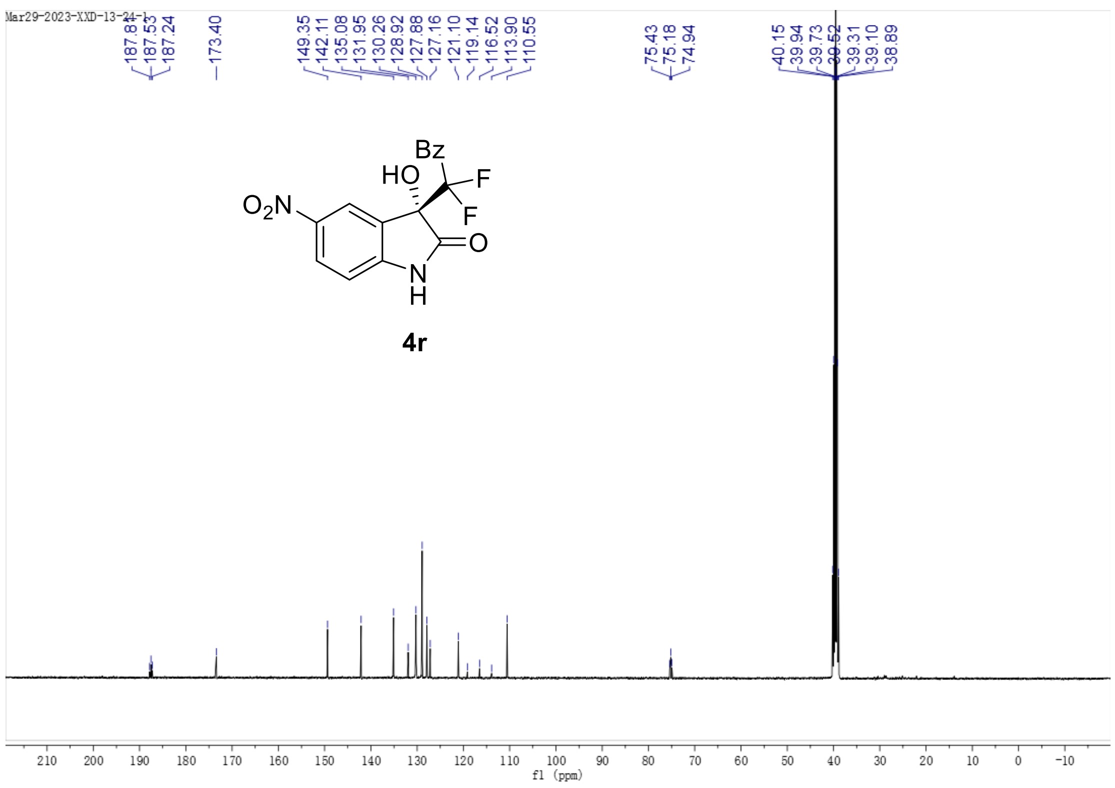
Mar29-2023-XXD-13-24-1
187.84
187.53
187.24
173.40
149.35
142.11
135.08
131.95
130.26
128.92
127.88
127.16
121.10
119.14
116.52
113.90
110.55
75.43
75.18
74.94
40.15
39.94
39.73
39.52
39.31
39.10
38.89
Bz
HO
F
F
O_2N
O
N
H
4r
f1 (ppm)

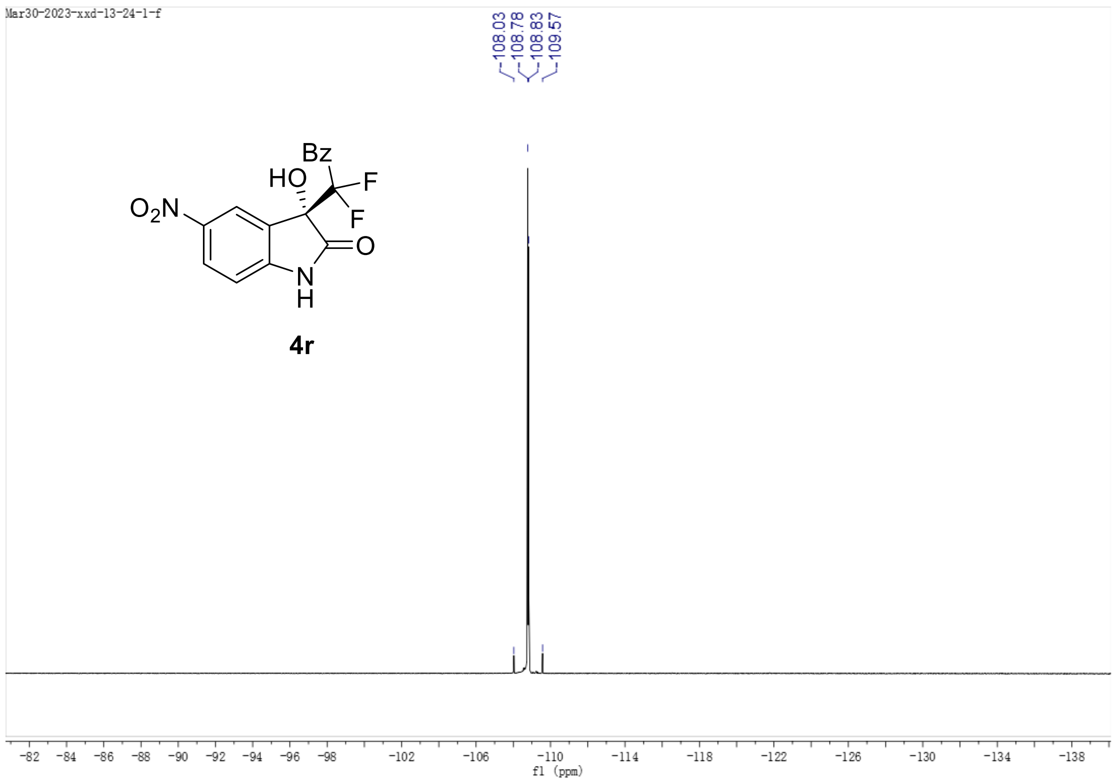
Mar30-2023-xxd-13-24-1-f
-108.03
-108.78
-108.83
-109.57
Bz
HO
F
F
O_2N
O
N
H
4r
f1 (ppm)

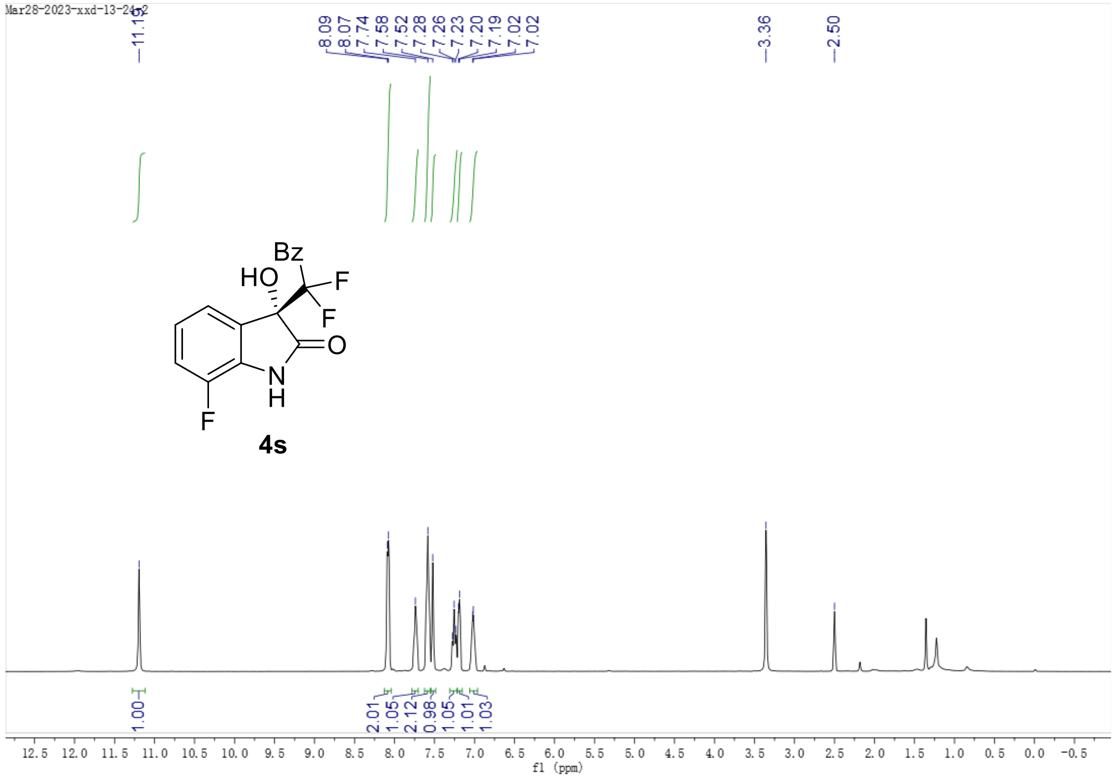
Mar28-2023-xxd-13-24-2
4s
f1 (ppm)

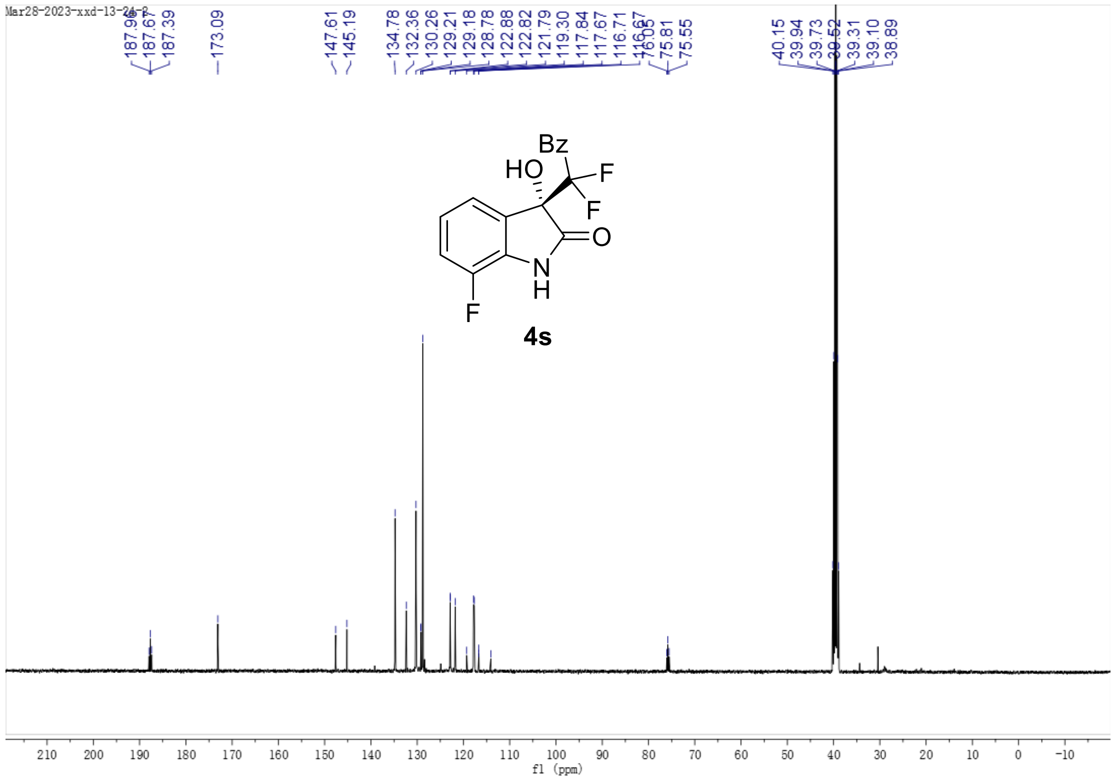
Mar28-2023-xxd-13-24-2
4s
f1 (ppm)

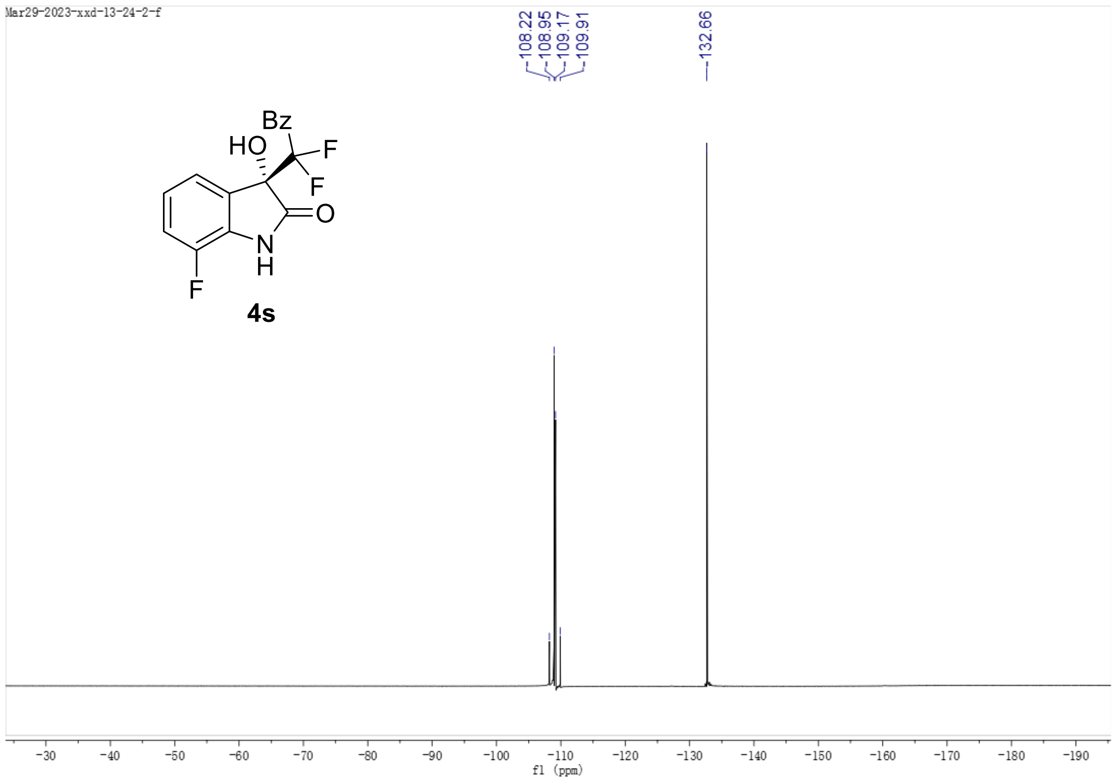
Mar29-2023-xxd-13-24-2-f
-108.22
-108.95
-109.17
-109.91
-132.66
Bz
HO
F
F
O
N
H
F
4s
f1 (ppm)

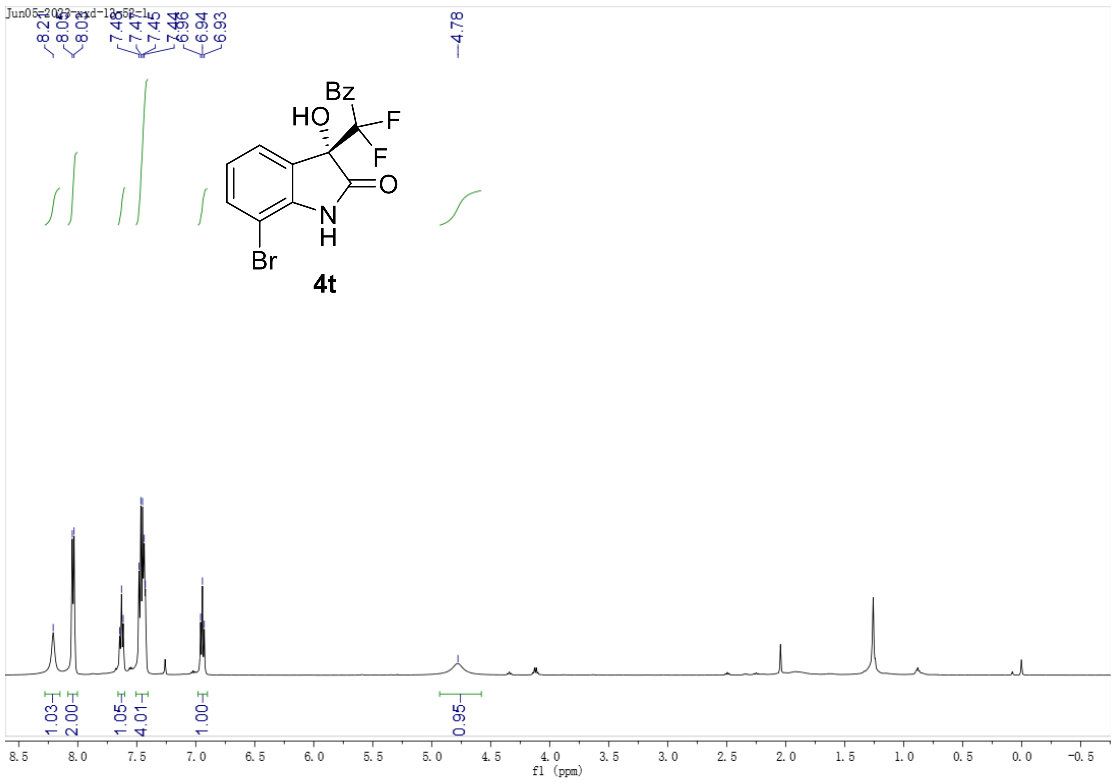
8.21
8.04
8.03
7.48
7.47
7.45
7.44
6.96
6.94
6.93
4.78
Bz
HO
F
F
O
N
H
Br
4t
1.03
2.00
1.05
4.01
1.00
0.95
f1 (ppm)

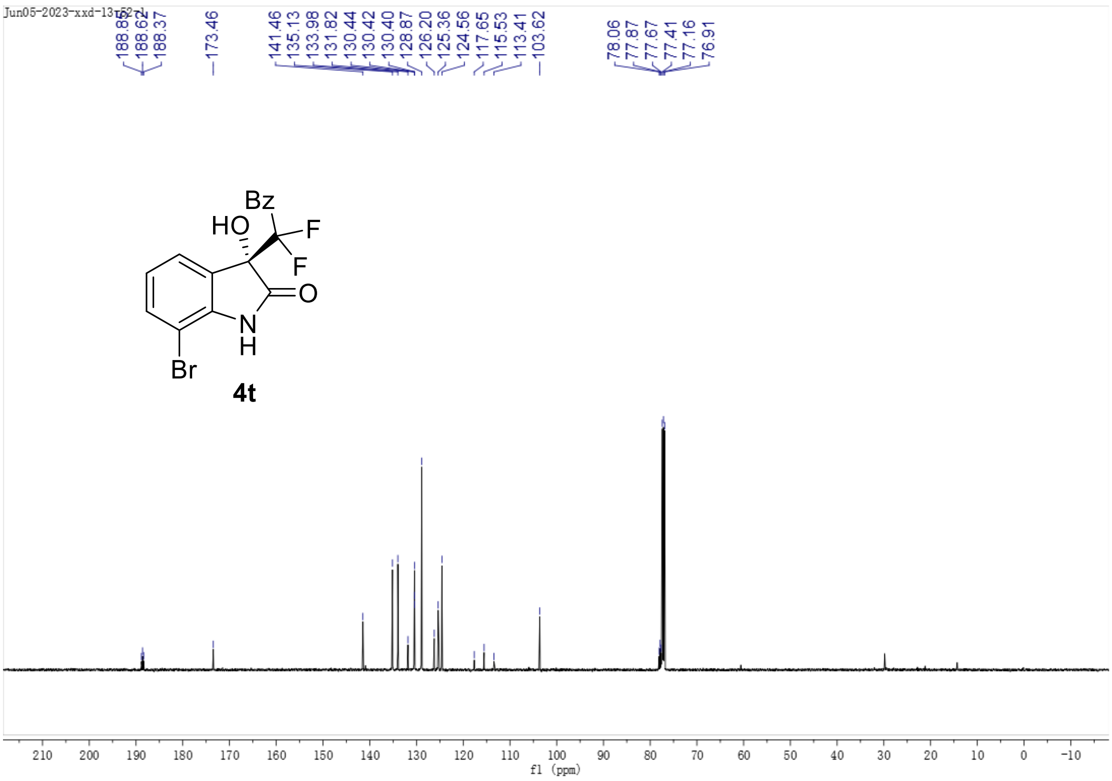
Jun05-2023-xxd-13-52-1
188.87
188.62
188.37
173.46
141.46
135.13
133.98
131.82
130.44
130.42
130.40
128.87
126.20
125.36
124.56
117.65
115.53
113.41
103.62
78.06
77.87
77.67
77.41
77.16
76.91
Bz
HO
F
F
O
N
H
Br
4t
f1 (ppm)

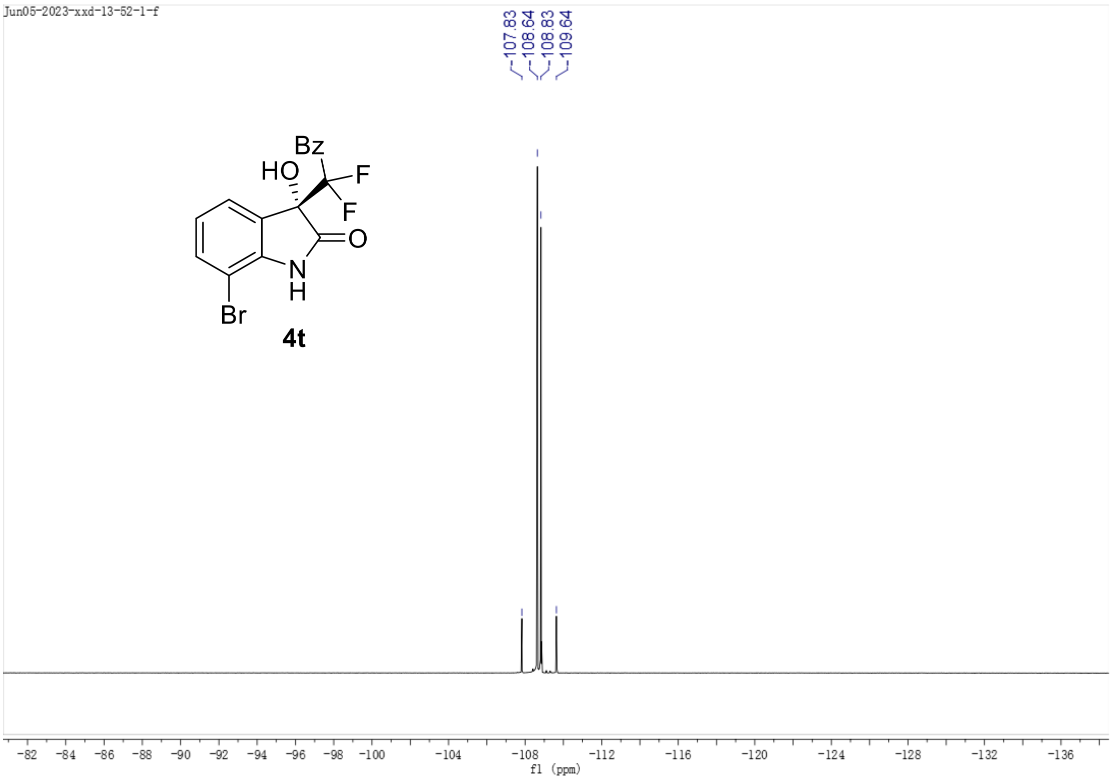
Jun05-2023-xxd-13-52-1-f
-107.83
-108.64
-108.83
-109.64
Bz
HO
F
F
O
N
H
Br
4t
f1 (ppm)

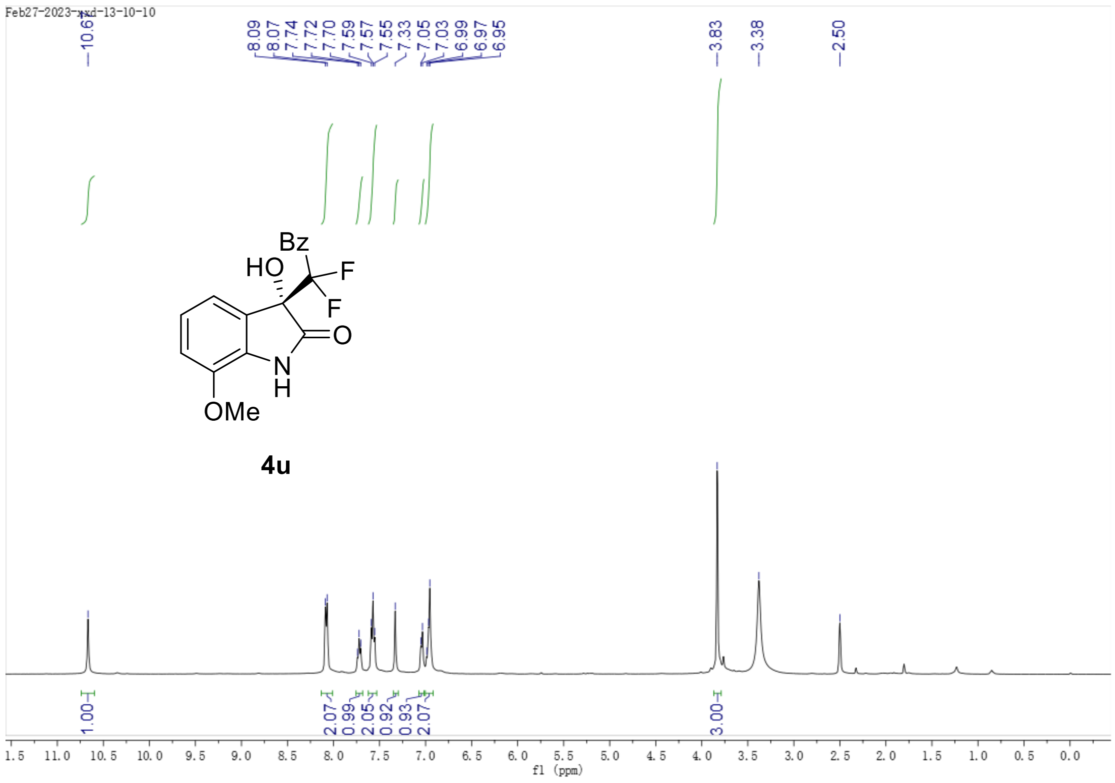

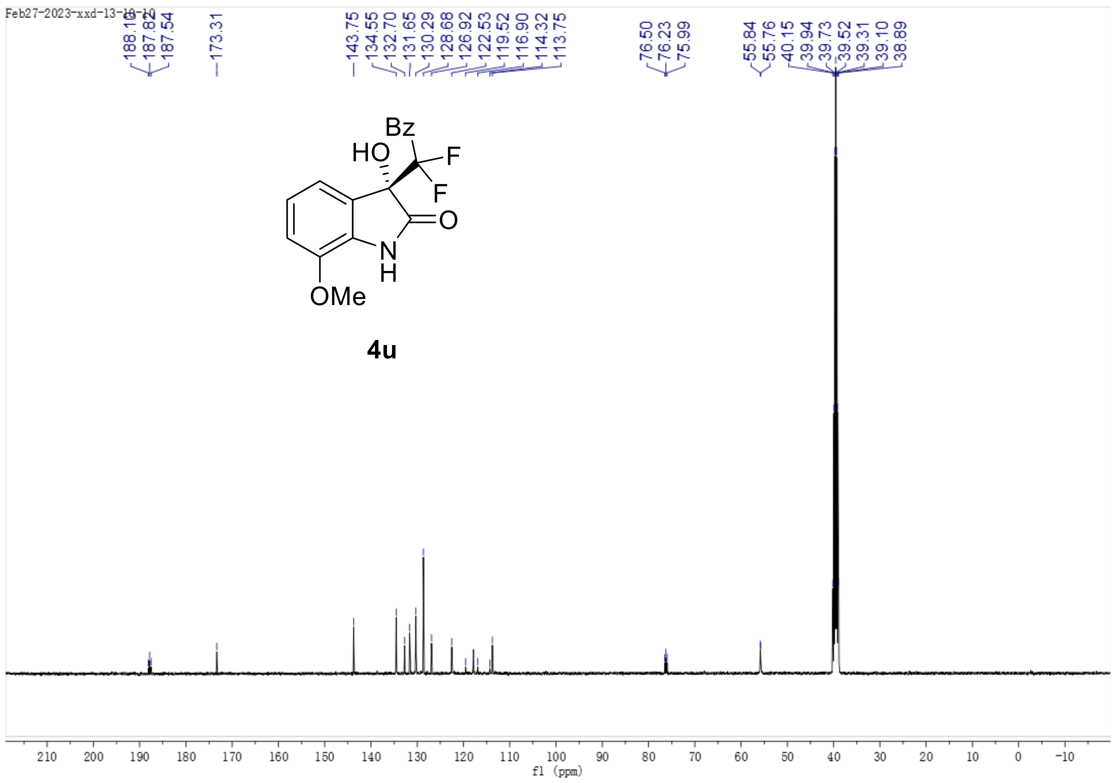

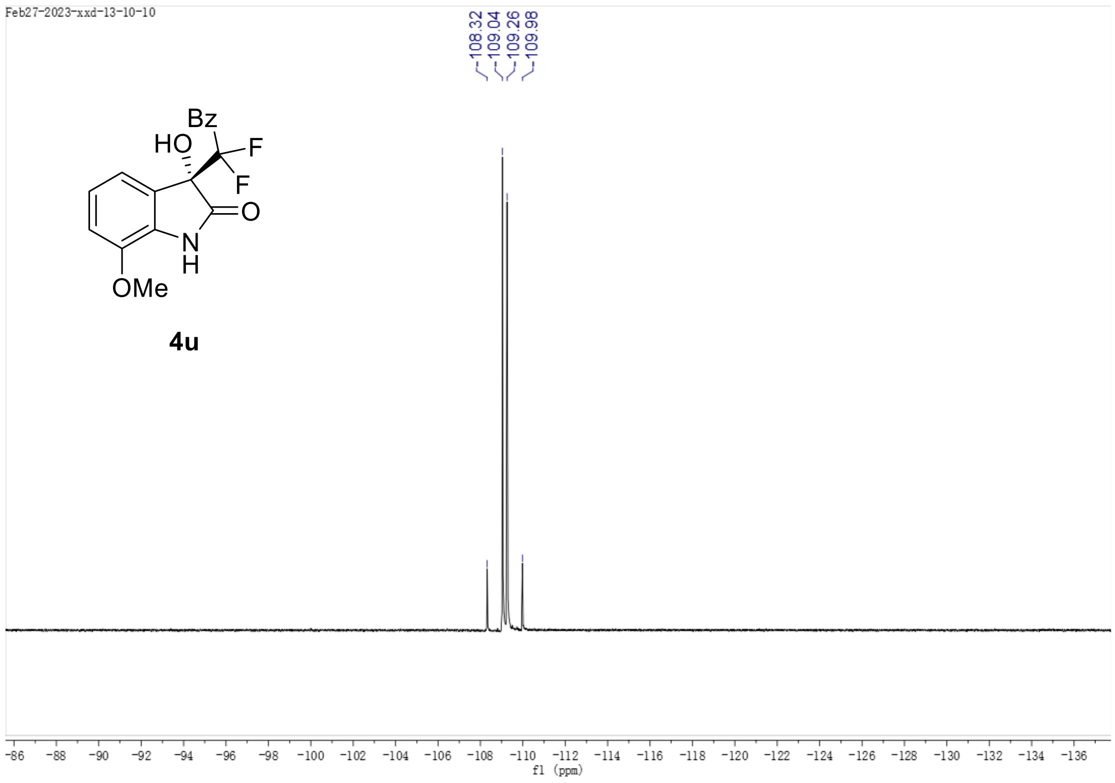

Feb27-2023-xxd-13-10-10
-108.32
-109.04
-109.26
-109.98
Bz
HO
F
F
O
N
H
OMe
4u
f1 (ppm)

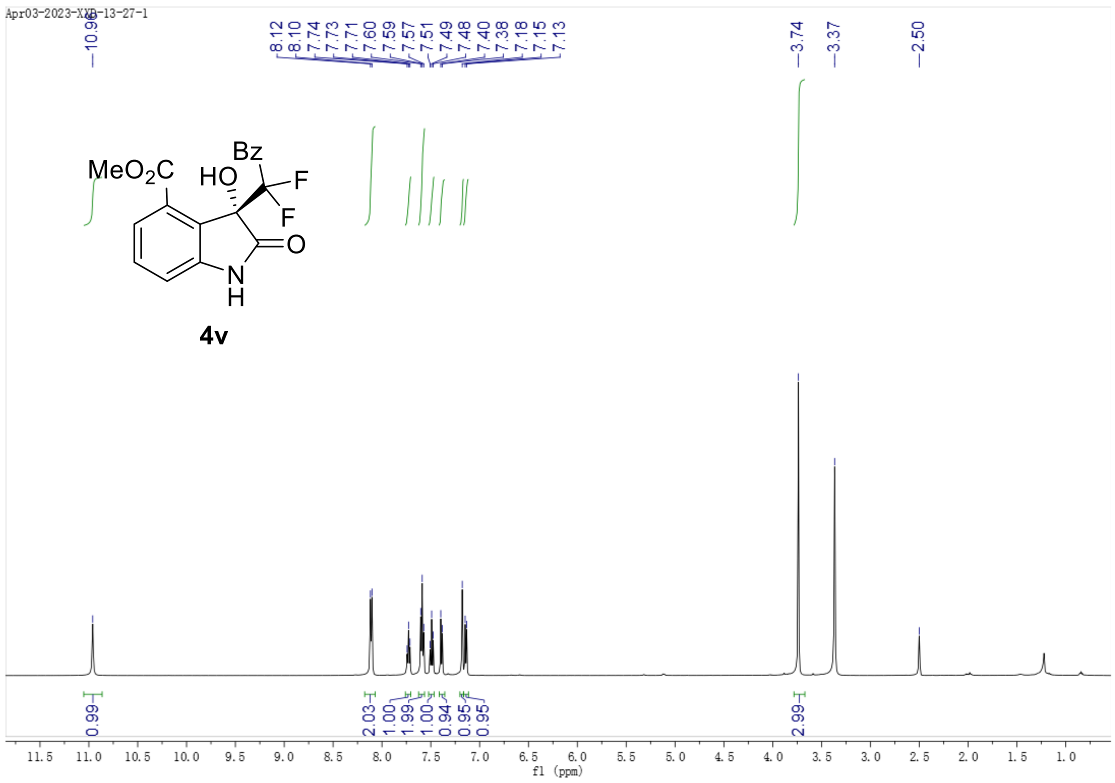

8.12
8.10
7.74
7.73
7.71
7.60
7.59
7.57
7.51
7.49
7.48
7.40
7.38
7.18
7.15
7.13
3.74
3.37
2.50
MeO2C
HO
Bz
F
F
O
N
H
4v
0.99
2.03
1.00
1.99
1.00
0.94
0.95
0.95
2.99
f1 (ppm)

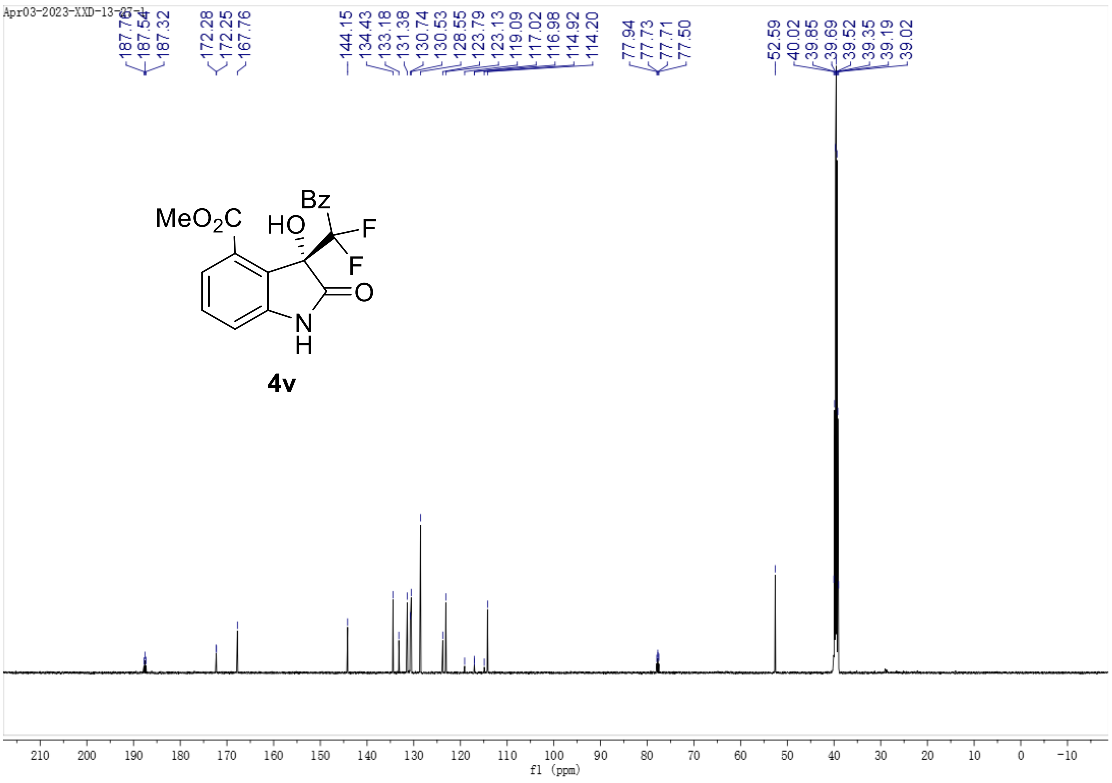
Apr03-2023-XXD-13-27-1
187.76
187.54
187.32
172.28
172.25
167.76
144.15
134.43
133.18
131.38
130.74
130.53
128.55
123.79
123.13
119.09
117.02
116.98
114.92
114.20
77.94
77.73
77.71
77.50
52.59
40.02
39.85
39.69
39.52
39.35
39.19
39.02
MeO2C
HO
Bz
F
F
O
N
H
4v
210 200 190 180 170 160 150 140 130 120 110 100 90 80 70 60 50 40 30 20 10 0 -10
f1 (ppm)

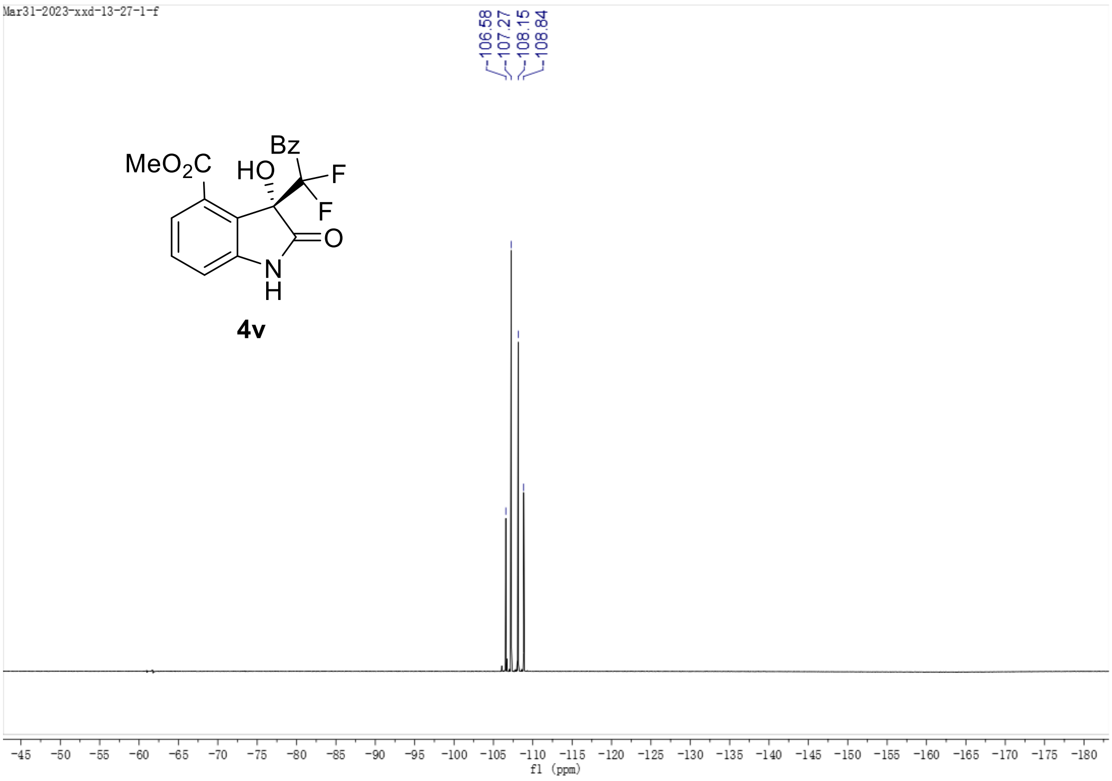
Mar31-2023-xxd-13-27-1-f
-106.58
-107.27
-108.15
-108.84
MeO2C
HO
Bz
F
F
O
N
H
4v
-45 -50 -55 -60 -65 -70 -75 -80 -85 -90 -95 -100 -105 -110 -115 -120 -125 -130 -135 -140 -145 -150 -155 -160 -165 -170 -175 -180
f1 (ppm)

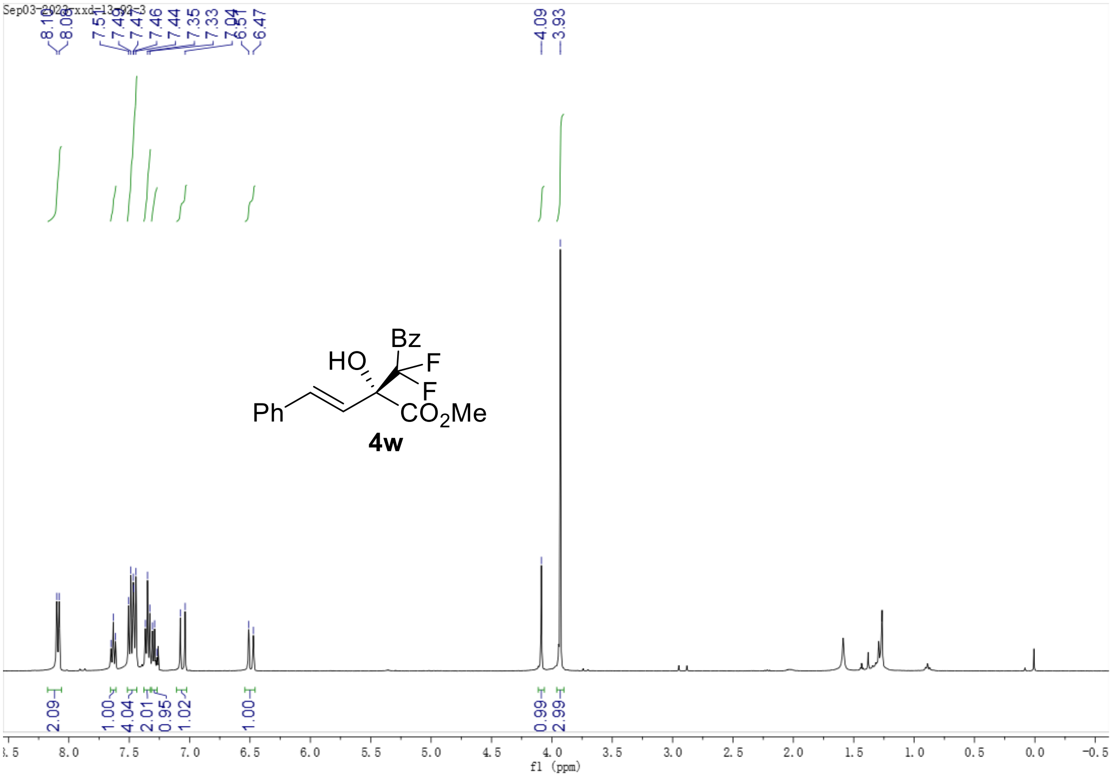

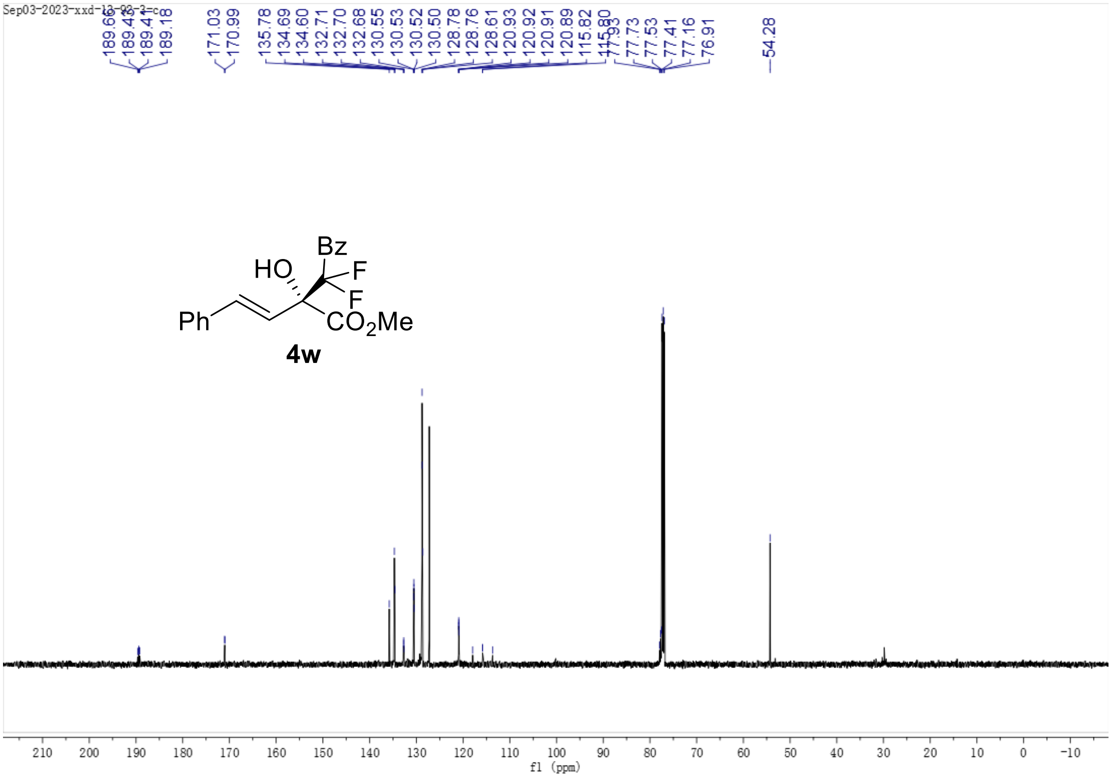

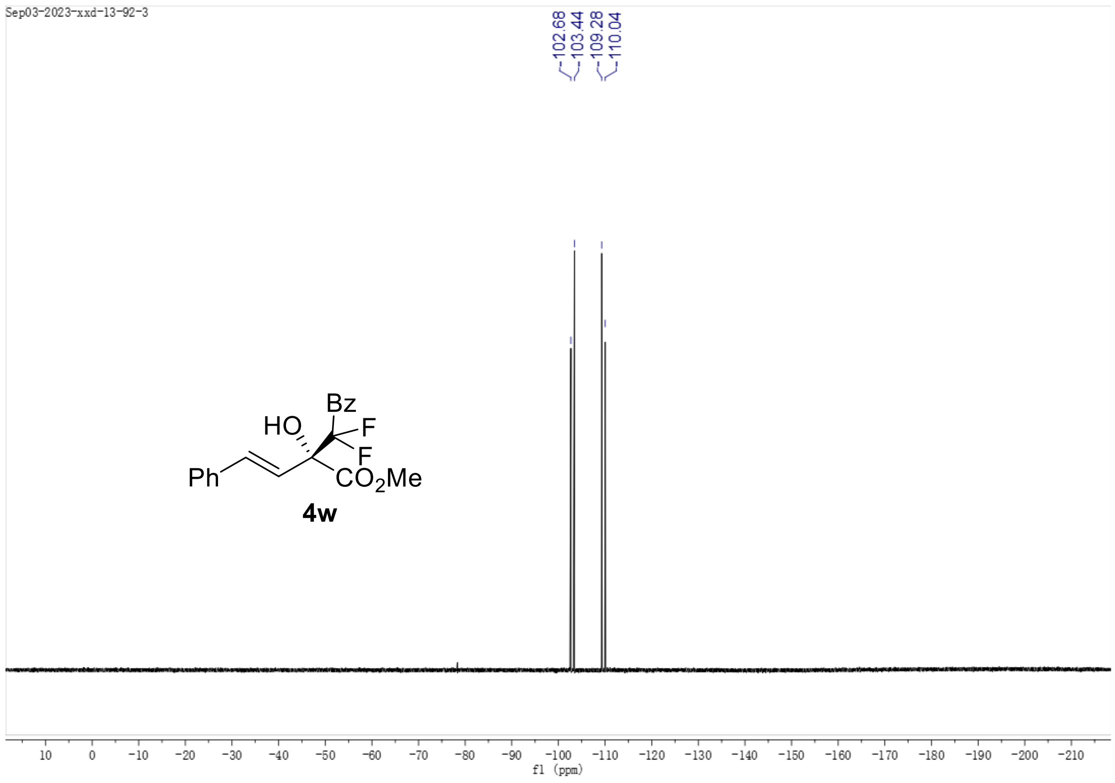
Sep03-2023-xxd-13-92-3
102.68
103.44
109.28
110.04
HO
Bz
F
F
Ph
CO2Me
4w
f1 (ppm)

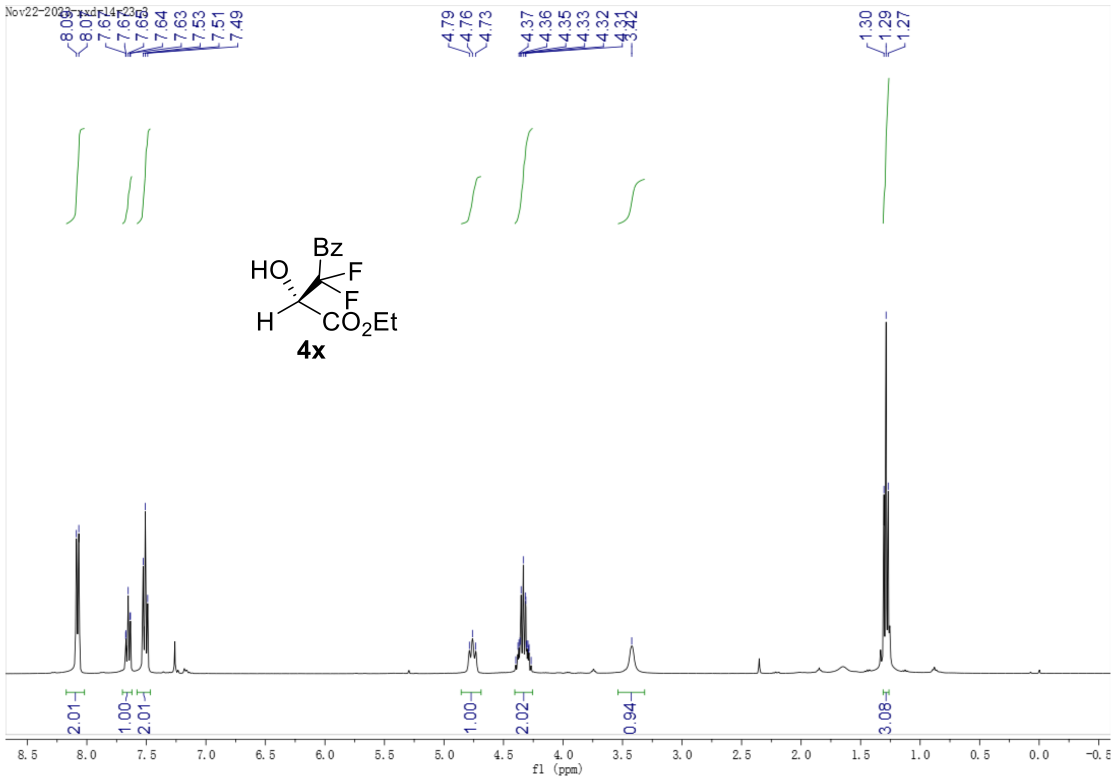
8.03
8.01
7.64
7.63
7.53
7.51
7.49
4.79
4.76
4.73
4.37
4.36
4.35
4.33
4.32
4.31
3.42
1.30
1.29
1.27
HO
Bz
F
F
H
CO2Et
4x
2.01
1.00
2.01
1.00
2.02
0.94
3.08
f1 (ppm)

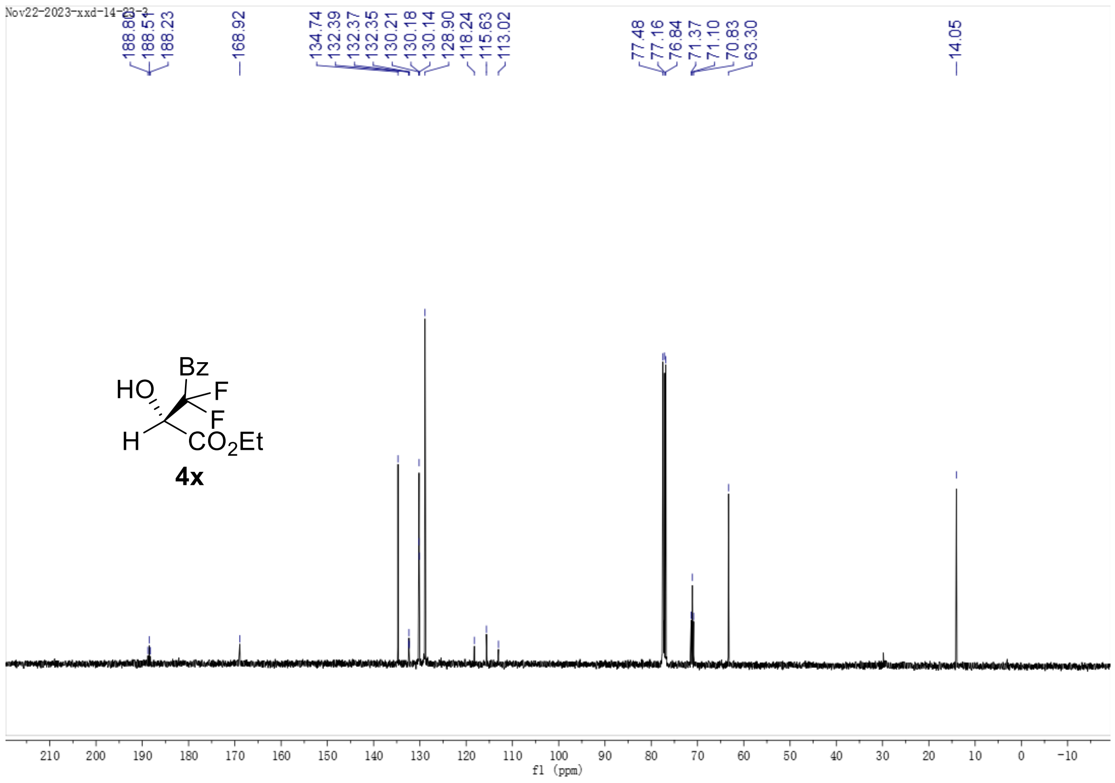
Nov22-2023-xxd-14-23-2
188.80
188.51
188.23
168.92
134.74
132.39
132.37
132.35
130.21
130.18
130.14
128.90
118.24
115.63
113.02
77.48
77.16
76.84
71.37
71.10
70.83
63.30
14.05
Bz
HO
F
F
H
CO_2Et
4x
f1 (ppm)

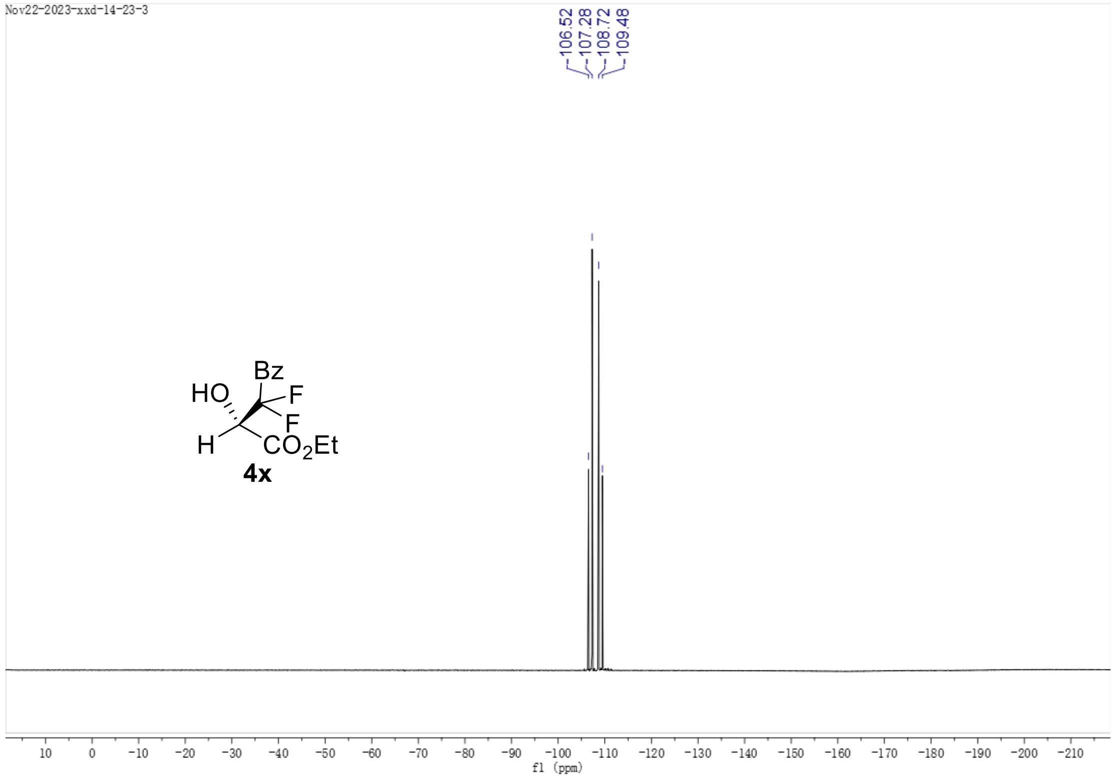
Nov22-2023-xxd-14-23-3
-106.52
-107.28
-108.72
-109.48
Bz
HO
F
F
H
CO_2Et
4x
f1 (ppm)

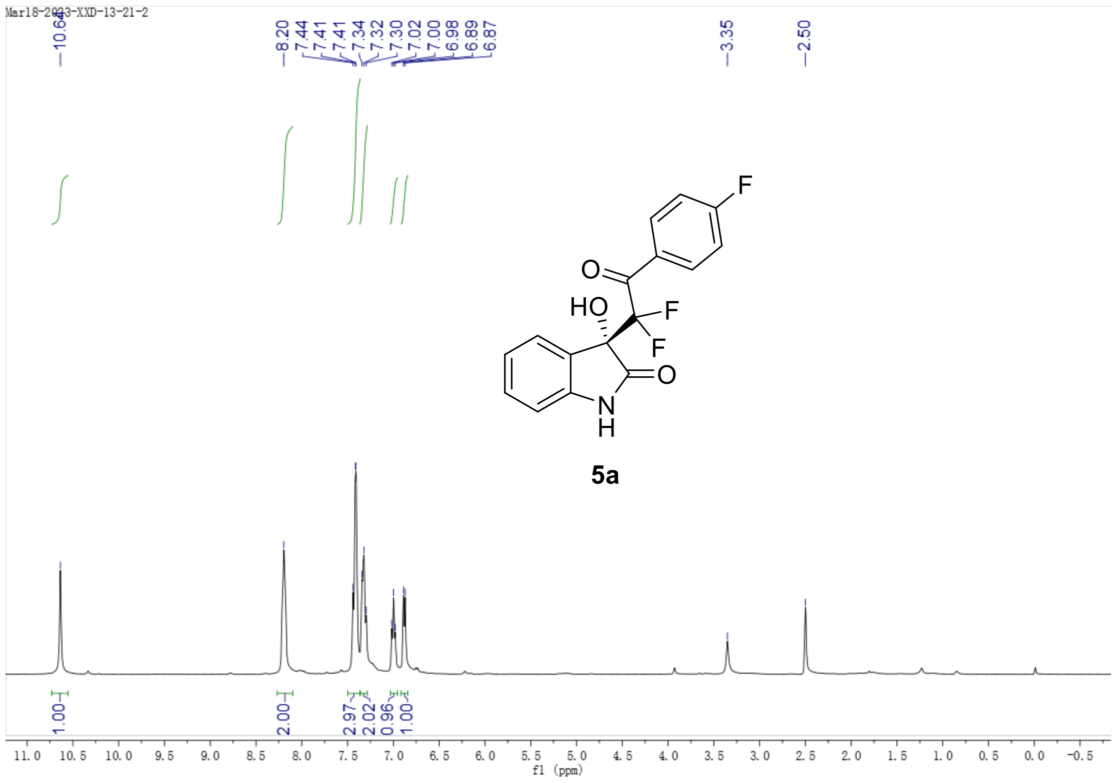
Mar18-2023-XXD-13-21-2
10.64
8.20
7.44
7.41
7.41
7.34
7.32
7.30
7.02
7.00
6.98
6.89
6.87
3.35
2.50
F
O
HO
F
F
O
N
H
5a
1.00
2.00
2.97
2.02
0.96
1.00
f1 (ppm)

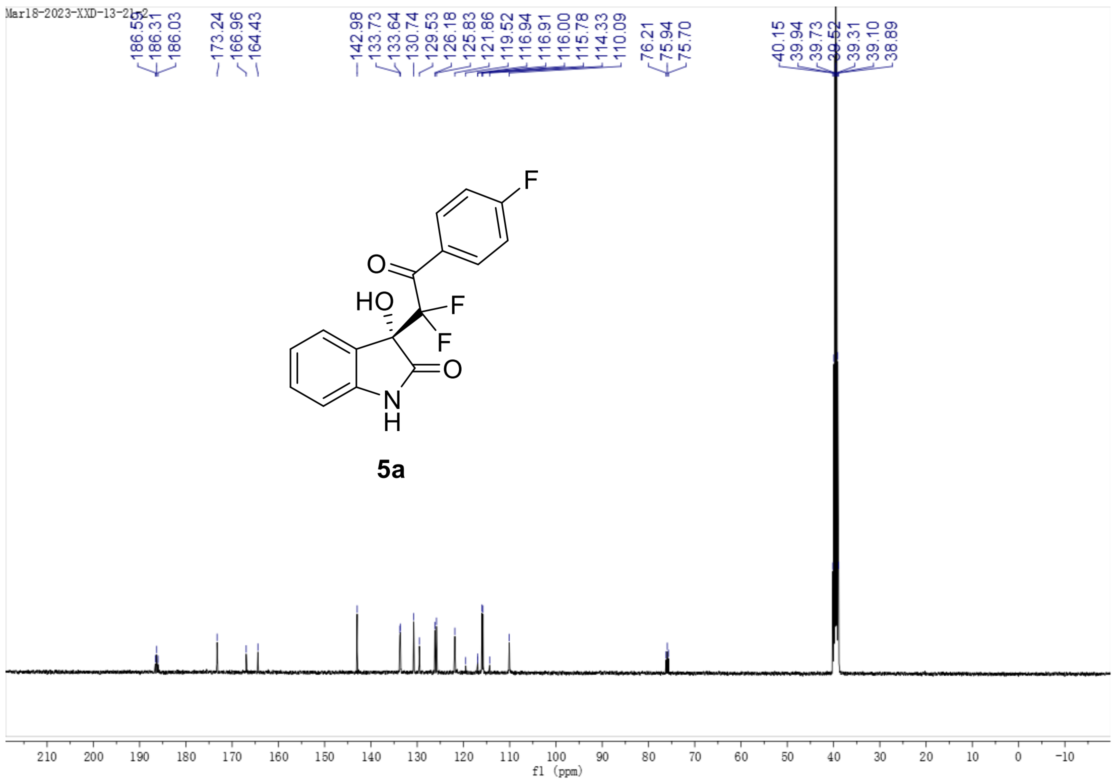
Mar18-2023-XXD-13-21-2
186.31
186.03
173.24
166.96
164.43
142.98
133.73
133.64
130.74
129.53
126.18
125.83
121.86
119.52
116.94
116.91
116.00
115.78
114.33
110.09
76.21
75.94
75.70
40.15
39.94
39.73
39.31
39.10
38.89
F
O
HO
F
F
O
N
H
5a
f1 (ppm)

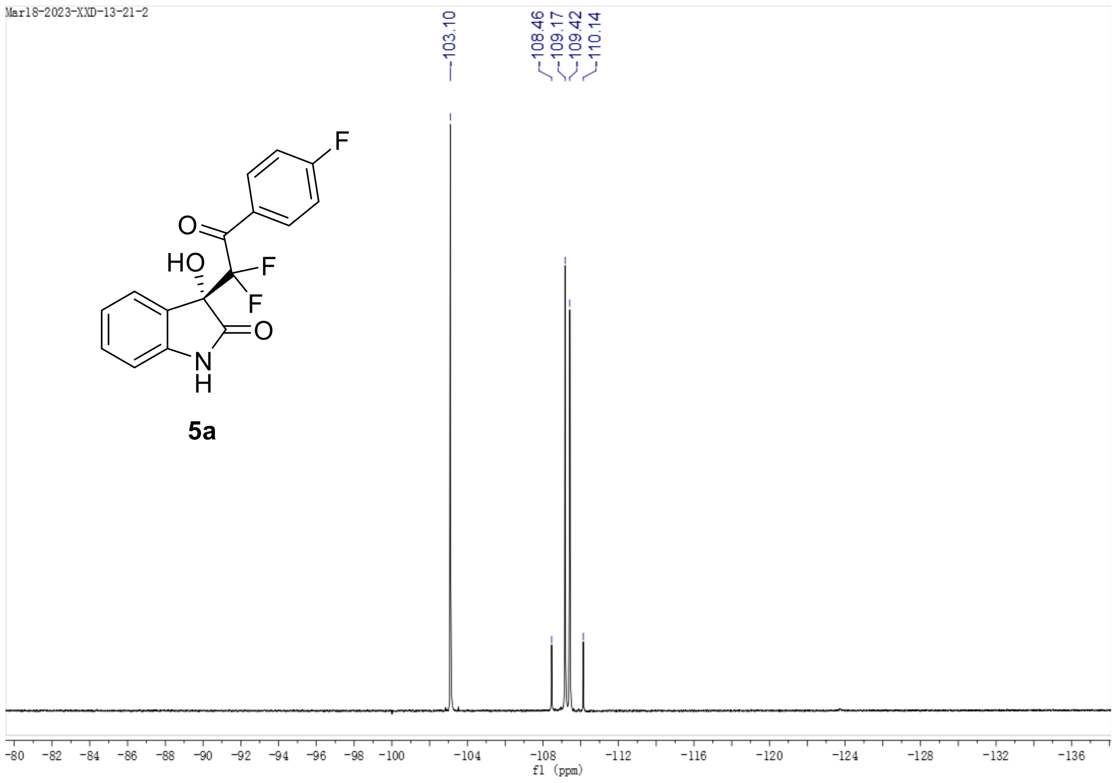
Mar18-2023-XXD-13-21-2
-103.10
-108.46
-109.17
-109.42
-110.14
F
O
HO
F
F
O
N
H
5a
-80 -82 -84 -86 -88 -90 -92 -94 -96 -98 -100 -104 -108 -112 -116 -120 -124 -128 -132 -136
f1 (ppm)

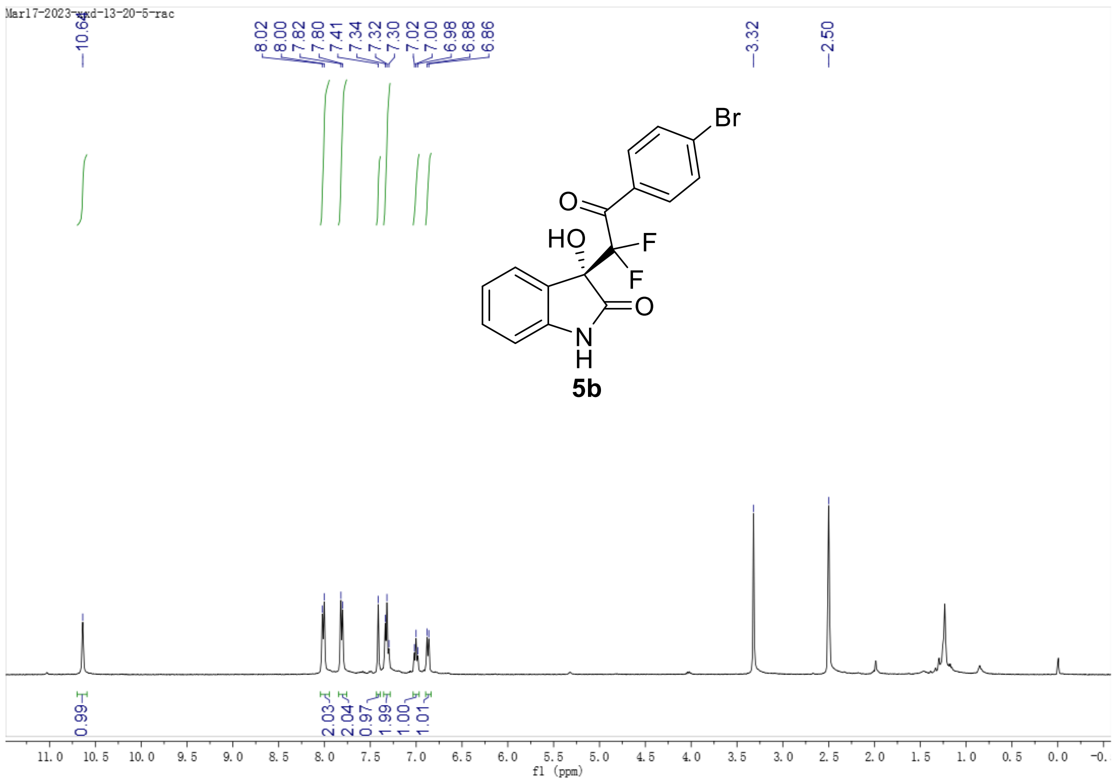
Mar17-2023-xxd-13-20-5-rac
10.64
8.02
8.00
7.82
7.80
7.41
7.34
7.32
7.30
7.02
7.00
6.98
6.88
6.86
3.32
2.50
Br
O
HO
F
F
O
N
H
5b
0.99
2.03
2.04
0.97
1.99
1.00
1.01
11.0 10.5 10.0 9.5 9.0 8.5 8.0 7.5 7.0 6.5 6.0 5.5 5.0 4.5 4.0 3.5 3.0 2.5 2.0 1.5 1.0 0.5 0.0 -0.
f1 (ppm)

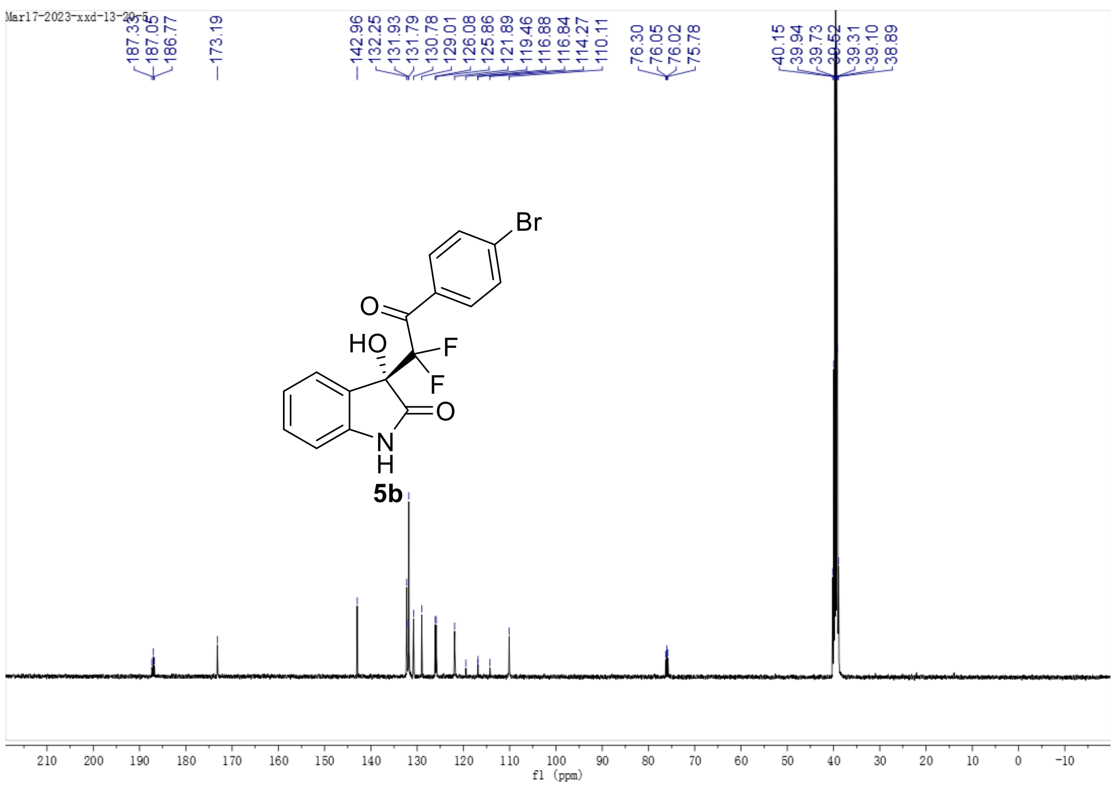
Mar17-2023-xxd-13-20-5
187.33
187.05
186.77
173.19
142.96
132.25
131.93
131.79
130.78
129.01
126.08
125.86
121.89
119.46
116.88
116.84
114.27
110.11
76.30
76.05
76.02
75.78
40.15
39.94
39.73
39.52
39.31
39.10
38.89
Br
O
HO
F
F
O
N
H
5b
210 200 190 180 170 160 150 140 130 120 110 100 90 80 70 60 50 40 30 20 10 0 -10
f1 (ppm)

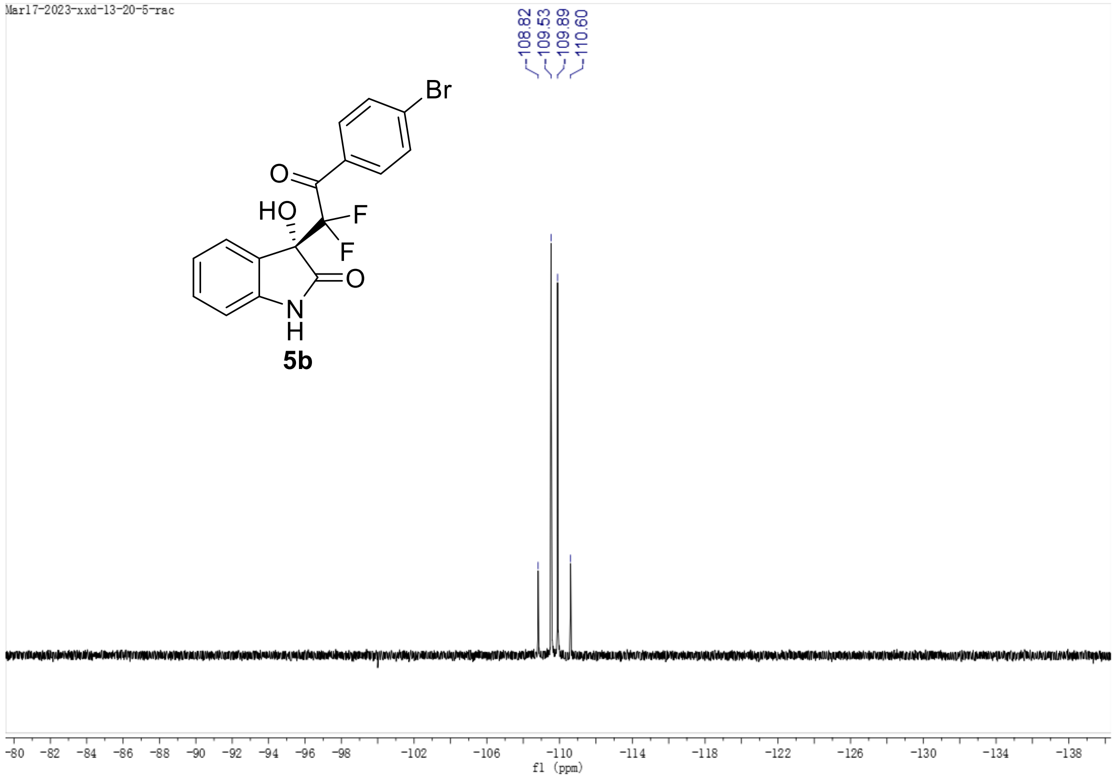
Mar17-2023-xxd-13-20-5-rac
-108.82
-109.53
-109.89
-110.60
Br
O
HO
F
F
O
N
H
5b
-80 -82 -84 -86 -88 -90 -92 -94 -96 -98 -102 -106 -110 -114 -118 -122 -126 -130 -134 -138
f1 (ppm)

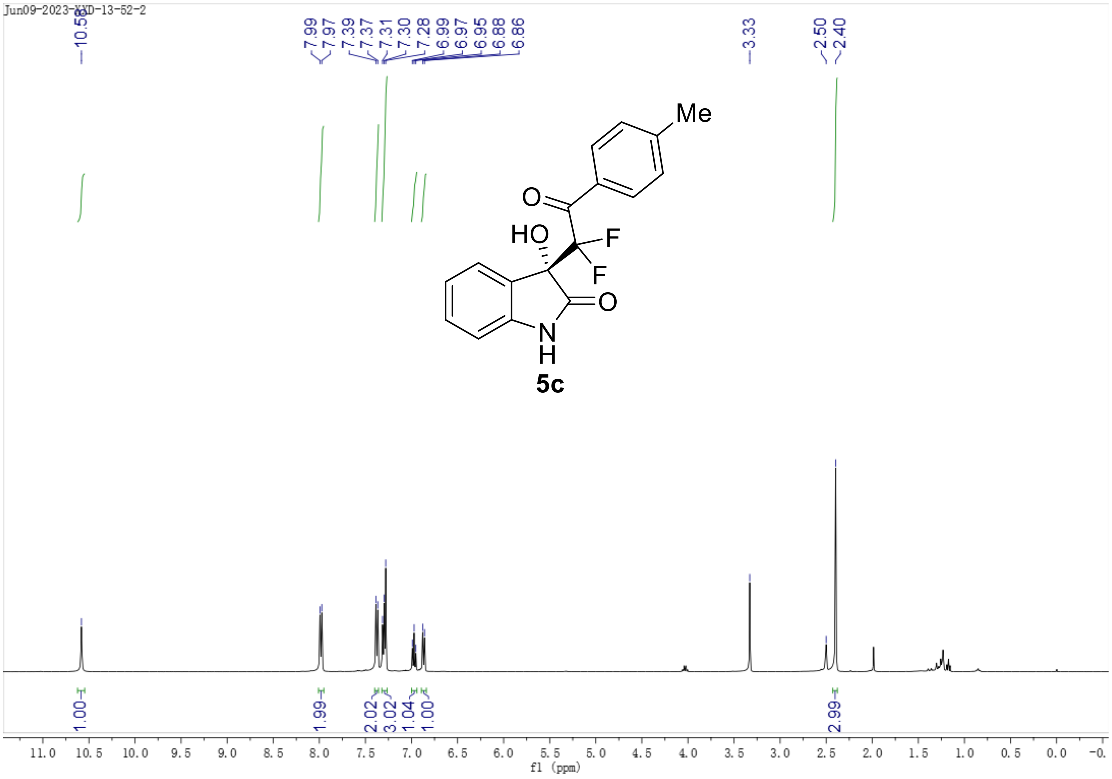
Jun09-2023-XXD-13-52-2
5c
fl (ppm)

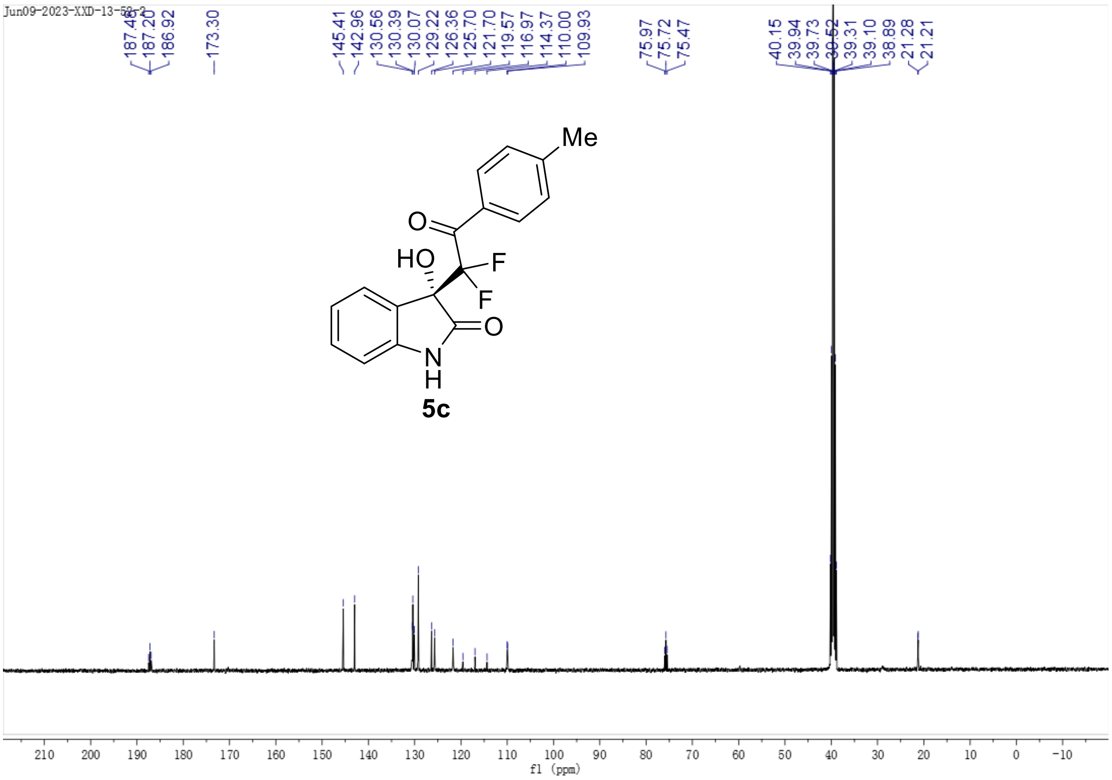
Jun09-2023-XXD-13-52-2
5c
fl (ppm)

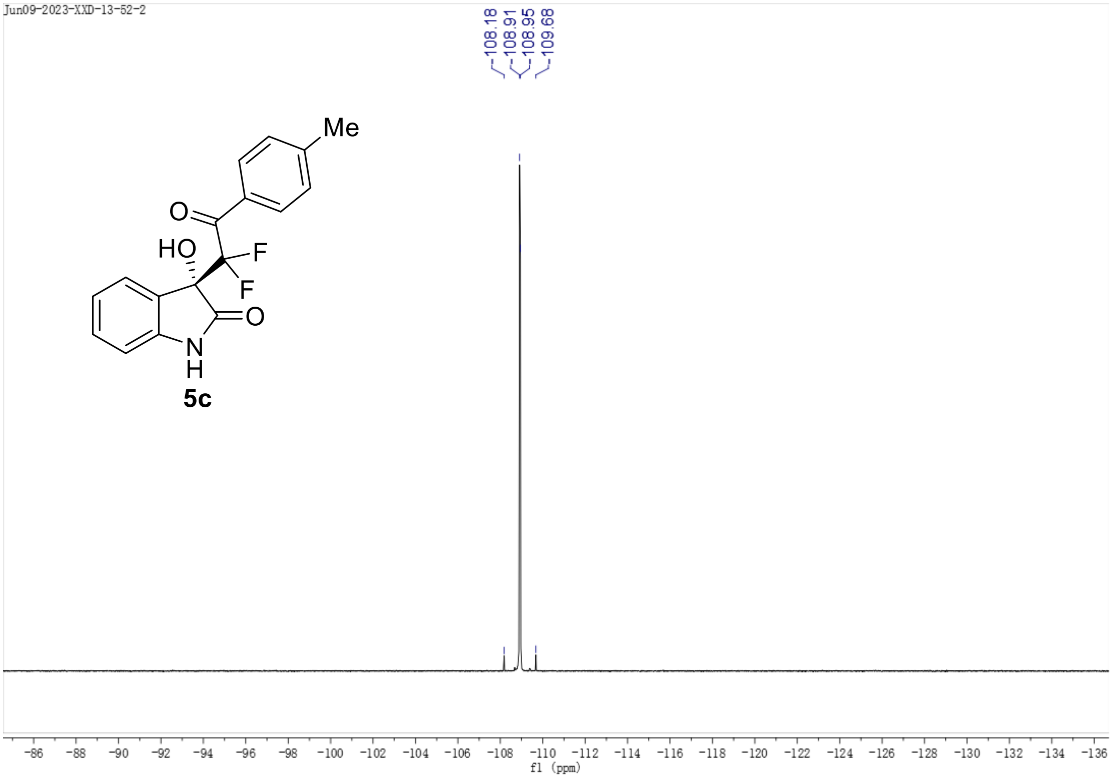
Jun09-2023-XXD-13-52-2
-108.18
-108.91
-108.95
-109.68
Me
O
HO
F
F
O
N
H
5c
f1 (ppm)

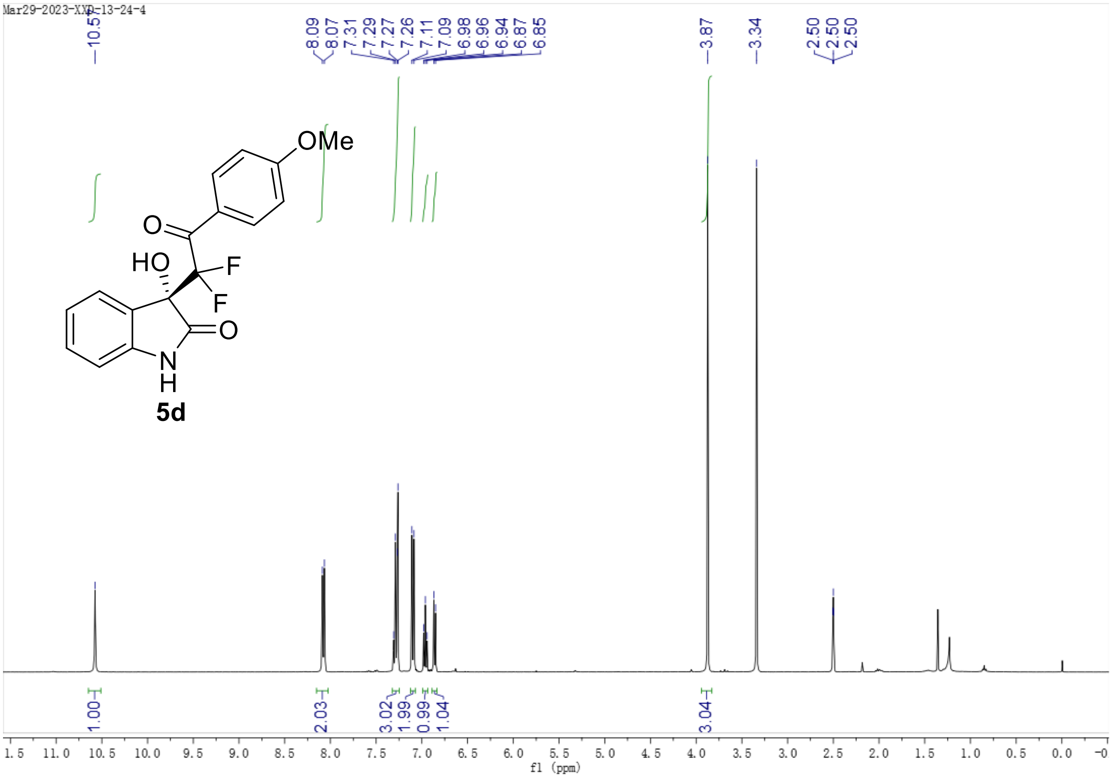
Mar29-2023-XXD-13-24-4
-10.57
8.09
8.07
7.31
7.29
7.27
7.26
7.11
7.09
6.98
6.96
6.94
6.87
6.85
-3.87
-3.34
2.50
2.50
2.50
OMe
O
HO
F
F
O
N
H
5d
1.00
2.03
3.02
1.99
0.99
1.04
3.04
f1 (ppm)

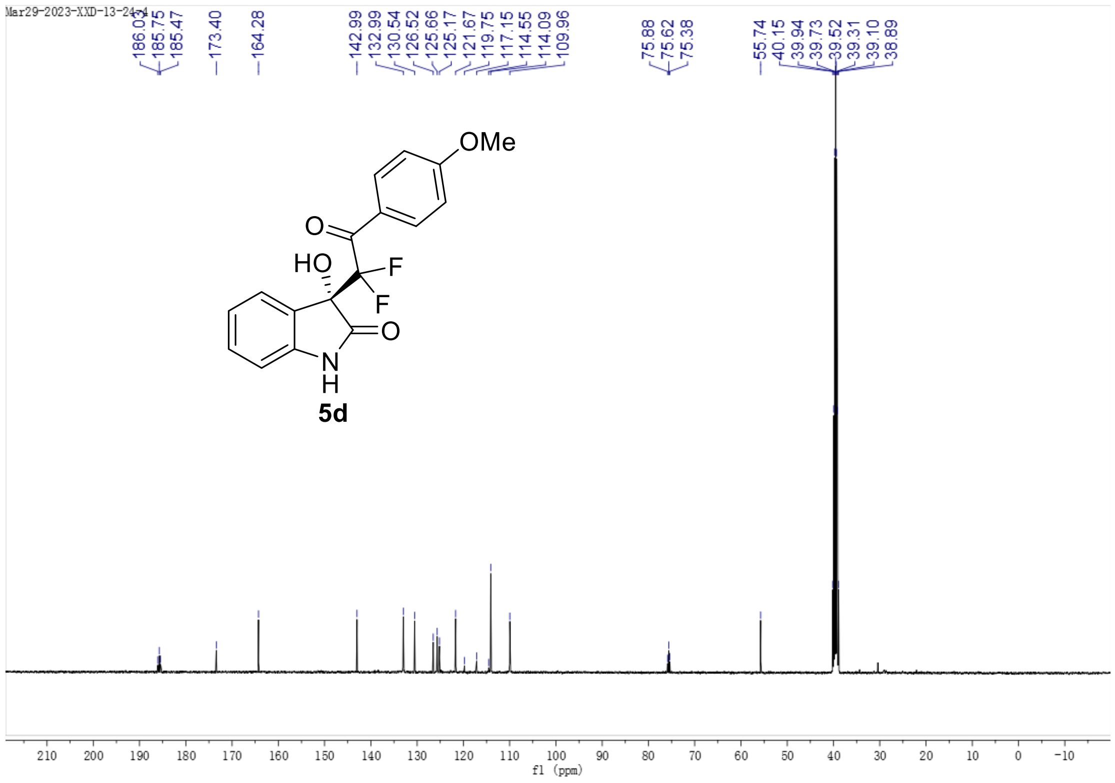
Mar29-2023-XXD-13-24-4
186.03
185.75
185.47
173.40
164.28
142.99
132.99
130.54
126.52
125.66
125.17
121.67
119.75
117.15
114.55
114.09
109.96
75.88
75.62
75.38
55.74
40.15
39.94
39.73
39.52
39.31
39.10
38.89
OMe
O
HO
F
F
O
N
H
5d
210 200 190 180 170 160 150 140 130 120 110 100 90 80 70 60 50 40 30 20 10 0 -10
f1 (ppm)

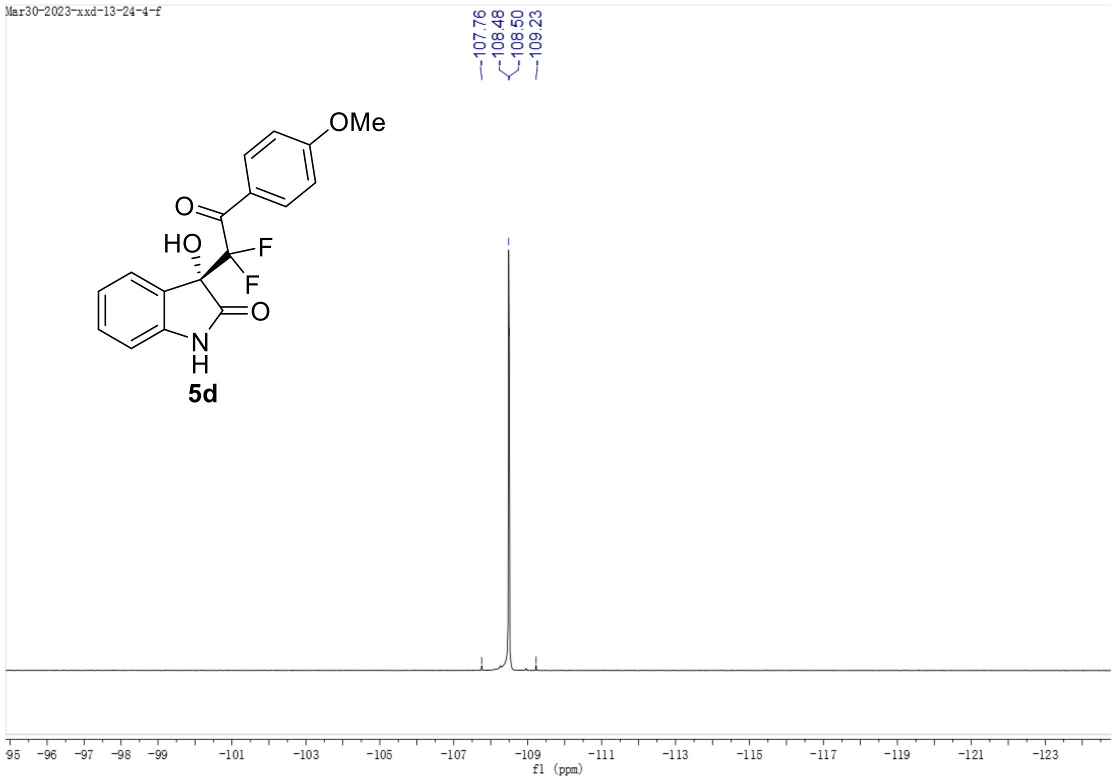
Mar30-2023-xxd-13-24-4-f
-107.76
-108.48
-108.50
-109.23
OMe
O
HO
F
F
O
N
H
5d
-95 -96 -97 -98 -99 -101 -103 -105 -107 -109 -111 -113 -115 -117 -119 -121 -123
f1 (ppm)

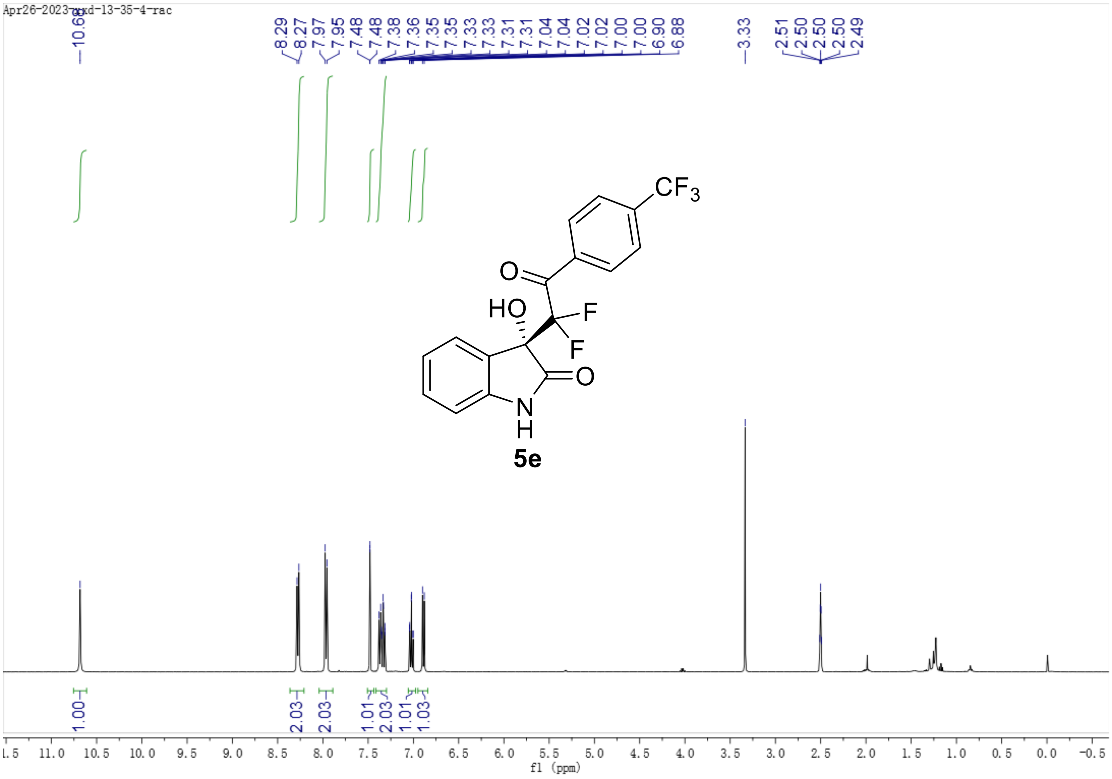
Apr26-2023-xxd-13-35-4-rac
10.69
8.29
8.27
7.97
7.95
7.48
7.48
7.38
7.36
7.35
7.35
7.33
7.33
7.31
7.31
7.04
7.04
7.02
7.02
7.00
7.00
6.90
6.88
3.33
2.51
2.50
2.50
2.50
2.49
CF3
O
HO
F
F
O
N
H
5e
1.00
2.03
2.03
1.01
2.03
1.01
1.03
f1 (ppm)

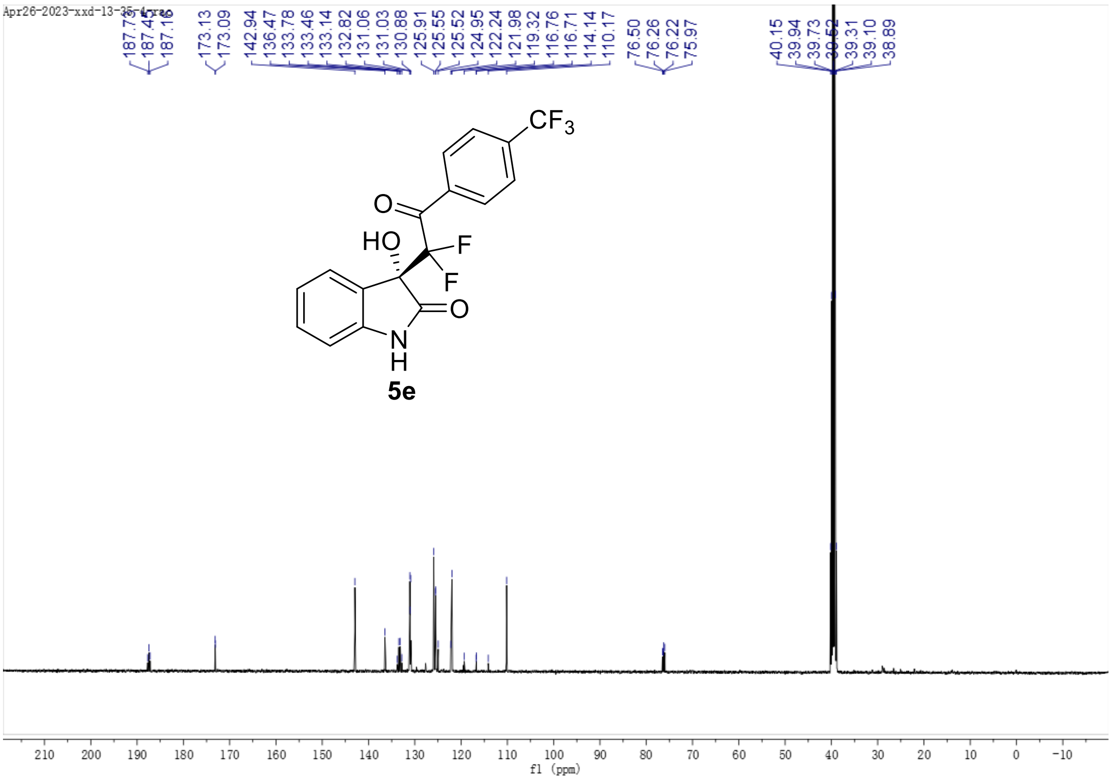
Apr26-2023-xxd-13-35-4-rac
173.13
173.09
142.94
136.47
133.78
133.46
133.14
132.82
131.06
131.03
130.88
125.91
125.55
125.52
124.95
122.24
121.98
119.32
116.76
116.71
114.14
110.17
76.50
76.26
76.22
75.97
40.15
39.94
39.73
39.31
39.10
38.89
CF3
O
HO
F
F
O
N
H
5e
f1 (ppm)

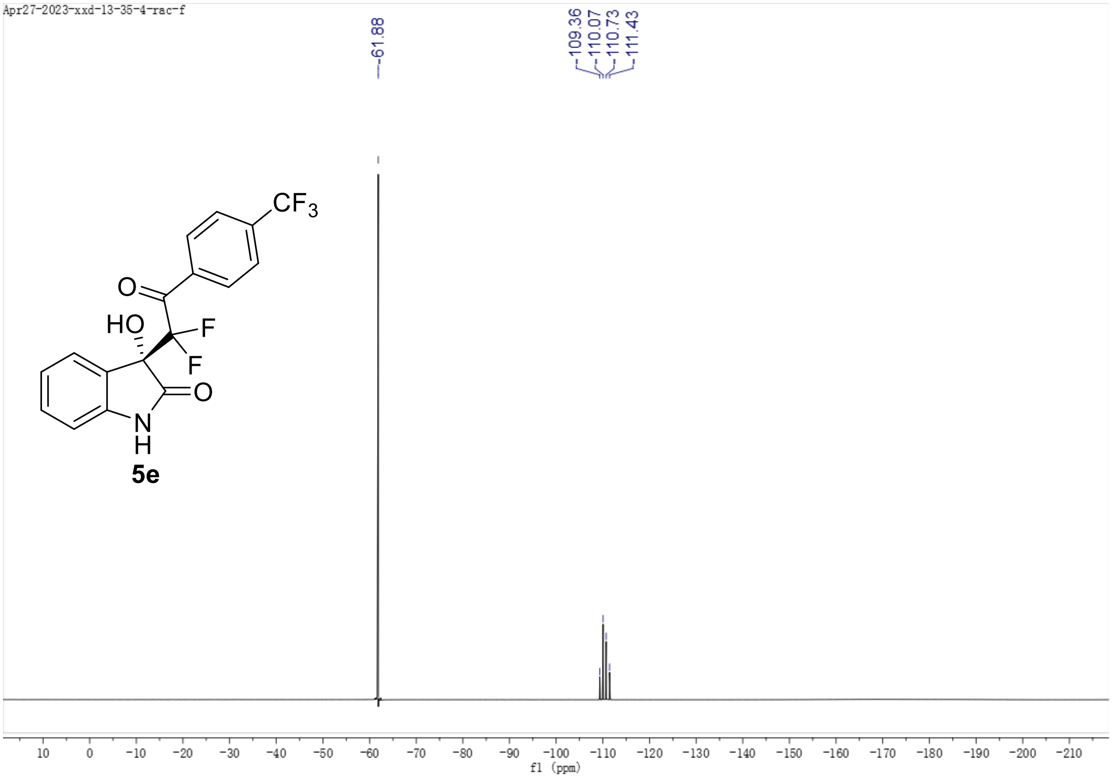
Apr27-2023-xxd-13-35-4-rac-f
-61.88
-109.36
-110.07
-110.73
-111.43
CF3
O
HO
F
F
O
N
H
5e
10 0 −10 −20 −30 −40 −50 −60 −70 −80 −90 −100 −110 −120 −130 −140 −150 −160 −170 −180 −190 −200 −210
f1 (ppm)

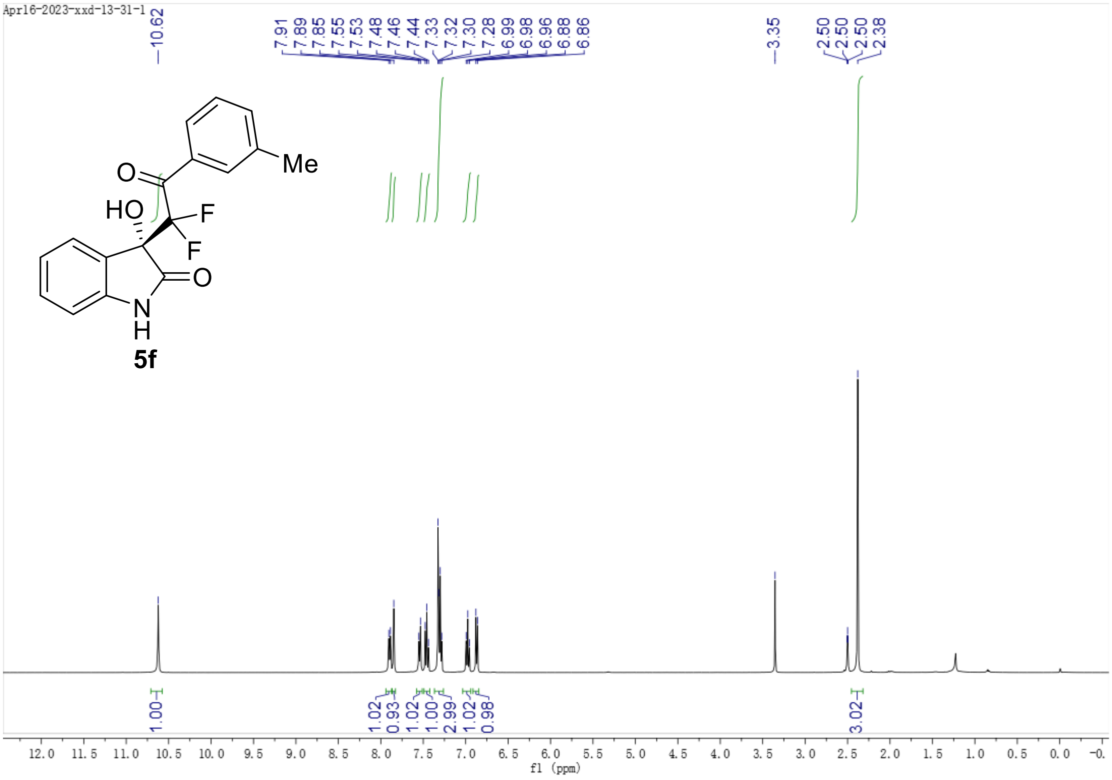
Apr16-2023-xxd-13-31-1
10.62
7.91
7.89
7.85
7.55
7.53
7.48
7.46
7.44
7.33
7.32
7.30
7.28
6.99
6.98
6.96
6.88
6.86
3.35
2.50
2.50
2.50
2.38
O
Me
HO
F
F
O
N
H
5f
1.00
1.02
0.93
1.02
1.00
2.99
1.02
0.98
3.02
12.0 11.5 11.0 10.5 10.0 9.5 9.0 8.5 8.0 7.5 7.0 6.5 6.0 5.5 5.0 4.5 4.0 3.5 3.0 2.5 2.0 1.5 1.0 0.5 0.0 -0.
f1 (ppm)

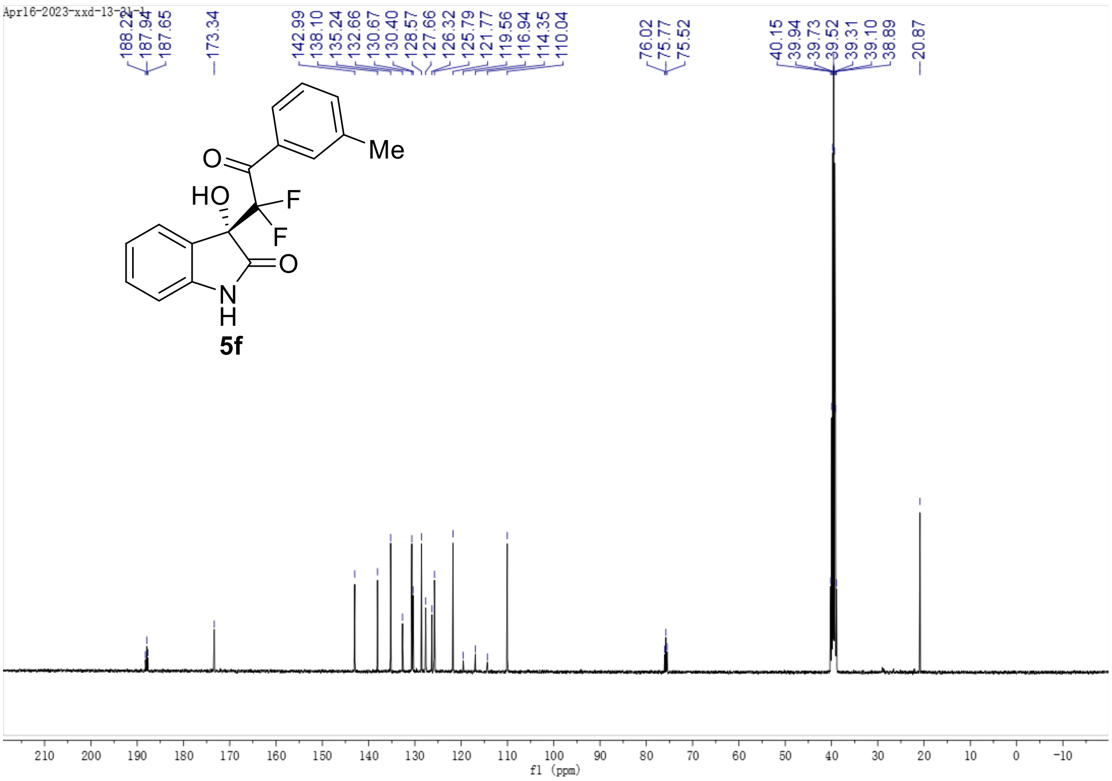
Apr16-2023-xxd-13-31-1
188.22
187.94
187.65
173.34
142.99
138.10
135.24
132.66
130.67
130.40
128.57
127.66
126.32
125.79
121.77
119.56
116.94
114.35
110.04
76.02
75.77
75.52
40.15
39.94
39.73
39.52
39.31
39.10
38.89
20.87
Me
O
HO
F
F
O
N
H
5f
f1 (ppm)

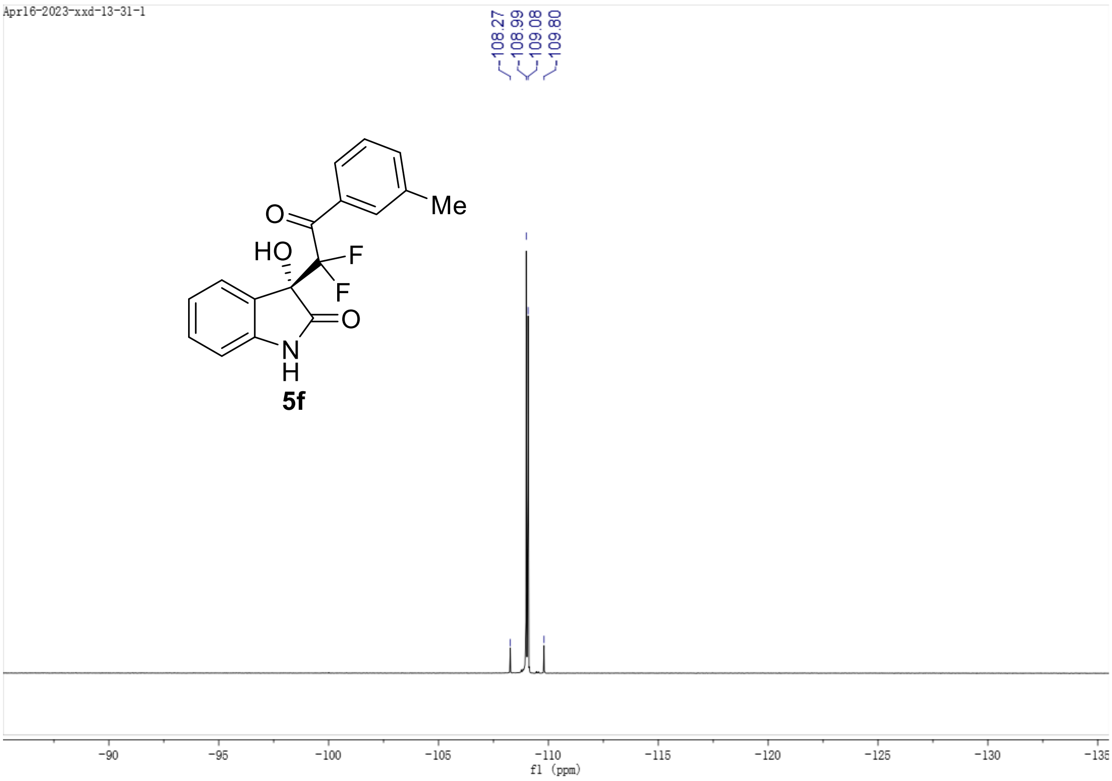
Apr16-2023-xxd-13-31-1
-108.27
-108.99
-109.08
-109.80
Me
O
HO
F
F
O
N
H
5f
f1 (ppm)

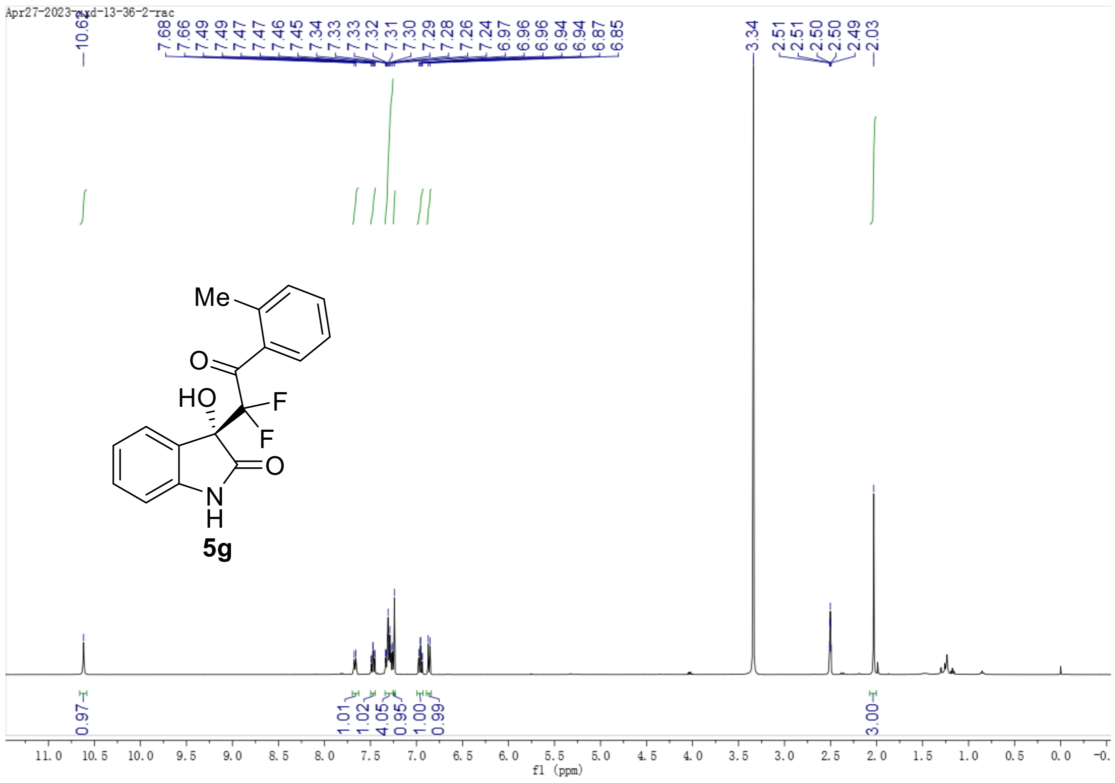

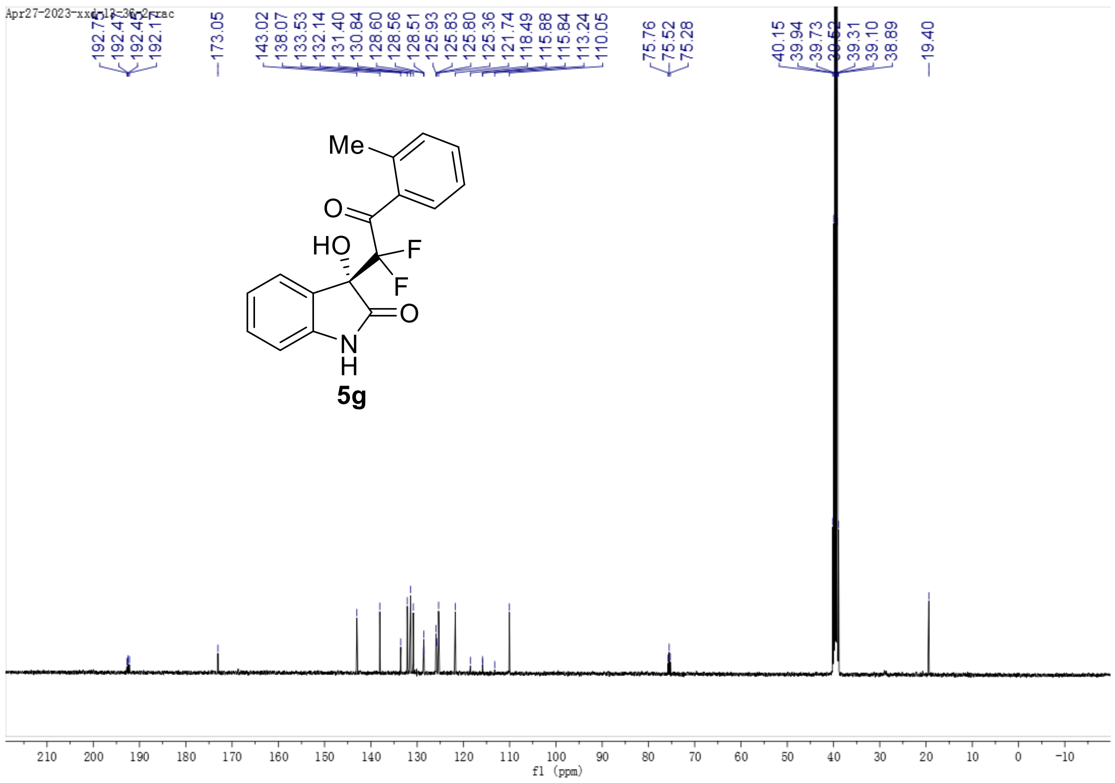

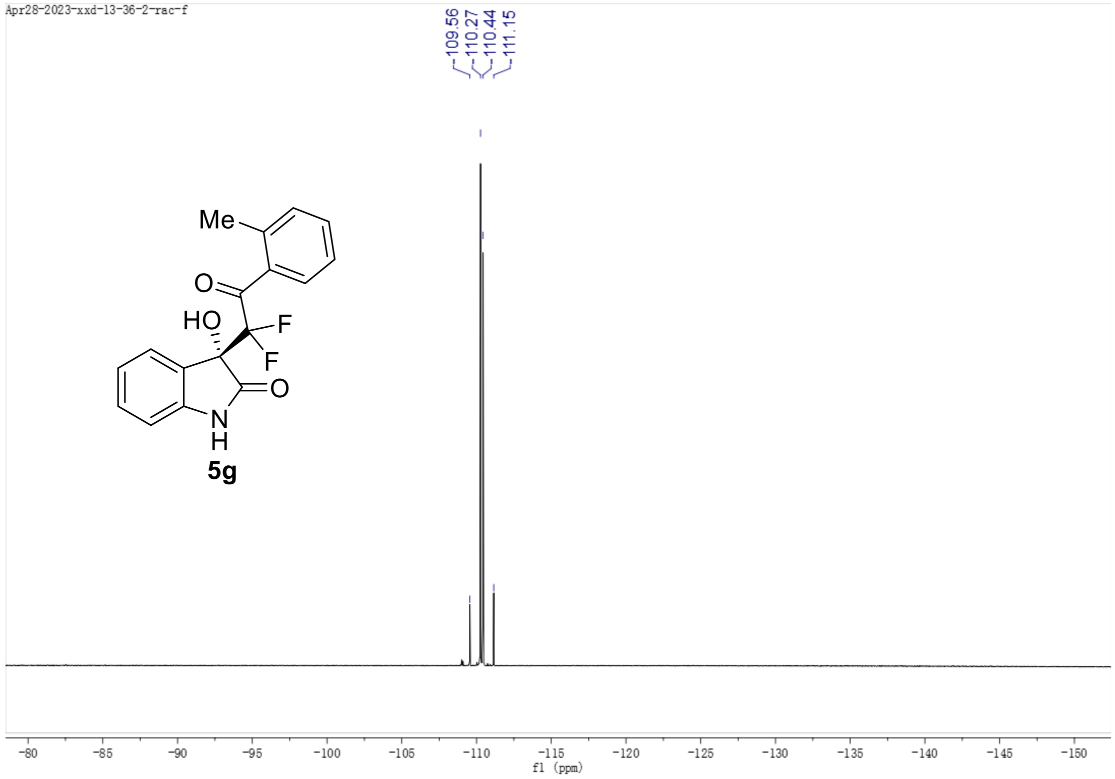
Apr28-2023-xxd-13-36-2-rac-f
-109.56
-110.27
-110.44
-111.15
Me
O
HO
F
F
O
N
H
5g
f1 (ppm)

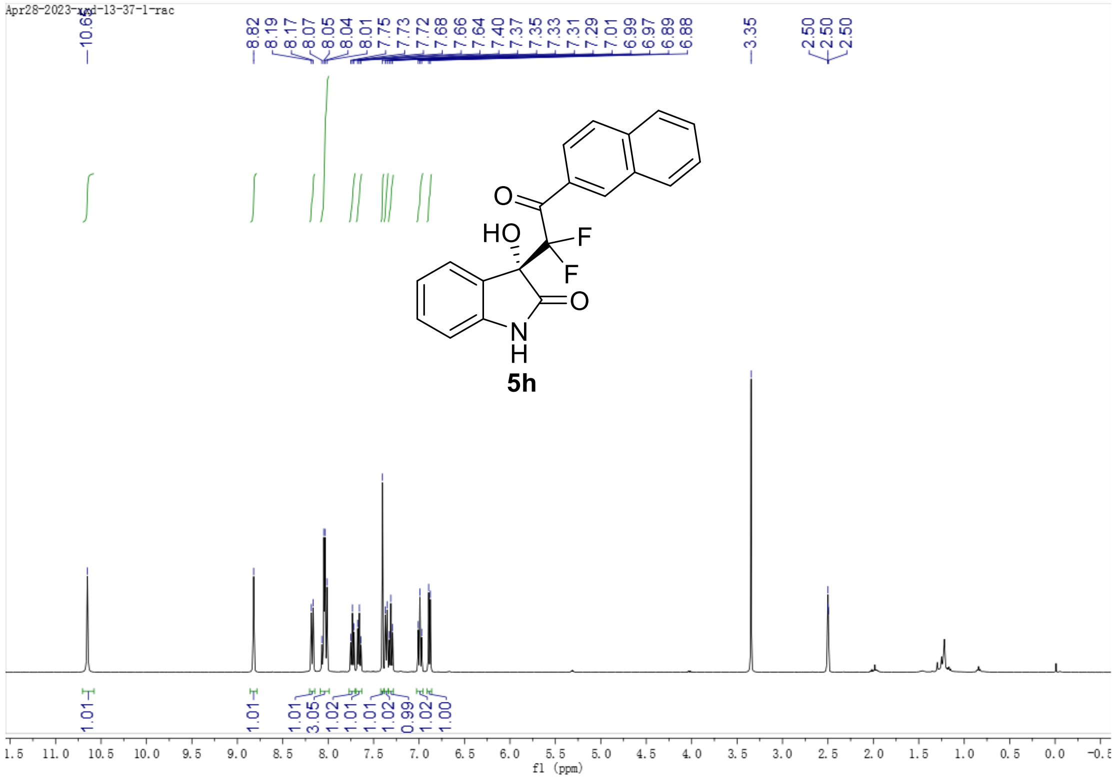
Apr28-2023-xxd-13-37-1-rac
10.65
8.82
8.19
8.17
8.07
8.05
8.04
8.01
7.75
7.73
7.72
7.68
7.66
7.64
7.40
7.37
7.35
7.33
7.31
7.29
7.01
6.99
6.97
6.89
6.88
3.35
2.50
2.50
2.50
O
HO
F
F
O
N
H
5h
1.01
1.01
1.01
3.05
1.02
1.01
1.01
1.02
0.99
1.02
1.00
f1 (ppm)

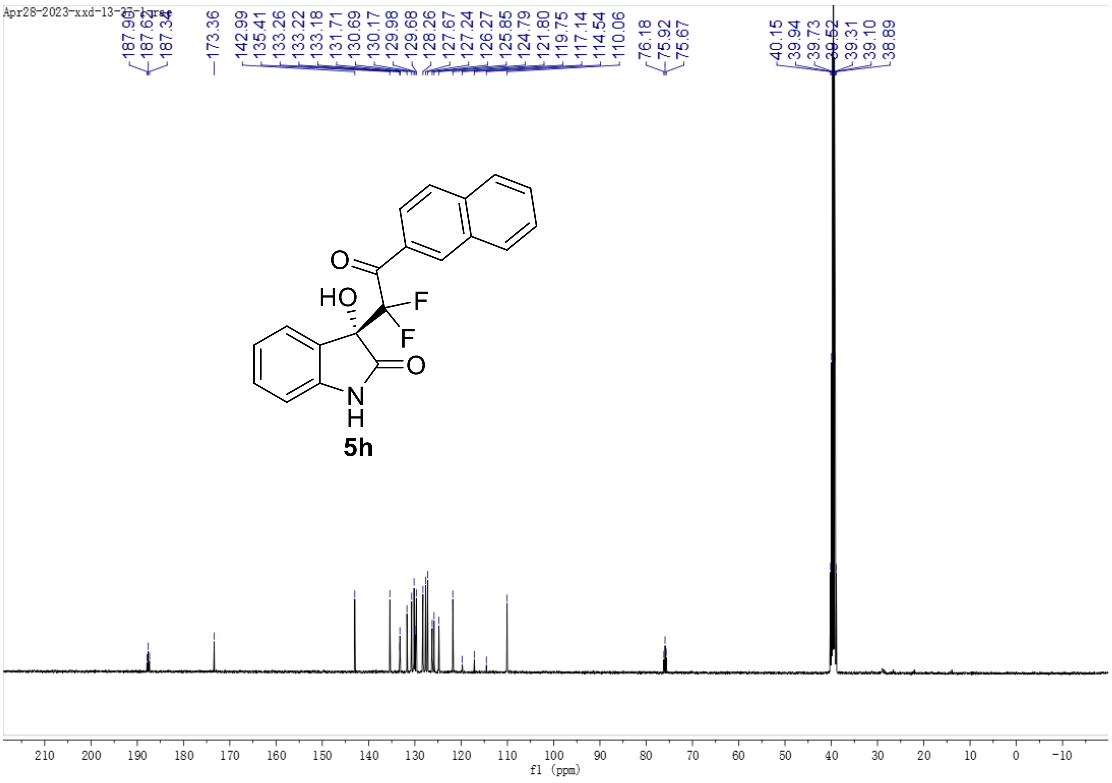
Apr28-2023-xxd-13-37-1-rac
187.90
187.62
187.34
173.36
142.99
135.41
133.26
133.22
133.18
131.71
130.69
130.17
129.98
129.68
128.26
127.67
127.24
126.27
125.85
124.79
121.80
119.75
117.14
114.54
110.06
76.18
75.92
75.67
40.15
39.94
39.73
39.52
39.31
39.10
38.89
O
HO
F
F
O
N
H
5h
210 200 190 180 170 160 150 140 130 120 110 100 90 80 70 60 50 40 30 20 10 0 -10
f1 (ppm)

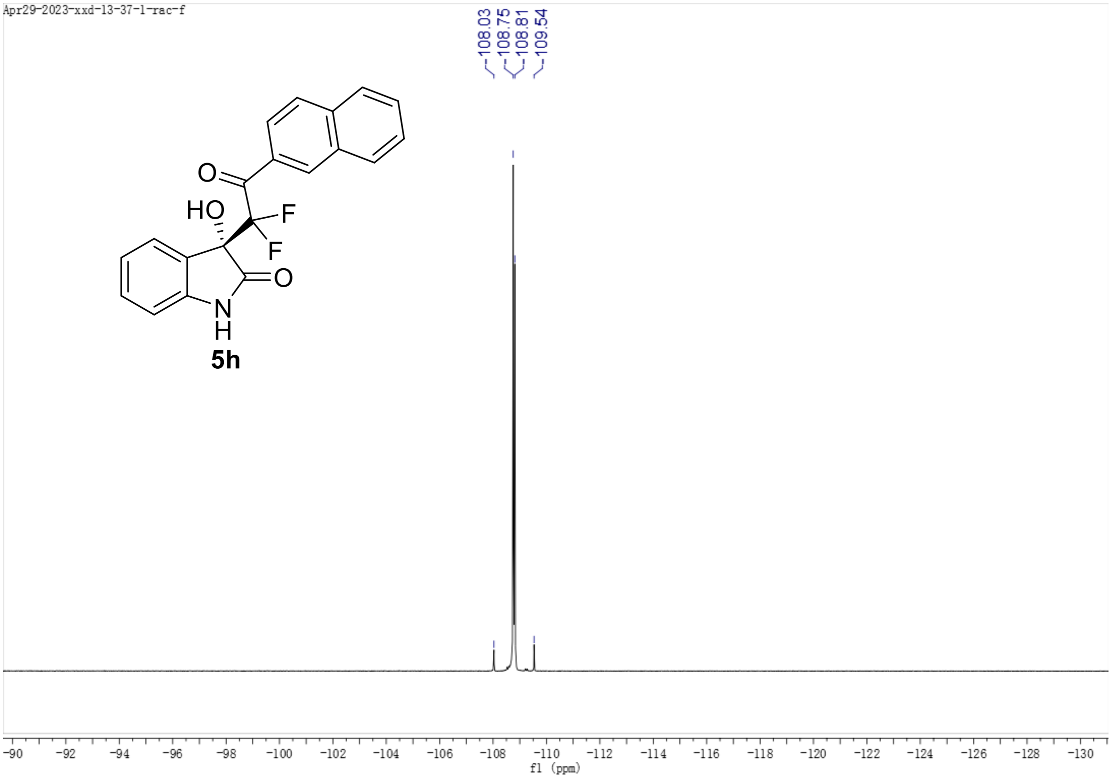
Apr29-2023-xxd-13-37-1-rac-f
-108.03
-108.75
-108.81
-109.54
O
HO
F
F
O
N
H
5h
-90 -92 -94 -96 -98 -100 -102 -104 -106 -108 -110 -112 -114 -116 -118 -120 -122 -124 -126 -128 -130
f1 (ppm)

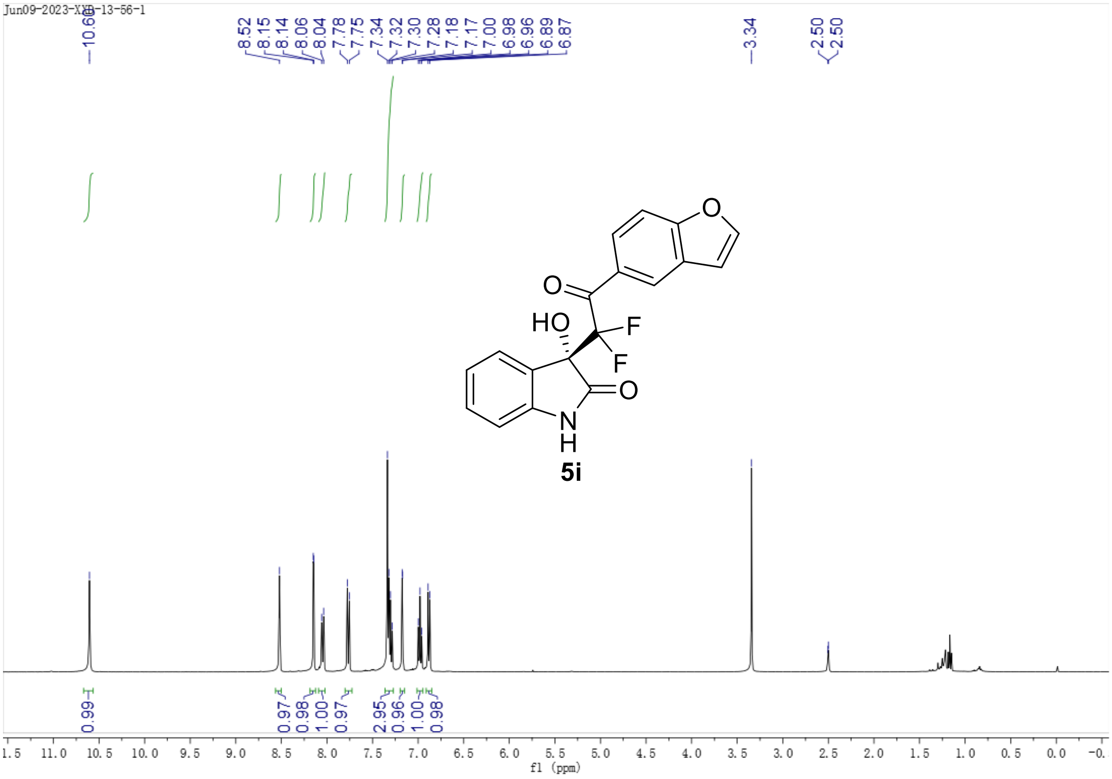
Jun09-2023-XXD-13-56-1
10.60
8.52
8.15
8.14
8.06
8.04
7.78
7.75
7.34
7.32
7.30
7.28
7.18
7.17
7.00
6.98
6.96
6.89
6.87
3.34
2.50
2.50
HO
F
F
O
O
O
N
H
5i
0.99
0.97
0.98
1.00
0.97
2.95
0.96
1.00
0.98
f1 (ppm)

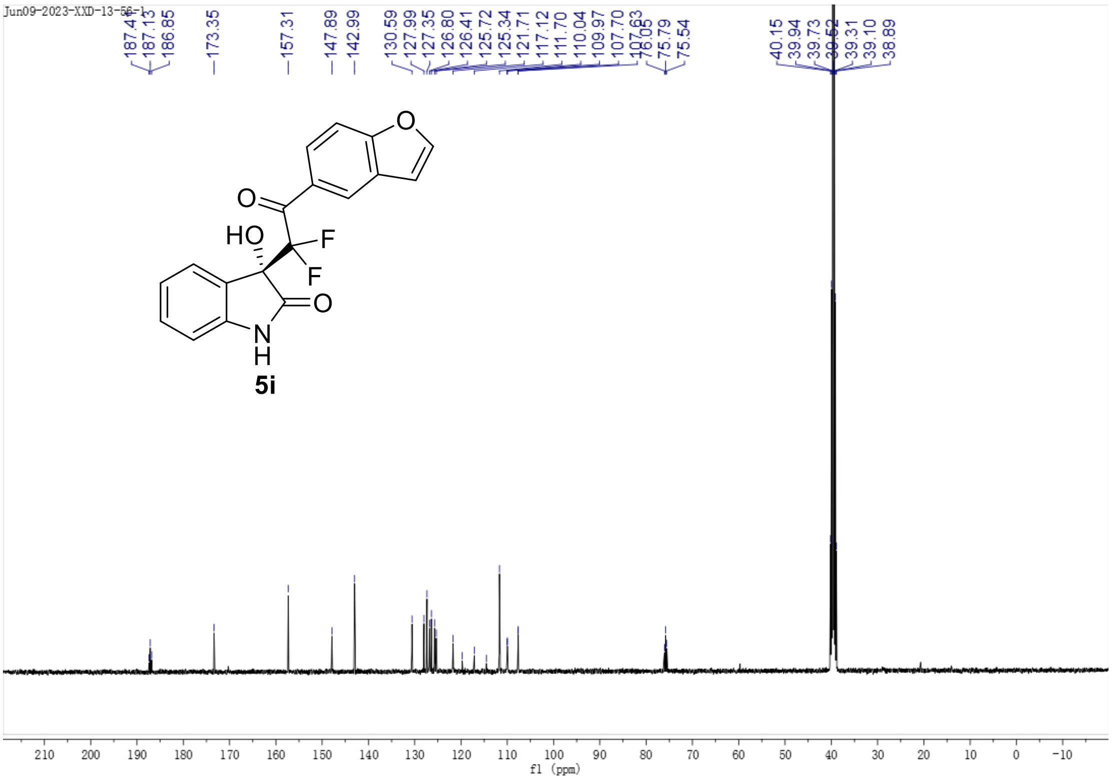
Jun09-2023-XXD-13-56-1
187.41
187.13
186.85
173.35
157.31
147.89
142.99
130.59
127.99
127.35
126.80
126.41
125.72
125.34
121.71
117.12
111.70
110.04
109.97
107.70
107.63
76.05
75.79
75.54
40.15
39.94
39.73
39.52
39.31
39.10
38.89
HO
F
F
O
O
O
N
H
5i
f1 (ppm)

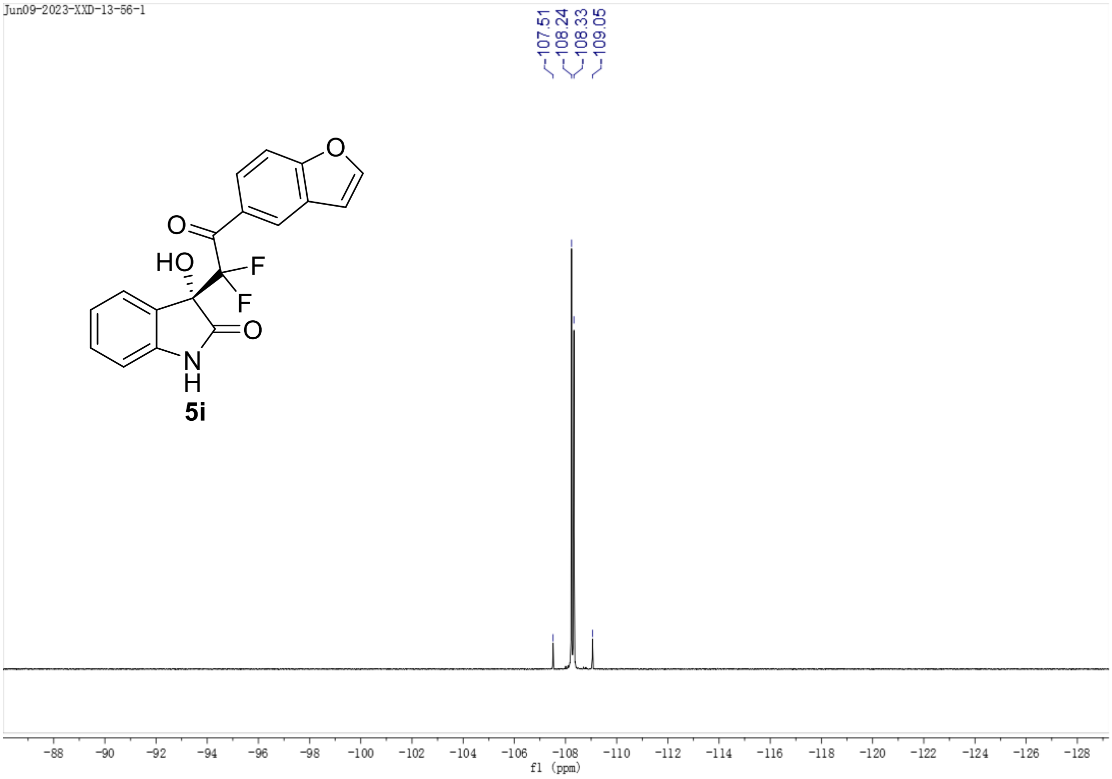
Jun09-2023-XXD-13-56-1
-107.51
-108.24
-108.33
-109.05
5i
f1 (ppm)

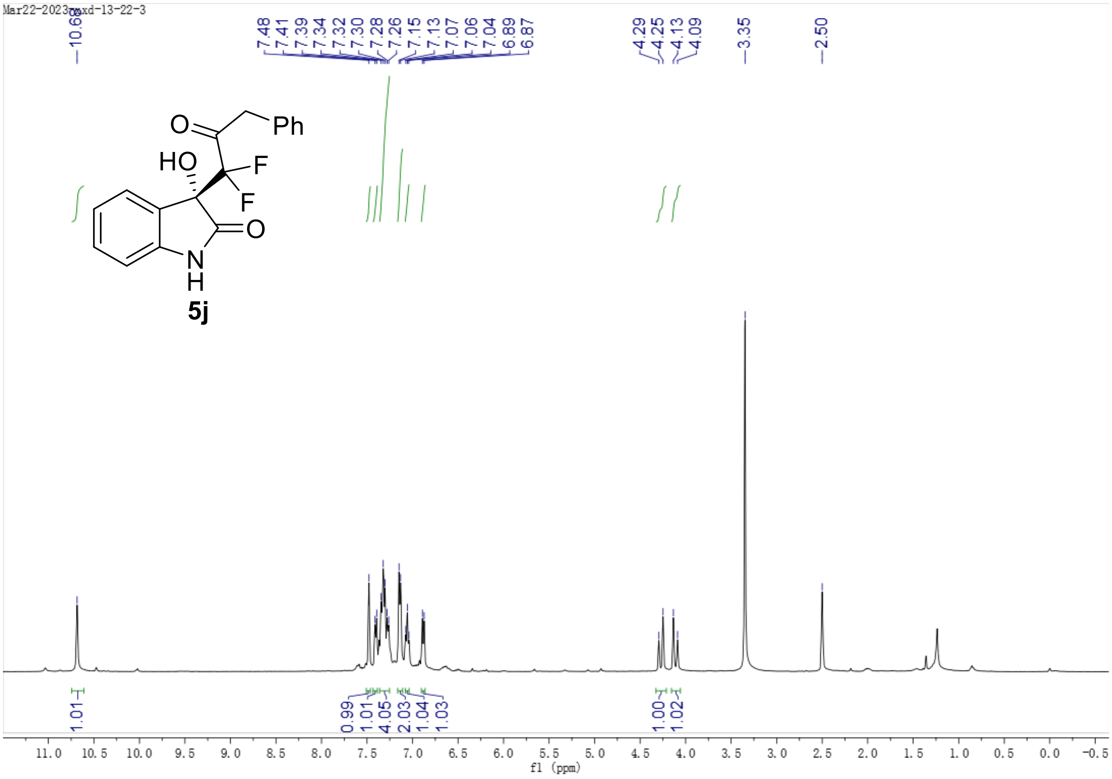
Mar22-2023-xxd-13-22-3
5j
f1 (ppm)

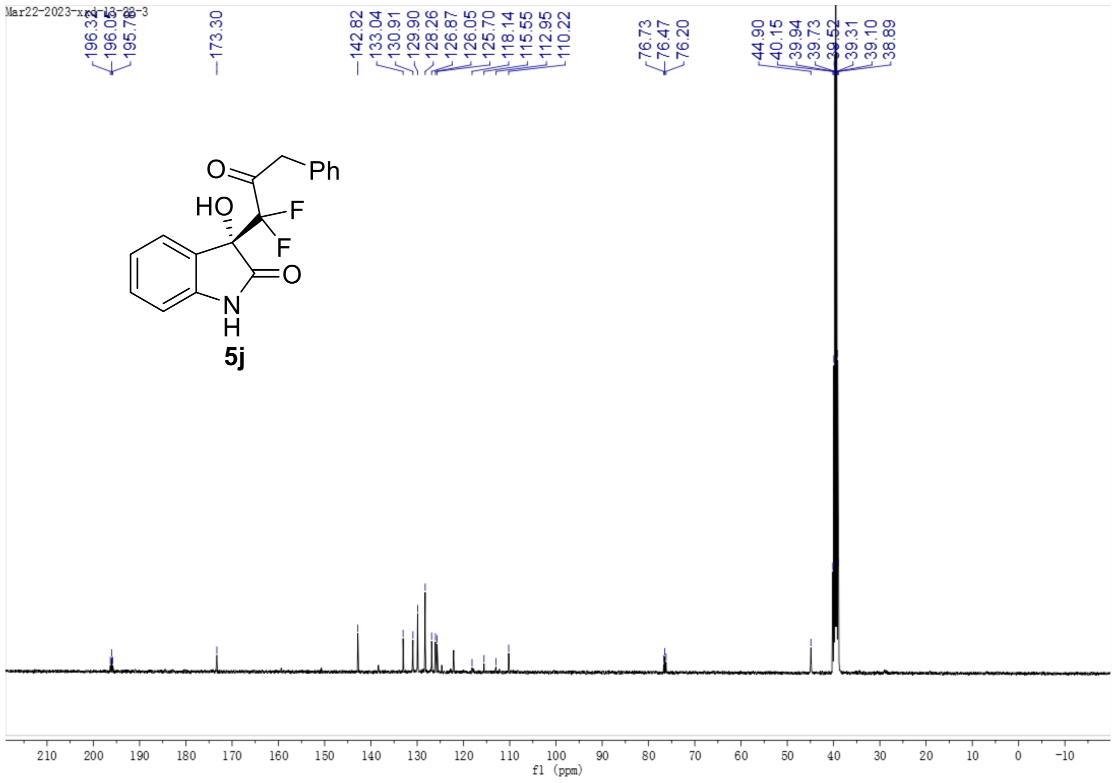
Mar22-2023-xxd-13-22-3
196.32
196.05
195.78
173.30
142.82
133.04
130.91
129.90
128.26
126.87
126.05
125.70
118.14
115.55
112.95
110.22
76.73
76.47
76.20
44.90
40.15
39.94
39.73
39.52
39.31
39.10
38.89
O
Ph
HO
F
F
O
N
H
5j
fl (ppm)

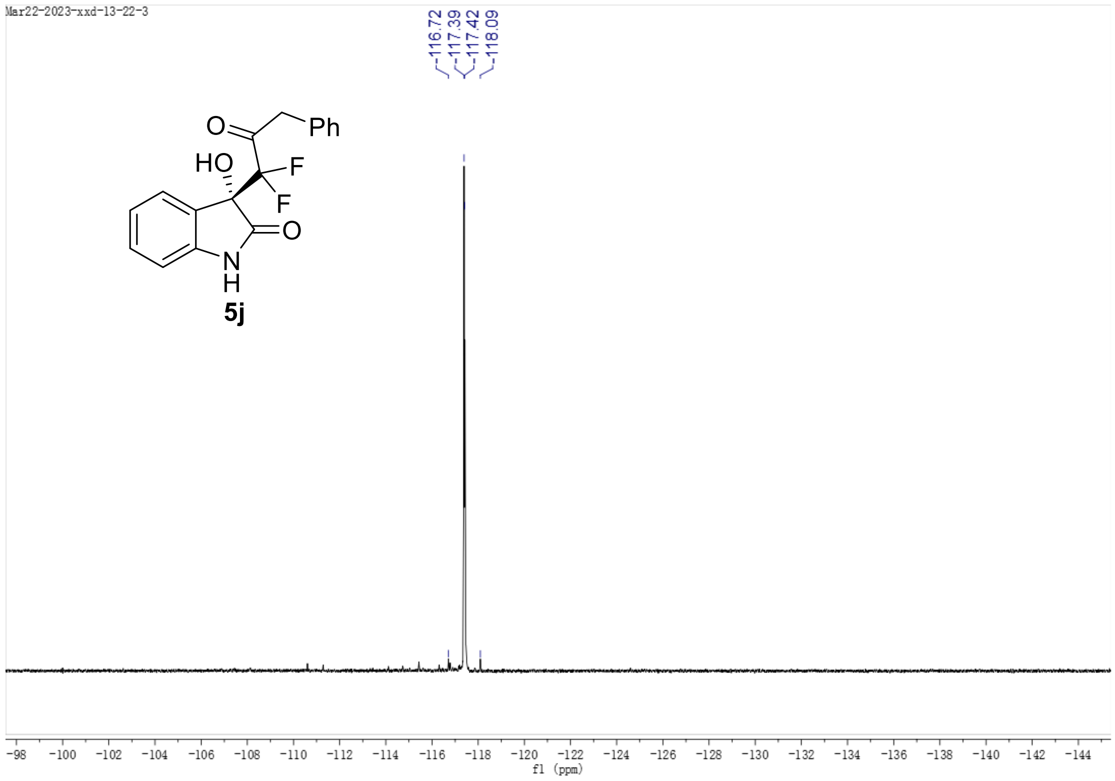
Mar22-2023-xxd-13-22-3
-116.72
-117.39
-117.42
-118.09
O
Ph
HO
F
F
O
N
H
5j
fl (ppm)

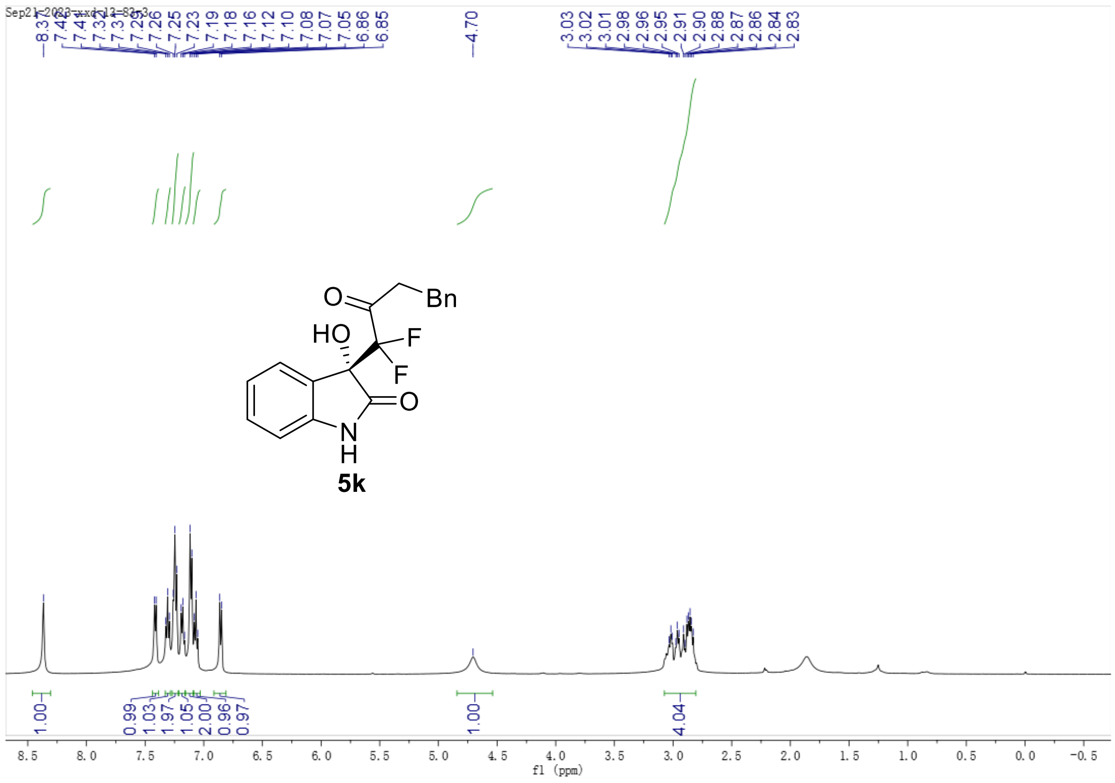

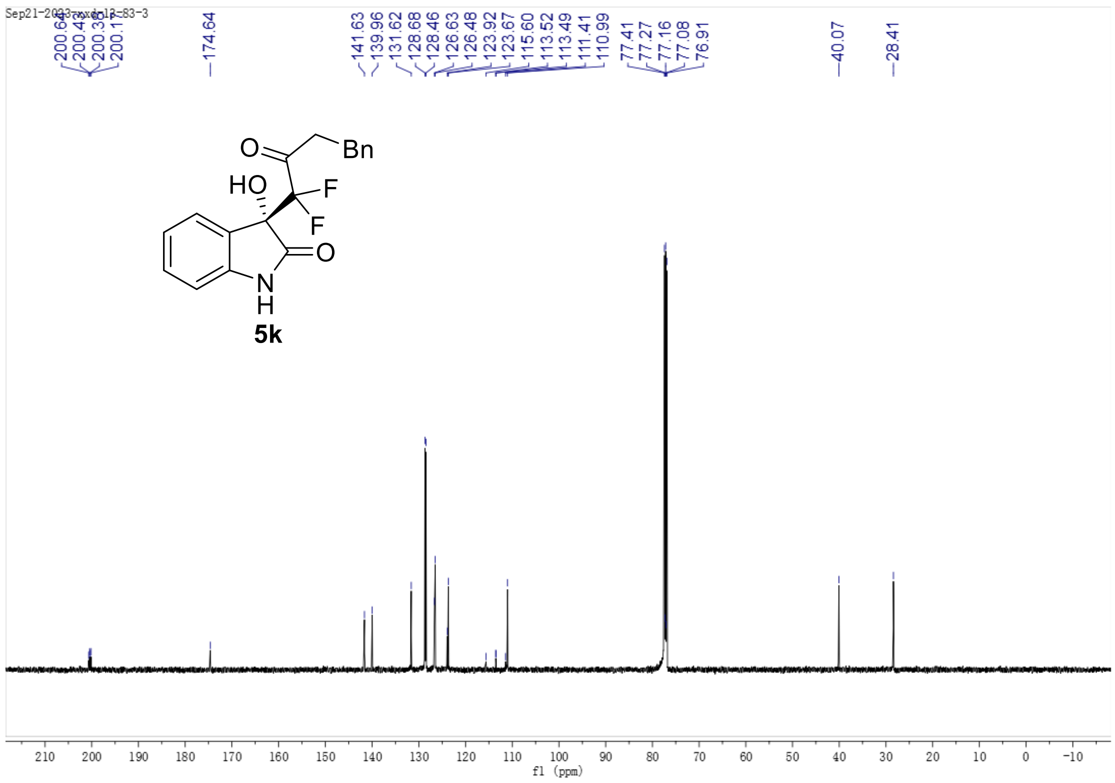

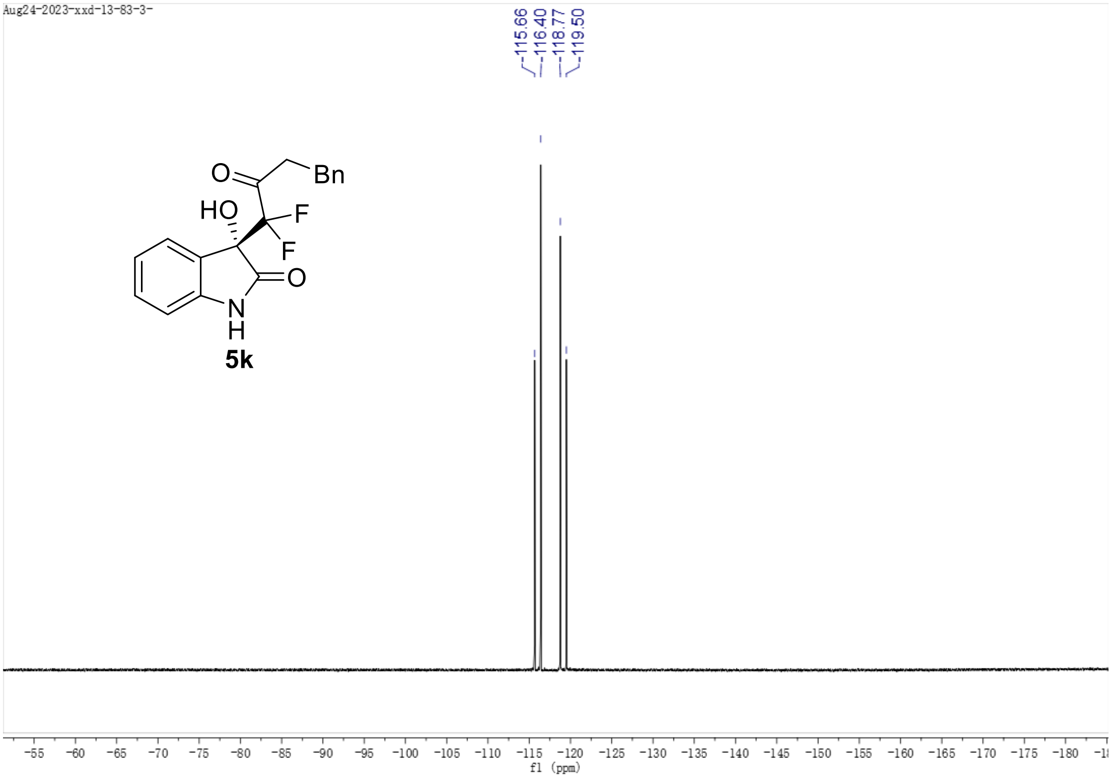
Aug24-2023-xxd-13-83-3-
-115.66
-116.40
-118.77
-119.50
O
Bn
HO
F
F
O
N
H
5k
f1 (ppm)

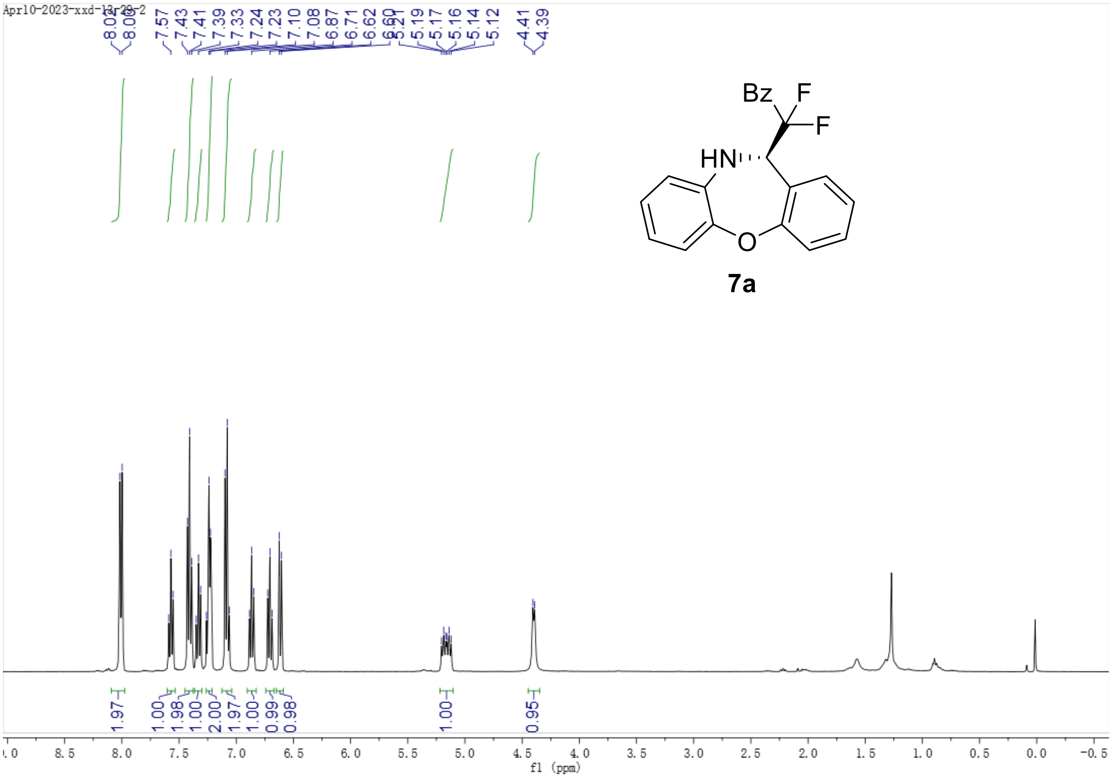
Apr10-2023-xxd-13-29-2
Bz
F
F
HN
O
7a
f1 (ppm)

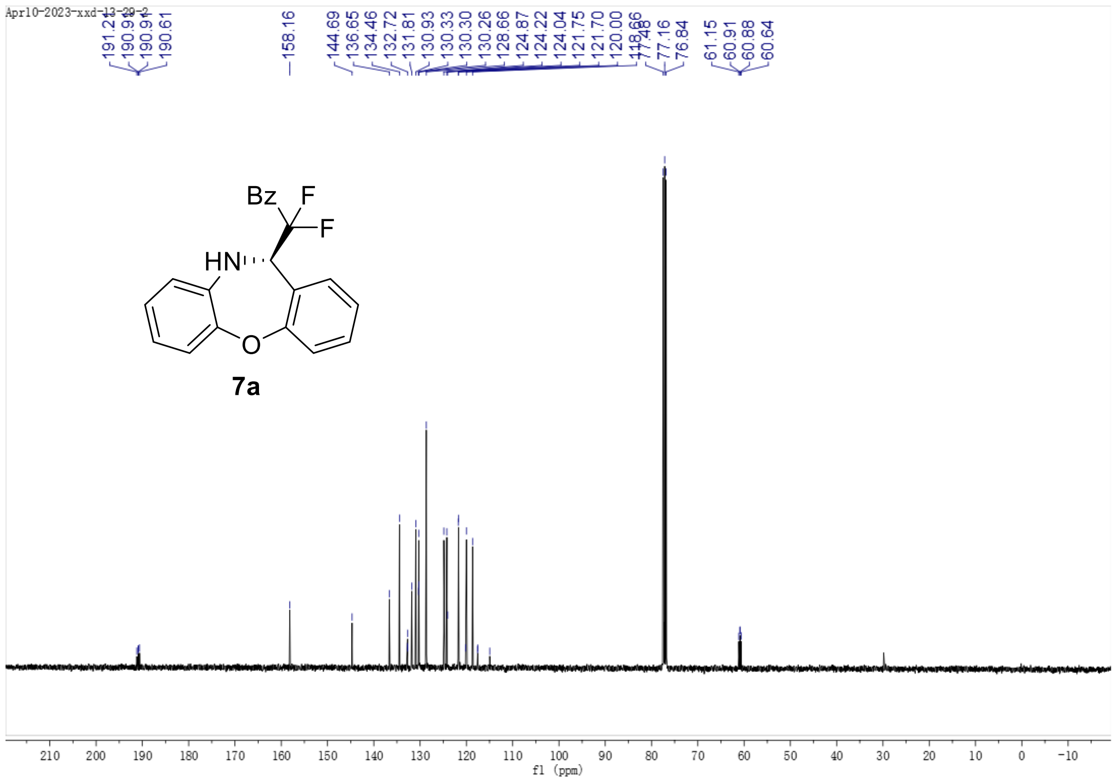

Apr10-2023-xxd-13-29-2
191.21
190.91
190.91
190.61
158.16
144.69
136.65
134.46
132.72
131.81
130.93
130.33
130.30
130.26
128.66
124.87
124.22
124.04
121.75
121.70
120.00
118.66
77.48
77.16
76.84
61.15
60.91
60.88
60.64
Bz
F
F
HN
O
7a
210 200 190 180 170 160 150 140 130 120 110 100 90 80 70 60 50 40 30 20 10 0 -10
f1 (ppm)

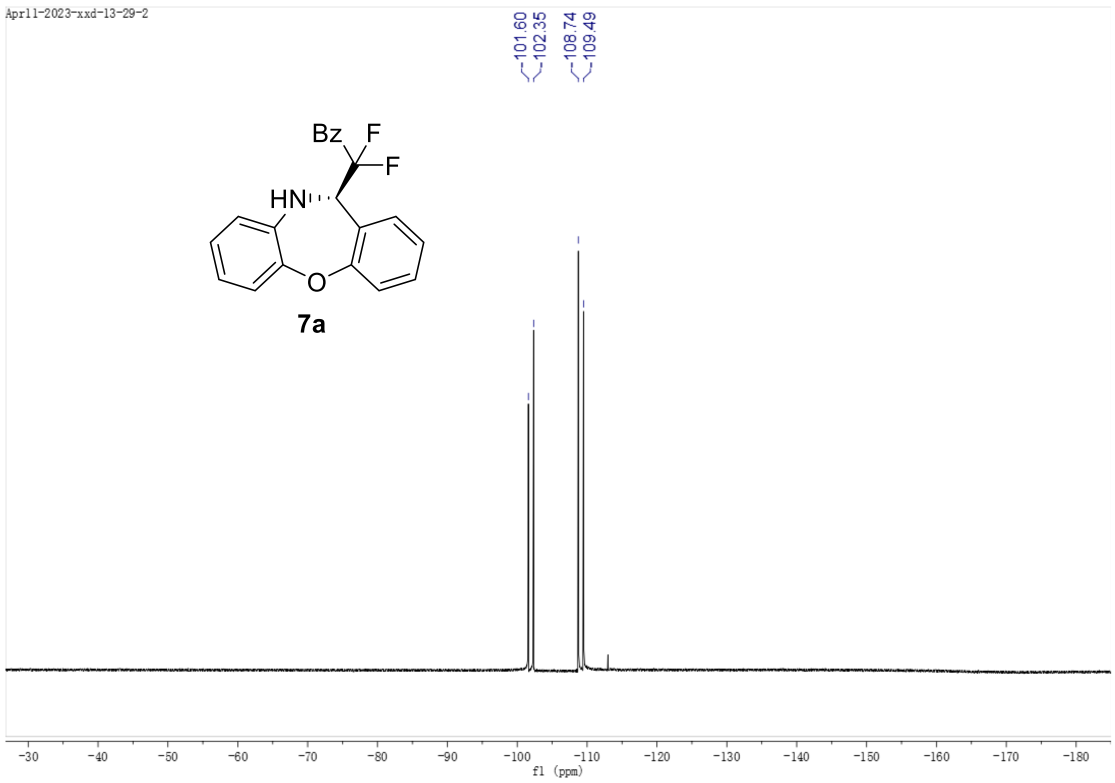

Apr11-2023-xxd-13-29-2
-101.60
-102.35
-108.74
-109.49
Bz
F
F
HN
O
7a
-30 -40 -50 -60 -70 -80 -90 -100 -110 -120 -130 -140 -150 -160 -170 -180
f1 (ppm)

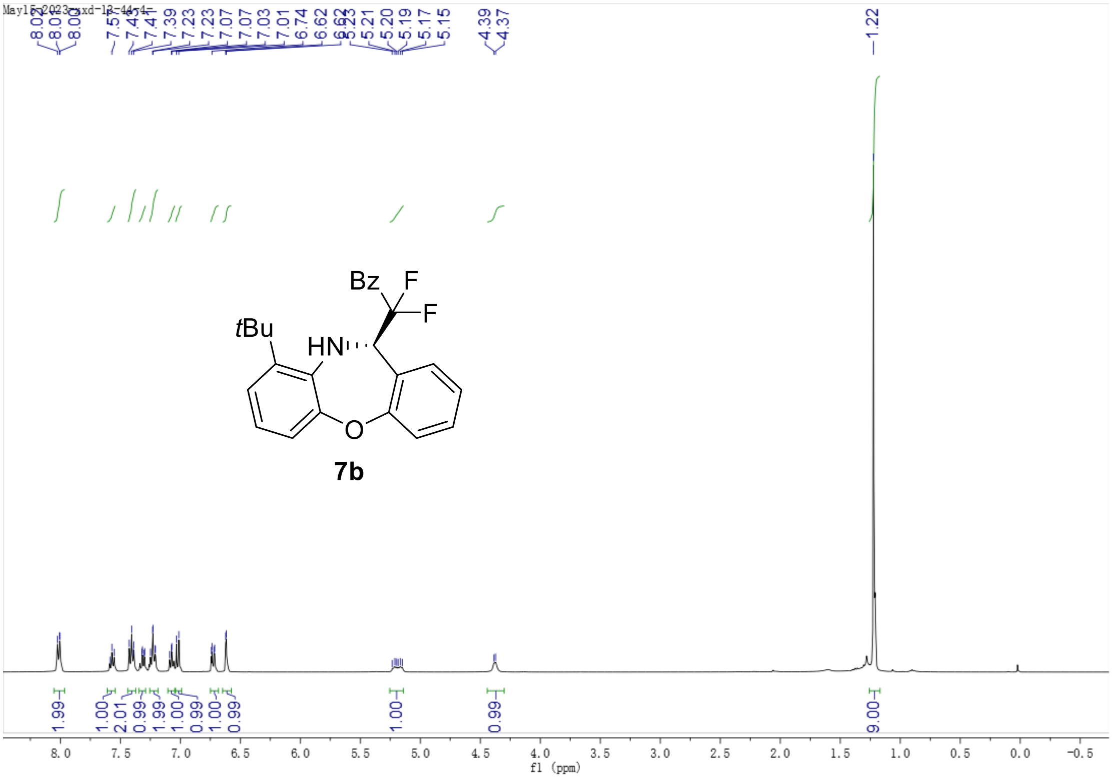

7b

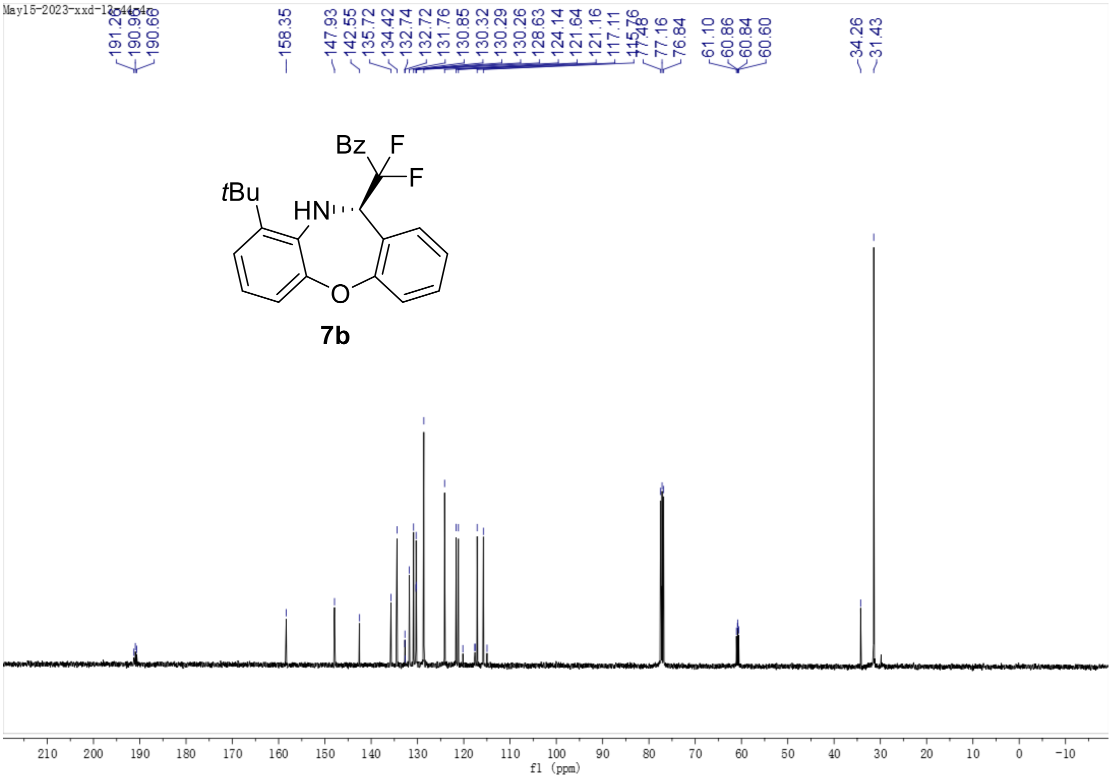

7b

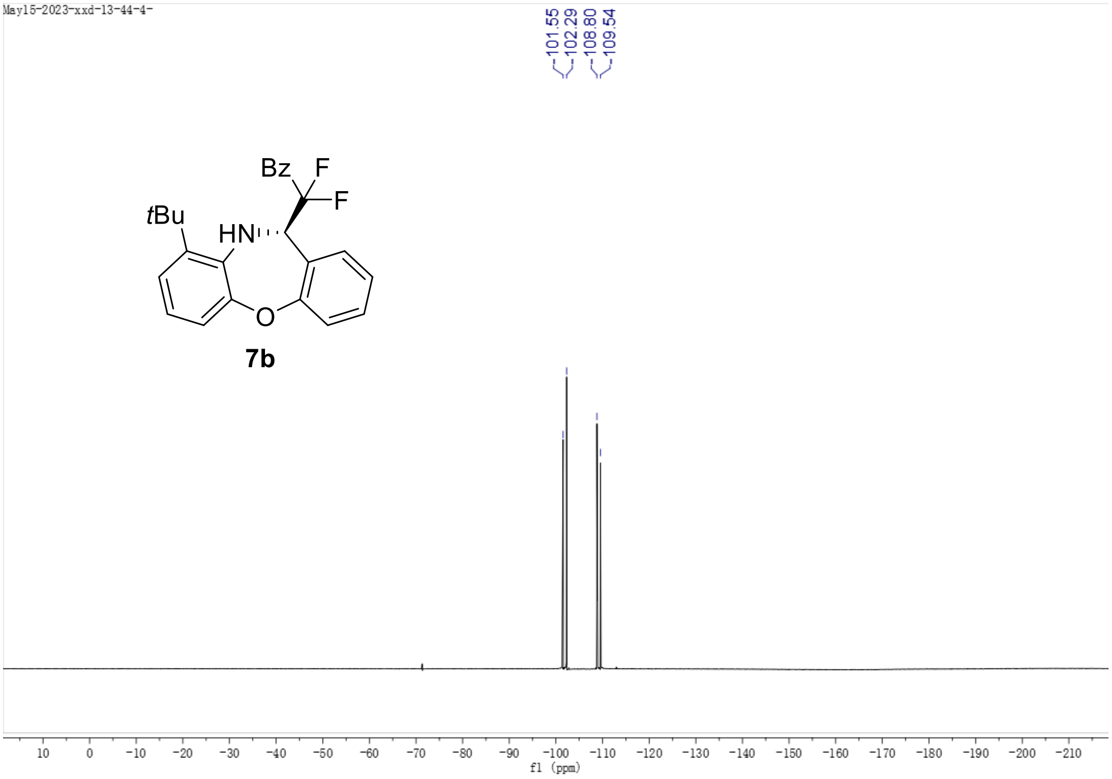
May15-2023-xxd-13-44-4-
-101.55
-102.29
-108.80
-109.54
Bz
F
F
tBu
HN
O
7b
f1 (ppm)

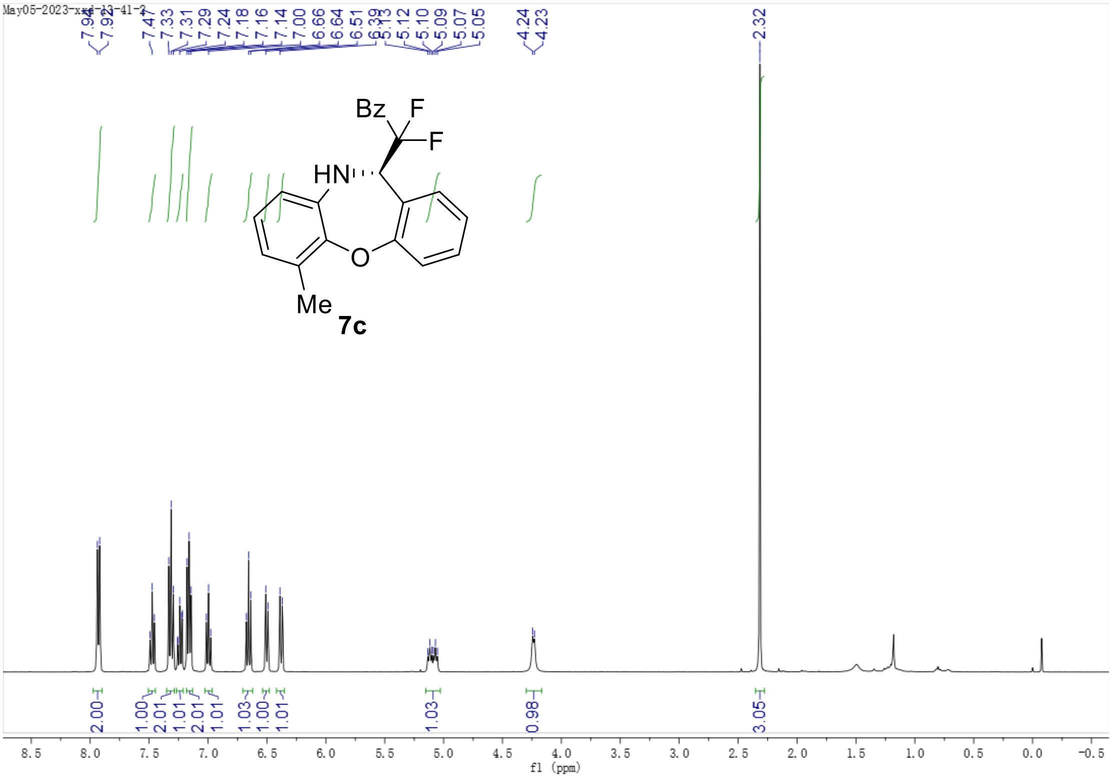
May05-2023-xxd-13-41-2
7.94
7.92
7.47
7.33
7.31
7.29
7.24
7.18
7.16
7.14
7.00
6.66
6.64
6.51
6.39
5.13
5.12
5.10
5.09
5.07
5.05
4.24
4.23
2.32
Bz
F
F
HN
O
Me
7c
2.00
1.00
2.01
1.01
2.01
1.01
1.03
1.00
1.01
1.03
0.98
3.05
f1 (ppm)

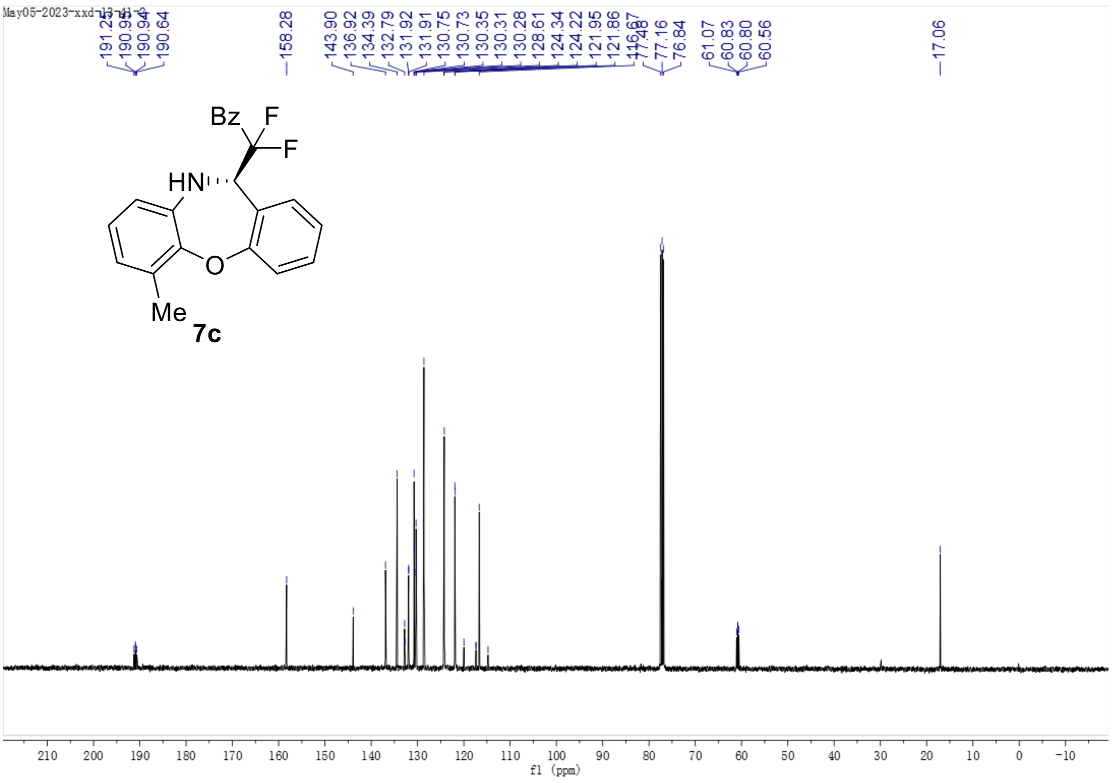

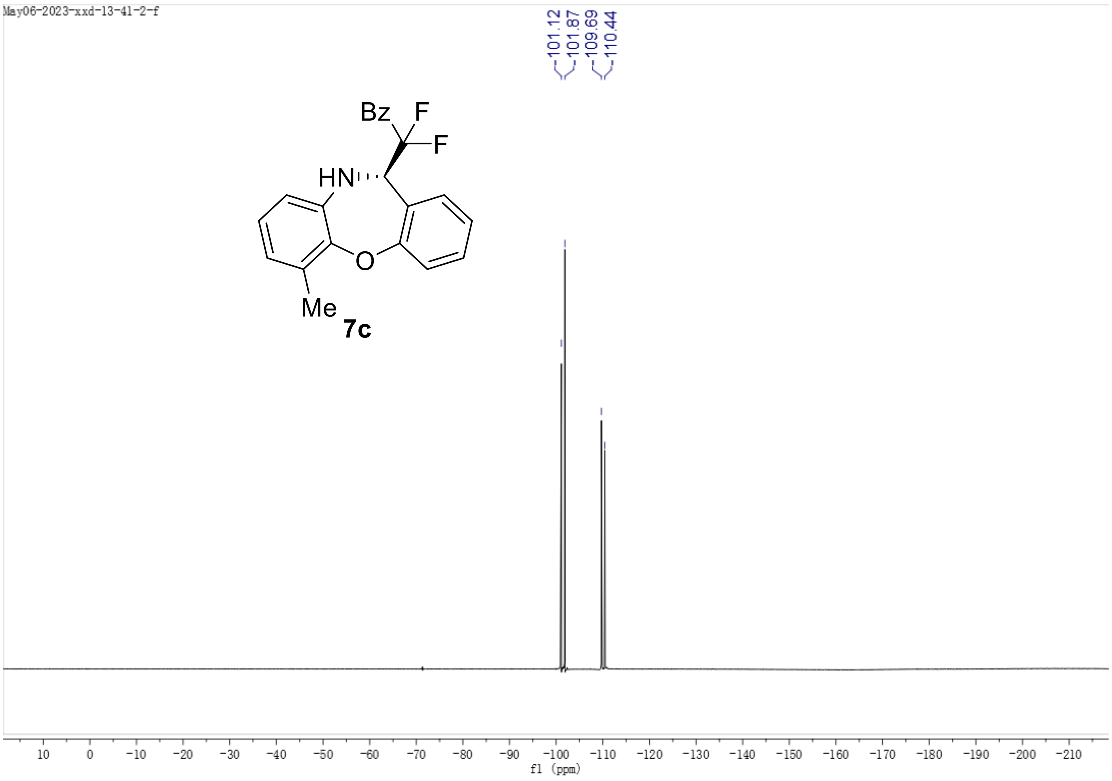

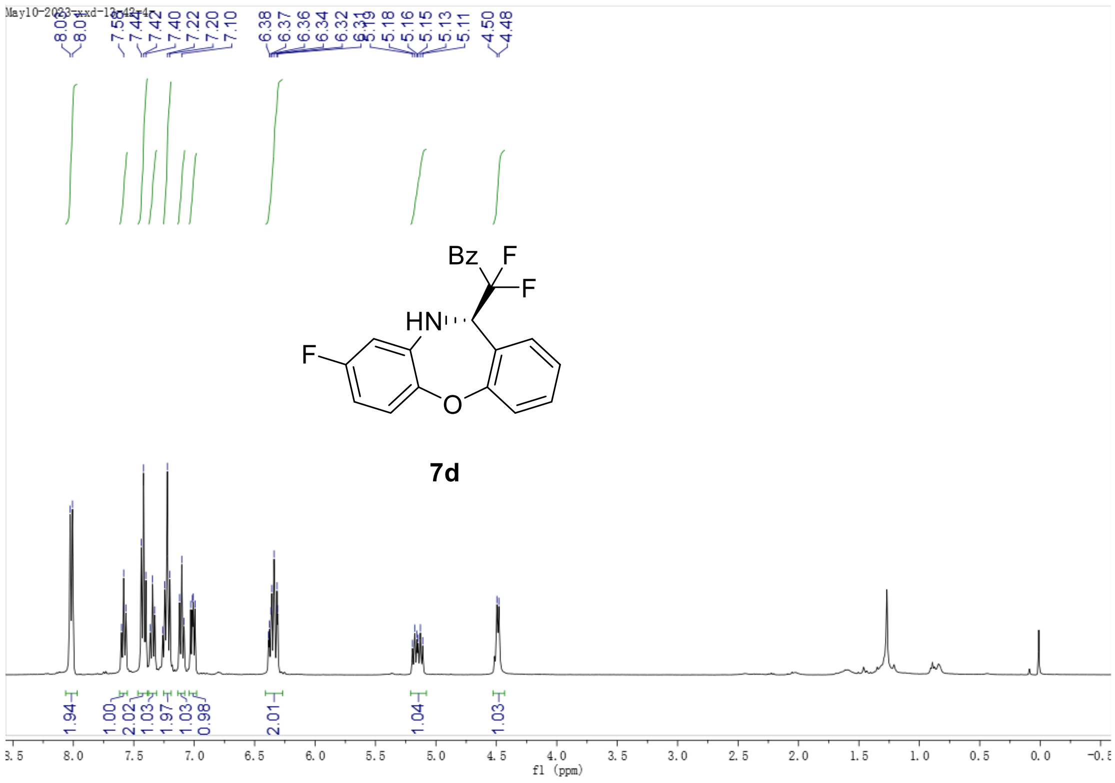
May10-2023-xxd-13-42r4
Bz
F
F
HN
F
O
7d
f1 (ppm)

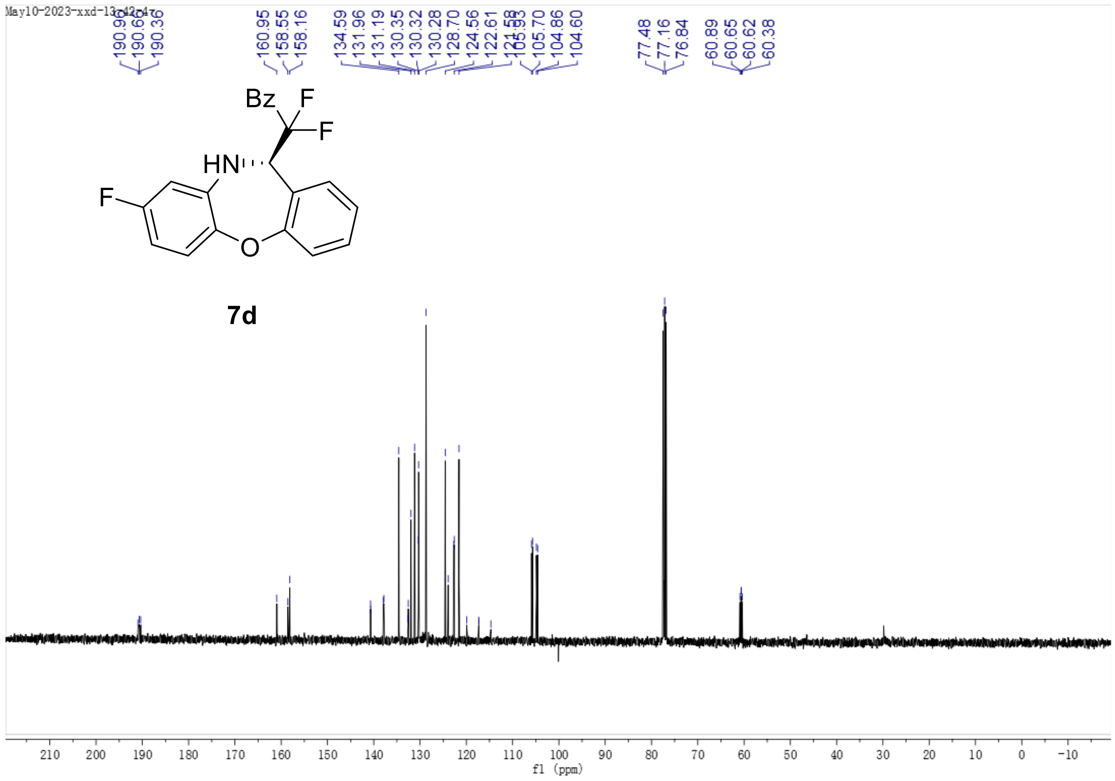
May10-2023-xxd-13-42r4
Bz
F
F
HN
F
O
7d
f1 (ppm)

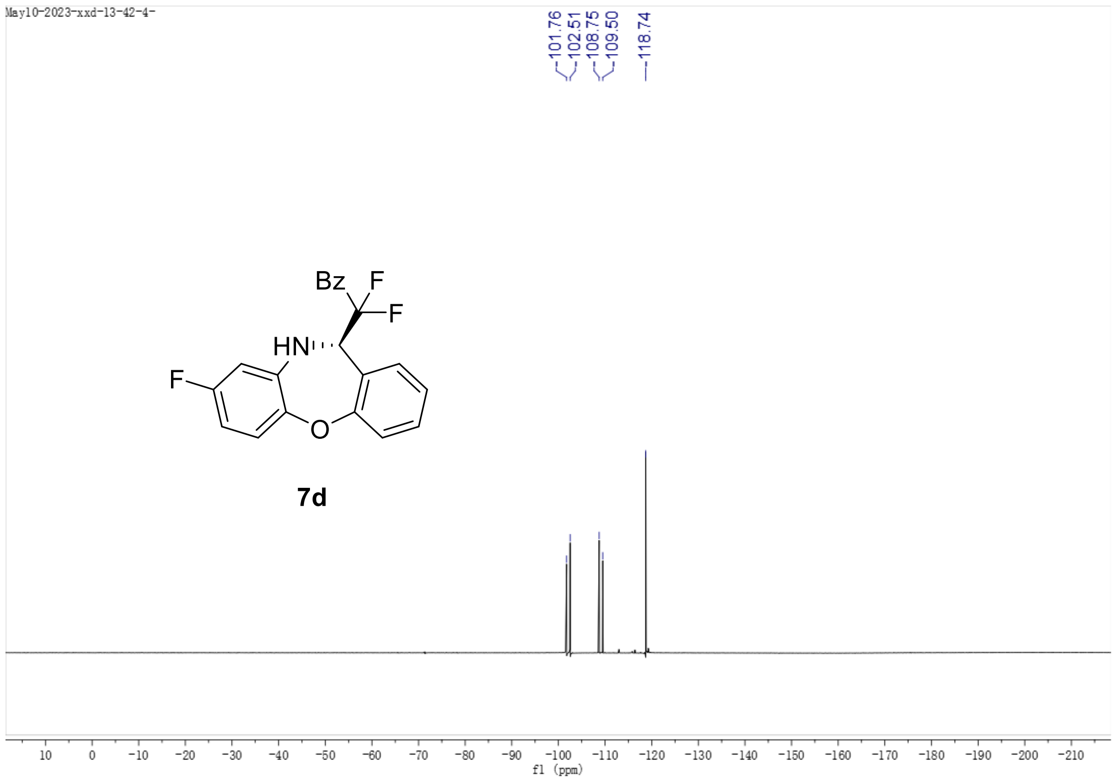

May10-2023-xxd-13-42-4-
101.76
102.51
108.75
109.50
118.74
Bz
F
F
HN
F
O
7d
f1 (ppm)

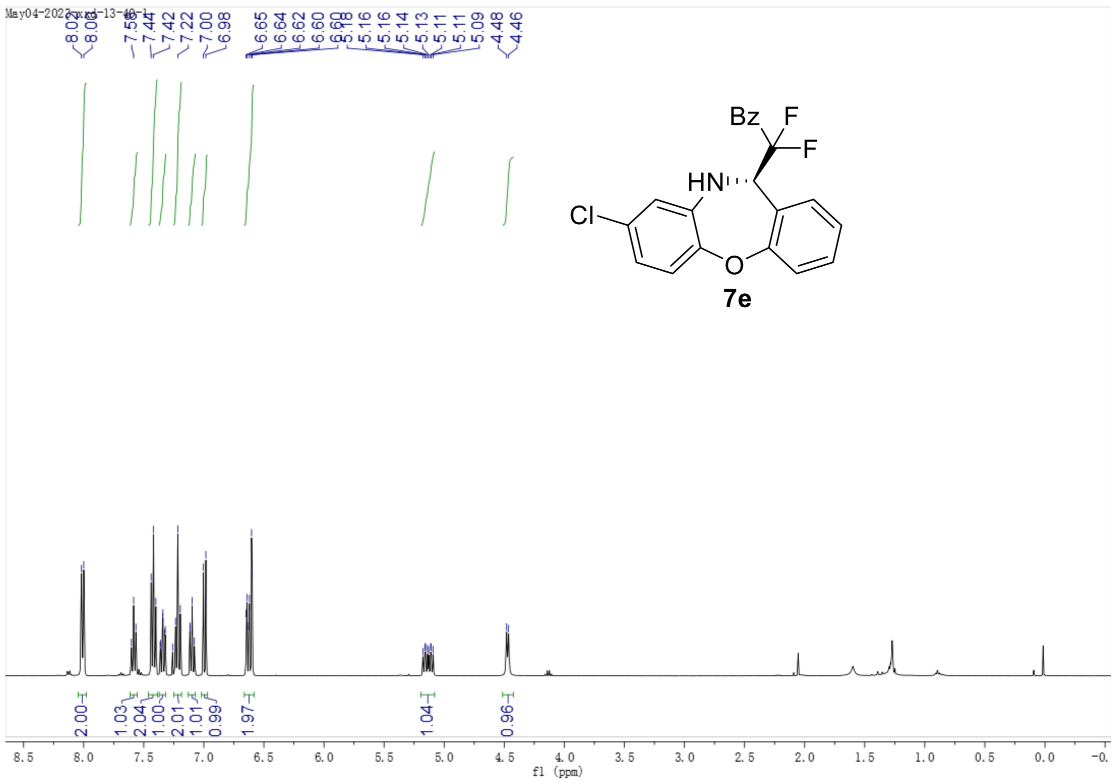

Bz
F
F
HN
Cl
O
7e
f1 (ppm)

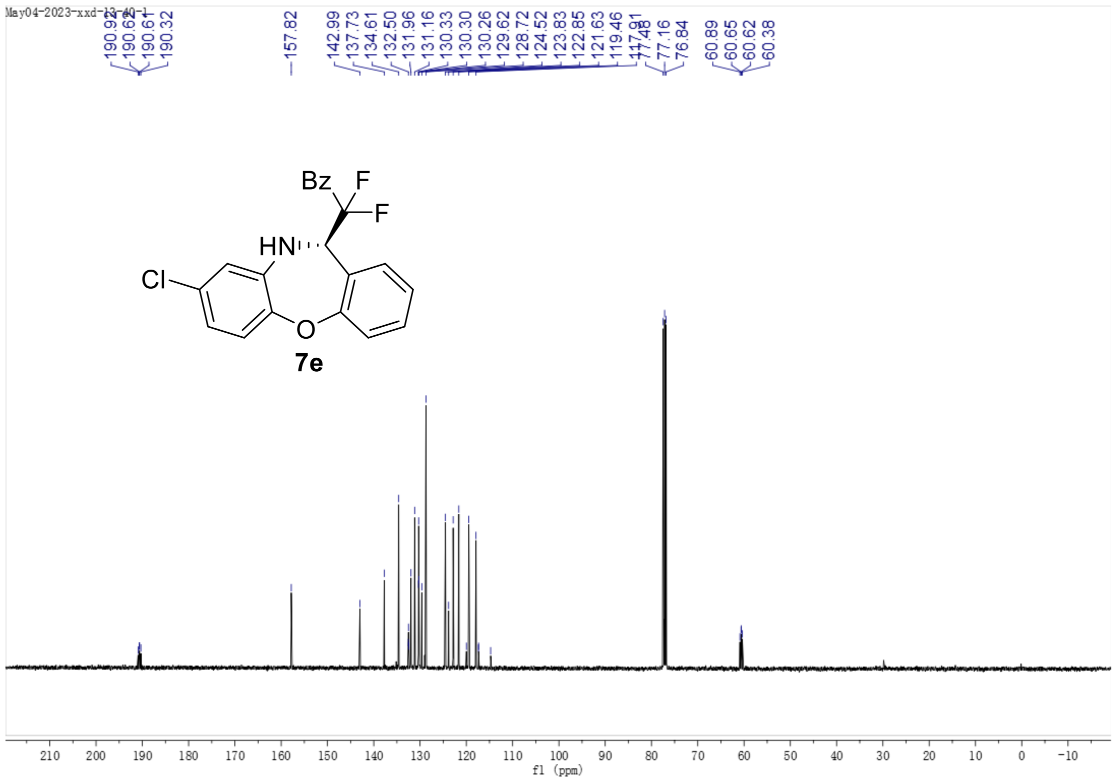
May04-2023-xxd-13-40-1
190.92
190.63
190.61
190.32
157.82
142.99
137.73
134.61
132.50
131.96
131.16
130.33
130.30
130.26
129.62
128.72
124.52
123.83
122.85
121.63
119.46
117.91
117.48
77.16
76.84
60.89
60.65
60.62
60.38
Bz
F
F
HN
Cl
O
7e
f1 (ppm)

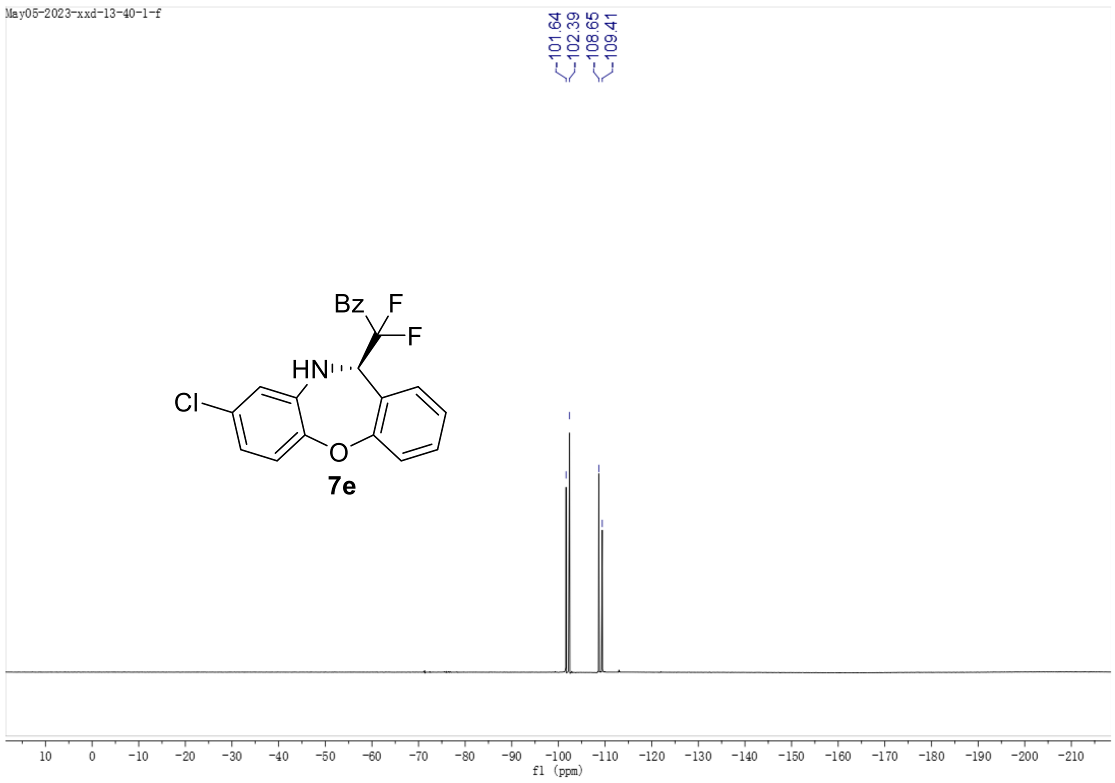
May05-2023-xxd-13-40-1-f
-101.64
-102.39
-108.65
-109.41
Bz
F
F
HN
Cl
O
7e
f1 (ppm)

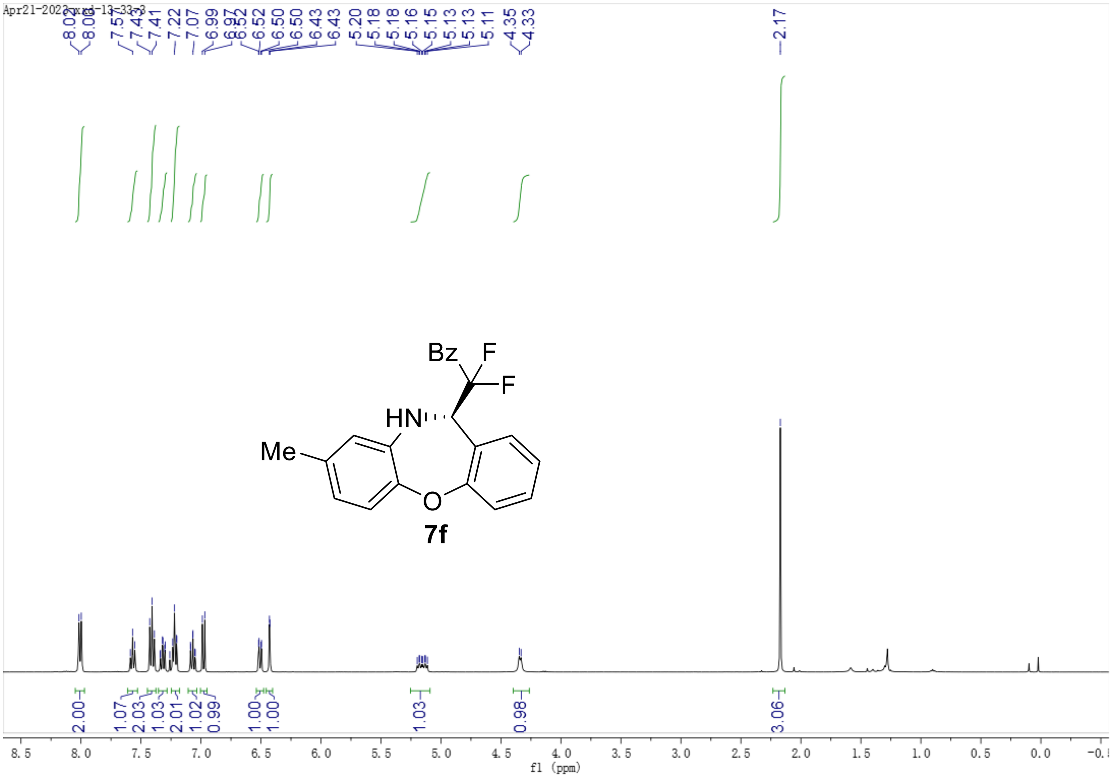

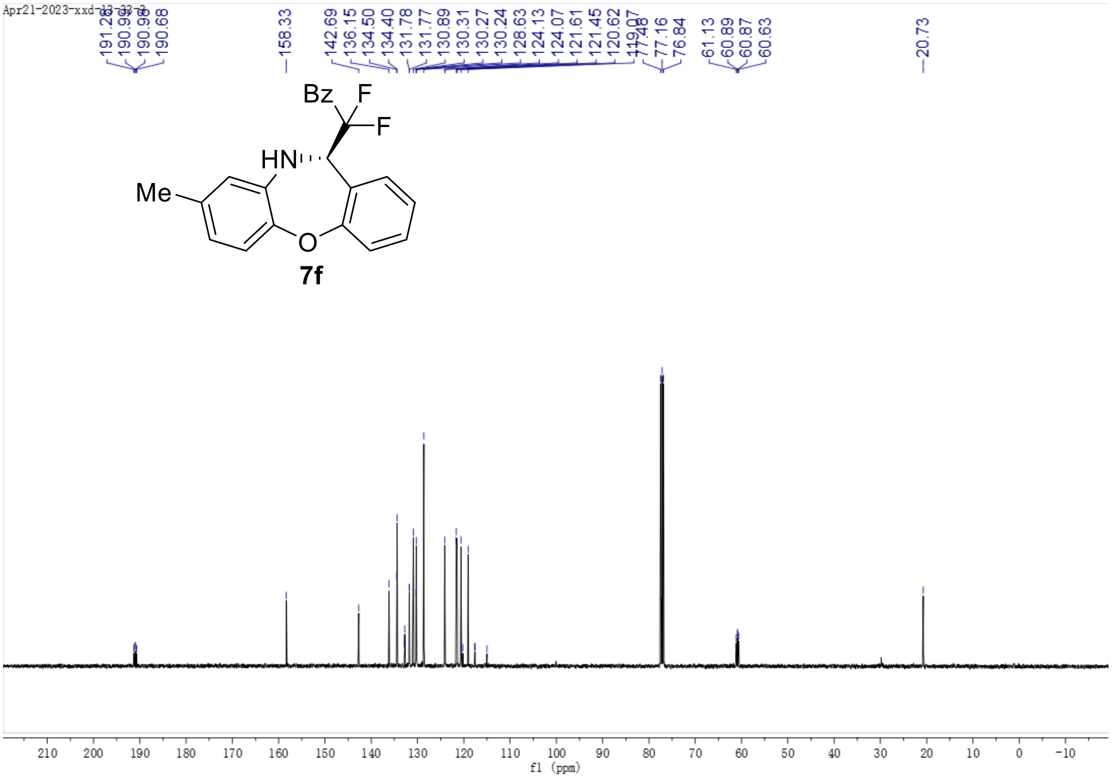

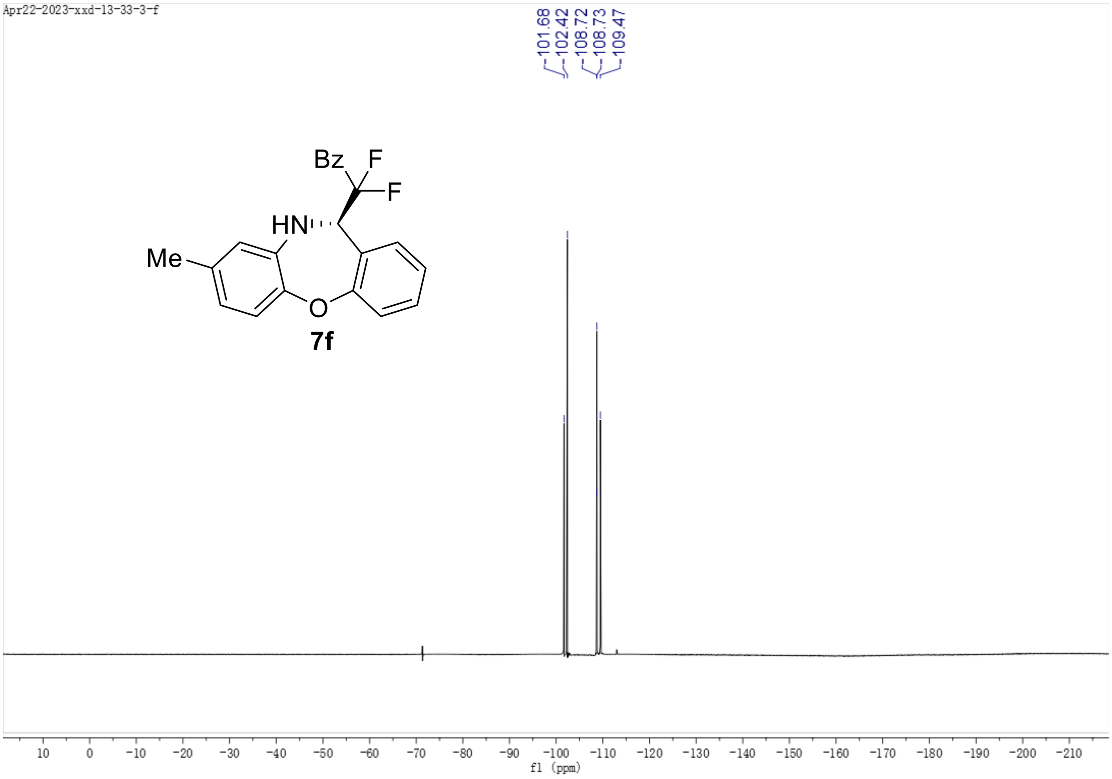
Apr22-2023-xxd-13-33-3-f
101.68
102.42
108.72
108.73
109.47
Bz
F
F
HN
Me
O
7f
f1 (ppm)

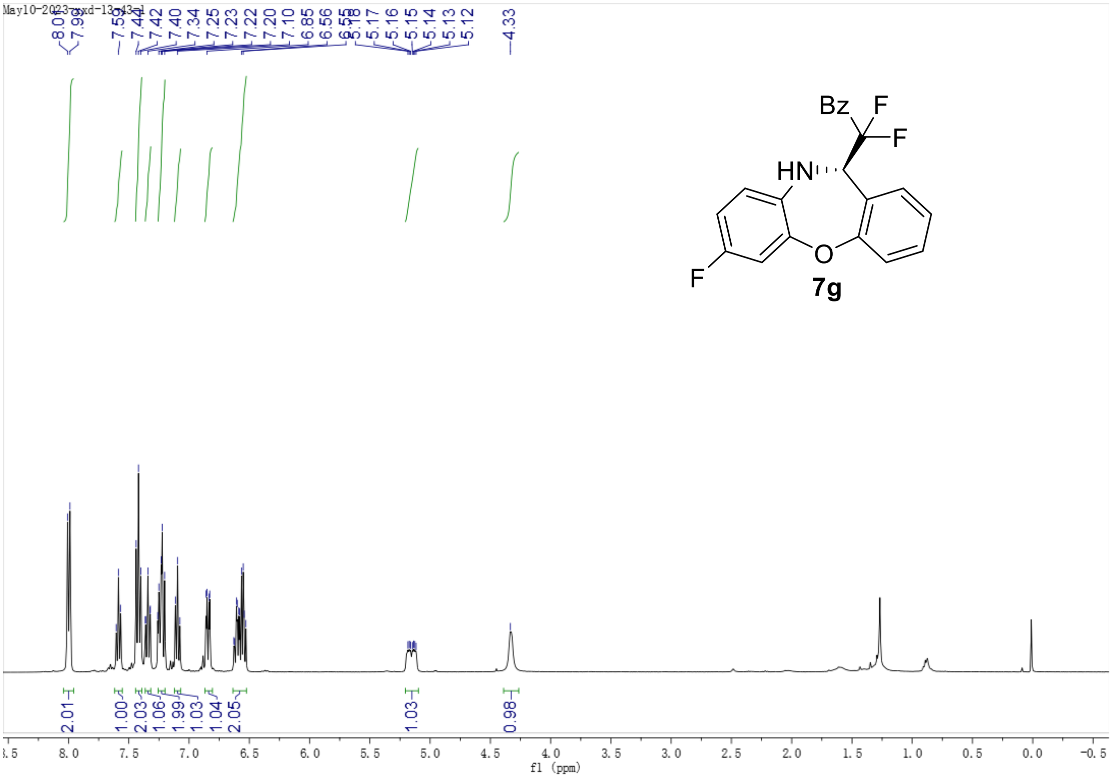
8.01
7.99
7.59
7.44
7.42
7.40
7.34
7.25
7.23
7.22
7.20
7.10
6.85
6.56
5.17
5.16
5.15
5.14
5.13
5.12
4.33
Bz
F
F
HN
F
O
7g
2.01
1.00
2.03
1.06
1.99
1.03
1.04
2.05
1.03
0.98
f1 (ppm)

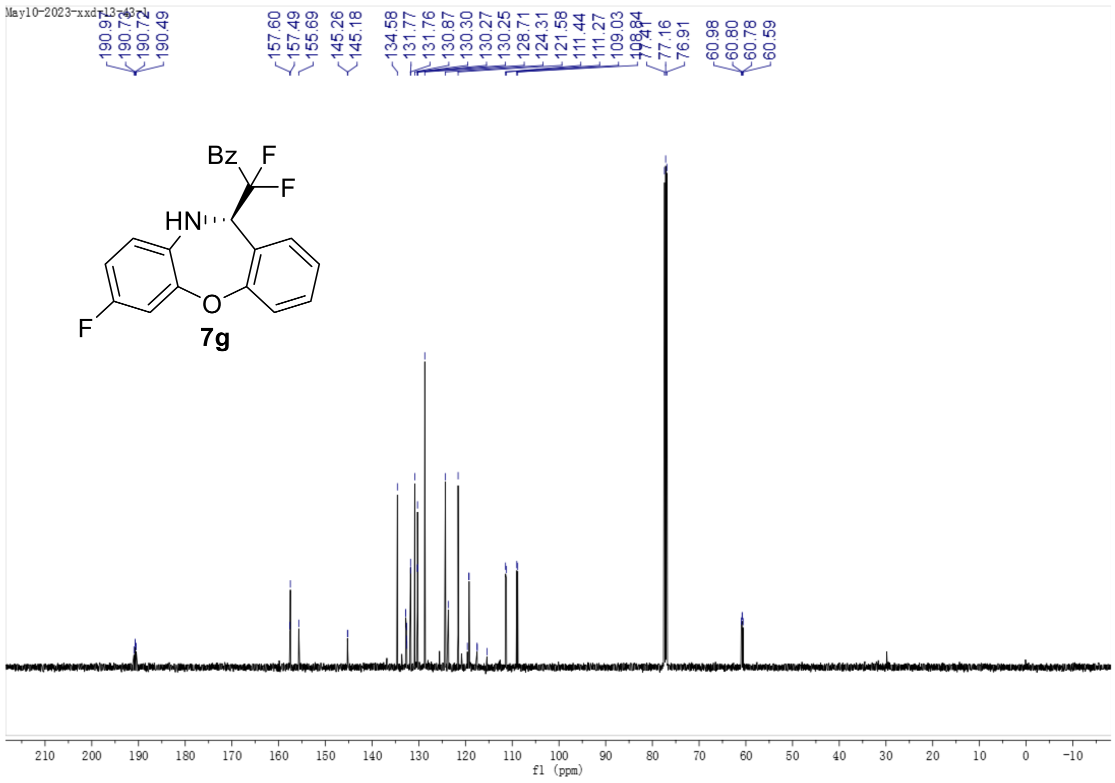
May10-2023-xxd-13-43-1
190.97
190.74
190.72
190.49
157.60
157.49
155.69
145.26
145.18
134.58
131.77
131.76
130.87
130.30
130.27
130.25
128.71
124.31
121.58
111.44
111.27
109.03
108.84
77.41
77.16
76.91
60.98
60.80
60.78
60.59
Bz
F
F
HN
O
F
7g
f1 (ppm)

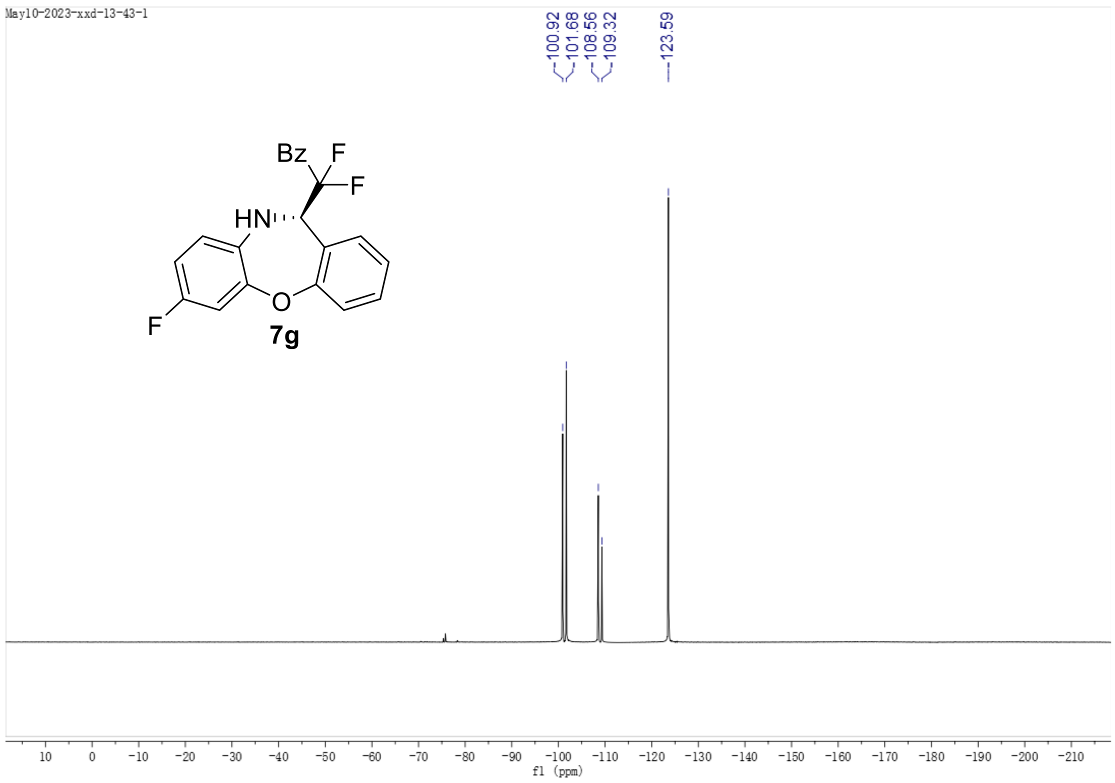
May10-2023-xxd-13-43-1
-100.92
-101.68
-108.56
-109.32
-123.59
Bz
F
F
HN
O
F
7g
f1 (ppm)

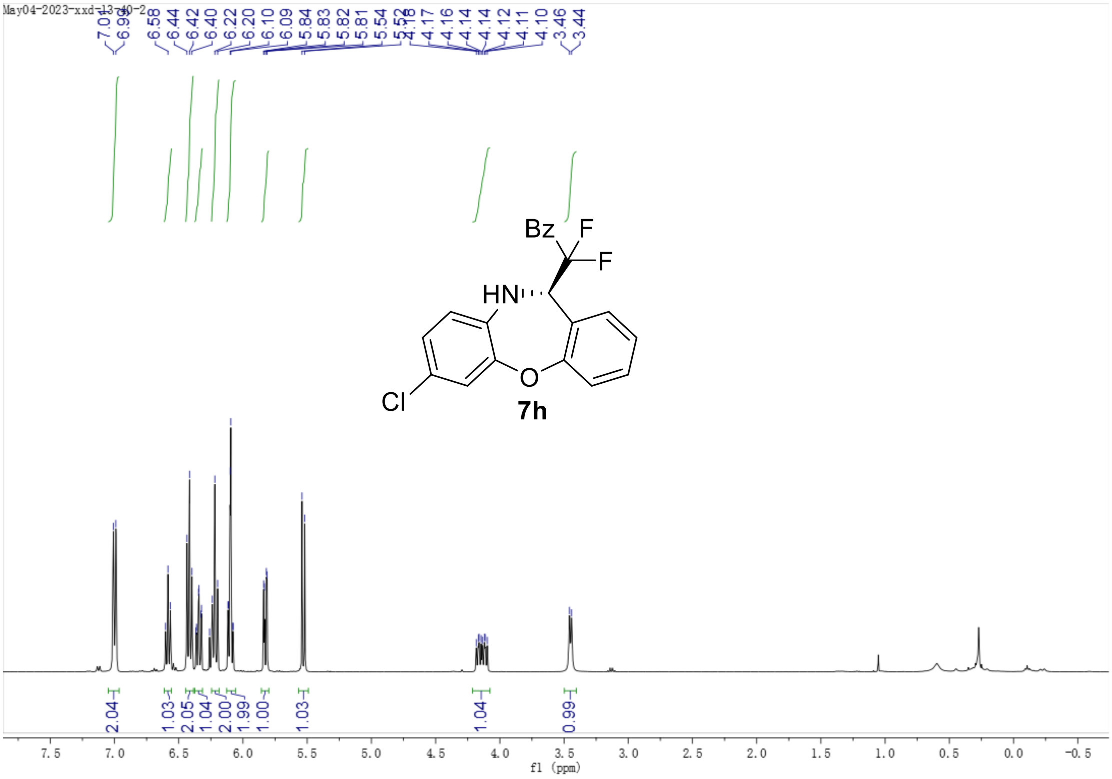
May04-2023-xxd-13-40-2
6.58
6.44
6.42
6.40
6.22
6.20
6.10
6.09
5.84
5.83
5.82
5.81
5.54
4.17
4.16
4.14
4.12
4.11
4.10
3.46
3.44
Bz
F
F
HN
O
Cl
7h
2.04
1.03
2.05
1.04
2.00
1.99
1.00
1.03
1.04
0.99
f1 (ppm)

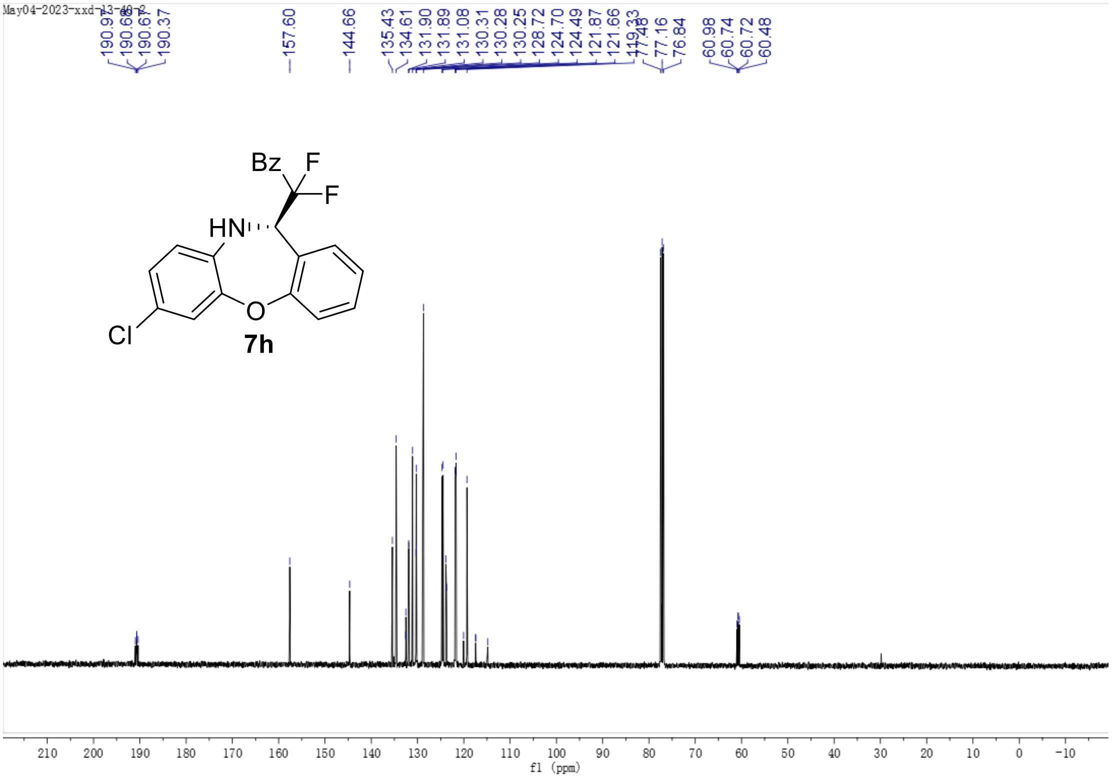
May04-2023-xxd-13-40-2
190.97
190.37
157.60
144.66
135.43
134.61
131.90
131.89
131.08
130.31
130.28
130.25
128.72
124.70
124.49
121.87
121.66
77.16
76.84
60.98
60.74
60.72
60.48
Bz
F
F
HN
O
Cl
7h
f1 (ppm)

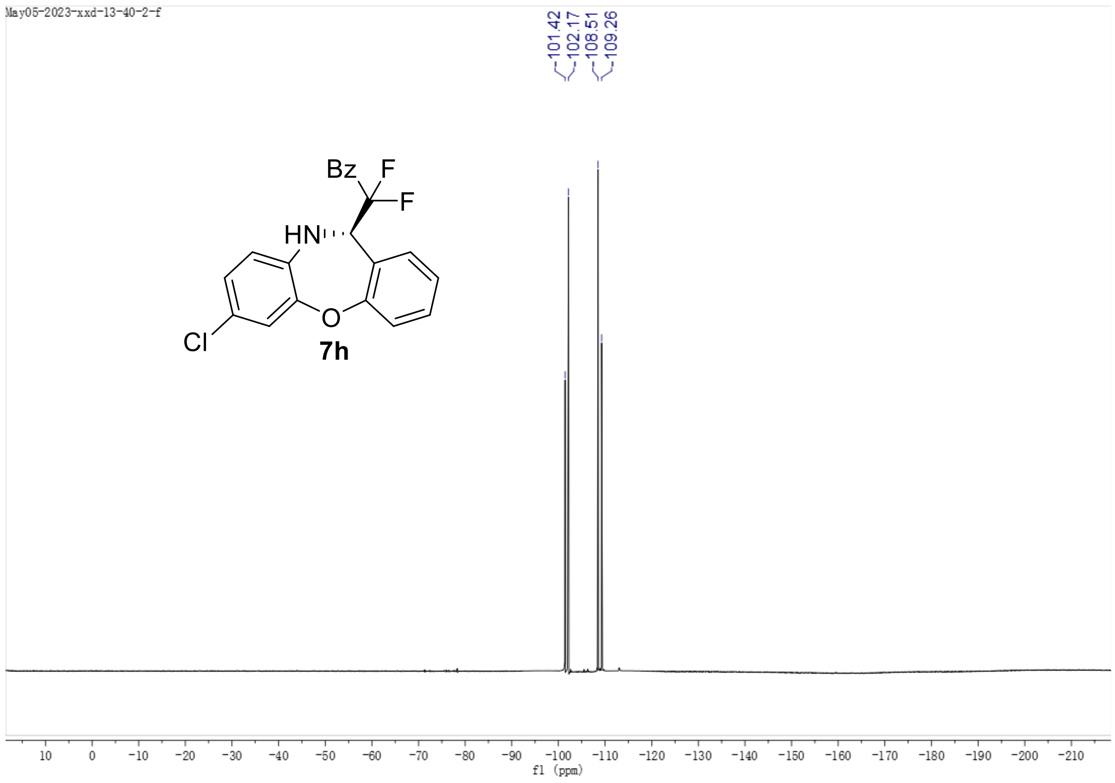
May05-2023-xxd-13-40-2-f
-101.42
-102.17
-108.51
-109.26
Bz
F
F
HN
O
Cl
7h
f1 (ppm)

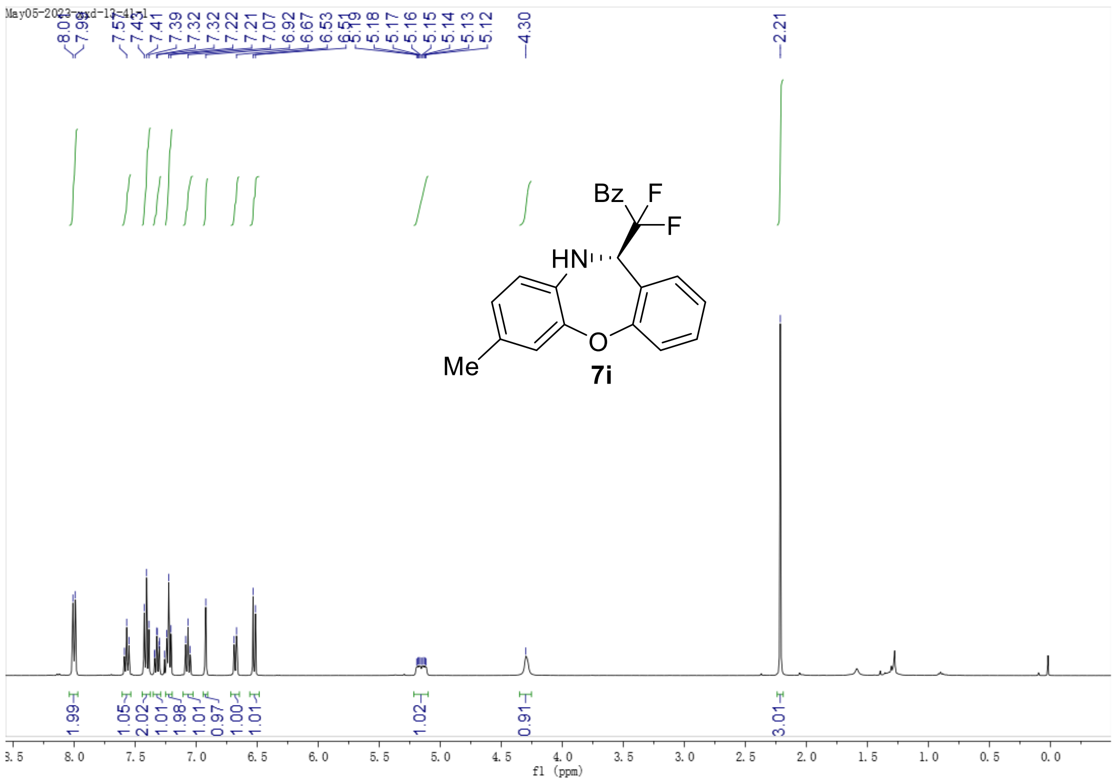
May05-2023-xxd-13-41-1
8.01
7.99
7.57
7.43
7.41
7.39
7.32
7.32
7.22
7.21
7.07
6.92
6.67
6.53
6.51
5.18
5.17
5.16
5.15
5.14
5.13
5.12
4.30
2.21
Bz
F
F
HN
O
Me
7i
1.99
1.05
2.02
1.01
1.98
1.01
0.97
1.00
1.01
1.02
0.91
3.01
f1 (ppm)

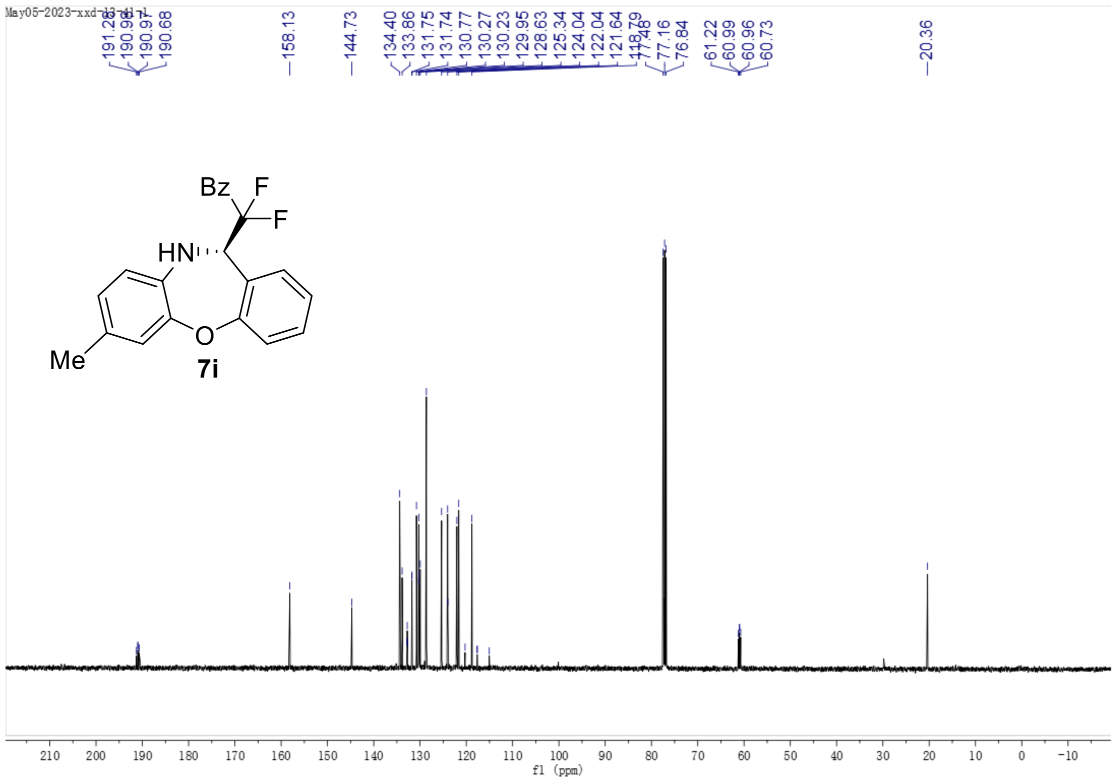
May05-2023-xxd-13-41-1
191.26
190.98
190.91
190.68
158.13
144.73
134.40
133.86
131.75
131.74
130.77
130.27
130.23
129.95
128.63
125.34
124.04
122.04
121.64
118.79
77.48
77.16
76.84
61.22
60.99
60.96
60.73
20.36
Bz
F
F
HN
O
Me
7i
210 200 190 180 170 160 150 140 130 120 110 100 90 80 70 60 50 40 30 20 10 0 -10
f1 (ppm)

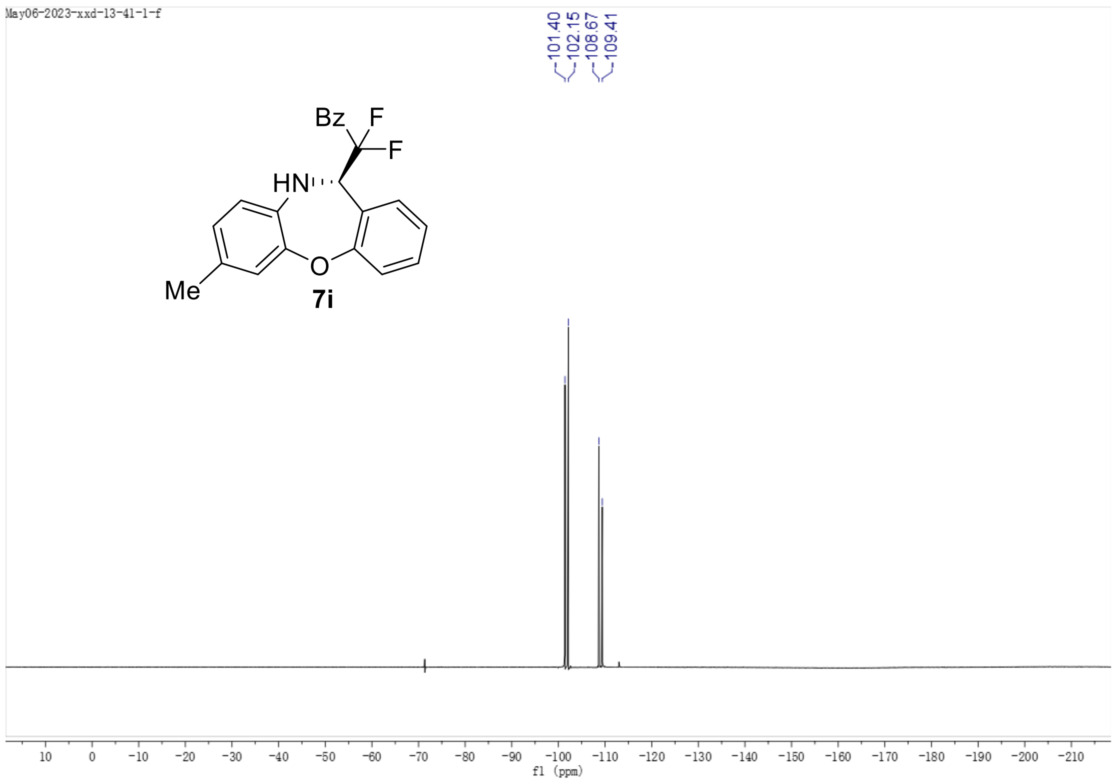
May06-2023-xxd-13-41-1-f
-101.40
-102.15
-108.67
-109.41
Bz
F
F
HN
O
Me
7i
10 0 -10 -20 -30 -40 -50 -60 -70 -80 -90 -100 -110 -120 -130 -140 -150 -160 -170 -180 -190 -200 -210
f1 (ppm)

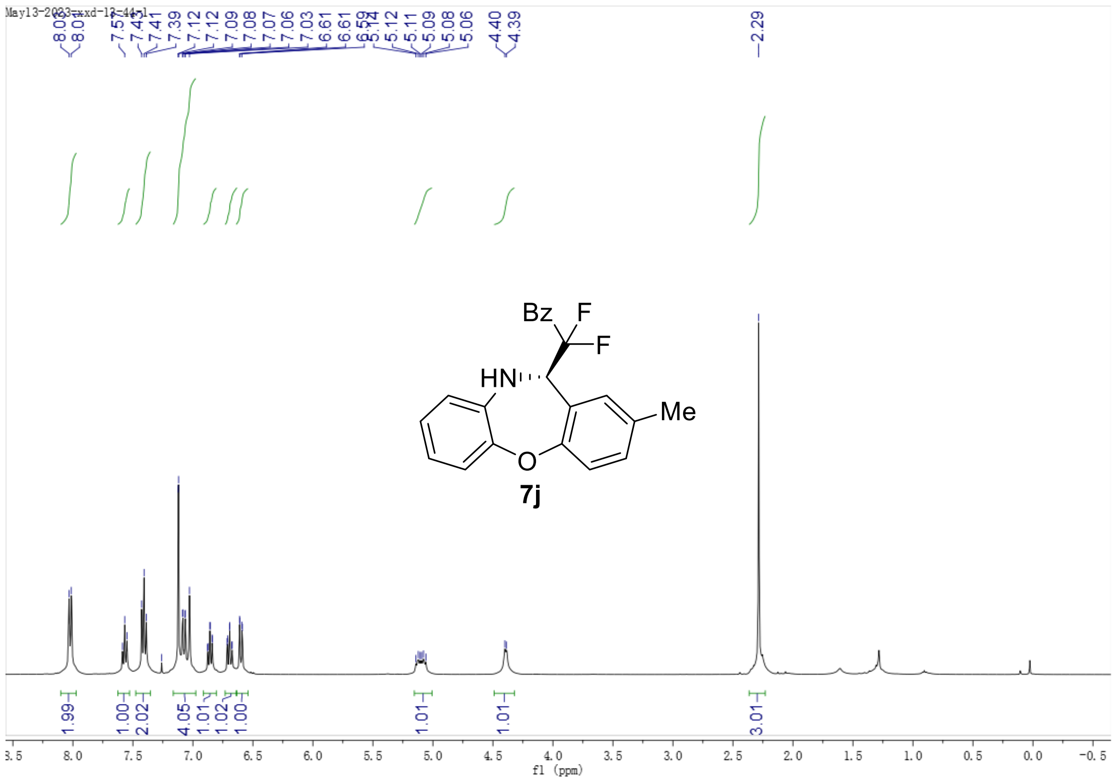

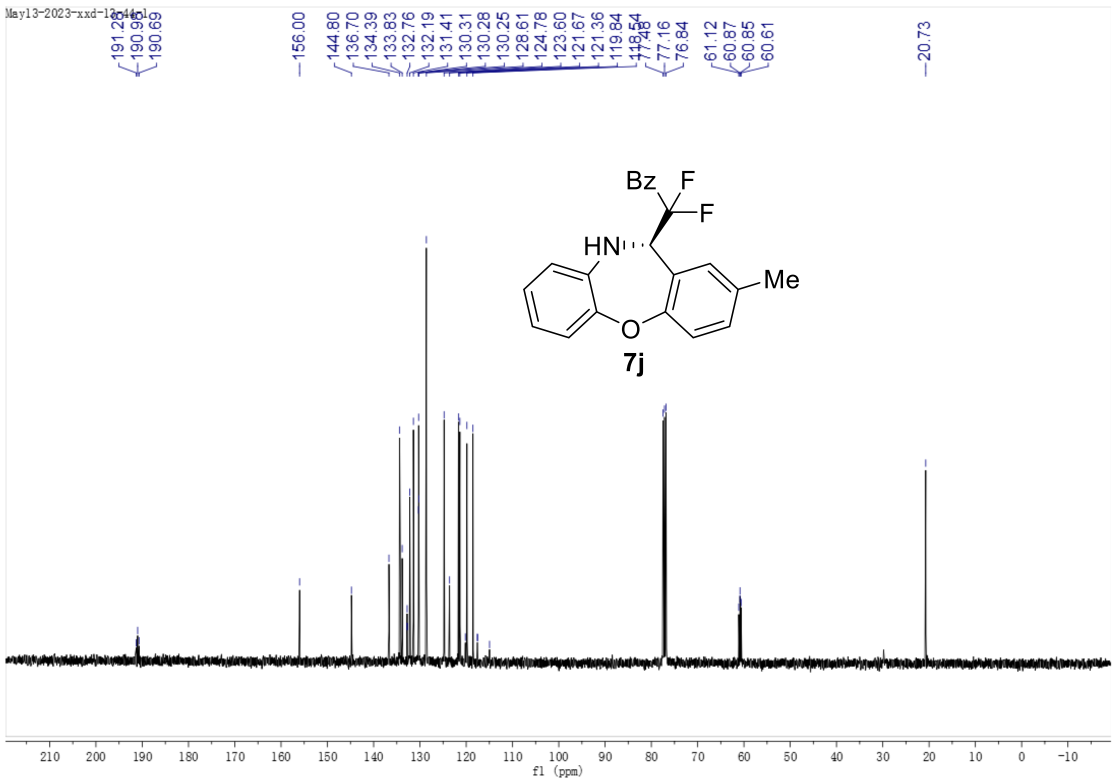

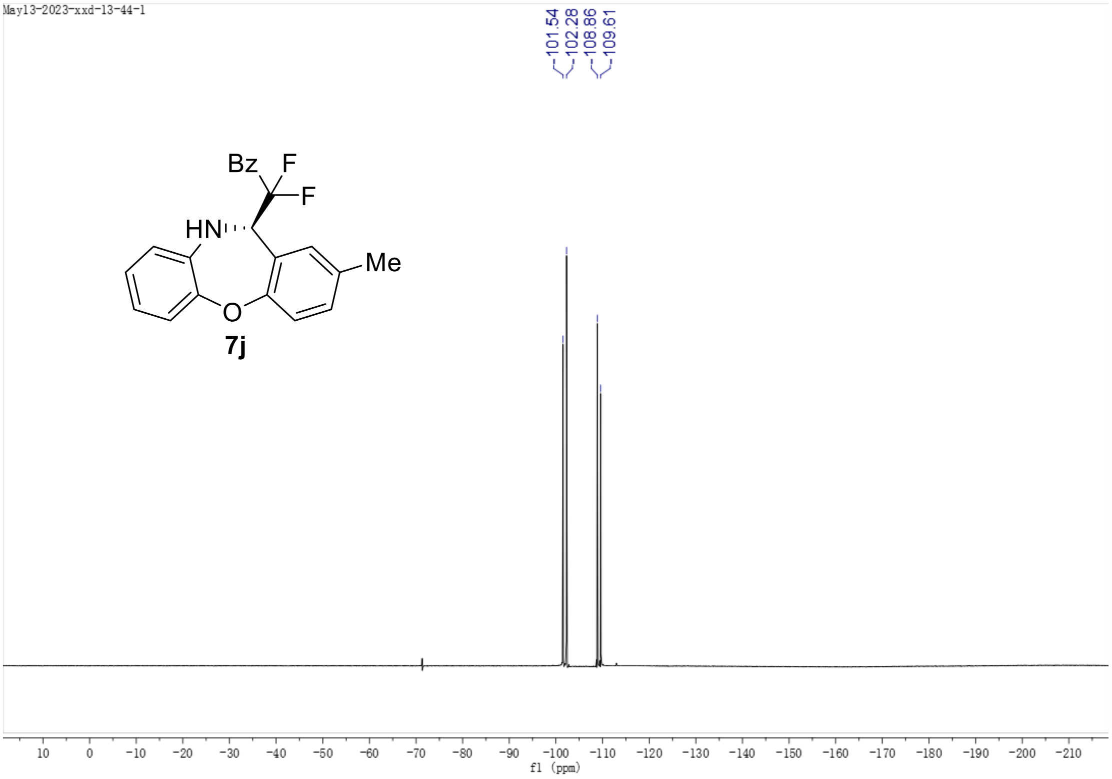

May13-2023-xxd-13-44-1
101.54
102.28
108.86
109.61
Bz
F
F
HN
Me
O
7j
10 0 −10 −20 −30 −40 −50 −60 −70 −80 −90 −100 −110 −120 −130 −140 −150 −160 −170 −180 −190 −200 −210
f1 (ppm)

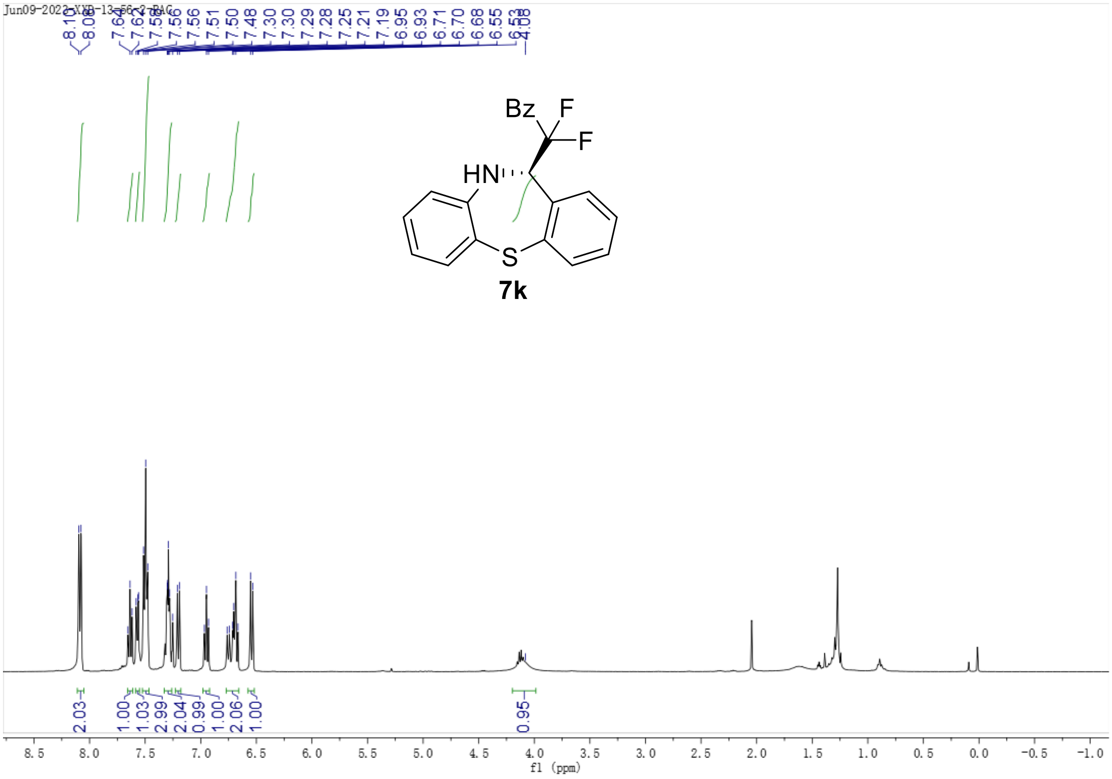

8.10
7.56
7.51
7.50
7.48
7.30
7.30
7.29
7.28
7.25
7.21
7.19
6.95
6.93
6.71
6.70
6.68
6.55
6.53
Bz
F
F
HN
S
7k
2.03
1.00
1.03
2.99
2.04
0.99
1.00
2.06
1.00
0.95
8.5 8.0 7.5 7.0 6.5 6.0 5.5 5.0 4.5 4.0 3.5 3.0 2.5 2.0 1.5 1.0 0.5 0.0 −0.5 −1.0
f1 (ppm)

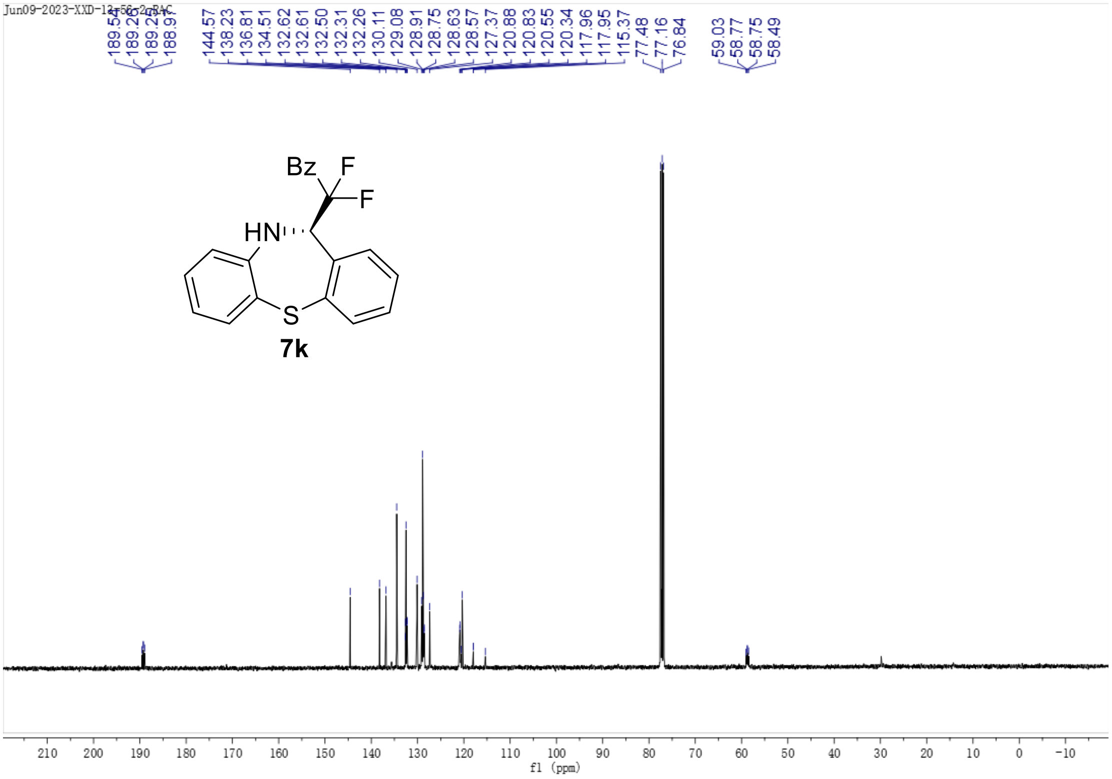
Jun09-2023-XXD-13-56-2-RAC
189.54
189.26
189.24
188.97
144.57
138.23
136.81
134.51
132.62
132.61
132.50
132.31
132.26
130.11
129.08
128.91
128.75
128.63
128.57
127.37
120.88
120.83
120.55
120.34
117.96
117.95
115.37
77.48
77.16
76.84
59.03
58.77
58.75
58.49
Bz
F
F
HN
S
7k
210 200 190 180 170 160 150 140 130 120 110 100 90 80 70 60 50 40 30 20 10 0 -10
f1 (ppm)

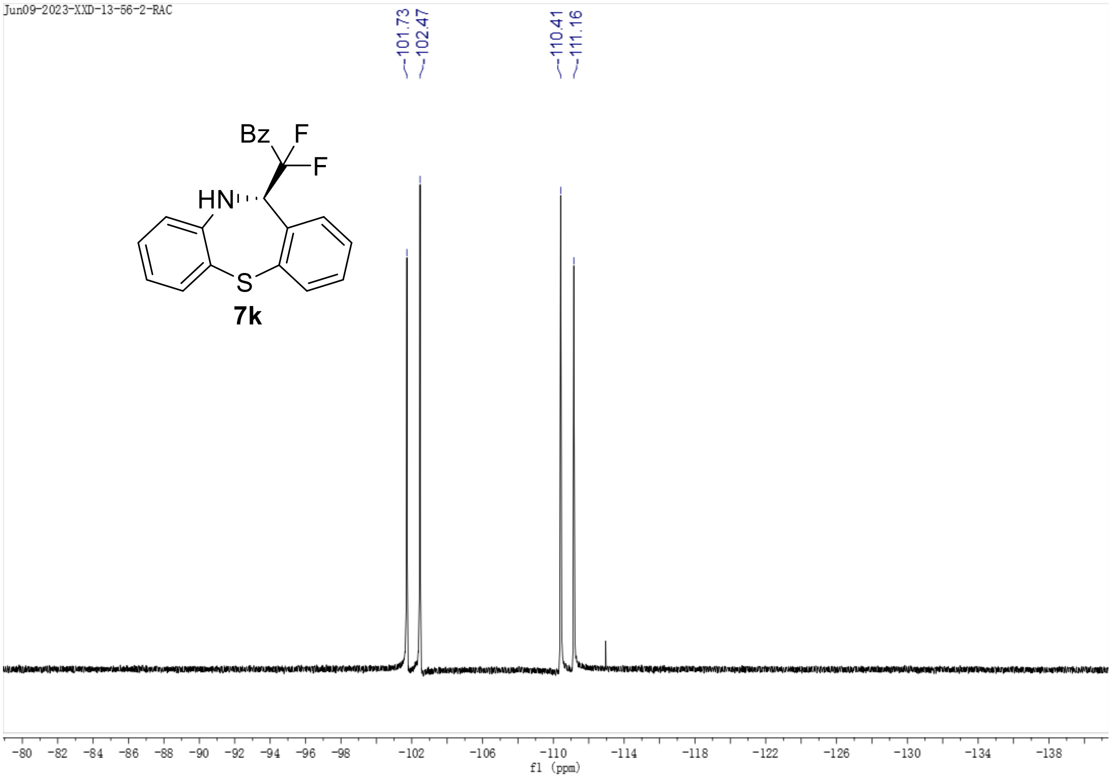
Jun09-2023-XXD-13-56-2-RAC
-101.73
-102.47
-110.41
-111.16
Bz
F
F
HN
S
7k
-80 -82 -84 -86 -88 -90 -92 -94 -96 -98 -102 -106 -110 -114 -118 -122 -126 -130 -134 -138
f1 (ppm)

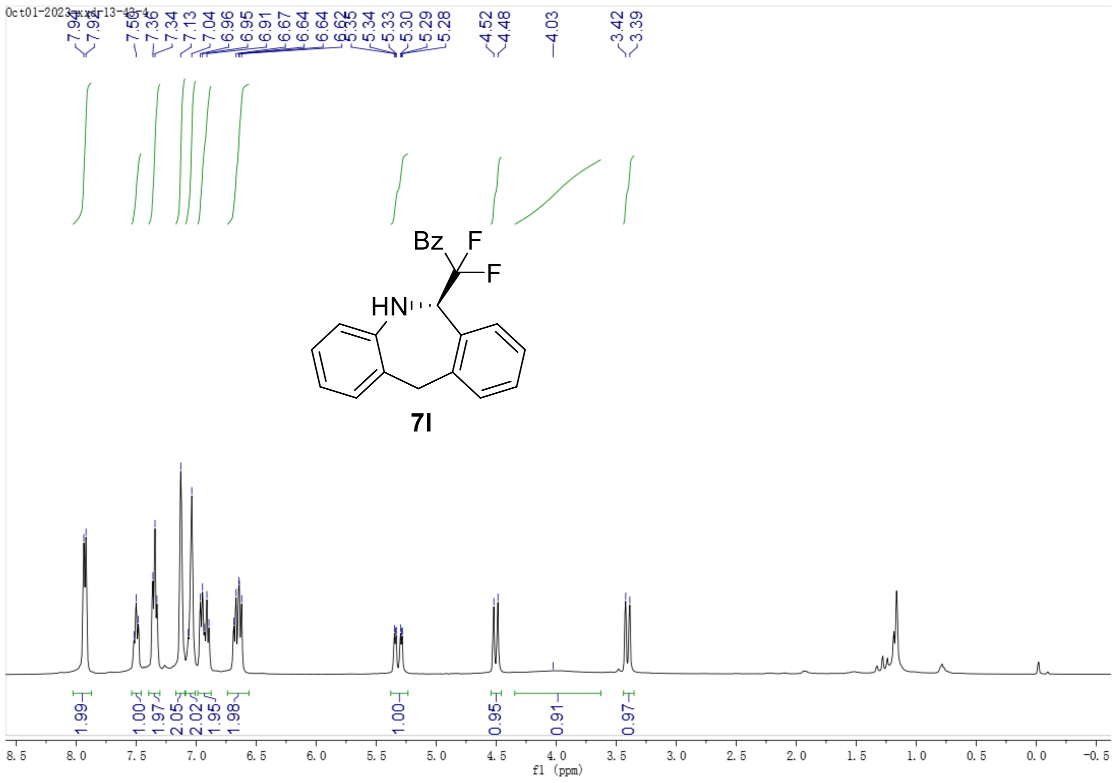

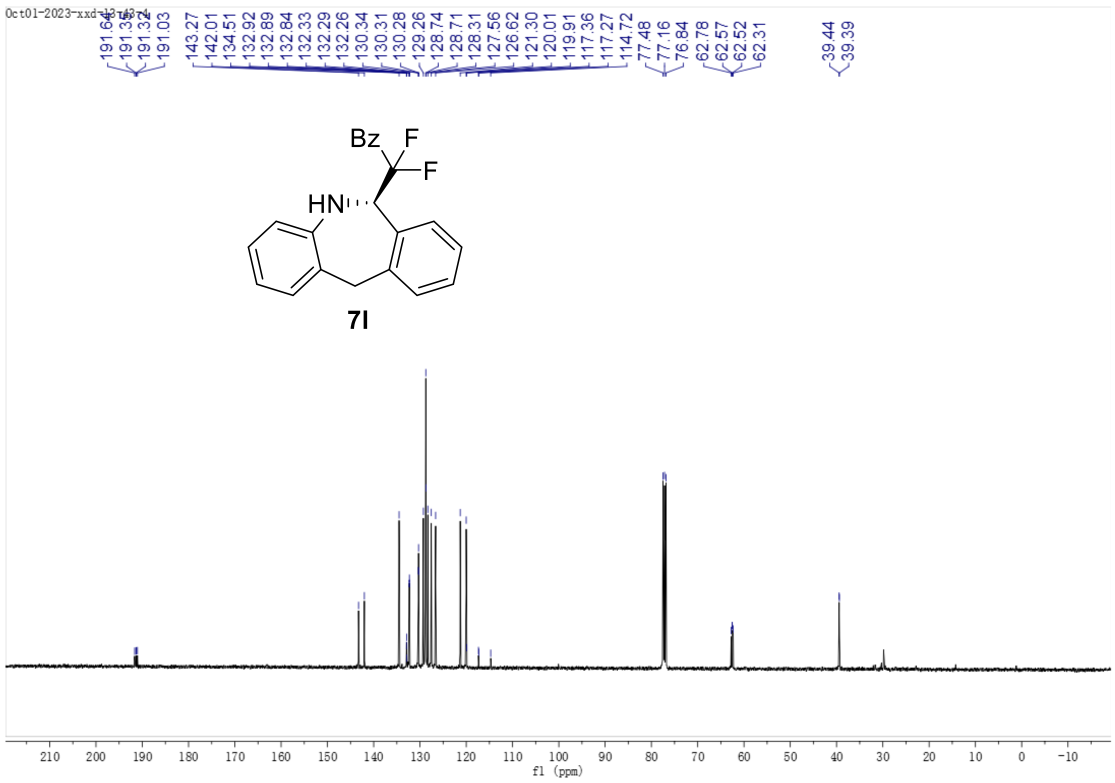

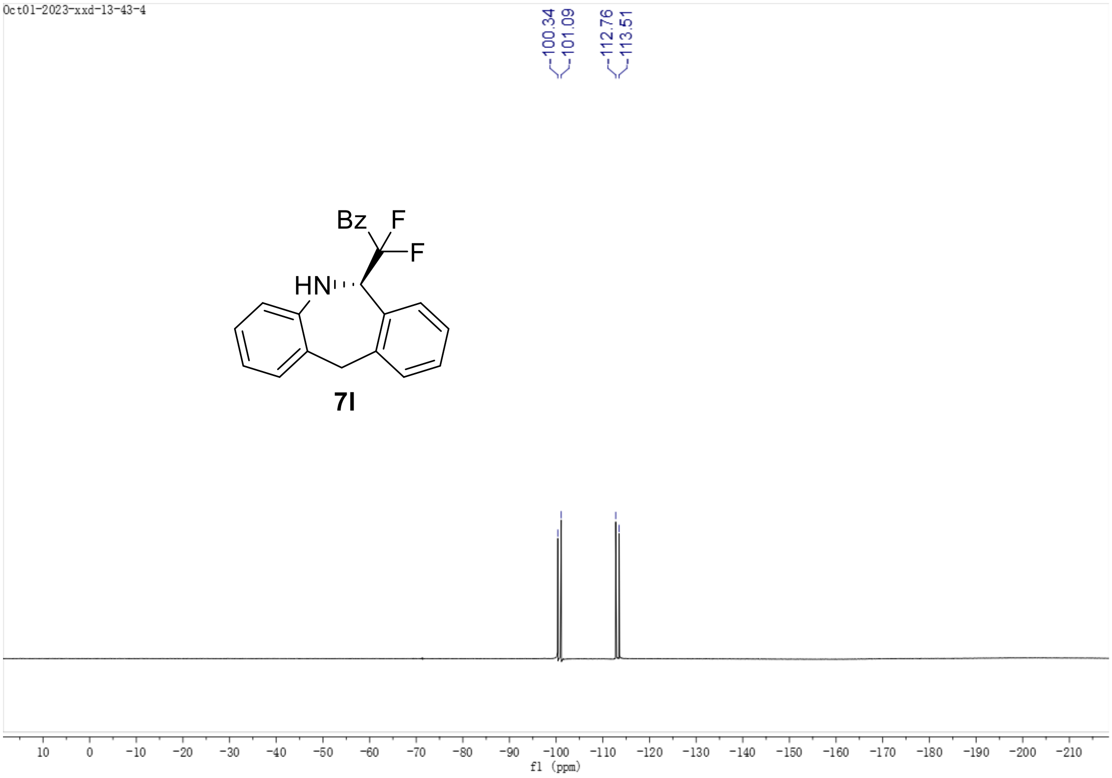
Oct01-2023-xxd-13-43-4
100.34
101.09
112.76
113.51
Bz
F
F
HN
7l
f1 (ppm)

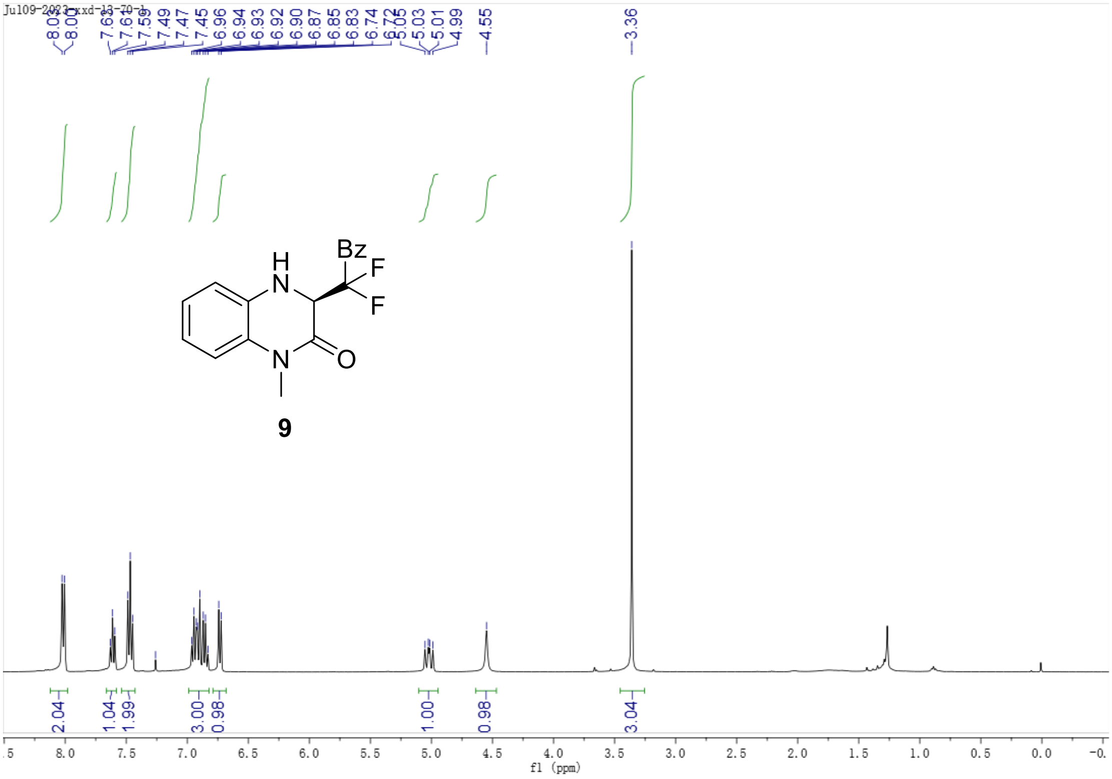
8.03
8.01
7.63
7.61
7.59
7.49
7.47
7.45
6.96
6.94
6.93
6.92
6.90
6.87
6.85
6.83
6.74
6.72
5.05
5.03
5.01
4.99
4.55
3.36
Bz
H
N
F
F
N
O
9
2.04
1.04
1.99
3.00
0.98
1.00
0.98
3.04
f1 (ppm)

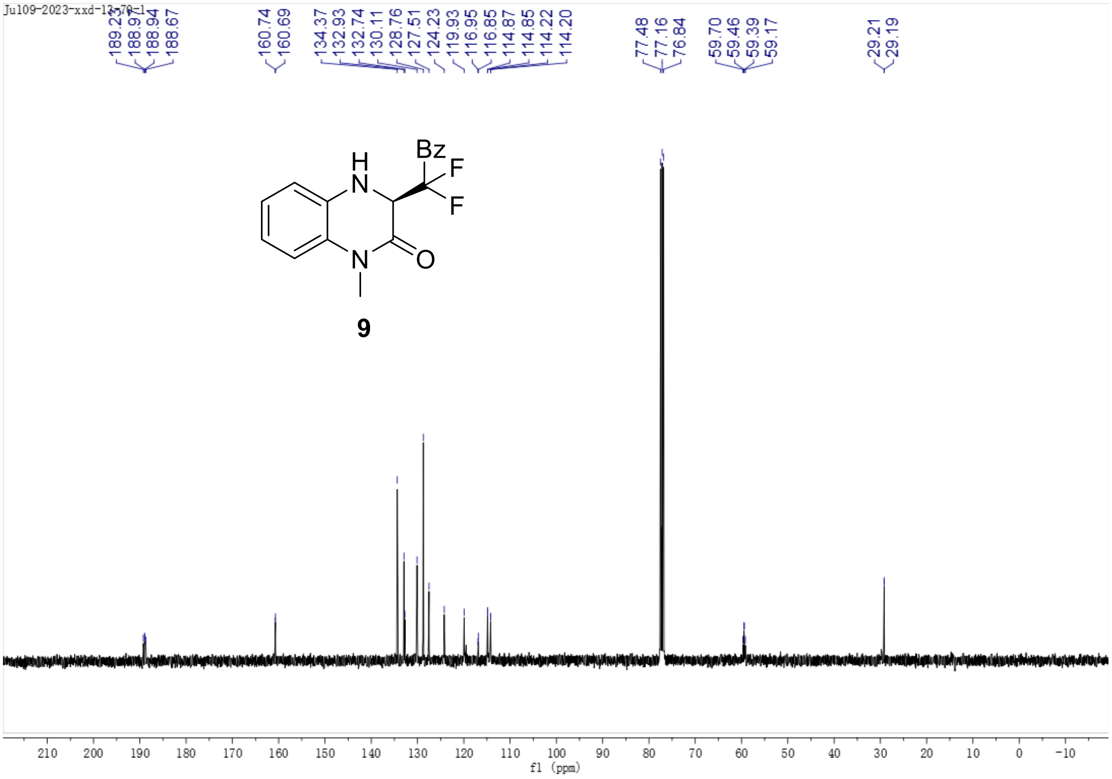
Ju109-2023-xxd-13-70-1
189.22
188.97
188.94
188.67
160.74
160.69
134.37
132.93
132.74
130.11
128.76
127.51
124.23
119.93
116.95
116.85
114.87
114.85
114.22
114.20
77.48
77.16
76.84
59.70
59.46
59.39
59.17
29.21
29.19
Bz
F
F
H
N
N
O
9
210 200 190 180 170 160 150 140 130 120 110 100 90 80 70 60 50 40 30 20 10 0 -10
f1 (ppm)

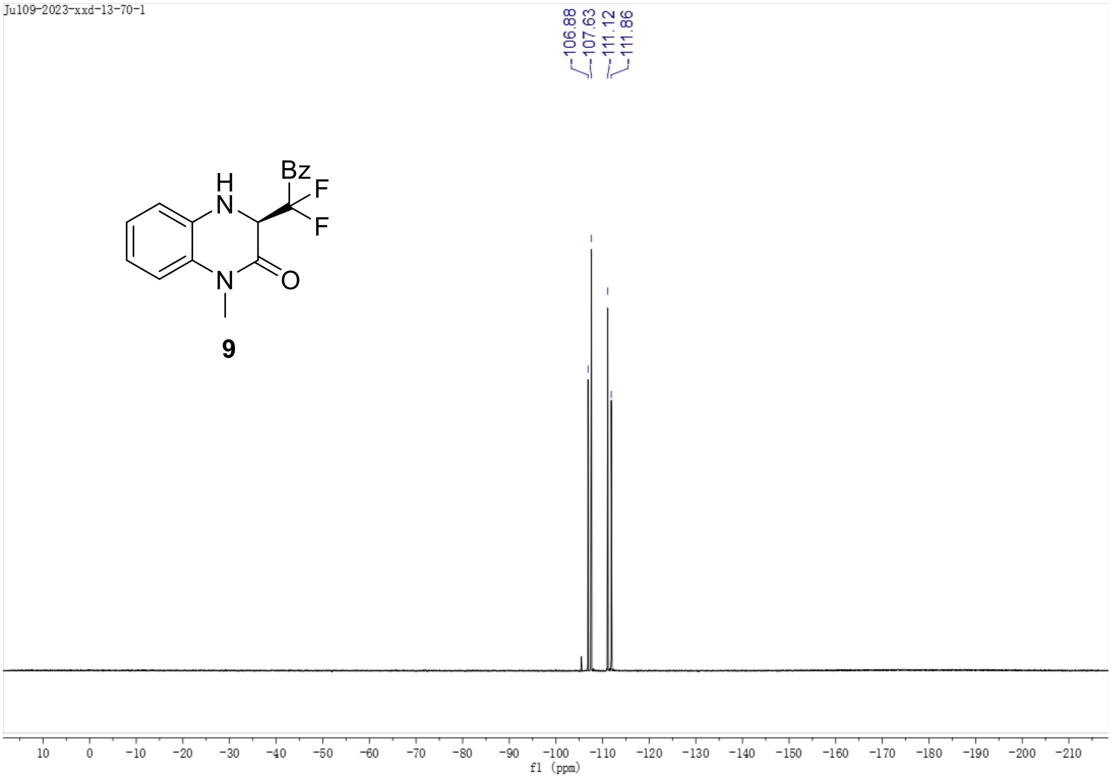
Ju109-2023-xxd-13-70-1
-106.88
-107.63
-111.12
-111.86
Bz
F
F
H
N
N
O
9
10 0 -10 -20 -30 -40 -50 -60 -70 -80 -90 -100 -110 -120 -130 -140 -150 -160 -170 -180 -190 -200 -210
f1 (ppm)

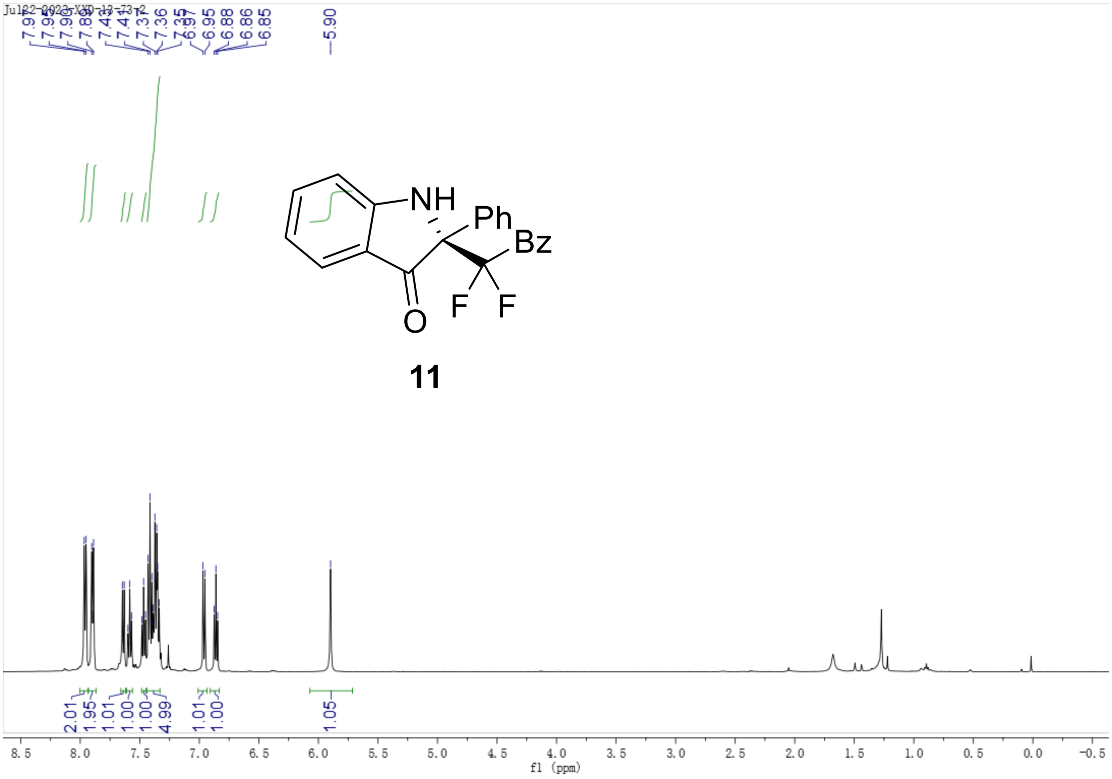

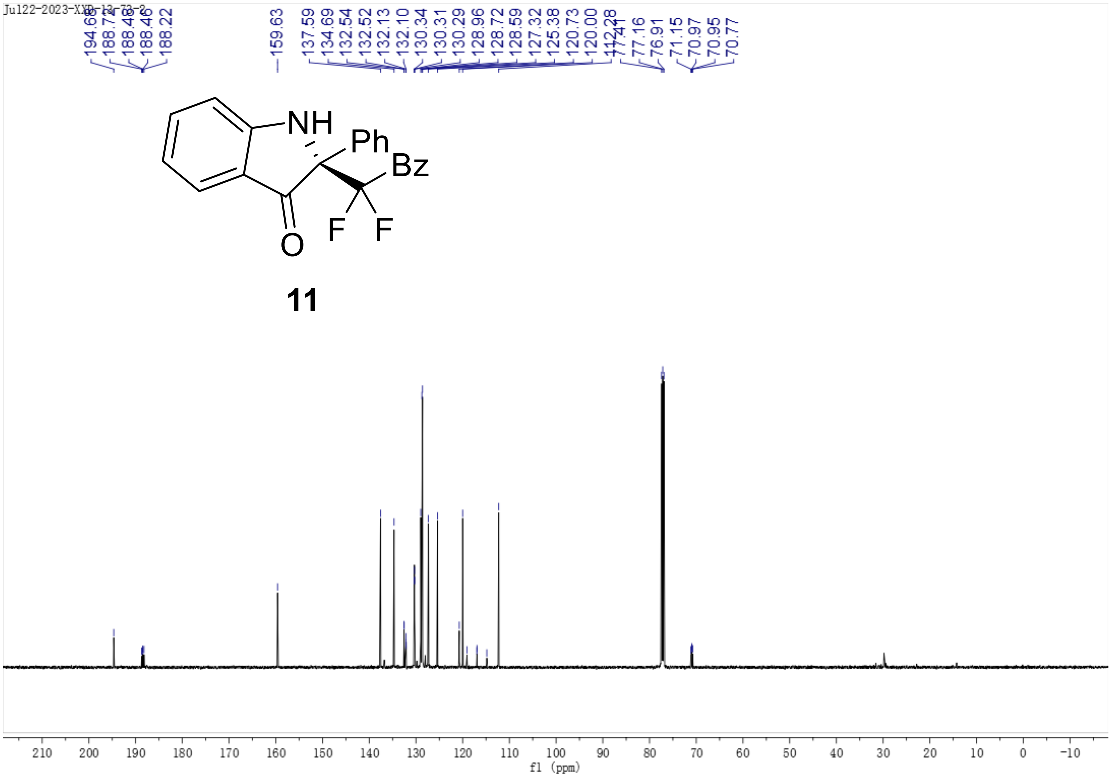

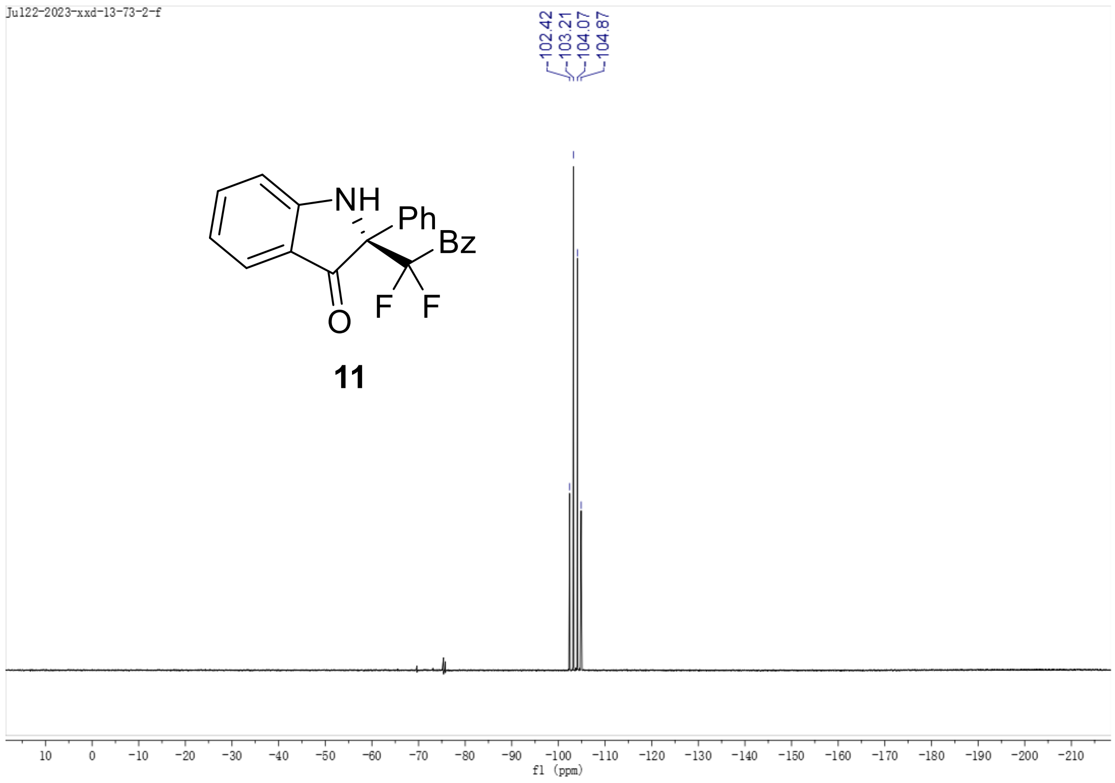

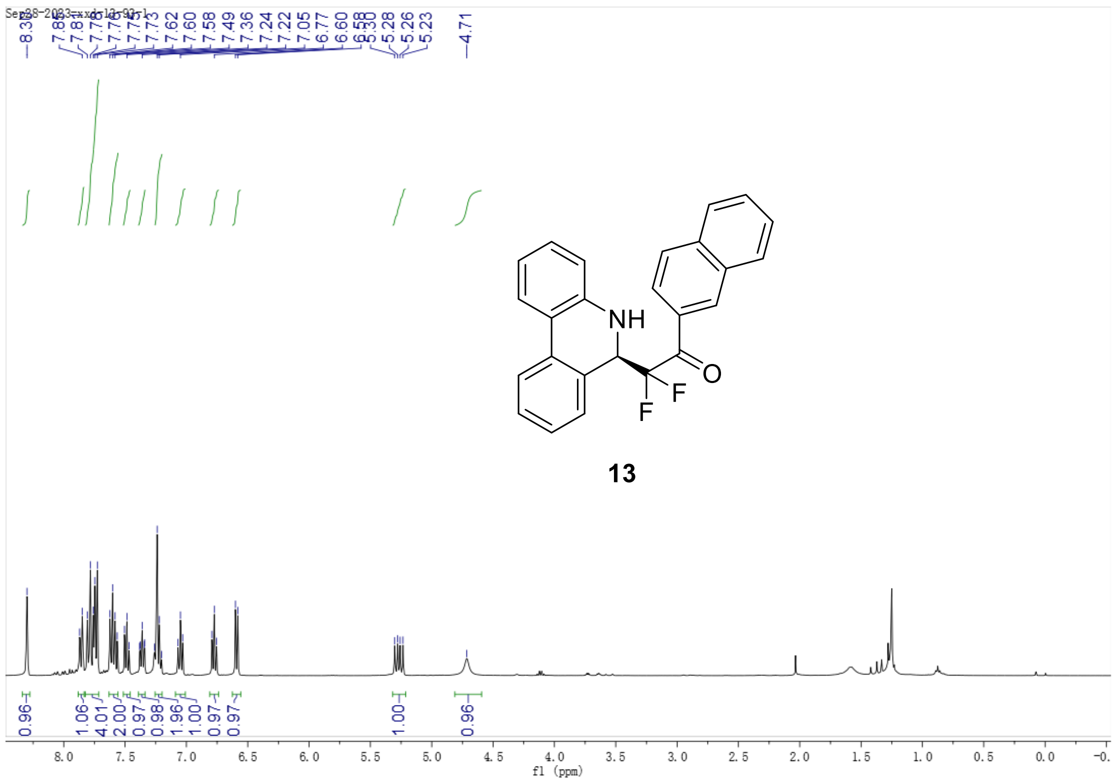

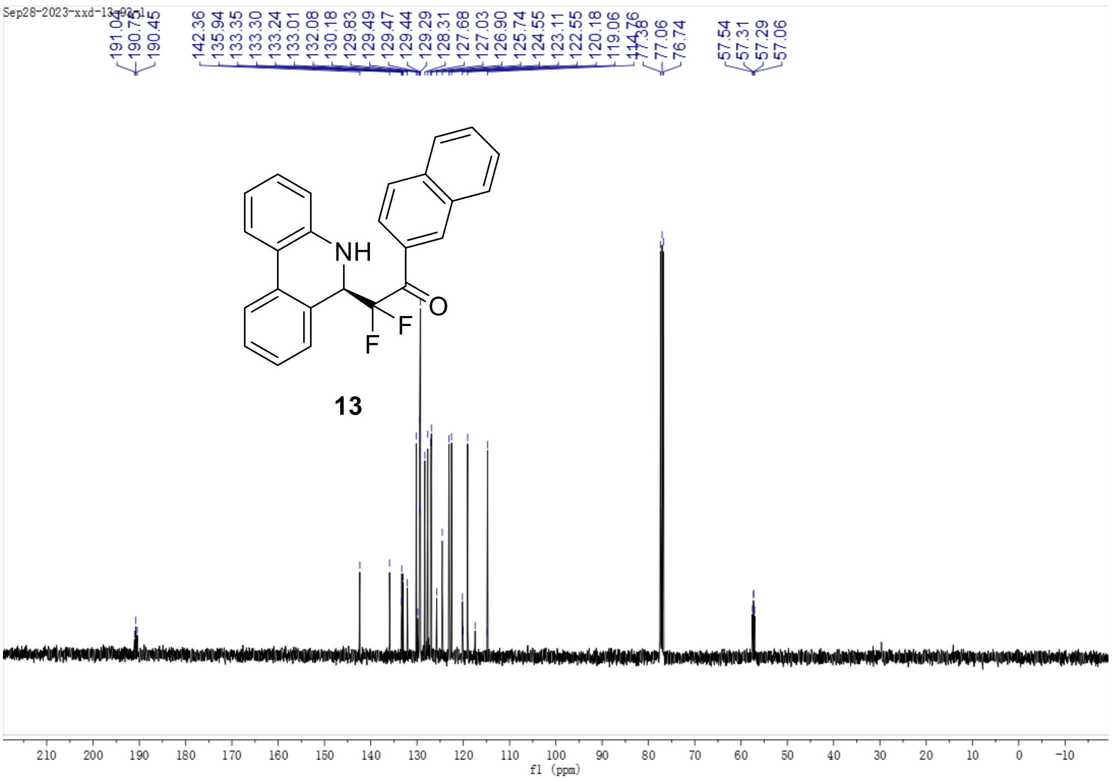
Sep28-2023-xxd-13-93-1
191.04
190.75
190.45
142.36
135.94
133.35
133.30
133.24
133.01
132.08
130.18
129.83
129.49
129.47
129.44
129.29
128.31
127.68
127.03
126.90
125.74
124.55
123.11
122.55
120.18
119.06
114.76
77.38
77.06
76.74
57.54
57.31
57.29
57.06
NH
O
F
F
13
210
200
190
180
170
160
150
140
130
120
110
100
90
80
70
60
50
40
30
20
10
0
-10
f1 (ppm)

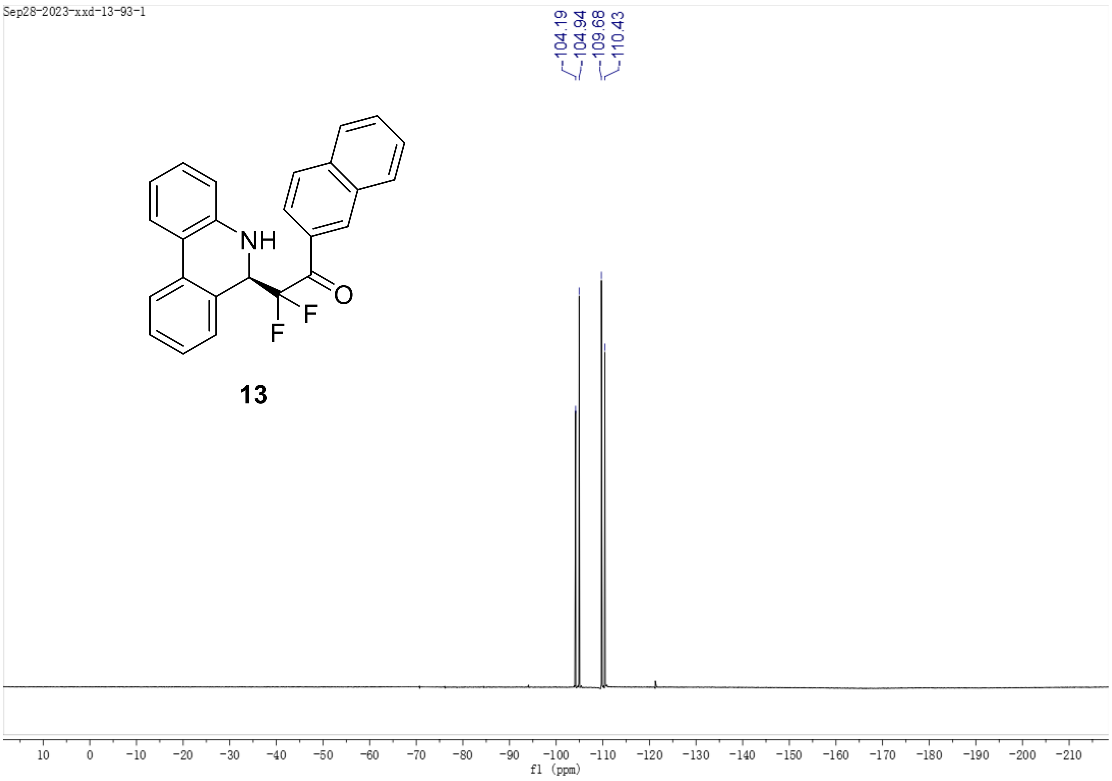
Sep28-2023-xxd-13-93-1
-104.19
-104.94
-109.68
-110.43
NH
O
F
F
13
10
0
-10
-20
-30
-40
-50
-60
-70
-80
-90
-100
-110
-120
-130
-140
-150
-160
-170
-180
-190
-200
-210
f1 (ppm)

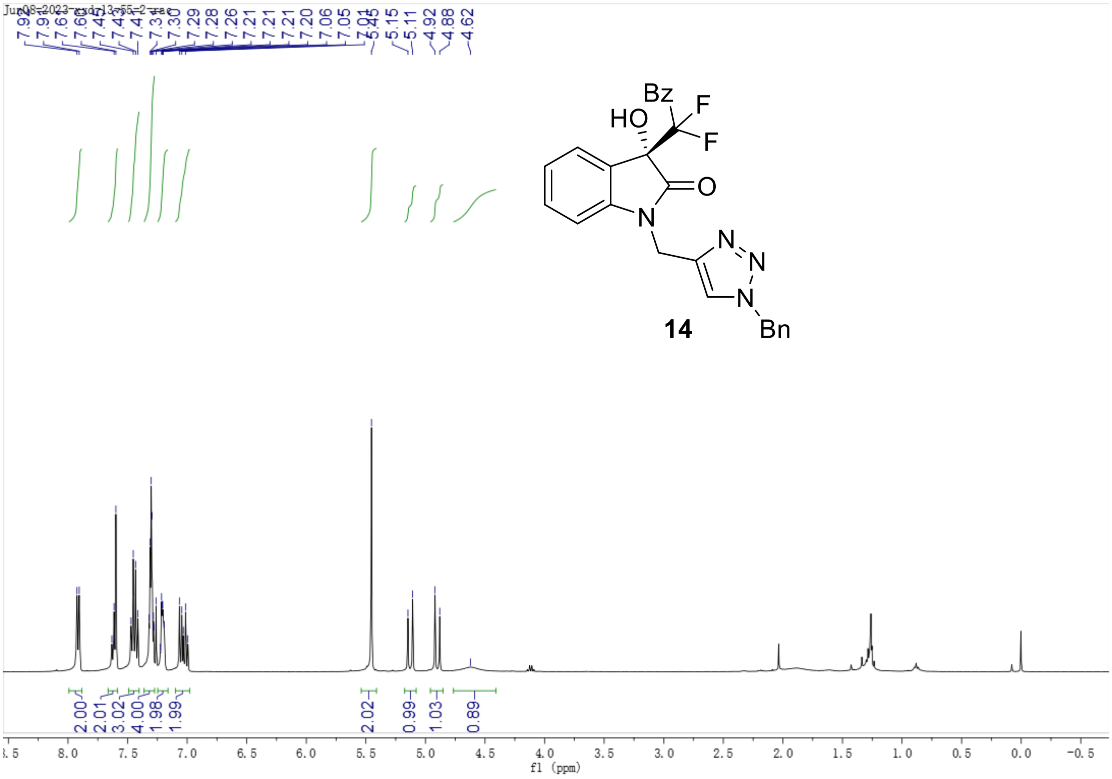
Jun08-2023-xxd-13-55-2-rac
5.45
5.15
5.11
4.92
4.88
4.62
2.00
2.01
3.02
4.00
1.98
1.99
2.02
0.99
1.03
0.89
Bz
HO
F
F
O
N
N
N
N
Bn
14
f1 (ppm)

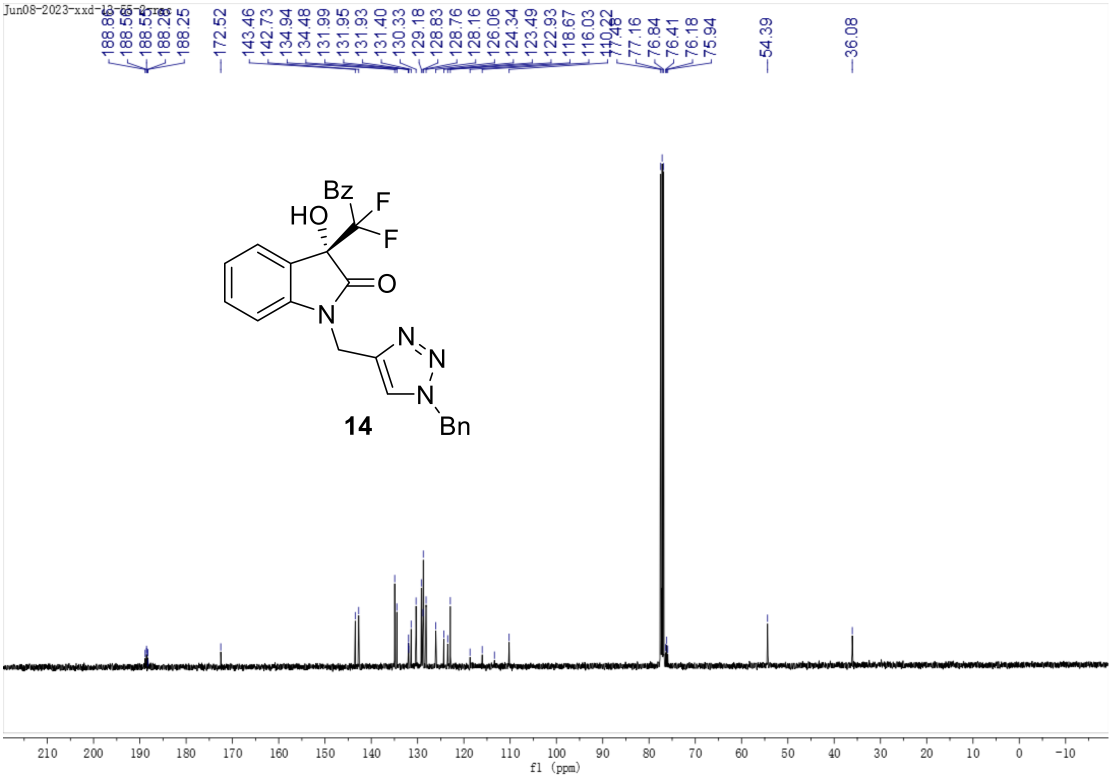
Jun08-2023-xxd-13-55-2-rac
172.52
143.46
142.73
134.94
134.48
131.99
131.95
131.93
131.40
130.33
129.18
128.83
128.76
128.16
126.06
124.34
123.49
122.93
118.67
116.03
77.16
76.84
76.41
76.18
75.94
54.39
36.08
Bz
HO
F
F
O
N
N
N
N
Bn
14
f1 (ppm)

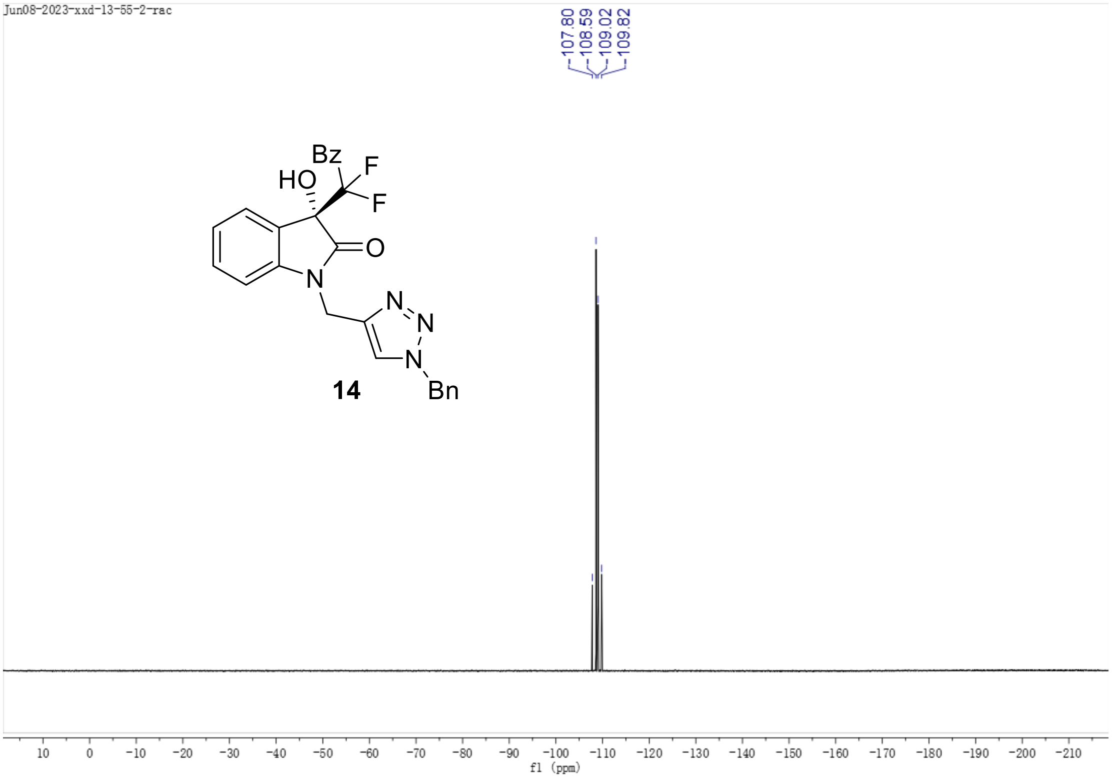
Jun08-2023-xxd-13-55-2-rac
-107.80
-108.59
-109.02
-109.82
Bz
HO
F
F
O
N
N
N
N
14
Bn
10 0 -10 -20 -30 -40 -50 -60 -70 -80 -90 -100 -110 -120 -130 -140 -150 -160 -170 -180 -190 -200 -210
f1 (ppm)

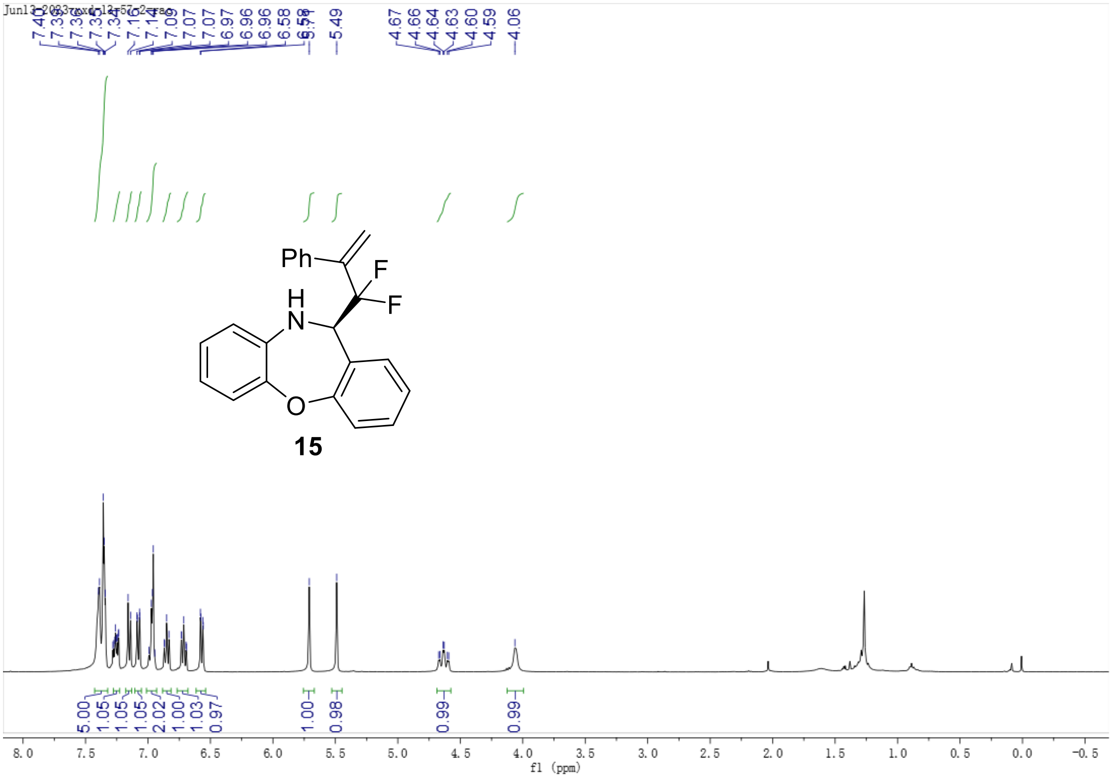
7.15
7.09
7.07
7.07
6.97
6.96
6.96
6.58
5.49
4.67
4.66
4.64
4.63
4.60
4.59
4.06
Ph
F
F
H
N
O
15
5.00
1.05
1.05
1.05
2.02
1.00
1.03
0.97
1.00
0.98
0.99
0.99
8.0 7.5 7.0 6.5 6.0 5.5 5.0 4.5 4.0 3.5 3.0 2.5 2.0 1.5 1.0 0.5 0.0 -0.5
f1 (ppm)

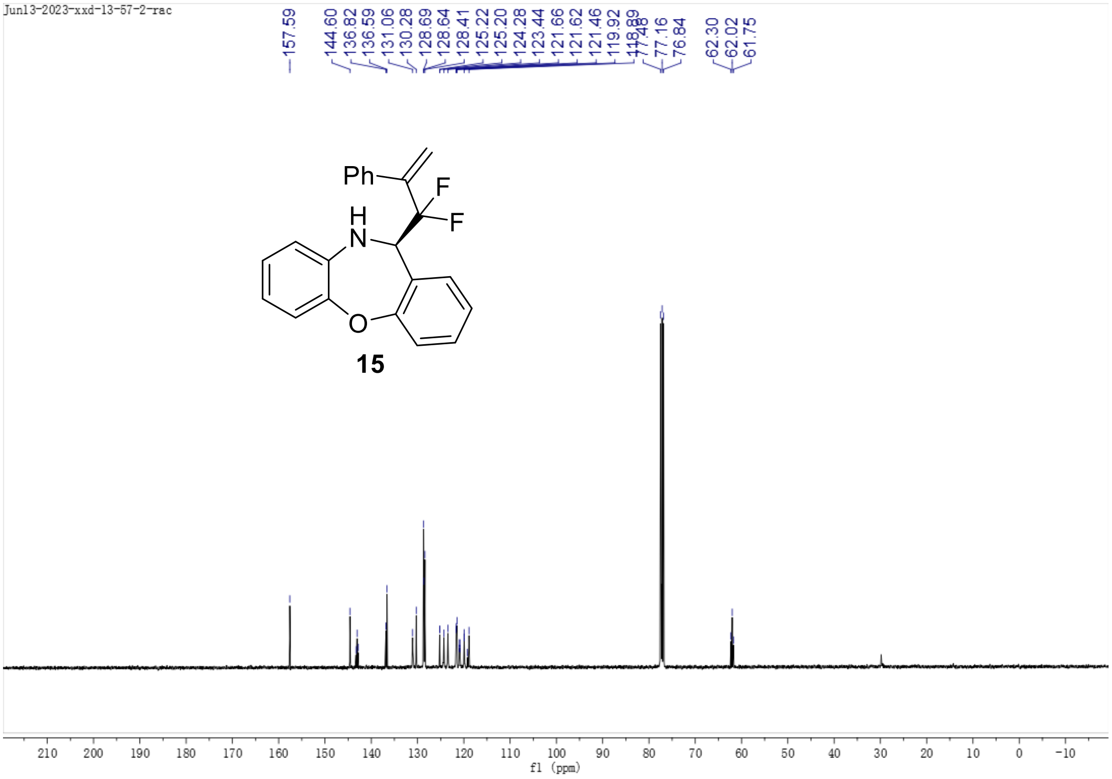
Jun13-2023-xxd-13-57-2-rac
157.59
144.60
136.82
136.59
131.06
130.28
128.69
128.64
128.41
125.22
125.20
124.28
123.44
121.66
121.62
121.46
119.92
118.89
77.48
77.16
76.84
62.30
62.02
61.75
Ph
H
N
F
F
O
15
f1 (ppm)

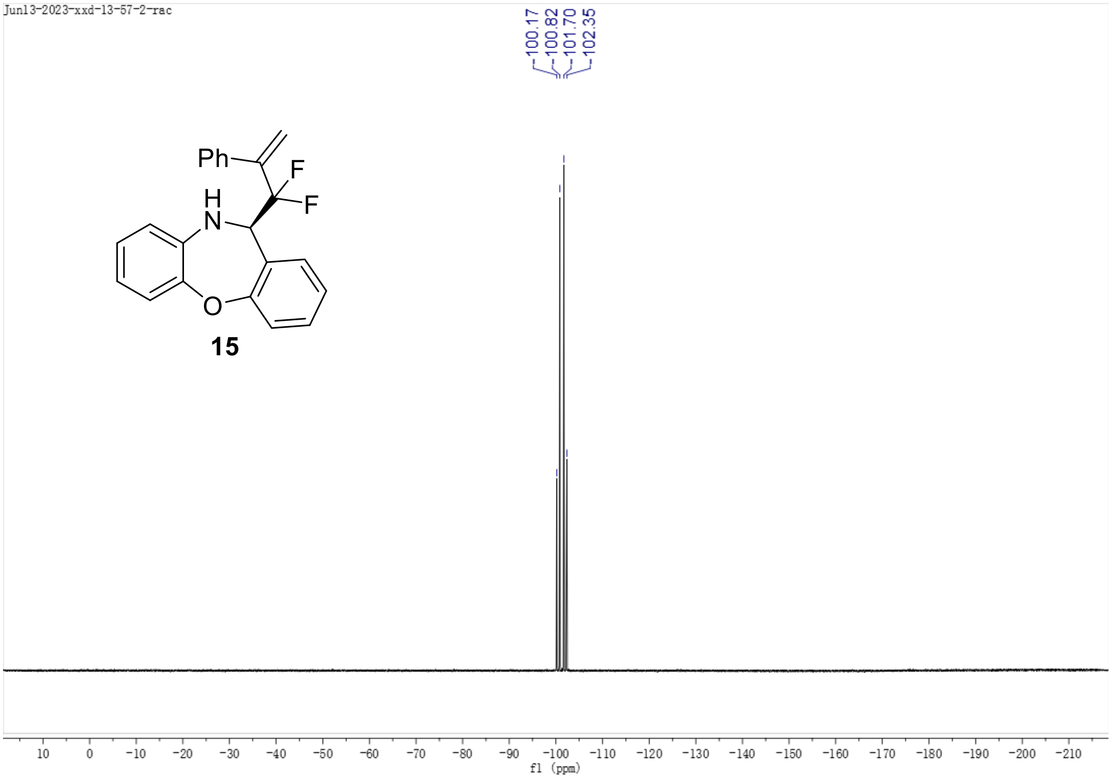
Jun13-2023-xxd-13-57-2-rac
-100.17
-100.82
-101.70
-102.35
Ph
H
N
F
F
O
15
f1 (ppm)

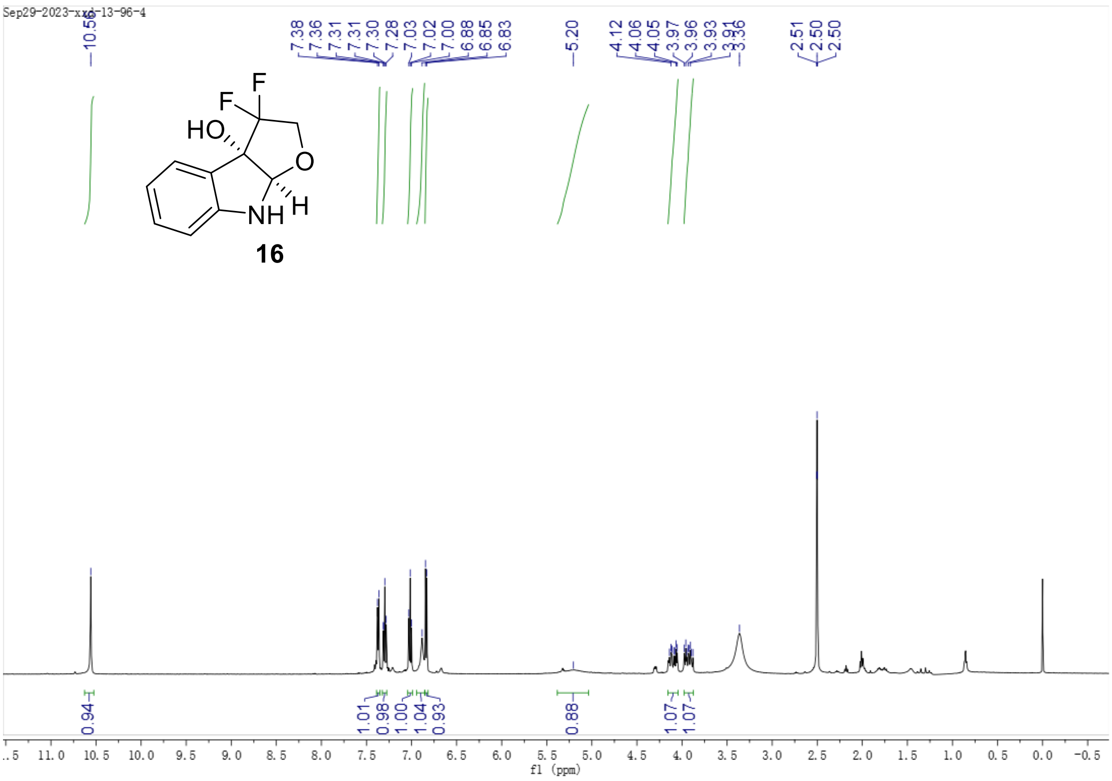
Sep29-2023-xxd-13-96-4
16
f1 (ppm)

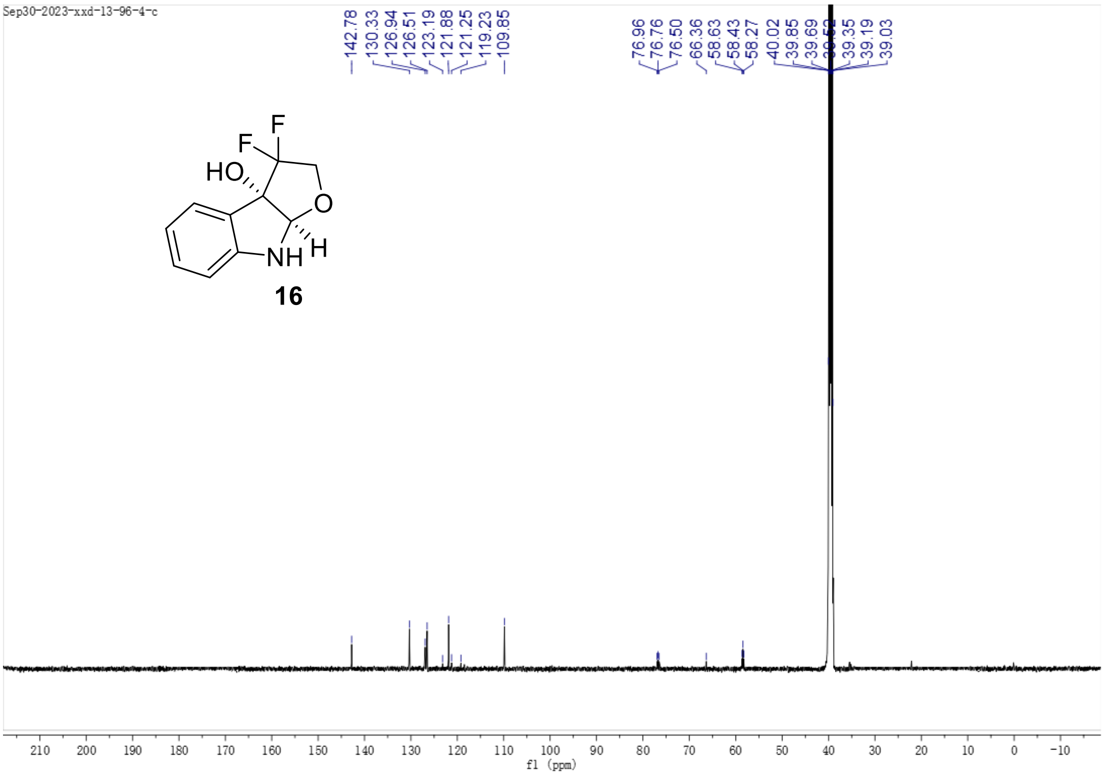
Sep30-2023-xxd-13-96-4-c
16
f1 (ppm)

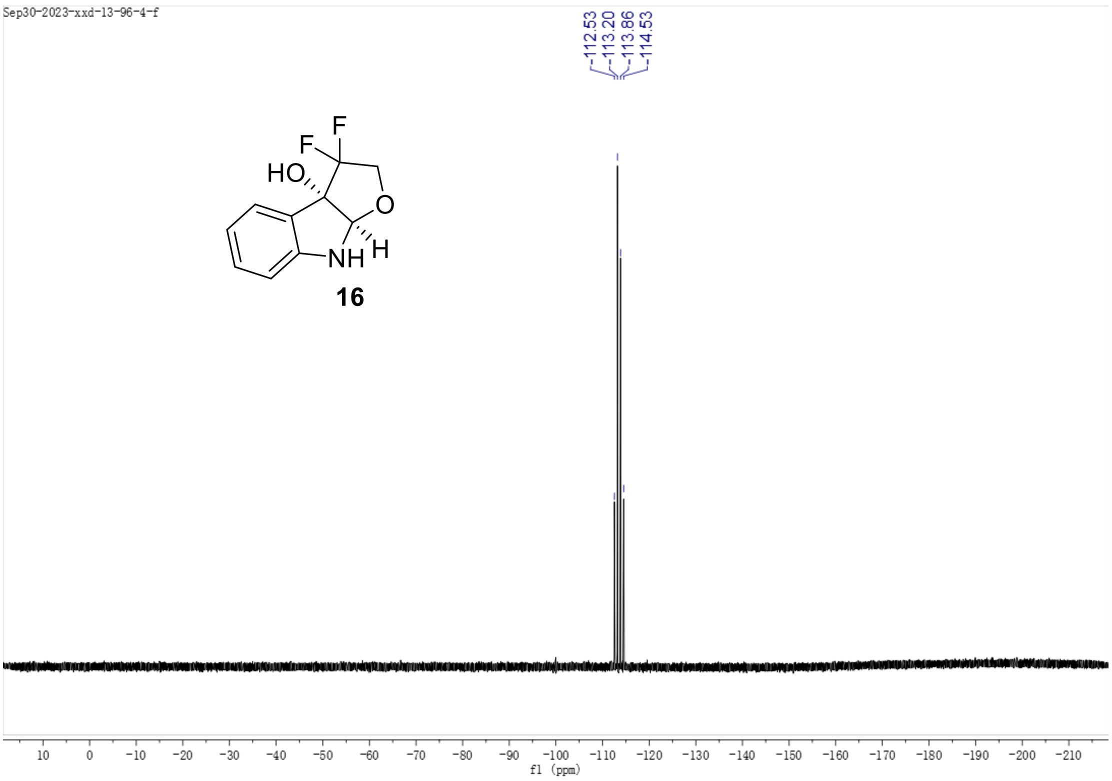
Sep30-2023-xxd-13-96-4-f
-112.53
-113.20
-113.86
-114.53
F
F
HO
O
NH
H
fl (ppm)

16

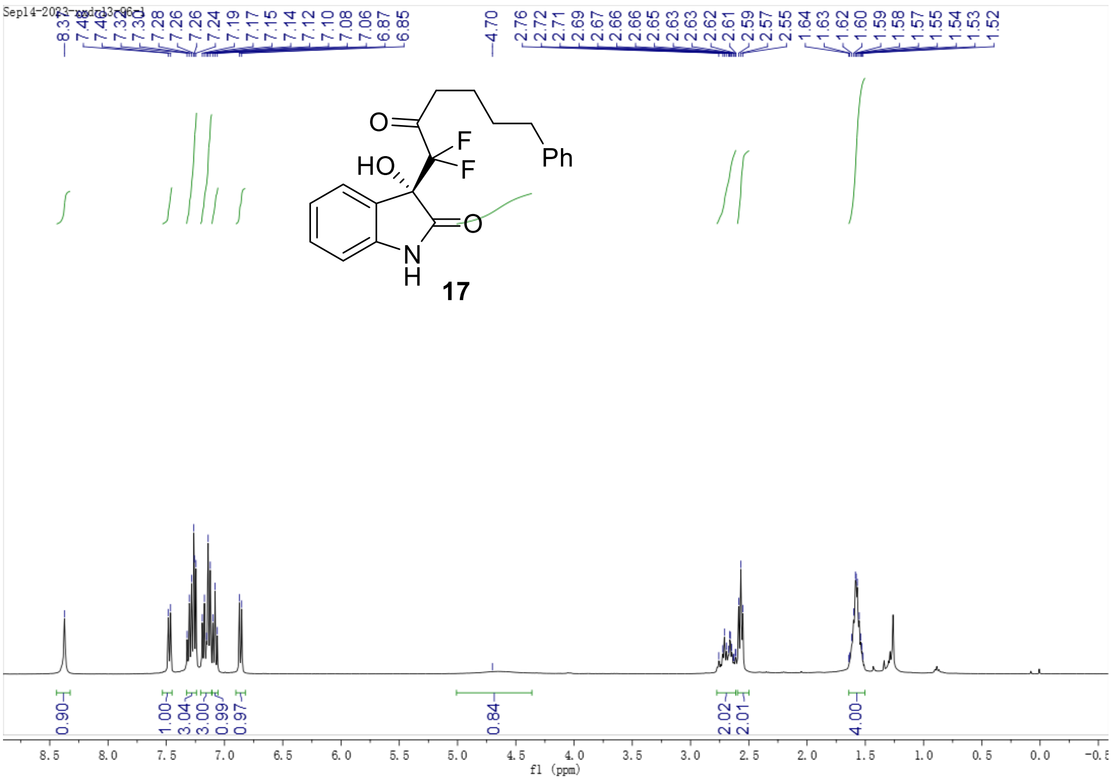
O
F
F
HO
Ph
O
N
H
0.90
1.00
3.04
3.00
0.99
0.97
0.84
2.02
2.01
4.00
fl (ppm)

17

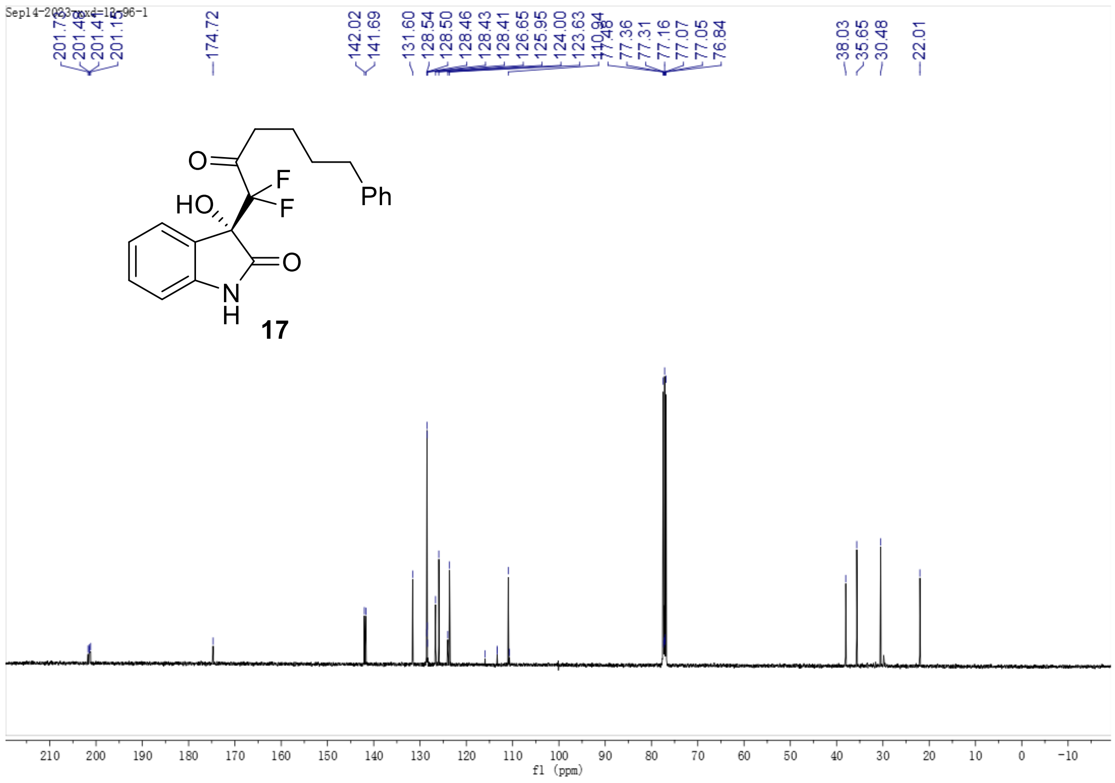

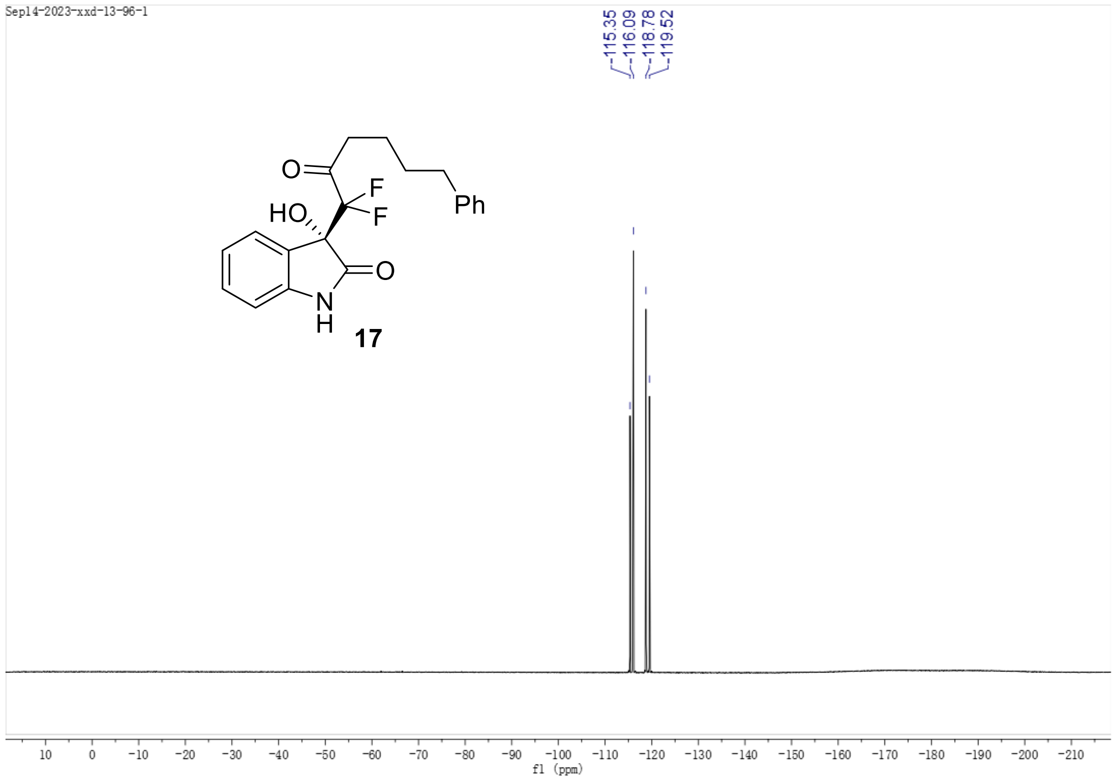

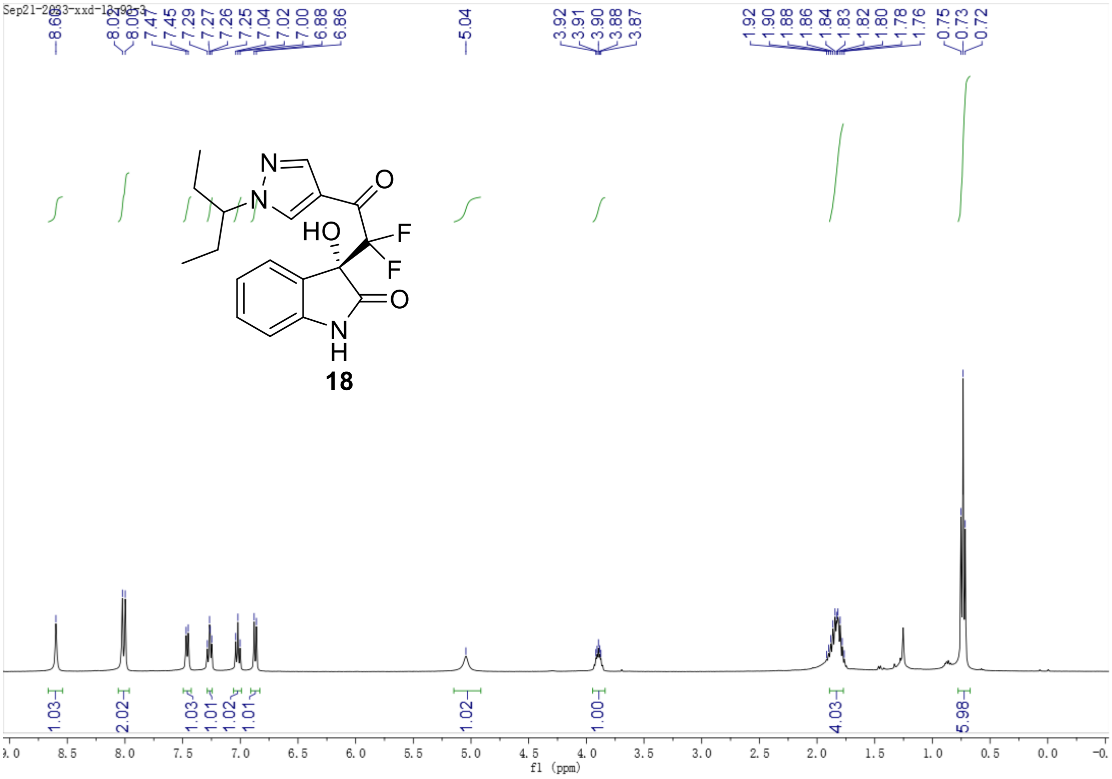
Sep21-2023-xxd-13-93-3
8.63
8.02
8.01
7.47
7.45
7.29
7.27
7.26
7.25
7.04
7.02
7.00
6.88
6.86
5.04
3.92
3.91
3.90
3.88
3.87
1.92
1.90
1.88
1.86
1.84
1.83
1.82
1.80
1.78
1.76
0.75
0.73
0.72
HO
F
F
O
N
N
O
N
H
18
1.03
2.02
1.03
1.01
1.02
1.01
1.02
1.00
4.03
5.98
f1 (ppm)

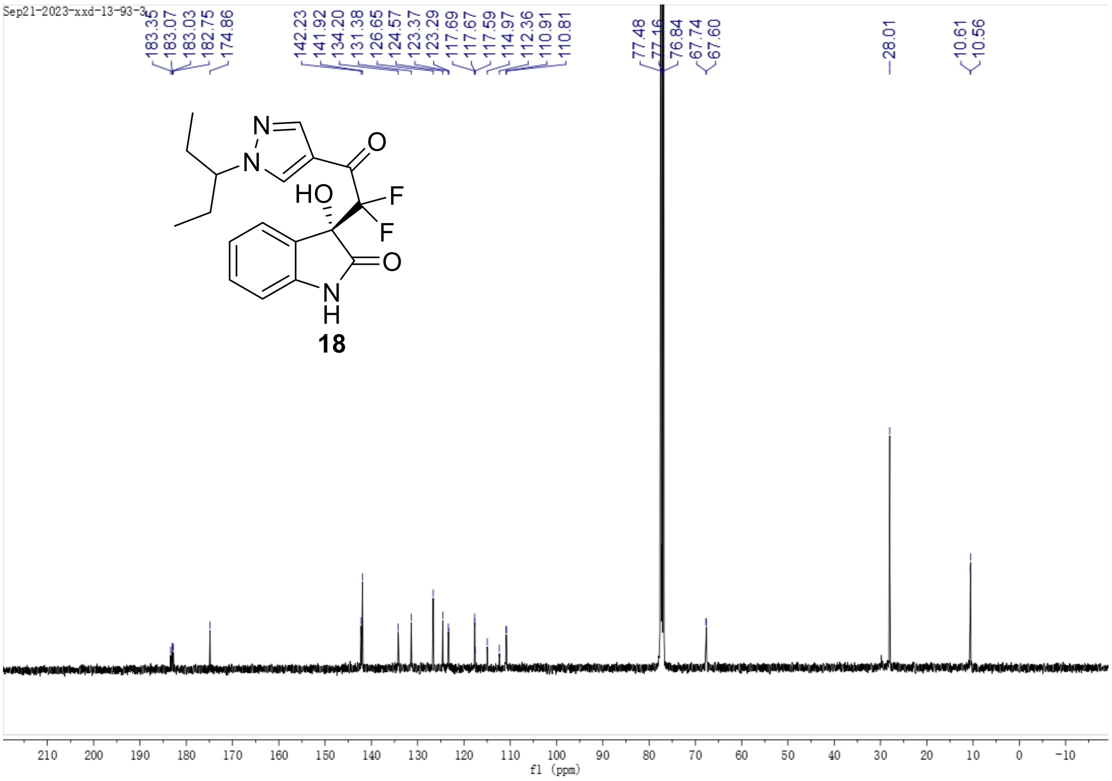
Sep21-2023-xxd-13-93-3
183.35
183.07
183.03
182.75
174.86
142.23
141.92
134.20
131.38
126.65
124.57
123.37
123.29
117.69
117.67
117.59
114.97
112.36
110.91
110.81
77.48
77.16
76.84
67.74
67.60
28.01
10.61
10.56
HO
F
F
O
N
N
O
N
H
18
f1 (ppm)

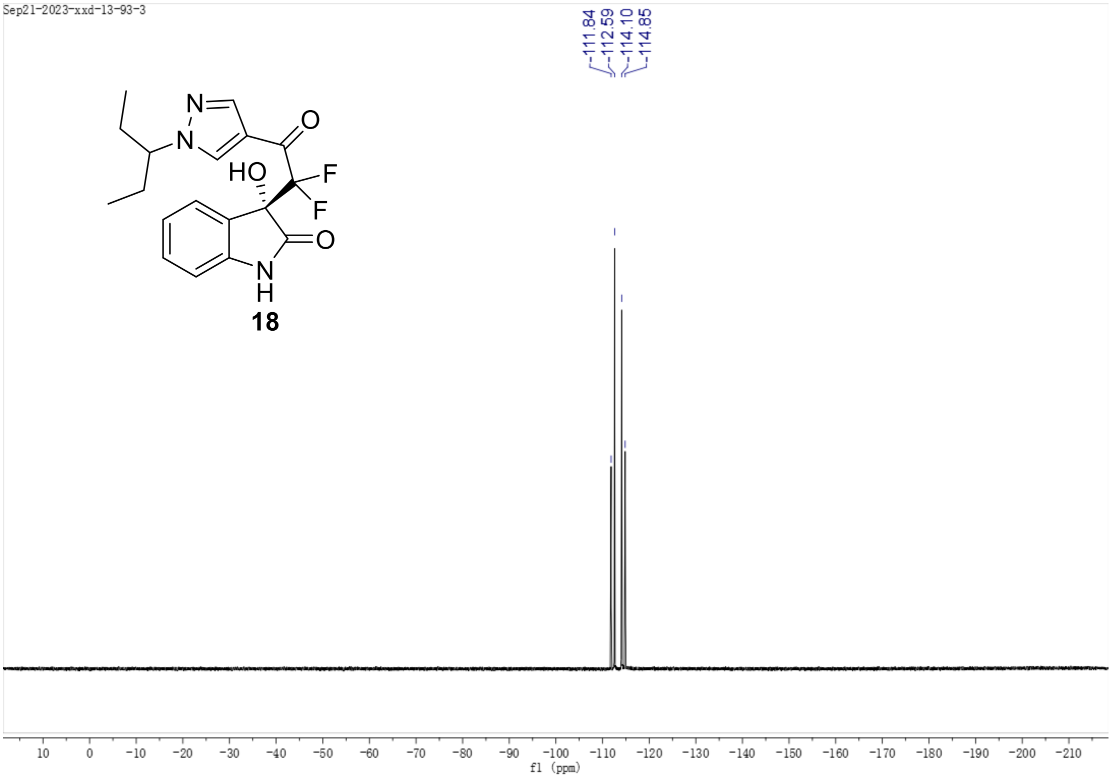
Sep21-2023-xxd-13-93-3
-111.84
-112.59
-114.10
-114.85
HO
F
F
O
O
N
N
N
H
10 0 -10 -20 -30 -40 -50 -60 -70 -80 -90 -100 -110 -120 -130 -140 -150 -160 -170 -180 -190 -200 -210
f1 (ppm)

18

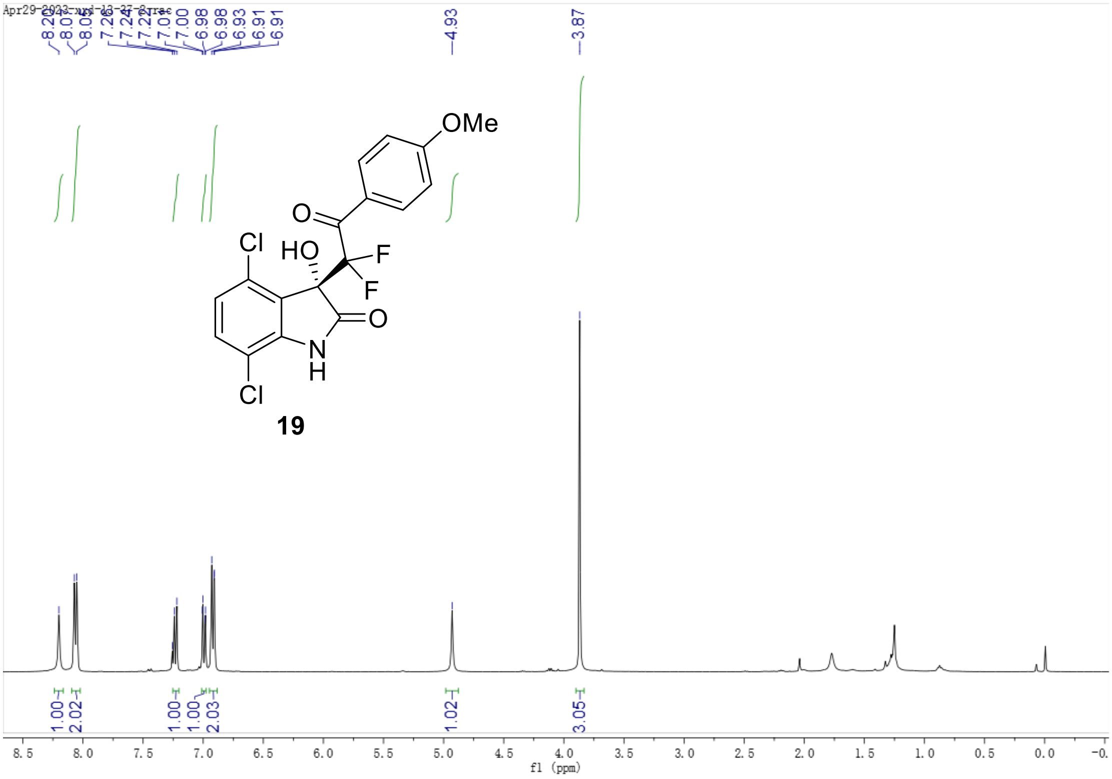
-4.93
-3.87
OMe
O
Cl
HO
F
F
O
N
H
Cl
1.00
2.02
1.00
1.00
2.03
1.02
3.05
8.5 8.0 7.5 7.0 6.5 6.0 5.5 5.0 4.5 4.0 3.5 3.0 2.5 2.0 1.5 1.0 0.5 0.0
f1 (ppm)

19

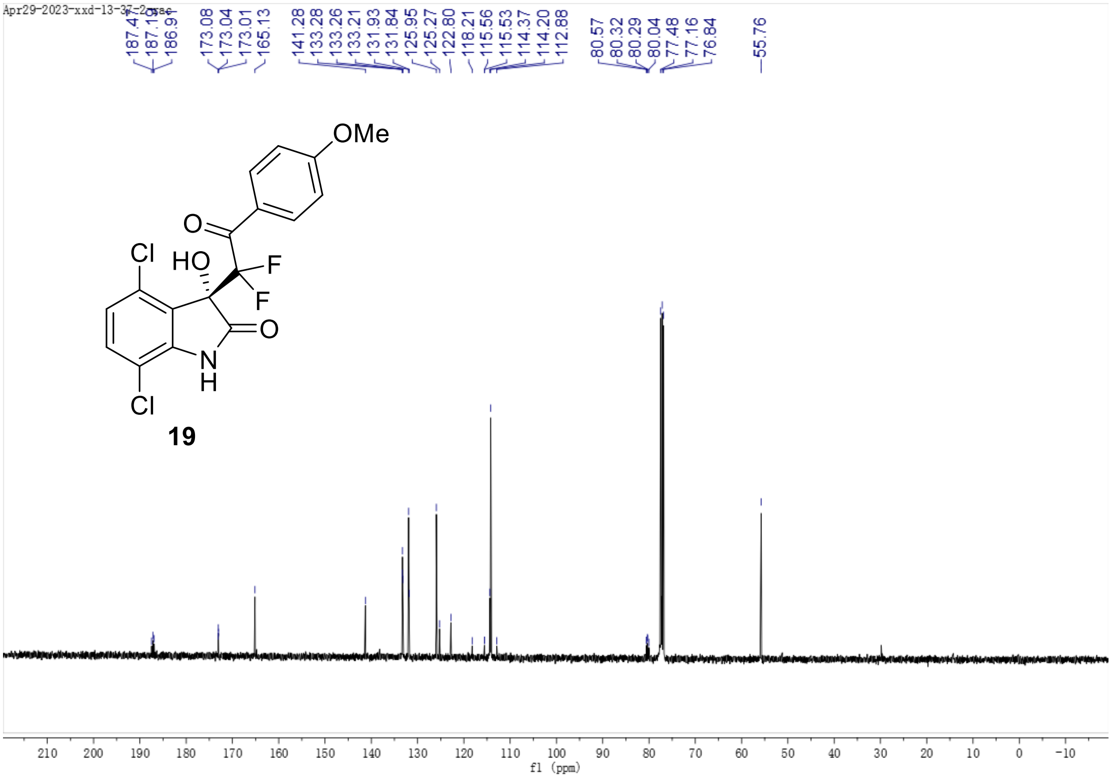
Apr29-2023-xxd-13-37-2-rac
187.41
187.18
186.91
173.08
173.04
173.01
165.13
141.28
133.28
133.26
133.21
131.93
131.84
125.95
125.27
122.80
118.21
115.56
115.53
114.37
114.20
112.88
80.57
80.32
80.29
80.04
77.48
77.16
76.84
55.76
OMe
O
Cl
HO
F
F
O
N
H
Cl
19
f1 (ppm)

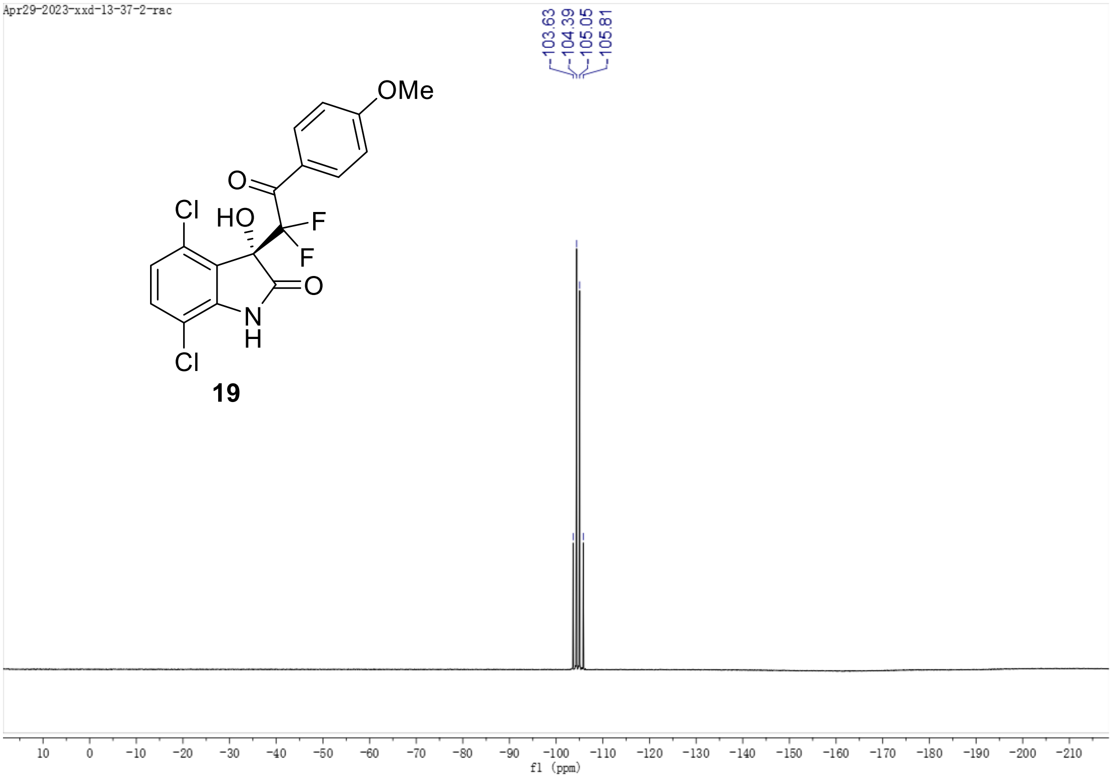
Apr29-2023-xxd-13-37-2-rac
-103.63
-104.39
-105.05
-105.81
OMe
O
Cl
HO
F
F
O
N
H
Cl
19
f1 (ppm)

7. HPLC Analyses Figures of Compounds

Condition：Daicel Chiralpak IC, λ = 254 nm, hexane/2-propanol = 80:20

flow rate = 1.0 mL/min

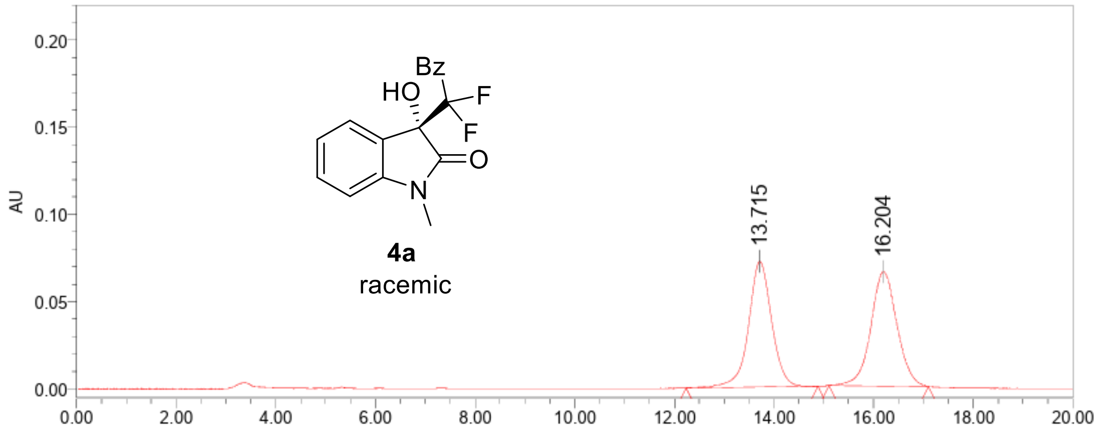

Entry	RT<br>min	Height<br>mV	Area<br>mV.sec	% Area<br>%
1	13.715	72077	2318205	49.13
2	16.204	65578	2400317	50.87

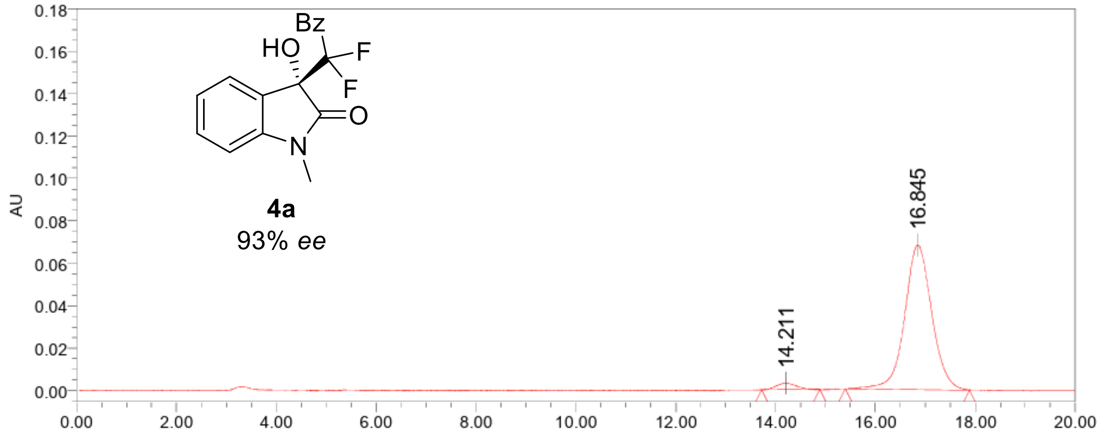

Entry	RT<br>min	Height<br>mV	Area<br>mV.sec	% Area<br>%
1	14.211	3053	85291	3.21
2	16.845	68109	2572423	96.79

Condition：Daicel Chiralpak IC, λ = 254 nm, hexane/2-propanol = 80:20

flow rate = 1.0 mL/min

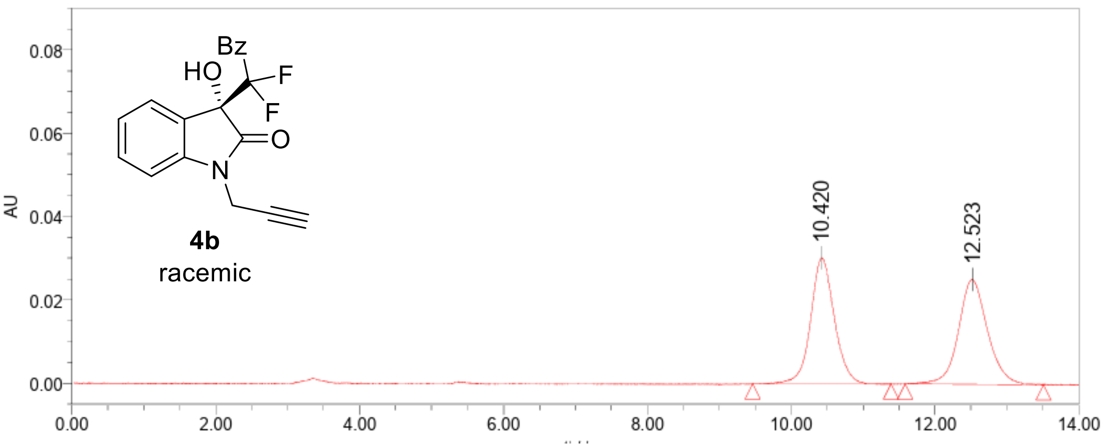

Entry	RT<br>min	Height<br>mV	Area<br>mV.sec	% Area<br>%
1	10.420	30220	693507	49.78
2	12.523	25138	699597	50.22

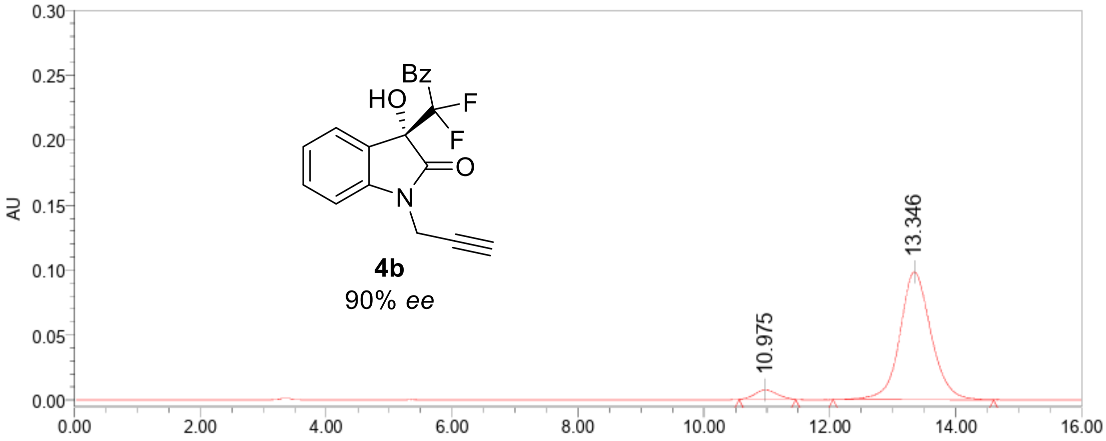

Entry	RT<br>min	Height<br>mV	Area<br>mV.sec	% Area<br>%
1	10.975	7251	175901	4.95
2	13.346	98463	3376365	95.05

Condition: Daicel Chiralpak IC, λ = 254 nm, hexane/2-propanol = 80:20

flow rate = 1.0 mL/min

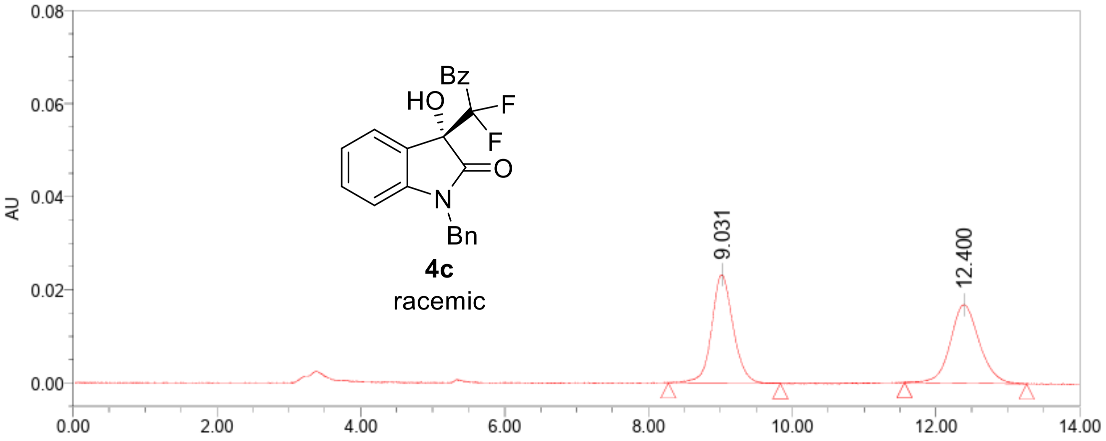

Entry	RT<br>min	Height<br>mV	Area<br>mV.sec	% Area<br>%
1	9.031	23293	484792	50.75
2	12.400	16869	470492	49.25

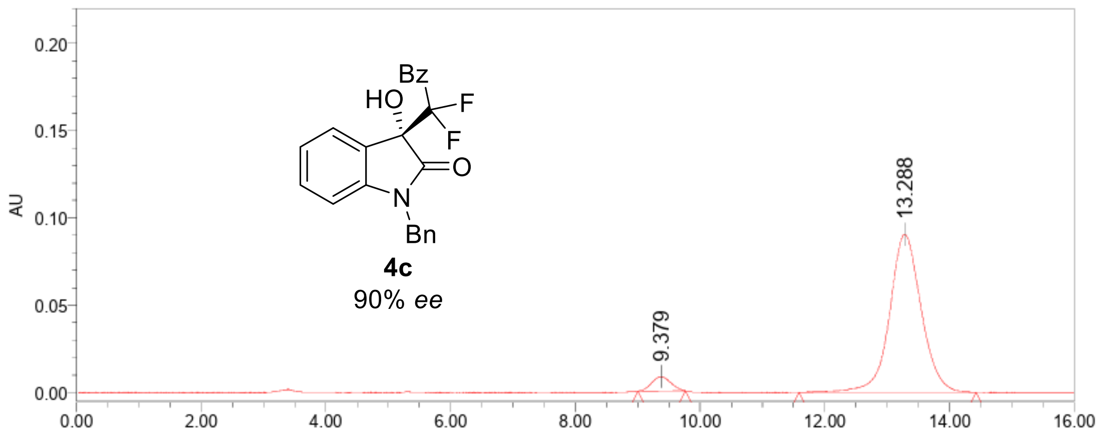

Entry	RT<br>min	Height<br>mV	Area<br>mV.sec	% Area<br>%
1	9.379	8300	175050	5.19
2	13.288	90542	3198874	94.81

Condition： Daicel Chiralpak IC, λ = 254 nm, hexane/2-propanol = 80:20

flow rate = 1.0 mL/min

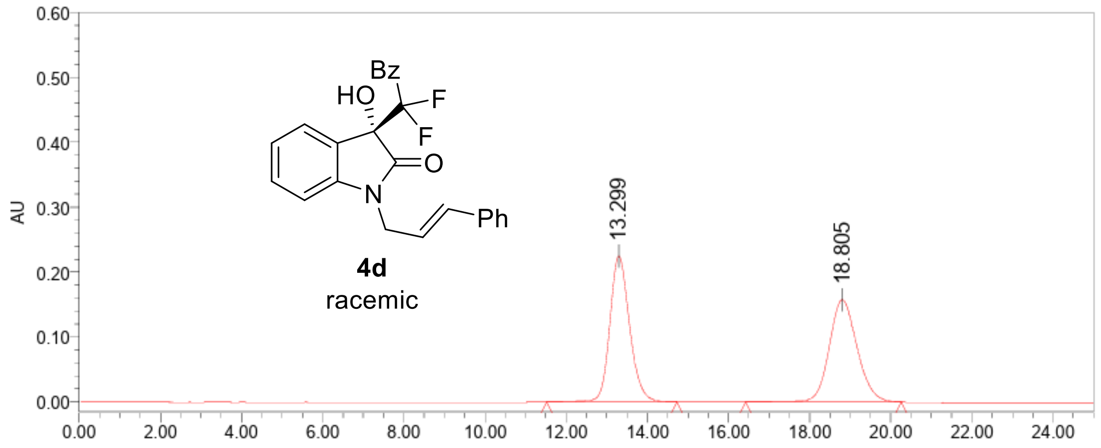

Entry	RT<br>min	Height<br>mV	Area<br>mV.sec	% Area<br>%
1	13.299	225100	7640254	50.31
2	18.805	157746	7545556	49.69

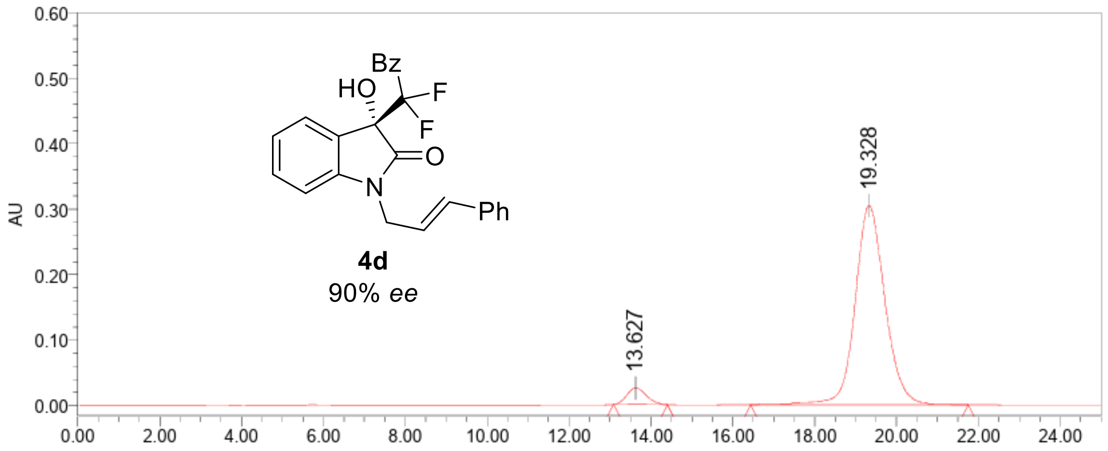

Entry	RT<br>min	Height<br>mV	Area<br>mV.sec	% Area<br>%
1	13.627	25466	838430	5.13
2	19.328	304032	15491703	94.87

Condition：Daicel Chiralpak IA, λ = 254 nm, hexane/2-propanol = 80:20

flow rate = 1.0 mL/min

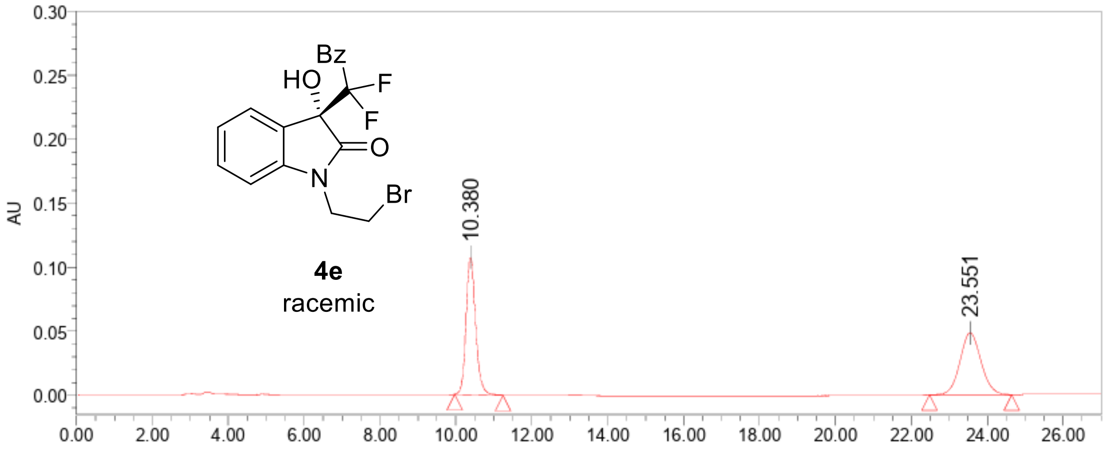

Entry	RT<br>min	Height<br>mV	Area<br>mV.sec	% Area<br>%
1	10.380	107182	1897515	50.28
2	23.551	48546	1876238	49.72

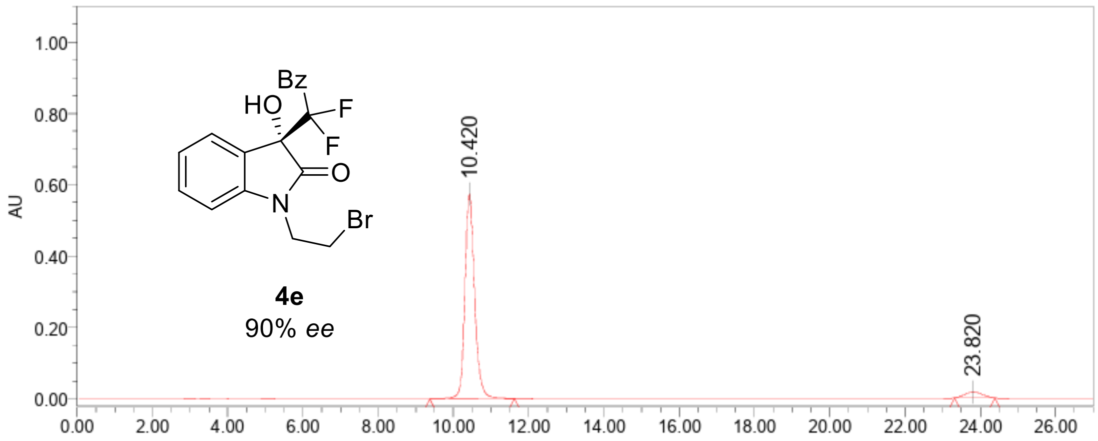

Entry	RT<br>min	Height<br>mV	Area<br>mV.sec	% Area<br>%
1	10.420	573154	10449129	95.03
2	23.820	16631	545908	4.97

Condition：Daicel Chiralpak IC, λ = 254 nm, hexane/2-propanol = 80:20

flow rate = 1.0 mL/min

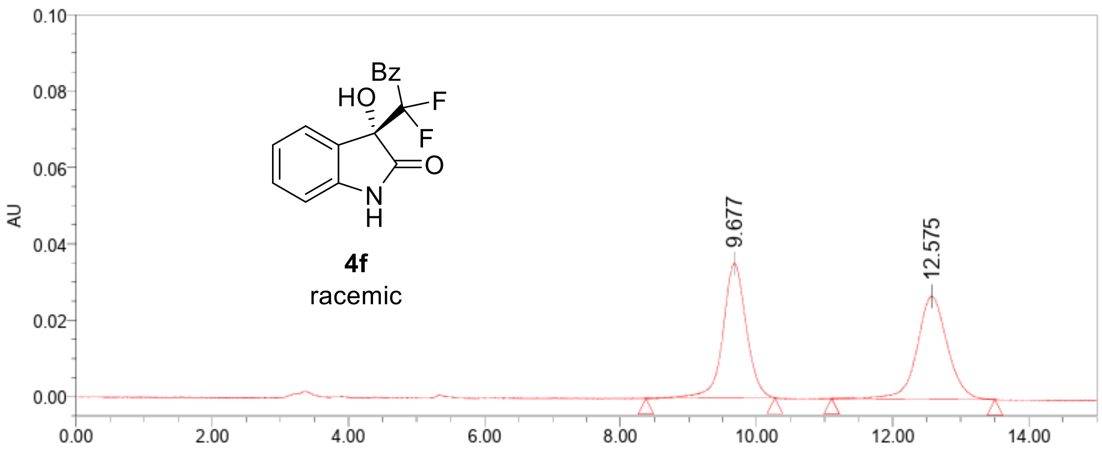

Entry	RT<br>min	Height<br>mV	Area<br>mV.sec	% Area<br>%
1	9.677	35236	839270	50.01
2	12.575	27024	839099	49.99

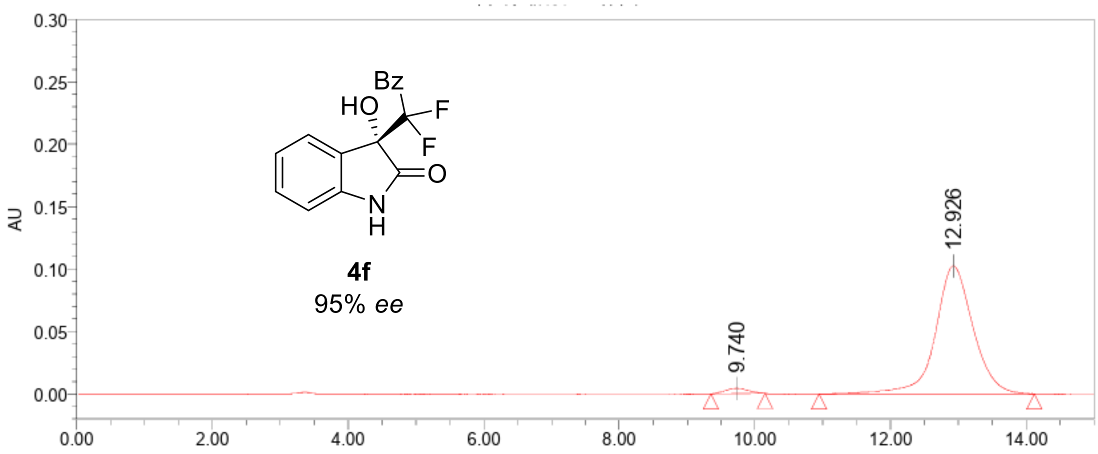

Entry	RT<br>min	Height<br>mV	Area<br>mV.sec	% Area<br>%
1	9.740	4133	95208	2.35
2	12.926	102533	3953548	97.65

Condition：Daicel Chiralpak IA, λ = 254 nm, hexane/2-propanol = 80:20

flow rate = 1.0 mL/min

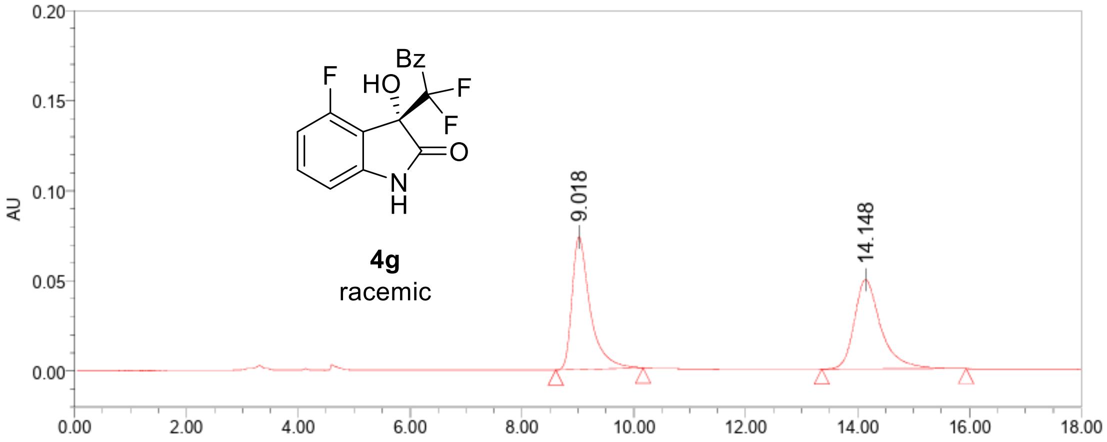

Entry	RT<br>min	Height<br>mV	Area<br>mV.sec	% Area<br>%
1	9.018	73533	1654084	50.00
2	14.148	49657	1653820	50.00

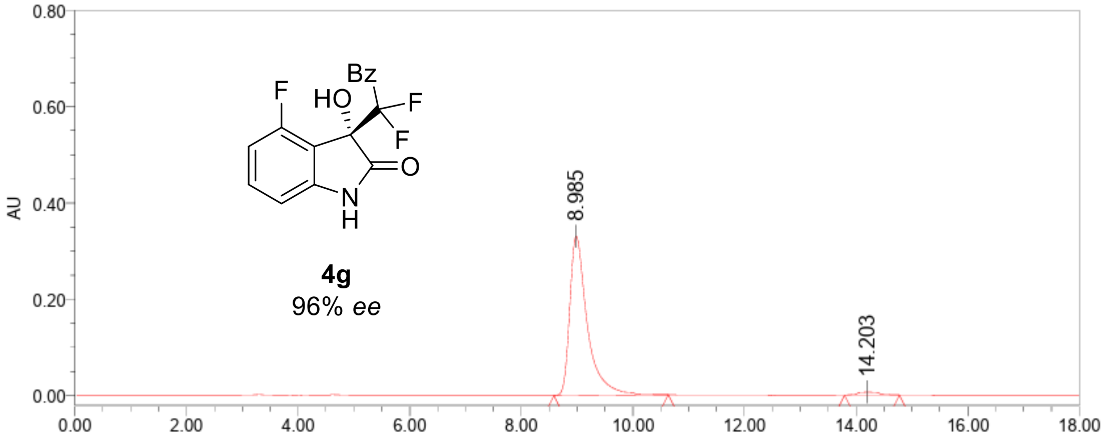

Entry	RT<br>min	Height<br>mV	Area<br>mV.sec	% Area<br>%
1	8.985	330688	7221038	97.75
2	14.203	5945	166027	2.25

Condition：Daicel Chiralpak IA, λ = 254 nm, hexane/2-propanol = 80:20

flow rate = 1.0 mL/min

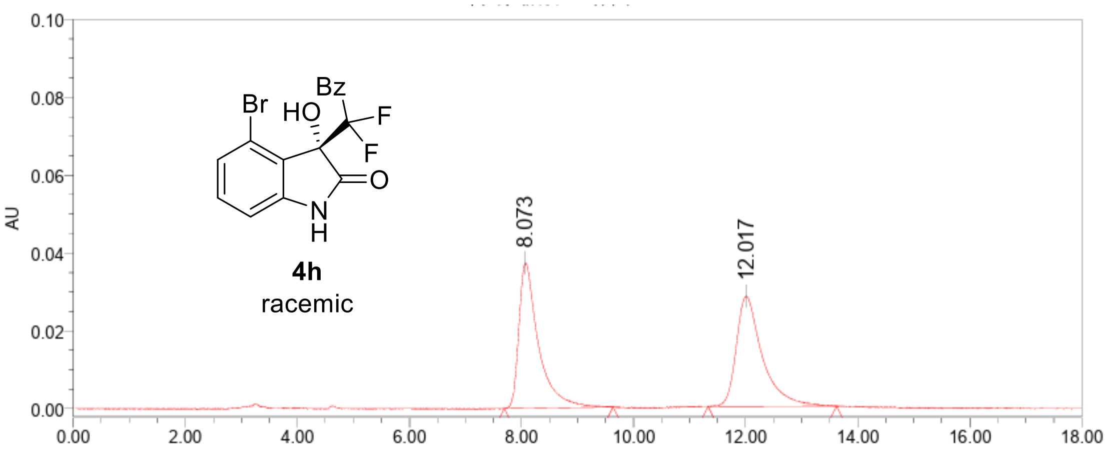

Entry	RT<br>min	Height<br>mV	Area<br>mV.sec	% Area<br>%
1	8.073	37315	902102	49.73
2	12.017	28289	911749	50.27

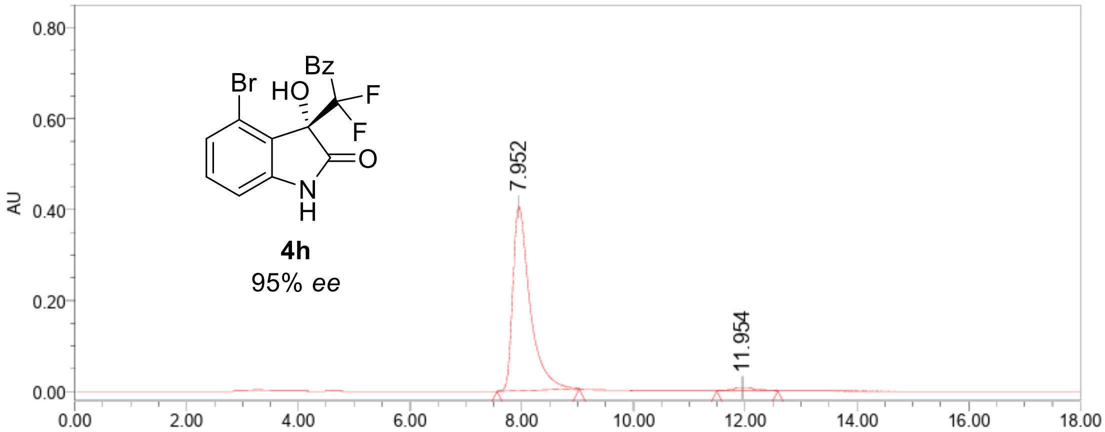

Entry	RT<br>min	Height<br>mV	Area<br>mV.sec	% Area<br>%
1	7.952	404545	8662956	97.72
2	11.954	7065	202205	2.28

Condition: Daicel Chiralpak IA, λ = 254 nm, hexane/2-propanol = 80:20

flow rate = 1.0 mL/min

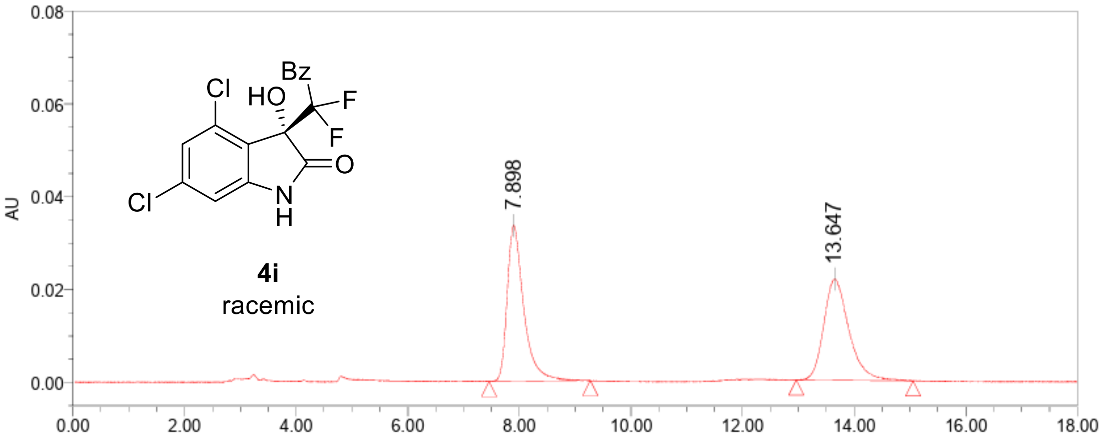

Entry	RT<br>min	Height<br>mV	Area<br>mV.sec	% Area<br>%
1	7.898	33555	690508	50.25
2	13.647	21806	683625	49.75

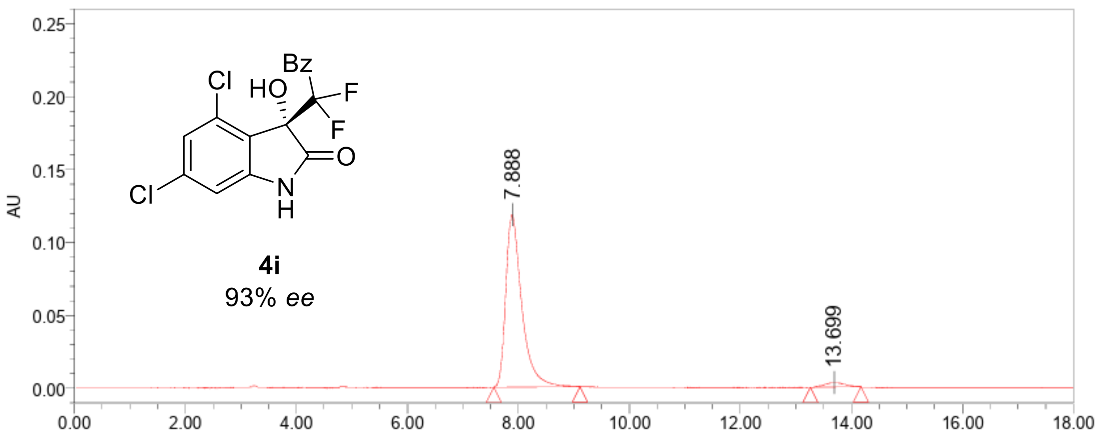

Entry	RT<br>min	Height<br>mV	Area<br>mV.sec	% Area<br>%
1	7.888	118133	2358959	96.69
2	13.699	3062	80780	3.31

Condition：Daicel Chiralpak IA, λ = 254 nm, hexane/2-propanol = 80:20

flow rate = 1.0 mL/min

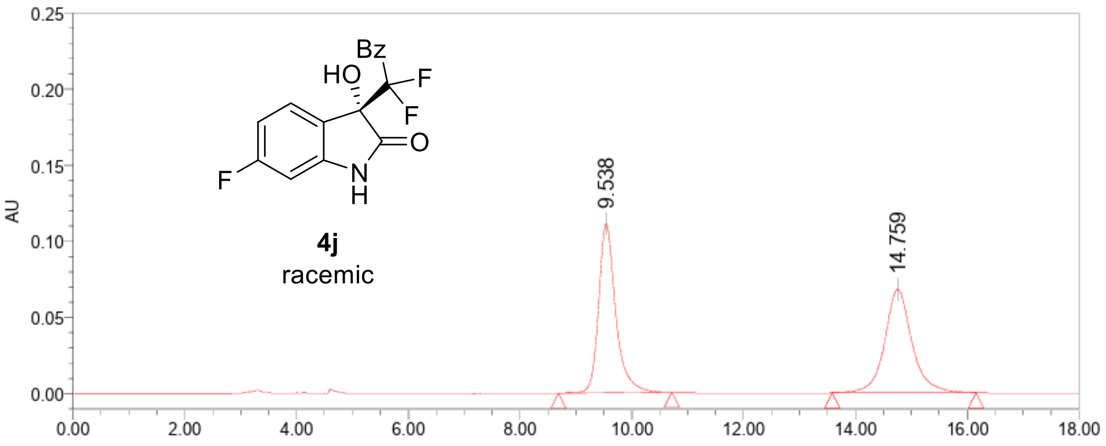

Entry	RT<br>min	Height<br>mV	Area<br>mV.sec	% Area<br>%
1	9.538	111074	2364154	50.48
2	14.759	68005	2318831	49.52

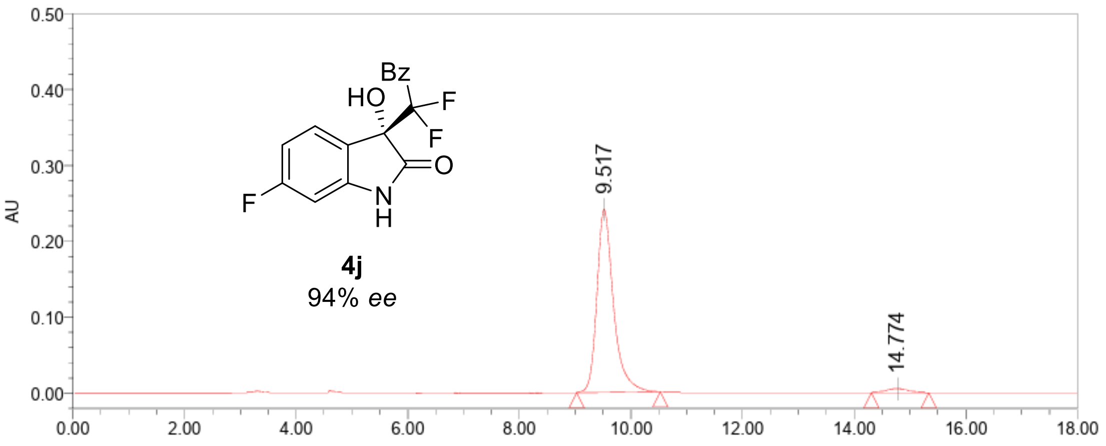

Entry	RT<br>min	Height<br>mV	Area<br>mV.sec	% Area<br>%
1	9.517	240650	4981671	97.19
2	14.774	5100	144028	2.81

Condition: Daicel Chiralpak IA, λ = 254 nm, hexane/2-propanol = 80:20

flow rate = 1.0 mL/min

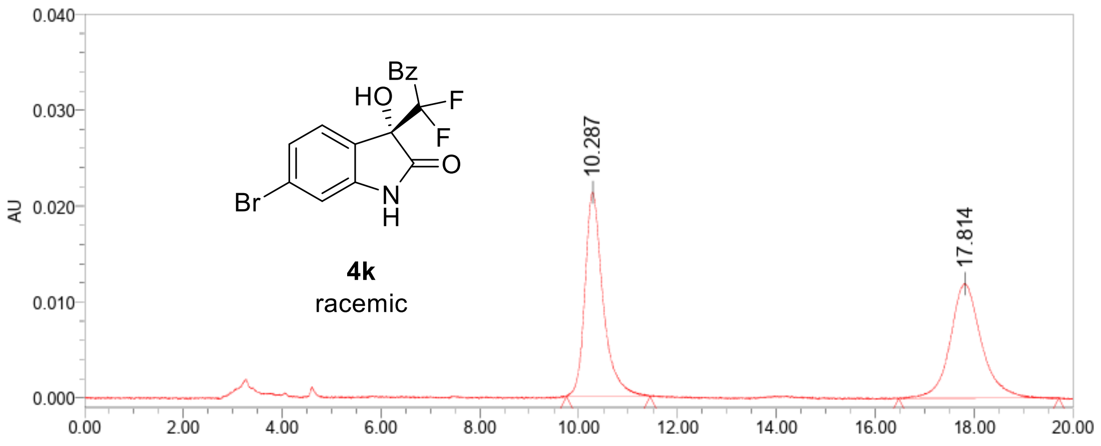

Entry	RT<br>min	Height<br>mV	Area<br>mV.sec	% Area<br>%
1	10.287	21254	538087	50.80
2	17.814	11959	521212	49.20

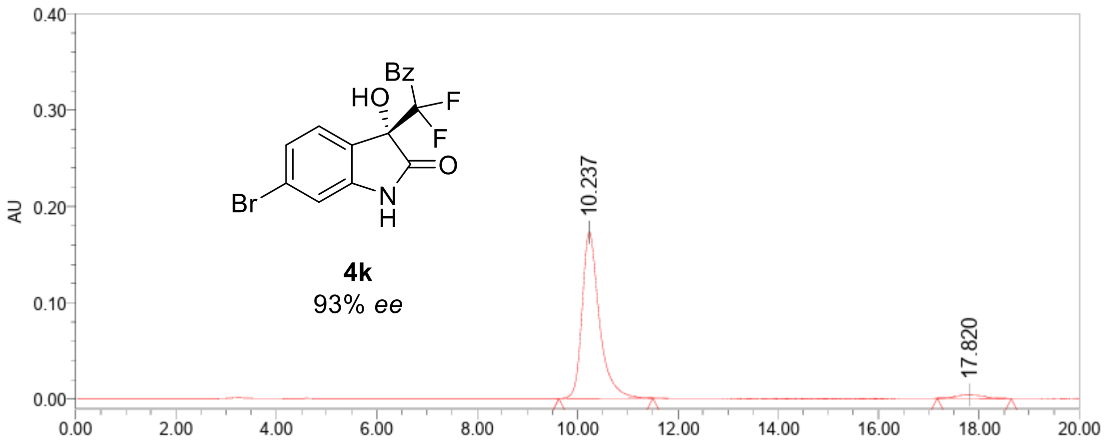

Entry	RT<br>min	Height<br>mV	Area<br>mV.sec	% Area<br>%
1	10.237	172620	4117294	96.58
2	17.820	3961	145786	3.42

Condition: Daicel Chiralpak IA, λ = 254 nm, hexane/2-propanol = 80:20

flow rate = 1.0 mL/min

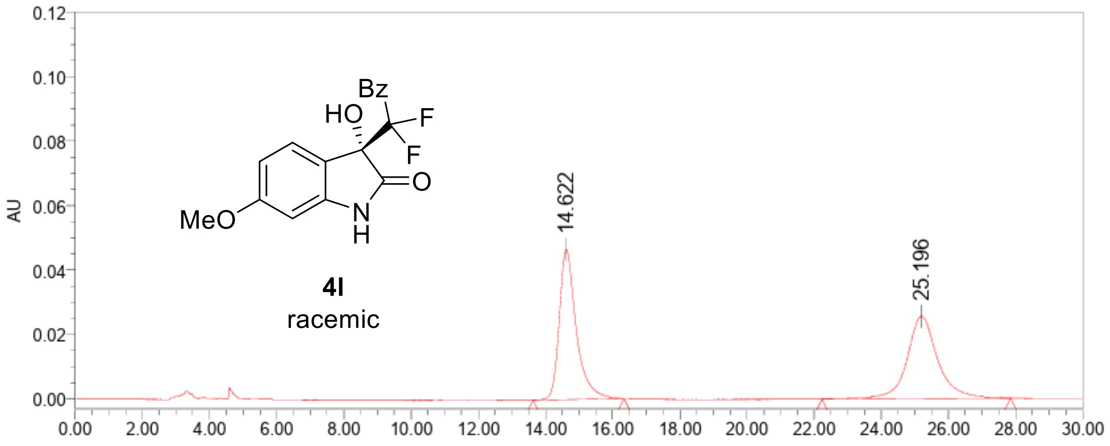

Entry	RT<br>min	Height<br>mV	Area<br>mV.sec	% Area<br>%
1	14.622	46550	1629871	50.10
2	25.196	25542	1623150	49.90

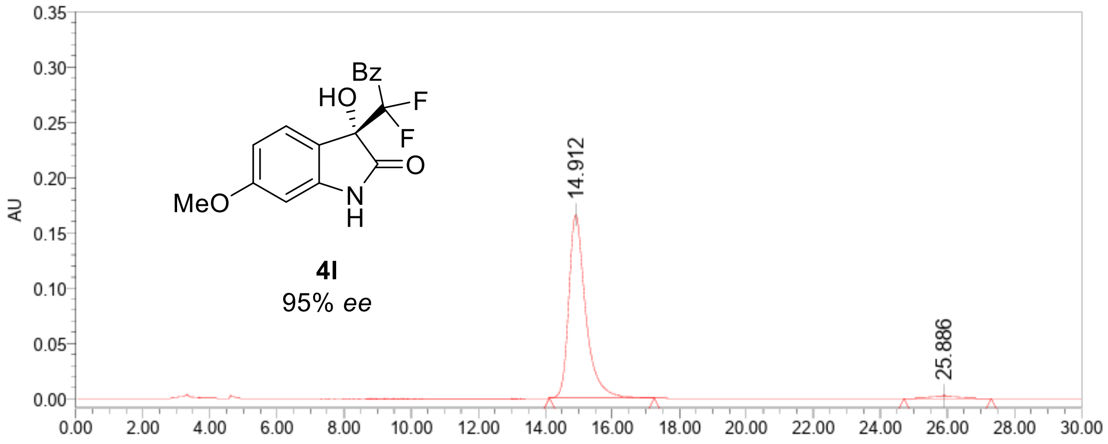

Entry	RT<br>min	Height<br>mV	Area<br>mV.sec	% Area<br>%
1	14.912	165799	5805600	97.55
2	25.886	2556	145713	2.45

Condition: Daicel Chiralpak IA, λ = 254 nm, hexane/2-propanol = 80:20

flow rate = 1.0 mL/min

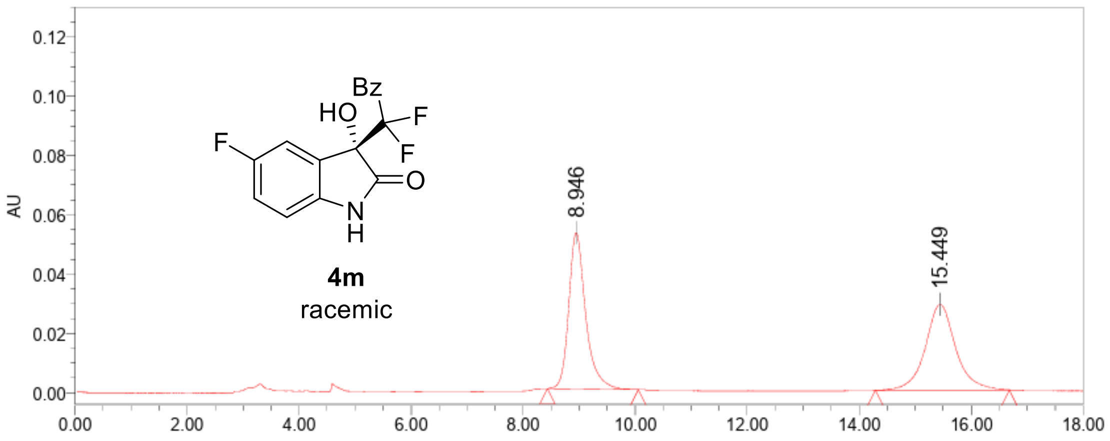

Entry	RT min	Height mV	Area mV.sec	% Area %
1	8.946	52671	1098805	50.20
2	15.449	28971	1090254	49.80

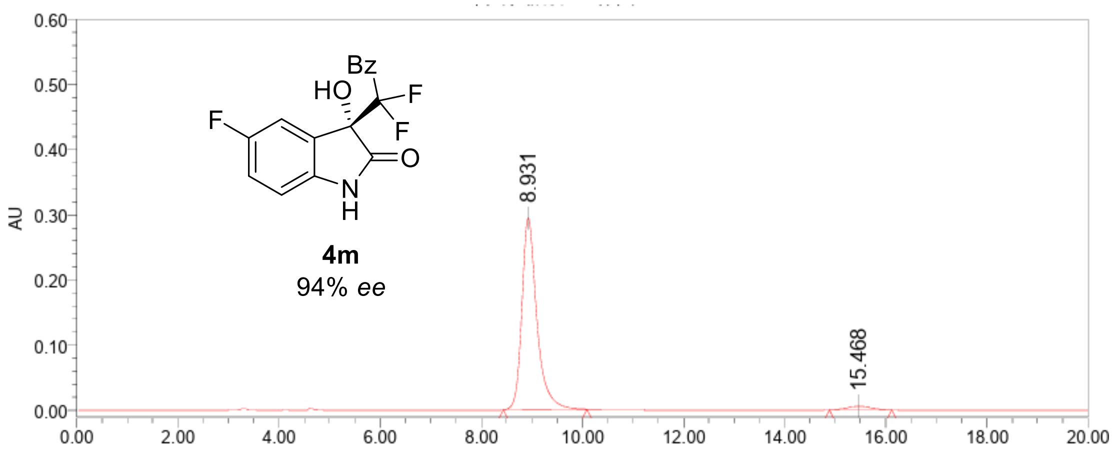

Entry	RT min	Height mV	Area mV.sec	% Area %
1	8.931	293955	6042813	97.05
2	15.468	5693	183631	2.95

Condition: Daicel Chiralpak IA, λ = 254 nm, hexane/2-propanol = 80:20

flow rate = 1.0 mL/min

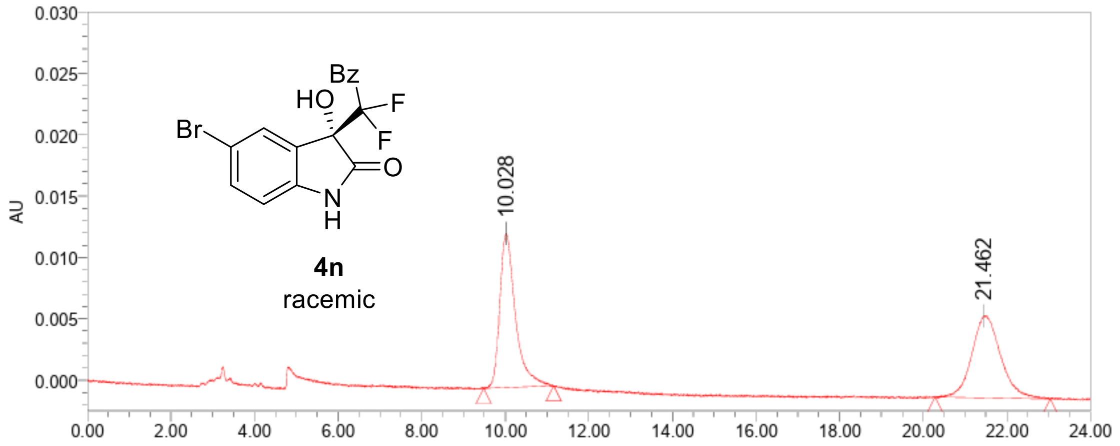

Entry	RT<br>min	Height<br>mV	Area<br>mV.sec	% Area<br>%
1	10.028	12582	325323	50.39
2	21.462	6706	320228	49.61

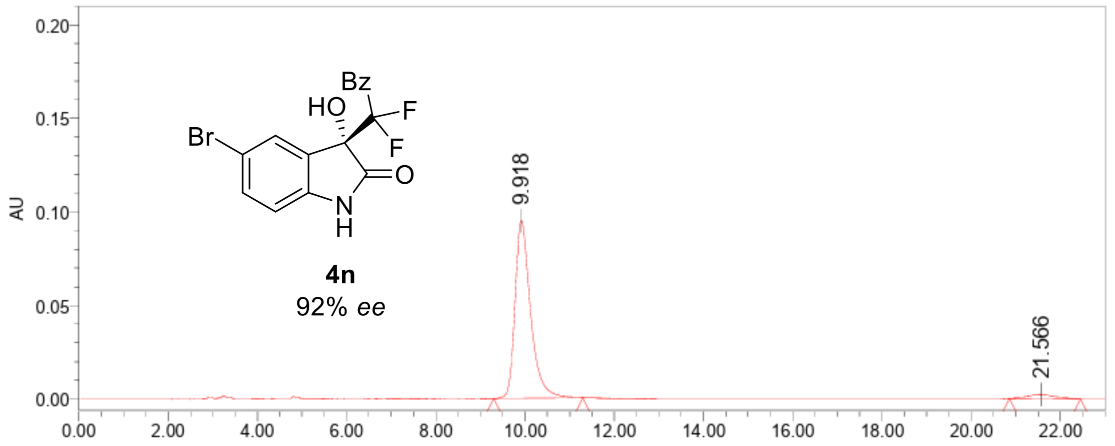

Entry	RT<br>min	Height<br>mV	Area<br>mV.sec	% Area<br>%
1	9.918	94936	2294053	96.20
2	21.566	2183	90716	3.80

Condition：Daicel Chiralpak IA, λ = 254 nm, hexane/2-propanol = 80:20

flow rate = 1.0 mL/min

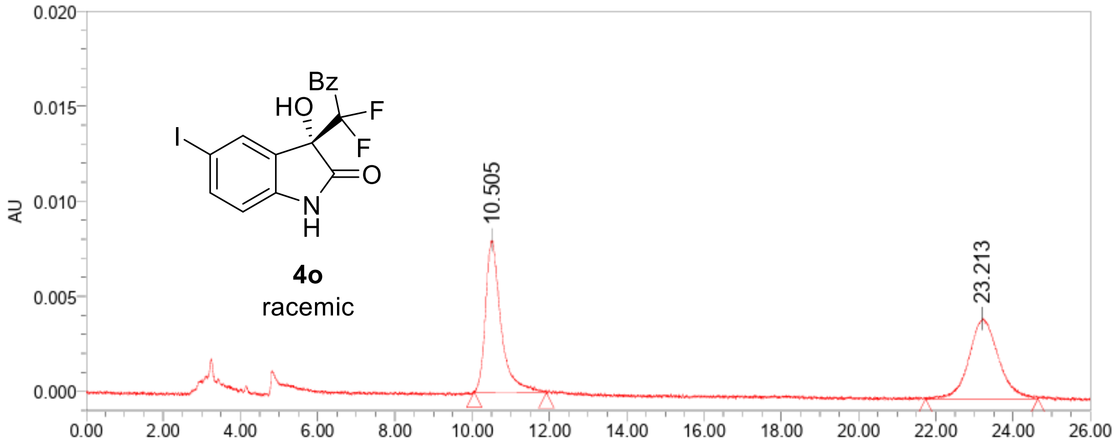

Entry	RT<br>min	Height<br>mV	Area<br>mV.sec	% Area<br>%
1	10.505	8022	225038	50.58
2	23.213	4210	219838	49.42

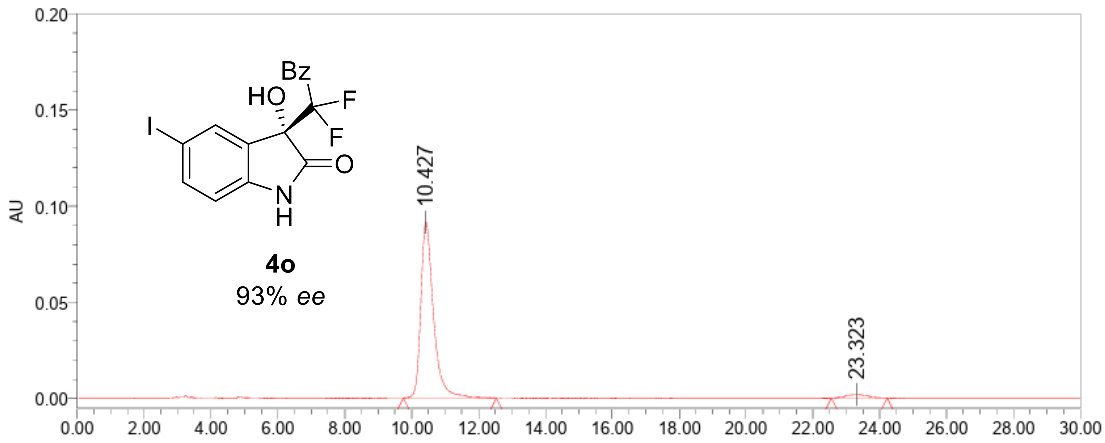

Entry	RT<br>min	Height<br>mV	Area<br>mV.sec	% Area<br>%
1	10.427	91439	2396324	96.43
2	23.323	1961	88653	3.57

Condition：Daicel Chiralpak IA, λ = 254 nm, hexane/2-propanol = 80:20

flow rate = 1.0 mL/min

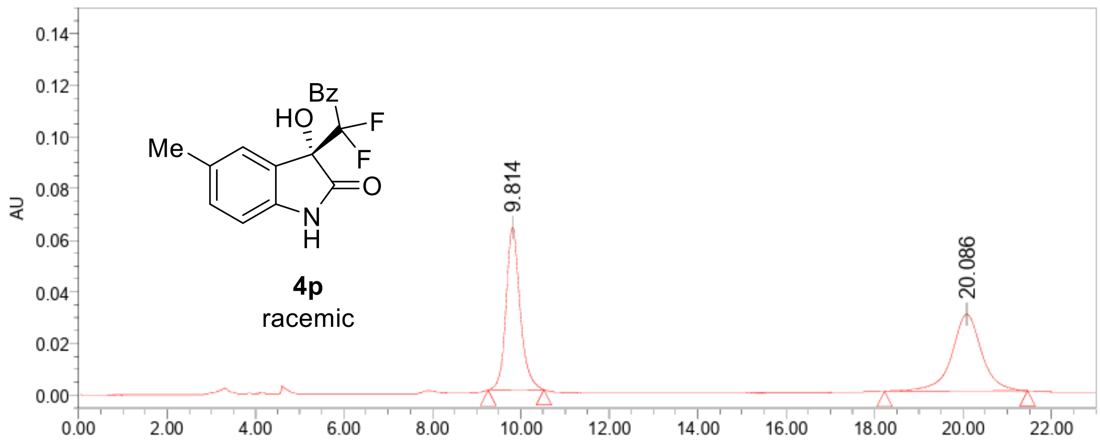

Entry	RT<br>min	Height<br>mV	Area<br>mV.sec	% Area<br>%
1	9.814	62879	1391115	49.86
2	20.086	29690	1399167	50.14

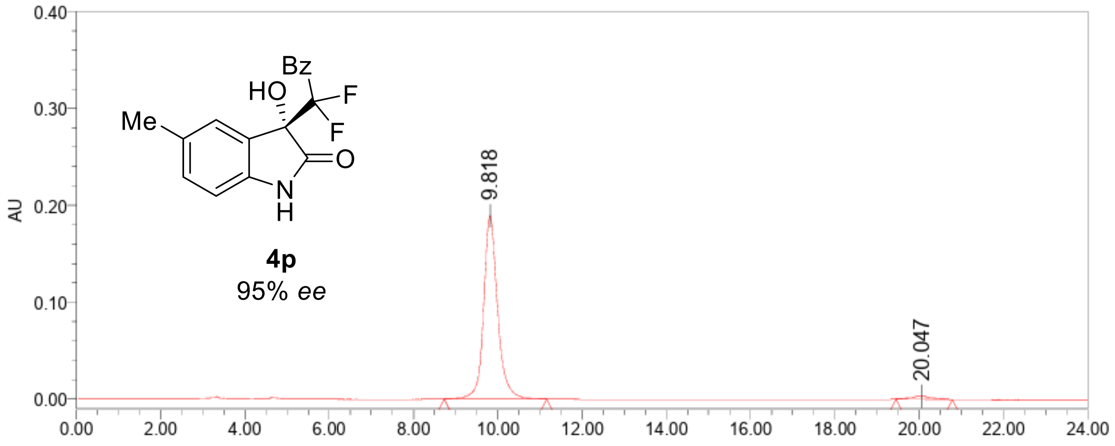

Entry	RT<br>min	Height<br>mV	Area<br>mV.sec	% Area<br>%
1	9.818	189408	4370444	97.55
2	20.047	2972	109897	2.45

Condition: Daicel Chiralpak IA, λ = 254 nm, hexane/2-propanol = 80:20

flow rate = 1.0 mL/min

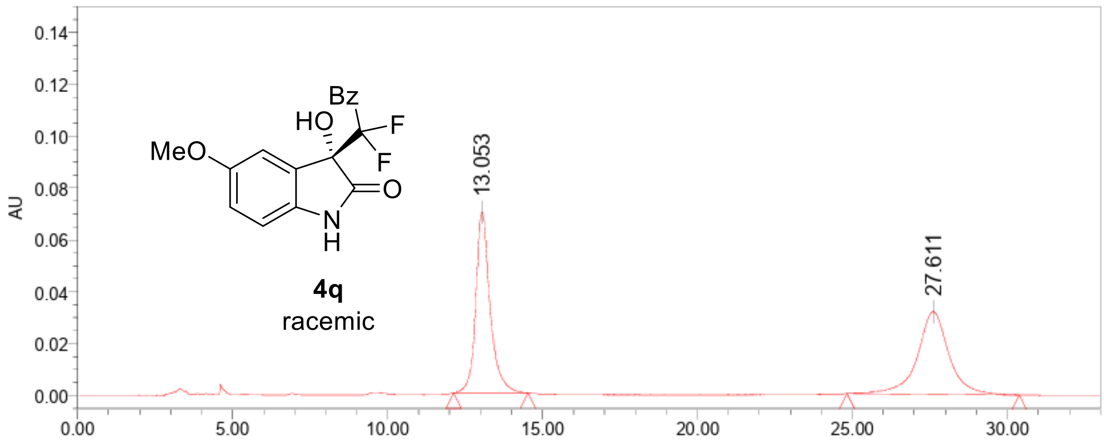

Entry	RT<br>min	Height<br>mV	Area<br>mV.sec	% Area<br>%
1	13.053	69718	2269613	49.98
2	27.611	31935	2271340	50.02

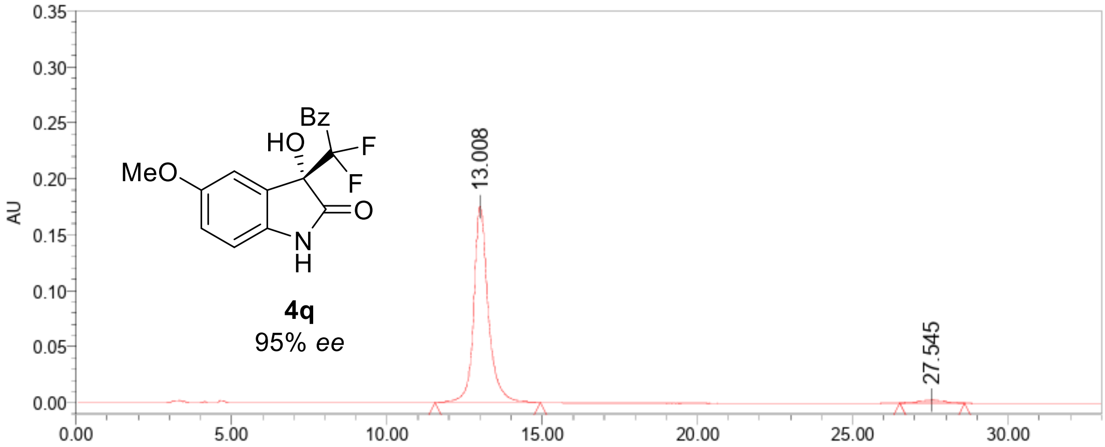

Entry	RT<br>min	Height<br>mV	Area<br>mV.sec	% Area<br>%
1	13.008	175019	5725730	97.50
2	27.545	2598	146671	2.50

Condition: Daicel Chiralpak IA, λ = 254 nm, hexane/2-propanol = 80:20

flow rate = 1.0 mL/min

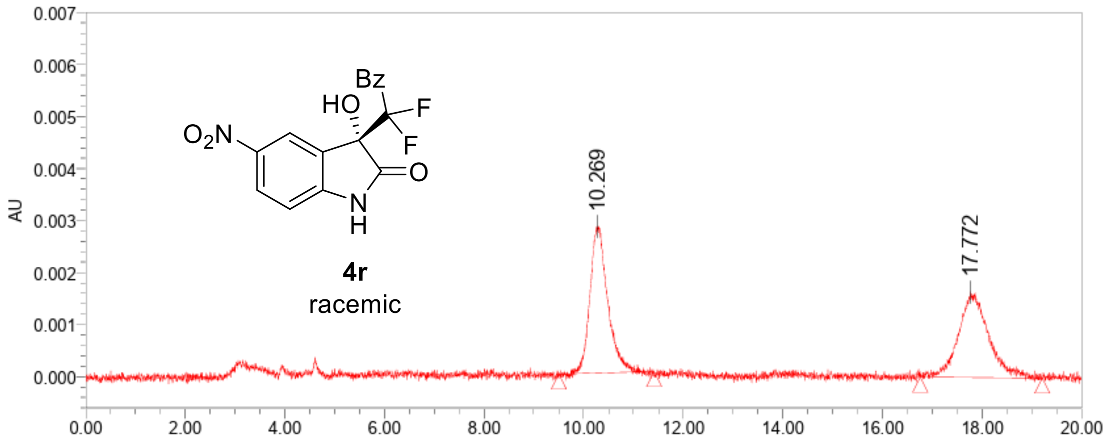

Entry	RT<br>min	Height<br>mV	Area<br>mV.sec	% Area<br>%
1	10.269	2819	70731	50.79
2	17.772	1654	68539	49.21

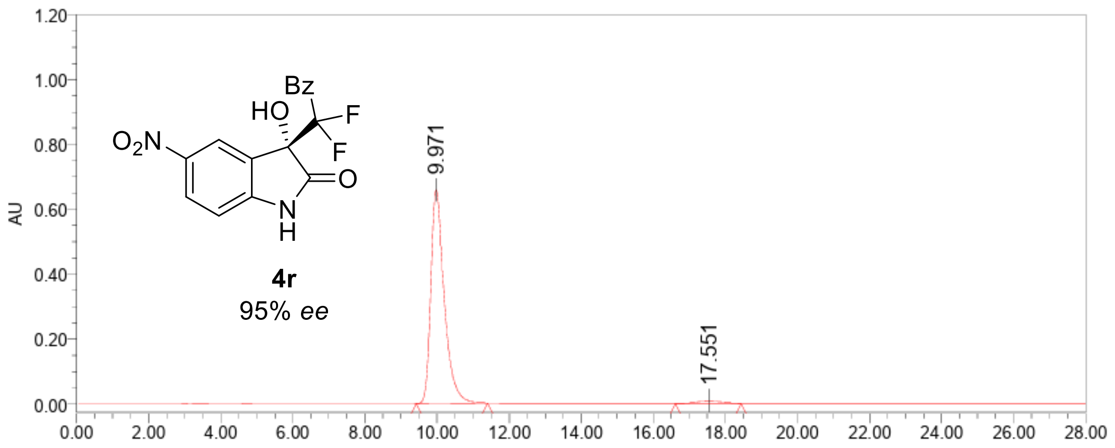

Entry	RT<br>min	Height<br>mV	Area<br>mV.sec	% Area<br>%
1	9.971	658632	17092951	97.52
2	17.551	9087	435182	2.48

Condition：Daicel Chiralpak IA, λ = 254 nm, hexane/2-propanol = 80:20

flow rate = 1.0 mL/min

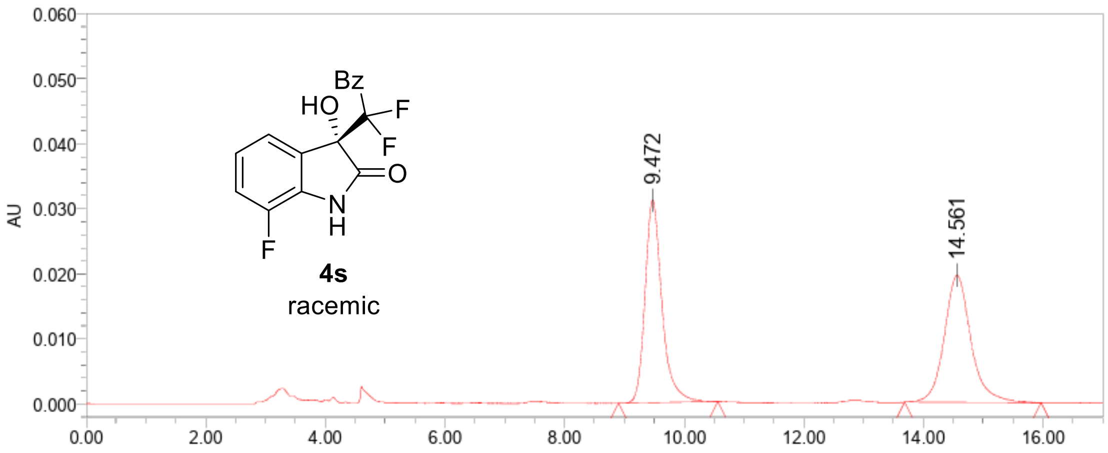

Entry	RT<br>min	Height<br>mV	Area<br>mV.sec	% Area<br>%
1	9.472	31111	611563	50.31
2	14.561	19530	603941	49.69

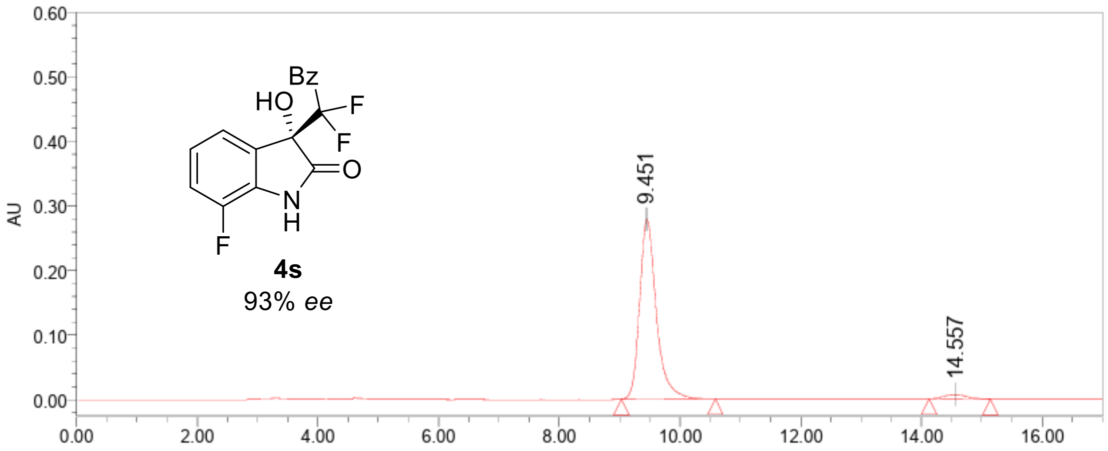

Entry	RT<br>min	Height<br>mV	Area<br>mV.sec	% Area<br>%
1	9.451	278478	5357981	96.58
2	14.557	7134	189463	3.42

Condition：Daicel Chiralpak IA, λ = 254 nm, hexane/2-propanol = 80:20

flow rate = 1.0 mL/min

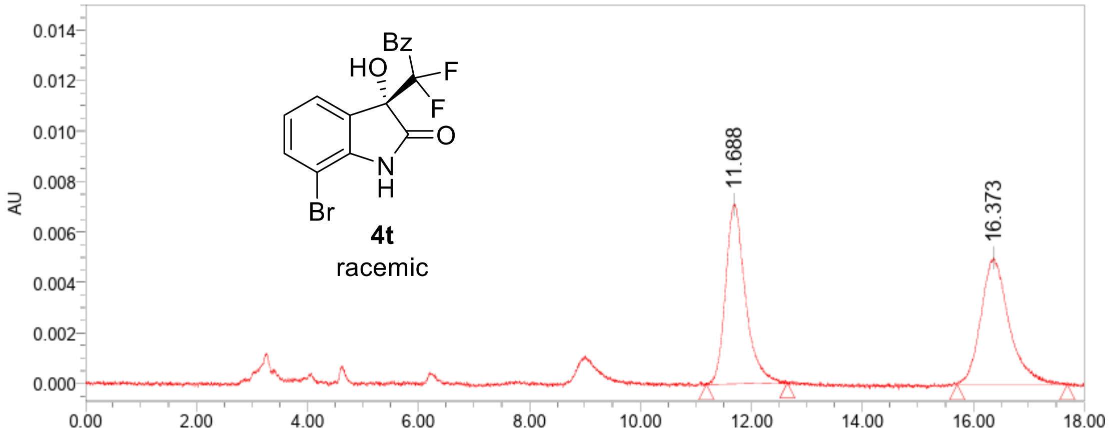

Entry	RT<br>min	Height<br>mV	Area<br>mV.sec	% Area<br>%
1	11.688	7118	177067	49.92
2	16.373	5026	177669	50.08

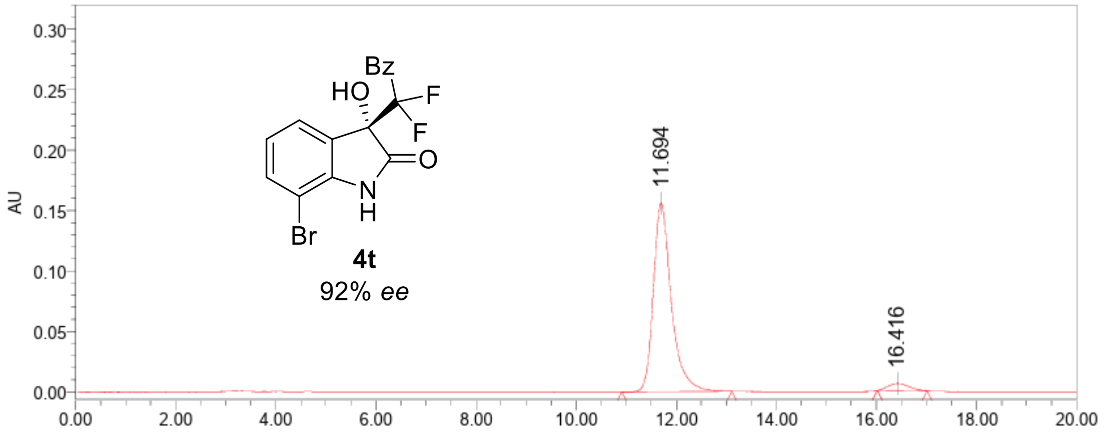

Entry	RT<br>min	Height<br>mV	Area<br>mV.sec	% Area<br>%
1	11.694	155883	3894005	95.81
2	16.416	5912	170449	4.19

Condition: Daicel Chiralpak IA, λ = 254 nm, hexane/2-propanol = 80:20

flow rate = 1.0 mL/min

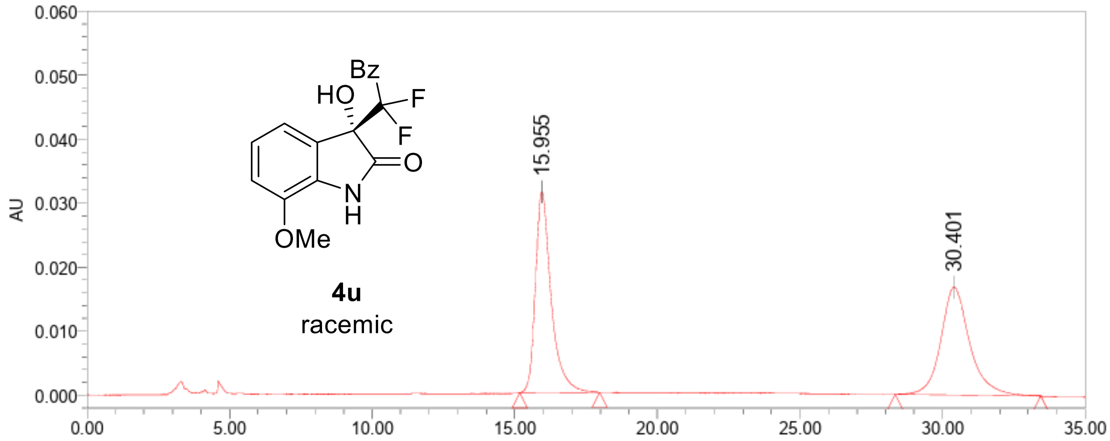

Entry	RT<br>min	Height<br>mV	Area<br>mV.sec	% Area<br>%
1	15.955	31386	1195844	50.10
2	30.401	16868	1191225	49.90

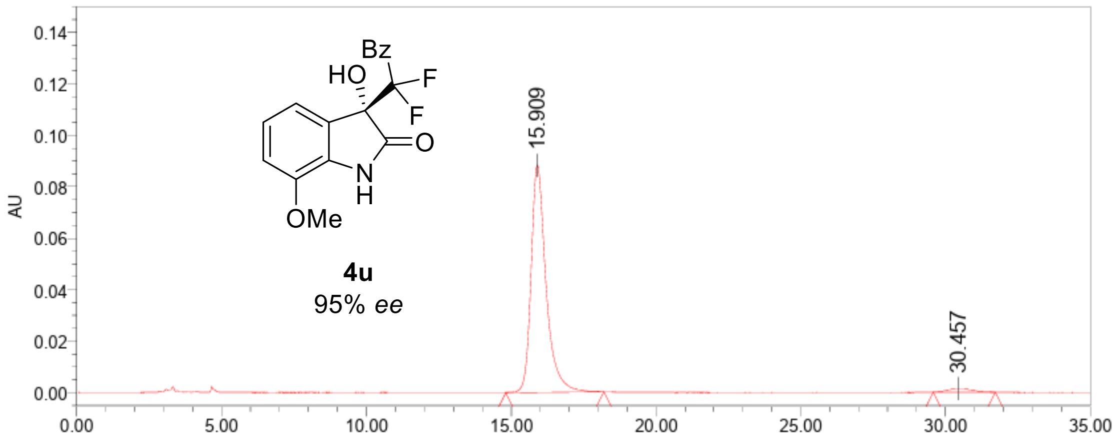

Entry	RT<br>min	Height<br>mV	Area<br>mV.sec	% Area<br>%
1	15.909	88252	3156176	97.54
2	30.457	1371	79575	2.46

Condition：Daicel Chiralpak IA, λ = 254 nm, hexane/2-propanol = 80:20

flow rate = 1.0 mL/min

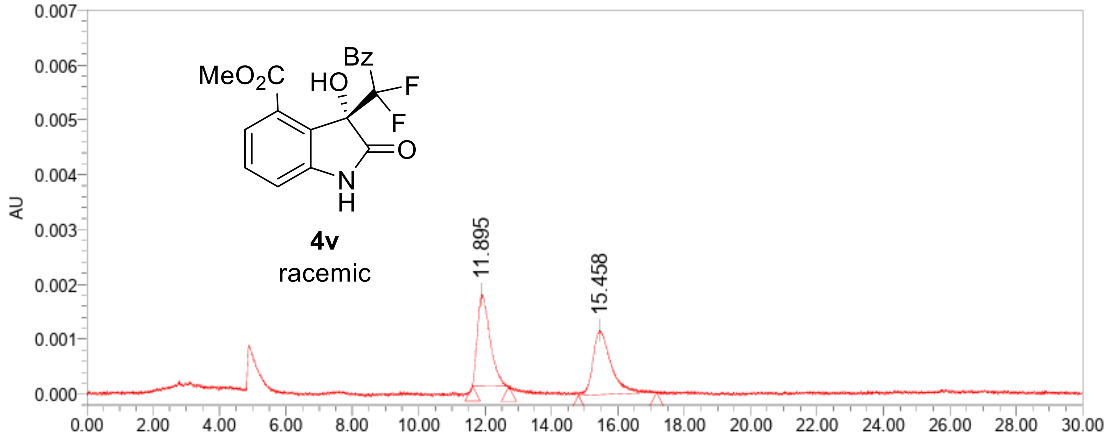

Entry	RT<br>min	Height<br>mV	Area<br>mV.sec	% Area<br>%
1	11.895	1674	44026	50.10
2	15.458	1174	44298	49.90

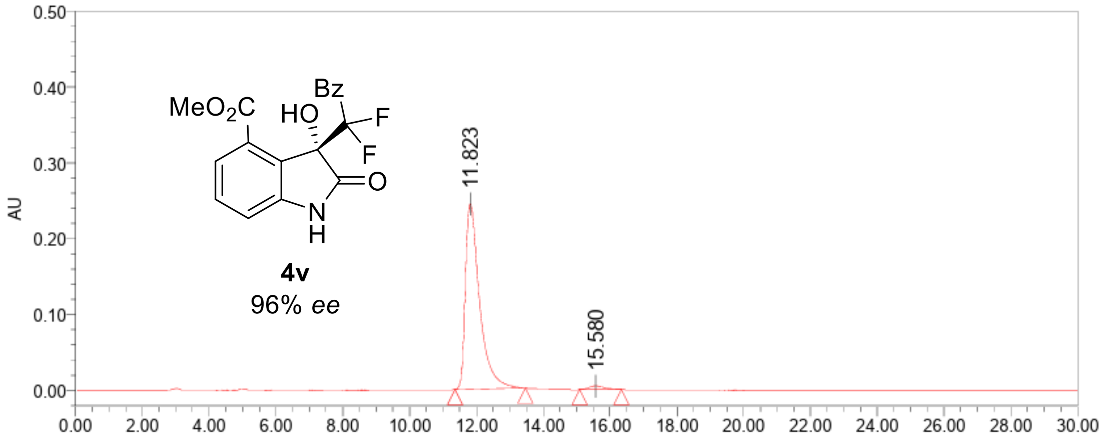

Entry	RT<br>min	Height<br>mV	Area<br>mV.sec	% Area<br>%
1	11.823	244181	7248992	97.89
2	15.580	4668	156147	2.11

Conditions: Chiralpak IA, λ = 254 nm, hexane : 2-propanol = 80:20, flow rate = 1.0 mL/min.

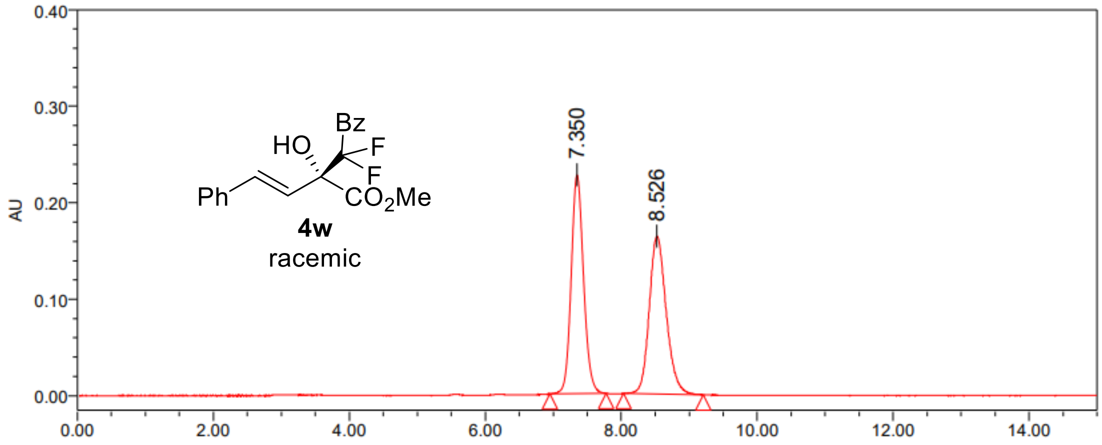

Entry	RT<br>min	Height<br>mV	Area<br>mV.sec	% Area<br>%
1	7.350	226733	2855514	50.14
2	8.526	163557	2839948	49.86

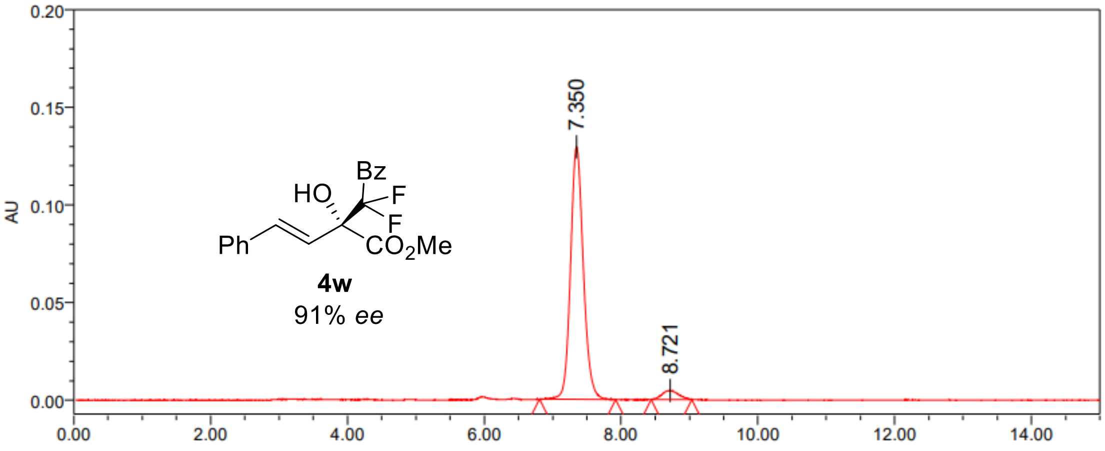

Entry	RT<br>min	Height<br>mV	Area<br>mV.sec	% Area<br>%
1	7.350	129402	1676888	95.67
2	8.721	4560	75818	4.33

Conditions: Chiralpak IA, λ = 254 nm, hexane : 2-propanol = 80:20, flow rate = 1.0 mL/min.

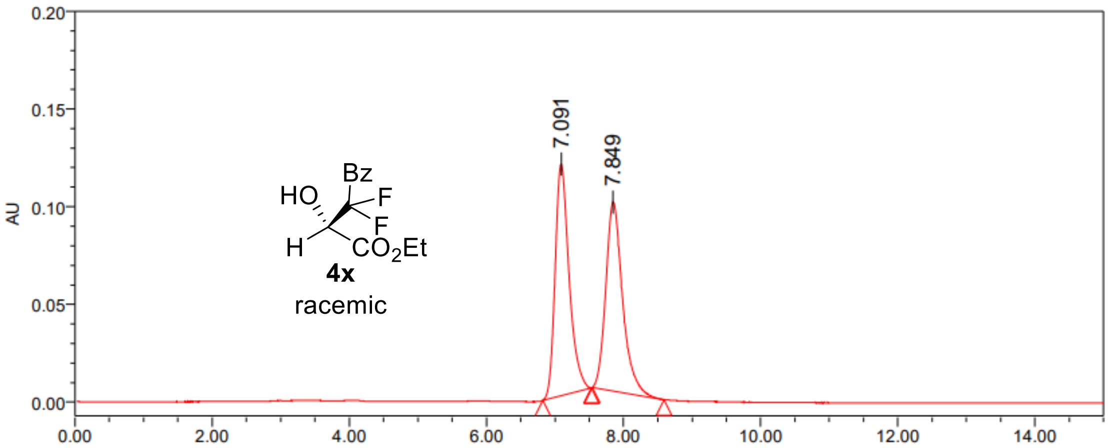

Entry	RT<br>min	Height<br>mV	Area<br>mV.sec	% Area<br>%
1	7.091	118459	1669605	50.46
2	7.849	96489	1639245	49.54

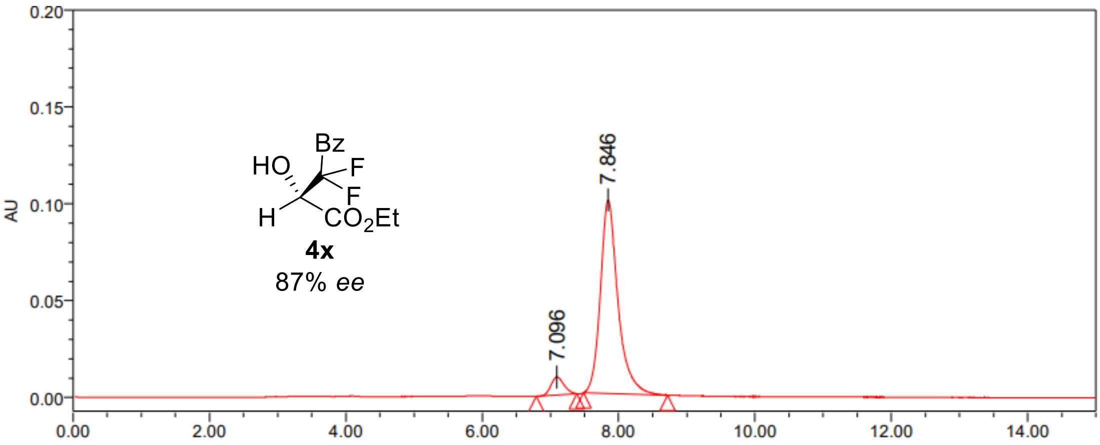

Entry	RT<br>min	Height<br>mV	Area<br>mV.sec	% Area<br>%
1	7.096	9247	122024	6.39
2	7.846	99825	1788686	93.61

Condition：Daicel Chiralpak IA, λ = 254 nm, hexane/2-propanol = 80:20

flow rate = 1.0 mL/min

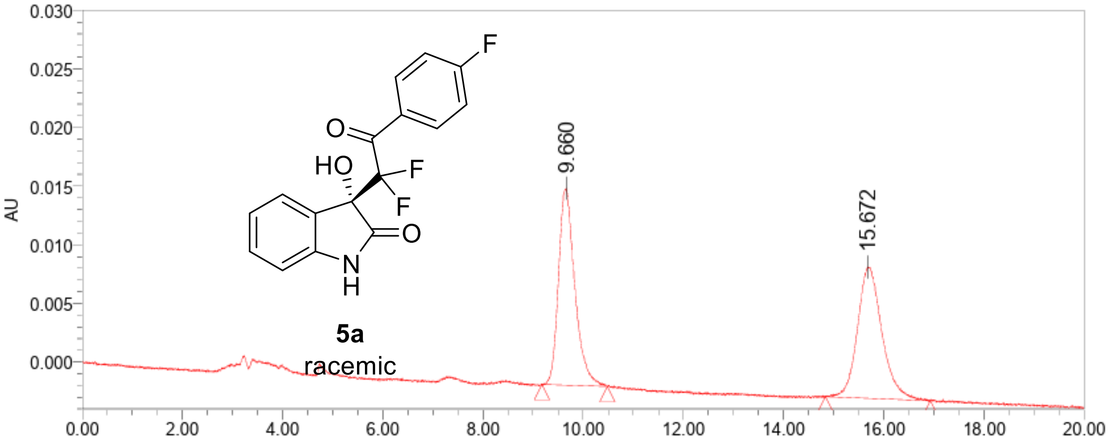

Entry	RT<br>min	Height<br>mV	Area<br>mV.sec	% Area<br>%
1	9.660	16806	383645	50.04
2	15.672	11210	382959	49.96

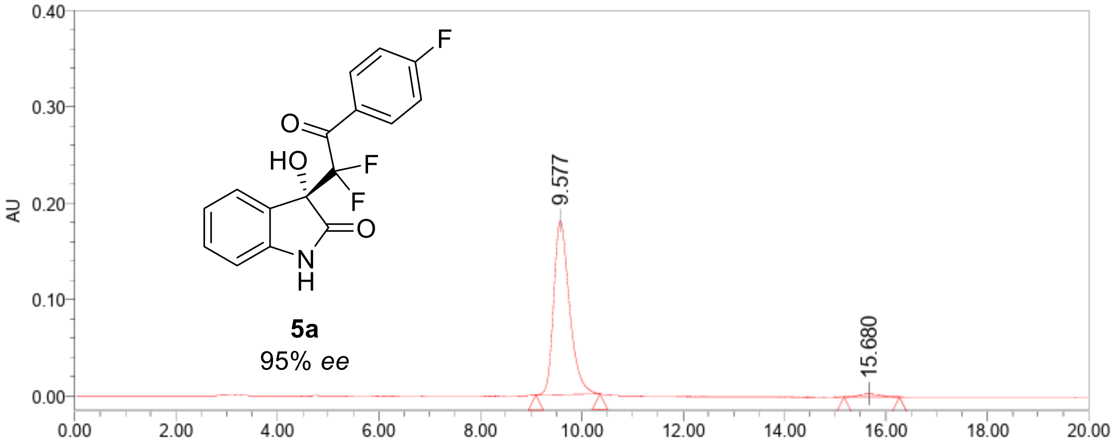

Entry	RT<br>min	Height<br>mV	Area<br>mV.sec	% Area<br>%
1	9.577	180380	3880201	97.50
2	15.680	3424	99608	2.50

Condition: Daicel Chiralpak IA, λ = 254 nm, hexane/2-propanol = 80:20

flow rate = 1.0 mL/min

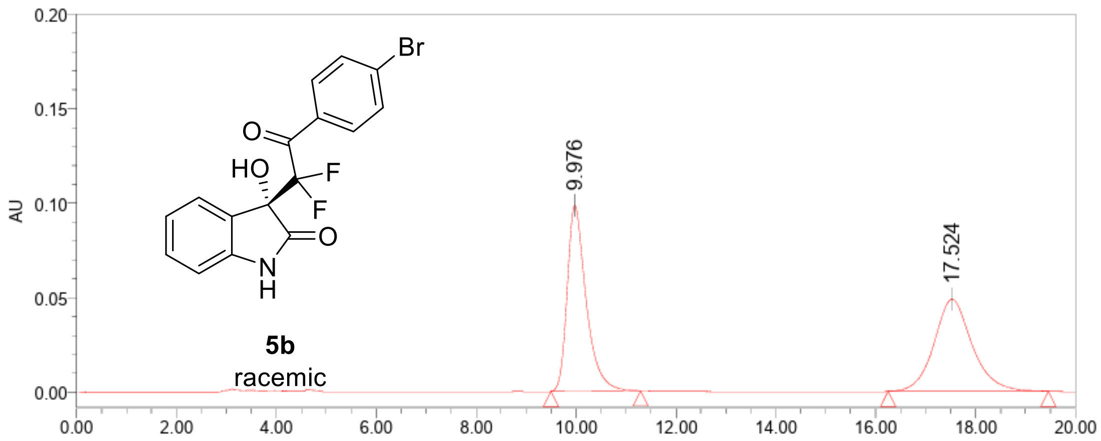

Entry	RT<br>min	Height<br>mV	Area<br>mV.sec	% Area<br>%
1	9.976	98310	2597296	50.14
2	17.524	48761	2583066	49.86

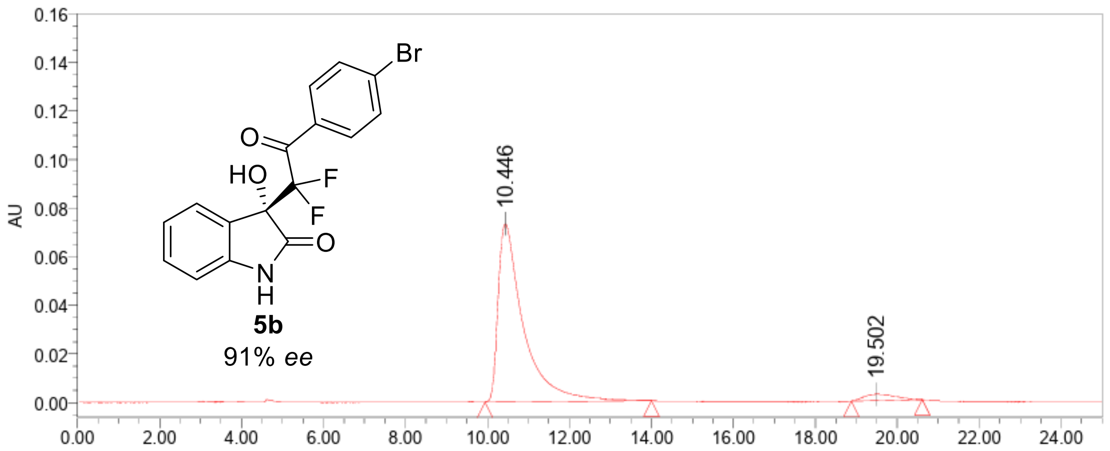

Entry	RT<br>min	Height<br>mV	Area<br>mV.sec	% Area<br>%
1	10.446	73287	3141299	95.66
2	19.502	2624	142392	4.34

Condition：Daicel Chiralpak IA, λ = 254 nm, hexane/2-propanol = 80:20

flow rate = 1.0 mL/min

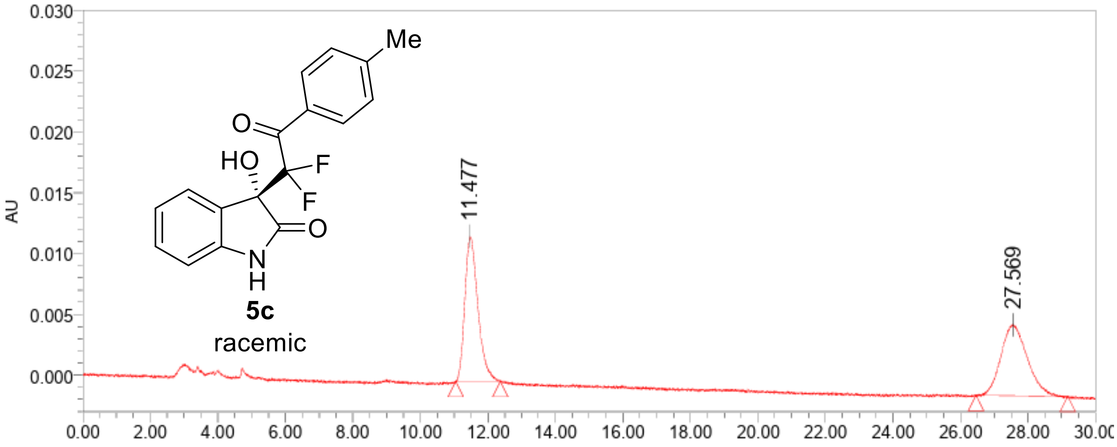

Entry	RT min	Height mV	Area mV.sec	% Area %
1	11.477	11925	318108	50.15
2	27.569	5835	316243	49.85

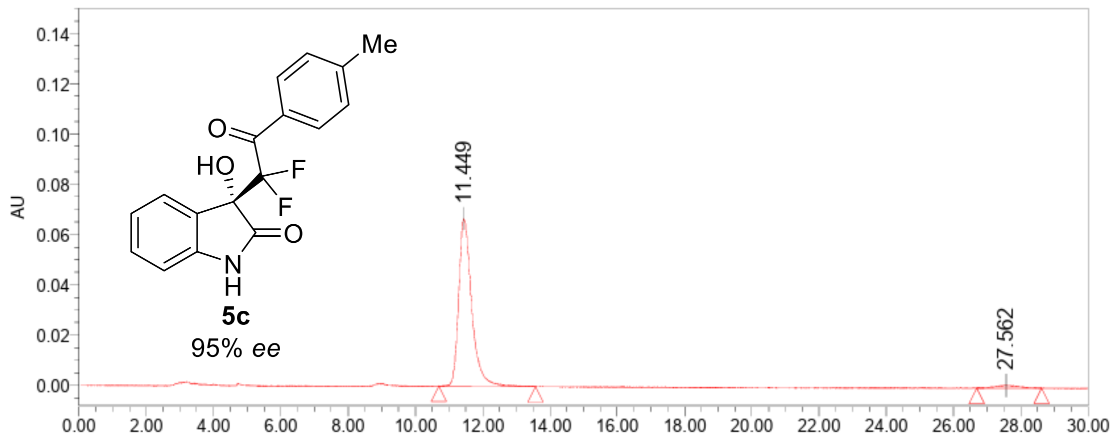

Entry	RT min	Height mV	Area mV.sec	% Area %
1	11.449	66650	1814340	97.47
2	27.562	1028	47120	2.53

Condition: Daicel Chiralpak IA, λ = 254 nm, hexane/2-propanol = 80:20

flow rate = 1.0 mL/min

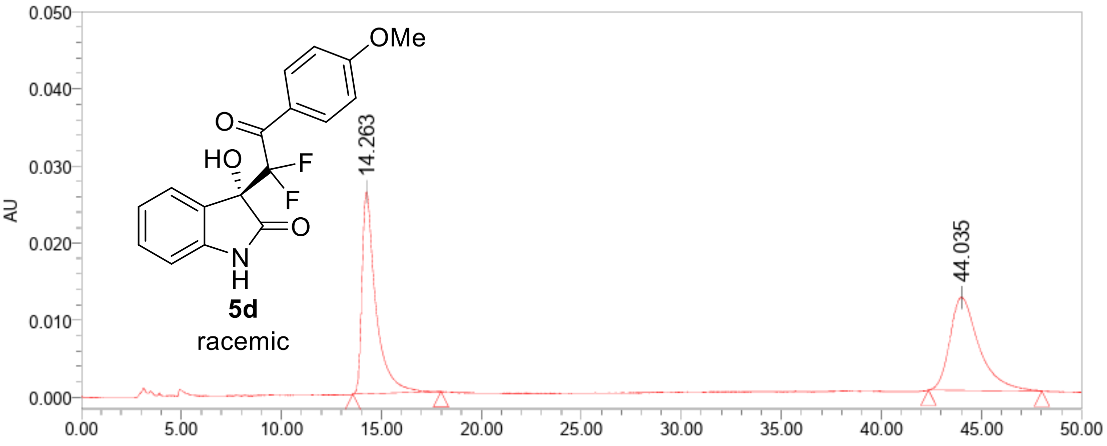

Entry	RT<br>min	Height<br>mV	Area<br>mV.sec	% Area<br>%
1	14.263	1257311	26205	50.57
2	44.035	1228748	12084	49.43

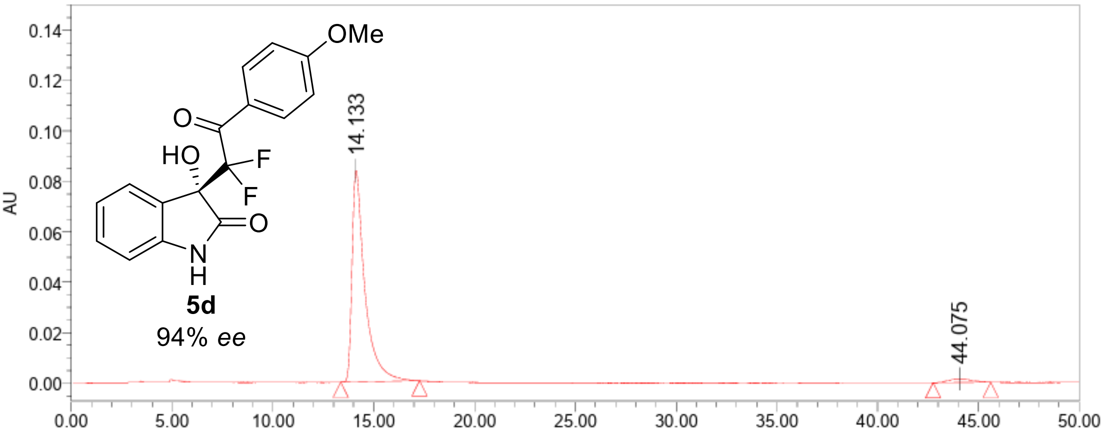

Entry	RT<br>min	Height<br>mV	Area<br>mV.sec	% Area<br>%
1	14.133	83878	3720674	96.98
2	44.075	1417	115804	3.02

Condition：Daicel Chiralpak IA, λ = 254 nm, hexane/2-propanol = 80:20

flow rate = 1.0 mL/min

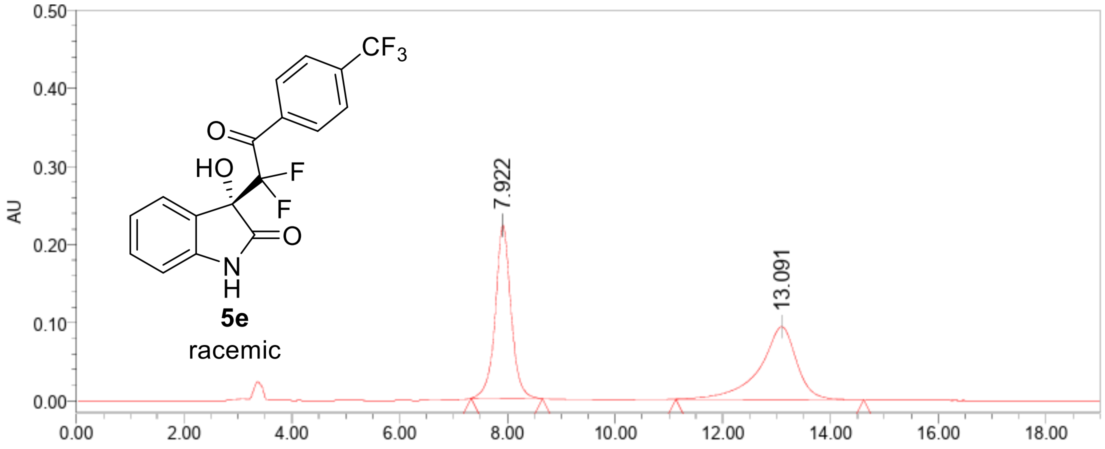

Entry	RT<br>min	Height<br>mV	Area<br>mV.sec	% Area<br>%
1	7.922	221616	4484633	50.25
2	13.091	93395	4440735	49.75

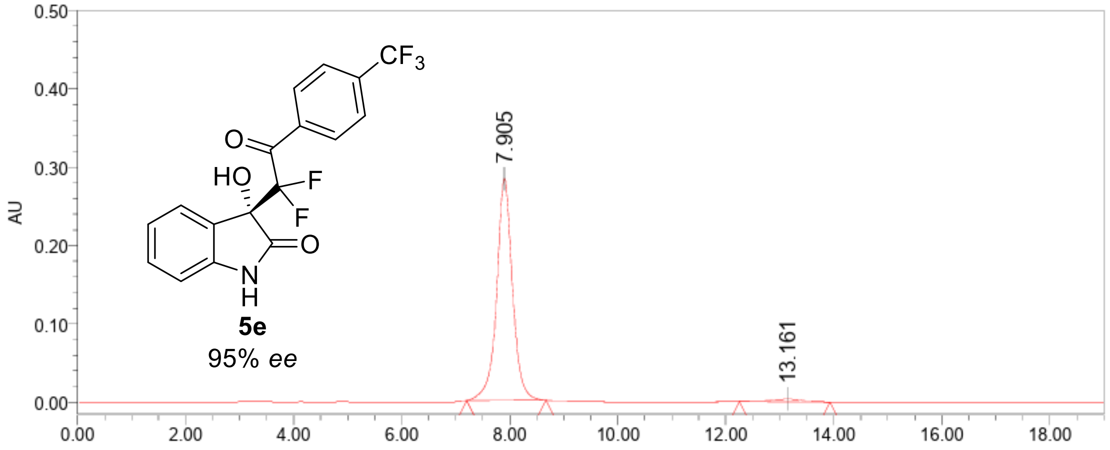

Entry	RT<br>min	Height<br>mV	Area<br>mV.sec	% Area<br>%
1	7.905	5771135	282835	97.69
2	13.161	136721	3472	2.31

Condition: Daicel Chiralpak IA, λ = 254 nm, hexane/2-propanol = 80:20

flow rate = 1.0 mL/min

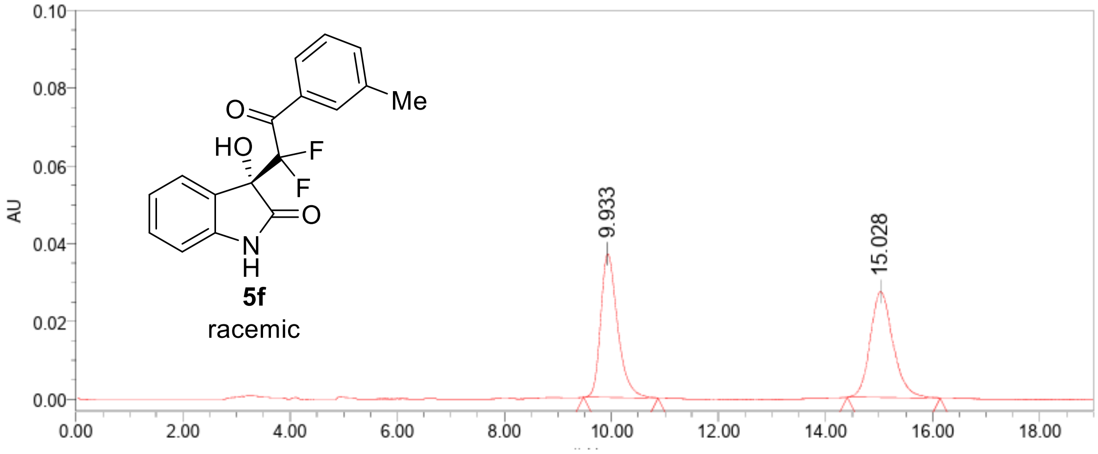

Entry	RT<br>min	Height<br>mV	Area<br>mV.sec	% Area<br>%
1	9.933	36982	809798	50.09
2	15.028	27177	806784	49.91

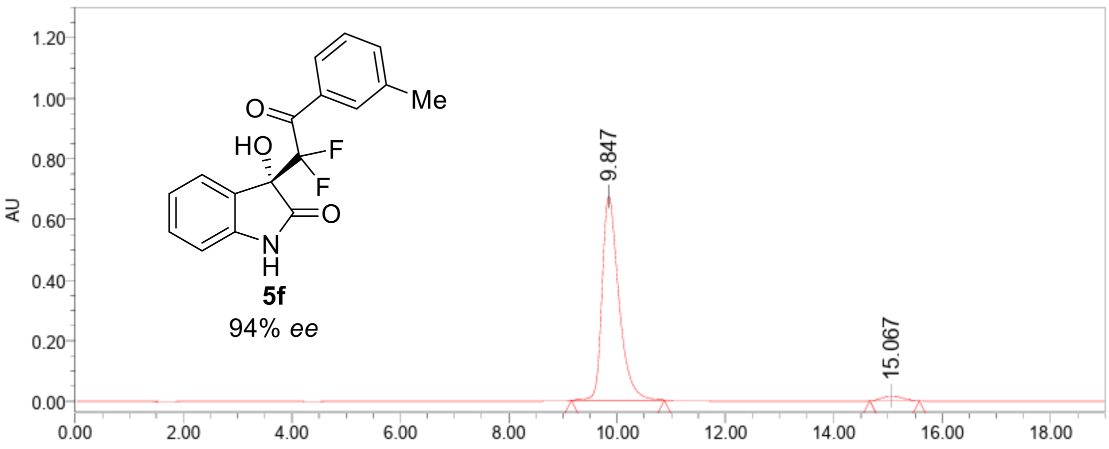

Entry	RT<br>min	Height<br>mV	Area<br>mV.sec	% Area<br>%
1	9.847	671844	14337396	97.18
2	15.067	15852	415941	2.82

Condition: Daicel Chiralpak IA, λ = 254 nm, hexane/2-propanol = 80:20

flow rate = 1.0 mL/min

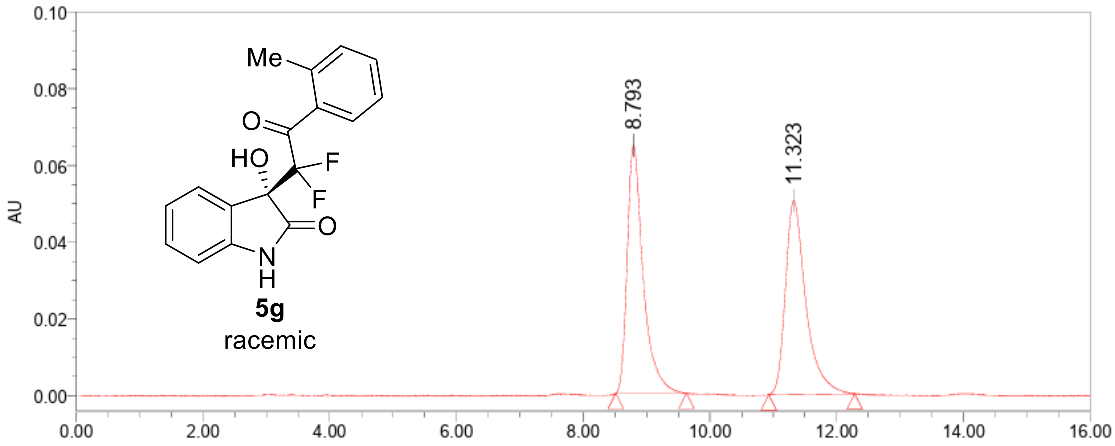

Entry	RT<br>min	Height<br>mV	Area<br>mV.sec	% Area<br>%
1	8.793	64635	1132827	50.14
2	11.323	50521	1126285	49.86

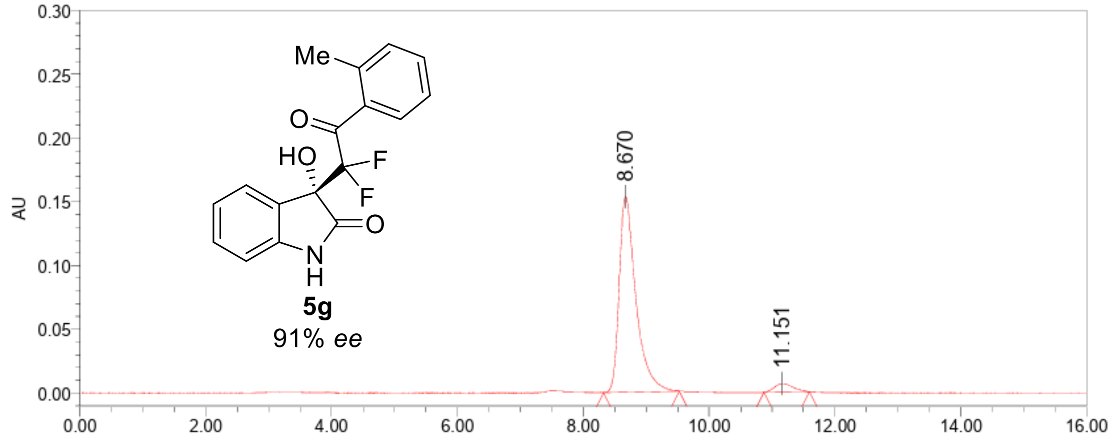

Entry	RT<br>min	Height<br>mV	Area<br>mV.sec	% Area<br>%
1	8.670	153224	2819810	95.42
2	11.151	6683	135398	4.58

Condition：Daicel Chiralpak IA, λ = 254 nm, hexane/2-propanol = 80:20

flow rate = 1.0 mL/min

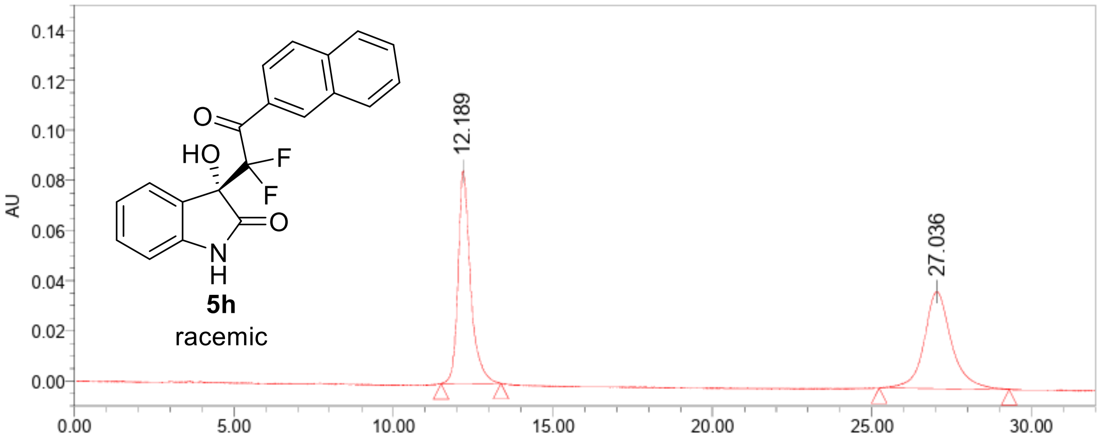

Entry	RT<br>min	Height<br>mV	Area<br>mV.sec	% Area<br>%
1	12.189	84829	2330341	50.39
2	27.036	38707	2294129	49.61

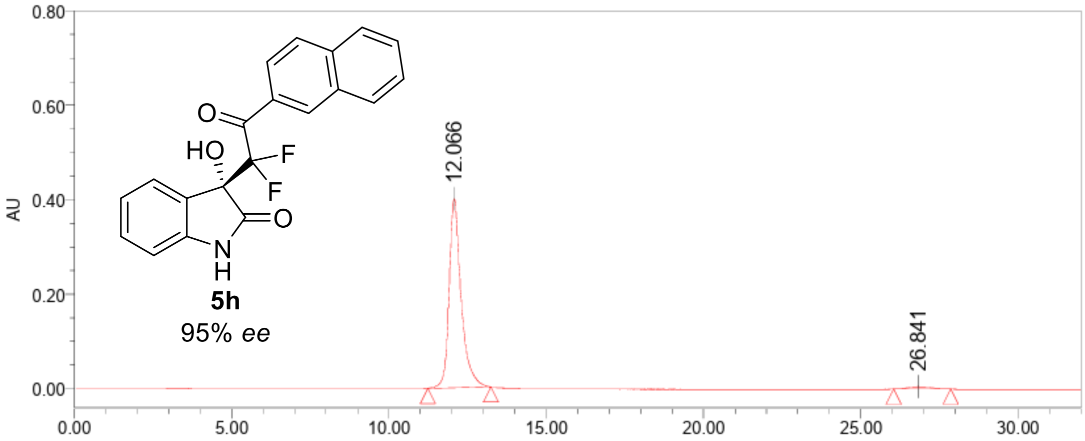

Entry	RT<br>min	Height<br>mV	Area<br>mV.sec	% Area<br>%
1	12.066	400526	10712310	97.65
2	26.841	257347	257347	2.35

Condition： Daicel Chiralpak IA, λ = 254 nm, hexane/2-propanol = 80:20

flow rate = 1.0 mL/min

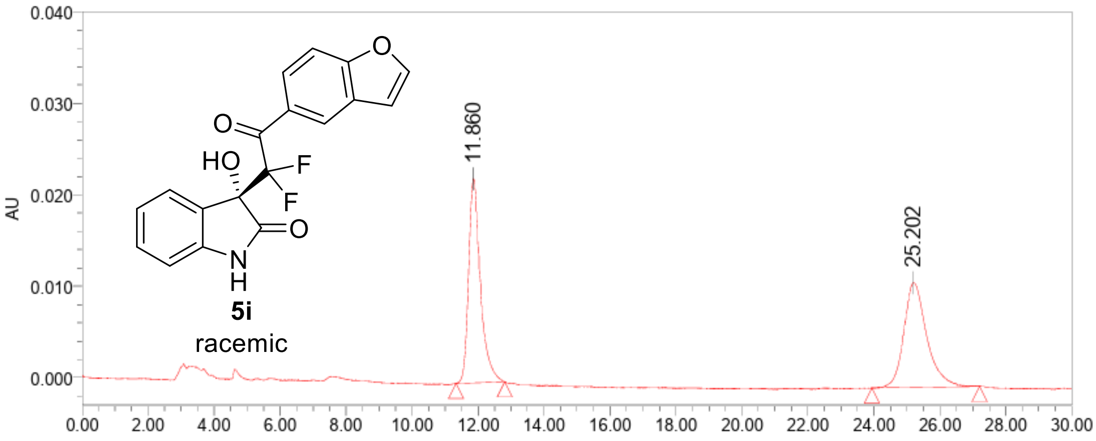

Entry	RT<br>min	Height<br>mV	Area<br>mV.sec	% Area<br>%
1	11.860	22334	560120	50.39
2	25.202	11460	551502	49.61

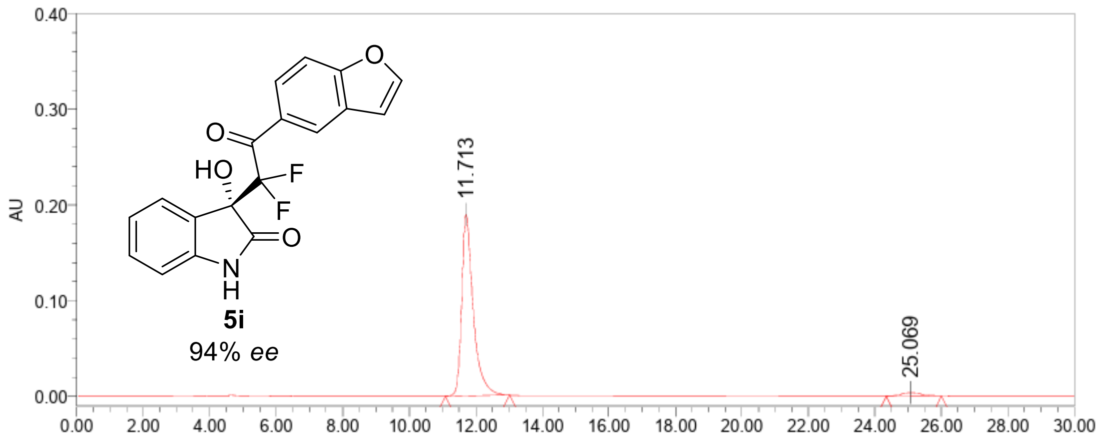

Entry	RT<br>min	Height<br>mV	Area<br>mV.sec	% Area<br>%
1	11.713	189718	4640484	97.04
2	25.069	3315	141749	2.96

Condition: Daicel Chiralpak IA, λ = 254 nm, hexane/2-propanol = 85:15

flow rate = 1.0 mL/min

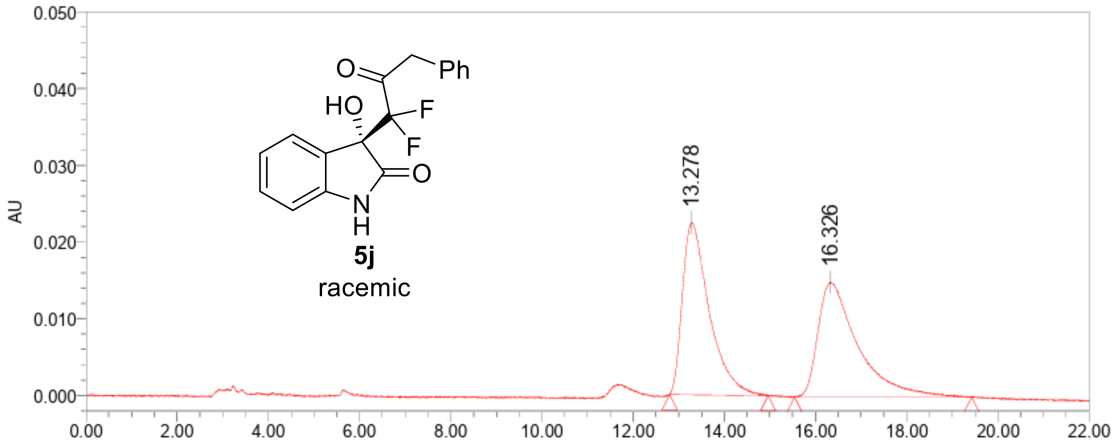

Entry	RT<br>min	Height<br>mV	Area<br>mV.sec	% Area<br>%
1	13.278	22388	913140	50.05
2	16.326	15000	911423	49.95

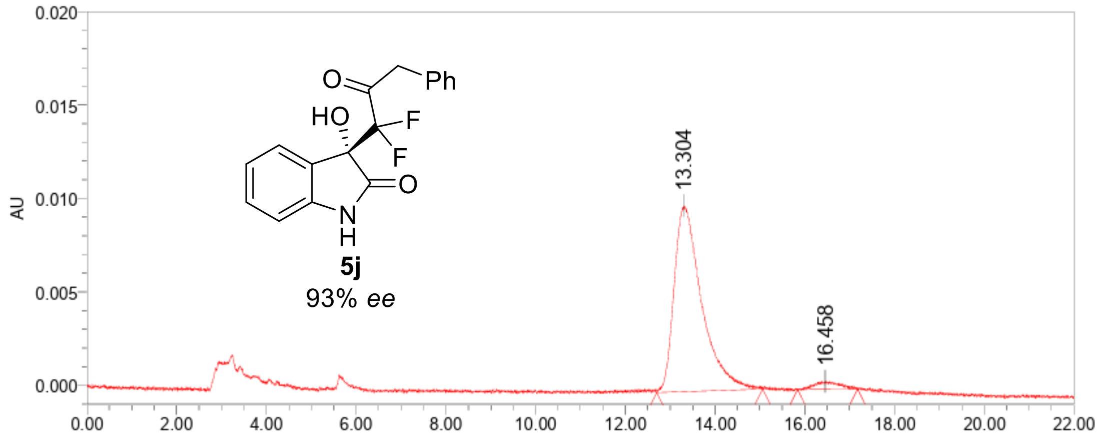

Entry	RT<br>min	Height<br>mV	Area<br>mV.sec	% Area<br>%
1	13.304	9954	423227	96.38
2	16.458	430	15904	3.62

Condition：Daicel Chiralpak OD-H, λ = 254 nm, hexane/2-propanol = 85:15

flow rate = 1.0 mL/min

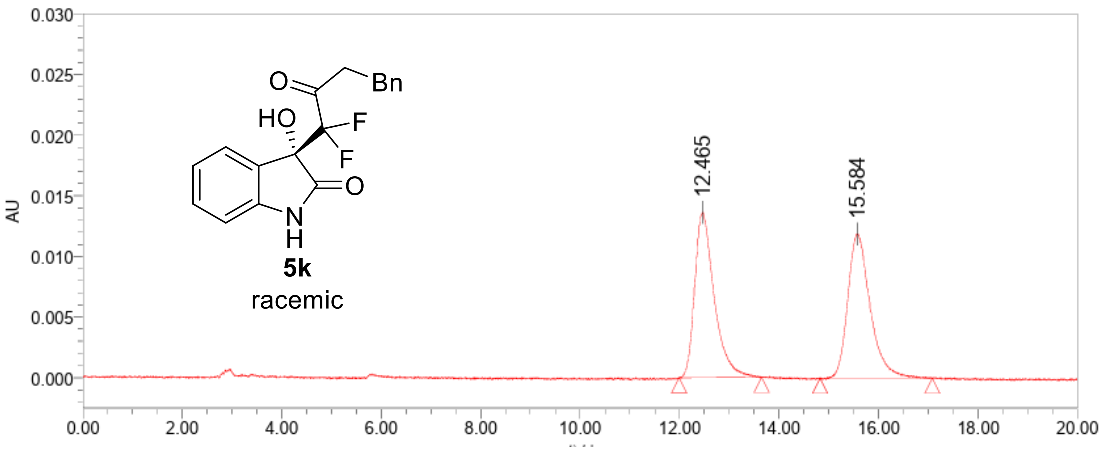

Entry	RT<br>min	Height<br>mV	Area<br>mV.sec	% Area<br>%
1	12.465	13630	372799	49.80
2	15.584	11909	375825	50.20

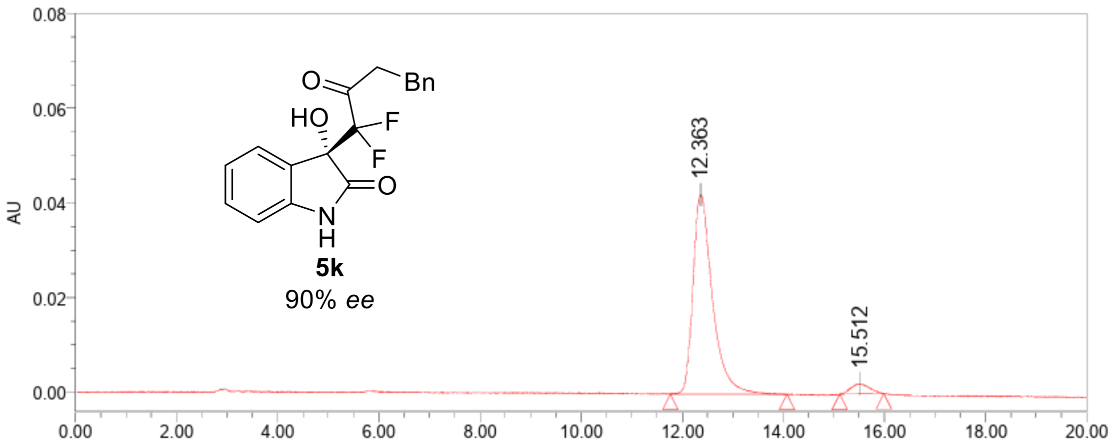

Entry	RT<br>min	Height<br>mV	Area<br>mV.sec	% Area<br>%
1	12.363	42112	1135022	95.29
2	15.512	2161	56080	4.71

Condition： Daicel Chiralpak AD-H, λ = 254 nm, hexane/2-propanol = 75:25

flow rate = 1.0 mL/min

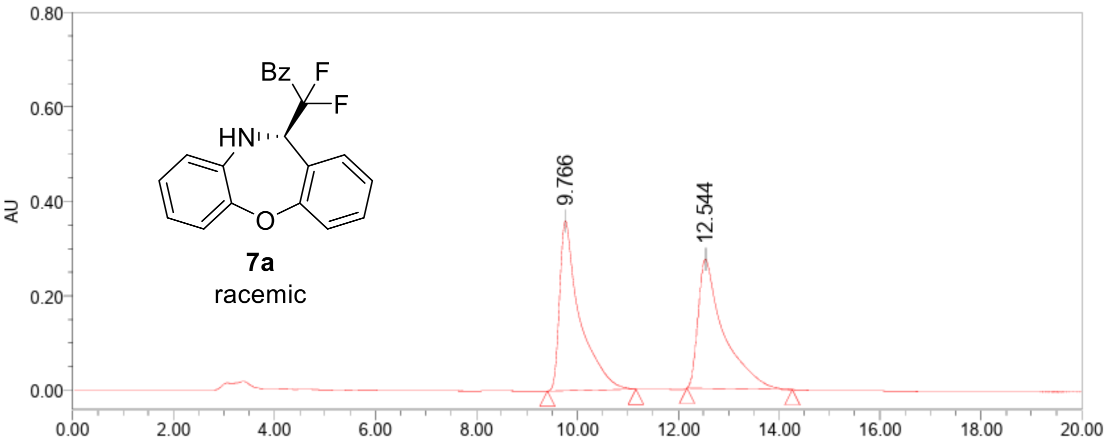

Entry	RT min	Height mV	Area mV.sec	% Area %
1	9.766	359120	9941337	50.81
2	12.544	272864	9623532	49.19

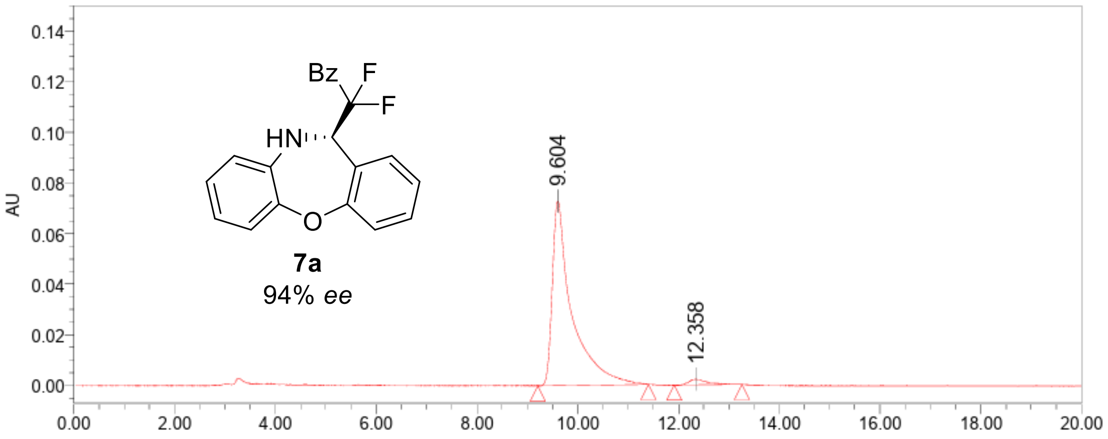

Entry	RT min	Height mV	Area mV.sec	% Area %
1	9.604	72873	1966473	97.22
2	12.358	2269	56199	2.78

Condition：Daicel Chiralpak AD-H, λ = 254 nm, hexane/2-propanol = 75:25

flow rate = 1.0 mL/min

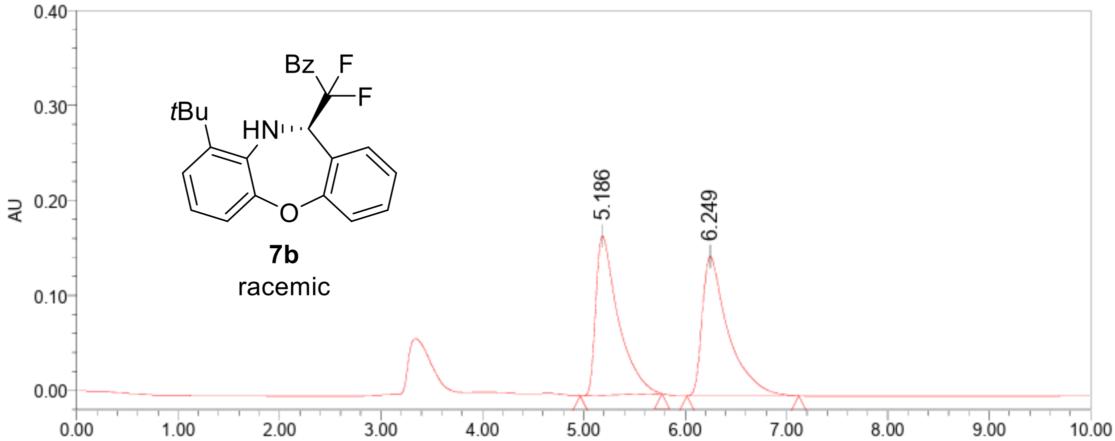

Entry	RT min	Height mV	Area mV.sec	% Area %
1	5.186	167526	2566508	49.40
2	6.249	146801	2629065	50.60

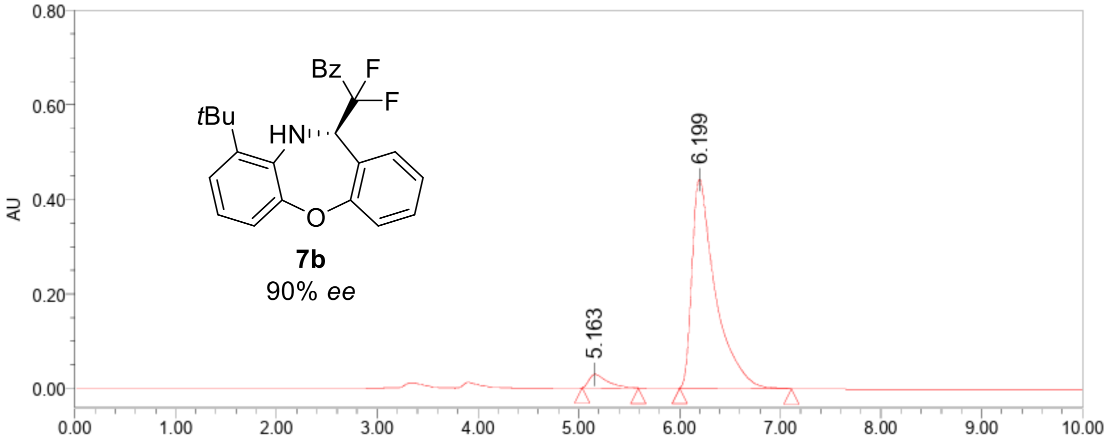

Entry	RT min	Height mV	Area mV.sec	% Area %
1	5.163	29092	369344	4.90
2	6.199	441568	7162957	95.10

Condition：Daicel Chiralpak IA, λ = 254 nm, hexane/2-propanol = 90:10

flow rate = 1.0 mL/min

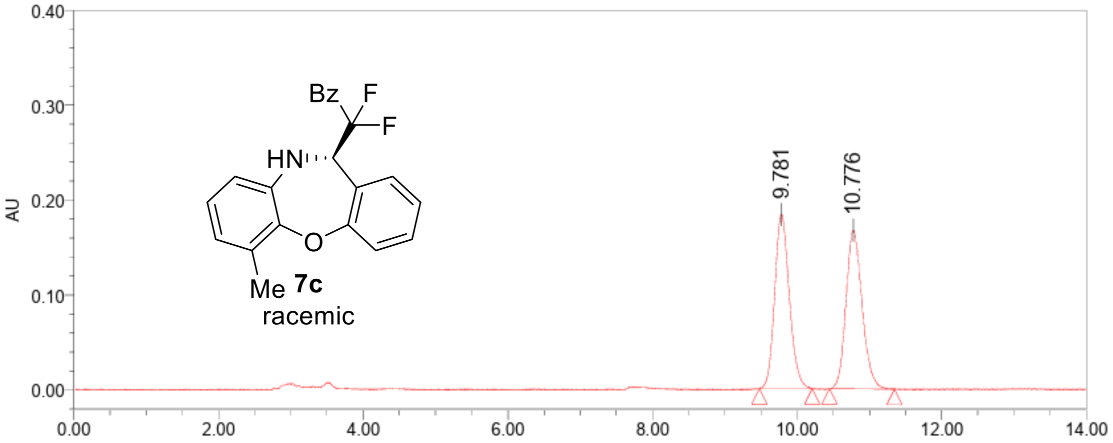

Entry	RT<br>min	Height<br>mV	Area<br>mV.sec	% Area<br>%
1	9.781	183815	2573777	49.88
2	10.776	167112	2585924	50.12

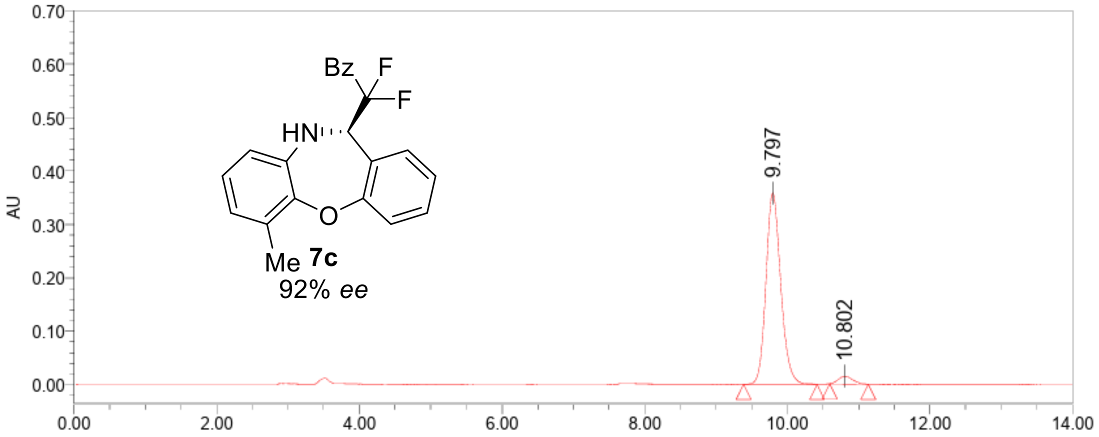

Entry	RT<br>min	Height<br>mV	Area<br>mV.sec	% Area<br>%
1	9.797	358138	5142659	96.17
2	10.802	14356	204879	3.83

Condition: Daicel Chiralpak IA-3, λ = 254 nm, hexane/2-propanol = 85:15

flow rate = 1.0 mL/min

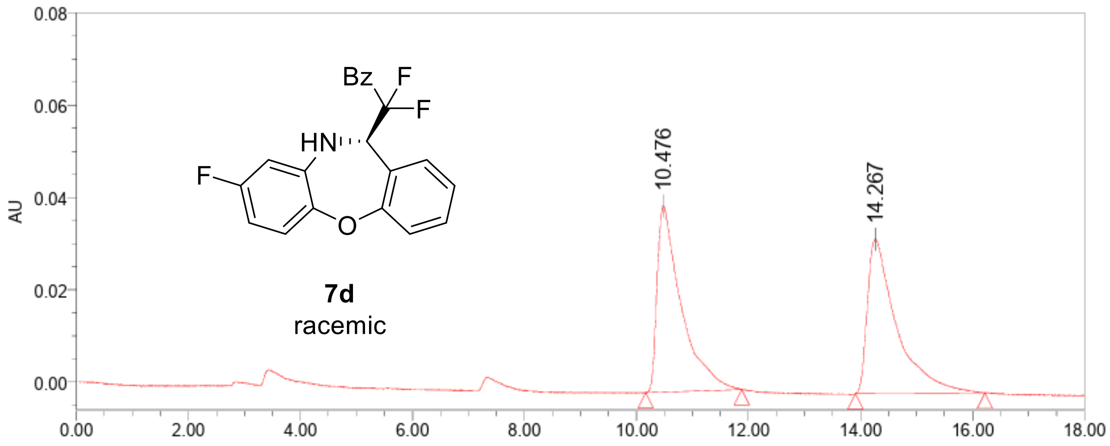

Entry	RT<br>min	Height<br>mV	Area<br>mV.sec	% Area<br>%
1	10.476	40340	1208099	49.85
2	14.267	33447	1215434	50.15

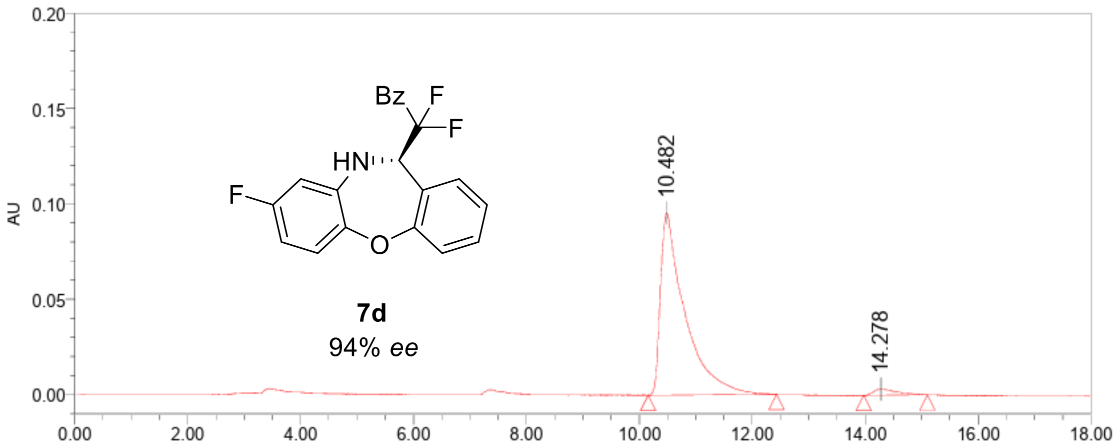

Entry	RT<br>min	Height<br>mV	Area<br>mV.sec	% Area<br>%
1	10.482	95406	2878085	96.83
2	14.278	3353	94230	3.17

Condition：Daicel Chiralpak IA, λ = 254 nm, hexane/2-propanol = 90:10

flow rate = 1.0 mL/min

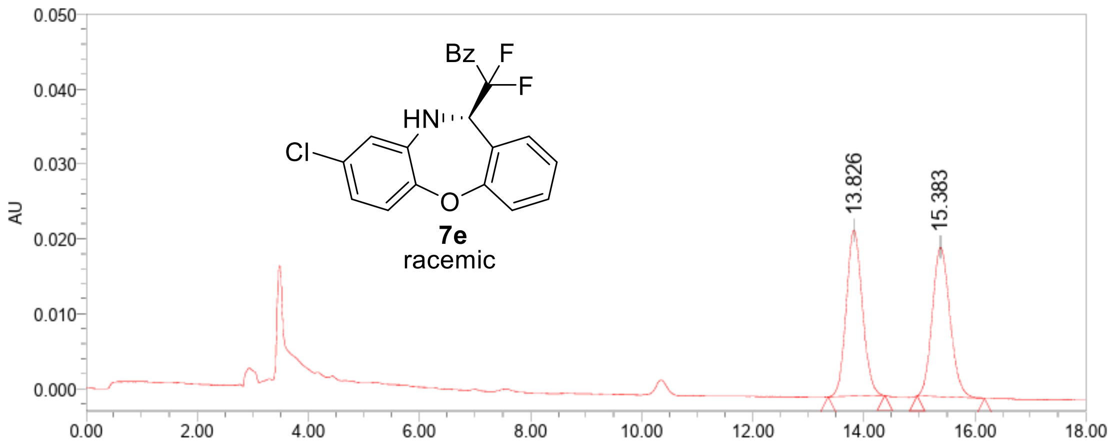

Entry	RT<br>min	Height<br>mV	Area<br>mV.sec	% Area<br>%
1	13.826	22163	443635	50.18
2	15.383	19925	440511	49.82

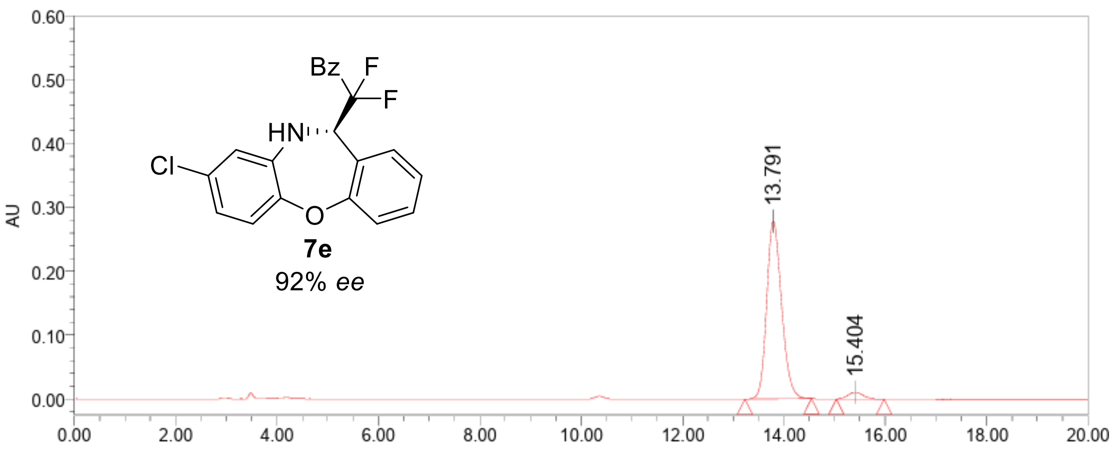

Entry	RT<br>min	Height<br>mV	Area<br>mV.sec	% Area<br>%
1	13.791	278441	5666392	96.21
2	15.404	10441	223039	3.79

Condition：Daicel Chiralpak IA, λ = 254 nm, hexane/2-propanol = 80:20

flow rate = 1.0 mL/min

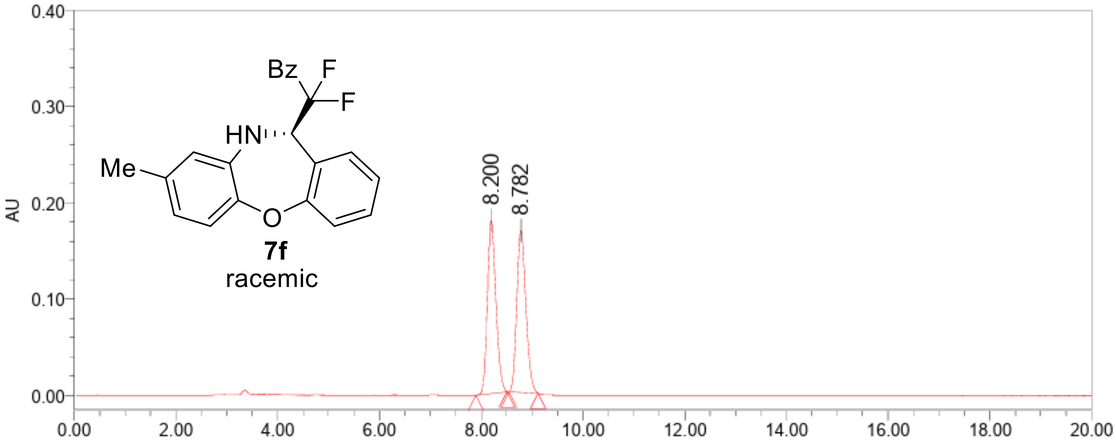

Entry	RT<br>min	Height<br>mV	Area<br>mV.sec	% Area<br>%
1	8.200	179802	2092262	50.17
2	8.782	168343	2078265	49.83

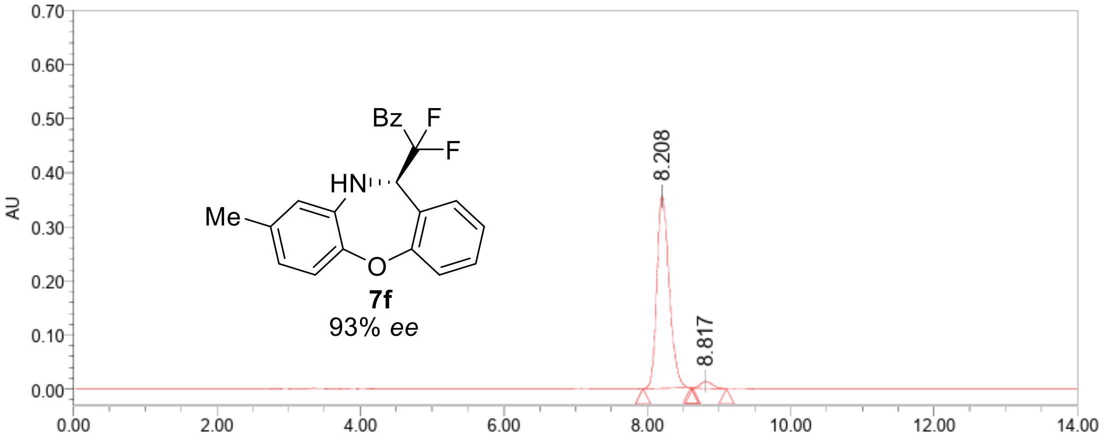

Entry	RT<br>min	Height<br>mV	Area<br>mV.sec	% Area<br>%
1	8.208	355398	4262316	96.69
2	8.817	12665	145963	3.31

Condition: Daicel Chiralpak IA-3, λ = 254 nm, hexane/2-propanol = 85:15

flow rate = 1.0 mL/min

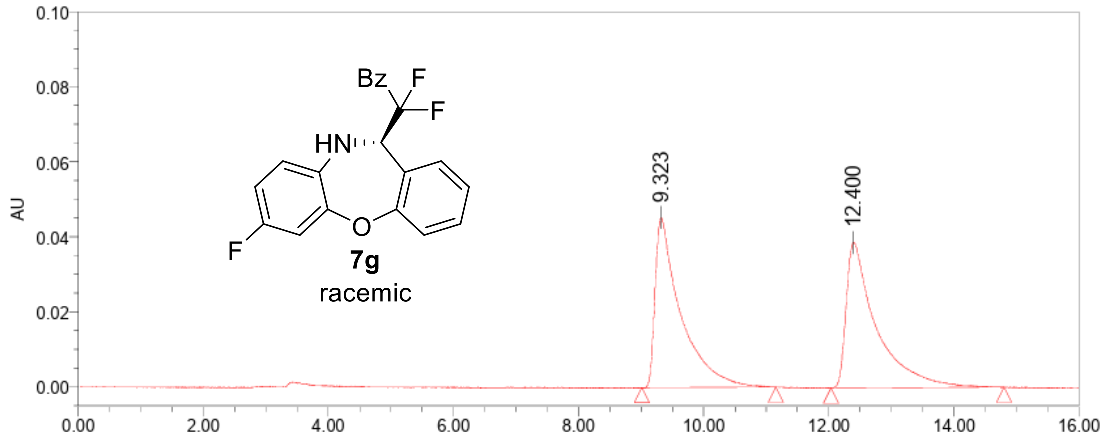

Entry	RT<br>min	Height<br>mV	Area<br>mV.sec	% Area<br>%
1	9.323	45381	1293655	49.83
2	12.400	38798	1302392	50.17

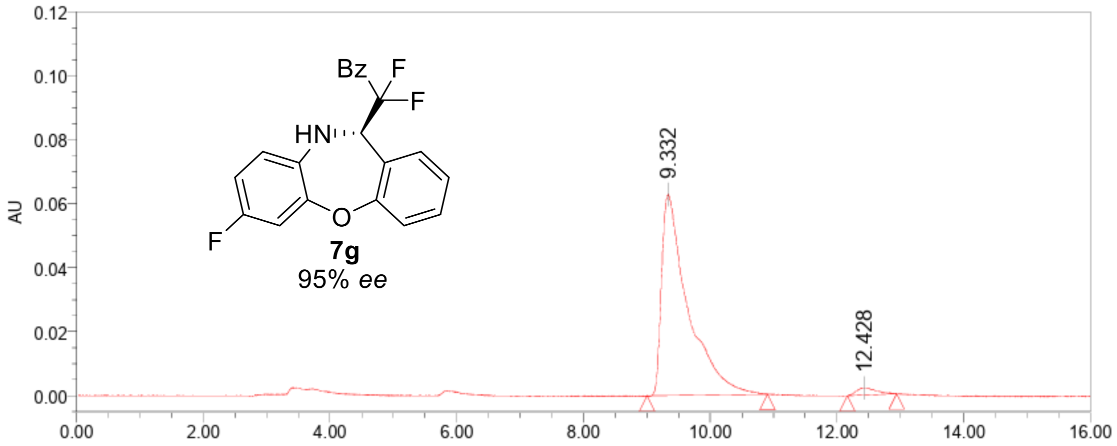

Entry	RT<br>min	Height<br>mV	Area<br>mV.sec	% Area<br>%
1	9.332	62987	1820498	97.48
2	12.428	2208	47099	2.52

Condition：Daicel Chiralpak IA, λ = 254 nm, hexane/2-propanol = 90:10

flow rate = 1.0 mL/min

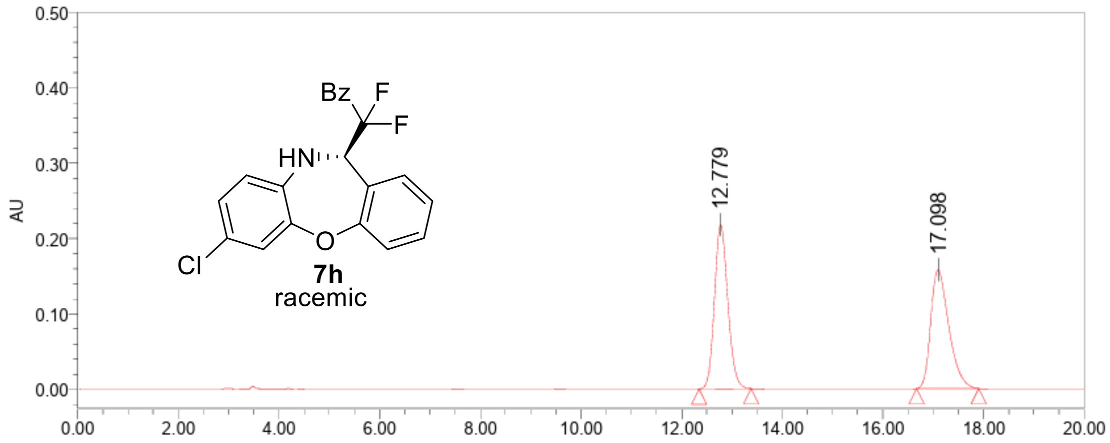

Entry	RT<br>min	Height<br>mV	Area<br>mV.sec	% Area<br>%
1	12.779	217791	4007875	50.34
2	17.098	157724	3954106	49.66

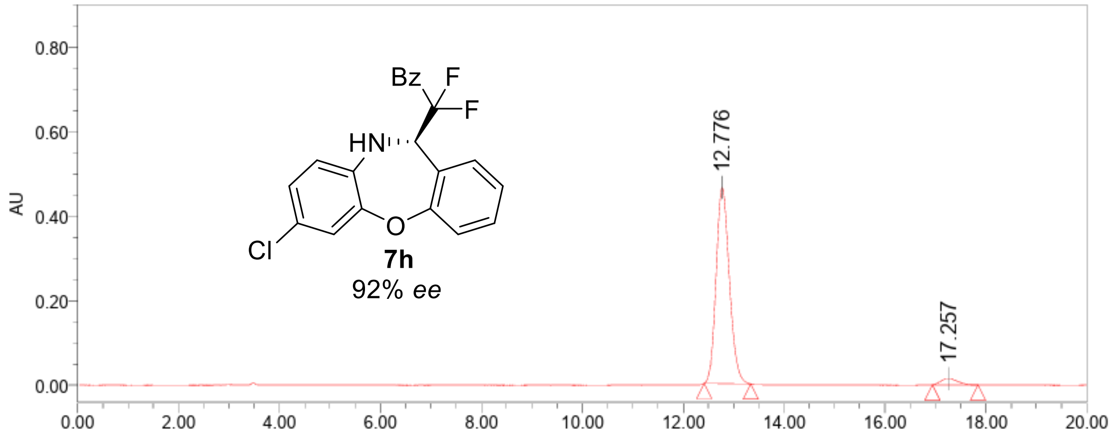

Entry	RT<br>min	Height<br>mV	Area<br>mV.sec	% Area<br>%
1	12.776	463777	8434526	96.14
2	17.257	14675	339009	3.86

Condition：Daicel Chiralpak IA, λ = 254 nm, hexane/2-propanol = 90:10

flow rate = 1.0 mL/min

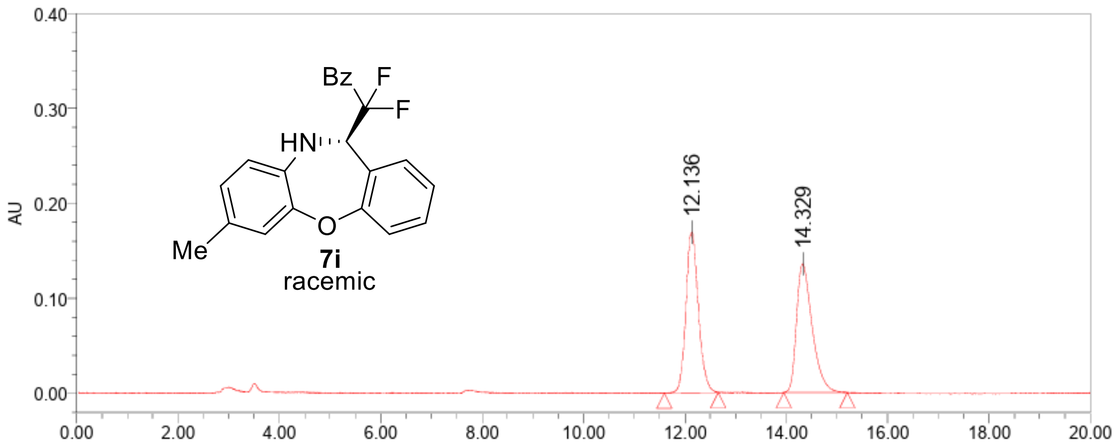

Entry	RT<br>min	Height<br>mV	Area<br>mV.sec	% Area<br>%
1	12.136	169132	2908520	50.04
2	14.329	135703	2904406	49.96

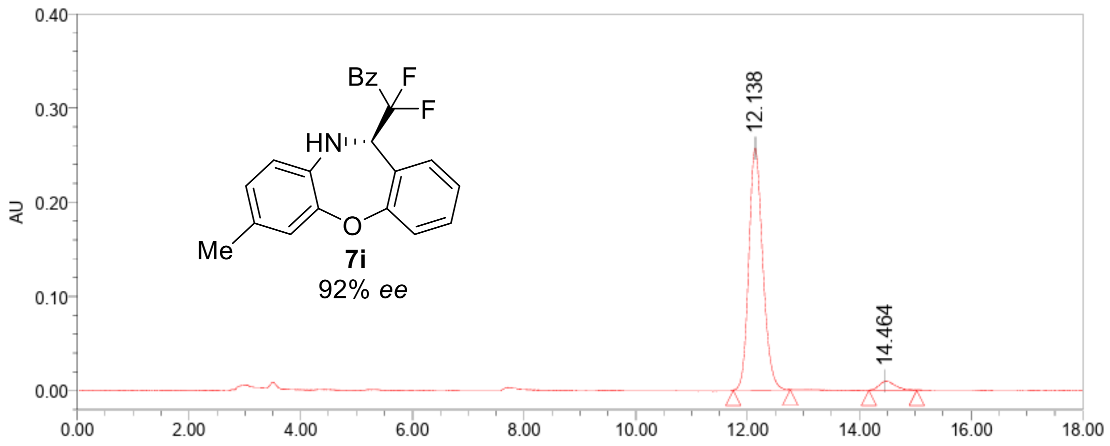

Entry	RT<br>min	Height<br>mV	Area<br>mV.sec	% Area<br>%
1	12.138	256721	4442788	95.92
2	14.464	9407	189085	4.08

Condition：Daicel Chiralpak IA, λ = 254 nm, hexane/2-propanol = 90:10

flow rate = 1.0 mL/min

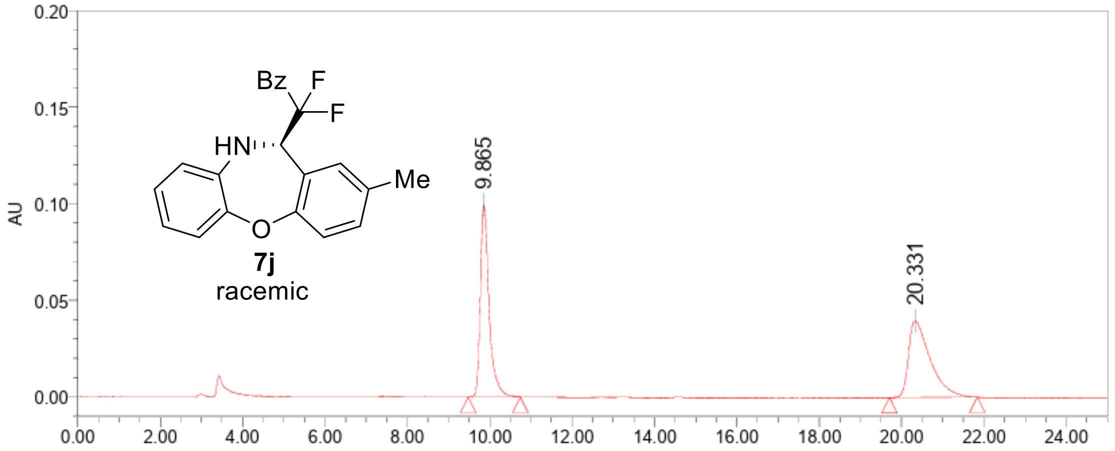

Entry	RT<br>min	Height<br>mV	Area<br>mV.sec	% Area<br>%
1	9.865	99433	1475196	50.31
2	20.331	39721	1456820	49.69

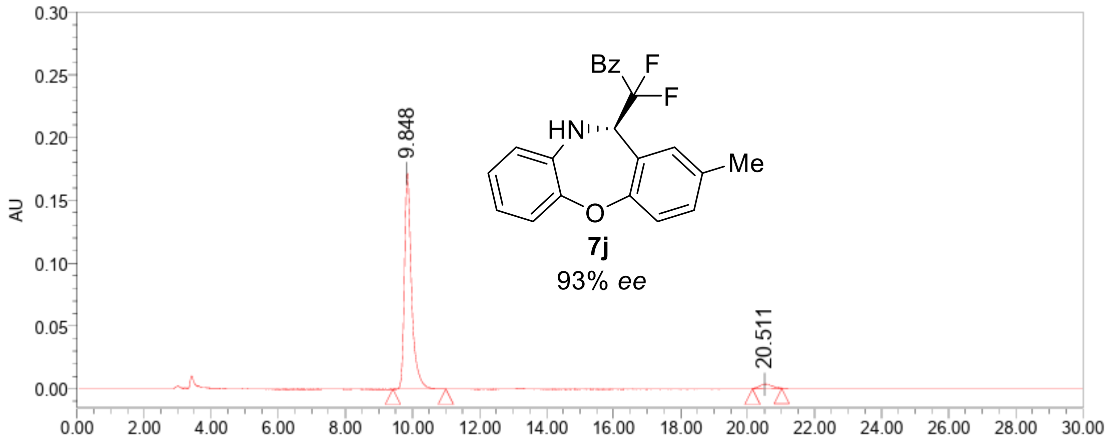

Entry	RT<br>min	Height<br>mV	Area<br>mV.sec	% Area<br>%
1	9.848	171650	2530111	96.52
2	20.511	3553	91137	3.48

Condition： Daicel Chiralpak ID-3, λ = 254 nm, hexane/2-propanol = 97:03

flow rate = 1.0 mL/min

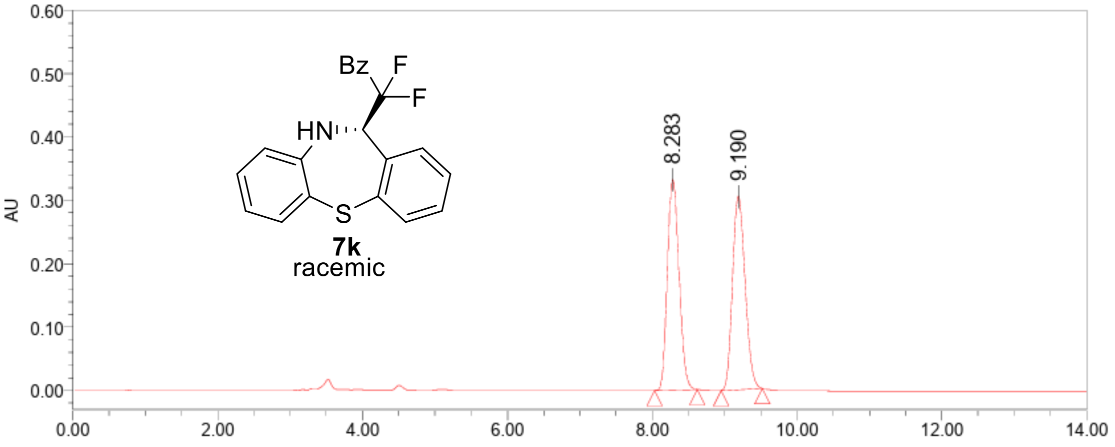

Entry	RT min	Height mV	Area mV.sec	% Area %
1	8.283	331995	3735675	50.38
2	9.190	304755	3679311	49.62

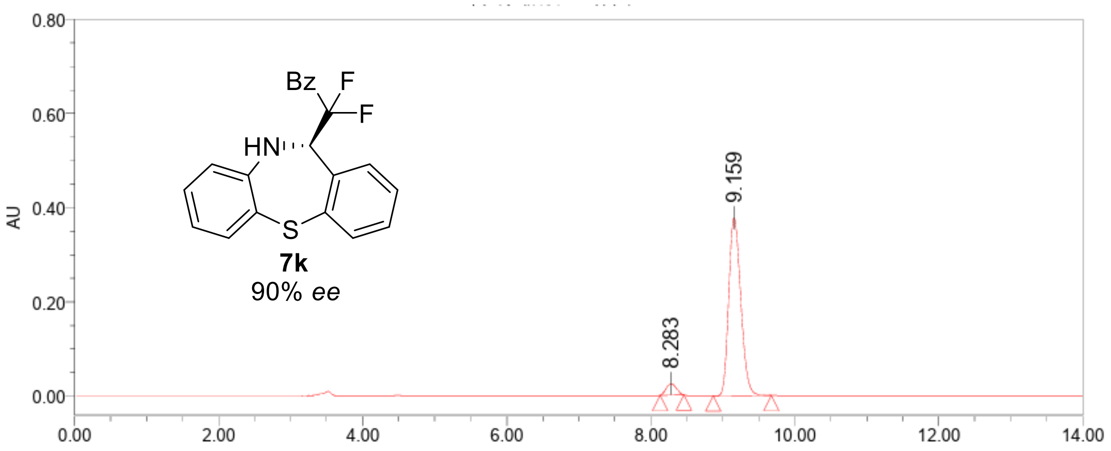

Entry	RT min	Height mV	Area mV.sec	% Area %
1	8.283	24219	241782	5.23
2	9.159	377560	4381312	94.77

Condition：Daicel Chiralpak IA, λ = 254 nm, hexane/2-propanol = 90:10

flow rate = 1.0 mL/min

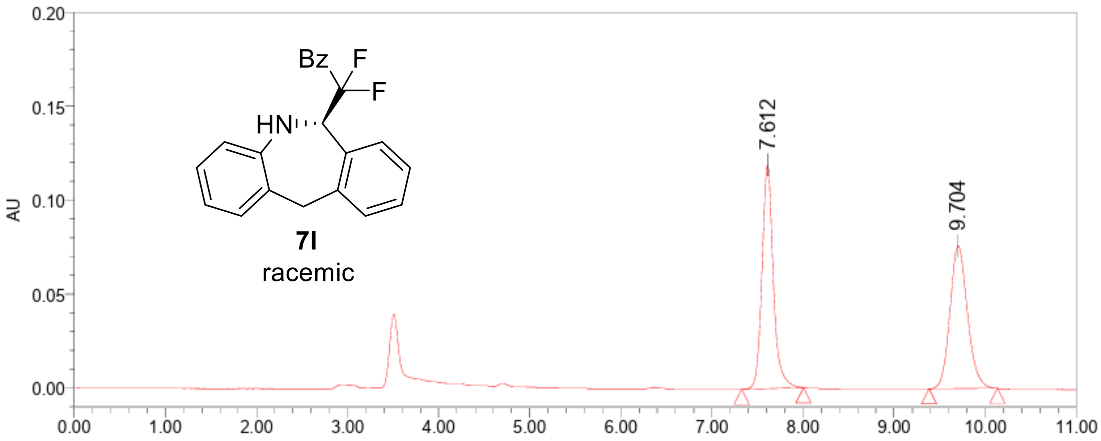

Entry	RT<br>min	Height<br>mV	Area<br>mV.sec	% Area<br>%
1	7.612	119000	1027105	50.24
2	9.704	76090	1017248	49.76

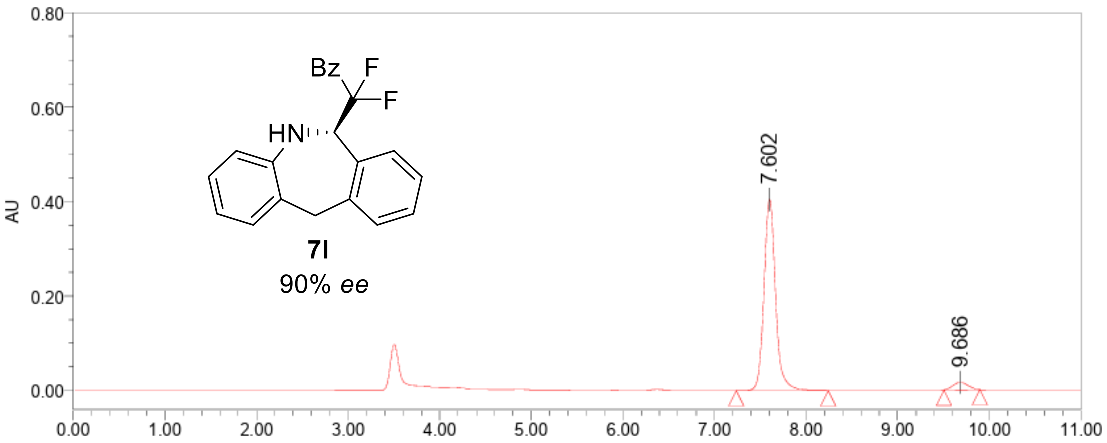

Entry	RT<br>min	Height<br>mV	Area<br>mV.sec	% Area<br>%
1	7.602	404812	3559943	95.08
2	9.686	15924	184239	4.92

Condition: Daicel Chiralpak IA, λ = 254 nm, hexane/2-propanol = 80:20

flow rate = 1.0 mL/min

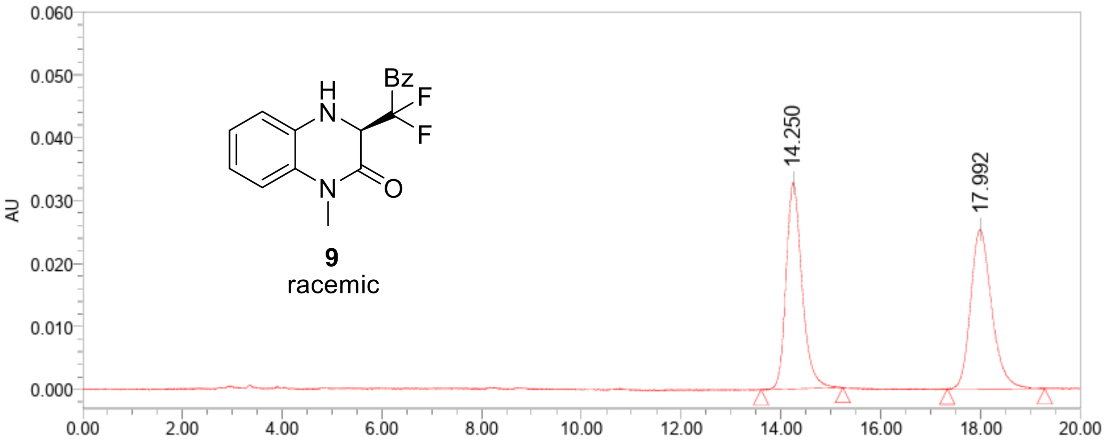

Entry	RT<br>min	Height<br>mV	Area<br>mV.sec	% Area<br>%
1	14.250	32791	728848	49.69
2	17.992	25335	738010	50.31

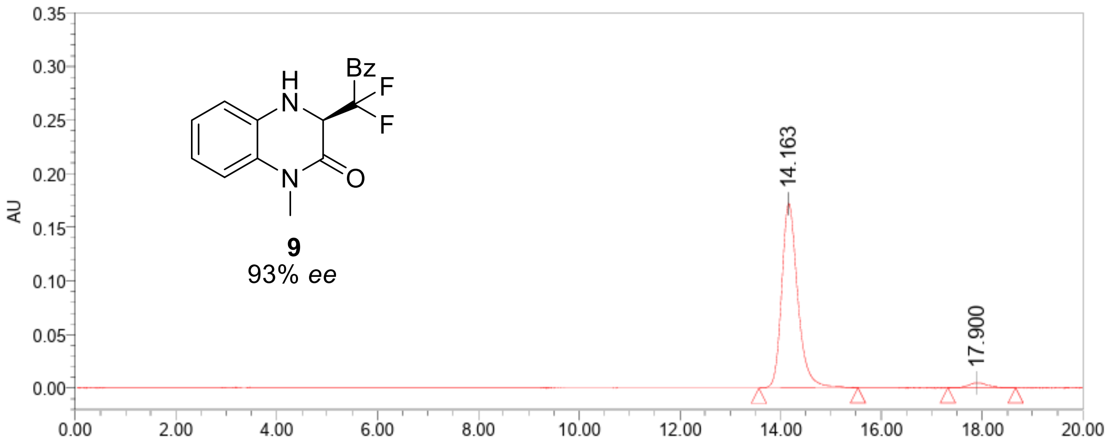

Entry	RT<br>min	Height<br>mV	Area<br>mV.sec	% Area<br>%
1	14.163	171995	3791165	96.64
2	17.900	4701	131818	3.36

Condition: Daicel Chiralpak AD-H, λ = 254 nm, hexane/2-propanol = 75:25

flow rate = 1.0 mL/min

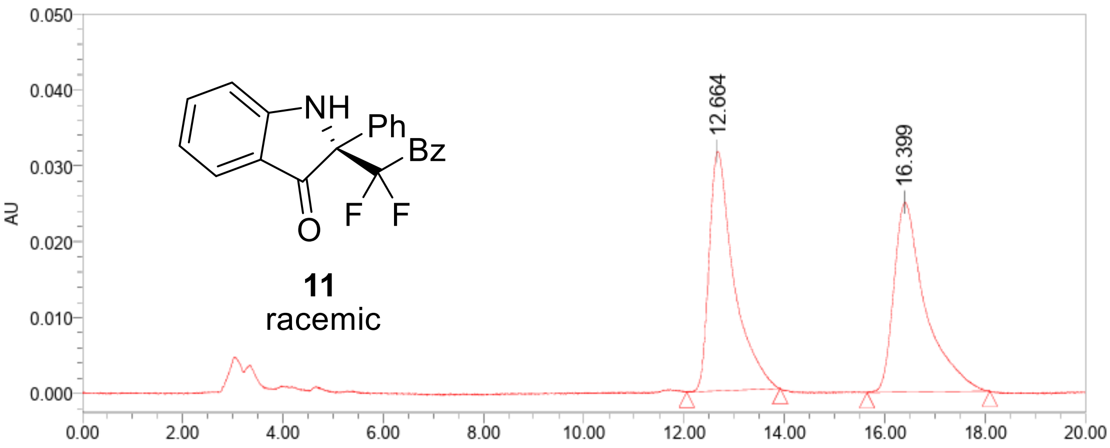

Entry	RT<br>min	Height<br>mV	Area<br>mV.sec	% Area<br>%
1	12.664	31560	1079932	49.66
2	16.399	25003	1094681	50.34

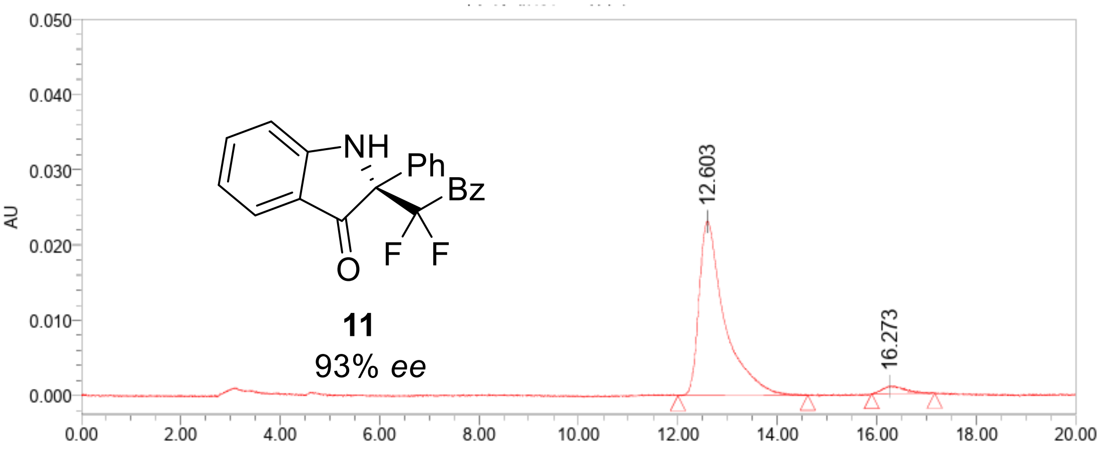

Entry	RT<br>min	Height<br>mV	Area<br>mV.sec	% Area<br>%
1	12.603	23176	828203	96.39
2	16.273	994	31034	3.61

Condition: Daicel Chiralpak AD-H, λ = 254 nm, hexane/2-propanol = 90:10

flow rate = 1.0 mL/min

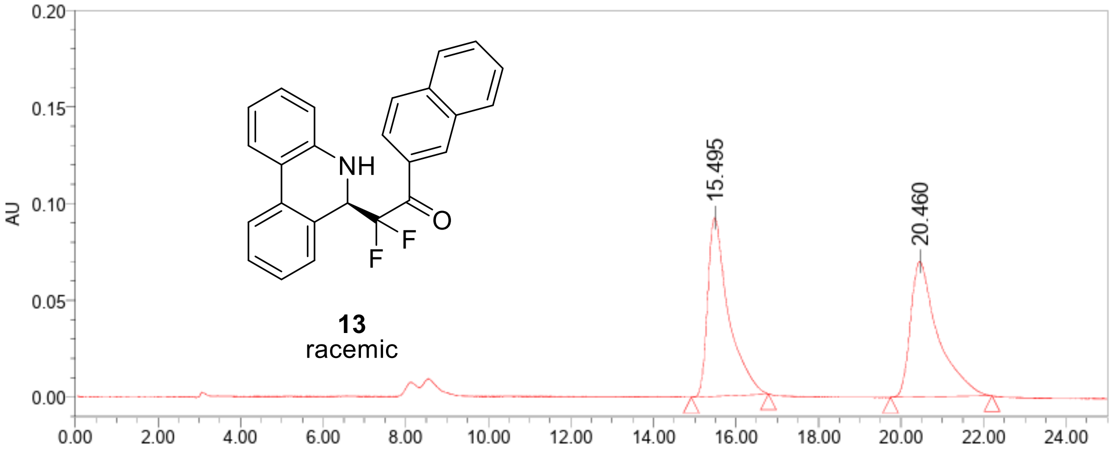

Entry	RT<br>min	Height<br>mV	Area<br>mV.sec	% Area<br>%
1	15.495	92331	3250988	49.91
2	20.460	70203	3262715	50.09

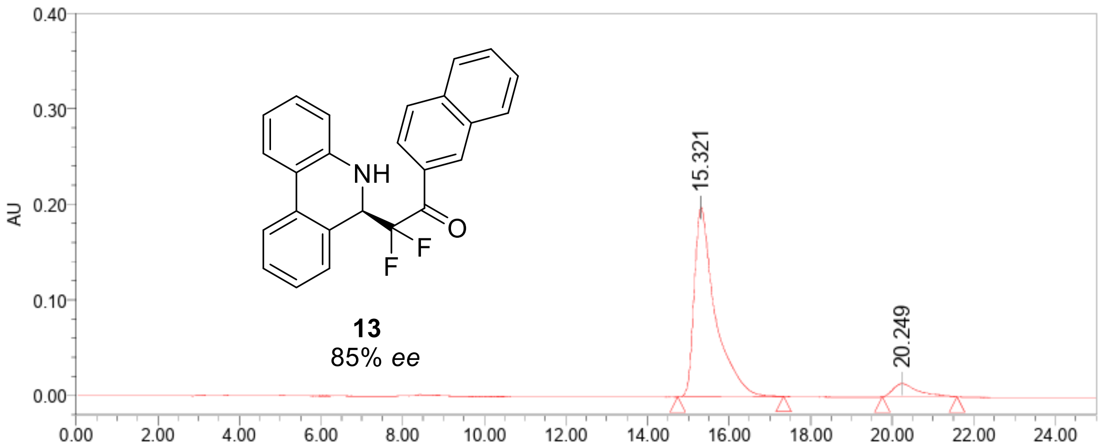

Entry	RT<br>min	Height<br>mV	Area<br>mV.sec	% Area<br>%
1	15.321	197973	7096566	92.59
2	20.249	13706	568026	7.41

Condition：Daicel Chiralpak IA, λ = 254 nm, hexane/2-propanol = 70:30

flow rate = 1.0 mL/min

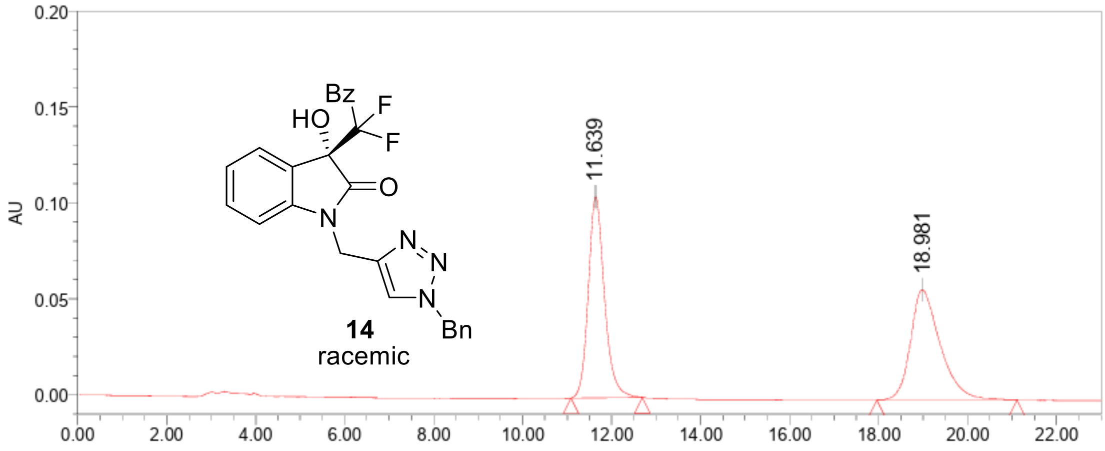

Entry	RT<br>min	Height<br>mV	Area<br>mV.sec	% Area<br>%
1	11.639	104769	2641562	49.66
2	18.981	57527	2677414	50.34

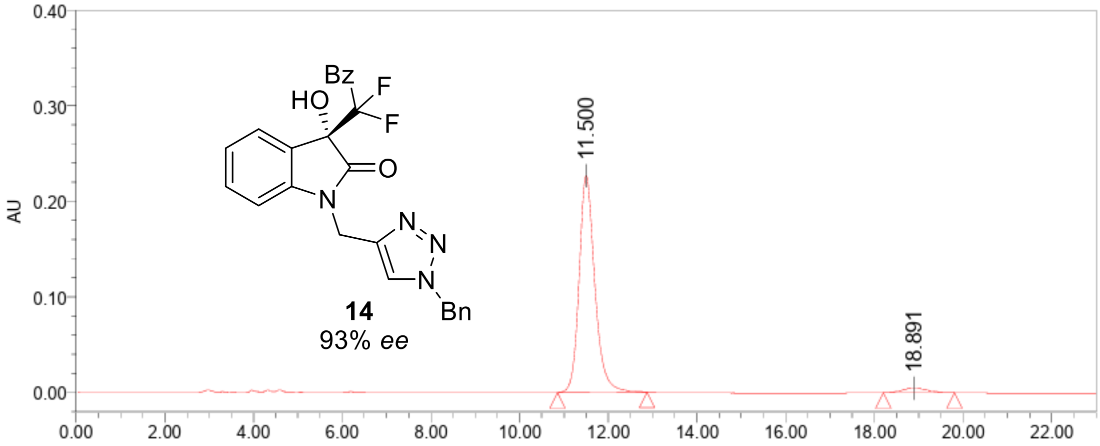

Entry	RT<br>min	Height<br>mV	Area<br>mV.sec	% Area<br>%
1	11.500	226835	5569895	96.59
2	18.891	4946	196376	3.41

Condition: Daicel Chiralpak IF-3, λ = 254 nm, hexane/2-propanol = 99:01

flow rate = 1.0 mL/min

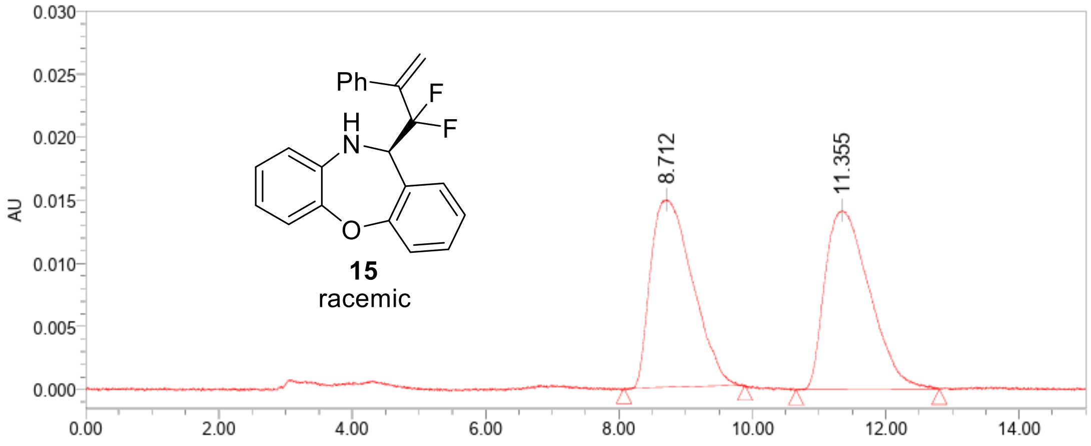

Entry	RT<br>min	Height<br>mV	Area<br>mV.sec	% Area<br>%
1	8.712	14877	652289	49.66
2	11.355	14187	661235	50.34

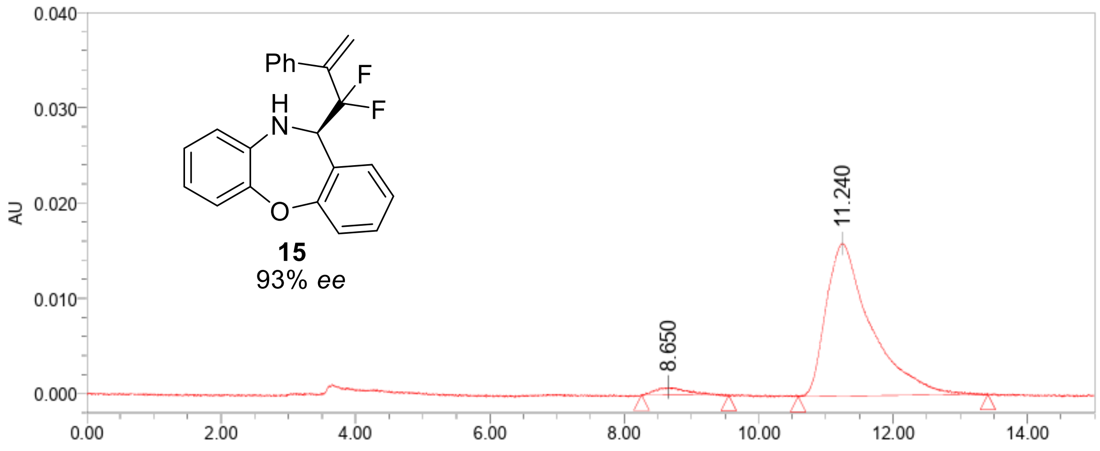

Entry	RT<br>min	Height<br>mV	Area<br>mV.sec	% Area<br>%
1	8.650	853	28239	3.65
2	11.240	16014	746095	96.35

Condition: Daicel Chiralpak OD-H, λ = 254 nm, hexane/2-propanol = 80:20

flow rate = 1.0 mL/min

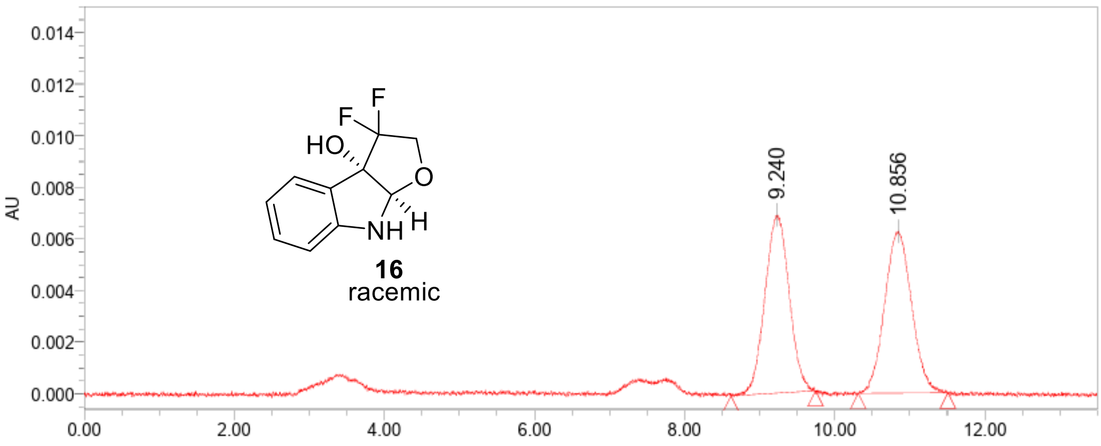

Entry	RT<br>min	Height<br>mV	Area<br>mV.sec	% Area<br>%
1	9.240	6894	153875	49.52
2	10.856	6275	156880	50.48

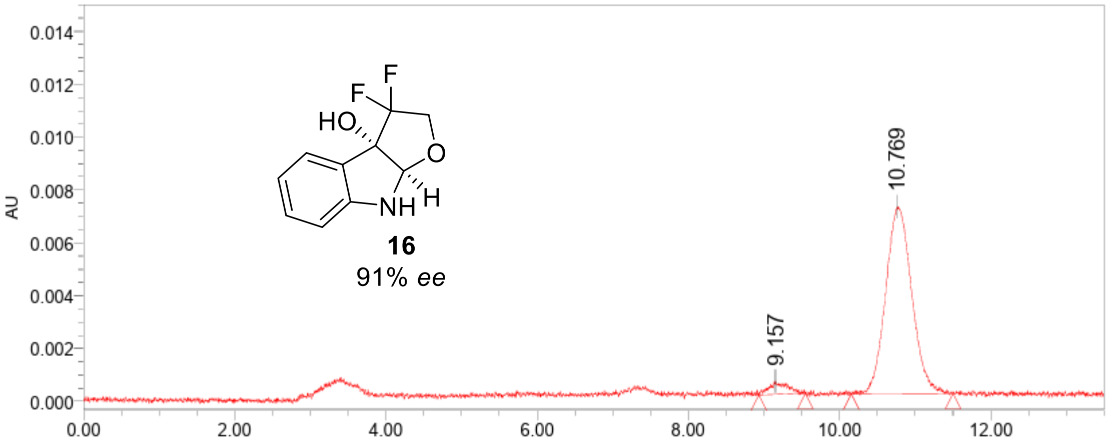

Entry	RT<br>min	Height<br>mV	Area<br>mV.sec	% Area<br>%
1	9.157	495	8154	4.59
2	10.769	7065	169609	95.41

Condition：Daicel Chiralpak OD-H, λ = 254 nm, hexane/2-propanol = 80:20

flow rate = 1.0 mL/min

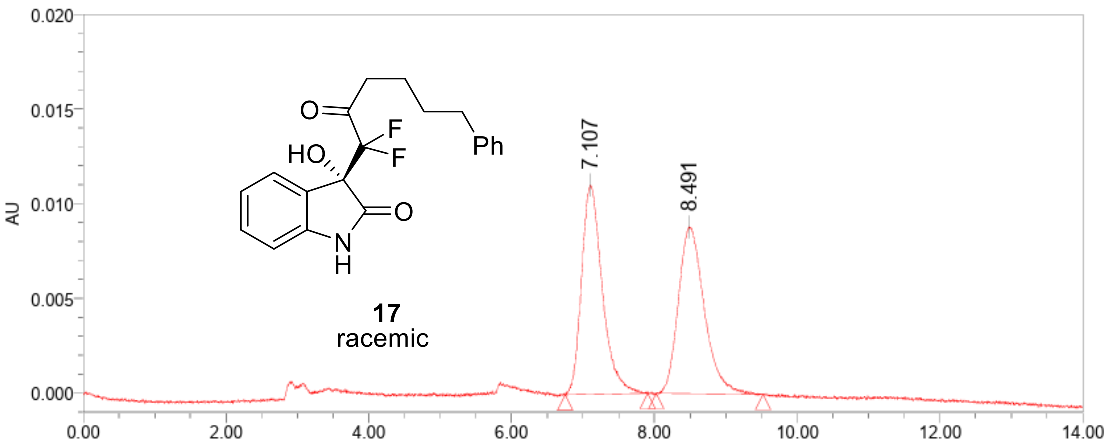

Entry	RT<br>min	Height<br>mV	Area<br>mV.sec	% Area<br>%
1	7.107	11065	223508	50.24
2	8.491	8799	221333	49.76

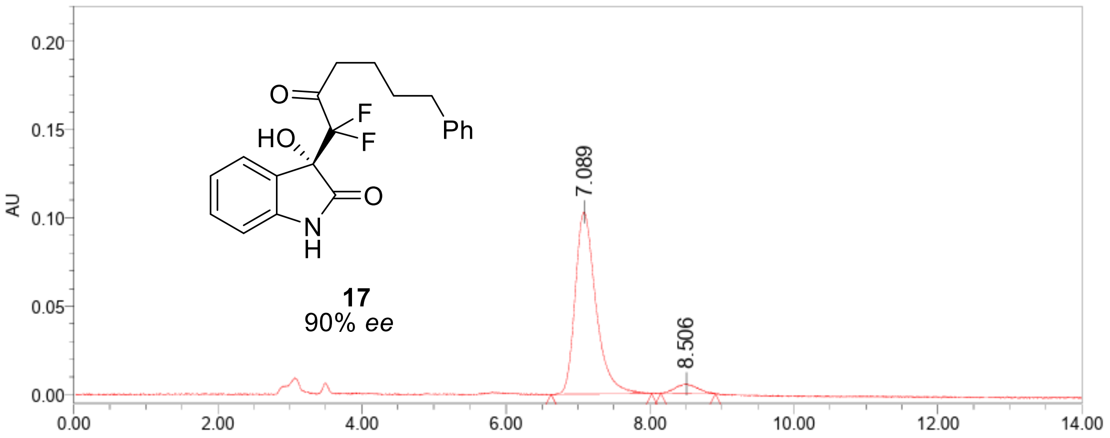

Entry	RT<br>min	Height<br>mV	Area<br>mV.sec	% Area<br>%
1	7.089	103180	2066114	94.93
2	8.506	5265	110332	5.07

Condition: Daicel Chiralpak IA, λ = 254 nm, hexane/2-propanol = 70:30

flow rate = 1.0 mL/min

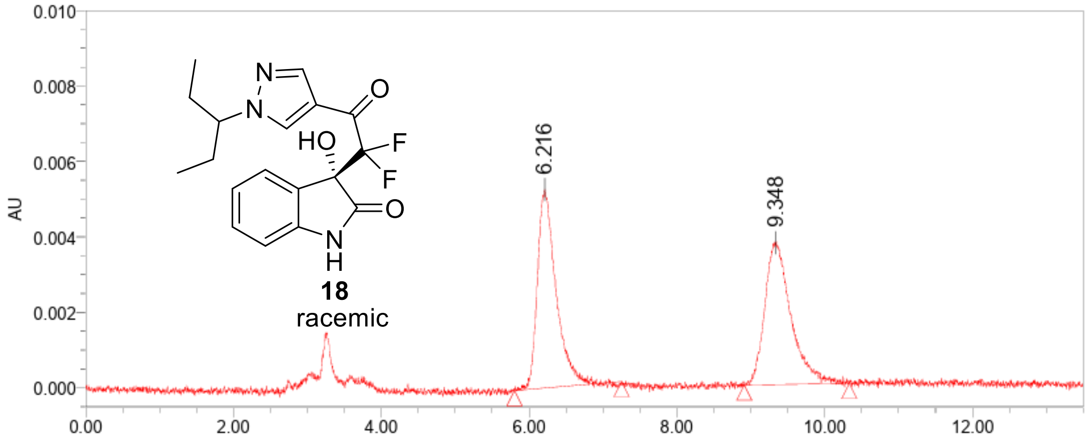

Entry	RT<br>min	Height<br>mV	Area<br>mV.sec	% Area<br>%
1	6.216	5246	92870	50.02
2	9.348	3783	92806	49.98

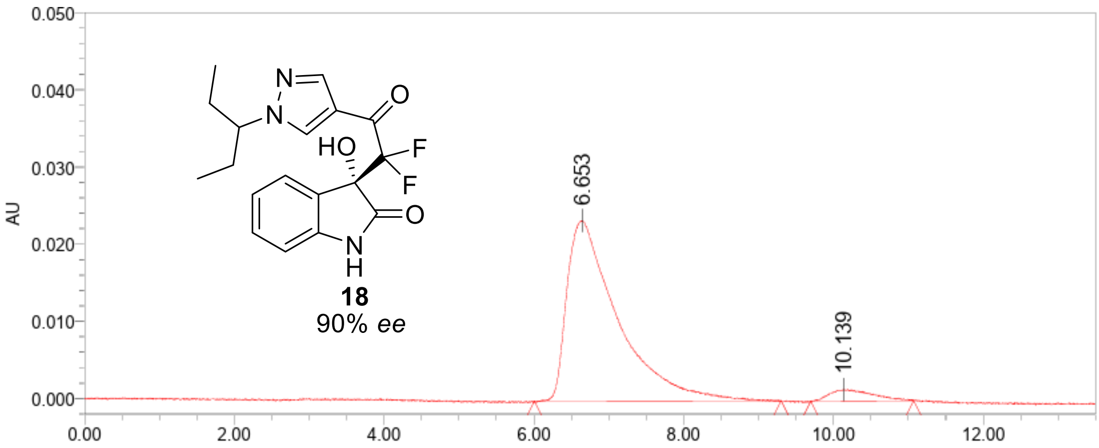

Entry	RT<br>min	Height<br>mV	Area<br>mV.sec	% Area<br>%
1	6.653	23382	1101409	94.91
2	10.139	1501	59060	5.09

Condition：Daicel Chiralpak IA, λ = 254 nm, hexane/2-propanol = 80:20

flow rate = 1.0 mL/min

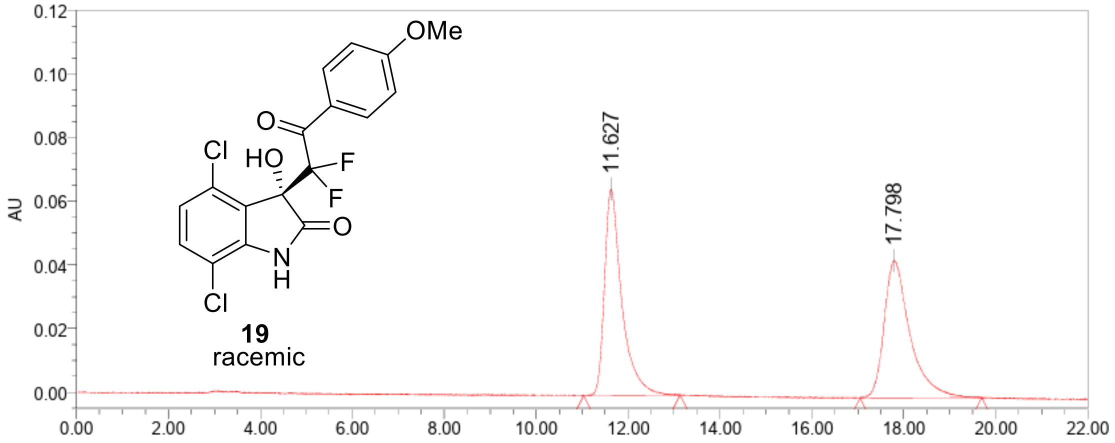

Entry	RT<br>min	Height<br>mV	Area<br>mV.sec	% Area<br>%
1	11.627	64881	1690945	50.04
2	17.798	43096	1688184	49.96

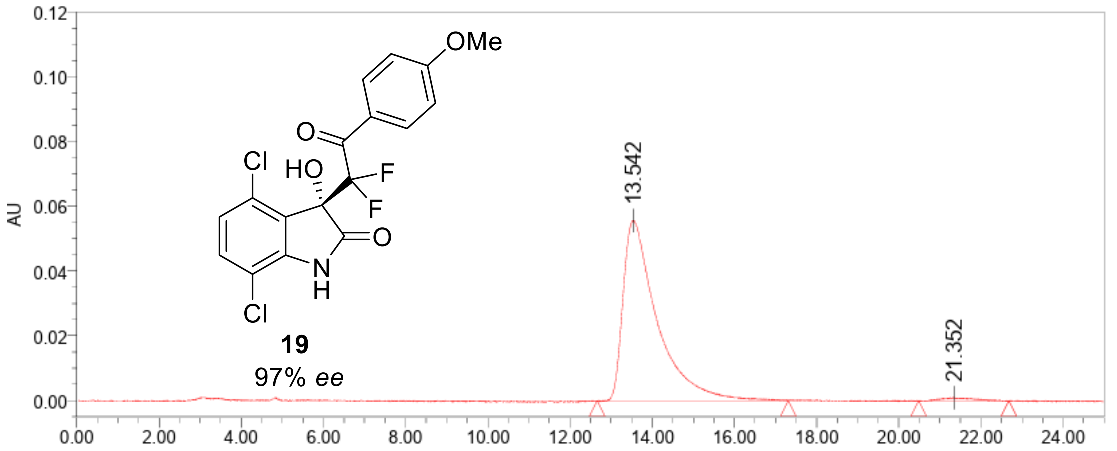

Entry	RT<br>min	Height<br>mV	Area<br>mV.sec	% Area<br>%
1	13.542	55668	3189819	98.53
2	21.352	937	47702	1.47

8. Single-Crystal X-ray Diffraction of 4a

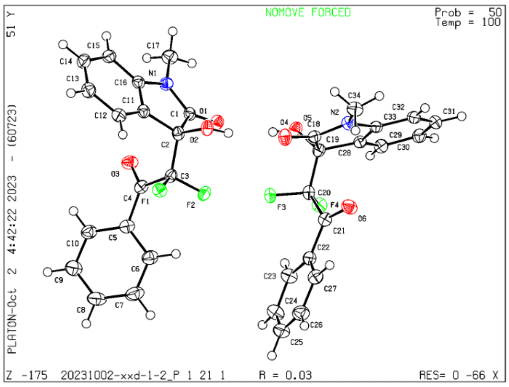

=

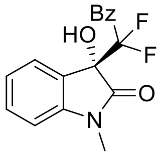

4a
CCDC 2298623

Table S5: Crystal data and structure refinement for **4a**.

Identification code	0231002-xxd-1-2_auto
Empirical formula	$C_{17}H_{13}F_2NO_3$
Formula weight	317.28
Temperature/K	100.00(10)
Crystal system	monoclinic
Space group	P 21
a/Å	8.74460(10)
b/Å	11.02550(10)
c/Å	15.3917(2)
α/°	90
β/°	93.0590(10)
γ/°	90
Volume/Å^3	1481.85(6)
Z	4
ρcalcg/cm^3	1.422
μ/mm^{-1}	0.977
F(000)	656.0
Crystal size/mm^3	0.2x0.15x0.1
Radiation	Cu Kα (λ = 1.54184)
2Θ range for data collection/°	9.83 to 150.93
Index ranges	$-10 \le h \le 10$, $-13 \le k \le 13$, $-19 \le l \le 19$
Reflections collected	16957
Independent reflections	5830 [Rint =0.0344, Rsigma = 0.0352]
Data/restraints/parameters	5830/0/420
Goodness-of-fit on F^2	1.032
Final R indexes [I>=2σ (I)]	R1 = 0.0287, wR2 = 0.0690
Final R indexes [all data]	R1 = 0.0302, wR2 = 0.0700
Largest diff. peak/hole / e Å^{-3}	0.21/-0.18

8. Single-Crystal X-ray Diffraction of 7a

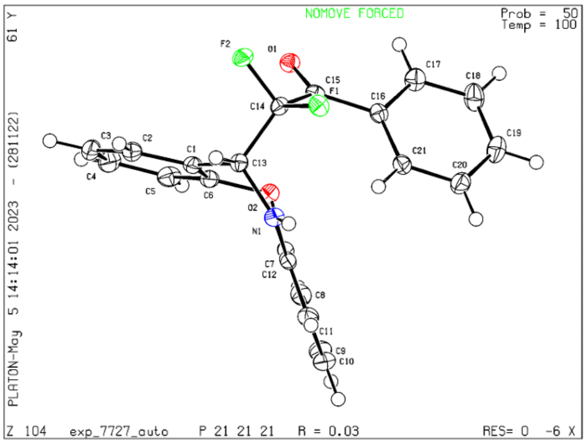

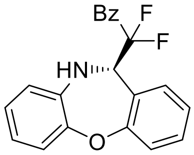

7a
CCDC 2298622

Table S6: Crystal data and structure refinement for **7a**.

Identification code	exp_7727_auto
Empirical formula	$C_{21}H_{15}F_2NO_2$
Formula weight	350.33
Temperature/K	100.00(10)
Crystal system	orthorhombic
Space group	P 21
a/Å	6.16104(5)
b/Å	15.56461(15)
c/Å	18.26285(18)
α/°	90
β/°	90
γ/°	90
Volume/Å^3	1751.30(3)
Z	4
ρcalcg/cm^3	1.329
μ/mm^{-1}	0.839
F(000)	724.0
Crystal size/mm^3	0.2x0.05x0.05
Radiation	Cu Kα (λ = 1.54184)
2Θ range for data collection/°	4.84 to 151.44
Index ranges	$-7 \le h \le 5$, $-19 \le k \le 19$, $-22 \le l \le 21$
Reflections collected	15819
Independent reflections	3488 [Rint =0.0203, Rsigma = 0.0115]
Data/restraints/parameters	3488/0/236
Goodness-of-fit on F^2	1.052
Final R indexes [I>=2σ (I)]	R1 = 0.0257, wR2 = 0.0660
Final R indexes [all data]	R1 = 0.0258, wR2 = 0.0661
Largest diff. peak/hole / e Å-3	0.27/-0.25

9. References:

[1] N. Tanbouza, L. Caron, A. Khoshoei, T. Ollevier, *Org. Lett.* **2022***, 24,* 2675.

[2] X. Xie, M. Bao, K. Chen, X. Xu, W. Hu, *Org. Chem. Front.* **2022***, 9,* 2102.

[3] G. Dong, M. Bao, X. Xie, S. Jia, W. Hu, X. Xu, *Angew. Chem. Int. Ed.* **2021***, 60,* 1992.

[4] Y.-L. Pan, Y.-B. Shao, Z. Liu, H.-L. Zheng, L. Cai, H.-C. Zhang, X. Li, *Org. Chem. Front.* **2022***, 9,* 3990.

[5] Y. -L. Liu, J. Zhou, *Chem. Commun.* **2012***, 48,* 1919.
