# Supplementary material for: Catalytic Asymmetric Difluoroalkylation Using In Situ Generated Difluoroenol Species as the Privileged Synthon
Source: Adv Sci (Weinh). 2024 Feb 6;11(14):2307520. doi: 10.1002/advs.202307520 (PMC11005710; doi:10.1002/advs.202307520)

checkCIF/PLATON report

Structure factors have been supplied for datablock(s) exp_7727_auto

THIS REPORT IS FOR GUIDANCE ONLY. IF USED AS PART OF A REVIEW PROCEDURE FOR PUBLICATION, IT SHOULD NOT REPLACE THE EXPERTISE OF AN EXPERIENCED CRYSTALLOGRAPHIC REFEREE.

No syntax errors found. CIF dictionary Interpreting this report

Datablock: exp_7727_auto

```
Bond precision:   C-C = 0.0024 A          Wavelength=1.54184

Cell:            a=6.16104(5)    b=15.56461(15)    c=18.26285(18)
                 alpha=90        beta=90           gamma=90
Temperature:     100 K

                Calculated                  Reported
Volume          1751.30(3)                  1751.30(3)
Space group     P 21 21 21                  P 21 21 21
Hall group      P 2ac 2ab                   P 2ac 2ab
Moiety formula  C21 H15 F2 N O2             C21 H15 F2 N O2
Sum formula     C21 H15 F2 N O2             C21 H14 F2 N O2
Mr              351.34                      350.33
Dx,g cm-3       1.332                       1.329
Z               4                           4
Mu (mm-1)       0.839                       0.839
F000            728.0                       724.0
F000'           730.52
h,k,lmax        7,19,22                     7,19,22
Nref            3629[ 2106]                 3488
Tmin,Tmax
Tmin'

Correction method= Not given

Data completeness= 1.66/0.96       Theta(max)= 75.723

R(reflections)= 0.0257( 3469)               wR2(reflections)=
                                            0.0661( 3488)
S = 1.052                Npar= 236
```

The following ALERTS were generated. Each ALERT has the format
test-name_ALERT_alert-type_alert-level.
Click on the hyperlinks for more details of the test.

Alert level C

PLAT041_ALERT_1_C Calc. and Reported SumFormula Strings Differ Please Check
PLAT043_ALERT_1_C Calculated and Reported Mol. Weight Differ by .. 1.01 Check
PLAT052_ALERT_1_C Info on Absorption Correction Method Not Given Please Do !
PLAT053_ALERT_1_C Minimum Crystal Dimension Missing (or Error) ... Please Check
PLAT054_ALERT_1_C Medium Crystal Dimension Missing (or Error) ... Please Check
PLAT055_ALERT_1_C Maximum Crystal Dimension Missing (or Error) ... Please Check
PLAT068_ALERT_1_C Reported F000 Differs from Calcd (or Missing)... Please Check
PLAT601_ALERT_2_C Unit Cell Contains Solvent Accessible VOIDS of . 77 Ang**3
PLAT911_ALERT_3_C Missing FCF Refl Between Thmin & STh/L= 0.600 2 Report

Alert level G

FORMU01_ALERT_1_G There is a discrepancy between the atom counts in the
_chemical_formula_sum and _chemical_formula_moiety. This is
usually due to the moiety formula being in the wrong format.
Atom count from _chemical_formula_sum: C21 H14 F2 N1 O2
Atom count from _chemical_formula_moiety:C21 H15 F2 N1 O2
FORMU01_ALERT_2_G There is a discrepancy between the atom counts in the
_chemical_formula_sum and the formula from the _atom_site* data.
Atom count from _chemical_formula_sum:C21 H14 F2 N1 O2
Atom count from the _atom_site data: C21 H15 F2 N1 O2
CELLZ01_ALERT_1_G Difference between formula and atom_site contents detected.
CELLZ01_ALERT_1_G ALERT: Large difference may be due to a
symmetry error - see SYMMG tests
From the CIF: _cell_formula_units_Z 4
From the CIF: _chemical_formula_sum C21 H14 F2 N O2
TEST: Compare cell contents of formula and atom_site data

atom	Z*formula	cif sites	diff
C	84.00	84.00	0.00
H	56.00	60.00	-4.00
F	8.00	8.00	0.00
N	4.00	4.00	0.00
O	8.00	8.00	0.00

PLAT007_ALERT_5_G Number of Unrefined Donor-H Atoms .... 1 Report
PLAT142_ALERT_4_G s.u. on b - Axis Small or Missing .... 0.00015 Ang.
PLAT143_ALERT_4_G s.u. on c - Axis Small or Missing .... 0.00018 Ang.
PLAT791_ALERT_4_G Model has Chirality at C13 (Sohnke SpGr) R Verify
PLAT912_ALERT_4_G Missing # of FCF Reflections Above STh/L= 0.600 50 Note
PLAT961_ALERT_5_G Dataset Contains no Negative Intensities .... Please Check
PLAT978_ALERT_2_G Number C-C Bonds with Positive Residual Density. 13 Info

0 **ALERT level A** = Most likely a serious problem - resolve or explain
0 **ALERT level B** = A potentially serious problem, consider carefully
9 **ALERT level C** = Check. Ensure it is not caused by an omission or oversight
11 **ALERT level G** = General information/check it is not something unexpected

10 ALERT type 1 CIF construction/syntax error, inconsistent or missing data

```
3 ALERT type 2 Indicator that the structure model may be wrong or deficient
1 ALERT type 3 Indicator that the structure quality may be low
4 ALERT type 4 Improvement, methodology, query or suggestion
2 ALERT type 5 Informative message, check
```

It is advisable to attempt to resolve as many as possible of the alerts in all categories. Often the minor alerts point to easily fixed oversights, errors and omissions in your CIF or refinement strategy, so attention to these fine details can be worthwhile. In order to resolve some of the more serious problems it may be necessary to carry out additional measurements or structure refinements. However, the purpose of your study may justify the reported deviations and the more serious of these should normally be commented upon in the discussion or experimental section of a paper or in the "special_details" fields of the CIF. checkCIF was carefully designed to identify outliers and unusual parameters, but every test has its limitations and alerts that are not important in a particular case may appear. Conversely, the absence of alerts does not guarantee there are no aspects of the results needing attention. It is up to the individual to critically assess their own results and, if necessary, seek expert advice.

Publication of your CIF in IUCr journals

A basic structural check has been run on your CIF. These basic checks will be run on all CIFs submitted for publication in IUCr journals (*Acta Crystallographica*, *Journal of Applied Crystallography*, *Journal of Synchrotron Radiation*); however, if you intend to submit to *Acta Crystallographica Section C* or *E* or *IUCrData*, you should make sure that full publication checks are run on the final version of your CIF prior to submission.

Publication of your CIF in other journals

Please refer to the *Notes for Authors* of the relevant journal for any special instructions relating to CIF submission.

PLATON version of 28/11/2022; check.def file version of 28/11/2022

Datablock exp_7727_auto - ellipsoid plot

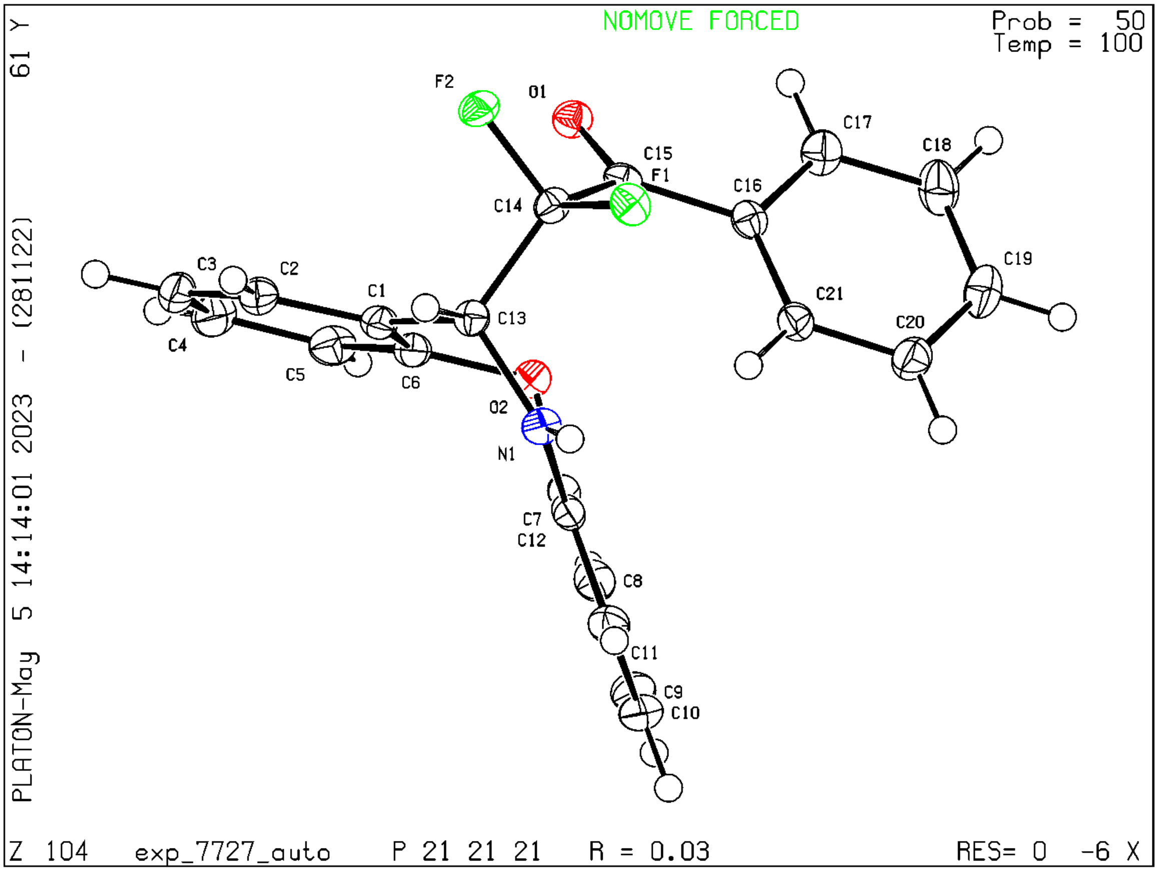

Supplement: Supplementary file 3 — Supporting Information [file ADVS-11-2307520-s002.cif]
